# Determination of Major, Minor and Chiral Components as Quality and Authenticity Markers of *Rosa damascena* Oil by GC-FID

**DOI:** 10.3390/plants12030506

**Published:** 2023-01-22

**Authors:** Justine Raeber, Sina Favrod, Christian Steuer

**Affiliations:** Institute of Pharmaceutical Sciences, Swiss Federal Institute of Technology, 8093 Zurich, Switzerland

**Keywords:** method validation, chiral GC-FID, *Rosa damascena*, essential oils, authenticity control

## Abstract

Rose oil is traditionally produced by the water distillation of *Rosa damascena* and is of high economic value due to the low essential oil yield. It is therefore a common target for adulteration, which can cause harm to consumers. Current standards for authenticity control only consider the analysis of major components and overlook minor quality markers as well as the enantiomeric ratio of terpenes, which have proven useful in originality determination. The aim of this study was the development of two analytical GC-FID methods for the analysis of 21 and 29 rose oil analytes including major, minor and chiral components on a DB-wax and BGB 178 30% CD (chiral) capillary column, respectively. The total run time for both methods was within 60 min. For all target analytes, the % bias at the lower and upper calibration range varied from −7.8 to 13.2% and −13.1 to 5.2% analysed on the DB-wax column and 0.5 to 13.3% and −6.9 to 7.0% analysed on the chiral column. The chiral analysis successfully separated the enantiomers (+/−)-camphene, (+/−)-rose oxide, (+/−)-linalool, (+/−)-citronellol and (+/−)-citronellyl acetate, as well as the diastereomers of citral and β-damascenone. Both methods were applied to the analysis of 10 authentic rose oil samples and the enantiomeric/diastereomeric ratios, as well as the content of major and minor components, were determined. The identity of the analysed components in the authentic samples was further confirmed by GC-MS.

## 1. Introduction

Rose oil is typically used as a flavouring, fragrance and medicinal agent in food, cosmetics and the pharmaceutical industry and is traditionally produced by the water distillation of fresh *Rosa damascena* (*R. damascena*) flowers [1,2]. *R. damascena* contains low amounts of essential oils (EOs) and distillation typically leads to a yield of 0.02%. The production of 1 kg rose oil requires 3.5–4 tons of flowers and, due to the lack of natural and synthetic substitutes, rose oil is traded at high prices, which makes it a lucrative target for adulteration [2,3,4]. Common adulteration techniques include adding individual (natural or synthetic) substitutes such as phenylethanol, citronellol, geraniol, geranyl acetate and linalool or mixing rose oil with other (cheaper) EOs such as palmarosa oil. In some cases, non-volatile components such as vegetable oils are added to the EO [3,5]. Falsified EOs do not only deceive consumers, but can have a severe impact on human health and therefore authenticity control is essential for consumerism [6]. Efforts have been made to identify valuable markers to guarantee rose oil quality and authenticity such as the content ratio of citronellol/geraniol, the enantiomeric excess of, e.g., rose oxides, linalool, citronellol and carvone, as well as the availability of trace substances such as β-damascenone, β-ionone and β-damascene [7,8,9]. High-resolution mass spectrometry (HRMS) has been applied to elucidate and allocate further potential components as quality markers; the identity verification of compounds, however, is still challenging and a limiting factor for their applicability as markers [10]. In addition to the identification of individual markers for authenticity control, more holistic approaches such as the study of isotope mass spectrometry to determine the δ13C values or enantio-multidimensional gas chromatography (MDGC) have been applied to identify fingerprints for rose oil origin [11]. Although MDGC can provide higher resolution power in the separation of samples as complex as EOs, such analyses are time-consuming, expensive and acquired retention times cannot be compared to literature reference values. Furthermore, acquired data can be highly complex and difficult to interpret [12]. The analysis of chiral components in natural products, especially, has been shown to be useful in authenticity control due to enantioselective synthesis in species [13]. Although new advancements and insights on the quality assurance of rose oil have been made, they have not been incorporated into international standards. The ISO norm 9842:2003 still only describes requirements based on appearance, colour, smell, density, refraction, and optical rotation. The chromatographic profile is limited to ethanol, citronellol, nerol, geraniol, β-phenylethanol, heptadecane, nonadecane and heneicosane and does not include any (minor) quality markers [14]. Depending on the study, between 38–132 individual compounds have been identified for rose oil and only a fraction is included in the ISO norm analysis [7,8,15]. The main components of rose oil are geraniol, nerol, phenylethanol, eugenol, methyleugenol farnesol, linalool and aliphatic hydrocarbons, while the rest only appears in trace amounts [8]. These minor compounds, however, are often viewed as important contributors to the unique aroma of rose oil. While the major compounds geraniol, nerol, citronellol and linalool can make up over 60% of the EO, factors such as genotype, growth environment and conditions, climate, harvesting time, storage and processing methods can lead to observable fluctuations in EO composition [16,17]. The aim of the study was to develop a convenient method for gas chromatography (GC) coupled to a flame ionisation detector (GC-FID), which takes major and minor components of rose oil, as well as their stereochemistry, into account. In accordance with the ISO norm 9842:2003, a FID was selected, which provides a broad detection range and robustness [14,18]. Extending current standard analysis methods, which can furthermore be easily applied in most analytical laboratories, has the potential to improve the quality and originality assessment and can increase consumer safety.

## 2. Results and Discussion

### 2.1. Method Validation for Polar GC-FID Analysis

**Figure 1:**
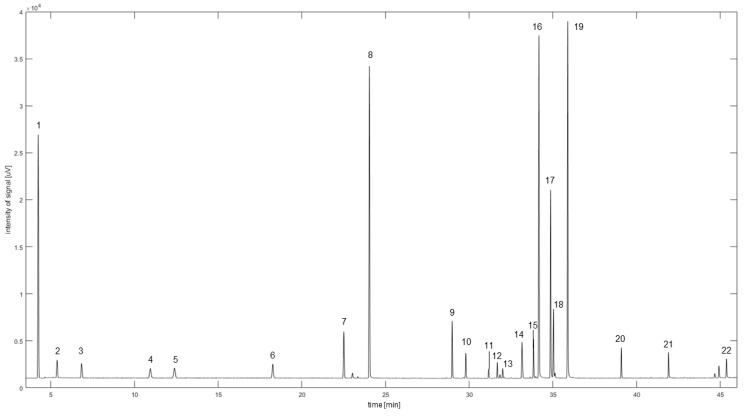
Chromatogram for the analysis of 22 rose oil components on DB-wax column.

Baseline separation was achieved for all 22 analytes on the DB-wax column with a Rs > 2.4 (see Table 1, Figure 1, Supplementary Materials) and a total run time of 60 min. LoD and LoQ values ranged for all analytes from 0.96–4.55 and 2.90–13.78 ng, respectively. The calculated RI values were in good agreement with the literature values and the bias [19,20,21,22,23,24,25,26,27,28,29,30]; RSD_R_ and RSD_T_ did not exceed ± 20% for QC_high_, QC_med_ and QC_low_ (Table 1). The QC stability was observed throughout the 14-day validation time span and the variation was within a 20% deviation limit (see Supplementary Material, Figure S1). QC samples measured on a different instrument for authentic sample quantification were in accordance with the results obtained during validation. These observations concluded that the method was at least reliable for data acquisition throughout a two-week time span and only required a single calibration at the beginning. Furthermore, the same calibration equations from the validation could be used for samples measured on different instruments. Additionally, using our in-house code for the integration resulted in similar results as Chromeleon.

Selectivity was demonstrated by spiking rose, lavender, rosemary, lemon and caraway oil. Furthermore, spiking experiments are well accepted in analytical method development for determining the accuracy of the analytical method [31]. The results are presented as the RE. EOs from other plant species were selected to demonstrate and test method transferability and selectivity. Spiked rose oil samples showed a good recovery, with deviations below 20% (Supplementary Materials, Table S1). Most analytes also showed excellent recovery with a few exceptions. The spiked amount of limonene in lemon oil was, for example, overestimated. This might be due to the spiked concentrations being much lower than the occurring concentrations of limonene in lemon oil. Small deviations in the peak intensity can already cause large fluctuations in the recovery. The method nevertheless showed an overall good transferability to other EOs with the limitation that, for some analytes, a different range and calibration must be selected. For this experiment, only the expected intermediate concentration of the analytes in rose oil was tested. A further spiking experiment was conducted to control for the selectivity of analytes at QC_high_ and QC_low_ concentrations in authentic rose oil. The results can be viewed in Table S1 in the Supplementary Materials. All analytes had RE values in an acceptable range. Method robustness was confirmed by testing two different settings for the split ratio, FID and inlet temperature, and flow as well as the temperature ramp [32]. As predicted, slight fold changes in the peak area for the changed split ratio, FID and inlet temperature were observed. Changing the flow and temperature ramp caused shifts in the retention times. The results can be viewed in detail in Table S3 in the Supplementary Materials.

### 2.2. Method Validation for Chiral GC-FID Analysis

**Figure 2:**
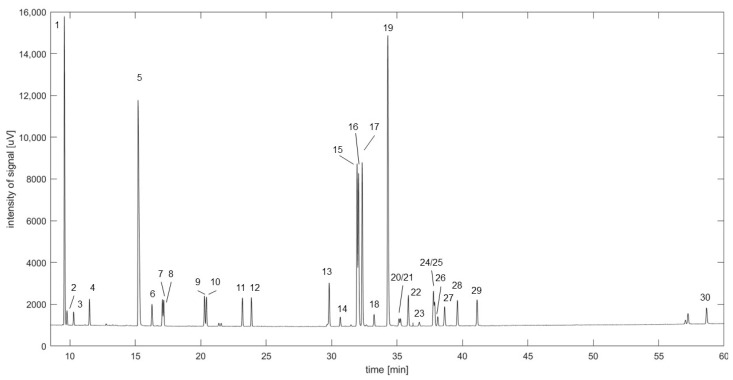
Chromatogram for the analysis of 30 rose oil components on the chiral column.

Baseline separation was achieved for 30 analytes on the chiral column with a R_s_ > 0.7 (see Figure 2, Supplementary Materials) and a total run time of 65 min. According to the Gaussian distribution, a R_s_ of 0.7 leads to a peak overlap of around 8%, while complete peak separation is expected at R_s_ values > 1.5. Therefore, not all analytes achieved complete separation. However, validation and spiking experiments showed that the analytes were still quantifiable in a repeatable manner [31]. The calculated RI values are presented in Table 2; however, due to the scarcity of literature RI values for natural products analysed on chiral columns, no literature comparison could be conducted [33]. The bias, RSD_R_ and RSD_T_ did not exceed ± 15% for QC_high_, QC_med_ and QC_low_ (See Table 2). LoD and LoQ values ranged for all analytes from 0.03–1.28 and 0.10–3.75 ng, respectively.

Spiking experiments produced similar results on the chiral column as the analysis on the DB-wax column. The RE did not exceed 20%. The recovery was excellent for spiked rose oils. Oils from other species showed mixed results. Lavender oil contains linalool in higher amounts than rose oil does and the RE for one enantiomer was heavily overestimated, while the other enantiomeric form showed good results [34]. Terpene production in plants occurs enantioselectively and lavender oil appears to mostly consist of one enantiomer of linalool [9]. Linalool was spiked in a too-low concentration for it to be reliably recovered. Limonene was also added in a too-low concentration to lemon oil for recovery and, furthermore, the limonene peak was so intense that it overlapped with p-cymene. Caraway oil showed an overestimation for limonene and p-cymene spiked contents, due to high limonene concentrations. In general, the analytical method can be transferred very well to a variety of other EOs if the calibration range is adjusted. QC_high_ and QC_low_ concentrations were spiked with authentic rose oil. The RE was below 20% for all analytes as well. The results of the spiking experiments can be viewed in Table S2 in the Supplementary Materials. The QC stability was tested and resulted in identical conclusions as for the polar GC-FID analysis (see Supplementary Materials, Figure S2). Method robustness was confirmed by testing two different settings for the split ratio, FID and inlet temperature, and pressure as well as the temperature ramp. As predicted, slight fold changes in the peak area for the changed split ratio, FID and inlet temperature were observed. Changing the pressure and temperature ramp caused shifts in retention. The results can be viewed in detail in Table S4 in the Supplementary Materials.

### 2.3. Measurement of Authentic Rose Oil Samples

**Figure 3:**
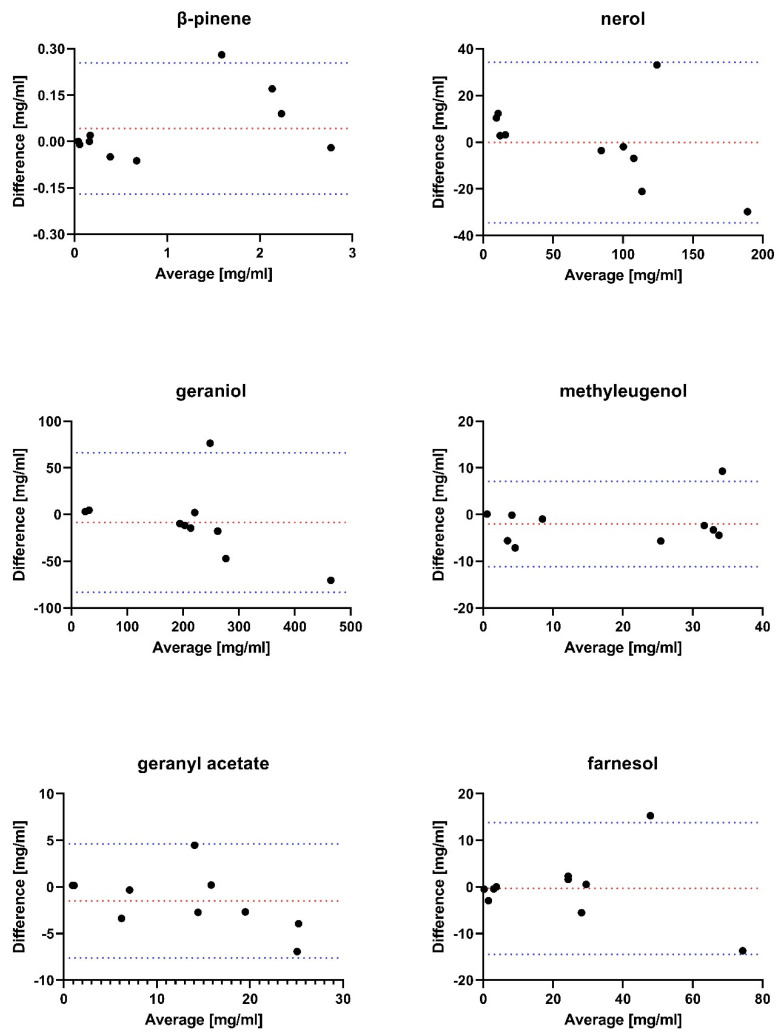
Bland-Altman plots for 6 major rose oil components. The x-axis depicts the measured average concentration determined from both analytical methods; the y-axis presents the difference between both. A total of n = 10 rose oil samples were analysed. Red dotted lines indicate the bias and blue dotted lines the 95% limits of agreement.

A total of 10 authentic rose oil samples were analysed on the DB-wax and chiral column with GC-FID. The results for the quantification of both methods were compared using Bland–Altman plots for six major compounds that were not separable stereoisomers (Figure 3). The results for the quantification of the authentic rose oils can be viewed in Tables S5 and S6 in the Supplementary Materials.

Almost all measurements were within the 95% limits of agreement. Consequently, the quantification for these analytes resulted in equal values for both methods. Since pure reference compounds for isomers were not commercially available and the literature RI values on chiral GC columns were non-existent, a semi-quantitative approach was chosen by comparing the EE and DE instead (Supplementary Materials, Table S6) and compound identification was neglected. DE and EE determination showed that either one stereoisomer was present or one of the two appeared dominantly. This observation supports the fact that specific plants can produce terpenes stereoselectively [5,13]. (−)-rose oxide, for example, predominantly appeared in authentic *R. damascena* samples while *P. graveolens* samples (a common adulterant used in rose oil) showed a slight excess of 0.15 for (+)-rose oxide. Rose oil sample number 1 only contained (+)-rose oxide, which might be an indication of adulteration. However, it has been observed that the enantiomeric ratio can change over time depending on the storage conditions. Especially high temperatures can have an effect on the ratio, but it does not seem to have the same impact on all isomers present [35]. Further investigations concerning the stability of isomer forms have to be conducted to exclude these types of effects. Another possible discrimination for *P. graveolens* and *R. damascena* can be made by looking at the citronellyl acetate enantiomeric ratio. Only one isomer form seems to appear in *R. damascena* samples, while *P. graveolens* contains both, but one in a slight excess. Interestingly, both diastereomers of β-damascenone were present in *P. graveolens* samples, while only one isomer was mostly observed for *R. damascena* oils. Nerol and geraniol were identified as the main constituents of rose oil, as expected [17,36]. All in all, further studies on a bigger sample population of rose oil have to be conducted to identify reliable markers for the authenticity control of rose oils, that are not only able to exclude adulteration but can furthermore differentiate sample origin and sample age, respectively.

### 2.4. Polar GC-MS Analysis

MS spectra acquired from reference compounds were compared to spectra recorded from authentic rose oil samples to exclude possible interferences and confusion with other compounds that might elute at the same time. The reference spectra, as well as spectra from authentic samples, can be viewed in the Supplementary Materials. The results are coherent with observations made after polar GC-FID analysis. However, β-damascenone was not detected in any of the samples with GC-MS, which contradicts the findings of the polar GC-FID analysis but is in good agreement with the results of the chiral GC-FID analysis. Sensitivity, LoD and LoQ were not determined for the GC-MS analysis. However, FID as a detection method is characterised by its high sensitivity and high linear range and might surpass that of the MS used in this study [37]. No MS spectra were acquired for methyleugenol, eugenol and farnesol from *P. graveolens* samples and quantitative results from the polar GC-FID analysis resulted in much lower concentrations compared to *R. damascena* samples, while the chiral GC-FID study did not detect farnesol in *P. graveolens* samples. Generally, the described GC-FID methods performed in a similar manner as GC-MS concerning univocally identifying and discriminating the selected analytes. While MS analysis adds an additional dimension for identification by producing characteristic fragment spectra, its maintenance and purchase are expensive, time-consuming and not feasible for all laboratories. FID, moreover, stands out by its broad detection range, robustness and sensitivity. Furthermore, it does not require extensive training as MS does. We propose from our observations that, by combining RI acquired retention times from both a chiral and polar GC-FID analysis, similar results for identification can be obtained while being cost-effective, robust and practical in use.

## 3. Conclusions

Two GC-FID methods for the analysis of rose oil were developed using a polar and chiral GC column with a total run time of around 60 min. A total of 21 and 29 analytes were included in the analysis on the polar and chiral columns, respectively.

The method was validated and proven to be specific, precise and accurate for all analytes. Spiking experiments showed good recovery and disclosed transferability to other EOs. Furthermore, both methods were robust and exhibited similar results regarding identification using MS, while being cost-effective and requiring little practical training, which makes the application feasible in most laboratories. Ten authentic rose oil samples were fully quantified by both methods and possible markers for the discrimination between *P. graveolens*, a common adulterant, and *R. damascena* were identified. Determining the enantiomeric ratio can be a useful tool for authenticity and originality testing and should be considered in future standardisation norms. We propose that current ISO norms to qualify the genuineness of *R. damascena* oil are insufficient and can be easily evaded, while imitating enantiomer ratios is an expensive undertaking and not a cost-effective method to defraud. In addition, enantiomeric ratios provide information about the origin and species of a plant due to enantiomeric-specific synthesis.

## 4. Material and Methods

### 4.1. Chemicals and Reagents

Phenylethanol, limonene and α-terpinene (pract.) were purchased from Fluka Chemie GmbH (Buchs, Switzerland). Camphene (95%), β-damascone (>95%), citronellyl acetate (>95%), β-damascenone natural, (+)-β-pinene (analytical standard), (−)-β-pinene (99%), (+)-α-pinene (≥99%), (−)-α-pinene (analytical standard), farnesol (95%), geranyl acetate (>99%), linalool (97%), p-cymene (99%), rose oxide (cis/trans mixture) and cis-3-hexen-1-ol (internal standard (IS), 98%) were purchased from Sigma Aldrich (St. Louis, MO, USA). Eugenol and β-caryophyllene were obtained from Systema Natura GmbH (Flintbek, Germany). Citral (cis/trans mixture, >98%) and neryl acetate (>95%) were purchased from TCI Chemical (Eschborn, Germany). Citronellol (95%), geraniol (99%) and nerol (97%) were obtained from Acros Organics (Geel, Belgium), and methyleugenol and n-heptane (99.9%) were purchased from Carl Roth GmbH (Karlsruhe, Germany) and VWR chemicals (Schlieren, Switzerland), respectively. Authentic rose oil samples and other EOs were purchased in Swiss pharmacies and online. All samples were stored at room temperature and in brown glass vials. Helium 6.0, nitrogen 6.0 and hydrogen 5.0 gas were purchased from PanGas (Dagmersellen, Switzerland).

### 4.2. Polar GC-FID Analysis

The GC-FID analysis was performed using a Thermo Fisher Scientific Focus GC (Thermo Fisher Scientific, Waltham, MA, USA) equipped with a DB-wax capillary column (Agilent J&W, Santa Clara, CA, USA) with the following dimensions: length 30 m × 0.25 mm i.d., film thickness 0.25 μm. The settings were as follows: inlet temperature 220 °C, detector temperature 250 °C, constant flow at 2.0 mL/min, gas safe flow 20 mL/min, split flow 100 mL/min and split ratio 1:50. The oven temperature was held at 45 °C for 15 min and afterwards increased by 5 °C/min up to 220 °C. The maximal temperature was held for another 9 min. Helium 6.0 was used as a carrier gas. The injection volume was 1 μL.

### 4.3. Chiral GC-FID Analysis

The chiral GC-FID analysis was performed using a Thermo Fisher Scientific Focus GC (Thermo Fisher Scientific, Waltham, MA, USA) equipped with a BGB 178 30% CD capillary column (BGB Analytik AG, Boeckten, Switzerland). The capillary column consists of 30% 2,6-diethyl-6-tert-butyldimethylsilyl-beta-cyclodextrin dissolved in a combination of 15% phenyl- and 85% methylpolysiloxane. The dimensions of the column were as follows: length 25 m × 0.25 mm i.d., film thickness 0.25 μm. The settings were as follows: inlet temperature 250 °C, detector temperature 260 °C, constant pressure at 100 kPa, gas safe flow 20 mL/min, split flow 140 mL/min and split ratio 1:60. The oven temperature was held at 60 °C for 2 min and afterwards increased by 2 °C/min up to 180 °C. The maximal temperature was held for another 3 min. Helium 6.0 was used as a carrier gas. The injection volume was 1 μL.

### 4.4. Polar GC-MS Analysis

MS spectra were acquired for 10 authentic rose oil samples and compared to spectra acquired from reference samples. Spectra can be viewed in the Supplementary Materials. For the analysis, a Trace GC Ultra connected to a TriPlus autosampler and coupled to a DSQ II MS (Thermo Fisher scientific, Waltham, MA, USA) was used. Data acquisition was performed using XCalibur (Thermo Fisher scientific, version 2.2 SP1.48). The GC-MS was equipped with the DB-wax capillary column and followed the temperature program described in Section 2.2. Settings for the MS were as follows: the ion source was set to 220 °C, MS transferline 250 °C, mass range 50–650 Da, ionisation mode was set positive at 70 eV and the scan rate was 500 amu/s.

### 4.5. Data Processing and Statistical Analysis

Data acquisition was performed using ChromCard (Thermo Fisher scientific, version 2.9) and peak integration was conducted using Matlab (Version R2021b 9.11.0.176998) for samples acquired with a Thermo Scientific Focus GC. The code used for integration can be found in the Supplementary Materials. Samples analysed on the Thermo Scientific Trace 1600 GC were processed using Chromeleon (Thermo Scientific, version 7.3.1.). All calculations were performed using Microsoft Excel 2016 (Version: 16.0.5305.1000) and statistical analysis was conducted using GraphPad Prism (Version 9.2.0). Graphical abstract was carried out using BioRender.

### 4.6. Method Validation

The analytical method was validated according to the International Council for Harmonisation of Technical Requirements for Registration of Pharmaceuticals for Human Use (ICH) Q2(R2) guidelines [38]. Six calibration standards (Cal) and three quality control (QC) samples at different concentrations (QC_high_, QC_med_, and QC_low_) were prepared by serial dilution of separate stock solutions containing 7.3 mM (−)-β-pinene, 88.1 mM (+)-α-pinene, 7.3 mM α-terpinene, 4.9 mM β-caryophyllene, 5.2 mM β-damascone, 7.3 mM camphene, 6.6 mM citral, 128.0 mM citronellol, 5.0 mM citronellyl acetate, 105.1 mM β-damascenone, 7.3 mM limonene, 12.2 mM eugenol, 11.2 mM methyleugenol, 9.0 mM farnesol, 129.7 mM geraniol, 10.2 mM geranyl acetate, 19.4 mM linalool, 64.8 mM nerol, 10.2 mM neryl acetate, 7.5 mM p-cymene, 7.5 mM phenylethanol and 25.9 mM rose oxide in n-heptane. Cis-3-hexen-1-ol was added as an internal standard (IS) with a final concentration of 10 mM to each Cal and QC. For accuracy and precision Cal and QC samples were analysed on 5 separate days over a course of 2 weeks. QC samples were measured in duplicates. Accuracy was determined as a bias; intra-day and inter-day imprecision were calculated as the relative standard deviation (RSD_R_ and RSD_T_, respectively) [39]. Specificity was demonstrated by spiking authentic rose oil samples and four different EO samples with the analytes at intermediate levels of the calibration range. A further spiking experiment was conducted by adding QC_high_ and QC_low_ level concentrations to an authentic rose oil sample. The recovery effect (RE) was determined for the spiking experiments (Formula (1)) [40]. Retention index (RI) was determined by analysing a homologous series of n-alkanes (C8–C20, C21–C40, analytical standard) purchased from Sigma Aldrich (St. Louis, MO, USA). The RI was calculated according to the van den Dool and Kratz equation [41]. Peak resolution (Rs) was determined according to European Pharmacopeia (Ph. Eur.) 11.2 [42]. Limit of detection (LoD) and limit of quantification (LoQ) were calculated based on the standard deviation of the response and the slope of the calibration curve [38]. The method was tested for robustness by changing the split ratio, flow, detector temperature, inlet temperature and temperature ramp of the established methods. Furthermore, the method was transferred to different instruments.

(1) $$RE\;\left[\%\right]=\frac{conc_{experimental\;spiked\;sample}-conc_{experimental\;sample}}{conc_{spiking\;}}\times 100$$

### 4.7. Quantification

Authentic samples were prepared as a 1:10 and 1:500 dilution with a final concentration of 10 mM IS to quantify minor and major components. Analysis was performed using a Thermo Scientific Trace 1600 GC (Thermo Fisher Scientific, Waltham, MA, USA) equipped with both the polar and chiral column. QC_high_, QC_med_ and QC_low_ samples were analysed at the beginning and the end of the run and results were compared to those from the validation days. For samples measured on the chiral column, an enantiomeric/diastereomeric excess (EE/DE) (Formula (2)) was calculated by forming a ratio between the peak areas (PA) of major and minor enantiomers [13].

(2) $$EE=\frac{PA\;of\;major\;enantiomer-PA\;of\;minor\;enantiomer}{PA\;of\;major\;enantiomer+PA\;of\;minor\;enantiomer}$$

## Figures and Tables

**Table 1:** Retention times and results for the validation of 22 rose oil compounds analysed on a DB-wax column with GC-FID. RI_Cal_ depicts the calculated RI values, while RI_Lit_ are the ones obtained from literature.

#	Analyte	RT [min]	Range [mg/mL]	LoD/LoQ [ng]	RI_Cal_/RI_Lit_ [19,20,21,22,23,24,25,26,27,28,29,30]	QC_high_			QC_med_			QC_low_		
						Bias [%]	RSD_R_ [%]	RSD_T_ [%]	Bias [%]	RSD_R_ [%]	RSD_T_ [%]	Bias [%]	RSD_R_ [%]	RSD_T_ [%]
**1**	**α-pinene**	4.24 ± 0.00	0.0062–0.6202	0.96/2.90	1013/1013	5.2	9.4	9.5	4.4	7.4	8.2	13.2	4.0	5.1
**2**	**camphene**	5.37 ± 0.00	0.0011–0.0545	1.30/3.94	1048/1053	−6.2	8.6	8.4	2.3	9.9	9.4	3.9	9.9	10.8
**3**	**β-pinene**	6.84 ± 0.00	0.0006–0.0561	1.27/3.85	1096/1096	2.4	7.8	8.8	1.8	9.6	8.5	11.2	8.8	8.4
**4**	**α-terpinene**	10.95 ± 0.00	0.0012–0.0618	1.48/4.49	1159/1159	−2.9	7.0	10.0	−0.8	10.4	14.1	4.0	10.8	10.1
**5**	**limonene**	12.38 ± 0.00	0.0012–0.0619	1.53/4.46	1166/1176	2.4	11.8	13.1	−0.2	10.1	15.3	6.8	9.5	9.2
**6**	**p-cymene**	18.26 ± 0.00	0.0011–0.0555	1.42/4.31	1264/1268	2.1	12.1	12.6	−2.6	12.9	12.0	7.2	6.4	7.1
**7**	**(+/−)-rose oxide**	22.51 ± 0.00	0.0020–0.2004	1.65/4.99	1344/1339	1.1	11.4	12.5	3.5	11.5	13.1	9.9	7.0	8.8
**8**	**cis-hexen-1-ol**	24.04 ± 0.00	0.0033–0.1320	NA	NA	NA	NA	NA	NA	NA	NA	NA	NA	NA
**9**	**linalool**	28.98 ± 0.00	0.0013–0.0533	1.89/5.71	1383/1380	−13.1	12.2	9.9	−14.0	9.2	10.2	−7.1	13.6	11.9
**10**	**β-caryophyllene**	29.79 ± 0.00	0.0203–1.0143	2.59/7.84	1550/1548	2.1	14.6	11.0	2.1	16.3	13.2	1.2	9.4	12.5
**11**	**β-damascenone**	31.16 ± 0.00	0.0027–0.0542	1.80/5.44	1582/1586	3.7	14.2	10.9	0.4	10.9	10.0	−0.9	8.3	12.3
**12**	**citronellyl acetate**	31.68 ± 0.00	0.0013–0.0644	2.71/8.21	1636/1803	3.1	14.0	11.0	1.0	14.3	11.0	−3.6	4.7	13.1
**13**	**citral**	32.00 ± 0.00	0.0022–0.0874	2.40/7.27	1659/1658	−0.9	9.2	8.8	1.7	14.0	11.6	−7.8	14.3	16.0
**14**	**neryl acetate**	33.15 ± 0.00	0.0063–0.1013	1.94/5.87	1671/1678	2.6	10.4	11.2	3.8	11.8	13.1	2.7	8.3	9.7
**15**	**geranyl acetate**	33.84 ± 0.00	0.0099–0.7951	1.94/5.93	1722/1719	3.4	13.2	10.1	3.3	17.3	13.3	0.5	10.7	8.1
**16**	**citronellol**	34.16 ± 0.00	0.0050–0.4038	1.73/5.24	1754/1746	4.1	10.9	8.9	2.9	15.3	13.6	5.9	10.0	10.3
**17**	**nerol**	34.86 ± 0.00	0.0012–0.0970	2.18/6.61	1768/1755	3.0	9.4	10.1	3.4	15.1	13.5	4.9	10.1	8.5
**18**	**phenylethanol**	35.03 ± 0.00	0.0100–0.8006	1.84/5.58	1800/1798	4.4	7.4	8.9	7.9	11.1	9.6	9.2	6.1	5.7
**19**	**geraniol**	35.88 ± 0.00	0.0023–0.0918	1.99/6.02	1806/1875	4.0	10.5	9.7	3.3	15.2	13.5	6.4	10.2	10.9
**20**	**methyleugenol**	39.09 ± 0.00	0.0025–0.1228	3.06/9.28	1848/1841	3.5	13.0	10.5	0.5	12.0	14.2	−1.0	7.5	9.6
**21**	**eugenol**	41.91 ± 0.00	0.0025–0.1272	3.19/9.68	2008/2001	3.6	10.8	11.7	4.3	11.4	12.4	2.4	8.6	10.8
**22**	**farnesol**	45.37 ± 0.00	0.0062–0.6202	4.55/13.78	2161/2167	1.4	9.7	9.9	−2.0	5.2	9.7	2.5	18.0	13.8

**Table 2:** Retention times and results for the validation of 30 rose oil compounds analysed on the chiral column with GC-FID.

#	Analyte	RT [min]	Range [mg/mL]	LoD/LoQ [ng]	RI_Cal_	QC_high_			QC_med_			QC_low_		
						Bias [%]	RSD_R_ [%]	RSD_T_ [%]	Bias [%]	RSD_R_ [%]	RSD_T_ [%]	Bias [%]	RSD_R_ [%]	RSD_T_ [%]
**1**	**(+)-α-pinene**	9.58 ± 0.00	0.0062–0.6202	0.35/1.07	927	−1.3	2.5	4.9	−1.5	2.1	3.8	10.4	2.4	5.3
**2**	**(+/−)-camphene**	9.77 ± 0.00	0.0028–0.0566	0.63/1.90	931	7.0	4.5	4.5	0.6	5.1	4.5	5.6	5.5	6.0
**3**	**(+/−)-camphene**	10.27 ± 0.00	0.0028–0.0566	0.59/1.79	942	−3.6	4.2	4.7	−6.8	2.3	6.2	0.5	4.3	8.6
**4**	**(−)-β-pinene**	11.49 ± 0.00	0.0028–0.0561	0.60/1.81	967	4.1	2.6	6.8	2.3	1.9	4.4	11.5	3.7	3.9
**5**	**cis-3-hexen-1-ol**	15.21 ± 0.00	NA	NA	NA	NA	NA	NA	NA	NA	NA	NA	NA	NA
**6**	**α-terpinene**	16.26 ± 0.00	0.0031–0.0618	0.54/1.63	1057	1.8	2.8	2.8	−2.7	2.6	4.0	3.9	3.8	8.9
**7**	**limonene**	17.06 ± 0.00	0.0031–0.0619	0.73/2.22	1071	4.3	3.2	7.5	4.0	1.8	3.7	12.8	6.6	6.8
**8**	**p-cymene**	17.16 ± 0.00	0.0028–0.0555	0.73/2.22	1073	6.4	2.4	3.0	1.7	1.5	2.9	5.8	5.0	5.4
**9**	**(+)-rose oxide**	20.28 ± 0.00	0.0100–0.1994	0.75/2.27	1126	0.3	2.7	3.5	1.2	1.8	2.6	11.5	5.1	4.1
**10**	**(−)-rose oxide**	20.43 ± 0.00	0.0100–0.1994	0.79/2.39	1129	0.8	2.8	3.6	1.3	1.0	2.5	10.9	5.2	4.7
**11**	**(+/−)-linalool**	23.18 ± 0.00	0.0033–0.1658	0.63/1.90	1175	−0.3	3.0	3.5	0.4	1.9	2.5	11.4	4.8	3.9
**12**	**(+/−)-linalool**	23.87 ± 0.00	0.0033–0.1658	0.52/1.57	1186	−0.6	3.2	4.0	−0.2	1.5	2.6	11.4	4.6	3.6
**13**	**phenylethanol**	29.82 ± 0.00	0.0024–0.1219	0.44/1.32	1286	−4.4	4.1	5.4	−4.8	2.6	3.7	9.9	6.5	6.7
**14**	**cis/trans-citral**	30.66 ± 0.00	0.0032–0.0644	0.74/2.25	1300	−3.3	4.4	3.6	−4.7	3.8	3.5	8.9	5.3	5.7
**15**	**(+/−)-citronellol**	31.95 ± 0.00	0.0099–0.9939	0.63/1.90	1321	−6.9	6.0	4.6	−7.2	3.9	3.3	7.1	6.2	8.6
**16**	**(+/−)-citronellol**	32.06 ± 0.00	0.0099–0.9939	0.85/2.57	1323	−4.7	5.5	5.7	−5.8	3.8	4.1	9.8	6.7	5.6
**17**	**nerol**	32.35 ± 0.00	0.0051–0.5071	0.42/1.29	1328	2.9	3.1	2.9	2.3	1.8	2.3	9.6	9.6	8.5
**18**	**cis/trans-citral**	33.26 ± 0.00	0.0032–0.0644	0.72/2.18	1344	−1.8	4.9	4.9	−3.5	3.7	3.9	9.4	8.3	7.6
**19**	**geraniol**	34.30 ± 0.00	0.0101–1.0055	0.43/1.29	1361	−3.3	5.8	5.7	−5.7	4.0	4.1	7.2	7.6	6.1
**20**	**(+/−)-citronellyl acetate**	35.16 ± 0.00	0.0081–0.0542	1.18/3.58	1376	−4.6	4.5	5.5	−10.3	5.8	5.8	1.6	5.7	8.6
**21**	**(+/−)-citronellyl acetate**	35.27 ± 0.00	0.0081–0.0542	1.24/3.75	1378	0.3	3.6	5.2	−4.6	6.0	4.5	5.6	3.4	6.0
**22**	**neryl acetate**	35.87 ± 0.00	0.0022–0.1097	0.57/1.73	1388	0.7	4.3	4.4	−0.3	2.1	2.3	9.3	6.3	4.6
**23**	**(cis/trans)-β-damascenone**	36.70 ± 0.00	0.0507–1.0143	1.28/3.89	1402	−1.0	6.5	5.1	3.6	4.8	4.6	9.2	4.1	5.5
**24**	**geranyl acetate**	37.78 ± 0.00	0.0063–0.1266	0.85/2.57	1421	1.5	4.9	5.6	0.6	4.2	6.4	4.4	7.3	9.9
**25**	**β-caryophyllene**	37.87 ± 0.00	0.0033–0.0666	0.82/2.49	1423	−4.9	4.5	5.4	−2.2	2.3	3.0	9.9	7.3	5.7
**26**	**(cis/trans)-β-damascenone**	38.11 ± 0.00	0.0507–1.0143	0.75/2.27	1427	6.8	3.6	3.1	5.7	2.8	5.1	13.3	2.9	4.2
**27**	**β-damascone**	38.64 ± 0.00	0.0013–0.0660	0.77/2.34	1436	−1.1	5.2	4.6	−2.5	2.1	3.5	7.5	7.5	6.5
**28**	**eugenol**	39.61 ± 0.00	0.0025–0.1228	0.64/1.95	1453	−0.2	4.7	4.5	−1.9	2.5	2.9	7.9	5.2	4.1
**29**	**methyleugenol**	41.11 ± 0.00	0.0023–0.1153	0.03/0.10	1479	0.3	4.4	4.1	0.5	2.4	2.9	7.6	4.1	4.6
**30**	**farnesol**	58.67 ± 0.00	0.0061–0.1227	0.79/2.39	1802	2.7	8.5	10.1	−0.3	2.7	4.3	2.8	5.1	6.2

## Data Availability

The data presented in this study are available on request from the corresponding author. Developed Matlab Code is available in the Supplementary Materials.

## Supplementary Materials

The following supporting information can be downloaded at: https://www.mdpi.com/article/10.3390/plants12030506/s1.
