# Supplementary material for: Determination of Major, Minor and Chiral Components as Quality and Authenticity Markers of Rosa damascena Oil by GC-FID
Source: Plants (Basel). 2023 Jan 22;12(3):506. doi: 10.3390/plants12030506 (PMC9921129; doi:10.3390/plants12030506)
Supplement: Supplementary file 1 [file plants-12-00506-s001.zip › plants-2139849-supplementary.pdf]

Determination of Major, Minor and Chiral Components as Quality and Authenticity Markers of Rosa damascena Oil by GC-FID

Justine Raeber, Sina Favrod and Christian Steuer *
Institute of Pharmaceutical Sciences, Swiss Federal Institute of Technology, 8093 Zurich, Switzerland
* Correspondence: christian.steuer@pharma.ethz.ch

Supplementary Materials

Contents

Validation Data 2
Matlab-Code 25
MS-spectra 27

Validation Data

Table S1: Resolution (Rs), recovery excess (RE) in % for authentic rose oil (RO) samples originating from Bulgaria (BG), Morocco (MA) and Turkey (TR) as well as lavender, rosemary, lemon and caraway oil. The RE is presented as the mean value with standard deviation. The number of repeated measurements are given with n. Samples were analysed on a DB-wax column.

Analyte	Rs	RE Authentic RO [%] (n=5)		RE RO_{BG} [%] (n=3)	RE RO_{MA} [%] (n=3)	RE RO_{TR} [%] (n=3)	RE RO_{real} [%] (n=3)	RE Lavender [%] (n=2)	RE Rosemary [%] (n=2)	RE Lemon [%] (n=2)	RE Caraway [%] (n=2)
		QC_{high}	QC_{low}								
**α-pinene**	16.1	104,5 ± 9.8	110,0 ± 3.2	94,6 ± 2.0	99,5 ± 7.3	87,8 ± 2.2	92,5 ± 1.2	99,0 ± 2.7	111,3 ± 4.7	98,6 ± 1.5	101,7 ± 3.1
**Camphene**	12.3	107,1 ± 8.4	107,3 ± 12.9	104,4 ± 4.4	111,2 ± 3.3	101,3 ± 3.5	109,3 ± 4.5	104,3 ± 2.3	133,3 ± 9.4	119,2 ± 4.8	110,9 ± 0.0
**(-)-β-pinene**	22.7	107,2 ± 11.7	119,9 ± 3.9	95,3 ± 3.0	99,1 ± 9.5	86,5 ± 3.5	91,2 ± 3.0	90,6 ± 7.5	142,0 ± 14.6	133,3 ± 11.5	103,0 ± 3.5
**α-terpinene**	6.0	106,5 ± 15.7	88,3 ± 10.3	91,9 ± 1.7	98,3 ± 9.2	86,6 ± 3.1	89,9 ± 2.6	95,7 ± 1.6	105,6 ± 4.5	101,6 ± 1.7	105,0 ± 5.7
**limonene**	28.3	104,7 ± 13.6	84,2 ± 5.9	89,6 ± 1.7	99,5 ± 10.7	82,6 ± 4.0	90,2 ± 1.5	91,6 ±2.3	126,6 ± 13.0	259,3 ± 10.8	568,4 ± 131.2
**p-cymene**	34.1	104,6 ± 12.9	114,0 ± 6.1	94,3 ± 2.8	98,1 ± 9.9	90,2 ± 1.5	88,1 ± 1.6	104,4 ± 4.0	116,2 ±8.1	97,6 ± 0.5	107,9 ± 5.3
**(+/-)-rose oxide**	17.6	103,7 ± 16.7	114,6 ± 5.6	90,7 ± 1.9	85,7 ± 2.7	80,0 ± 1.8	84,2 ± 3.0	96,0 ± 2.4	101,9 ± 4.4	92,7 ± 0.4	103,4 ± 5.5
**cis-3-hexen-1-ol**	85.8	NA	NA	NA	NA	NA	NA	NA	NA	NA	NA
**linalool**	11.3	104,1 ± 16.8	99,4 ± 8.6	85,9 ± 3.0	94,1 ± 6.7	83,2 ± 2.2	84,8 ± 3.4	93,8 ± 6.8	104,5 ± 6.0	93,0 ± 1.2	103,9 ± 5.5
**β-caryophyllene**	16.8	95,5 ± 16.2	97,2 ± 15.9	80,5 ± 1.2	85,3 ± 7.5	100,2 ± 2.0	82,5 ± 6.9	73.3 ± 2.1	123,0 ± 26.2	85,5 ± 1.7	104,2 ± 5.1
**β-damascenone**	6.3	94,6 ± 16.6	103,5 ± 3.7	87,5 ± 7.8	85,1 ± 5.7	87,5 ± 1.7	85,7 ± 4.6	92,8 ± 0.8	105,5 ± 14.2	99,2 ± 8.4	111,9 ± 6.6
**citronellyl acetate**	4.0	96,7 ± 13.6	116,5 ± 14.5	81,9 ± 0.9	92,2 ± 5.6	82,8 ± 1.2	83,6 ± 3.7	95,9 ± 0.3	110,2 ± 10.9	101,8 ± 8.9	111,8 ± 8.5
**citral**	14.2	102,6 ± 16.5	119,2 ± 9.3	86,1 ± 7.4	85,1 ± 8.3	87,7 ± 1.5	83,1 ± 9.0	91,2 ± 2.3	108,6 ± 9.3	112,7 ± 4.7	107,6 ± 7.4
**neryl acetate**	8.5	93,2 ± 14.5	115,5 ± 13.5	85,0 ± 1.8	92,8 ± 8.0	81,4 ± 0.5	84,3 ± 6.7	92,6 ± 0.1	105,2 ± 7.7	105,1 ± 0.6	750,9 ± 161.1
**geranyl acetate**	4.8	106,9 ± 12.1	103,2 ± 11.8	81,7 ± 2.7	81.1 ± 4.5	93,5 ± 1.8	84,8 ± 8.6	115,6 ± 0.8	92,9 ± 3.4	82,4 ± 8.8	90,6 ± 5.0
**citronellol**	10.6	110,2 ± 9.5	82,6 ± 18.8	109,6 ± 3.6	88,8 ± 5.5	87.0 ± 2.6	113,3 ± 10.2	92,9 ± 0.8	98,5 ± 6.4	87,9 ± 0.0	101,9 ± 6.4
**nerol**	2.4	93,9 ± 17.7	104,3 ± 10.0	104,1 ± 3.2	92,7 ± 4.9	92,8 ± 1.6	88,1 ± 6.5	95,3 ± 0.4	96,8 ± 6.3	93,1 ± 6.7	100,2 ± 6.2
**phenylethanol**	13.0	103,6 ± 17.4	119,1 ± 9.3	101,4 ± 4.2	98,6 ± 5.1	85,0 ± 1.0	95,5 ± 4.4	105,5 ± 0.4	109,7 ± 6.1	101,2 ± 2.4	109,0 ± 5.2
**geraniol**	44.0	93,9 ± 16.3	110,7 ± 11.0	103,2 ± 3.2	83,9 ± 4.8	87,3 ± 1.4	82,3 ± 7.1	89,5 ±1.2	94,6 ± 6.7	84,1 ± 0.1	98,5 ± 6.4
**methyleugenol**	38.6	91,8 ± 18.0	90,8 ± 10.1	81,9 ± 1.8	83,0 ± 5.5	82,0 ± 2.9	85,3 ± 11.4	90,5 ± 0.4	97,6 ± 7.3	83,7 ± 1.0	104,5 ± 5.9
**eugenol**	47.6	110,5 ± 11.1	100,8 ± 14.7	104,7 ± 1.3	92,4 ± 3.2	96,7 ± 1.1	99,9 ± 4.5	95,4 ± 0.6	107,9 ± 14.2	93,9 ± 0.7	115,1 ± 8.8
**farnesol**	0	87,5 ± 30.1	96,0 ± 17.8	97,6 ± 4.7	89,5 ± 15.7	116.3 ± 15.7	88,1 ± 9.8	99,1 ± 2.8	110,9 ± 12.2	101,6 ± 7.1	125,3 ± 10.9

Table S2: Resolution (Rs), recovery excess (RE) in % for authentic rose oil (RO) samples originating from Bulgaria (BG), Morocco (MA) and Turkey (TR) as well as lavender, rosemary, lemon and caraway oil. The RE is presented as the mean value with standard deviation. The number of repeated measurements are given with n. Samples were analysed on a chiral column.

Analyte	Rs	RE Authentic RO [%] (n=5)		RE RO_{BG} [%] (n=3)	RE RO_{MA} [%] (n=3)	RE RO_{TR} [%] (n=3)	RE RO_{real} [%] (n=3)	RE Lavender [%] (n=3)	RE Rosemary [%] (n=3)	RE Lemon [%] (n=3)	RE Caraway [%] (n=3)
		QC_{high}	QC_{low}								
**(+)-α-pinene**	2.4	91,2 ± 0.9	88,0 ± 0.8	87,7 ± 0.2	92,2 ± 0.8	89,3 ± 0.5	92,4 ± 4.1	94,4 ± 8.4	96,5 ± 3.3	90,7 ± 0.9	87,8 ± 2.9
**(+/-)-camphene**	5.9	100,2 ± 1.6	118,3 ± 3.2	104,7 ± 1.4	109,2 ± 1.5	104,3 ± 2.2	111,6 ± 1.3	116,0 ± 6.5	116,3 ± 5.8	117,3 ± 4.8	100,4 ± 1.9
**(+/-)-camphene**	12.0	91,7 ± 6.3	107,3 ± 3.5	92,0 ± 4.3	98,0 ± 1.0	91,9 ± 1.5	95,3 ± 4.3	104,6 ± 12.4	98,7 ± 12.4	94,3 ± 4.5	87,1 ± 5.5
**(-)-β-pinene**	28.6	91,1 ± 1.8	105,3 ± 10.5	91,6 ± 1.1	96,6 ± 0.8	93,4 ± 0.4	95,6 ± 4.7	100,3 ± 9.6	105,4 ± 11.4	83,5 ± 18.0	89,0 ± 3.3
**cis-3-hexen-1-ol**	8.3	NA	NA	NA	NA	NA	NA	NA	NA	NA	NA
**α-terpinene**	7.9	91,9 ± 1.5	100,8 ± 2.6	91,9 ± 4.2	92,5 ± 4.6	89,6 ± 1.5	94,8 ± 3.0	93,9 ± 6.2	99,2 ± 12.8	95,6 ± 7.7	90,8 ± 3.4
**limonene**	1.2	99,7 ± 4.8	119,8 ± 2.9	88,5 ± 1.9	101,5 ± 7.5	91,1 ± 2.4	97,2 ± 4.9	98,1 ± 4.6	108,2 ± 24.3	54,6 ± 101.9	229,5 ± 102.2
**p-cymene**	30.6	97,2 ± 3.9	116,1 ± 1.9	89,8 ± 1.3	89,7 ± 3.0	90,6 ± 0.9	88,8 ± 1.8	86,1 ± 18.8	87,7 ± 13.3	36,7 ± 7.4	252,1 ± 420.3
**(+)-rose oxide**	1.5	96,4 ± 2.0	106,5 ± 5.9	88,7 ± 0.7	96,7 ± 1.4	91,5 ± 0.1	96,6 ± 4.7	93,6 ± 6.2	103,0 ± 3.1	92,0 ± 4.0	107,7 ± 6.0
**(-)-rose oxide**	27.1	98,7 ± 1.6	112,6 ± 5.1	91,4 ± 1.2	96,5 ± 2.1	93,6 ± 0.3	97,4 ± 3.7	93,6 ± 1.8	96,9 ± 1.8	94,1 ± 4.3	95,5 ± 3.6
**(+/-)-linalool**	6.7	95,1 ± 1.5	87,5 ± 3.9	86,5 ± 1.5	91,3 ± 1.2	88,4 ± 1.7	93,7 ± 7.5	128,5 ± 88.5	97,4 ± 2.6	94,4 ± 3.1	89,9 ± 3.7
**(+/-)-linalool**	58.4	95,3 ± 1.3	110,4 ± 3.1	86,6 ± 1.4	92,3 ± 0.8	89,1 ± 1.4	94,8 ± 6.7	90,4 ± 3.8	94,7 ± 1.7	89,0 ±1.7	94,7 ± 4.2
**phenylethanol**	6.7	91,7 ± 1.8	92,0 ± 1.5	88,8 ± 0.8	89,9 ± 3.1	87,9 ± 3.1	92,2 ± 4.0	82,8 ± 3.4	90,5 ± 0.6	91,1 ± 2.6	85,5 ± 3.6
**cis/trans-citral**	9.0	107,8 ± 2.3	117,7 ± 7.1	93,0 ± 2.8	98,4 ± 6.1	86,2 ± 5.8	96,3 ± 8.7	81,0 ± 2.2	91,3 ±5.0	89,5 ±7.2	104,0 ± 13.7
**(+/-)-citronellol**	0.9	103,7 ± 3.7	107,4 ± 2.6	106,1 ± 5.3	117,6 ± 10.7	106,5 ± 7.4	95,9 ± 10.6	103,8 ± 3.6	97,6 ± 3.4	100,2 ± 3.2	99,5 ± 5.8
**(+/-)-citronellol**	2.2	103,0 ± 2.7	118,6 ± 13.0	90,5 ± 4.9	104,6 ± 9.4	99,1 ± 9.1	84,8 ± 9.1	101,1 ± 4.3	103,5 ± 2.9	99,5 ± 2.4	95,3 ± 3.2
**nerol**	7.7	94,5 ± 1.5	103,0 ± 5.3	89,9 ± 2.2	94,2 ± 4.4	101,0 ± 2.5	98,7 ± 12.8	92,5 ± 3.2	96,9 ± 2.2	94,3 ± 2.8	111,3 ± 35.4

**cis/trans-citral**	7.3	103,2 ± 3.2	117,0 ± 2.2	99,7 ± 3.7	104,8 ± 7.9	97,7 ± 3.7	104,2 ± 8.2	98,5 ± 8.1	96,8 ± 3.8	95,4 ± 8.5	96,1 ± 8.0
**geraniol**	5.9	94,5 ± 1.5	112,2 ± 4.7	87,5 ± 3.4	93,9 ± 1.6	98,3 ± 1.4	99,1 ± 9.6	92,4 ± 4.1	92,6 ± 2.1	90,2 ± 2.7	88,4 ± 4.3
**(+/-)-citronellyl acetate**	0.8	107,0 ± 5.7	110,8 ± 4.7	89,0 ± 3.7	110,7 ± 11.0	85,4 ± 4.4	88,6 ± 17.8	103,7 ± 8.9	103,2 ± 3.9	98,8 ± 4.6	116,4 ± 19.8
**(+/-)-citronellyl acetate**	4.1	109,0 ± 2.5	117,6 ± 3.0	91,6 ± 2.3	101,9 ± 8.9	94,5 ± 4.2	100,7 ± 8.5	89,6 ± 9.3	107,4 ± 3.5	97,9 ± 13.6	89,8 ± 11.5
**neryl acetate**	6.5	95,3 ± 2.0	108,2 ± 1.5	92,4 ± 3.1	97,4 ± 1.7	89,7 ±2.6	90,8 ± 11.1	92,8 ± 9.6	94,0 ± 1.7	92,9 ± 3.7	88,5 ± 6.5
**(cis/trans)-β-damascenone**	8.4	97,6 ± 2.2	86,3 ± 0.0	99,4 ± 3.2	105,7 ± 11.5	85,0 ± 6.9	94,8 ± 11.7	86,7 ± 6.0	100,4 ± 9.0	89,2 ± 12.2	85,6 ± 0.6
**geranyl acetate**	0.7	93,3 ± 8.7	109,6 ± 4.7	81,7 ± 3.9	80,2 ± 1.4	81,2 ±3.2	87,6 ± 10.6	91,2 ± 4.0	84,8 ± 3.1	103,1 ± 18.4	103,7 ± 2.3
**β-caryophyllene**	1.9	101,0 ± 11.8	110,3 ± 3.3	93,5 ± 0.6	92,4 ± 3.6	88,4 ± 2.7	93,6 ± 3.3	95,0 ± 9.1	109,5 ± 9.9	113,9 ± 15.2	99,1 ± 1.8
**(cis/trans)-β-damascenone**	4.0	96,9 ± 3.0	119,4 ± 3.5	91,7 ± 4.4	92,1 ± 5.2	89,8 ± 5.1	86,7 ± 6.7	90,7 ± 5.8	90,4 ± 2.3	93,2 ± 10.2	88,6 ± 10.2
**β-damascone**	7.6	95,9 ± 2.3	96,9 ± 3.0	93,5 ± 3.3	97,9 ± 1.4	92,6 ± 2.8	93,1 ± 4.7	92,7 ± 4.0	94,1 ± 2.3	94,4 ± 4.2	90,5 ± 3.4
**eugenol**	11.6	97,3 ± 2.3	85,5 ± 13.2	91,5 ± 4.8	95,9 ± 1.7	102,8 ± 2.7	108,9 ± 9.6	91,9 ± 3.6	96,9 ± 2.7	94,5 ± 4.2	91,2 ± 3.3
**methyleugenol**	76.1	95,2 ± 1.8	83,8 ± 12.6	91,0 ± 4.1	98,4 ± 1.1	101,6 ± 3.7	102,7 ± 9.9	94,7 ± 3.3	99,3 ± 2.5	96,1 ± 4.4	93,4 ± 4.0
**farnesol**	0	95,8 ± 1.6	108,1 ± 10.1	97,4 ± 20.8	120,0 ± 4.9	113,9 ± 8.5	118,3 ± 19.8	102,8 ± 17.8	117,6 ± 7.1	114,5 ± 6.2	114,7 ± 4.9

Table S3: Results for robustness testing on the DB-wax column.

	Analyte	α-pinene	Camphene	(-)-β-pinene	α-terpinene	limonene	p-cymene	(+/-)-rose oxide	cis-3-hexen-1-ol	linalool	β-caryophyllene	β-damascenone
Standard method	RT [min]	4.25	5.36	6.84	10.98	12.43	18.28	22.52	24.03	29.00	29.83	31.17
	R_s	16.1	12.3	22.7	6.0	28.3	34.1	17.6	85.8	11.3	16.8	6.3
FID temp. 275 °C	Fold change	1,02	1,03	1,01	1,03	1,01	1,01	1,04	1,00	1,04	1,04	1,18
FID temp. 225 °C	Fold change	0,95	0,96	0,95	0,94	0,96	0,93	0,92	1,00	0,92	0,88	0,87
Inlet temp. 240 °C	Fold change	1,05	1,05	1,06	1,06	1,07	1,05	1,06	1,00	1,07	1,09	1,08
Inlet. temp. 220 °C	Fold change	0,94	0,96	0,93	0,93	0,93	0,92	0,91	1,00	0,92	0,89	0,88

Split ratio 1:100	Fold change	1,07	1,06	1,08	1,07	1,09	1,08	1,11	1,00	1,10	1,13	1,10
Split ratio 1:25	Fold change	1,10	1,11	1,10	1,18	1,14	1,12	1,14	1,00	1,13	1,16	1,15
Flow 2.5 ml/min	RT [min]	3.67	4.65	5.90	9.43	10.67	16.78	21.54	23.20	28.27	28.99	30.45
	R_s	12.2	12.9	27.2	7.2	35.3	35.1	17.5	68.8	8.4	14.4	5.0
Flow 1.5 ml/min	RT [min]	5.16	6.54	8.31	13.31	15.07	19.93	23.74	25.12	29.93	30.86	32.11
	R_s	11.6	13.1	34.7	8.7	27.3	35.7	18.4	73.2	11.8	15.4	6.5
Temperature ramp 7.5 °C/min	RT [min]	4.24	5.37	6.82	10.91	12.35	17.82	21.04	22.12	25.56	26.26	27.09
	R_s	12.3	11.9	22.3	6.7	33.4	35.3	14.8	70.0	14.2	16.9	7.1
Temperature ramp 2.5 °C/min	RT [min]	4.24	5.37	6.82	10.92	12.36	18.87	25.39	28.13	37.31	38.24	41.33
	R_s	13.3	12.2	26.9	7.7	48.0	27.1	15.2	70.3	8.4	23.7	7.4

	Analyte	citronellyl acetate	citral	neryl acetate	geranyl acetate	citronellol	nerol	phenylethanol	geraniol	methyleugenol	eugenol	farnesol
Standard method	RT [min]	31.70	32.00	33.17	33.86	34.17	34.87	35.00	35.89	39.10	41.92	45.38
	R_s	4.0	14.2	8.5	4.8	10.6	2.4	13.0	44.0	38.6	47.6	NA
FID temp. 275 °C	Fold change	1,08	1,10	1,06	1,04	1,05	1,05	1,05	1,05	1,04	1,20	1,06
FID temp. 225 °C	Fold change	0,88	0,89	0,89	0,88	0,90	0,92	0,91	0,91	0,89	0,84	0,85
Inlet temp. 240 °C	Fold change	1,02	1,08	1,07	1,07	1,10	1,09	1,10	1,08	1,04	1,06	1,02
Inlet. temp. 220 °C	Fold change	0,95	0,89	0,87	0,88	0,92	0,94	0,90	0,88	0,85	0,88	0,95

Split ratio 1:100	Fold change	1,07	0,94	1,11	1,07	1,17	1,16	1,17	1,13	1,13	1,19	1,14
Split ratio 1:1:25	Fold change	1,13	1,20	1,14	1,14	1,13	1,17	1,17	1,14	1,16	1,10	1,16
Flow 2.5 ml/min	RT [min]	30.96	31.25	32.44	33.13	33.47	34.16	34.30	35.19	38.37	41.17	44.65
	R_s	2.9	11.7	6.8	4.5	10.9	1.6	10.0	37.0	28.5	43.2	NA
Flow 1.5 ml/min	RT [min]	32.64	33.00	34.11	34.79	35.08	35.80	35.99	36.81	40.04	42.88	46.31
	R_s	4.4	13.6	8.4	4.1	11.8	2.7	10.8	42.3	37.2	45.0	NA
Temperature ramp 7.5 °C/min	RT [min]	27.44	27.72	28.45	28.91	29.10	29.60	29.74	30.77	32.47	34.41	36.68
	R_s	5.7	11.0	7.0	3.4	11.3	2.5	9.6	44.8	39.5	46.2	NA
Temperature ramp 2.5 °C/min	RT [min]	42.29	42.75	45.11	46.47	47.22	48.47	48.67	50.54	56.63	62.00	69.07

	R_s	3.5	16.6	9.6	5.3	8.8	1.4	13.1	64.2	37.7	54.5	NA

Table S4: Results for robustness testing on the chiral column.

	Analyte	α-pinene	camphene	camphene	(-)-β-pinene	cis-3-hexen-1-ol	α-terpinene	limonene	p-cymene	(+)-rose oxide	(-)-rose oxide	linalool	linalool	phenylethanol	cis/trans-citral	citronellol
Standard method	RT [min]	9.58	9.77	10.27	11.49	15.21	16.26	17.06	17.16	20.28	20.43	23.18	23.87	29.82	30.66	31.95
	R_s	2.4	5.9	12.0	28.6	8.3	7.9	1.2	30.6	1.5	27.1	6.7	58.4	6.7	9.0	0.9
FID temp 280 °C	Fold change	1,06	1,10	1,07	1,07	1,00	1,07	1,09	1,05	1,10	1,10	1,14	1,13	1,09	1,13	1,07
FID temp 240 °C	Fold change	1,03	1,02	1,05	1,04	1,00	1,04	1,07	0,98	1,09	1,07	1,08	1,07	1,04	1,06	1,08
Inlet temp. 260 °C	Fold change	1,02	1,07	1,02	1,02	1,00	1,04	1,02	1,07	1,06	1,07	1,05	1,05	1,03	1,05	0,99
Inlet. temp. 220 °C	Fold change	0,94	0,93	0,94	0,92	1,00	0,86	0,91	0,93	0,91	0,89	0,95	0,95	0,92	0,91	0,91
Split flow 200 ml/min	Fold change	1,07	1,08	1,07	1,08	1,00	1,09	1,09	1,10	1,14	1,14	1,14	1,14	1,09	1,17	1,09
Split flow 80 ml/min	Fold change	0,98	1,00	0,98	0,98	1,00	0,95	1,00	0,94	0,98	0,97	1,01	1,01	0,99	1,00	1,01
Constant flow 120 kPa	RT [min]	8.51	8.67	9.17	10.29	13.99	14.90	15.67	15.75	18.74	18.89	21.71	22.41	28.35	28.99	30.35

	R_s	1.4	4.5	10.0	27.1	6.7	5.6	0.6	24.0	1.4	22.6	5.6	43.8	41.6	11.8	0.9
Flow const. Pressure 80 kPa	RT [min]	11.05	11.30	11.83	13.13	16.82	18.09	18.89	19.02	22.28	22.43	25.07	25.74	31.67	32.79	33.98
	R_s	2.3	4.8	11.8	30.03	8.6	6.5	1.3	26.7	1.2	19.5	4.9	43.7	8.3	8.8	0.7
Temperature ramp 4 °C/min	RT [min]	8.09	8.28	8.59	9.38	11.20	12.12	12.57	12.66	14.49	14.57	15.78	16.09	19.04	19.19	20.40
	R_s	2.6	4.2	10.7	19.2	9.7	5.5	1.1	27.7	1.2	17.8	4.6	45.8	1.6	10.6	0.7
Temperature ramp 1 °C/min	RT [min]	10.90	11.06	11.81	13.51	20.12	21.17	22.52	22.52	27.66	27.92	33.71	35.16	46.87	49.46	50.26
	R_s	1.3	6.1	11.9	31.5	5.0	7.1	24.1	24.1	1.4	30.5	6.4	51.6	12.7	4.4	1.2
	Analyte	citronellol	nerol	cis/trans-citral	geraniol	citronellyl acetate	citronellyl acetate	neryl acetate	(cis/trans)-β-damascenone	geranyl acetate	β-caryophyllene	(cis/trans)-β-damascenone	β-damascone	eugenol	methyleugenol	farnesol
Standard method	RT [min]	32.06	32.35	33.26	34.30	35.16	35.27	35.87	36.70	37.78	37.87	38.11	38.64	39.61	41.11	58.67
	R_s	2.2	7.7	7.3	5.9	0.8	4.1	6.5	8.4	0.7	1.9	4.0	7.6	11.6	76.1	-
FID temp 280 °C	RT [min]	32.06	32.35	33.26	34.30	35.16	35.27	35.87	36.70	37.78	37.87	38.11	38.64	39.61	41.11	58.67
	Fold change	1,02	1,11	1,11	1,13	1,16	1,12	1,13	1,13	1,05	1,03	1,16	1,14	1,14	1,12	1,15
FID temp. 240 °C	RT [min]	32.06	32.35	33.26	34.30	35.16	35.27	35.87	36.70	37.78	37.87	38.11	38.64	39.61	41.11	58.67

	Fold change	1,04	1,06	1,06	1,07	1,14	1,09	1,07	1,09	1,01	0,96	1,10	1,09	1,15	1,12	1,08
Inlet temp. 260 °C	RT [min]	32.06	32.35	33.26	34.30	35.16	35.27	35.87	36.70	37.78	37.87	38.11	38.64	39.61	41.11	58.67
	Fold change	0,97	1,05	1,04	1,05	1,19	1,20	1,05	1,09	0,98	1,01	1,06	1,03	1,08	1,08	1,04
Inlet. Temp. 220 °C	RT [min]	32.06	32.35	33.26	34.30	35.16	35.27	35.87	36.70	37.78	37.87	38.11	38.64	39.61	41.11	58.67
	Fold change	0,89	0,91	0,90	0,90	0,94	0,91	0,89	0,89	0,89	0,87	0,91	0,88	0,89	0,90	0,84
Split flow 200 ml/min	Fold change	1,05	1,13	1,13	1,16	1,29	1,28	1,13	1,14	1,17	1,18	1,17	1,16	1,19	1,17	1,31
Split flow 80 ml/min	Fold change	1,00	0,92	0,98	1,01	1,06	1,03	0,97	0,96	1,04	1,03	1,00	0,98	1,03	1,00	0,99
Constant flow 120 kPa	RT [min]	30.47	30.75	31.55	32.69	33.38	33.51	34.06	34.76	35.97	35.97	36.35	36.70	37.79	39.28	56.67
	R_s	1.9	6.2	7.7	4.8	1.0	4.3	5.4	7.3	2.4	NA	2.3	7.5	11.2	123.6	NA
Flow const. Pressure	RT [min]	34.07	34.36	35.41	36.34	37.40	37.50	38.15	39.16	40.05	40.34	40.34	41.10	41.93	43.45	61.16

	R_s	2.1	7.4	6.5	7.4	0.7	4.6	7.1	6.3	1.9	5.0	NA	5.4	10.0	130.6	NA
Temperature ramp 4 °C/min	RT [min]	20.45	20.6	21.27	21.61	22.38	22.38	22.82	23.53	23.76	23.85	24.14	24.50	24.75	25.54	34.12
	R_s	2.0	8.2	4.2	7.6	3.6	3.6	6.3	2.5	1.1	3.6	4.4	2.7	9.3	68.9	NA
Temperature ramp 1 °C/min	RT [min]	50.52	51.08	52.03	54.83	55.23	55.54	56.36	57.08	59.49	60.26	60.92	61.17	63.68	66.56	100.56
	R_s	2.3	3.9	11.5	1.6	1.3	3.4	3.1	10.3	3.3	2.8	1.1	9.0	11.4	120.1	NA

Table S5: concentration determination of 10 authentic rose oils after analysis on a DB-wax column. Bold numbers are concentrations slightly out of the calibration range.

**Species**	***R. damascena***	***P. graveolens***	***P. graveolens***	***R. damascena***	***R. damascena***	***R. damascena***	***R. damascena***	***R. damascena***	***R. damascena***	***R. damascena***
**Quality**	pure	pure	pure	10%	pure	pure	pure	pure	pure	10%
**Origin**	Bulgaria	NA	NA	Morocco	Turkey	Bulgaria	Bulgaria	Turkey	Turkey	Bulgaria
**analyte**	conc [mg/ml]	conc [mg/ml]	conc [mg/ml]	conc [mg/ml]	conc [mg/ml]	conc [mg/ml]	conc [mg/ml]	conc [mg/ml]	conc [mg/ml]	conc [mg/ml]
**α-pinene**	1.62 ± 0.00	1.99 ± 0.02	3.24 ± 0.02	0.51 ± 0.01	5.11 ± 0.01	6.24 ± 0.04	2.94 ± 0.01	3.60 ± 0.02	14.24 ± 0.05	0.58 ± 0.03
**camphene**	0.03 ± 0.00	0.04 ± 0.00	0.05 ± 0.00	n.d.	0.04 ± 0.00	0.05 ± 0.00	0.04 ± 0.00	0.04 ± 0.00	0.05 ± 0.00	0.03 ± 0.00
**β-pinene**	0.41 ± 0.00	0.04 ± 0.00	0.06 ± 0.00	0.16 ± 0.00	2.05 ± 0.08	2.19 ± 0.01	0.70 ± 0.00	1.45 ± 0.03	2.78 ± 0.08	0.16 ± 0.01
**α-terpinene**	0.02 ± 0.00	0.04 ± 0.00	0.07 ± 0.00	n.d.	0.05 ± 0.00	0.14 ± 0.00	0.03 ± 0.00	0.12 ± 0.00	0.21 ± 0.02	0.02 ± 0.00
**limonene**	0.12 ± 0.00	1.46 ± 0.05	1.99 ± 0.09	0.25 ± 0.01	0.28 ± 0.00	0.33 ± 0.00	0.23 ± 0.00	0.25 ± 0.00	0.40 ± 0.00	0.23 ± 0.91
**p-cymene**	0.21 ± 0.00	0.42 ± 0.01	**1.06 ± 0.01**	0.09 ± 0.00	0.55 ± 0.01	0.25 ± 0.00	0.49 ± 0.00	0.38 ± 0.00	0.67 ± 0.00	0.03 ± 0.00
**(+/-)-rose oxide**	6.18 ± 0.03	16.12 ± 0.20	18.46 ± 0.14	0.26 ± 0.00	**2.67 ± 0.07**	1.75 ± 0.04	2.83 ± 0.05	4.92 ± 0.00	2.12 ± 0.02	0.20 ± 0.01
**linalool**	14.79 ± 0.09	63.94 ± 0.34	69.88 ± 0.16	1.84 ± 0.04	8.85 ± 0.08	14.27 ± 0.19	17.79 ± 0.09	6.78 ± 0.05	10.51 ± 0.11	1.19 ± 0.04
**β-caryophyllene**	12.42 ± 0.32	11.12 ± 0.10	13.11 ± 0.09	0.63 ± 0.02	8.90 ± 0.31	8.22 ± 0.14	5.74 ± 0.08	10.13 ± 0.30	8.62 ± 0.90	0.32 ± 0.02
**β-damascenone**	1.51 ± 0.22	6.45 ± 0.25	6.86 ± 0.10	0.37 ± 0.05	2.41 ± 0.08	1.81 ± 0.14	1.69 ± 0.08	2.12 ± 0.16	2.08 ± 0.01	0.31 ± 0.01
**citronellyl acetate**	9.89 ± 0.17	8.27 ± 0.07	9.34 ± 0.31	0.53 ± 0.01	9.90 ± 0.17	4.42 ± 0.07	5.21 ± 0.19	9.57 ± 0.11	9.88 ± 0.32	0.25 ± 0.01
**citral**	11.81 ± 0.23	9.19 ± 0.06	10.66 ± 0.06	0.76 ± 0.08	5.68 ± 0.12	12.89 ± 0.22	13.07 ± 0.02	6.93 ± 0.14	7.92 ± 0.30	0.98 ± 0.04
**neryl acetate**	15.27 ± 0.24	12.74 ± 0.13	13.36 ± 0.08	0.49 ± 0.01	7.47 ± 0.12	9.52 ± 0.16	10.11 ± 0.07	6.53 ± 0.08	7.73 ± 0.24	0.65 ± 0.02
**geranyl acetate**	28.54 ± 0.65	7.23 ± 0.06	7.90 ± 0.06	1.04 ± 0.03	15.74 ± 0.27	15.79 ± 0.26	11.82 ± 0.09	20.84 ± 0.15	27.20 ± 0.16	0.87 ± 0.04
**citronellol**	503.42 ± 8.22	479.94 ± 3.50	496.13 ± 2.97	55.38 ± 1.40	433.24 ± 5.56	300.10 ± 4.18	297.12 ± 2.21	419.55 ± 4.16	378.10 ± 2.23	33.81 ± 1.49
**nerol**	204.05 ± 3.53	4.39 ± 0.18	4.11 ± 0.10	14.28 ± 0.20	101.25 ± 1.29	124.17 ± 1.69	107.74 ± 1.00	86.26 ± 0.84	111.13 ± 0.64	10.65 ± 0.23
**phenylethanol**	0.97 ± 0.02	0.19 ± 0.01	0.18 ± 0.01	0.22 ± 0.01	**2.12 ± 0.06**	0.93 ± 0.05	1.11 ± 0.31	4.11 ± 0.05	4.40 ± 0.04	0.12 ± 0.00
**geraniol**	499.85 ± 9.12	221.04 ± 1.78	209.02 ± 1.00	29.52 ± 0.55	220.03 ± 2.75	300.28 ± 4.29	210.33 ± 1.57	199.17 ± 2.21	270.95 ± 2.09	23.73 ± 0.50
**methyleugenol**	28.25 ± 0.69	8.14 ± 0.75	6.27 ± 0.06	4.16 ± 0.07	32.84 ± 0.38	8.97 ± 0.17	29.63 ± 0.29	34.61 ± 0.61	35.98 ± 0.55	0.50 ± 0.02
**eugenol**	21.93 ± 0.72	3.39 ± 0.07	3.02 ± 0.04	3.19 ± 0.06	15.32 ± 0.45	12.25 ± 0.34	8.00 ± 0.11	13.31 ± 0.12	22.19 ± 1.47	0.73 ± 0.01
**farnesol**	**81.18 ± 5.82**	2.95 ± 0.27	0.49 ± 0.07	3.73 ± 0.19	23.16 ± 0.57	30.92 ± 0.57	**40.26 ± 0.47**	23.53 ± 0.52	29.20 ± 1.25	3.21 ± 0.15

Table S6: Concentration determination of 10 authentic rose oils after chiral analysis. Bold numbers are concentrations slightly out of the calibration range. EE and DE for stereoisomers is given.

**Species**	***R. damascena***	***P. graveolens***	***P. graveolens***	***R. damascena***	***R. damascena***	***R. damascena***	***R. damascena***	***R. damascena***	***R. damascena***	***R. damascena***
**Quality**	pure	pure	pure	10%	pure	pure	pure	pure	pure	10%
**Origin**	Bulgaria	NA	NA	Morocco	Turkey	Bulgaria	Bulgaria	Turkey	Turkey	Bulgaria
**analyte**	conc [mg/ml]	conc [mg/ml]	conc [mg/ml]	conc [mg/ml]	conc [mg/ml]	conc [mg/ml]	conc [mg/ml]	conc [mg/ml]	conc [mg/ml]	conc [mg/ml]
**α-pinene**	0.21 ± 0.00	0.21 ± 0.00	0.23 ± 0.00	0.15 ± 0.00	4.84 ± 0.03	7.15 ± 0.01	0.45 ± 0.00	0.34 ± 0.00	16.60 ± 0.08	0.75 ± 0.04
**β-pinene**	0.36 ± 0.00	0.04 ± 0.00	0.05 ± 0.00	0.16 ± 0.01	2.22 ± 0.06	2.28 ± 0.09	0.64 ± 0.00	1.73 ± 0.10	2.76 ± 0.02	0.18 ± 0.01
**α-terpinene**	0.08 ± 0.00	0.25 ± 0.00	0.37 ± 0.00	0.03 ± 0.00	0.15 ± 0.00	0.31 ± 0.00	0.13 ± 0.00	0.23 ± 0.01	0.35 ± 0.00	0.04 ± 0.00
**limonene**	0.07 ± 0.00	0.43 ± 0.00	0.63 ± 0.00	0.20 ± 0.02	0.09 ± 0.00	0.33 ± 0.00	0.12 ± 0.00	0.22 ± 0.05	0.20 ± 0.00	0.29 ± 0.02
**p-cymene**	0.15 ± 0.00	0.23 ± 0.00	0.29 ± 0.00	0.05 ± 0.00	0.30 ± 0.00	0.10 ± 0.00	0.15 ± 0.00	0.17 ± 0.04	0.32 ± 0.00	0.04 ± 0.01
**phenylethanol**	n.d.	n.d.	n.d.	n.d.	n.d.	n.d.	n.d.	n.d.	0.11 ± 0.00	n.d.
**nerol**	174.27 ± 5.82	16.78 ± 0.82	14.56 ± 0.51	17.44 ± 0.76	99.35 ± 0.09	103.05 ± 1.29	140.88 ± 1.57	82.69 ± 1.43	104.22 ± 1.27	13.51 ± 0.16
**neryl acetate**	0.96 ± 0.02	0.09 ± 0.01	0.07 ± 0.01	0.18 ± 0.01	2.93 ± 0.06	0.93 ± 0.00	0.71 ± 0.01	1.09 ± 0.01	1.19 ± 0.01	0.15 ± 0.01
**geraniol**	429.48 ± 2.45	206.47 ± 1.25	197.30 ± 0.74	34.13 ± 0.76	222.15 ± 0.90	253.16 ± 4.94	286 ± 3.39	189.39 ± 3.46	253.27 ± 3.47	26.75 ± 0.30
**geranyl acetate**	21.61 ± 0.20	6.91 ± 0.37	4.53 ± 0.26	1.19 ± 0.06	15.95 ± 0.19	13.06 ± 0.24	16.29 ± 0.24	18.48 ± 0.13	23.26 ± 0.12	1.04 ± 0.07
**β-caryophyllene**	6.97 ± 0.21	9.08 ± 0.20	12.19 ± 0.92	1.02 ± 0.04	3.74 ± 0.15	4.28 ± 0.03	4.58 ± 0.07	5.29 ± 0.16	8.38 ± 0.24	0.32 ± 0.01
**β-damascone**	n.d.	2.65 ± 0.20	3.41 ± 0.10	n.d.	n.d.	0.09 ± 0.01	0.13 ± 0.00	n.d.	n.d.	n.d.
**eugenol**	42.71 ± 0.64	15.82 ± 0.15	17.04 ± 0.44	**2.08 ± 0.05**	37.24 ± 0.86	23.53 ± 1.00	28.97 ± 0.51	37.20 ± 0.43	52.64 ± 0.53	**1.60 ± 0.07**
**methyleugenol**	22.61 ± 0.21	1.01 ± 0.02	0.70 ± 0.01	4.02 ± 0.21	30.47 ± 0.38	7.97 ± 0.12	38.90 ± 0.51	31.32 ± 0.15	31.56 ± 0.11	0.58 ± 0.02
**farnesol**	67.50 ± 1.30	n.d.	n.d.	3.74 ± 0.69	25.47 ± 0.33	25.40 ± 0.49	55.54 ± 1.81	25.14 ± 0.55	29.74 ± 0.91	2.80 ± 0.06
**Enantiomeric excess (EE)/ diastereomeric excess (DE)**										
**(+/-)-camphene**	n.d.	1.00	1.00	n.d.	1.00	1.00	1.00	1.00	n.d.	n.d.
**(+)-rose oxide**	1.00	0.16	0.15	n.d.	n.d.	n.a.	n.d.	n.d.	n.d.	n.a.
**(-)-rose oxide**	n.d.	n.a.	n.a.	1.00	1.00	0.80	1.00	1.00	1.00	0.78
**(+/-)-linalool**	0.06	0.00	0.11	0.16	0.16	0.19	0.05	0.12	0.09	0.19
**(+/-)-citronellol**	1.00	0.24	0.23	1.00	1.00	1.00	0.99	1.00	1.00	1.00
**(trans/cis)-citral**	0.44	0.27	0.17	0.09	0.52	0.21	0.33	0.29	0.28	0.52
**(+/-)-citronellyl acetate**	1.00	0.27	0.22	1.00	1.00	1.00	0.86	0.68	0.69	1.00
**(trans/cis)-β-damascenone**	1.00	0.19	0.10	1.00	n.d.	0.57	0.85	n.d.	n.d.	n.d.

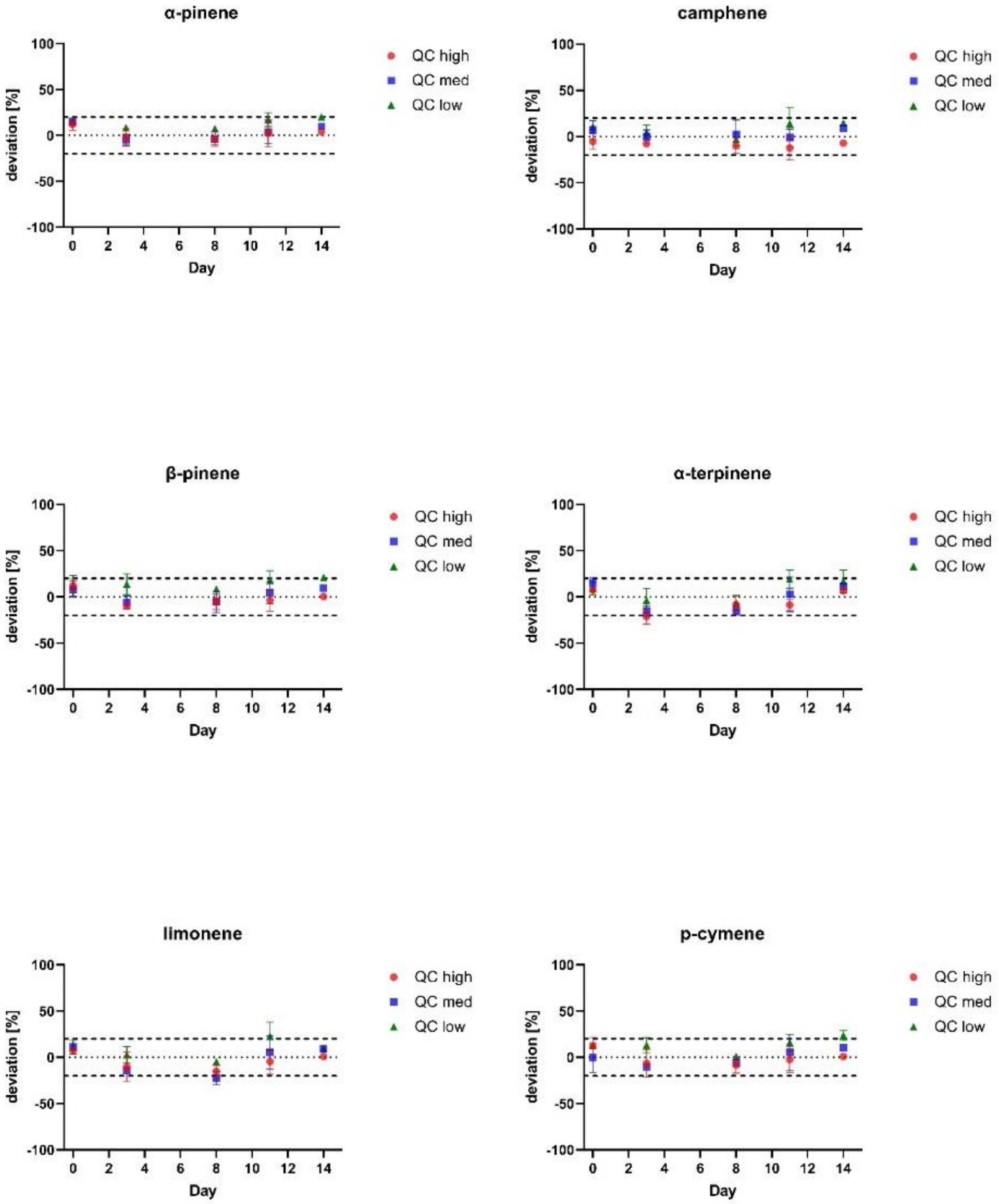
α-pinene
camphene
β-pinene
α-terpinene
limonene
p-cymene
deviation [%]
Day
QC high
QC med
QC low
100
50
0
-50
-100
0
2
4
6
8
10
12
14

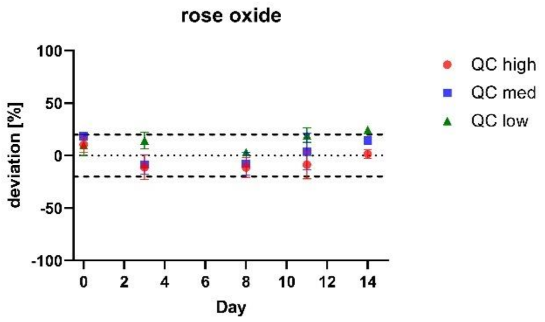
rose oxide
deviation [%]
100
50
0
-50
-100
0 2 4 6 8 10 12 14
Day
QC high
QC med
QC low

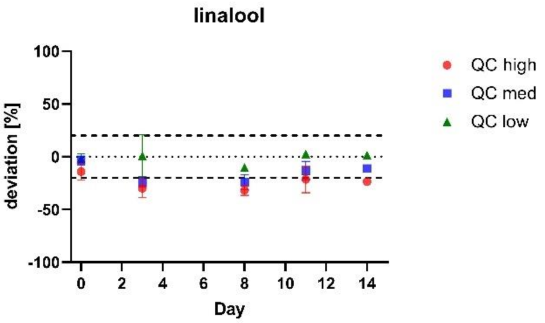
linalool
deviation [%]
100
50
0
-50
-100
0 2 4 6 8 10 12 14
Day
QC high
QC med
QC low

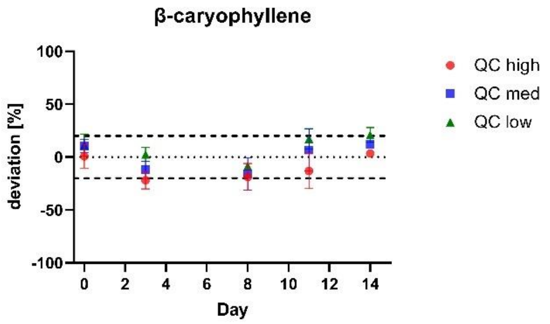
β-caryophyllene
deviation [%]
100
50
0
-50
-100
0 2 4 6 8 10 12 14
Day
QC high
QC med
QC low

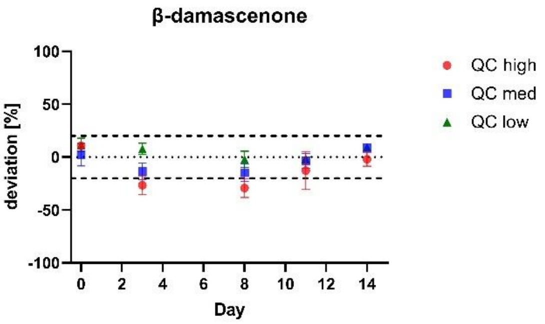
β-damascenone
deviation [%]
100
50
0
-50
-100
0 2 4 6 8 10 12 14
Day
QC high
QC med
QC low

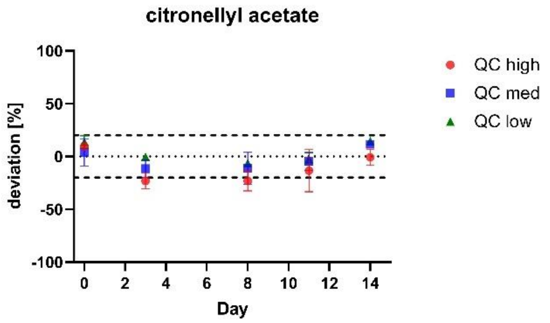
citronellyl acetate
deviation [%]
100
50
0
-50
-100
0 2 4 6 8 10 12 14
Day
QC high
QC med
QC low

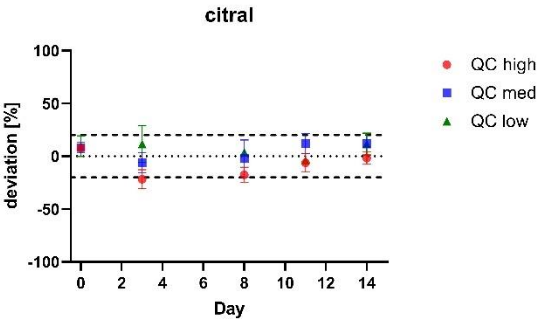
citral
deviation [%]
100
50
0
-50
-100
0 2 4 6 8 10 12 14
Day
QC high
QC med
QC low

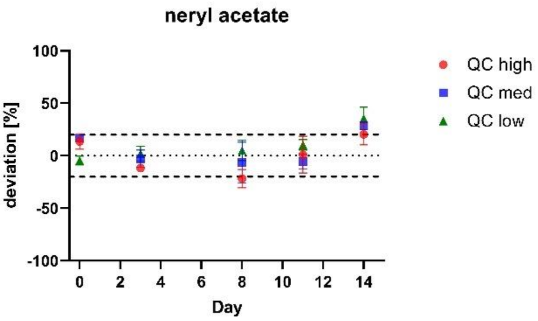
neryl acetate
deviation [%]
Day
QC high
QC med
QC low

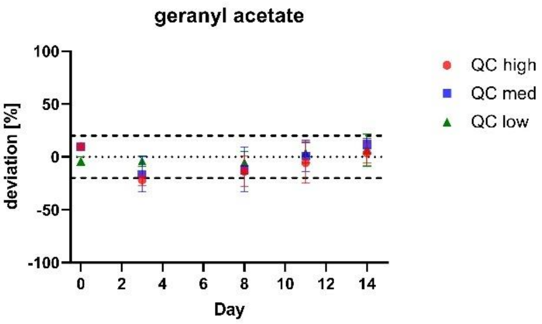
geranyl acetate
deviation [%]
Day
QC high
QC med
QC low

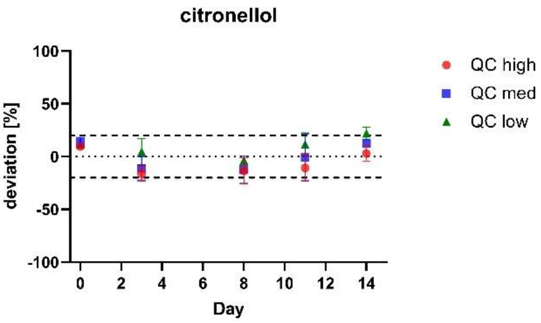
citronellol
deviation [%]
Day
QC high
QC med
QC low

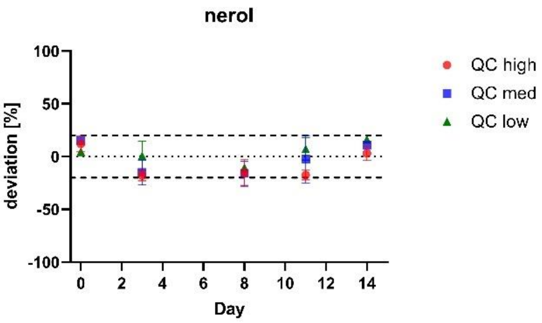
nerol
deviation [%]
Day
QC high
QC med
QC low

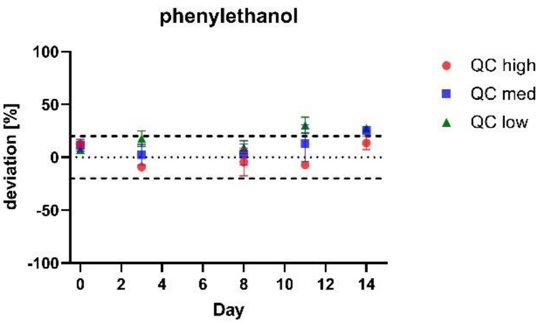
phenylethanol
deviation [%]
Day
QC high
QC med
QC low

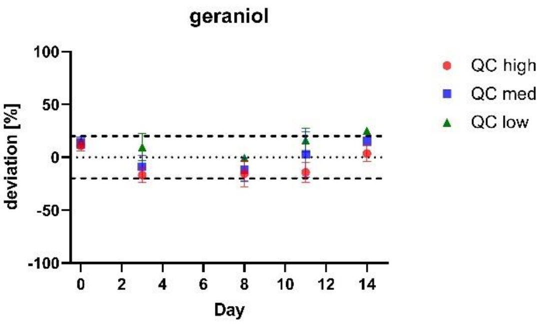
geraniol
deviation [%]
Day
QC high
QC med
QC low

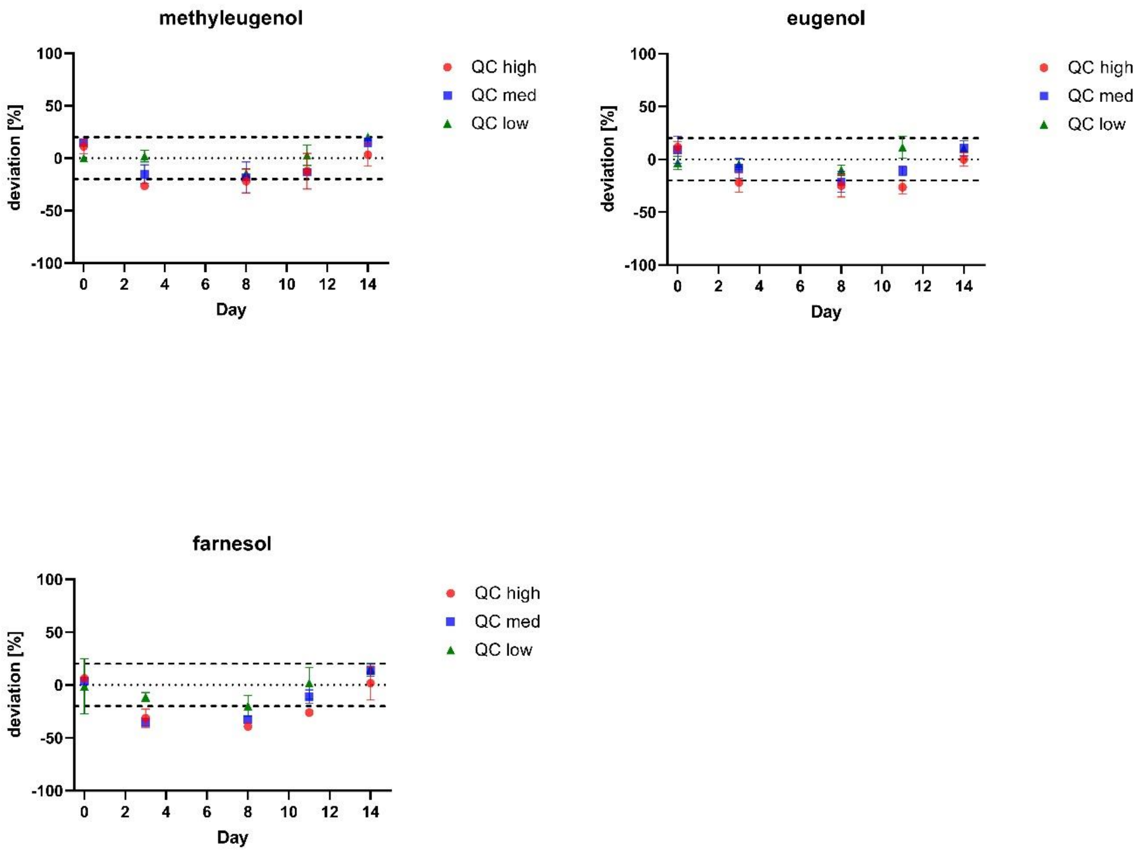

Figure S1: QC stability for samples analysed on the DB Wax column. Thick stippled lines indicate a 20% deviation from measured to calculated value. Validation took place on 5 days during a course of 2 weeks.

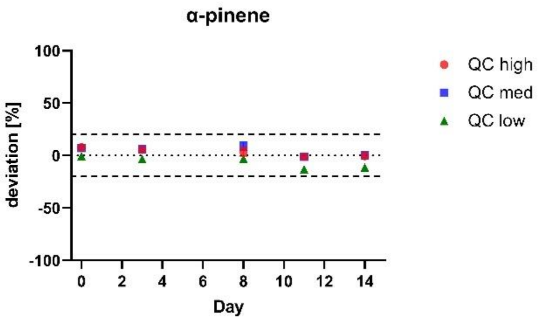
α-pinene
deviation [%]
Day
QC high
QC med
QC low

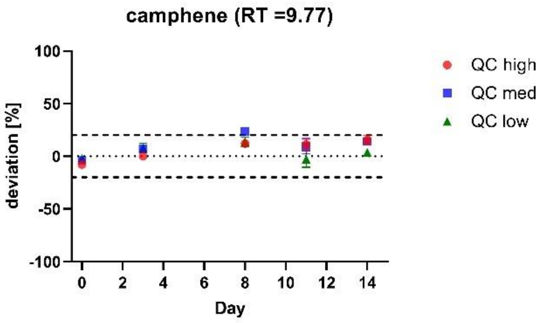
camphene (RT =9.77)
deviation [%]
Day
QC high
QC med
QC low

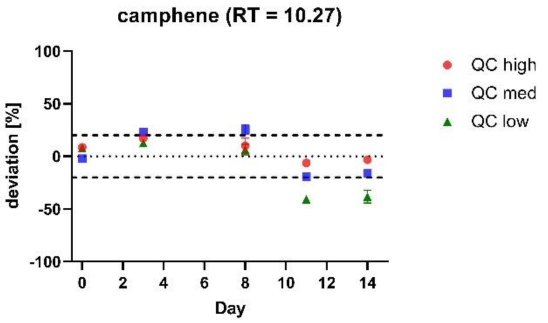
camphene (RT = 10.27)
deviation [%]
Day
QC high
QC med
QC low

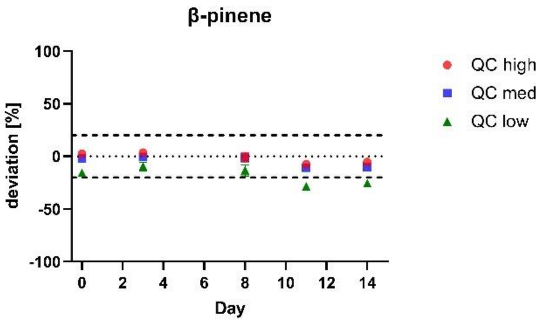
β-pinene
deviation [%]
Day
QC high
QC med
QC low

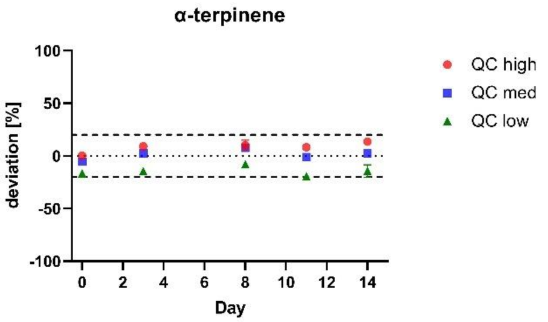
α-terpinene
deviation [%]
Day
QC high
QC med
QC low

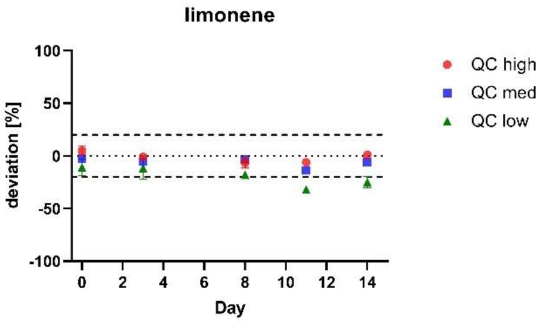
limonene
deviation [%]
Day
QC high
QC med
QC low

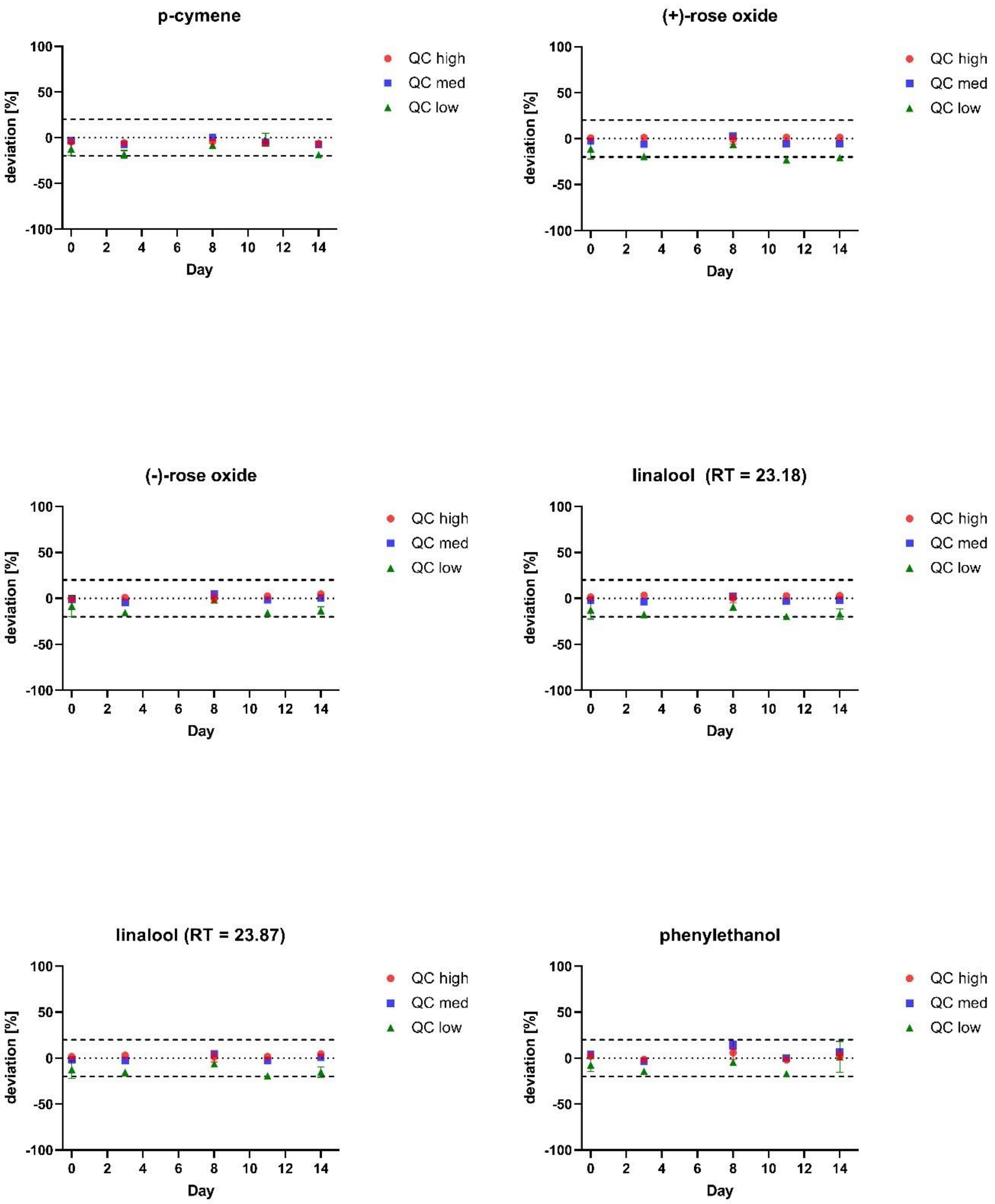
p-cymene
(+)-rose oxide
(-)-rose oxide
linalool (RT = 23.18)
linalool (RT = 23.87)
phenylethanol
deviation [%]
100
50
0
-50
-100
Day
0
2
4
6
8
10
12
14
QC high
QC med
QC low

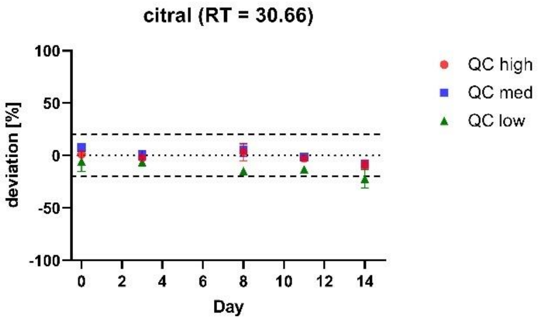
citral (RT = 30.66)
deviation [%]
100
50
0
-50
-100
0 2 4 6 8 10 12 14
Day
QC high
QC med
QC low

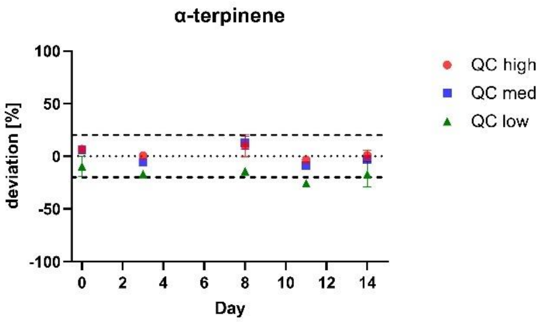
α-terpinene
deviation [%]
100
50
0
-50
-100
0 2 4 6 8 10 12 14
Day
QC high
QC med
QC low

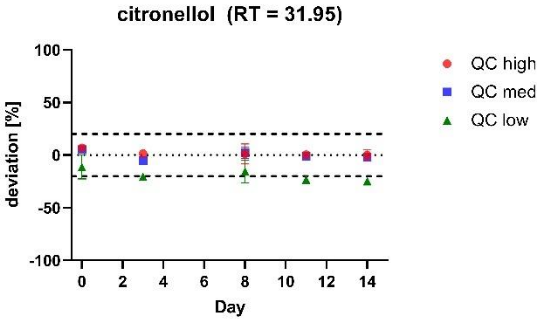
citronellol (RT = 31.95)
deviation [%]
100
50
0
-50
-100
0 2 4 6 8 10 12 14
Day
QC high
QC med
QC low

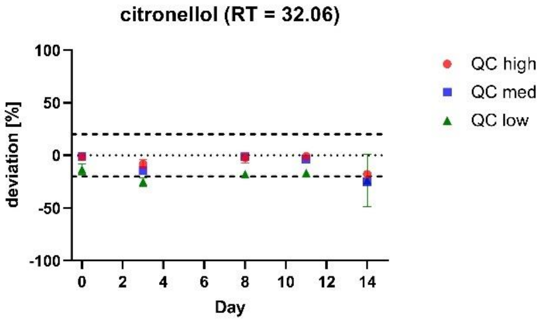
citronellol (RT = 32.06)
deviation [%]
100
50
0
-50
-100
0 2 4 6 8 10 12 14
Day
QC high
QC med
QC low

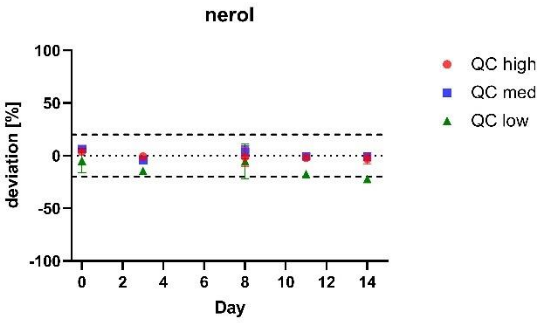
nerol
deviation [%]
100
50
0
-50
-100
0 2 4 6 8 10 12 14
Day
QC high
QC med
QC low

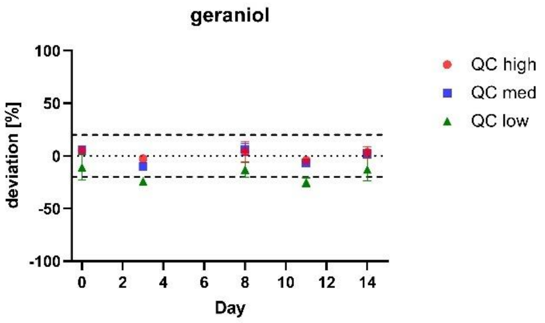
geraniol
deviation [%]
100
50
0
-50
-100
0 2 4 6 8 10 12 14
Day
QC high
QC med
QC low

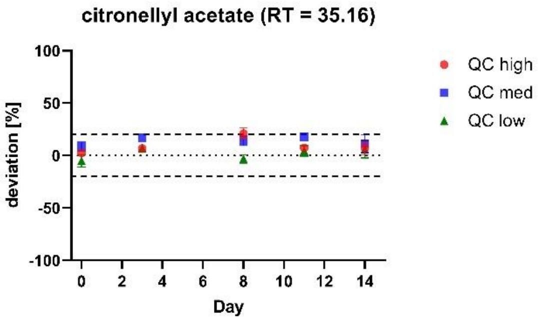
citronellyl acetate (RT = 35.16)
deviation [%]
Day
QC high
QC med
QC low

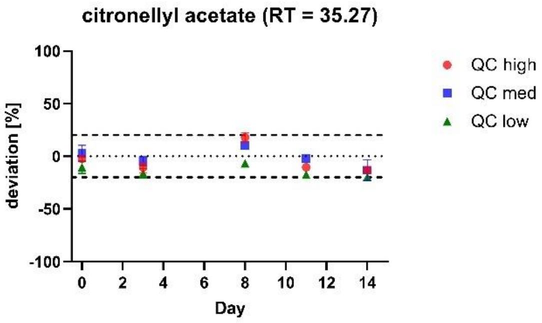
citronellyl acetate (RT = 35.27)
deviation [%]
Day
QC high
QC med
QC low

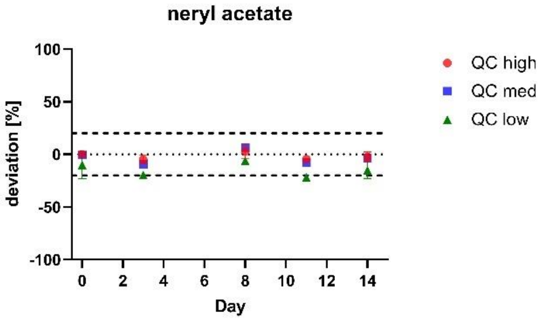
neryl acetate
deviation [%]
Day
QC high
QC med
QC low

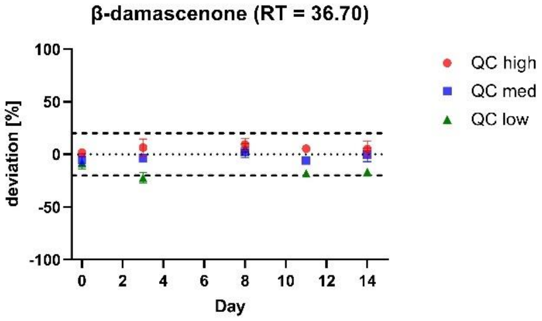
β-damascenone (RT = 36.70)
deviation [%]
Day
QC high
QC med
QC low

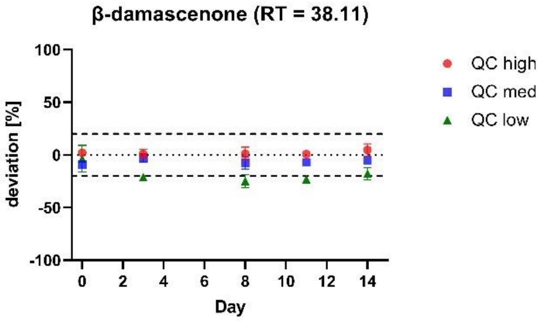
β-damascenone (RT = 38.11)
deviation [%]
Day
QC high
QC med
QC low

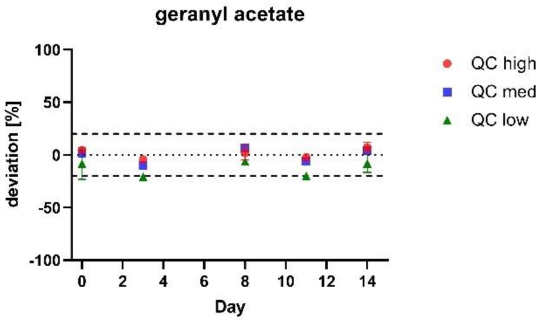
geranyl acetate
deviation [%]
Day
QC high
QC med
QC low

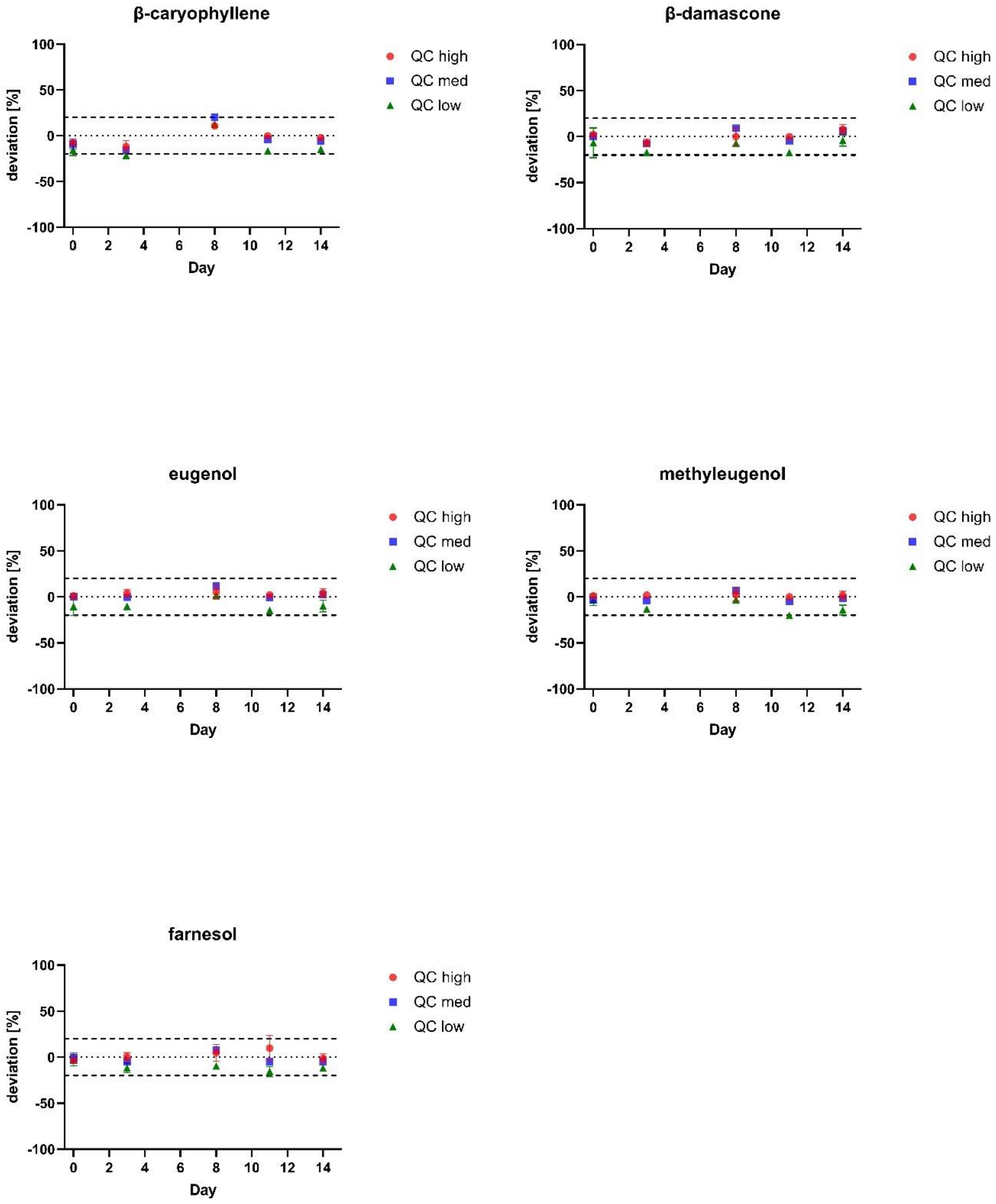

Figure S2: QC stability for samples analysed on the chiral column. Thick stippled lines indicate a 20% deviation from measured to calculated value. Validation took place on 5 days during a course of 2 weeks.

Matlab-Code

```
%% Script used for the chromatographic peak integration from data collected using
ChromCard. Data can be downloaded as a txt-file (export data as ASCII).
%% Retention times for peaks have to be selected beforehand and imported as a txt-
file to the script.



peak_width          = 0.1;  %% Select desired peak width. As a default 0.1 can be
selected
peak_width_half     = peak_width/2;
retention_times     = readmatrix("Retention_times.txt"); %%import retention times
as a column in form of a txt file
peak_bounds_start   = abs(retention_times - peak_width_half);
peak_bounds_end     = abs(retention_times + peak_width_half);
peak_bounds         = zeros(1, length(retention_times)*2);
npeaks              = length(peak_bounds)/2;

for i=1:npeaks

    peak_bounds(2*i-1) = peak_bounds_start(i);
    peak_bounds(2*i)   = peak_bounds_end(i);

end


idx_peak_bounds = zeros(1, length(peak_bounds)); % zero vector with number of Peak
bounds
peaks           = zeros(1, npeaks); % zero vector with amount of peaks -> used to
store area under the curve

% upload data as .txt file

Chrom_Data          = readmatrix("myData.txt"); %Import ChromCard Data as txt file
time                = Chrom_Data(:,1); % extract time as vector in minutes
uV                  = Chrom_Data(:, end); % extract uV as vector
noiseThresh         = 1340; % read from chromatogram where the noise is about
Ts                  = time(2)-time(1); % sampling interval (in what amount of time
is a data point constructed in [min])

noise               = uV;
noise(noise > noiseThresh) = NaN; % NaN=not a number, defines value not as zero
but not defined yet, defines noise as all values below the noise

% Moving Average for noise filtering
T_window    = 1; % min ... width over which the moving average is taken
nSamples    = T_window / Ts; % amounts of data points that are collected over a
minute
noise_avg   = movmean(noise, nSamples, 'omitnan'); % movmean -> returns mean
values over an array -> sliding window. Noise (is uV below threshold), nSamples is
data points for a certain time frame and omitnan a function which jumps over NaN
values



% Determine Peak Index and Integration

for i=1:npeaks
```

```
    timeDiffStart      = abs(time - peak_bounds(2*i - 1)); % find minimum
difference between peak bounds and time
    timeDiffMinStart   = min(timeDiffStart);
    idxPkStart         = find(timeDiffStart == timeDiffMinStart); % find index of
minimum (equal to index of startpeak)
    timeDiffEnd        = abs(time - peak_bounds(2*i));
    timeDiffMinEnd     = min(timeDiffEnd);
    idxPkEnd           = find(timeDiffEnd == timeDiffMinEnd);
    idx_peak_bounds(2*i-1)  = idxPkStart;
    idx_peak_bounds(2*i)    = idxPkEnd;
    peaks(i)                = trapz(time(idxPkStart:idxPkEnd,1),
uV(idxPkStart:idxPkEnd,1) - noise_avg(idxPkStart))*60; % Integration + Offset
correction, Unit is s*uV

    meep = noise_avg(idxPkStart);
   if (isscalar(meep) && isfinite(meep))
        % Plot area of peaks
        plot(time,uV)
        xlabel('time [min]');
        ylabel('uV');
        legend('Data');
        hold on;
%         area(time(idxPkStart:idxPkEnd,1),uV(idxPkStart:idxPkEnd,1),
noise_avg(idxPkStart))

        area(time(idxPkStart:idxPkEnd,1),uV(idxPkStart:idxPkEnd,1), meep)

        colororder('red')
    end
   end

area = transpose(peaks);
area_RT = [retention_times(:), area(:)];

filename = 'myData.xlsx' %write data output from integration as an excel file
xlswrite(filename, area_RT)

clear all
```

MS-spectra:

Table 1: detailed information about the samples analysed using GC-MS.

Number	3018	3057	3058	3053	3021	3072	3073
species	*R. damascena*	*P. graveolens*	*P. graveolens*	*R. damascena*	*R. damascena*	*R. damascena*	*R. damascen*[illegible]
quality	pure	pure	pure	10%	pure	pure	pure
origin	Bulgaria	NA	NA	Morocco	Turkey	Bulgaria	Bulgaria

Alpha pinene

Reference:

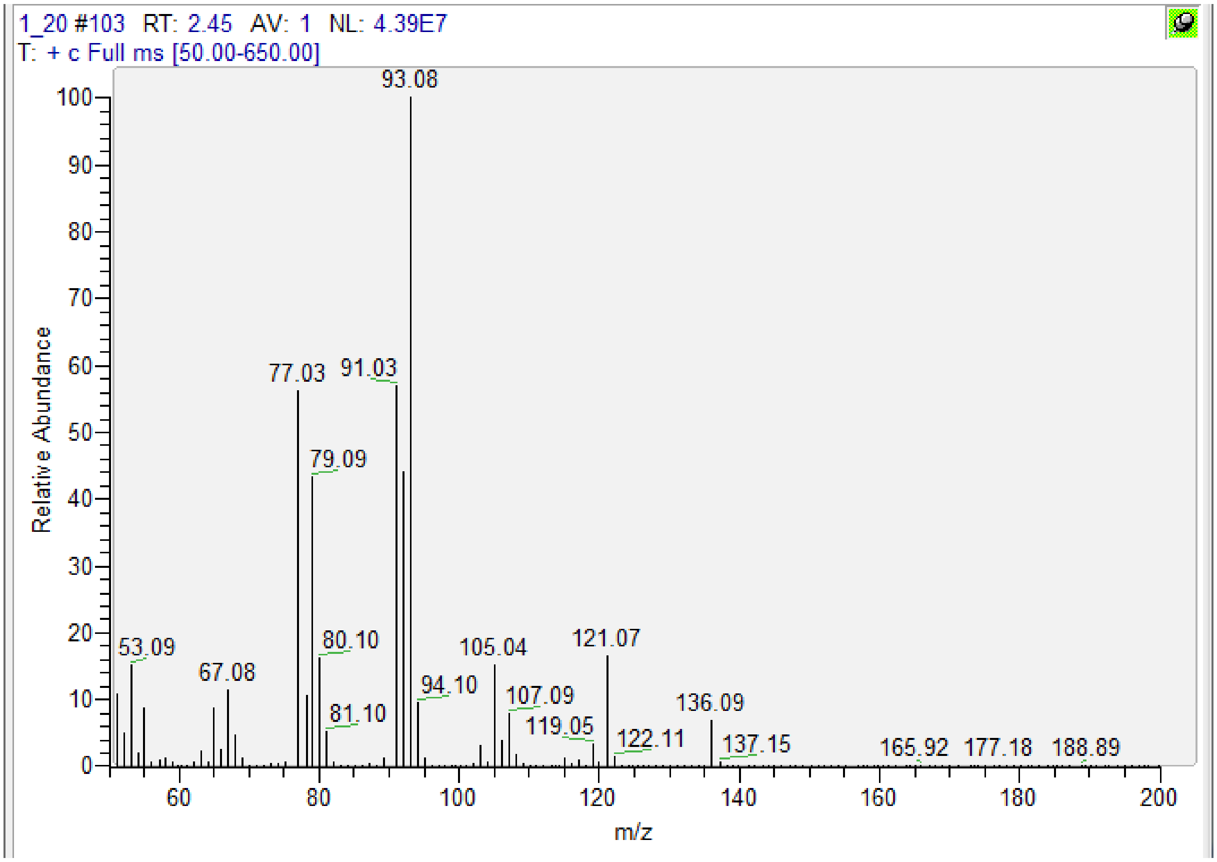

Samples:

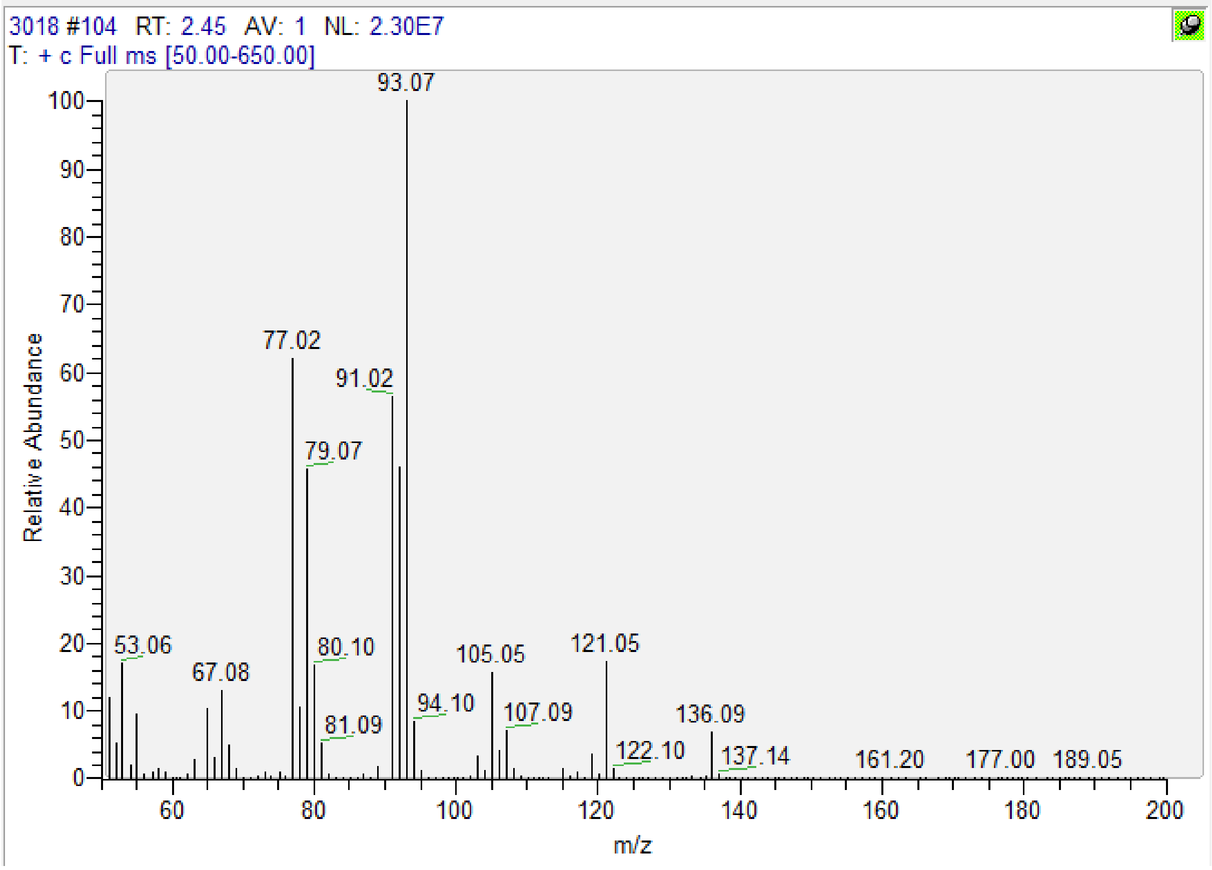

3018 #104 RT: 2.45 AV: 1 NL: 2.30E7
T: + c Full ms [50.00-650.00]
Relative Abundance
m/z
93.07
77.02
91.02
79.07
53.06
67.08
80.10
105.05
121.05
81.09
94.10
107.09
136.09
122.10
137.14
161.20
177.00
189.05

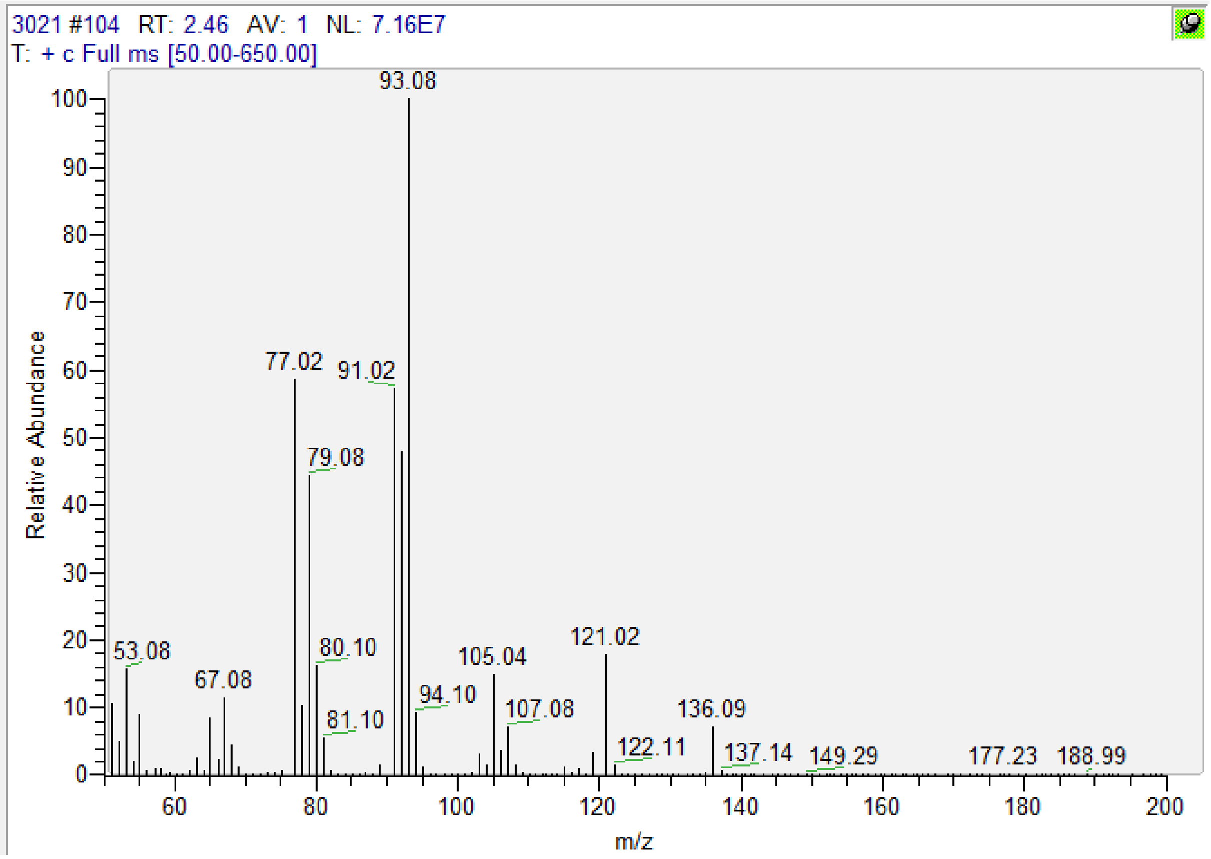
3021 #104 RT: 2.46 AV: 1 NL: 7.16E7
T: + c Full ms [50.00-650.00]
Relative Abundance
m/z
93.08
77.02
91.02
79.08
53.08
67.08
80.10
81.10
94.10
105.04
107.08
121.02
122.11
136.09
137.14
149.29
177.23
188.99

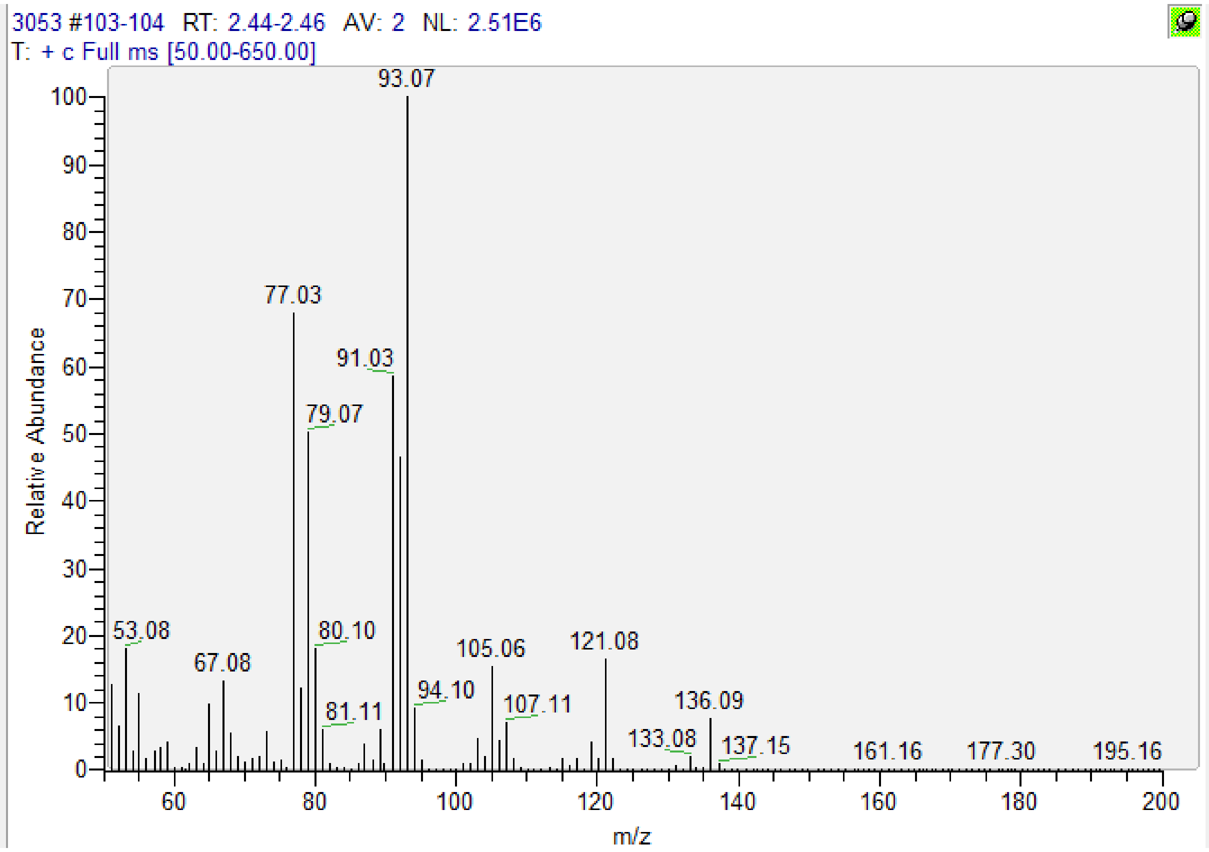
3053 #103-104 RT: 2.44-2.46 AV: 2 NL: 2.51E6
T: + c Full ms [50.00-650.00]
Relative Abundance
m/z
93.07
77.03
91.03
79.07
53.08
67.08
80.10
81.11
94.10
105.06
107.11
121.08
133.08
136.09
137.15
161.16
177.30
195.16

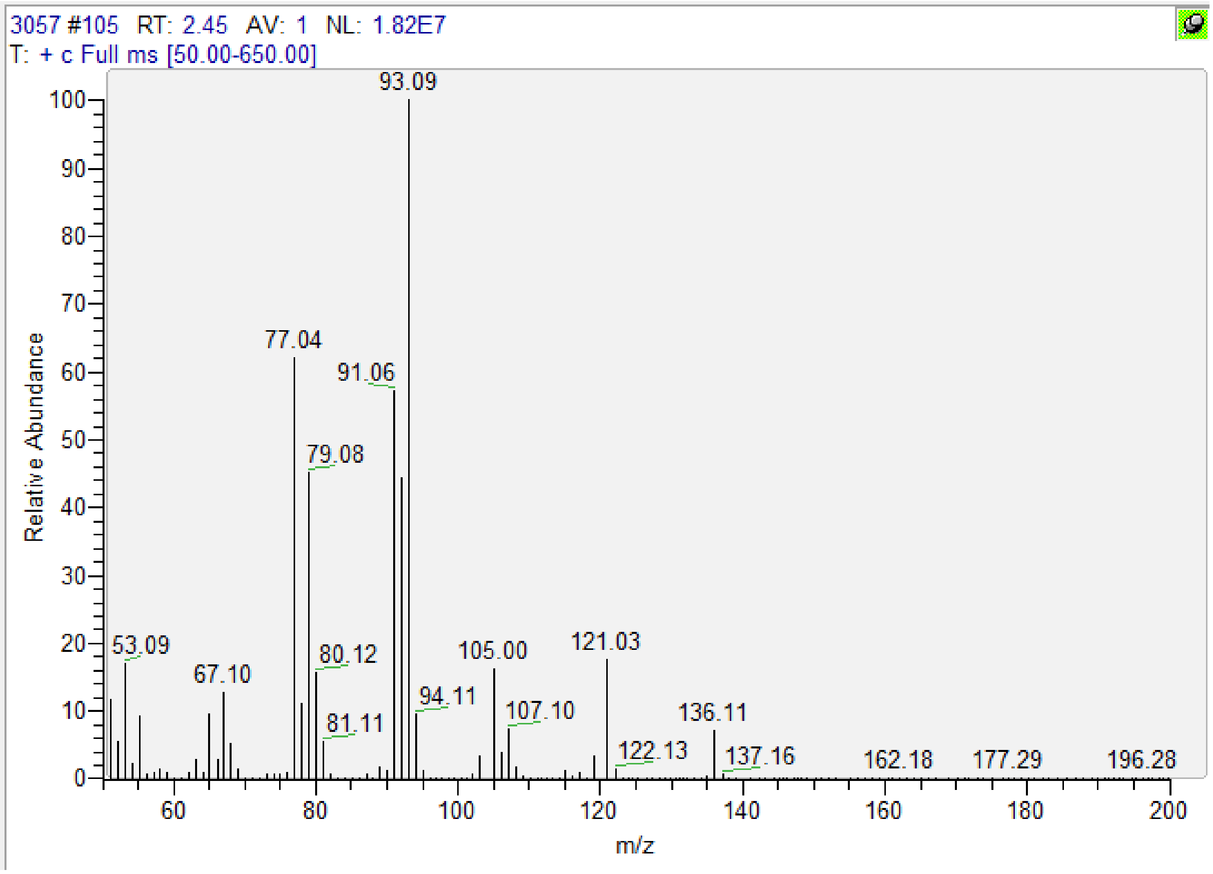

3057 #105 RT: 2.45 AV: 1 NL: 1.82E7
T: + c Full ms [50.00-650.00]
Relative Abundance
m/z
93.09
77.04
91.06
79.08
53.09
67.10
80.12
81.11
94.11
105.00
107.10
121.03
122.13
136.11
137.16
162.18
177.29
196.28

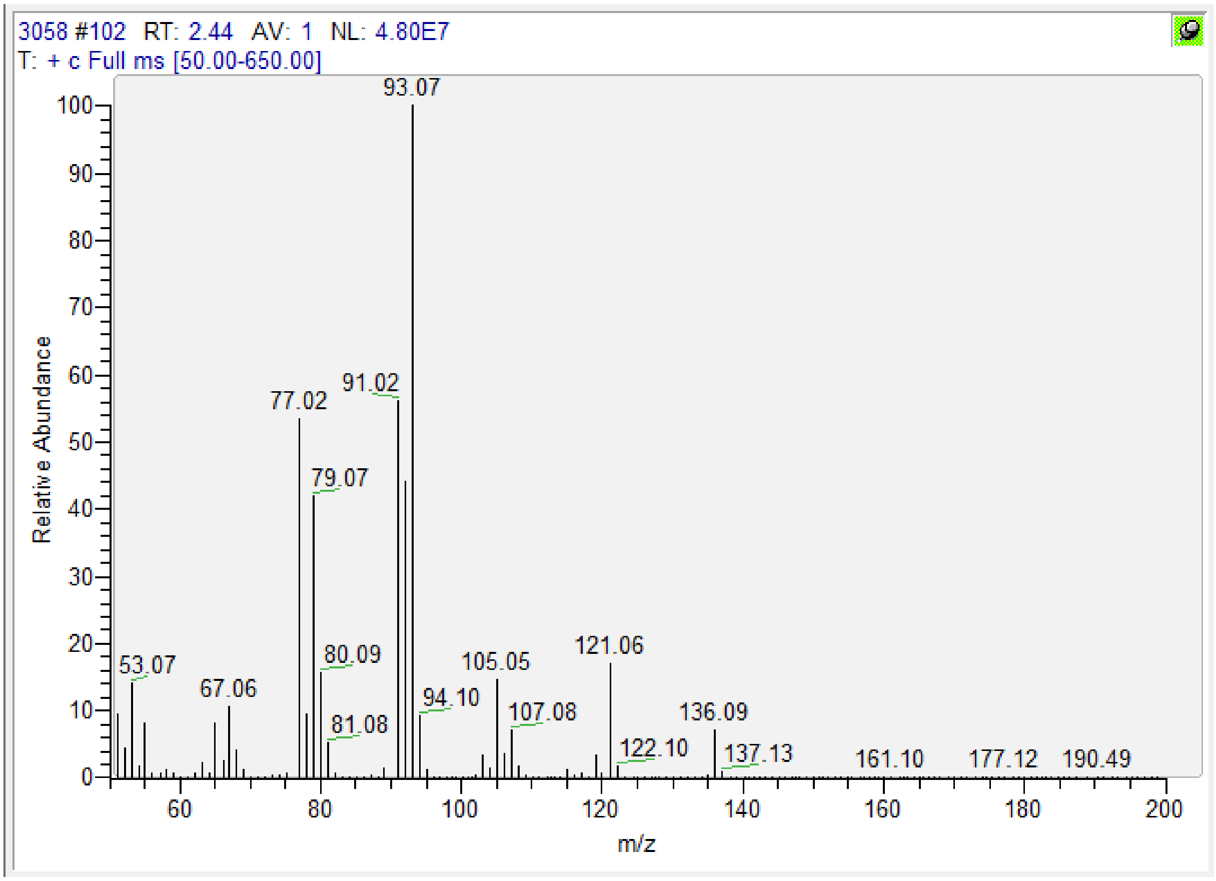
3058 #102 RT: 2.44 AV: 1 NL: 4.80E7
T: + c Full ms [50.00-650.00]
Relative Abundance
m/z
93.07
91.02
77.02
79.07
80.09
53.07
67.06
81.08
94.10
105.05
107.08
121.06
122.10
136.09
137.13
161.10
177.12
190.49

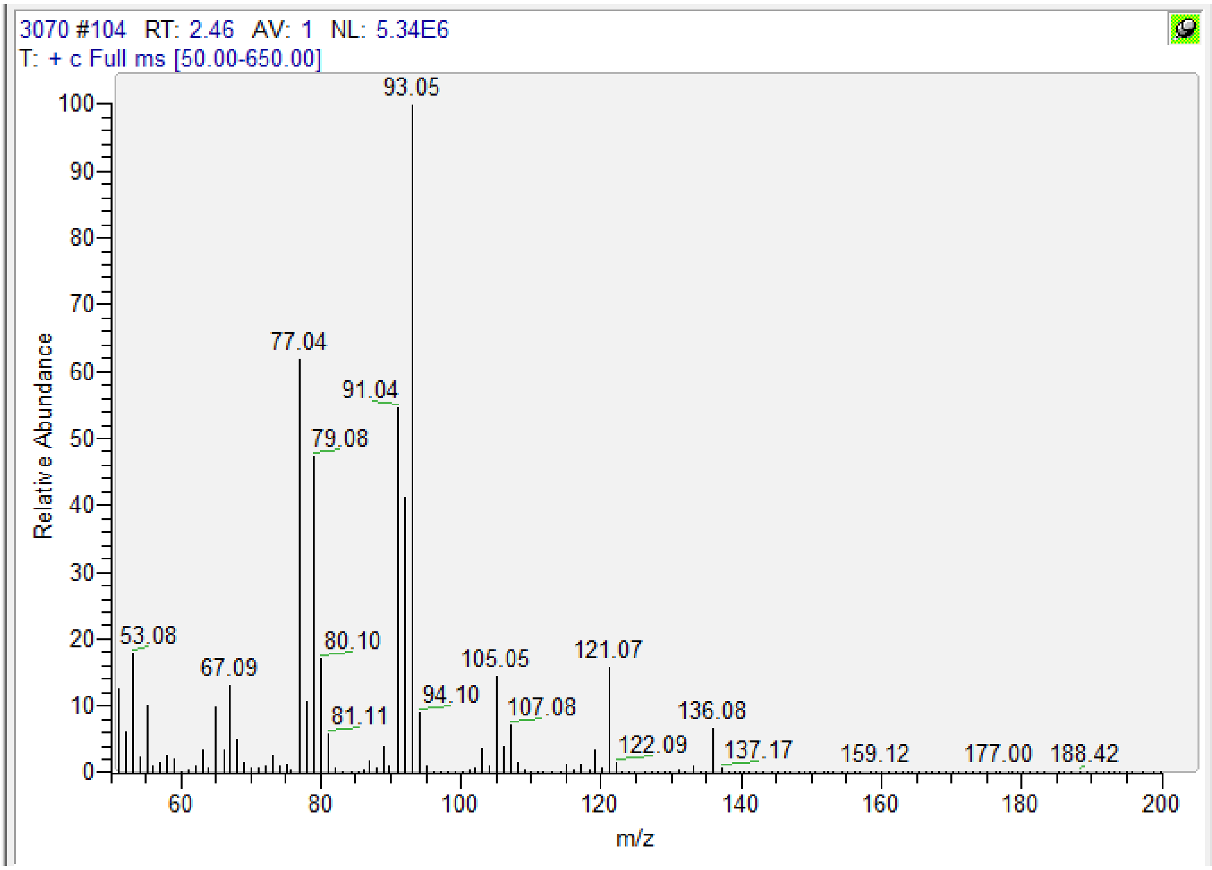
3070 #104 RT: 2.46 AV: 1 NL: 5.34E6
T: + c Full ms [50.00-650.00]
Relative Abundance
m/z
93.05
77.04
91.04
79.08
53.08
67.09
80.10
105.05
121.07
94.10
107.08
81.11
136.08
122.09
137.17
159.12
177.00
188.42

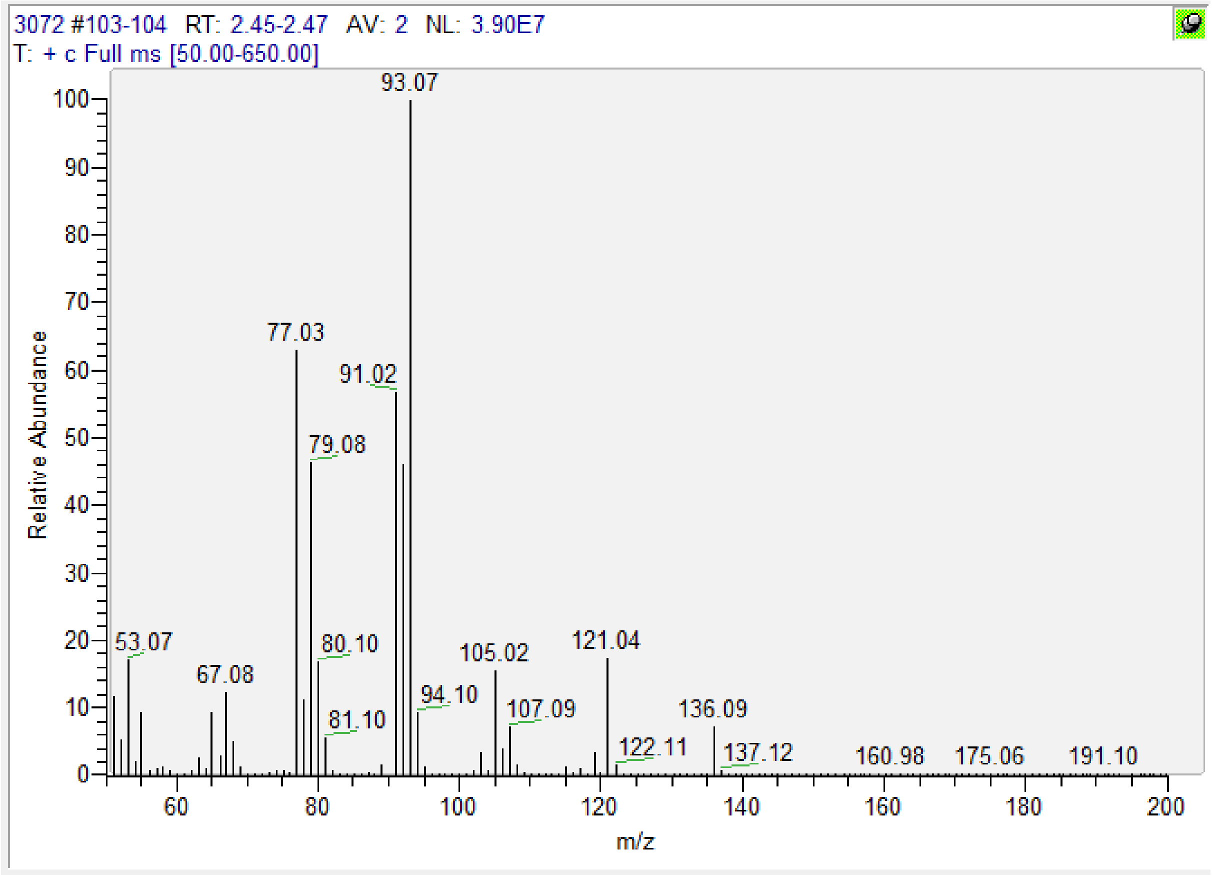

3072 #103-104 RT: 2.45-2.47 AV: 2 NL: 3.90E7
T: + c Full ms [50.00-650.00]
Relative Abundance
m/z
93.07
77.03
91.02
79.08
53.07
67.08
80.10
81.10
94.10
105.02
107.09
121.04
122.11
136.09
137.12
160.98
175.06
191.10

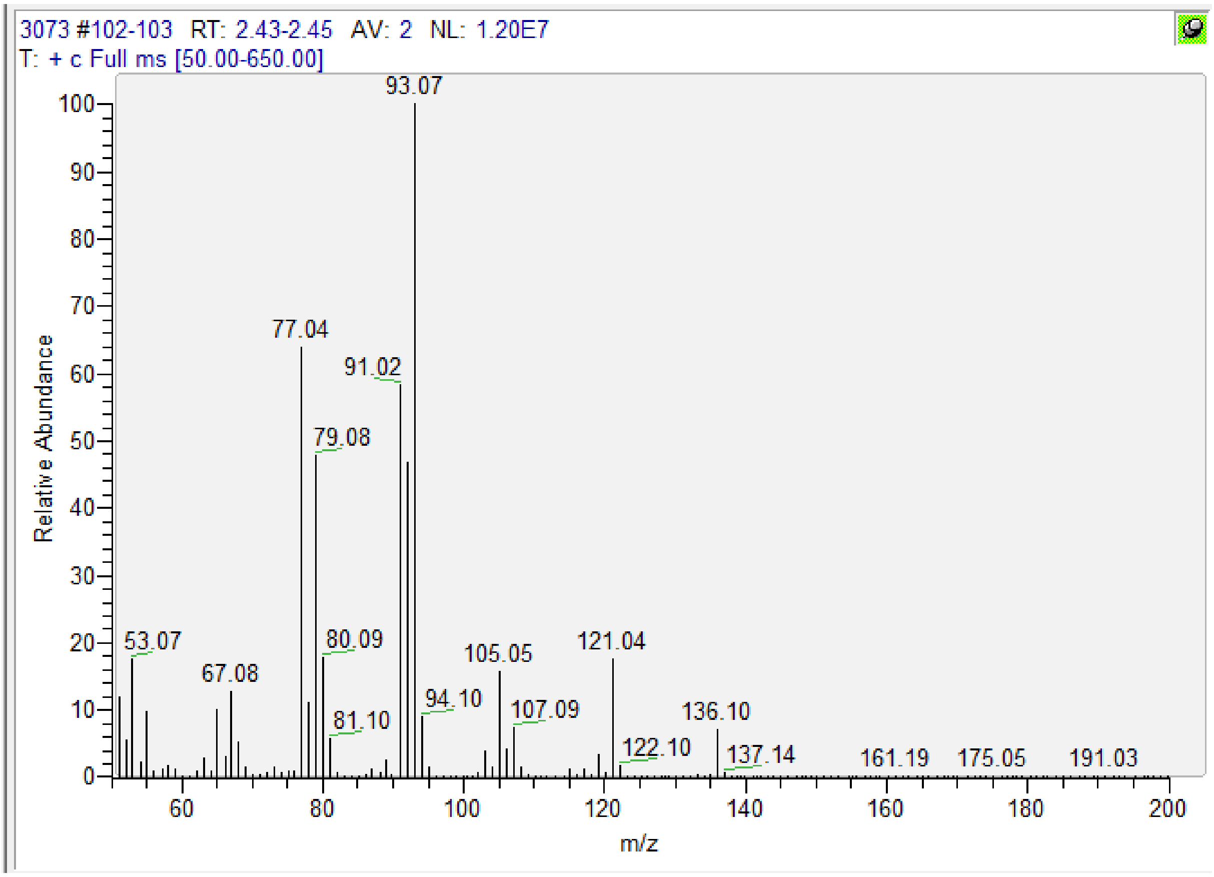

3073 #102-103 RT: 2.43-2.45 AV: 2 NL: 1.20E7
T: + c Full ms [50.00-650.00]
Relative Abundance
m/z
93.07
77.04
91.02
79.08
53.07
67.08
80.09
81.10
94.10
105.05
107.09
121.04
122.10
136.10
137.14
161.19
175.05
191.03

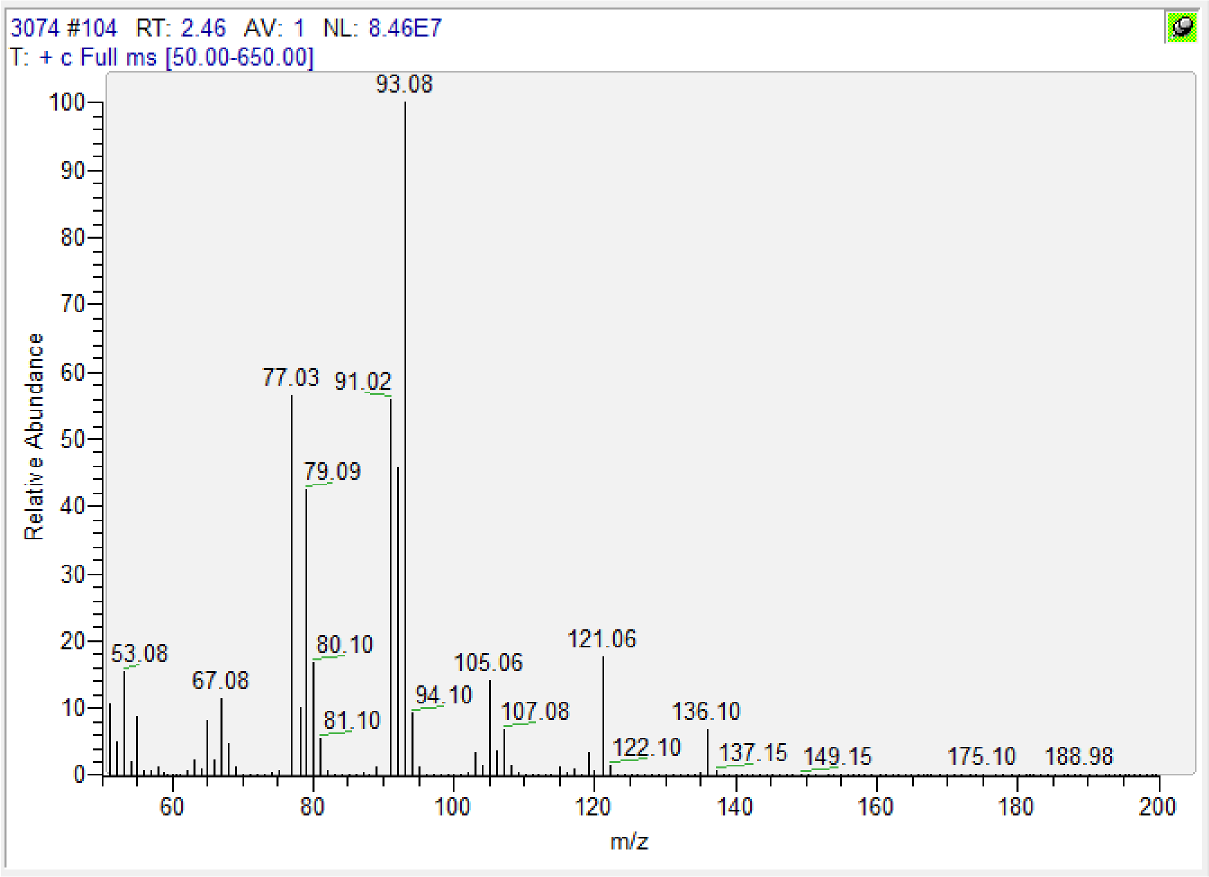
3074 #104 RT: 2.46 AV: 1 NL: 8.46E7
T: + c Full ms [50.00-650.00]
Relative Abundance
m/z
93.08
77.03
91.02
79.09
80.10
53.08
67.08
81.10
94.10
105.06
107.08
121.06
122.10
136.10
137.15
149.15
175.10
188.98

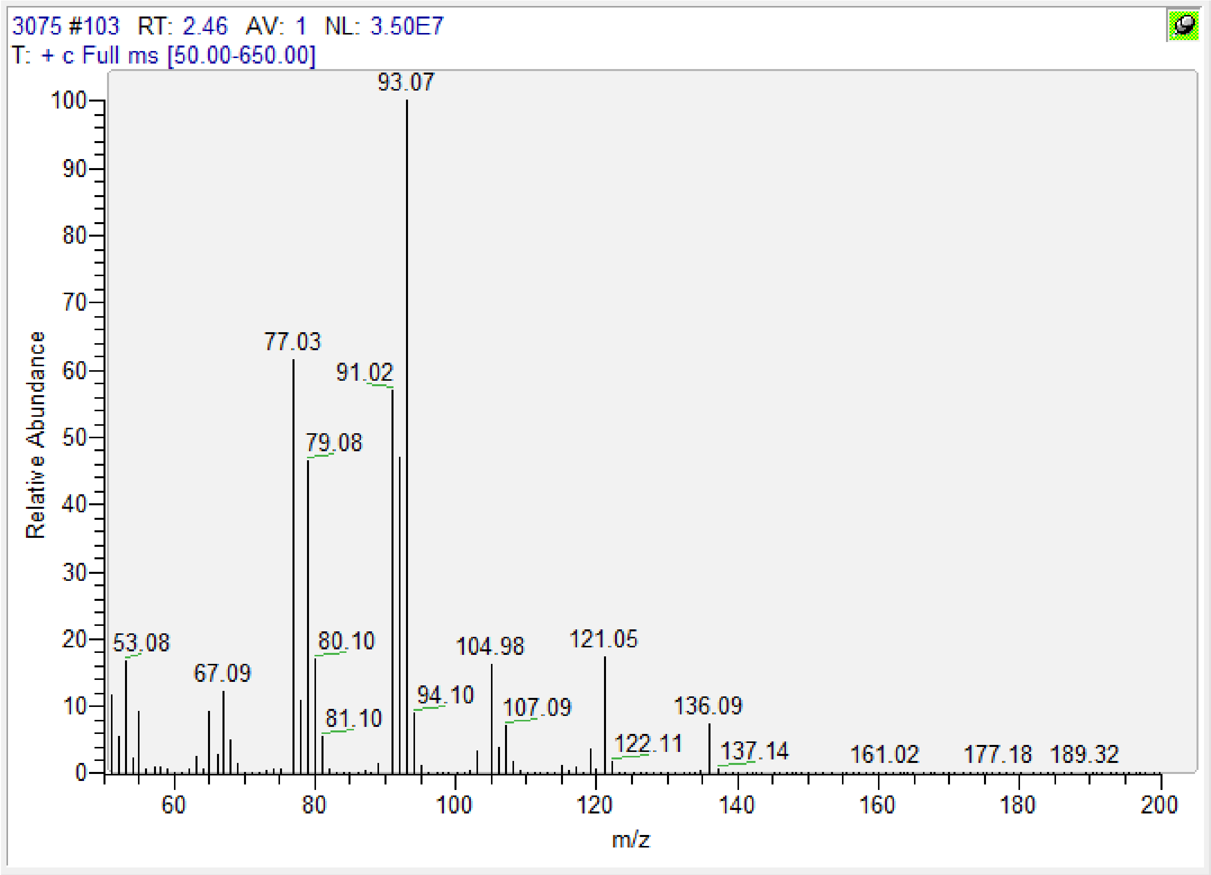

Camphene

Reference

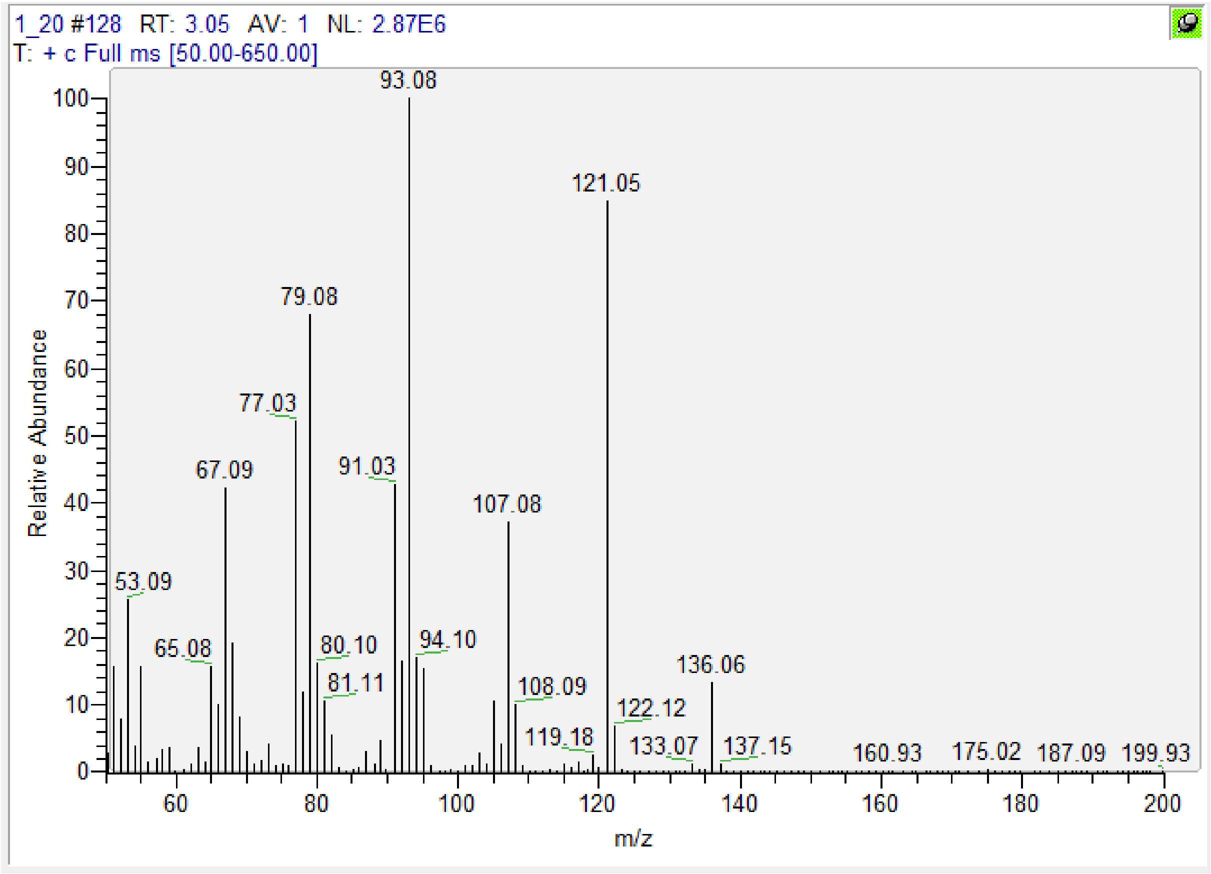
1_20 #128 RT: 3.05 AV: 1 NL: 2.87E6
T: + c Full ms [50.00-650.00]
Relative Abundance
m/z
93.08
121.05
79.08
77.03
67.09
91.03
107.08
53.09
65.08
80.10
94.10
81.11
108.09
136.06
122.12
119.18
133.07
137.15
160.93
175.02
187.09
199.93

Samples:

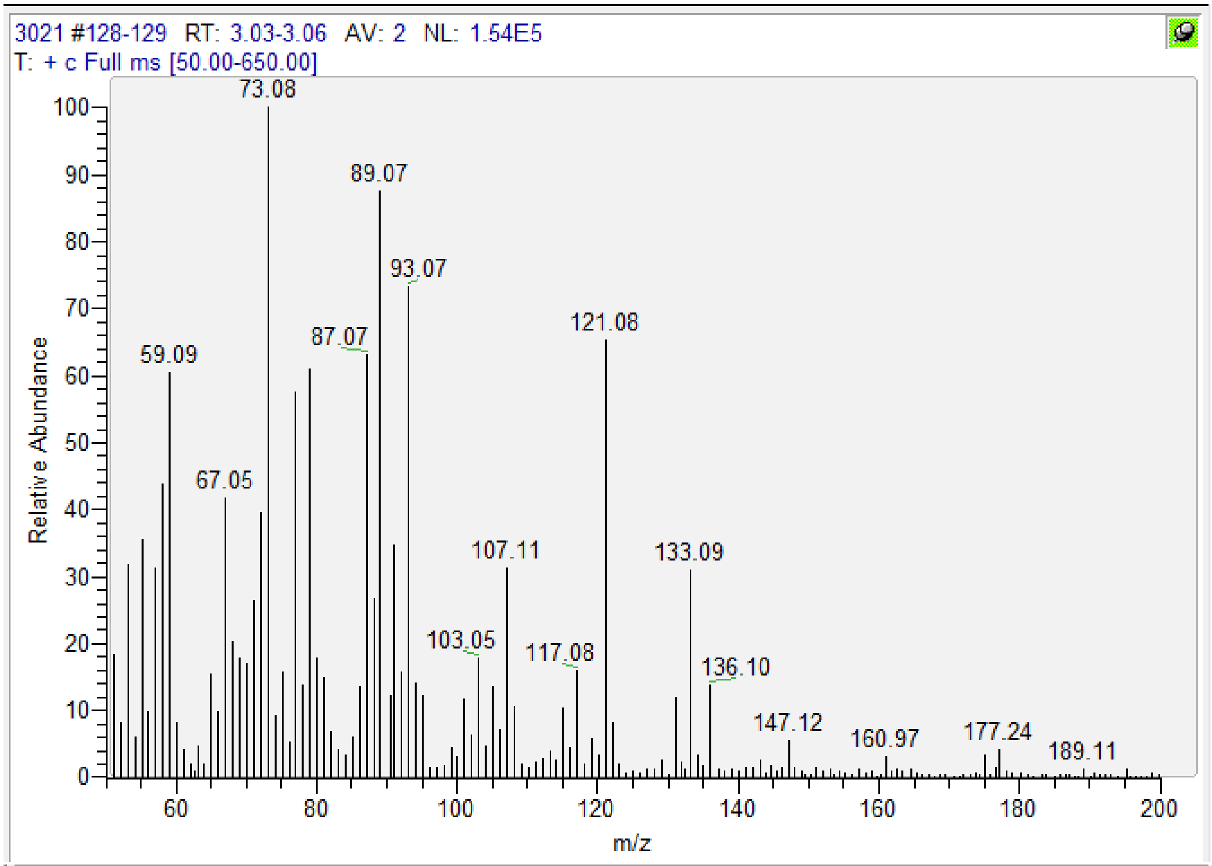
3021 #128-129 RT: 3.03-3.06 AV: 2 NL: 1.54E5
T: + c Full ms [50.00-650.00]
73.08
89.07
93.07
121.08
87.07
59.09
67.05
107.11
133.09
103.05
117.08
136.10
147.12
160.97
177.24
189.11
Relative Abundance
m/z

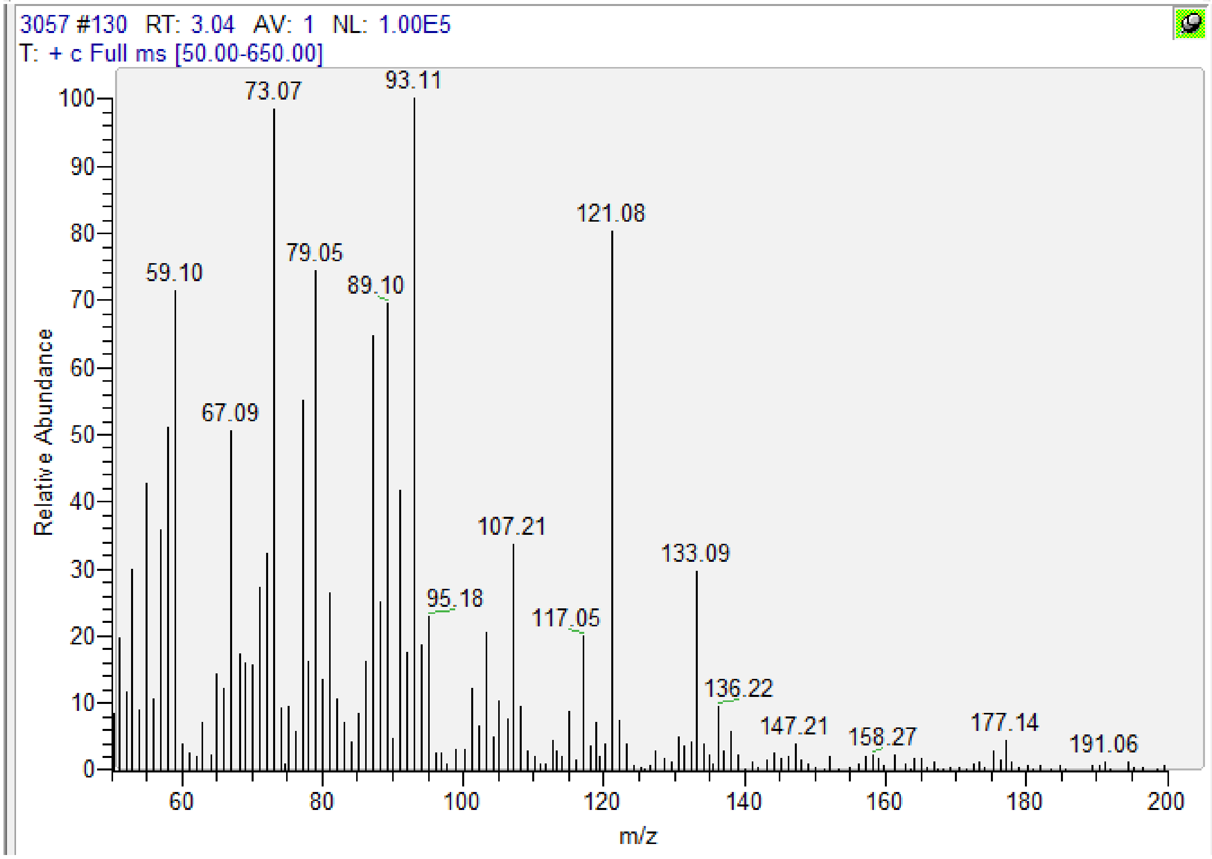
3057 #130 RT: 3.04 AV: 1 NL: 1.00E5
T: + c Full ms [50.00-650.00]
73.07
93.11
121.08
59.10
79.05
89.10
67.09
107.21
133.09
95.18
117.05
136.22
147.21
158.27
177.14
191.06
Relative Abundance
m/z

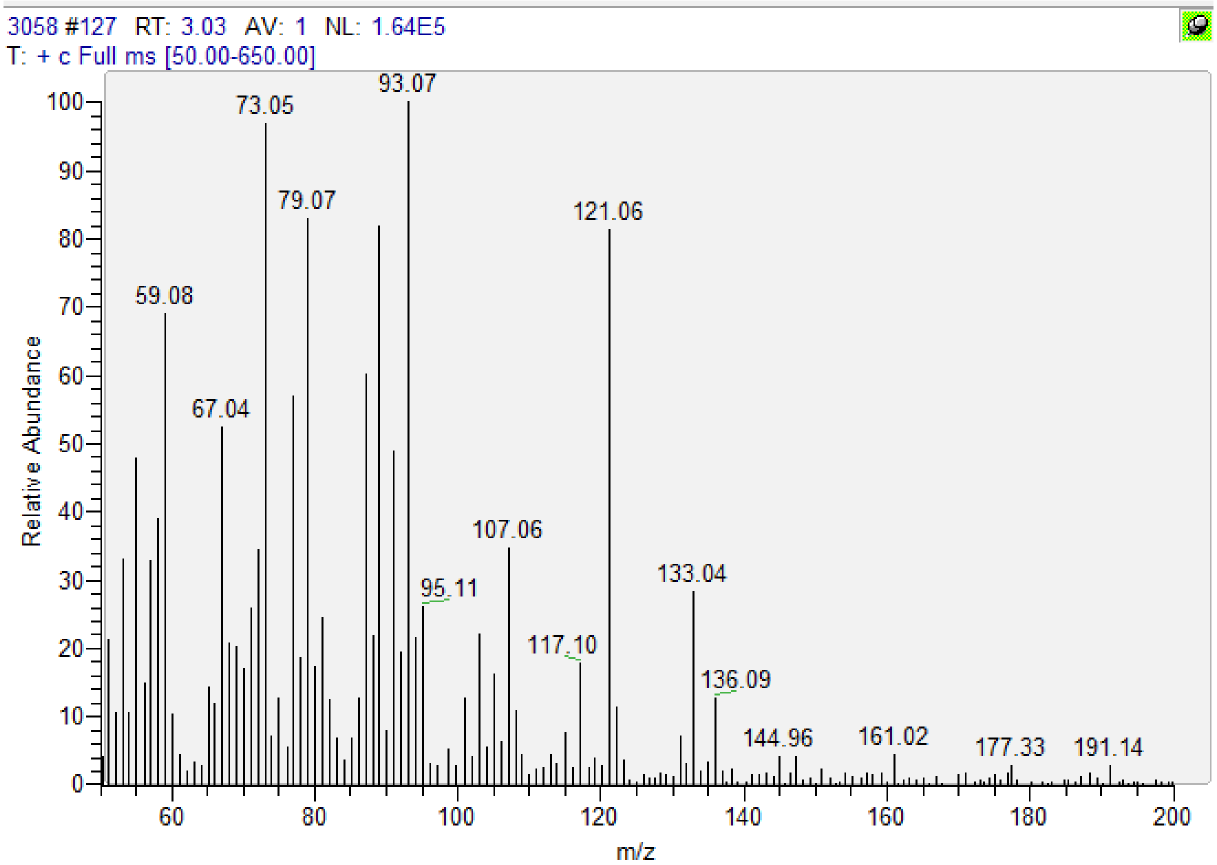
3058 #127 RT: 3.03 AV: 1 NL: 1.64E5
T: + c Full ms [50.00-650.00]
Relative Abundance
m/z
93.07
73.05
79.07
121.06
59.08
67.04
107.06
95.11
133.04
117.10
136.09
144.96
161.02
177.33
191.14

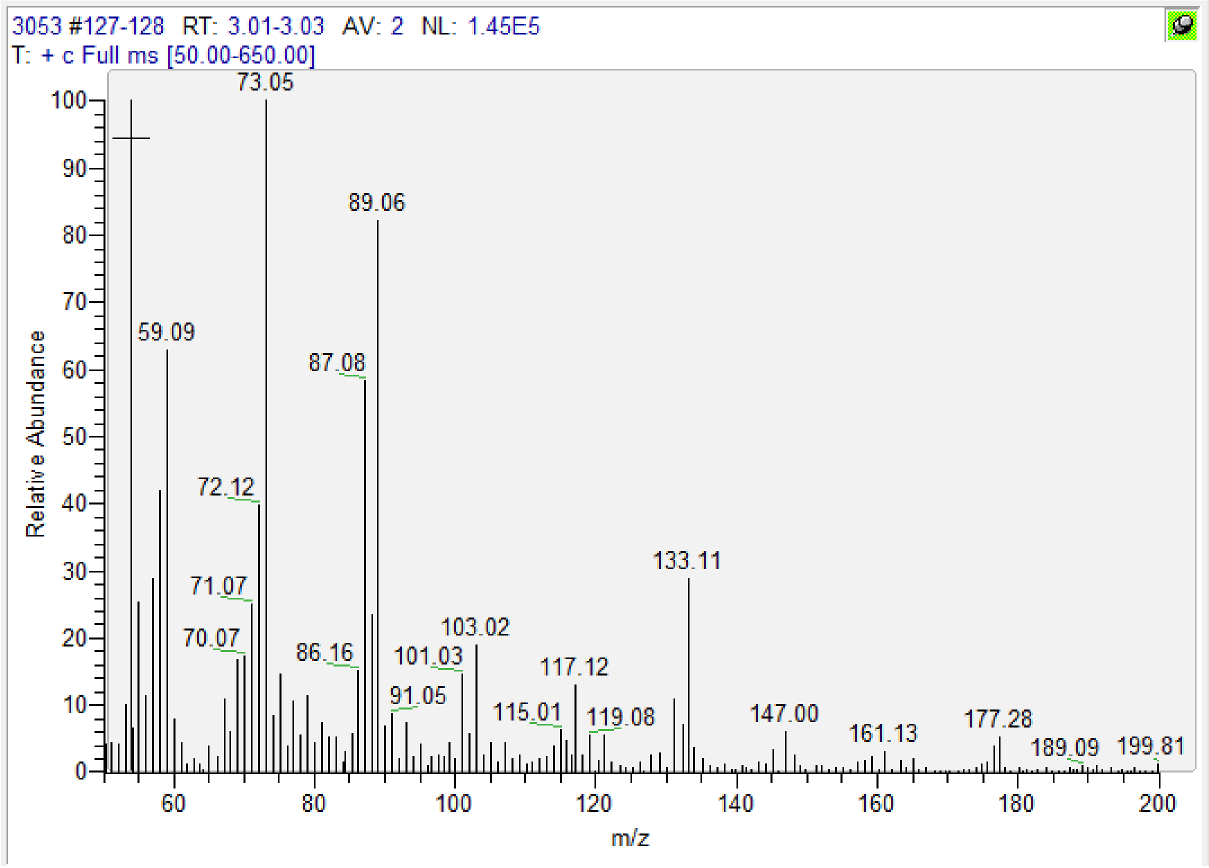
3053 #127-128 RT: 3.01-3.03 AV: 2 NL: 1.45E5
T: + c Full ms [50.00-650.00]
73.05
89.06
59.09
87.08
72.12
71.07
70.07
86.16
101.03
103.02
91.05
115.01
117.12
119.08
133.11
147.00
161.13
177.28
189.09
199.81
Relative Abundance
m/z

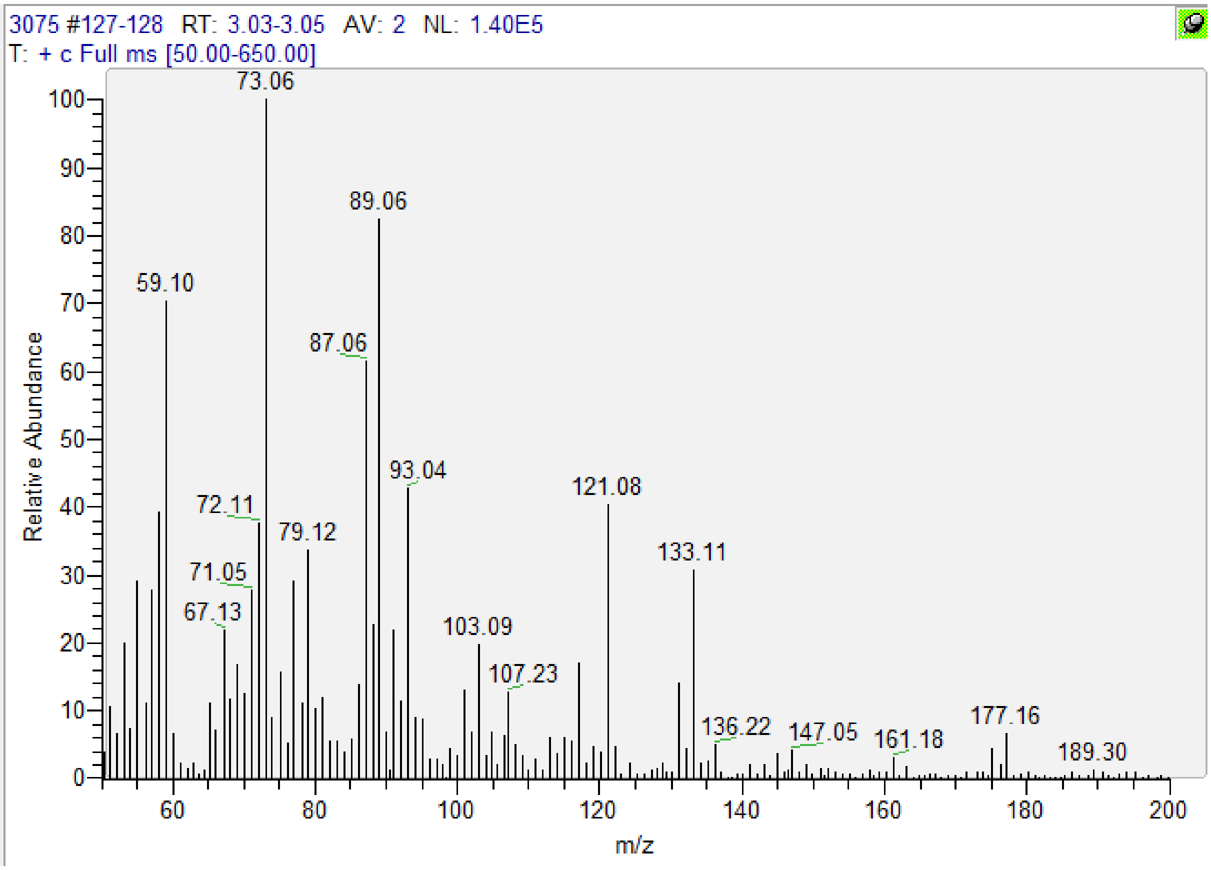
3075 #127-128 RT: 3.03-3.05 AV: 2 NL: 1.40E5
T: + c Full ms [50.00-650.00]
73.06
89.06
59.10
87.06
93.04
121.08
72.11
79.12
133.11
71.05
67.13
103.09
107.23
136.22
147.05
161.18
177.16
189.30
Relative Abundance
m/z

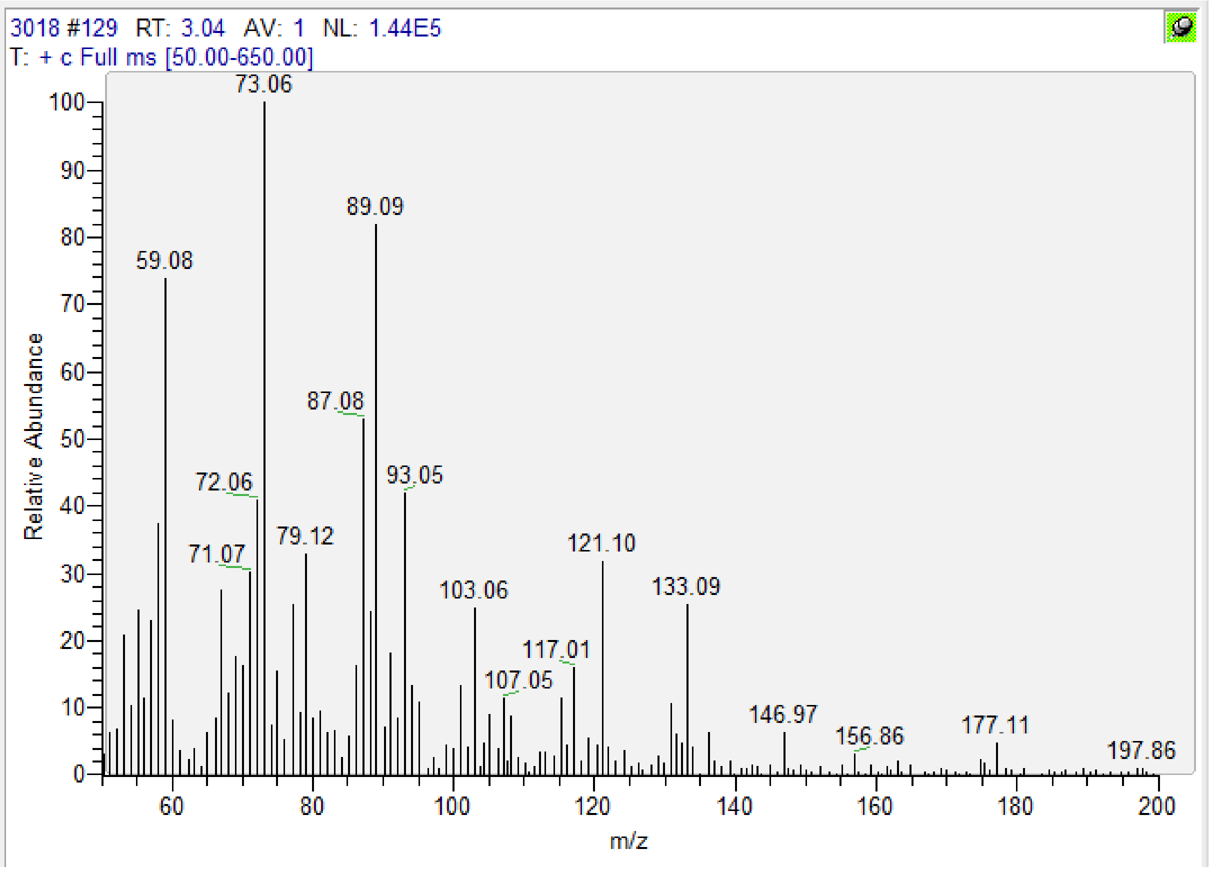
3018 #129 RT: 3.04 AV: 1 NL: 1.44E5
T: + c Full ms [50.00-650.00]
Relative Abundance
m/z
73.06
89.09
59.08
87.08
72.06
93.05
71.07
79.12
121.10
103.06
133.09
117.01
107.05
146.97
156.86
177.11
197.86

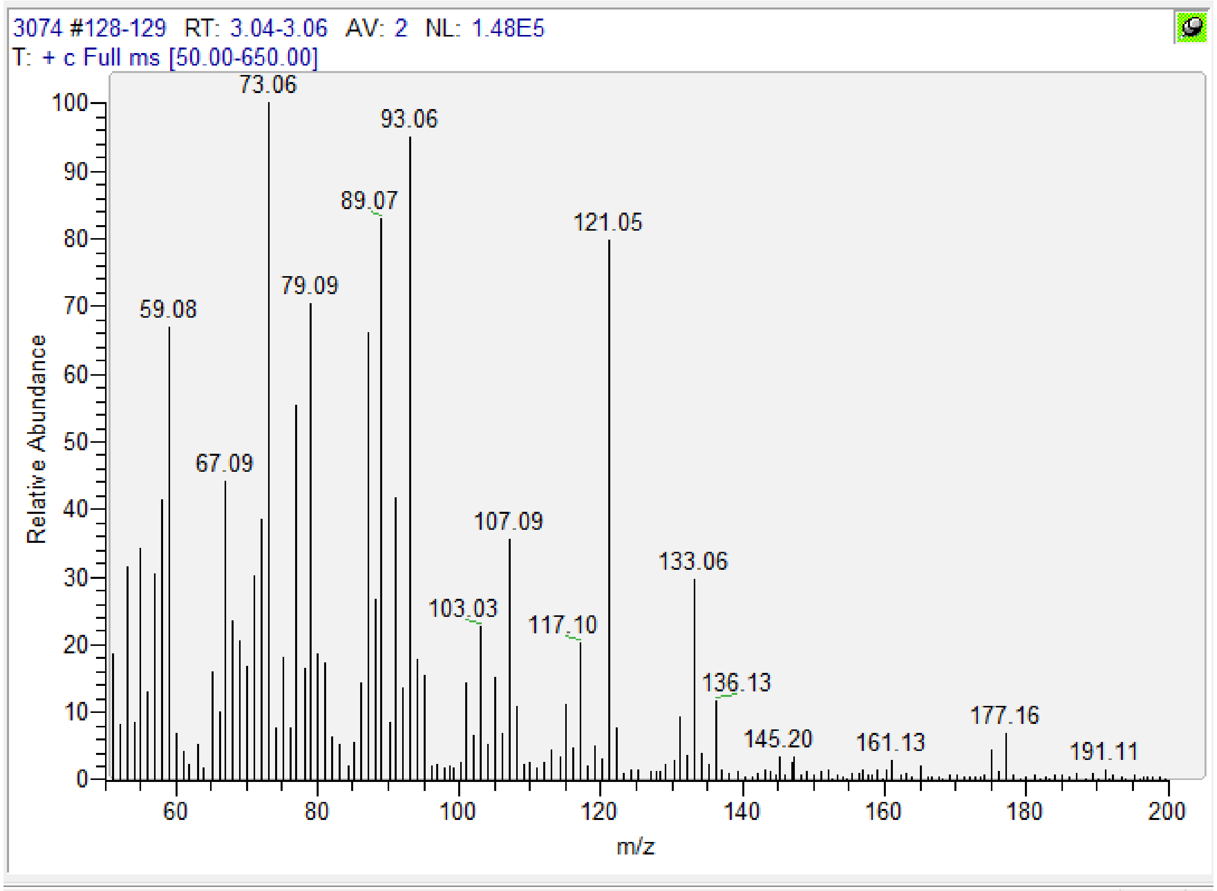
3074 #128-129 RT: 3.04-3.06 AV: 2 NL: 1.48E5
T: + c Full ms [50.00-650.00]
Relative Abundance
m/z
73.06
93.06
89.07
121.05
79.09
59.08
67.09
107.09
133.06
103.03
117.10
136.13
145.20
161.13
177.16
191.11

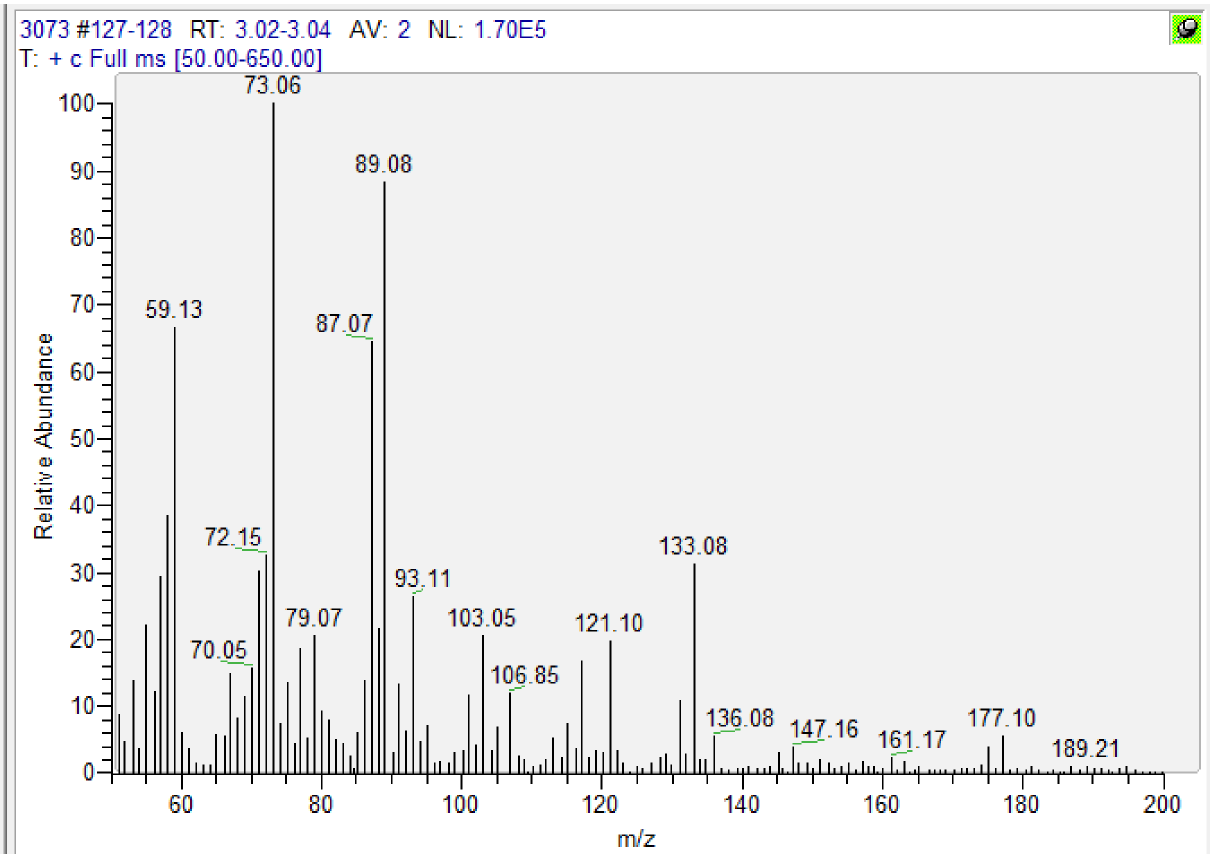

3073 #127-128 RT: 3.02-3.04 AV: 2 NL: 1.70E5
T: + c Full ms [50.00-650.00]
Relative Abundance
m/z
73.06
89.08
59.13
87.07
72.15
70.05
79.07
93.11
103.05
106.85
121.10
133.08
136.08
147.16
161.17
177.10
189.21

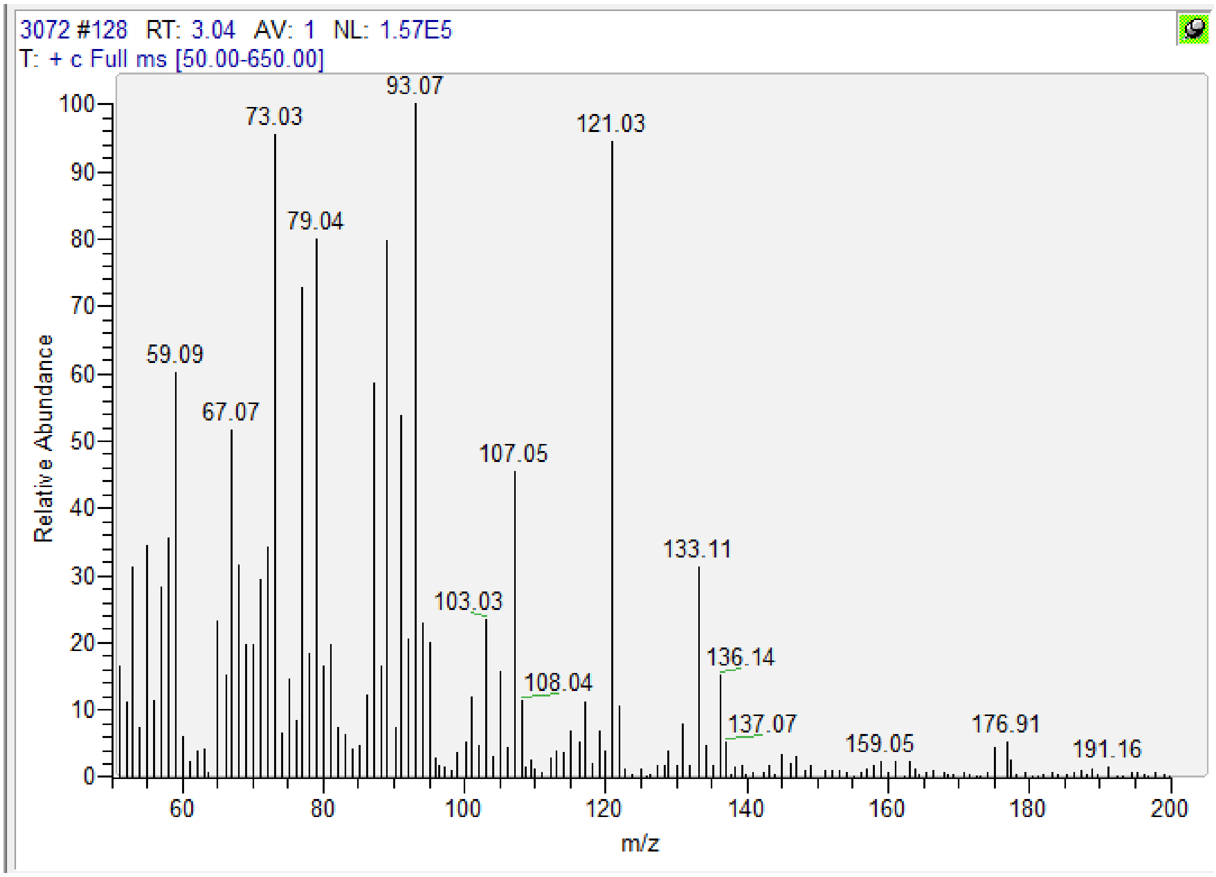
3072 #128 RT: 3.04 AV: 1 NL: 1.57E5
T: + c Full ms [50.00-650.00]
Relative Abundance
m/z
93.07
73.03
121.03
79.04
59.09
67.07
107.05
133.11
103.03
136.14
108.04
137.07
159.05
176.91
191.16

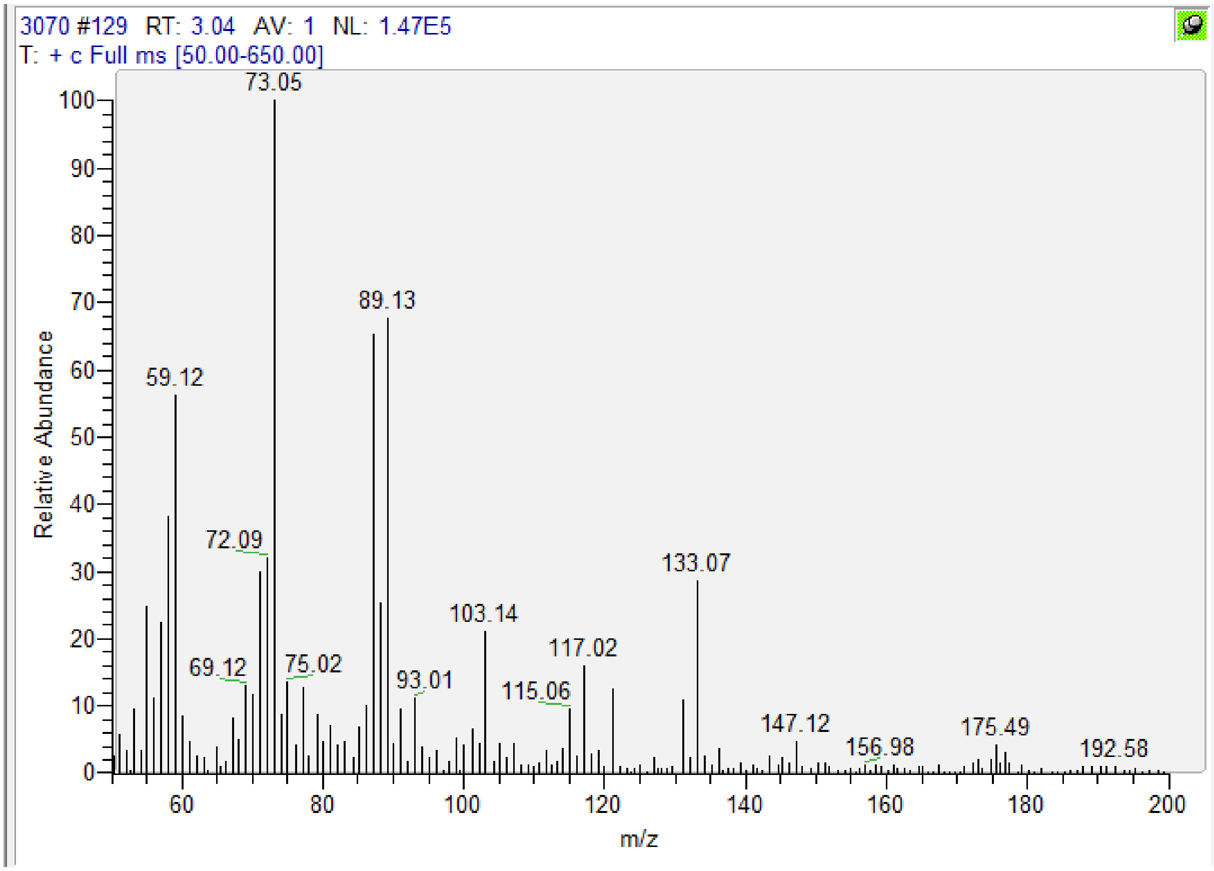

3070 #129 RT: 3.04 AV: 1 NL: 1.47E5
T: + c Full ms [50.00-650.00]
73.05
89.13
59.12
72.09
133.07
103.14
117.02
69.12
75.02
93.01
115.06
147.12
156.98
175.49
192.58
Relative Abundance
m/z

Beta Pinene:

Reference:

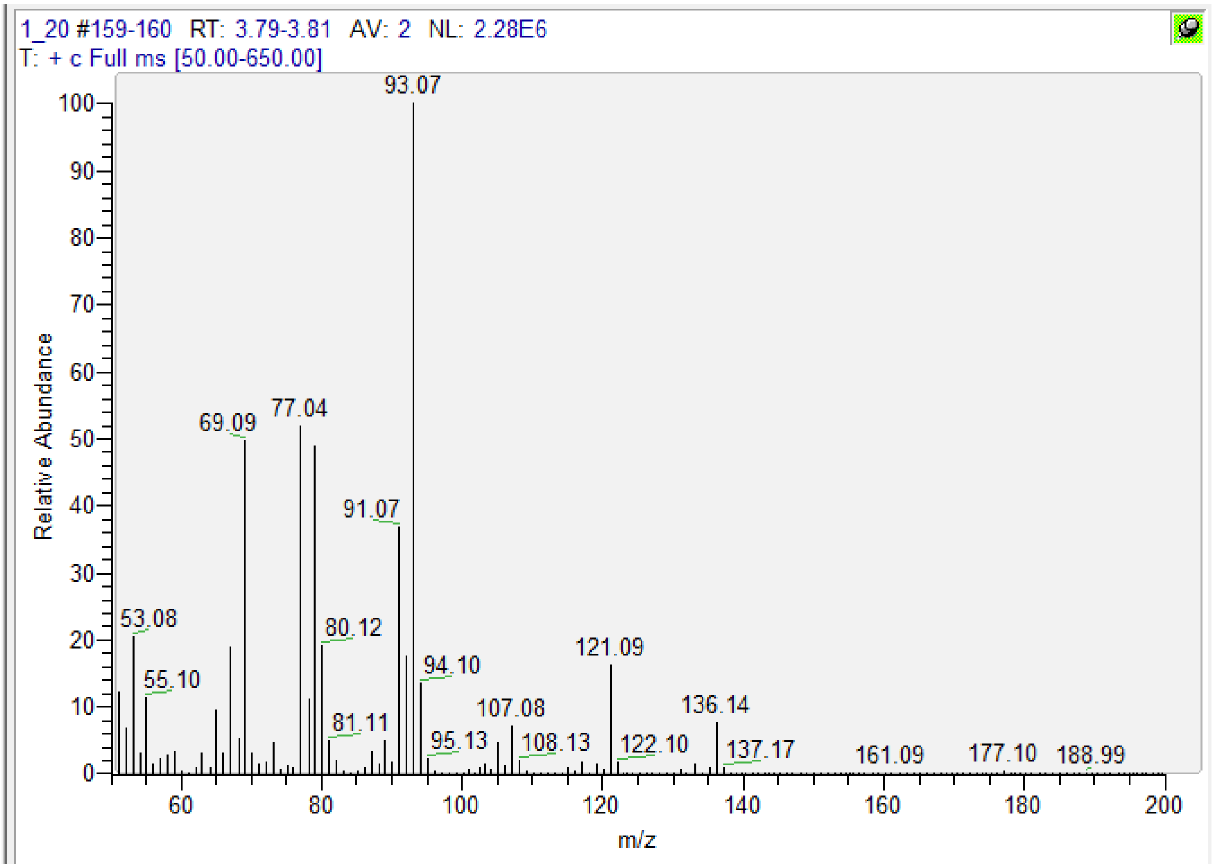

Samples:

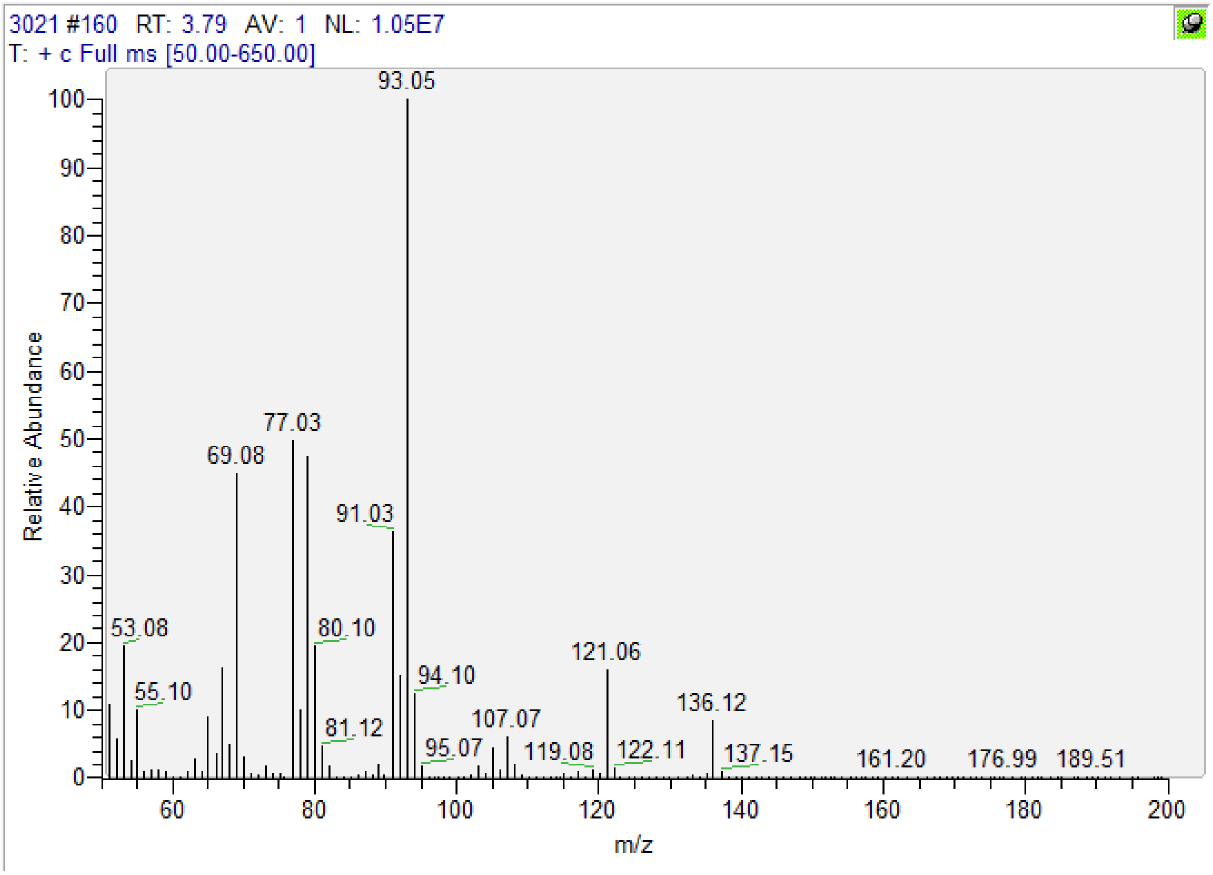

3021 #160 RT: 3.79 AV: 1 NL: 1.05E7
T: + c Full ms [50.00-650.00]
Relative Abundance
m/z
93.05
77.03
69.08
91.03
53.08
80.10
121.06
94.10
55.10
136.12
107.07
81.12
95.07
119.08
122.11
137.15
161.20
176.99
189.51

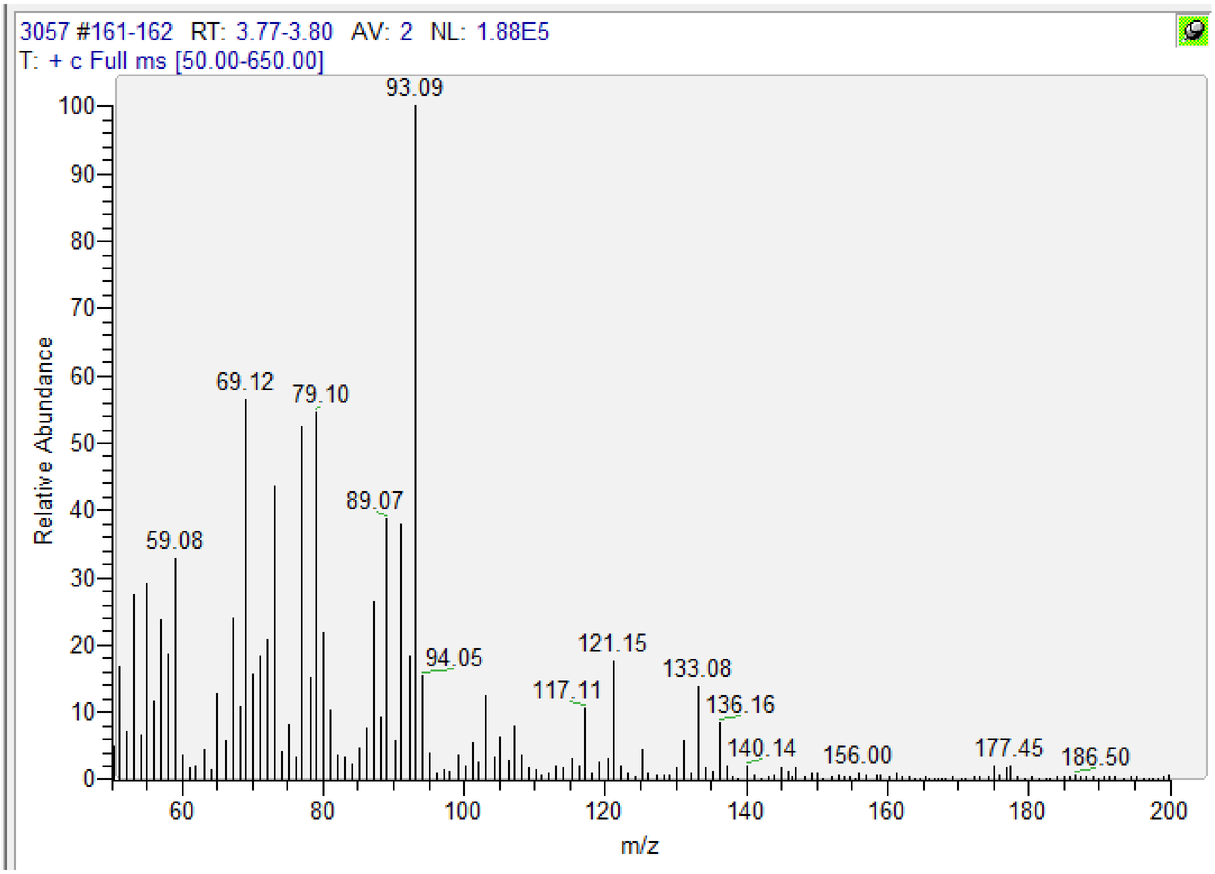

3057 #161-162 RT: 3.77-3.80 AV: 2 NL: 1.88E5
T: + c Full ms [50.00-650.00]
Relative Abundance
m/z
93.09
69.12
79.10
89.07
59.08
94.05
121.15
117.11
133.08
136.16
140.14
156.00
177.45
186.50

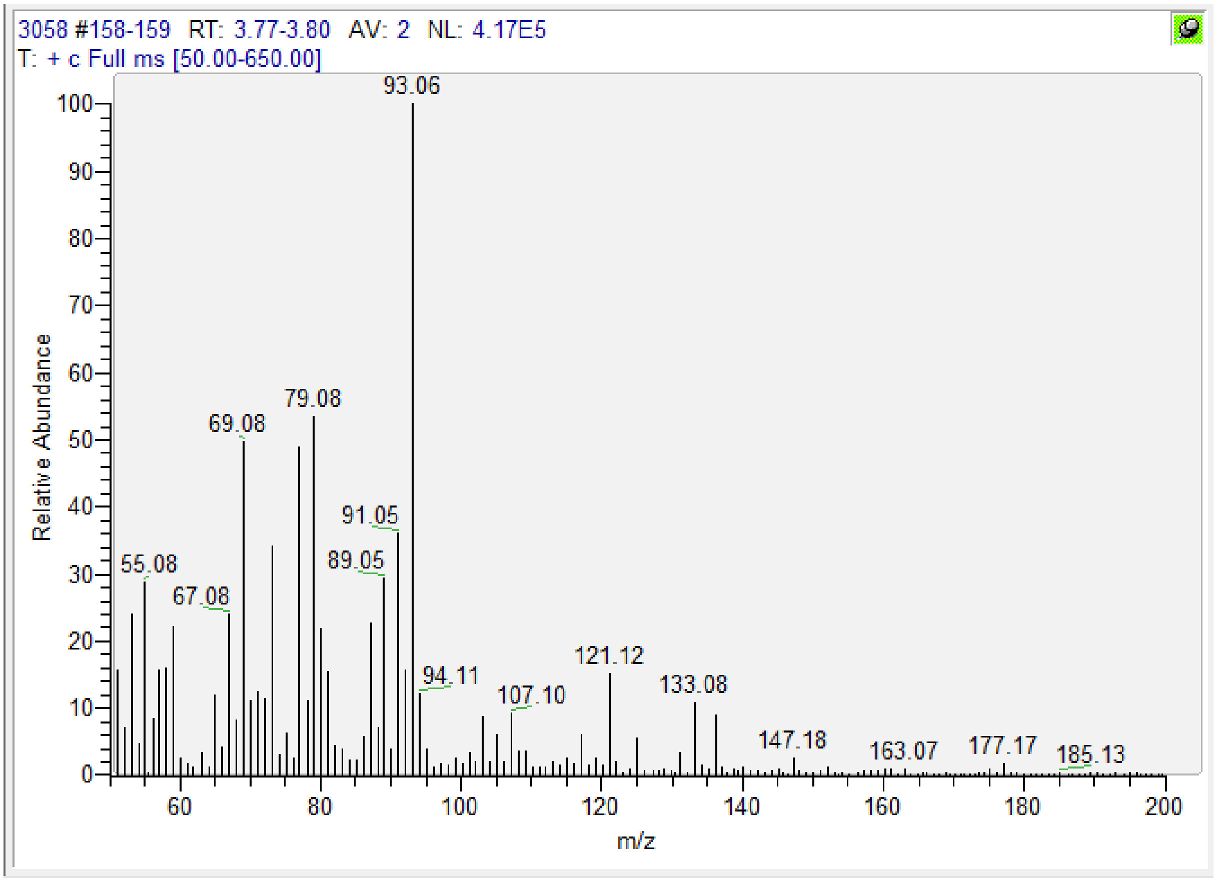
3058 #158-159 RT: 3.77-3.80 AV: 2 NL: 4.17E5
T: + c Full ms [50.00-650.00]
Relative Abundance
m/z
93.06
79.08
69.08
91.05
89.05
55.08
67.08
121.12
94.11
107.10
133.08
147.18
163.07
177.17
185.13

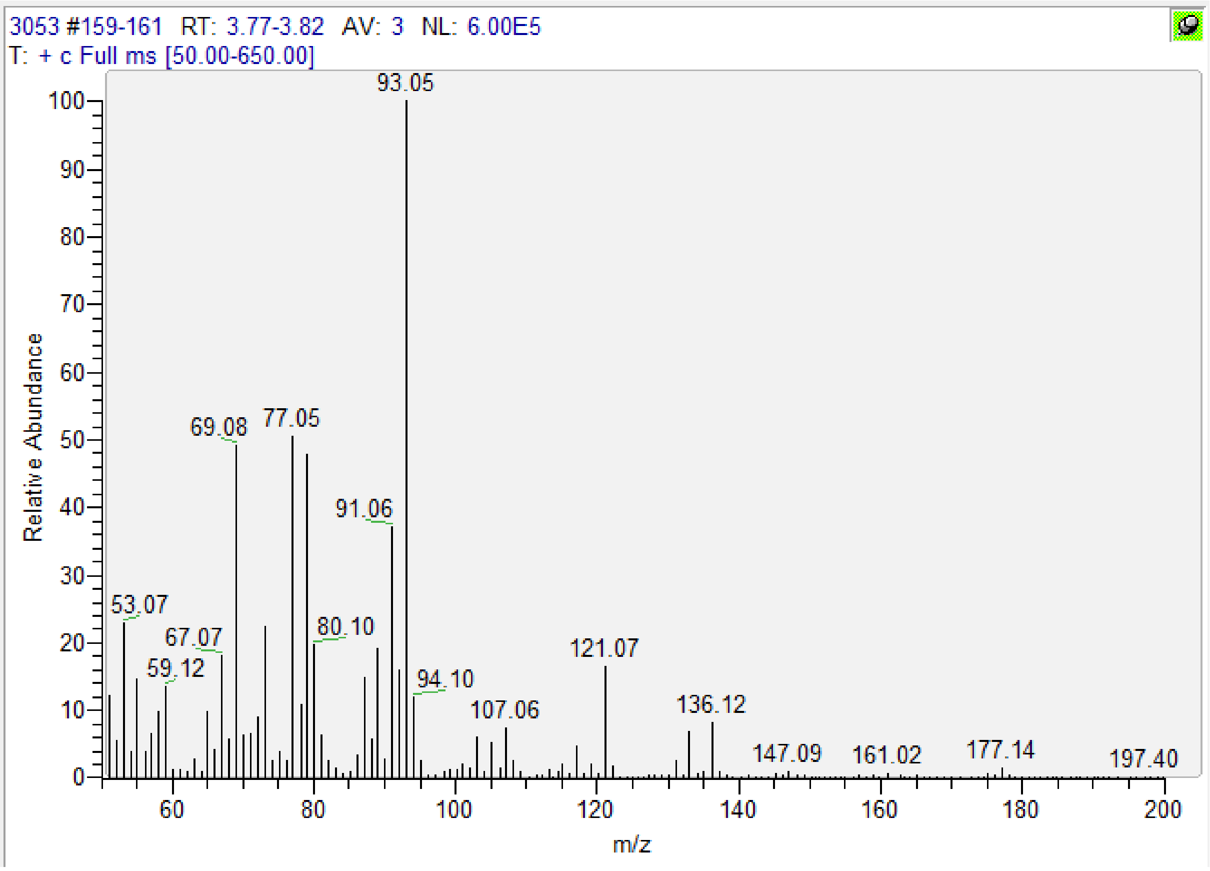

3053 #159-161 RT: 3.77-3.82 AV: 3 NL: 6.00E5
T: + c Full ms [50.00-650.00]
Relative Abundance
m/z
93.05
69.08
77.05
91.06
53.07
67.07
80.10
59.12
94.10
107.06
121.07
136.12
147.09
161.02
177.14
197.40

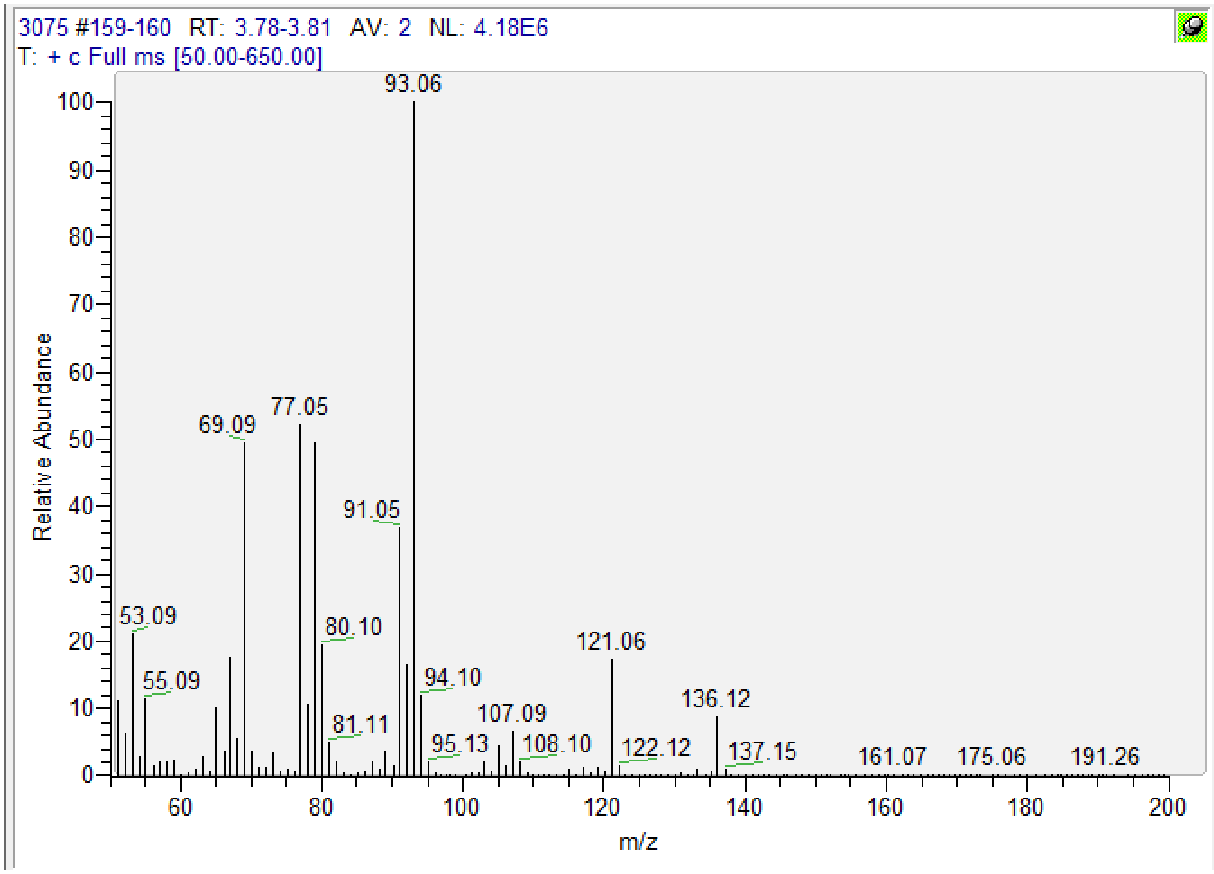

3075 #159-160 RT: 3.78-3.81 AV: 2 NL: 4.18E6
T: + c Full ms [50.00-650.00]
Relative Abundance
m/z
93.06
77.05
69.09
91.05
53.09
80.10
121.06
55.09
94.10
136.12
81.11
107.09
95.13
108.10
122.12
137.15
161.07
175.06
191.26

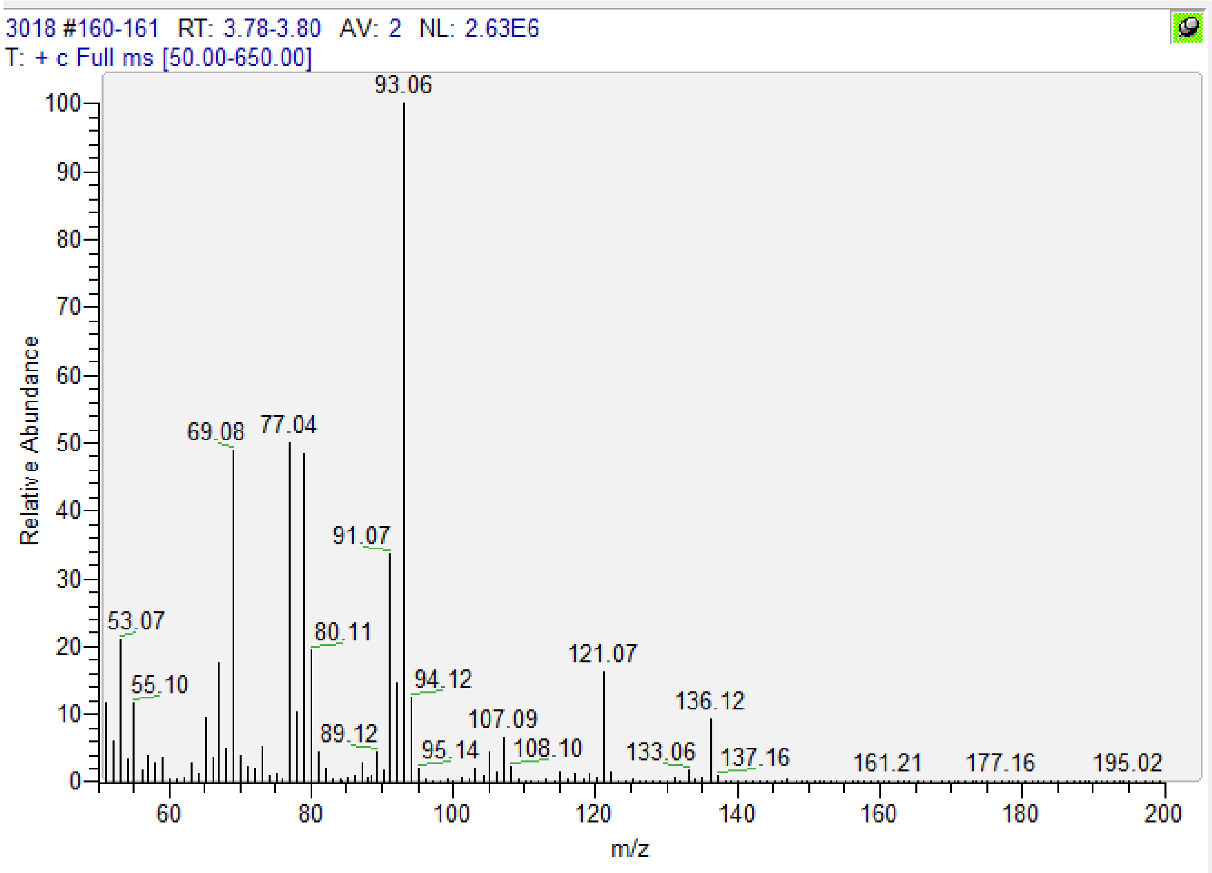
3018 #160-161 RT: 3.78-3.80 AV: 2 NL: 2.63E6
T: + c Full ms [50.00-650.00]
Relative Abundance
m/z
93.06
69.08
77.04
91.07
53.07
80.11
55.10
94.12
121.07
136.12
107.09
89.12
95.14
108.10
133.06
137.16
161.21
177.16
195.02

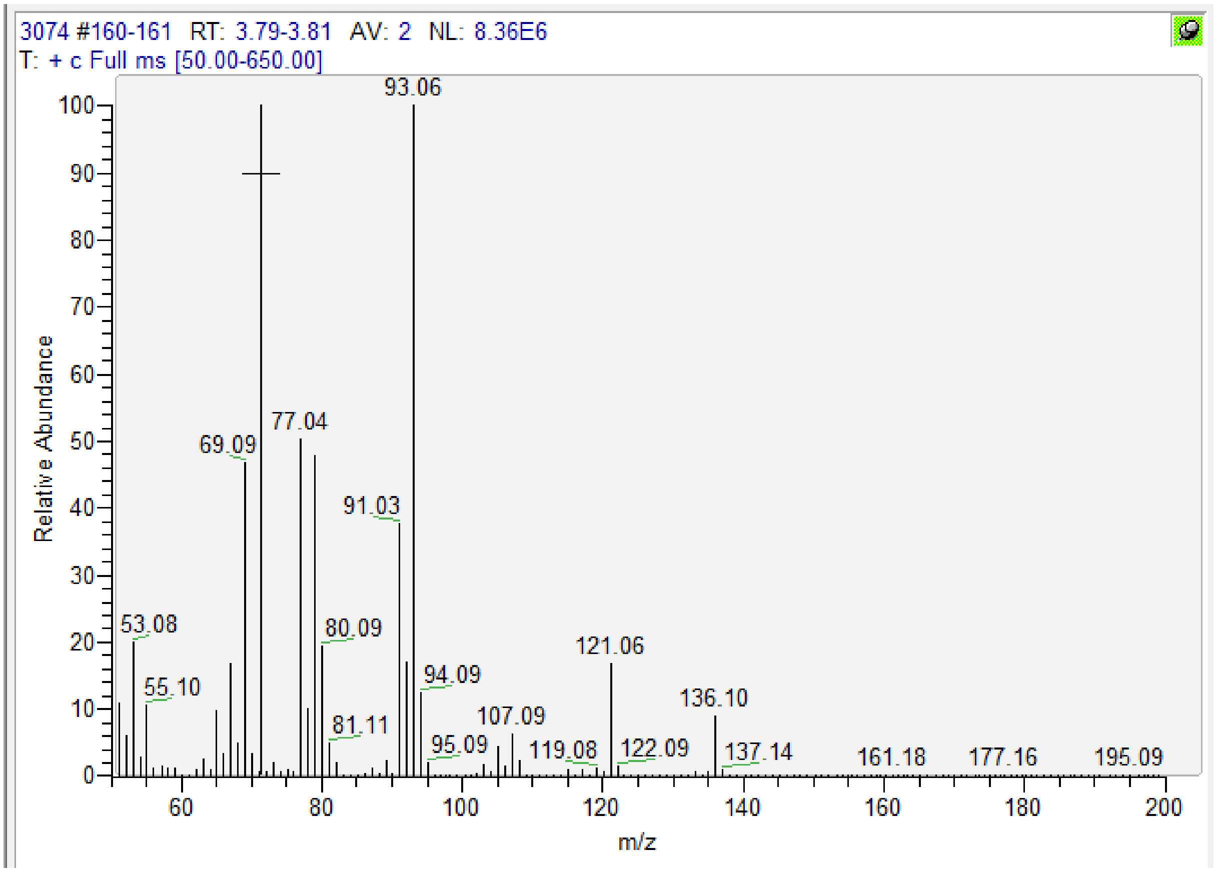
3074 #160-161 RT: 3.79-3.81 AV: 2 NL: 8.36E6
T: + c Full ms [50.00-650.00]
Relative Abundance
m/z
93.06
77.04
69.09
91.03
53.08
80.09
55.10
94.09
121.06
136.10
107.09
81.11
95.09
119.08
122.09
137.14
161.18
177.16
195.09

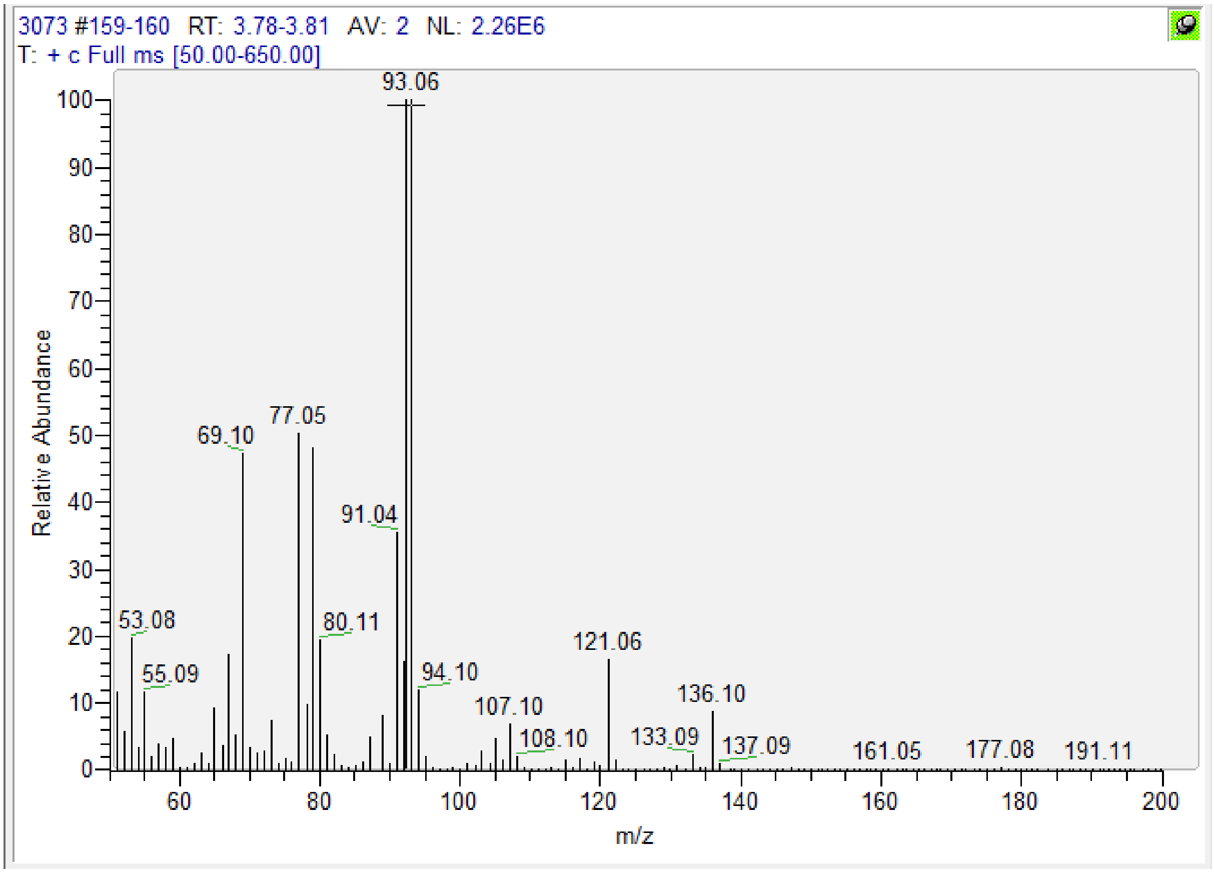

3073 #159-160 RT: 3.78-3.81 AV: 2 NL: 2.26E6
T: + c Full ms [50.00-650.00]
93.06
77.05
69.10
91.04
53.08
80.11
55.09
94.10
121.06
107.10
136.10
108.10
133.09
137.09
161.05
177.08
191.11
Relative Abundance
m/z

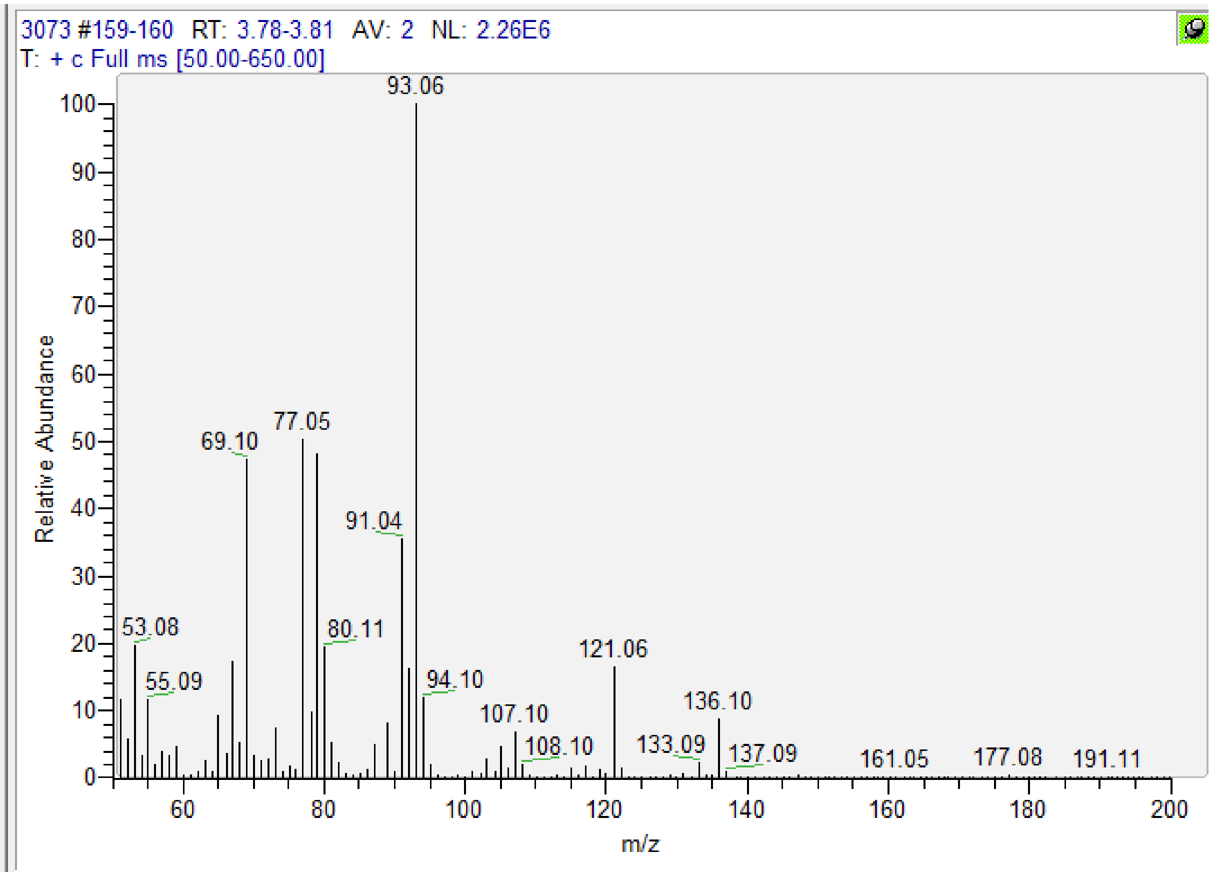

3073 #159-160 RT: 3.78-3.81 AV: 2 NL: 2.26E6
T: + c Full ms [50.00-650.00]
Relative Abundance
m/z
93.06
77.05
69.10
91.04
53.08
80.11
121.06
55.09
94.10
136.10
107.10
108.10
133.09
137.09
161.05
177.08
191.11

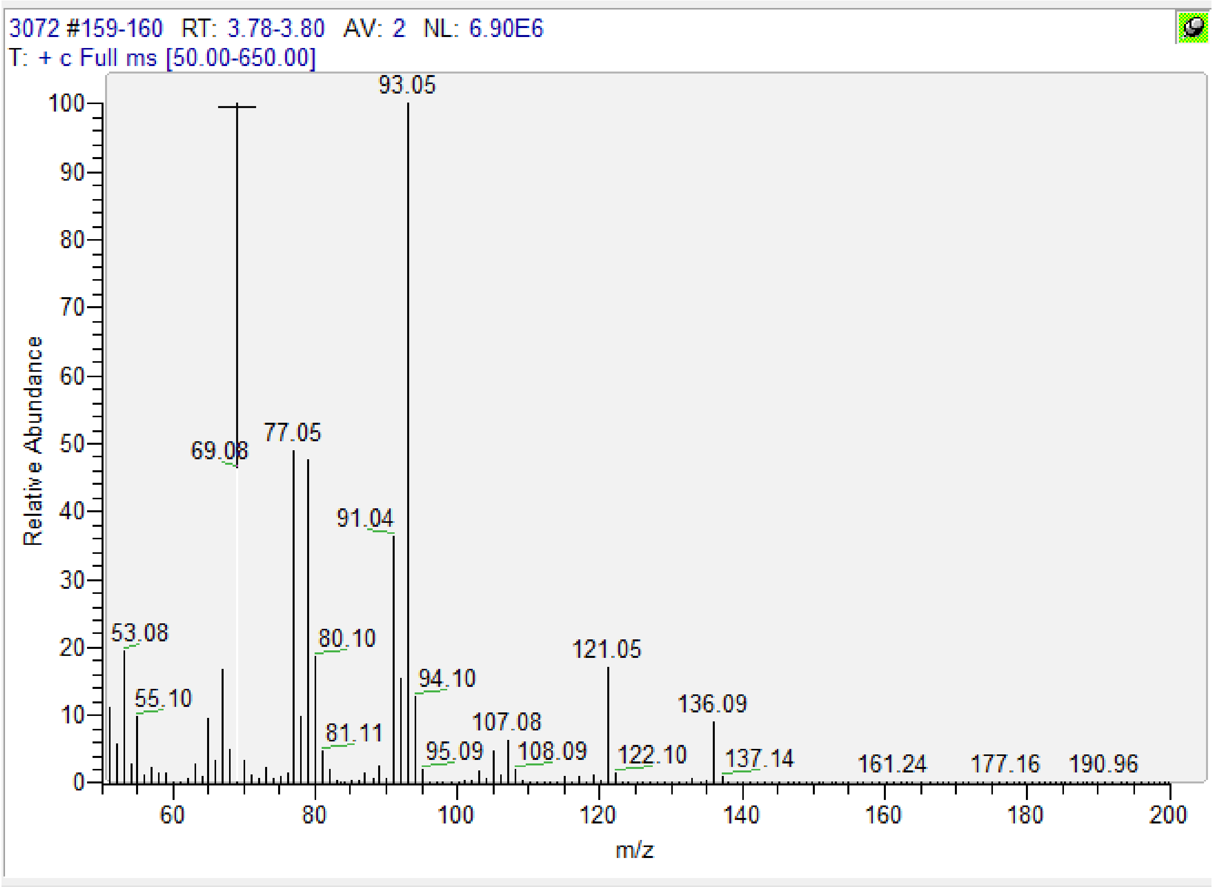
3072 #159-160 RT: 3.78-3.80 AV: 2 NL: 6.90E6
T: + c Full ms [50.00-650.00]
Relative Abundance
m/z
93.05
69.08
77.05
91.04
53.08
80.10
94.10
55.10
81.11
107.08
95.09
108.09
121.05
122.10
136.09
137.14
161.24
177.16
190.96

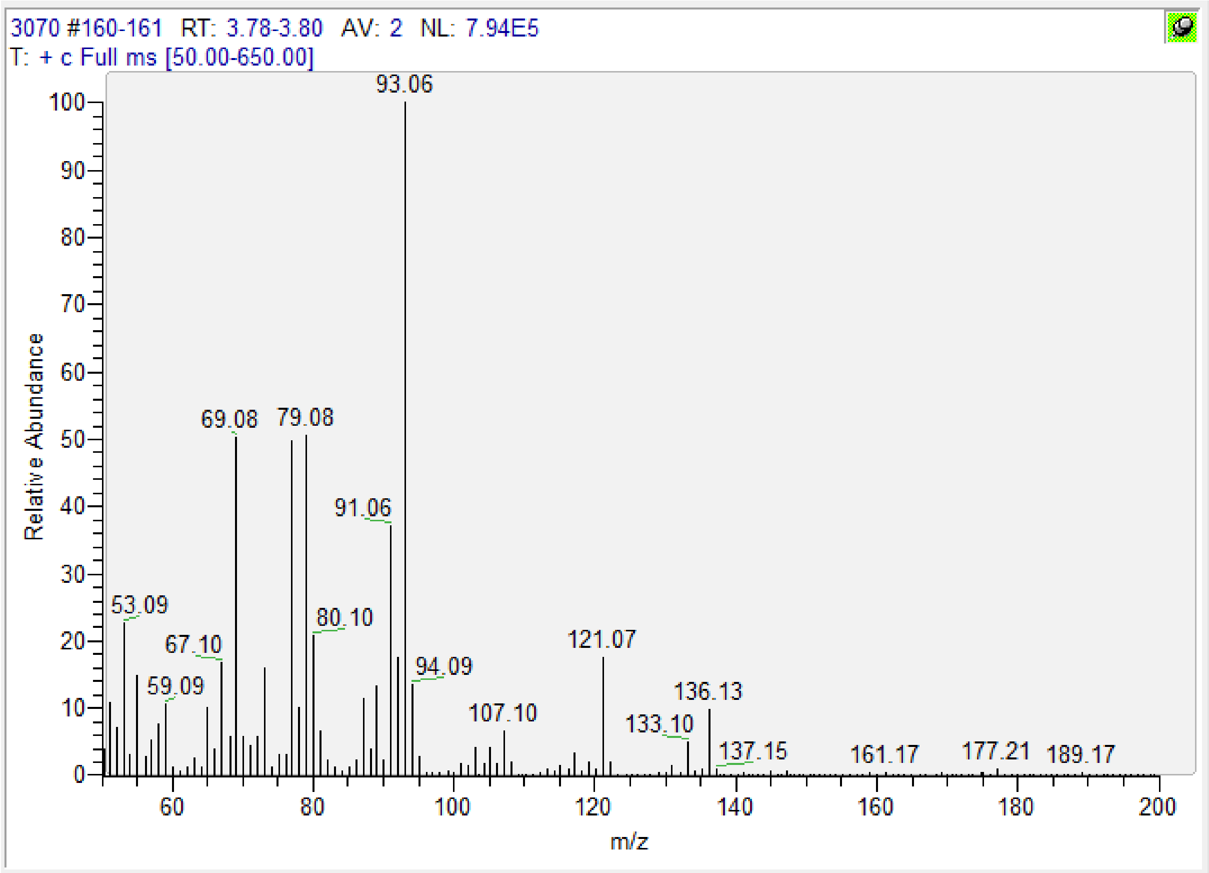

3070 #160-161 RT: 3.78-3.80 AV: 2 NL: 7.94E5
T: + c Full ms [50.00-650.00]
93.06
69.08
79.08
91.06
53.09
80.10
67.10
121.07
59.09
94.09
136.13
107.10
133.10
137.15
161.17
177.21
189.17
Relative Abundance
m/z

Alpha Terpinene

Reference:

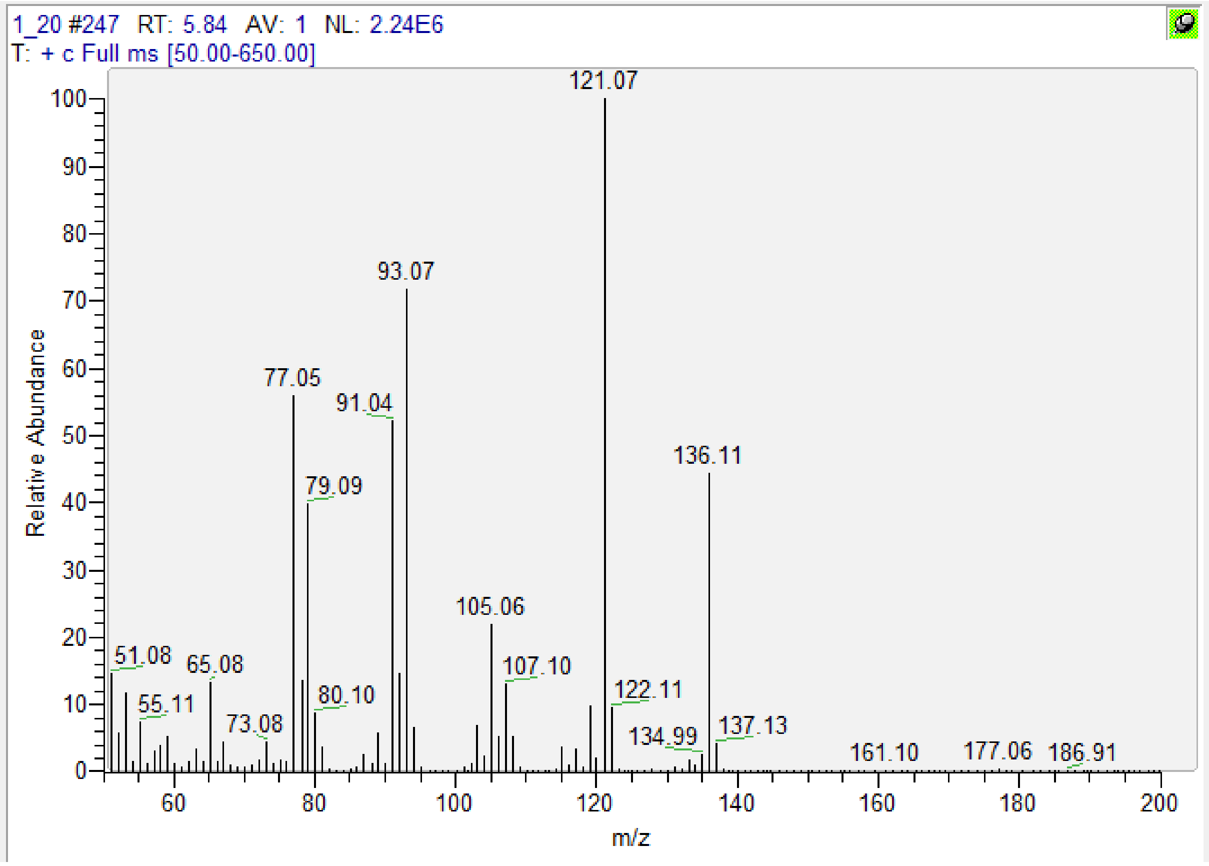

Samples:

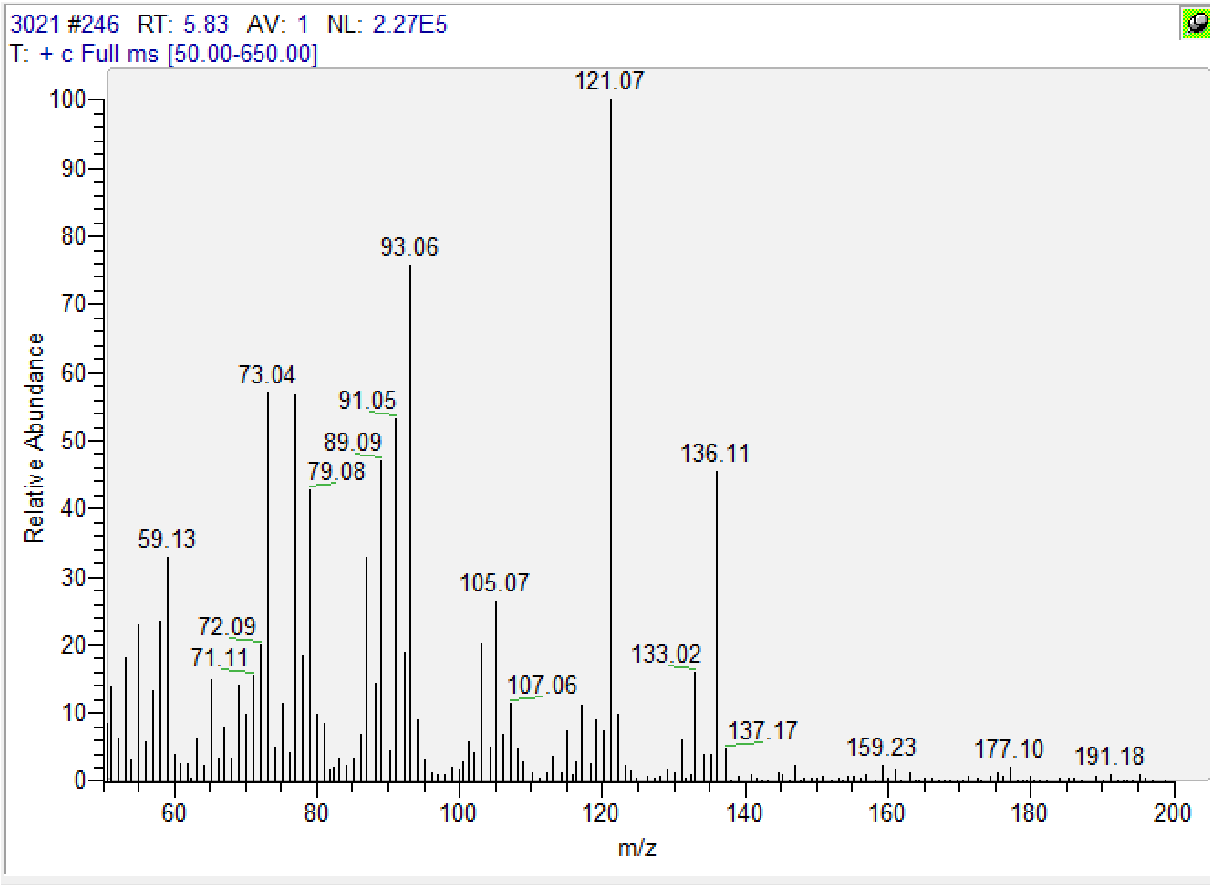
3021 #246 RT: 5.83 AV: 1 NL: 2.27E5
T: + c Full ms [50.00-650.00]
121.07
93.06
73.04
91.05
89.09
79.08
136.11
59.13
105.07
72.09
71.11
133.02
107.06
137.17
159.23
177.10
191.18
Relative Abundance
m/z

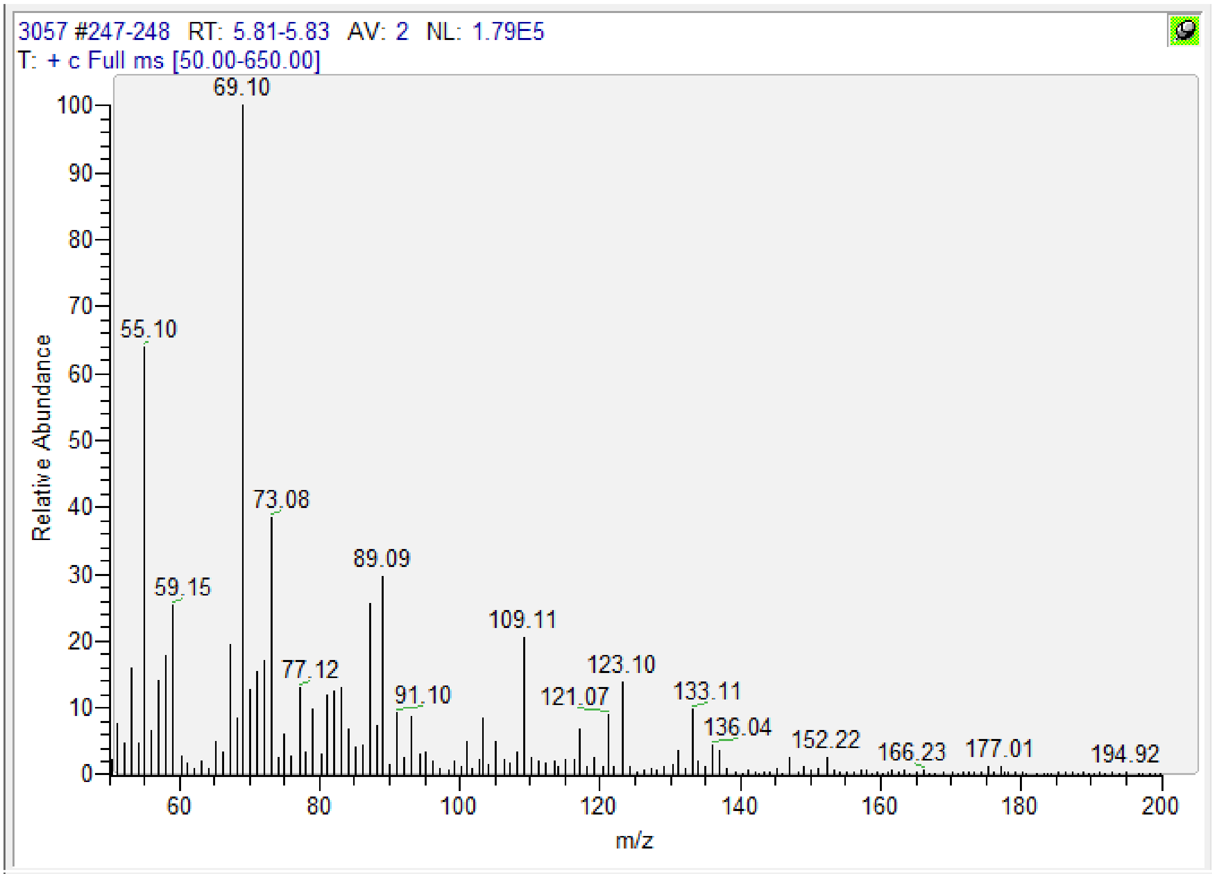

3057 #247-248 RT: 5.81-5.83 AV: 2 NL: 1.79E5
T: + c Full ms [50.00-650.00]
Relative Abundance
m/z
69.10
55.10
73.08
59.15
89.09
109.11
77.12
91.10
121.07
123.10
133.11
136.04
152.22
166.23
177.01
194.92

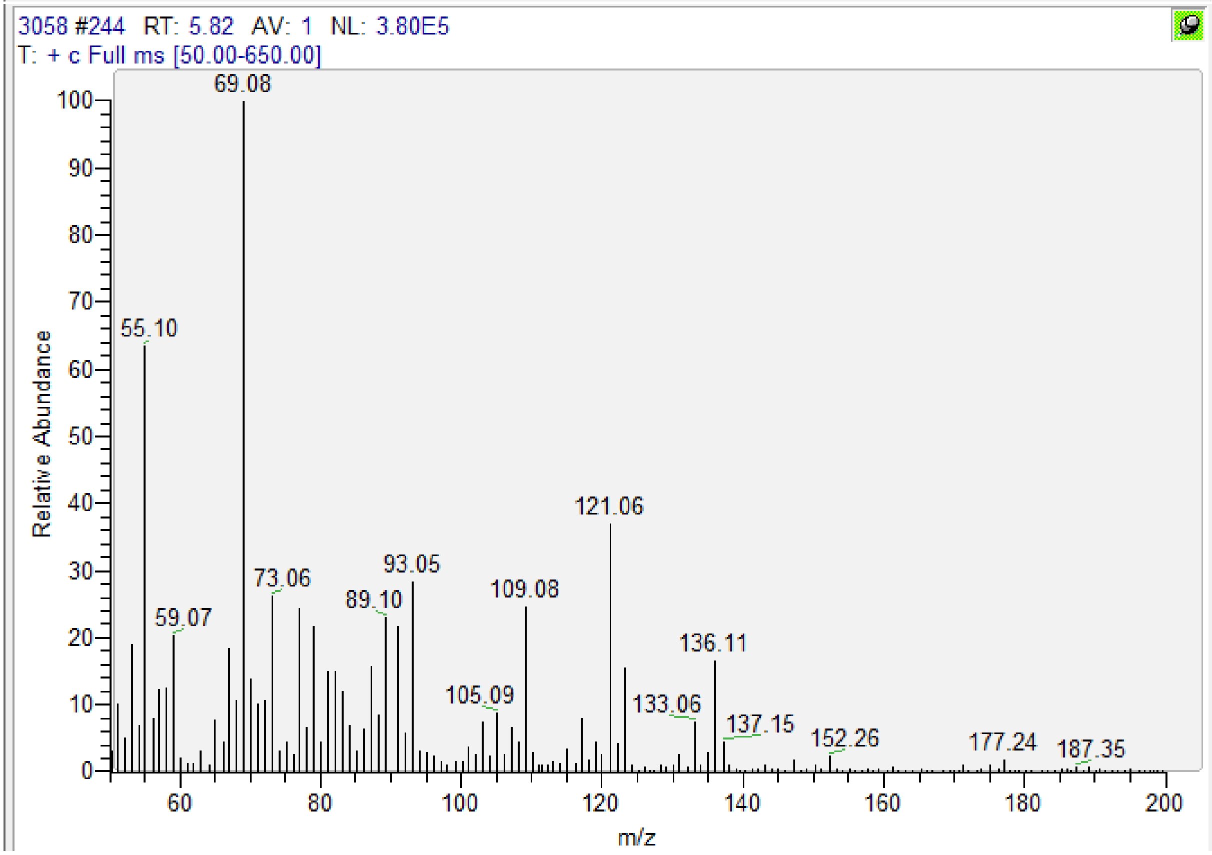

3058 #244 RT: 5.82 AV: 1 NL: 3.80E5
T: + c Full ms [50.00-650.00]
Relative Abundance
m/z
69.08
55.10
121.06
93.05
73.06
59.07
89.10
109.08
136.11
105.09
133.06
137.15
152.26
177.24
187.35

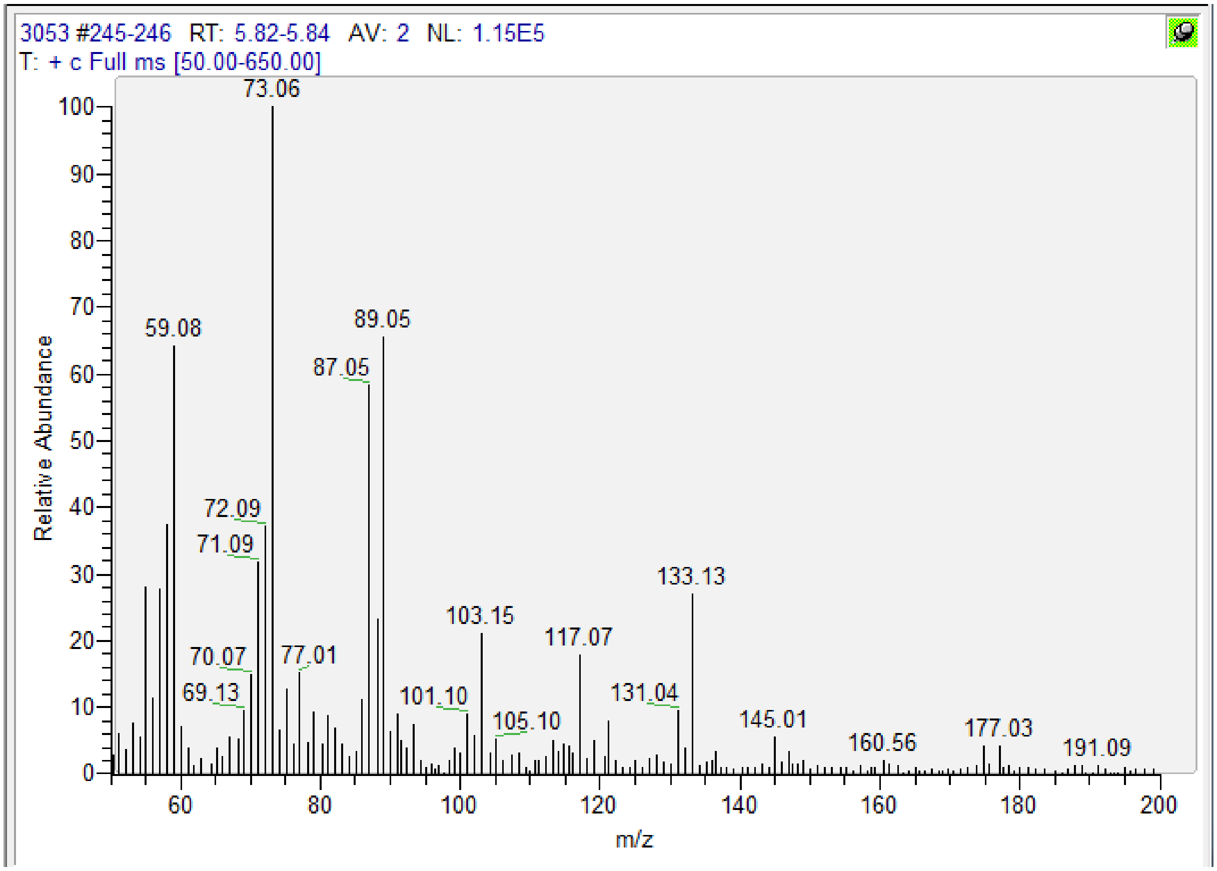

3053 #245-246 RT: 5.82-5.84 AV: 2 NL: 1.15E5
T: + c Full ms [50.00-650.00]
Relative Abundance
m/z
73.06
59.08
89.05
87.05
72.09
71.09
70.07
69.13
77.01
103.15
101.10
105.10
117.07
133.13
131.04
145.01
160.56
177.03
191.09

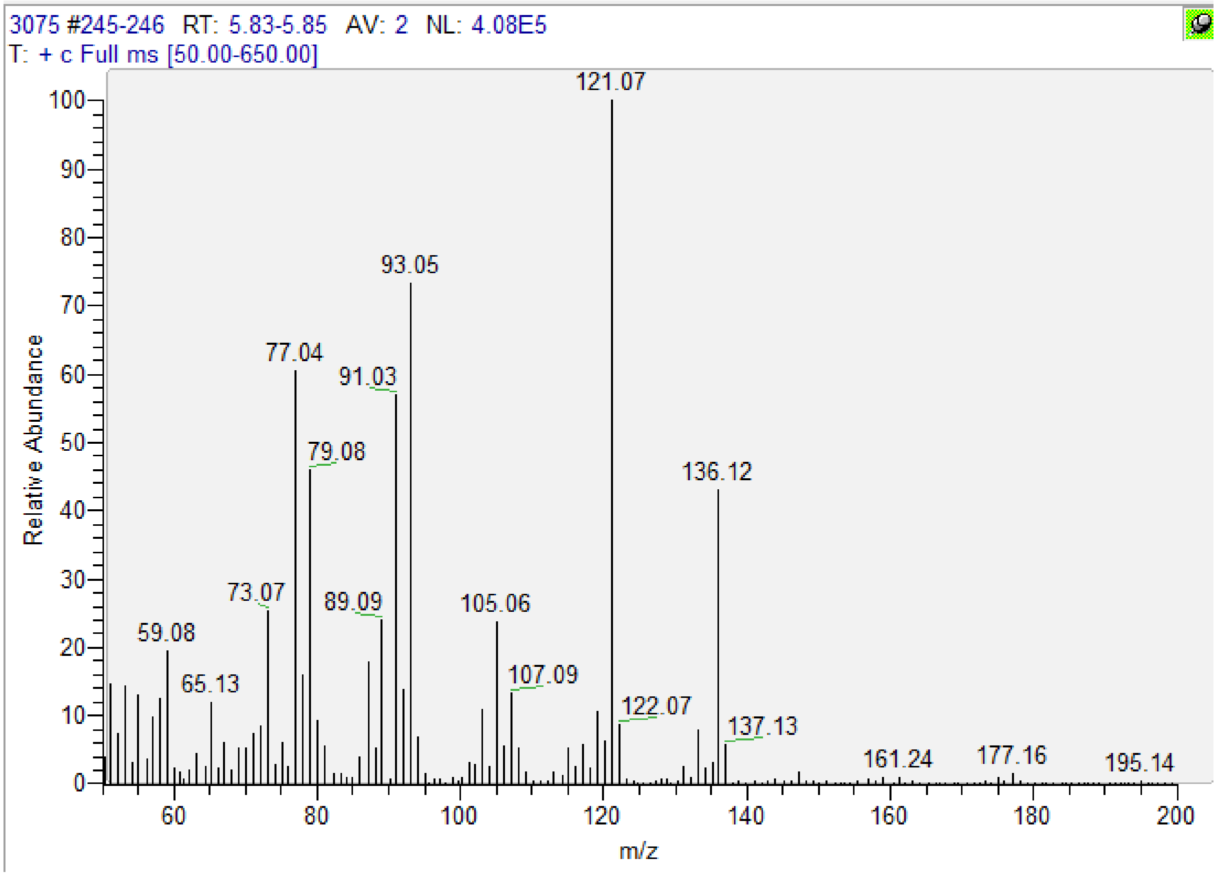
3075 #245-246 RT: 5.83-5.85 AV: 2 NL: 4.08E5
T: + c Full ms [50.00-650.00]
Relative Abundance
m/z
121.07
93.05
77.04
91.03
79.08
136.12
73.07
89.09
105.06
59.08
65.13
107.09
122.07
137.13
161.24
177.16
195.14

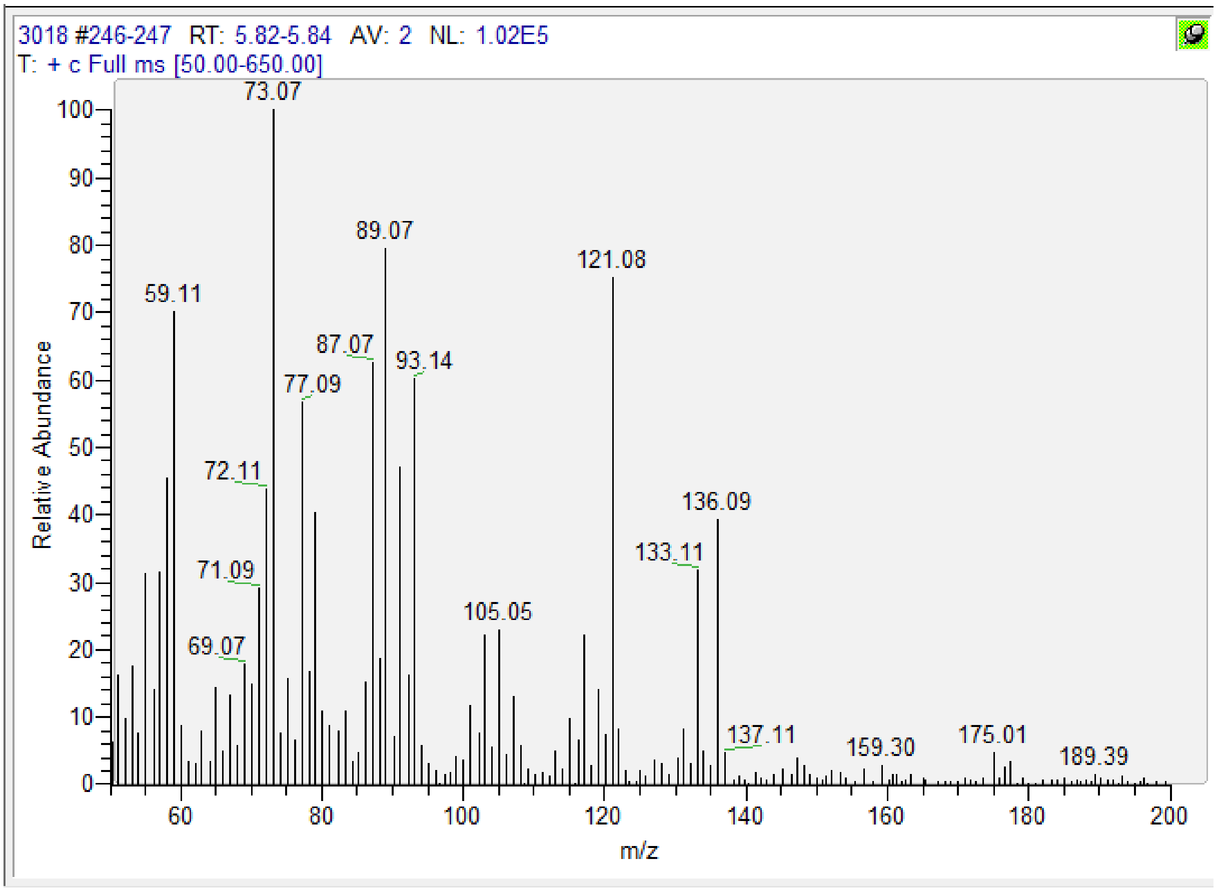

3018 #246-247 RT: 5.82-5.84 AV: 2 NL: 1.02E5
T: + c Full ms [50.00-650.00]
Relative Abundance
m/z
73.07
89.07
121.08
59.11
87.07
93.14
77.09
72.11
136.09
133.11
71.09
105.05
69.07
137.11
159.30
175.01
189.39

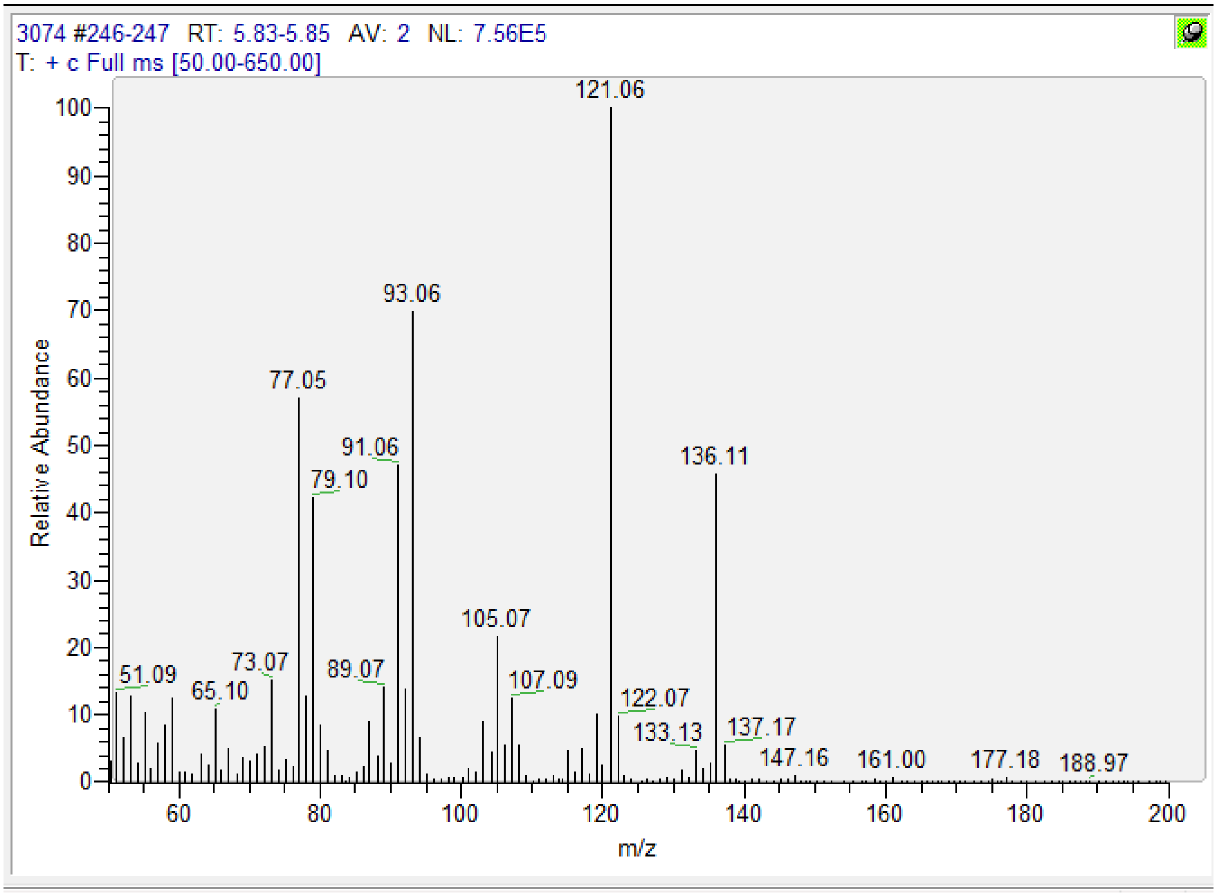
3074 #246-247 RT: 5.83-5.85 AV: 2 NL: 7.56E5
T: + c Full ms [50.00-650.00]
Relative Abundance
m/z
121.06
93.06
77.05
91.06
79.10
136.11
105.07
51.09
65.10
73.07
89.07
107.09
122.07
133.13
137.17
147.16
161.00
177.18
188.97

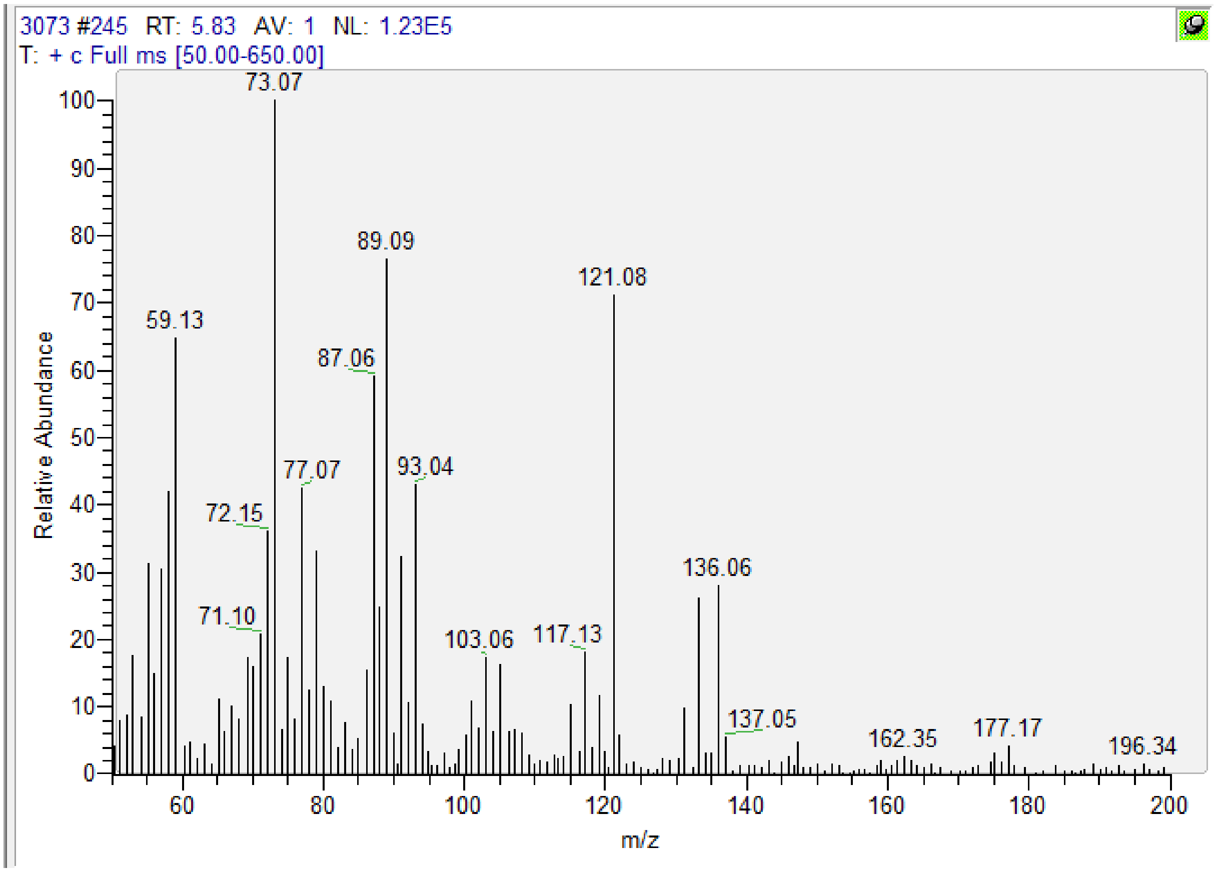
3073 #245 RT: 5.83 AV: 1 NL: 1.23E5
T: + c Full ms [50.00-650.00]
Relative Abundance
m/z
73.07
89.09
121.08
59.13
87.06
77.07
93.04
72.15
136.06
71.10
103.06
117.13
137.05
162.35
177.17
196.34

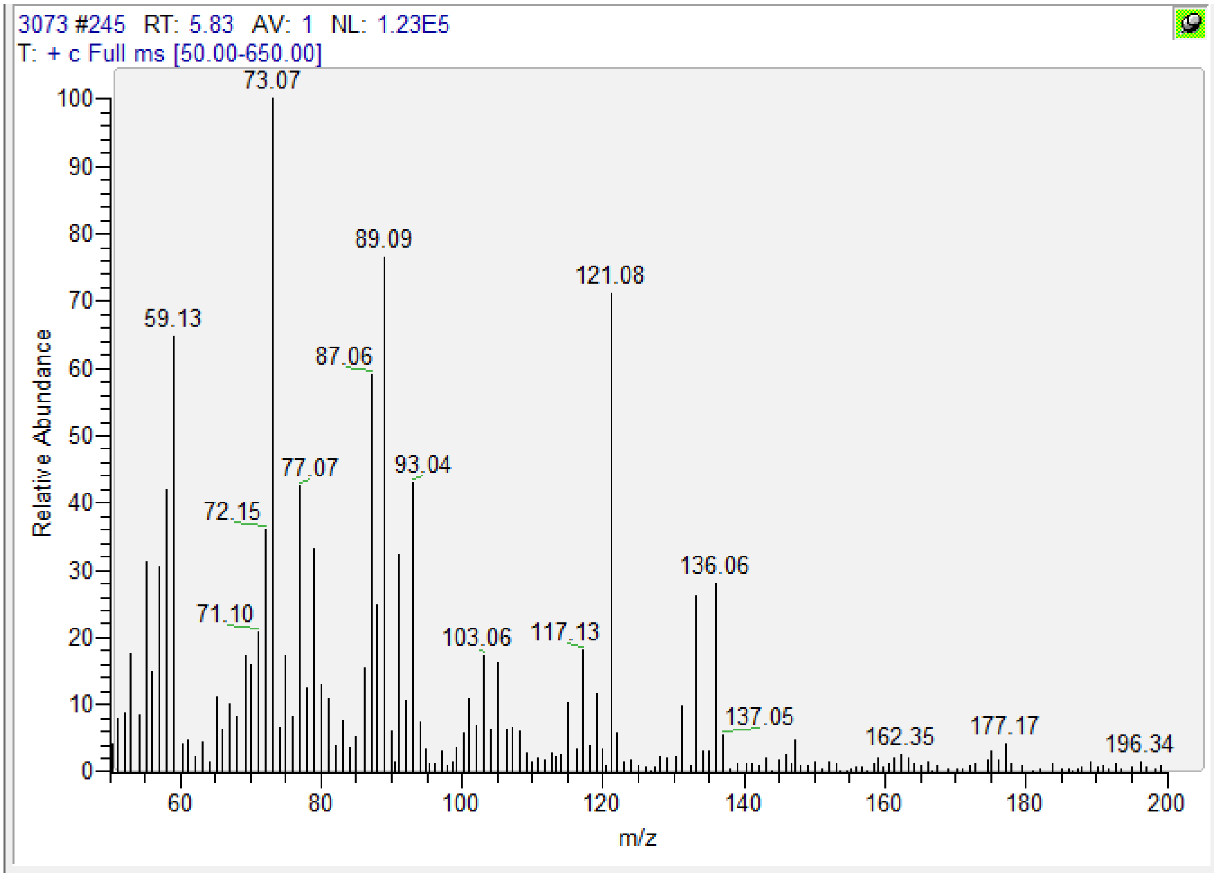

3073 #245 RT: 5.83 AV: 1 NL: 1.23E5
T: + c Full ms [50.00-650.00]
Relative Abundance
m/z
73.07
89.09
121.08
59.13
87.06
77.07
93.04
72.15
136.06
71.10
103.06
117.13
137.05
162.35
177.17
196.34

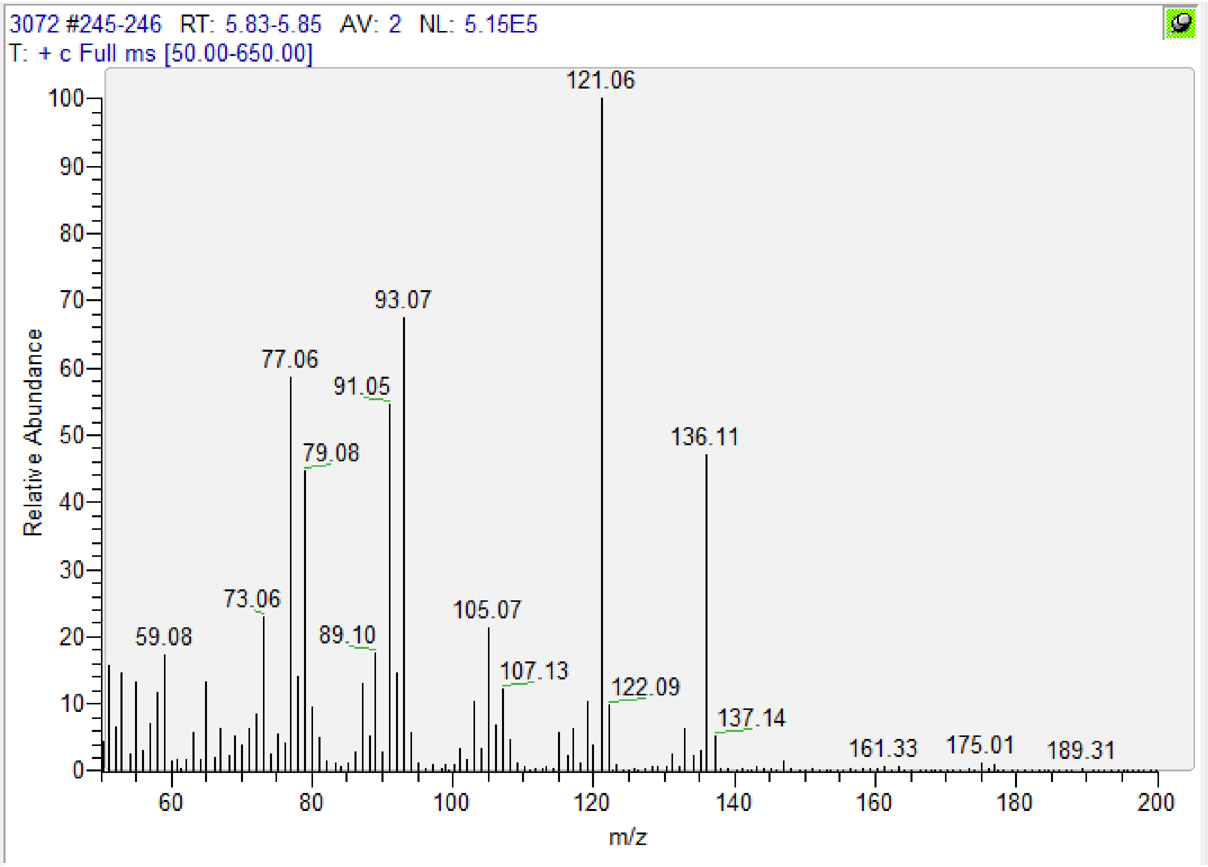

3072 #245-246 RT: 5.83-5.85 AV: 2 NL: 5.15E5
T: + c Full ms [50.00-650.00]
121.06
93.07
77.06
91.05
79.08
136.11
73.06
105.07
59.08
89.10
107.13
122.09
137.14
161.33
175.01
189.31
Relative Abundance
m/z

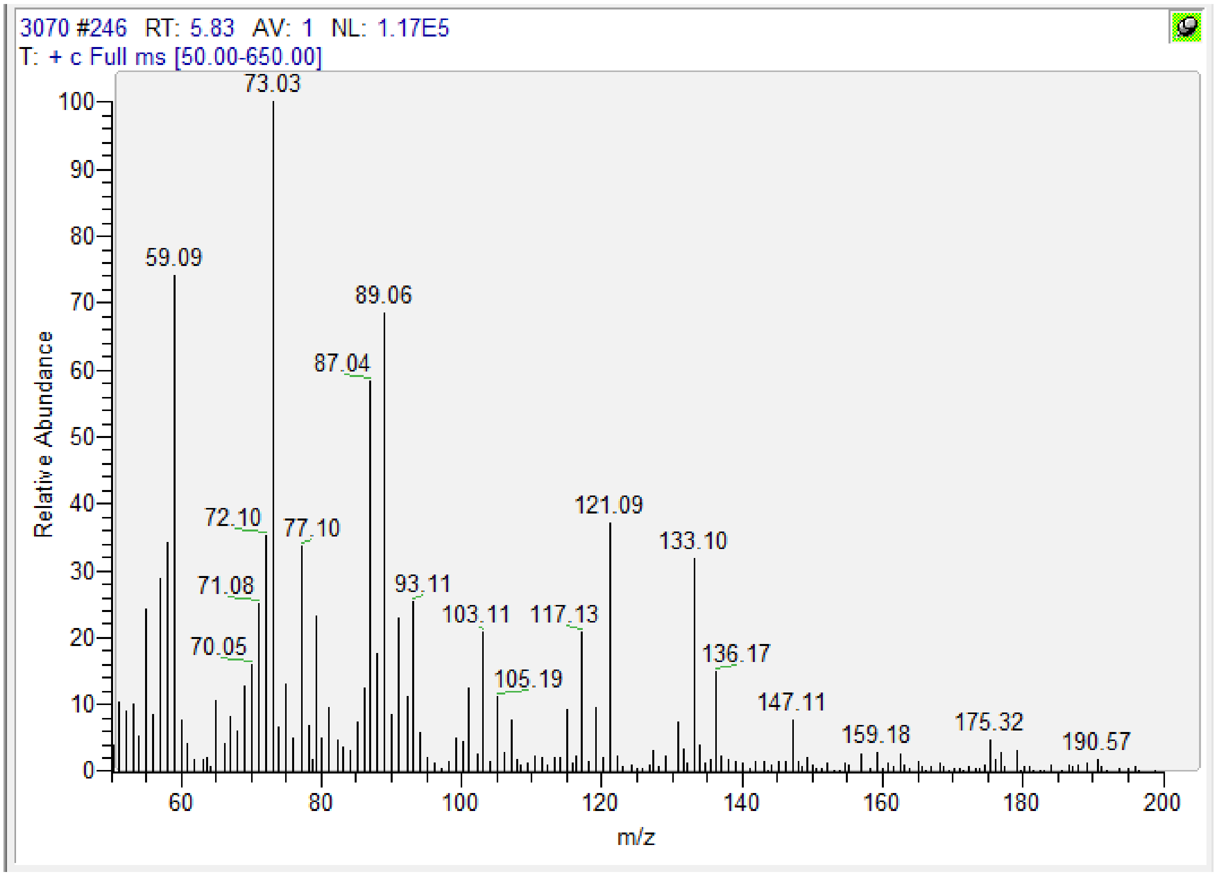

3070 #246 RT: 5.83 AV: 1 NL: 1.17E5
T: + c Full ms [50.00-650.00]
Relative Abundance
m/z
73.03
59.09
89.06
87.04
72.10
77.10
71.08
70.05
93.11
103.11
117.13
121.09
133.10
136.17
105.19
147.11
159.18
175.32
190.57

Limonene

Reference:

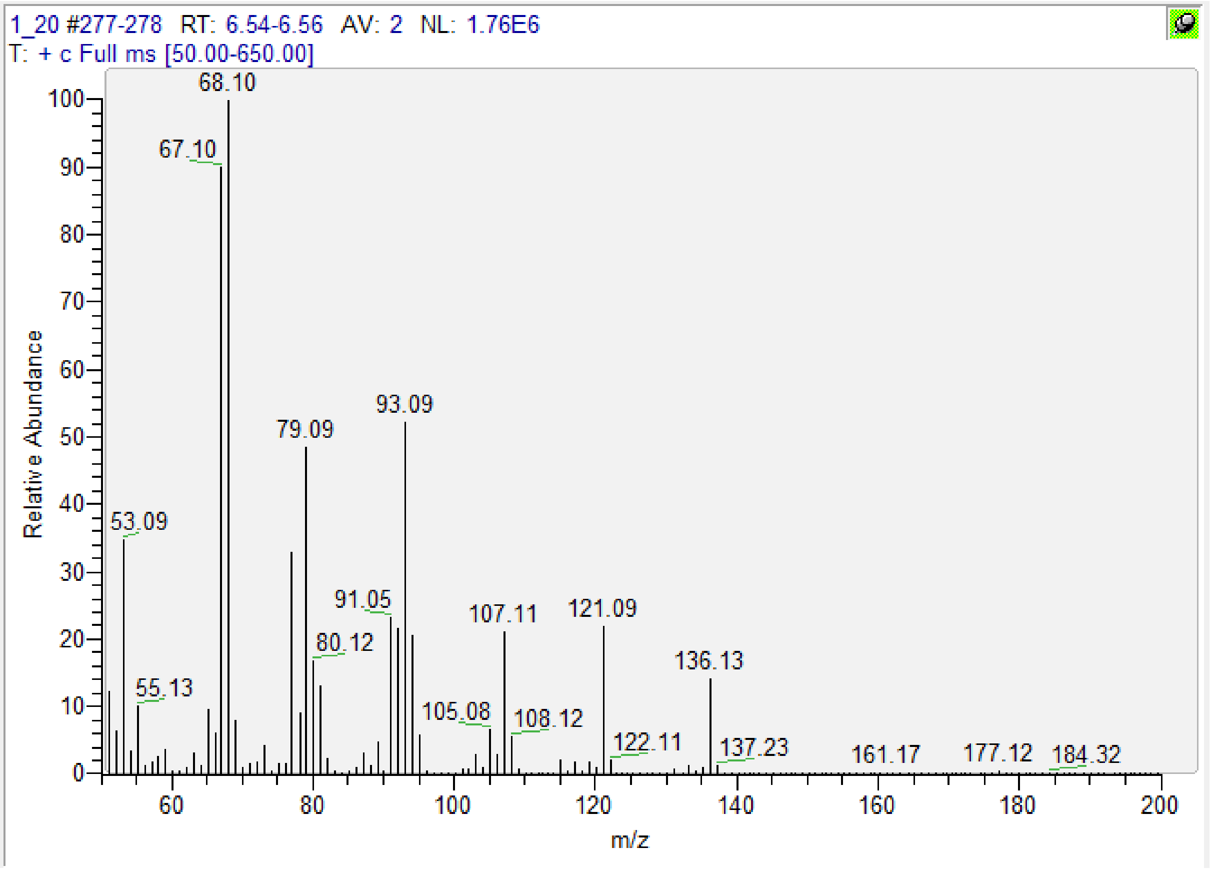

Samples:

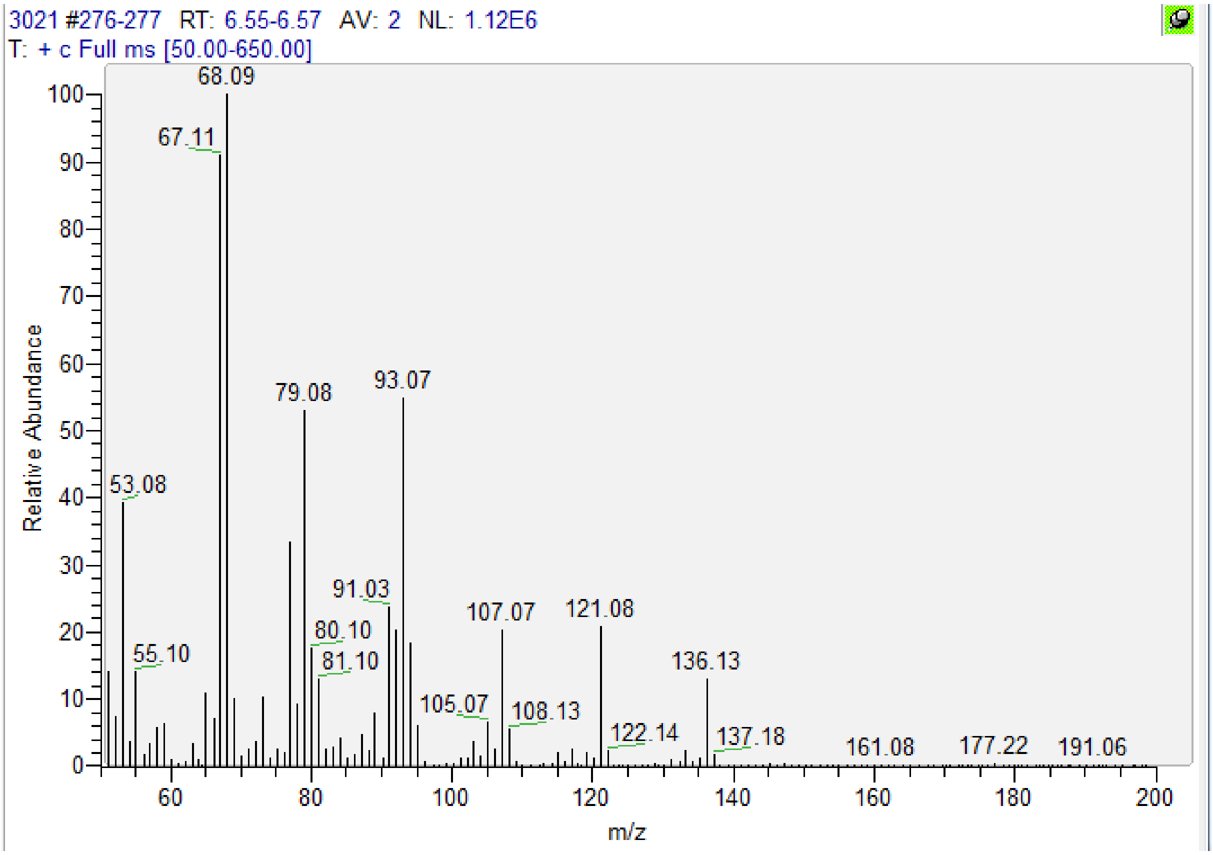

3021 #276-277 RT: 6.55-6.57 AV: 2 NL: 1.12E6
T: + c Full ms [50.00-650.00]
Relative Abundance
m/z
68.09
67.11
79.08
93.07
53.08
91.03
80.10
81.10
55.10
107.07
121.08
136.13
105.07
108.13
122.14
137.18
161.08
177.22
191.06

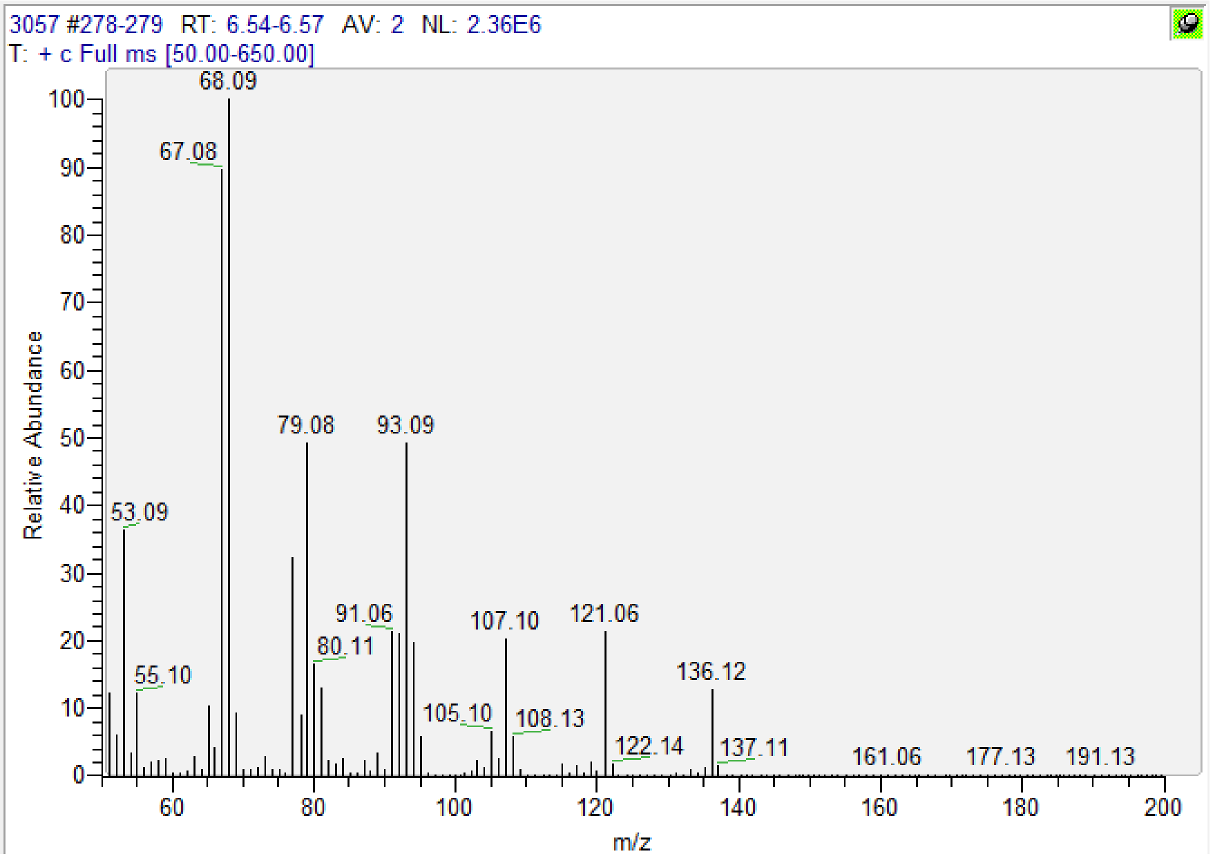
3057 #278-279 RT: 6.54-6.57 AV: 2 NL: 2.36E6
T: + c Full ms [50.00-650.00]
Relative Abundance
m/z
68.09
67.08
79.08
93.09
53.09
91.06
80.11
55.10
107.10
121.06
136.12
105.10
108.13
122.14
137.11
161.06
177.13
191.13

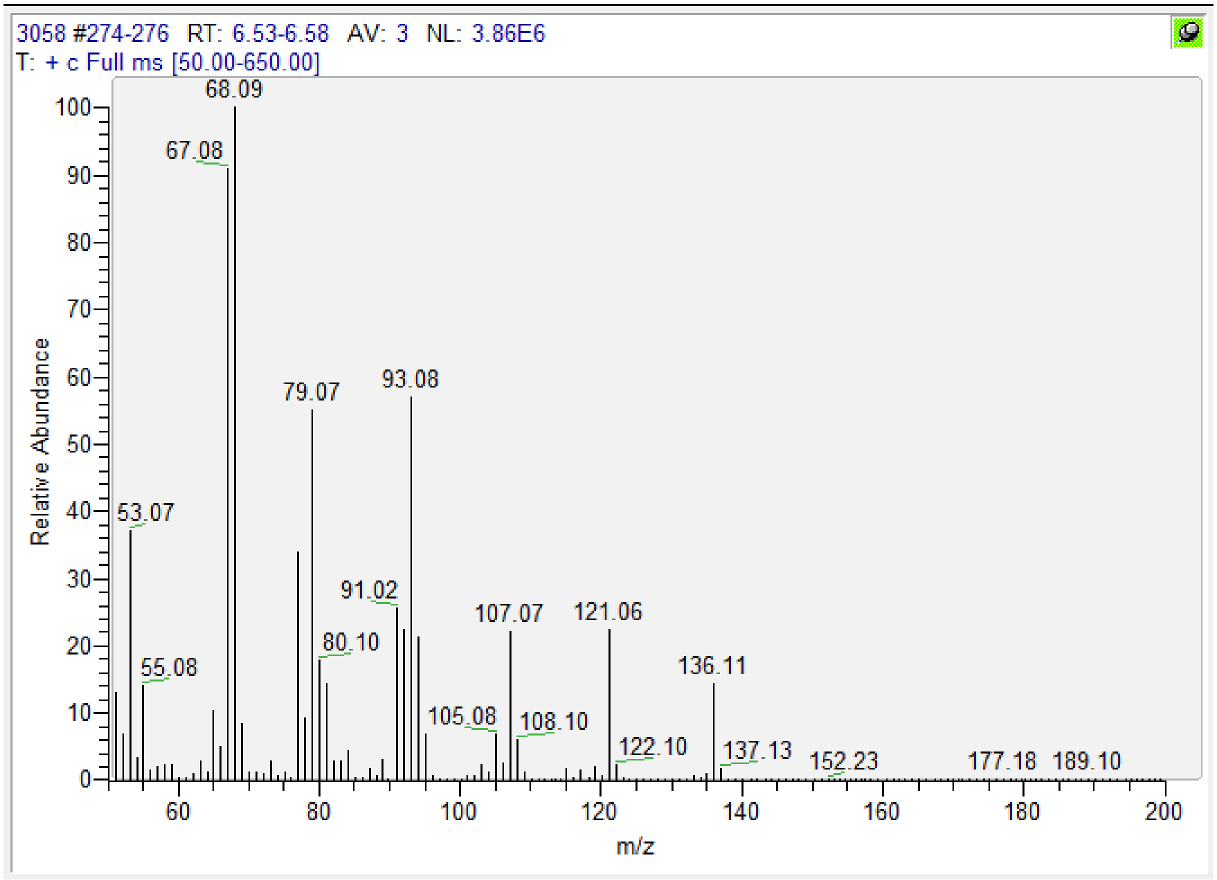

3058 #274-276 RT: 6.53-6.58 AV: 3 NL: 3.86E6
T: + c Full ms [50.00-650.00]
Relative Abundance
m/z
68.09
67.08
79.07
93.08
53.07
91.02
80.10
55.08
107.07
121.06
136.11
105.08
108.10
122.10
137.13
152.23
177.18
189.10

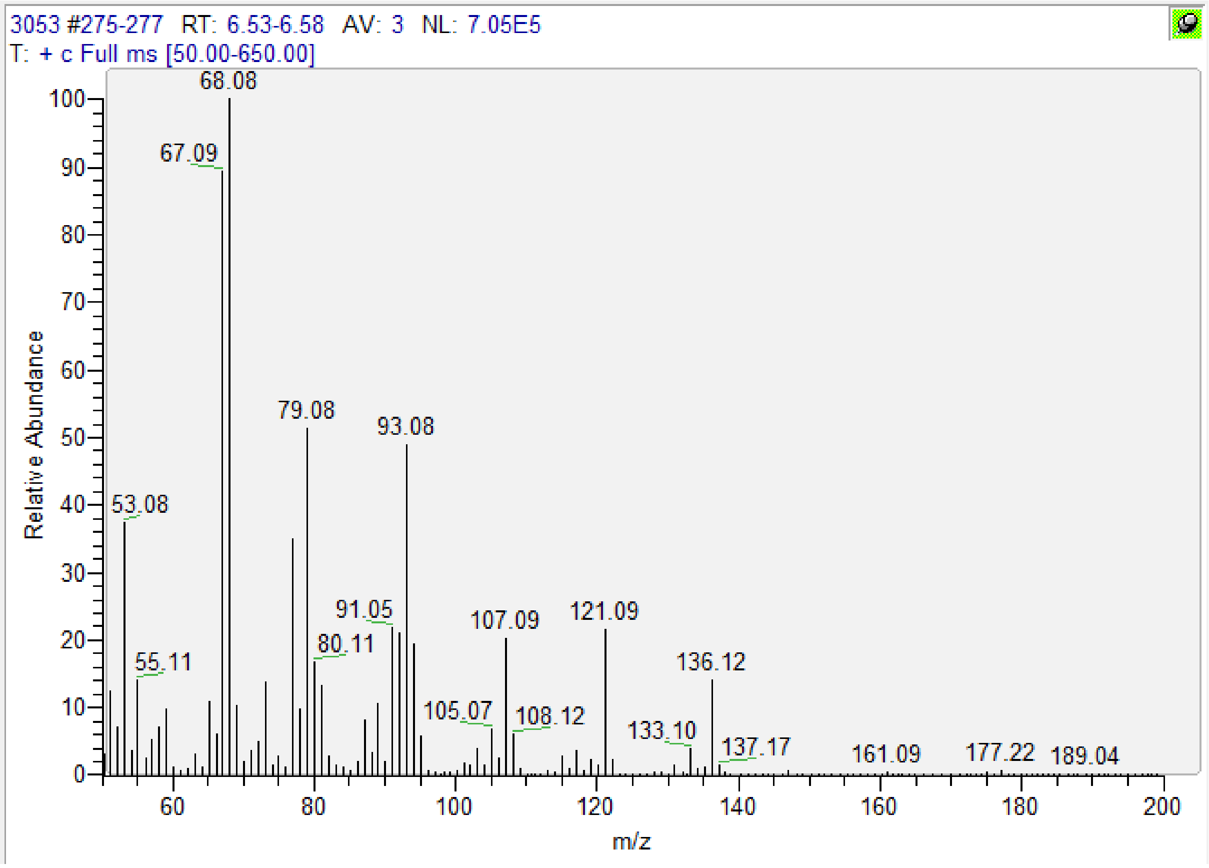
3053 #275-277 RT: 6.53-6.58 AV: 3 NL: 7.05E5
T: + c Full ms [50.00-650.00]
Relative Abundance
m/z
68.08
67.09
79.08
93.08
53.08
91.05
107.09
121.09
80.11
55.11
136.12
105.07
108.12
133.10
137.17
161.09
177.22
189.04

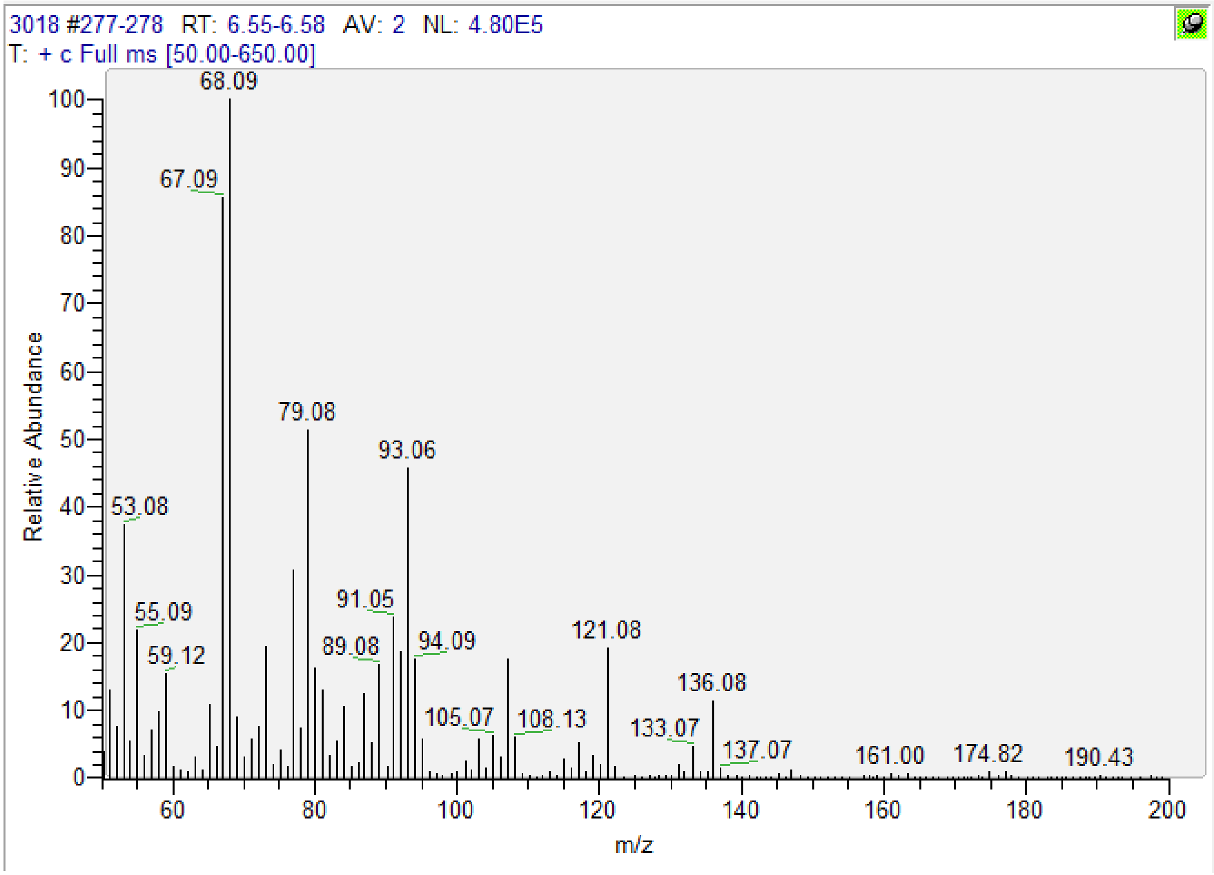
3018 #277-278 RT: 6.55-6.58 AV: 2 NL: 4.80E5
T: + c Full ms [50.00-650.00]
Relative Abundance
m/z
68.09
67.09
79.08
93.06
53.08
55.09
59.12
91.05
89.08
94.09
121.08
136.08
105.07
108.13
133.07
137.07
161.00
174.82
190.43

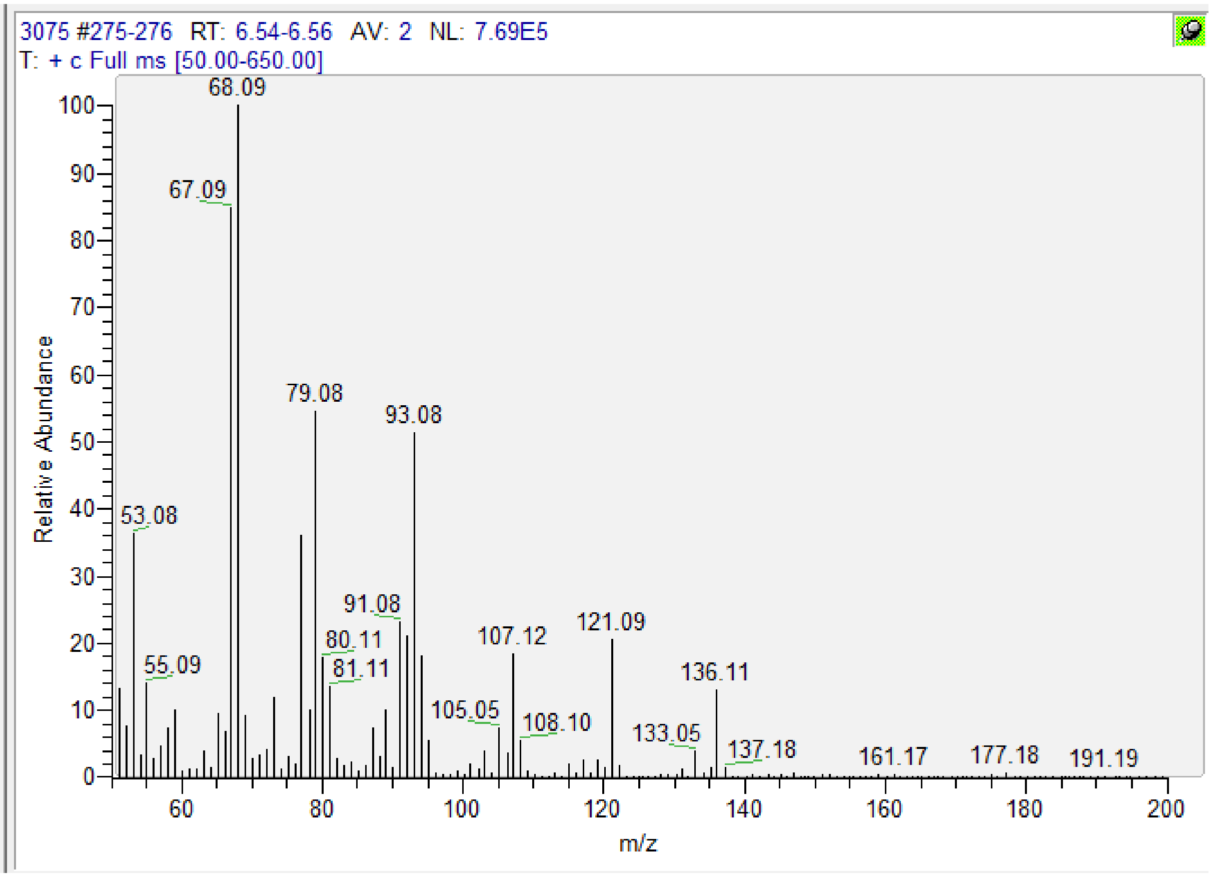
3075 #275-276 RT: 6.54-6.56 AV: 2 NL: 7.69E5
T: + c Full ms [50.00-650.00]
Relative Abundance
m/z
68.09
67.09
79.08
93.08
53.08
91.08
80.11
81.11
55.09
107.12
121.09
136.11
105.05
108.10
133.05
137.18
161.17
177.18
191.19

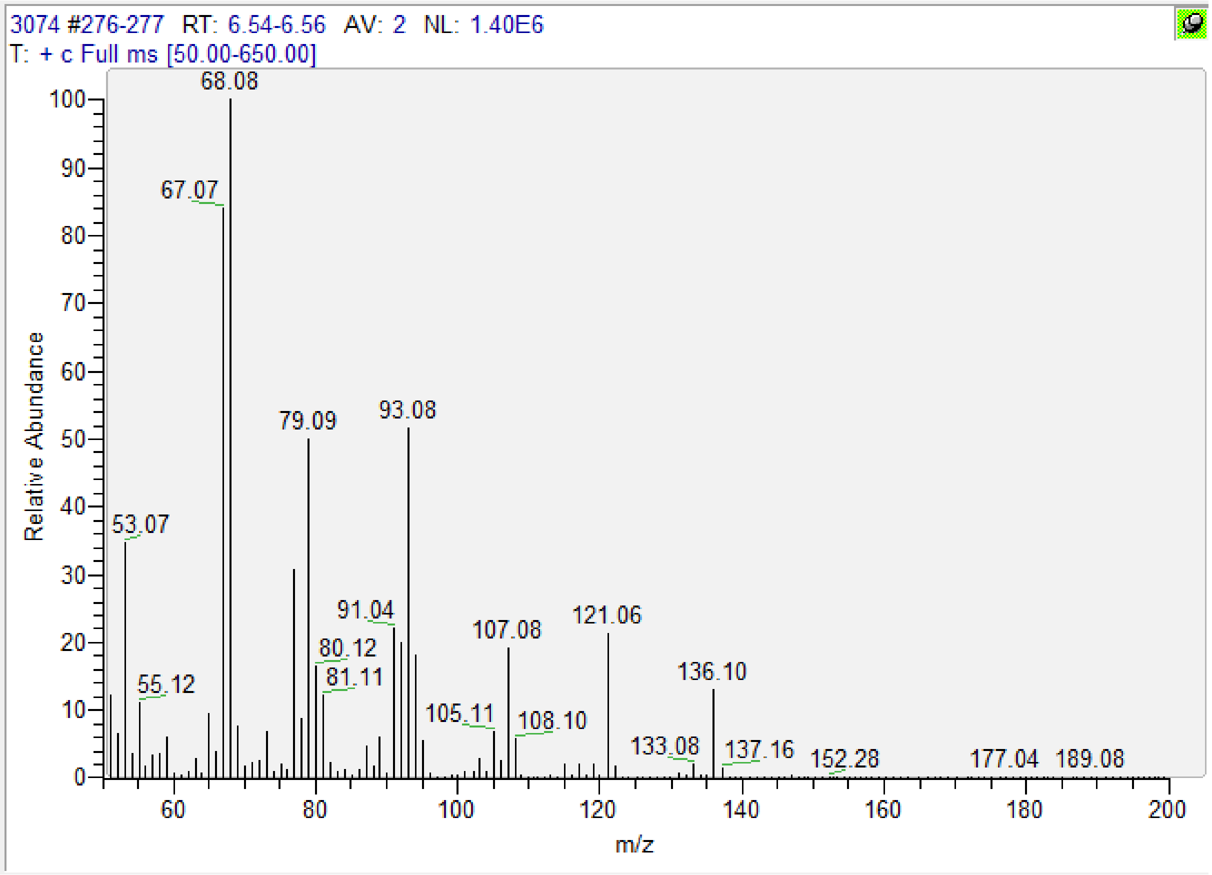

3074 #276-277 RT: 6.54-6.56 AV: 2 NL: 1.40E6
T: + c Full ms [50.00-650.00]
Relative Abundance
m/z
68.08
67.07
79.09
93.08
53.07
91.04
80.12
81.11
55.12
107.08
121.06
136.10
105.11
108.10
133.08
137.16
152.28
177.04
189.08

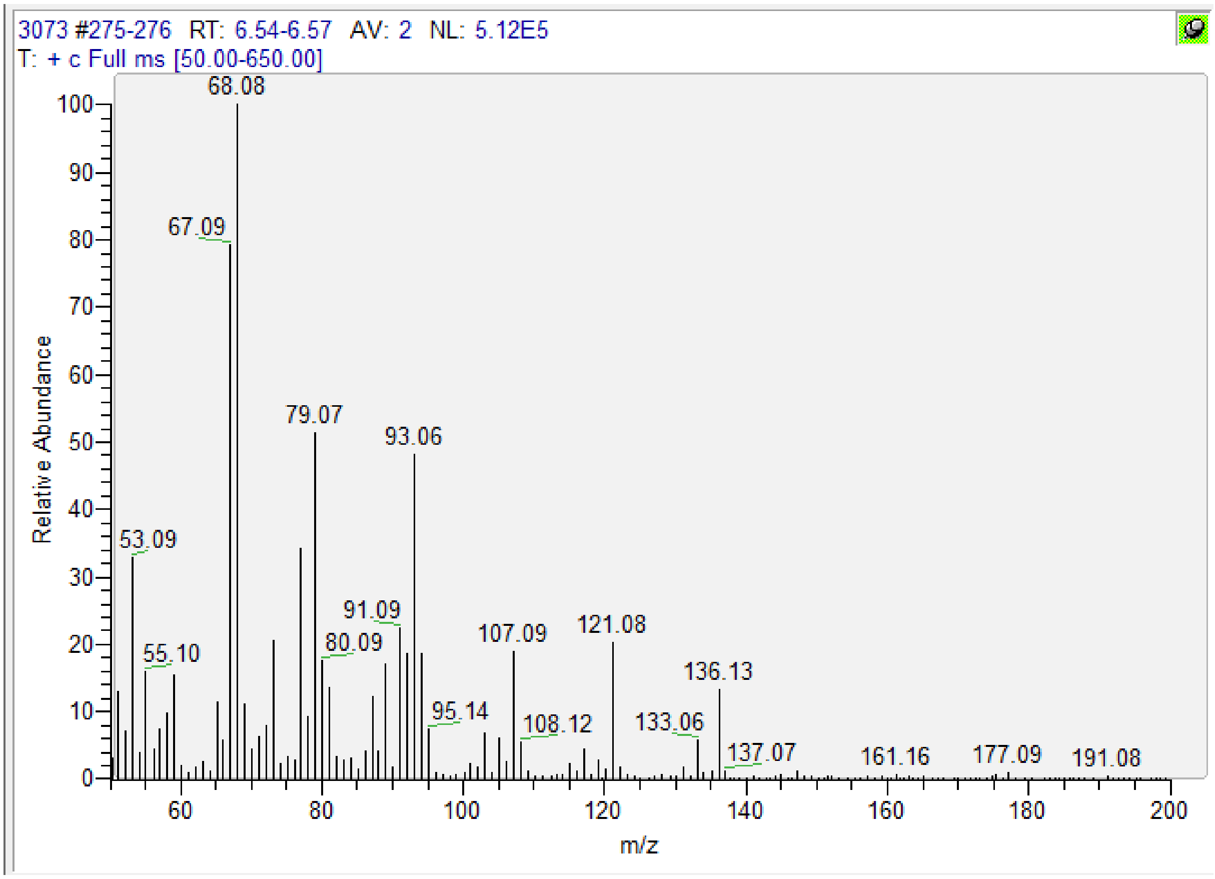
3073 #275-276 RT: 6.54-6.57 AV: 2 NL: 5.12E5
T: + c Full ms [50.00-650.00]
Relative Abundance
m/z
68.08
67.09
79.07
93.06
53.09
91.09
55.10
80.09
107.09
121.08
136.13
95.14
108.12
133.06
137.07
161.16
177.09
191.08

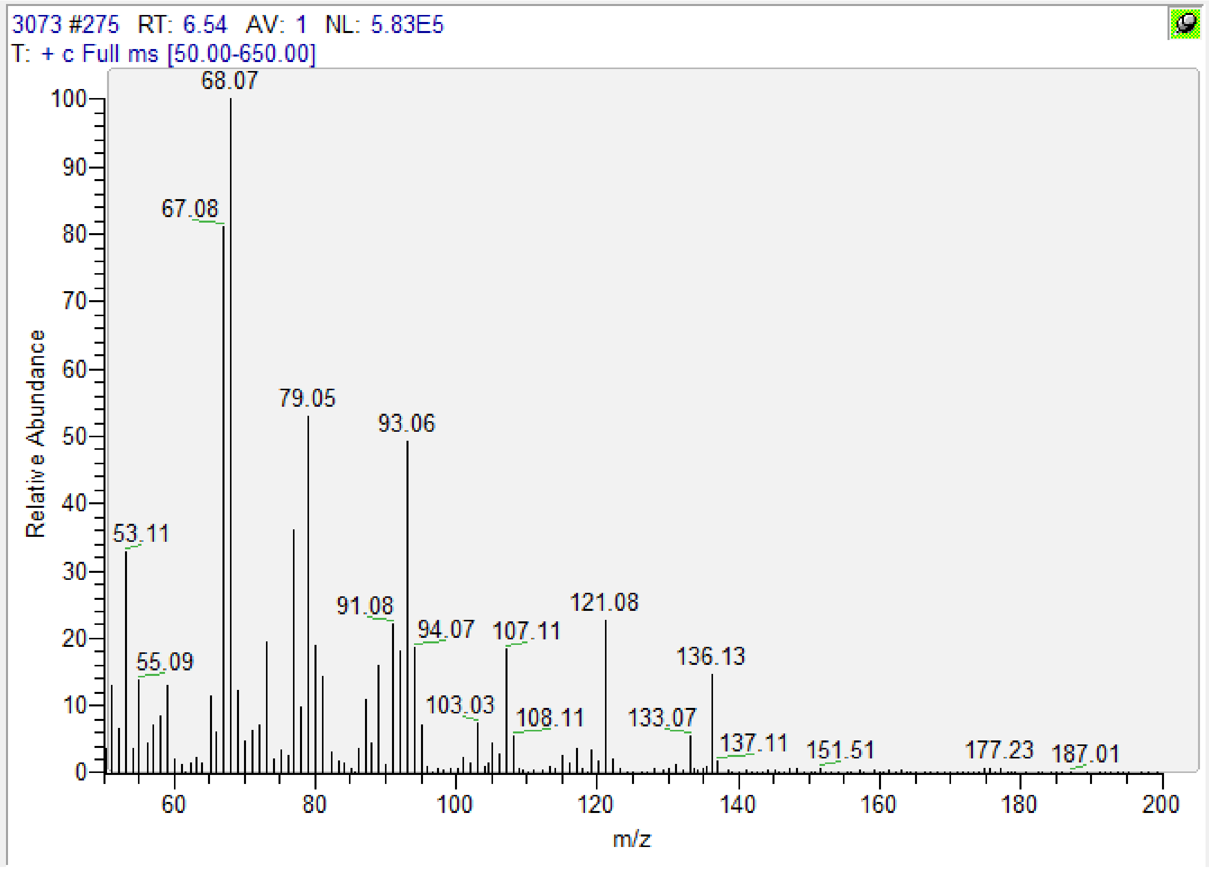
3073 #275 RT: 6.54 AV: 1 NL: 5.83E5
T: + c Full ms [50.00-650.00]
Relative Abundance
m/z
68.07
67.08
79.05
93.06
53.11
55.09
91.08
94.07
107.11
121.08
136.13
103.03
108.11
133.07
137.11
151.51
177.23
187.01

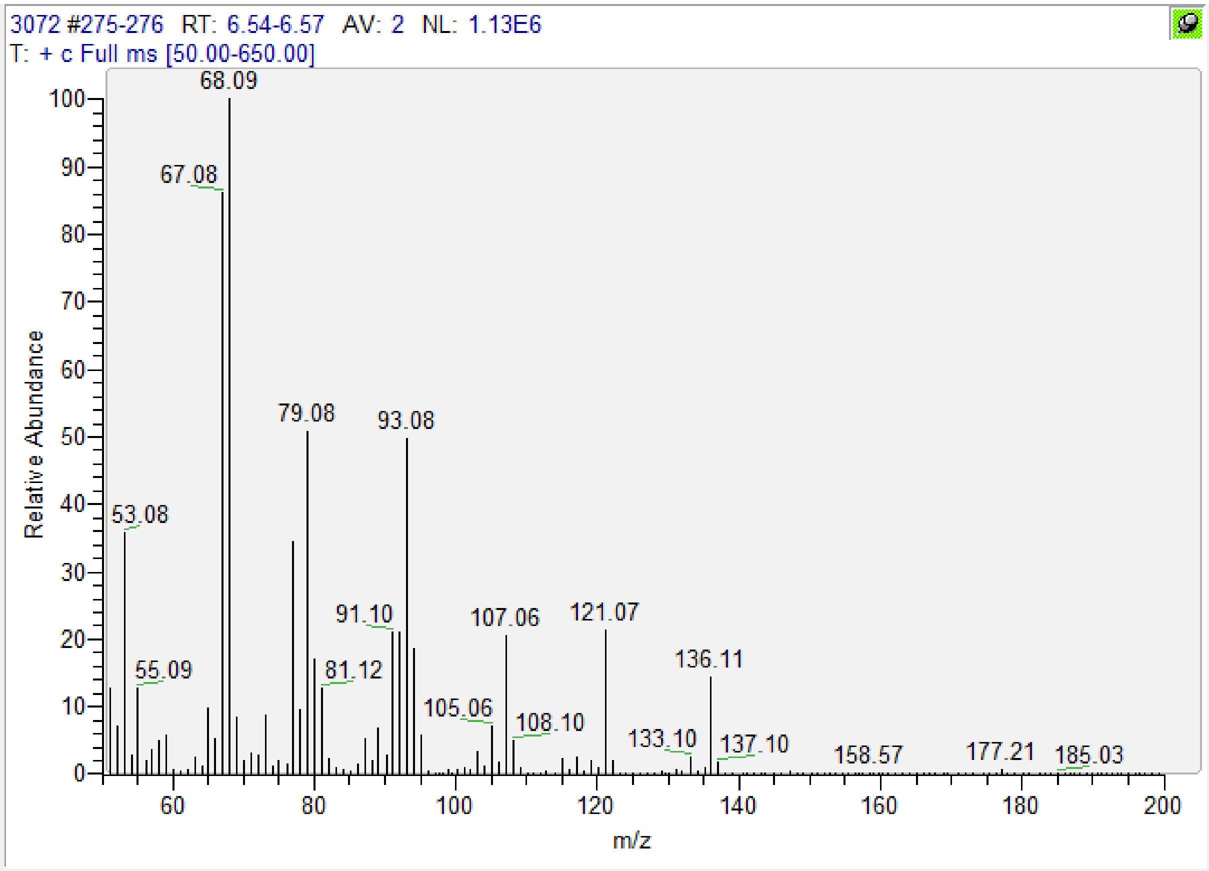
3072 #275-276 RT: 6.54-6.57 AV: 2 NL: 1.13E6
T: + c Full ms [50.00-650.00]
Relative Abundance
m/z
68.09
67.08
79.08
93.08
53.08
55.09
91.10
81.12
107.06
121.07
136.11
105.06
108.10
133.10
137.10
158.57
177.21
185.03

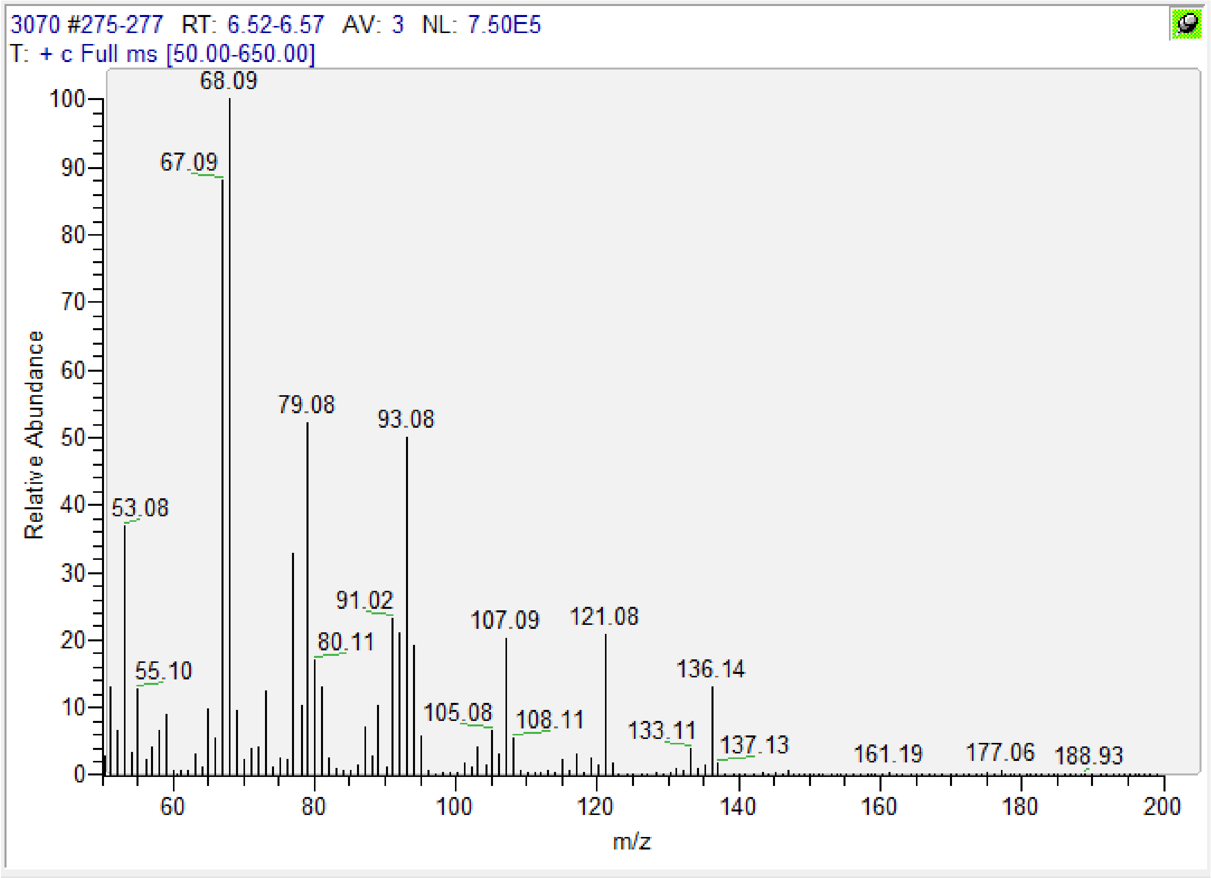

3070 #275-277 RT: 6.52-6.57 AV: 3 NL: 7.50E5
T: + c Full ms [50.00-650.00]
68.09
67.09
79.08
93.08
53.08
91.02
80.11
107.09
121.08
55.10
136.14
105.08
108.11
133.11
137.13
161.19
177.06
188.93
Relative Abundance
m/z

p-cymene

Reference:

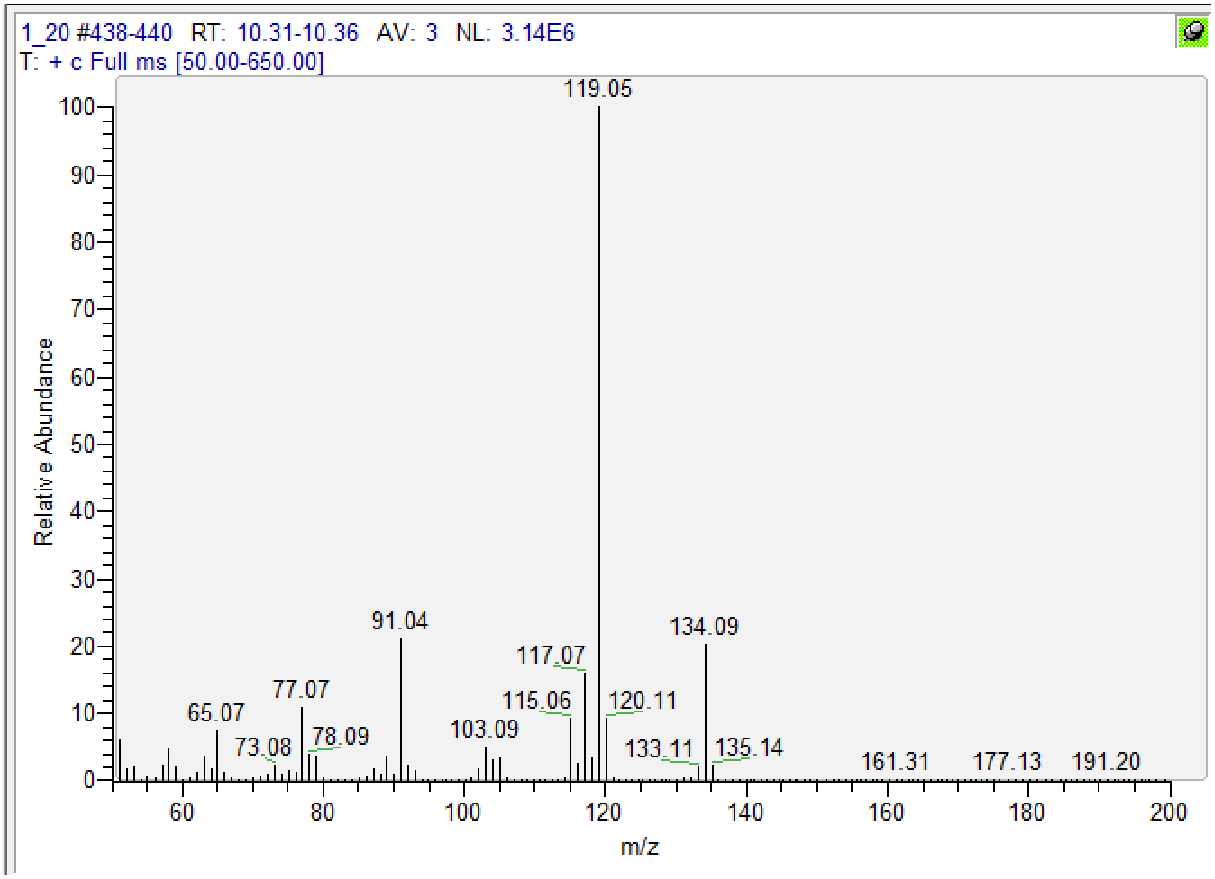

Samples

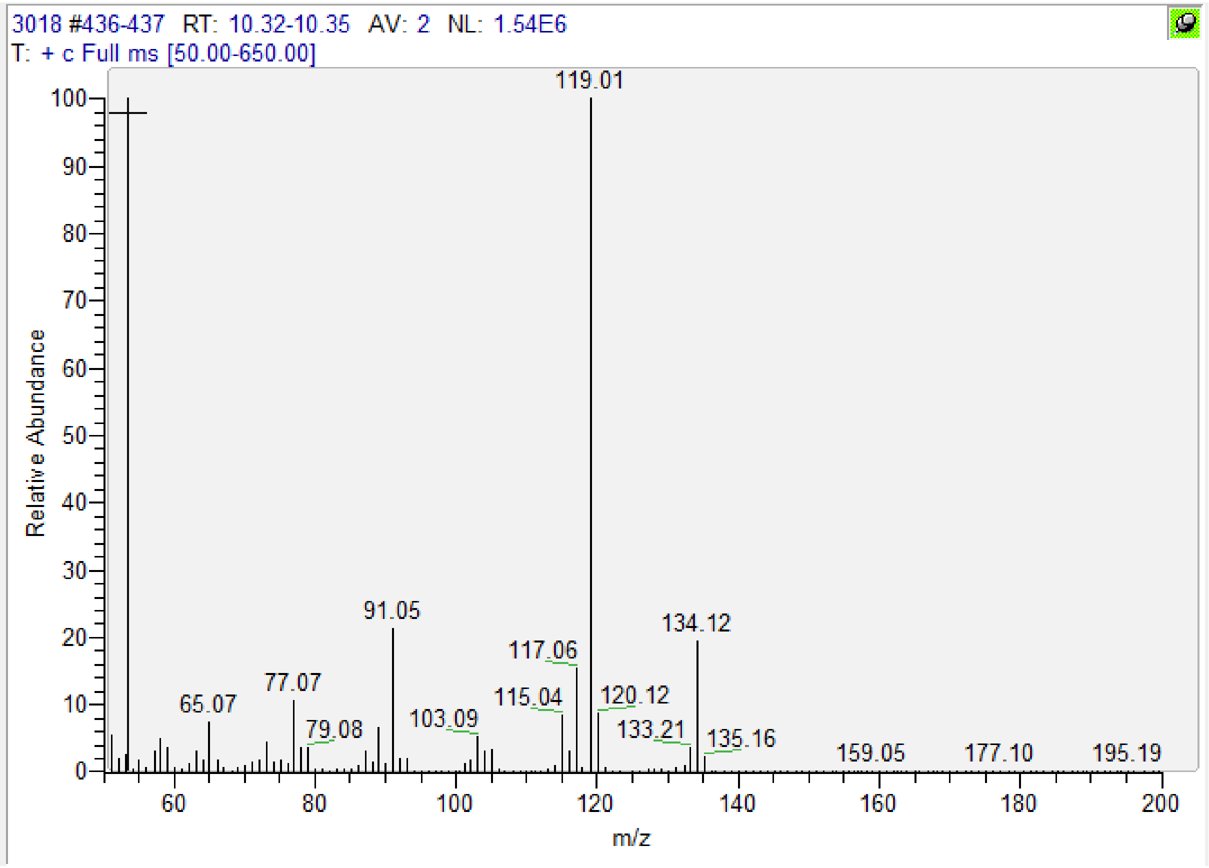

3018 #436-437 RT: 10.32-10.35 AV: 2 NL: 1.54E6
T: + c Full ms [50.00-650.00]
Relative Abundance
m/z
119.01
91.05
134.12
117.06
77.07
65.07
115.04
120.12
79.08
103.09
133.21
135.16
159.05
177.10
195.19

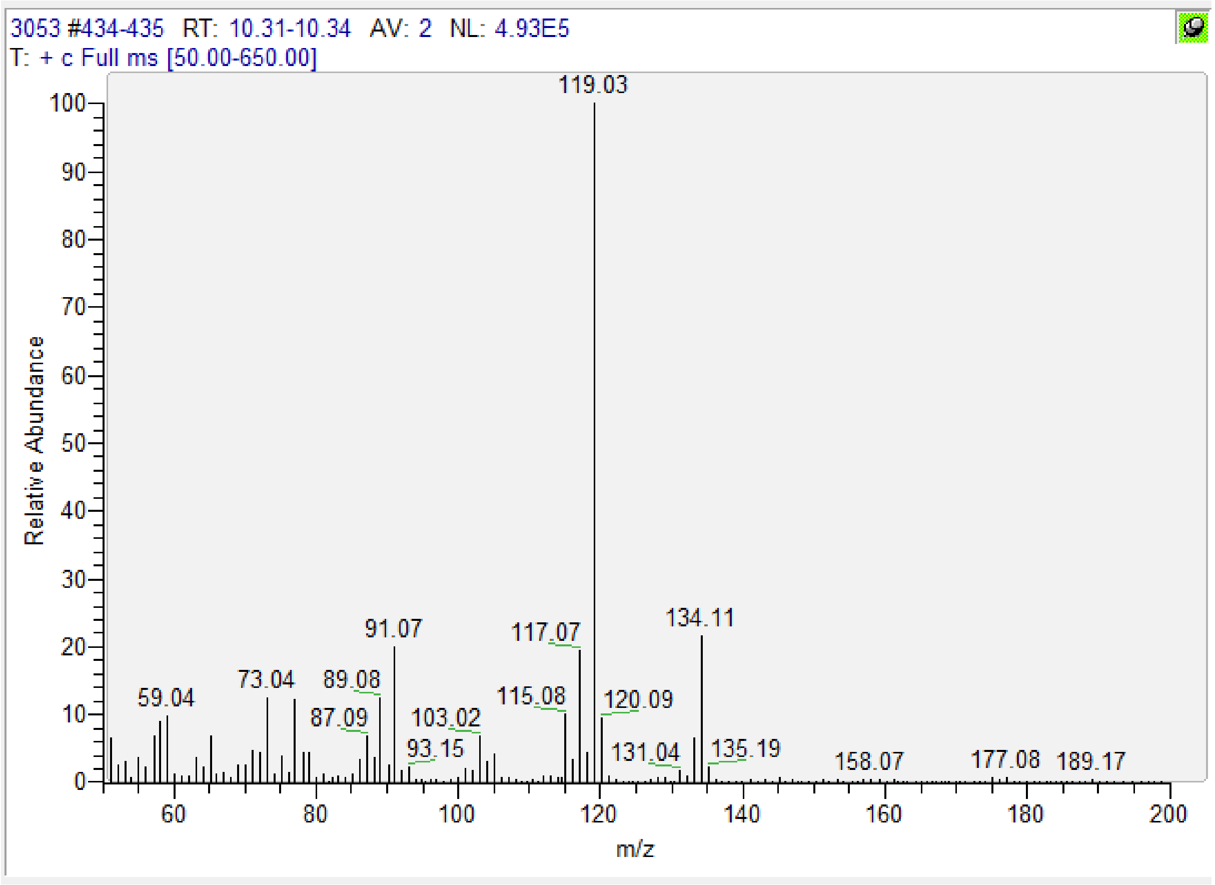

3053 #434-435 RT: 10.31-10.34 AV: 2 NL: 4.93E5
T: + c Full ms [50.00-650.00]
Relative Abundance
m/z
119.03
134.11
91.07
117.07
73.04
89.08
59.04
115.08
120.09
87.09
103.02
93.15
131.04
135.19
158.07
177.08
189.17

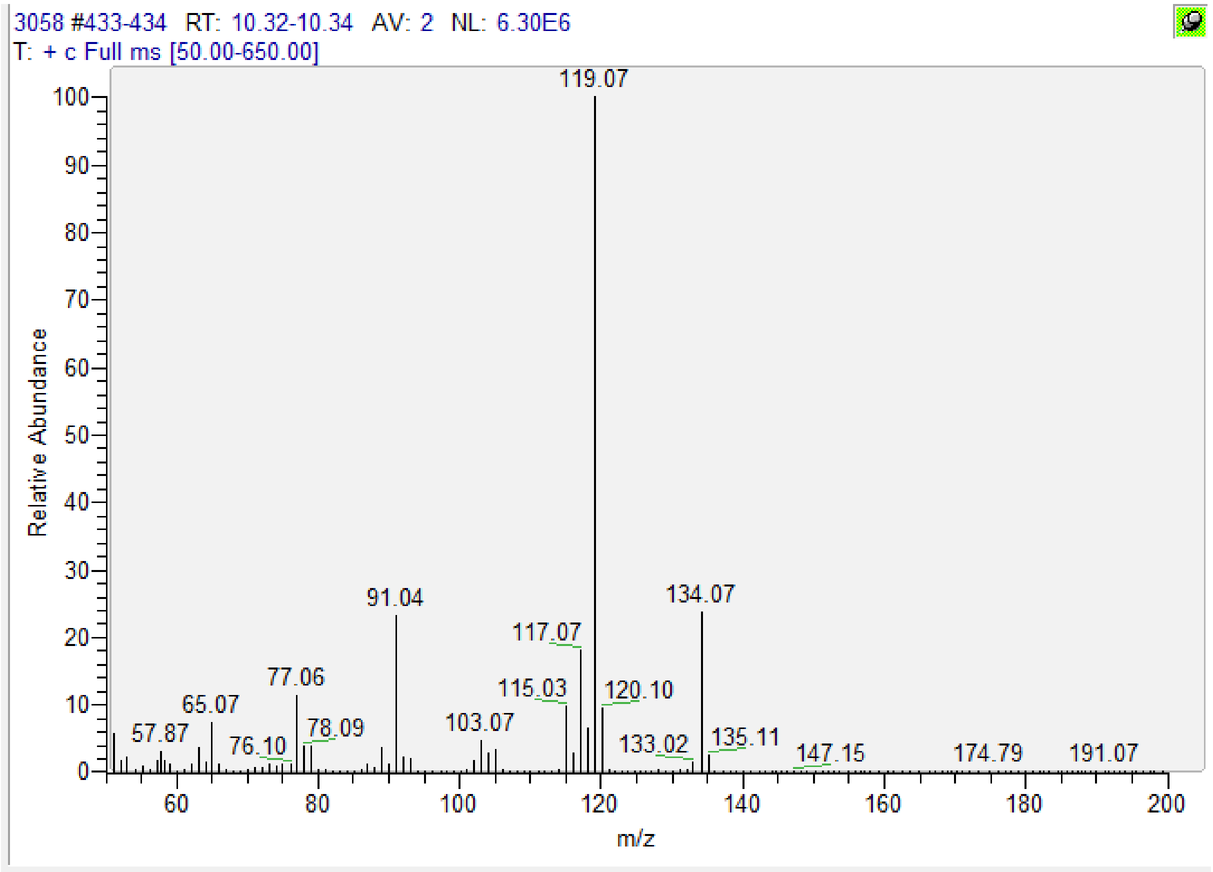
3058 #433-434 RT: 10.32-10.34 AV: 2 NL: 6.30E6
T: + c Full ms [50.00-650.00]
119.07
91.04
134.07
117.07
77.06
115.03
120.10
65.07
78.09
103.07
57.87
76.10
133.02
135.11
147.15
174.79
191.07
Relative Abundance
m/z

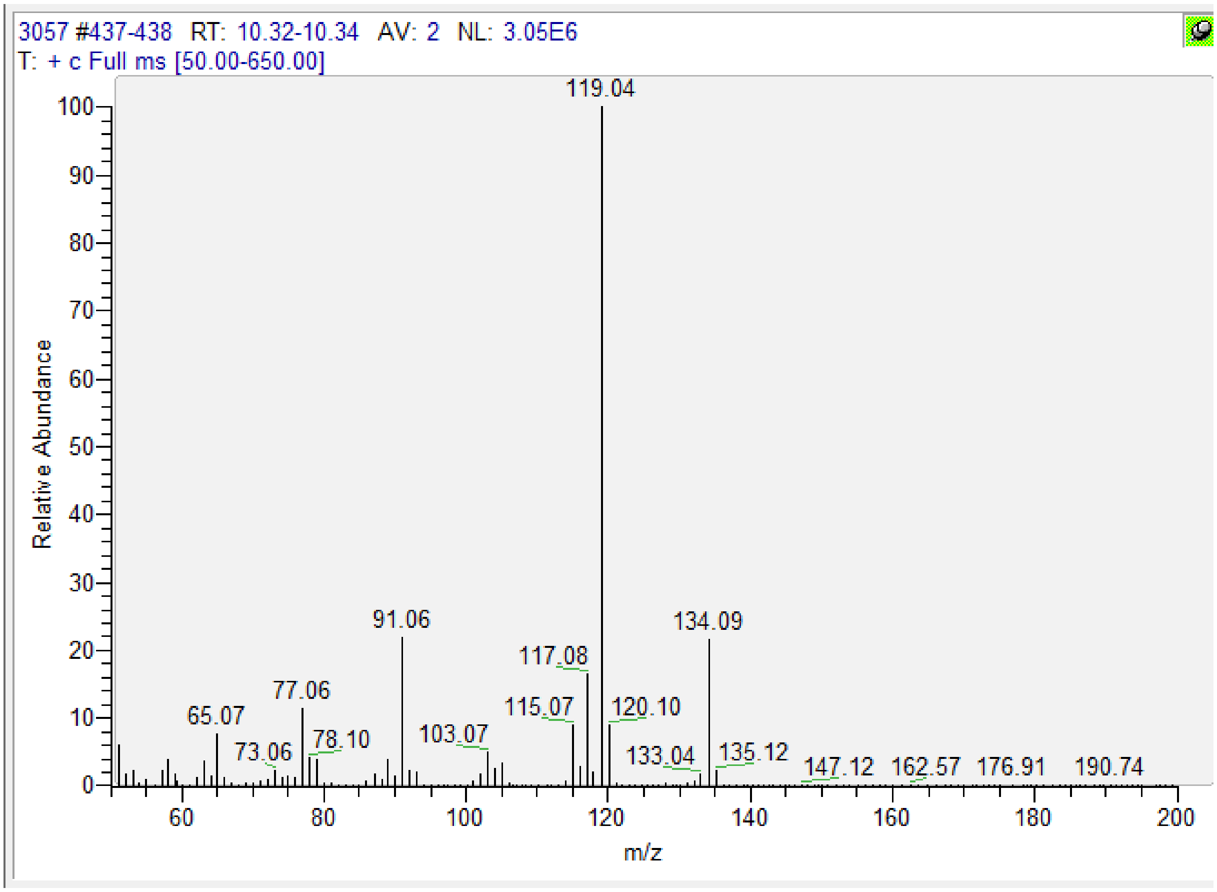
3057 #437-438 RT: 10.32-10.34 AV: 2 NL: 3.05E6
T: + c Full ms [50.00-650.00]
Relative Abundance
m/z
119.04
91.06
134.09
117.08
77.06
65.07
115.07
120.10
103.07
78.10
73.06
133.04
135.12
147.12
162.57
176.91
190.74

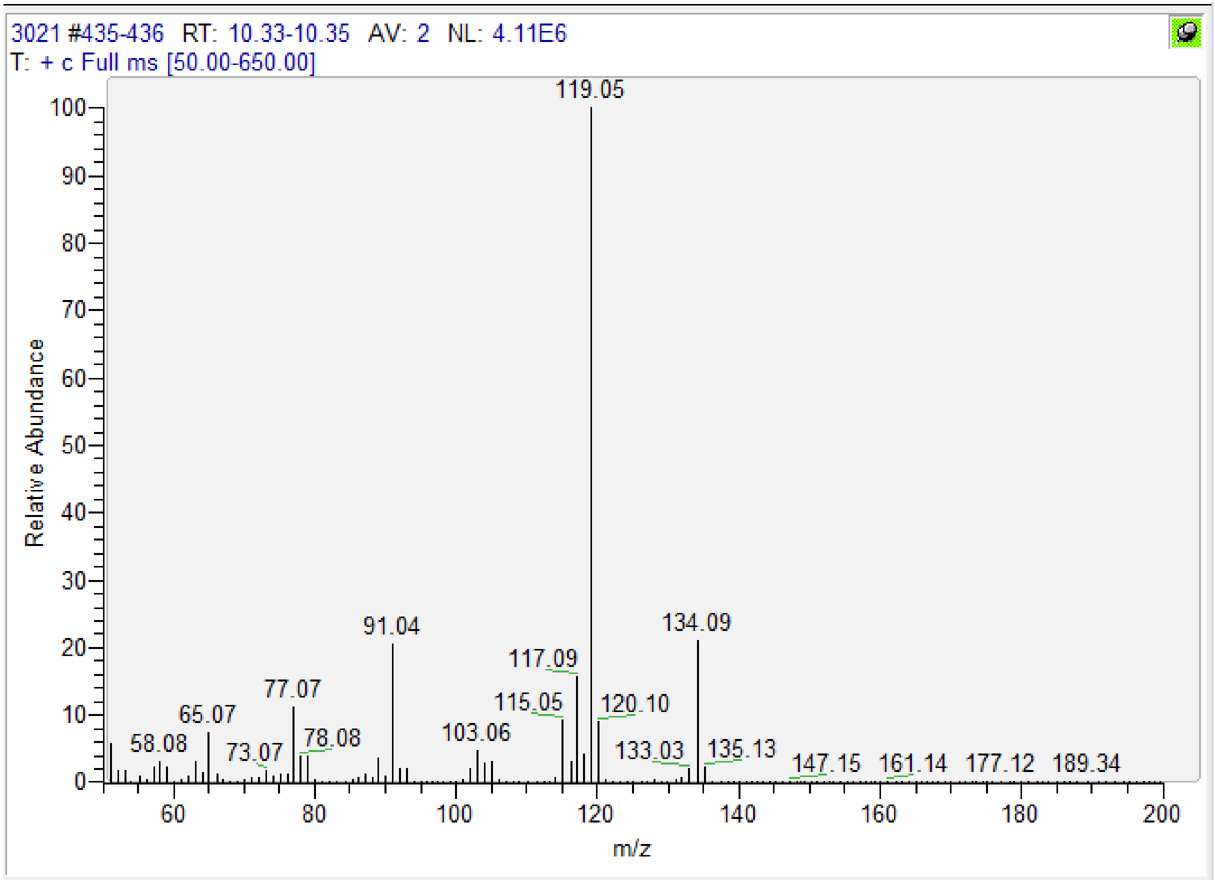

3021 #435-436 RT: 10.33-10.35 AV: 2 NL: 4.11E6
T: + c Full ms [50.00-650.00]
119.05
91.04
134.09
117.09
77.07
115.05
120.10
65.07
103.06
58.08
78.08
73.07
133.03
135.13
147.15
161.14
177.12
189.34
Relative Abundance
m/z

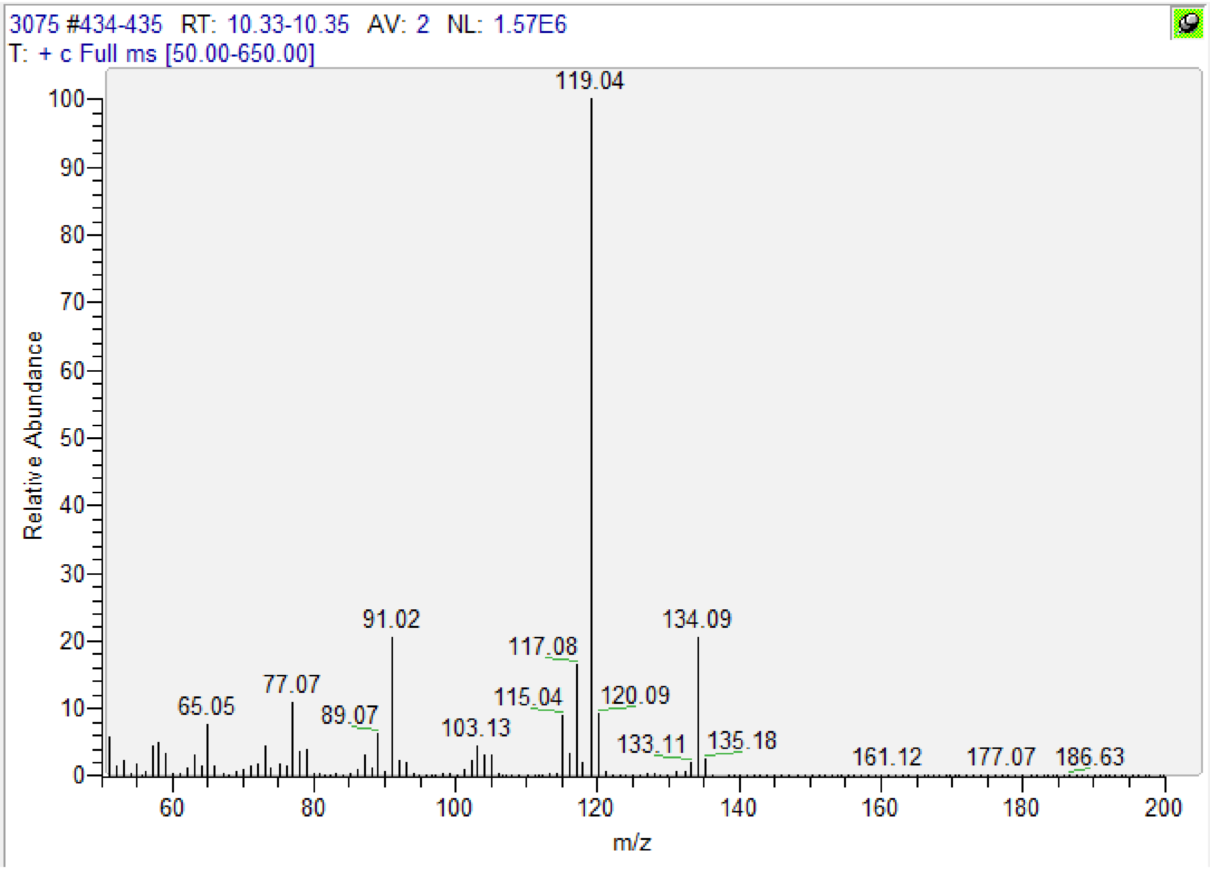
3075 #434-435 RT: 10.33-10.35 AV: 2 NL: 1.57E6
T: + c Full ms [50.00-650.00]
119.04
91.02
134.09
117.08
77.07
115.04
120.09
65.05
89.07
103.13
133.11
135.18
161.12
177.07
186.63
Relative Abundance
m/z
0
10
20
30
40
50
60
70
80
90
100
60
80
100
120
140
160
180
200

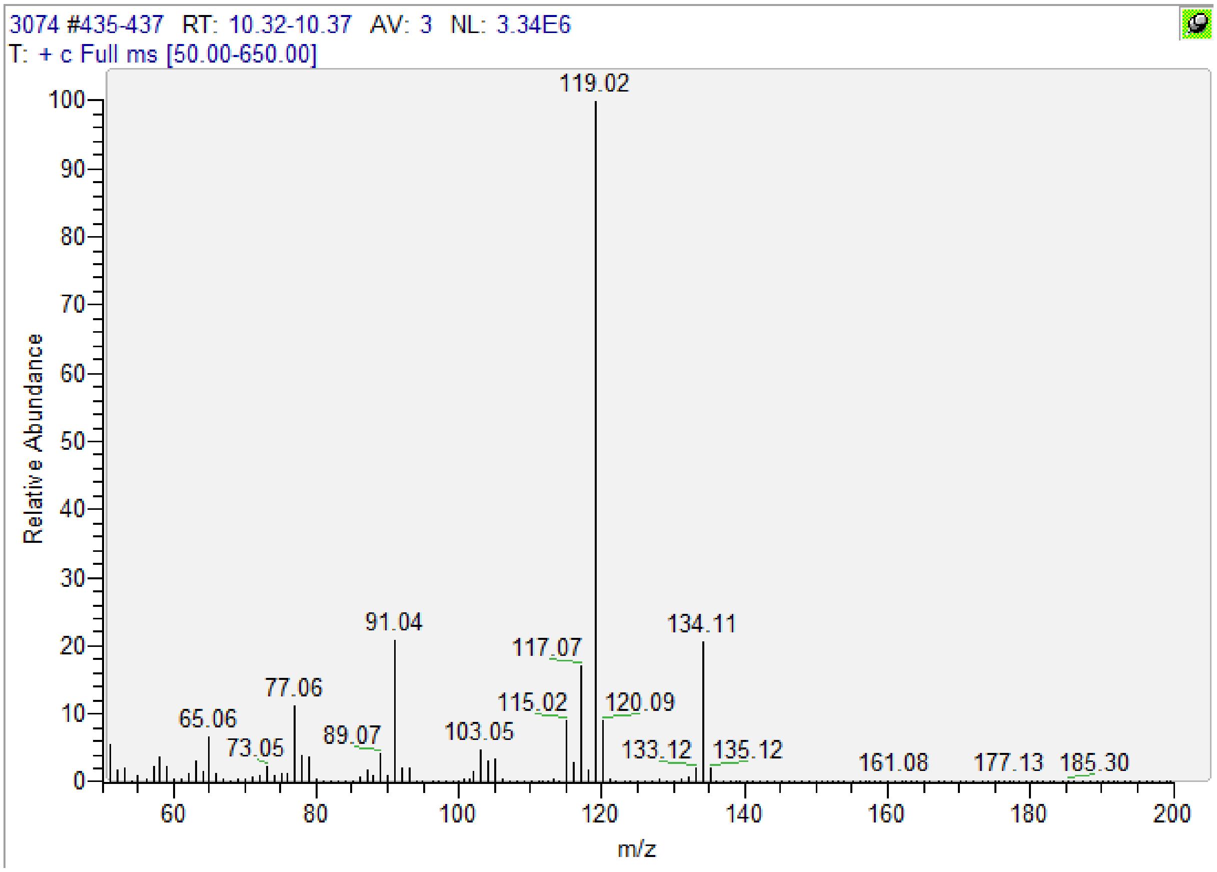
3074 #435-437 RT: 10.32-10.37 AV: 3 NL: 3.34E6
T: + c Full ms [50.00-650.00]
119.02
134.11
91.04
117.07
77.06
115.02
120.09
65.06
103.05
89.07
73.05
133.12
135.12
161.08
177.13
185.30
Relative Abundance
m/z

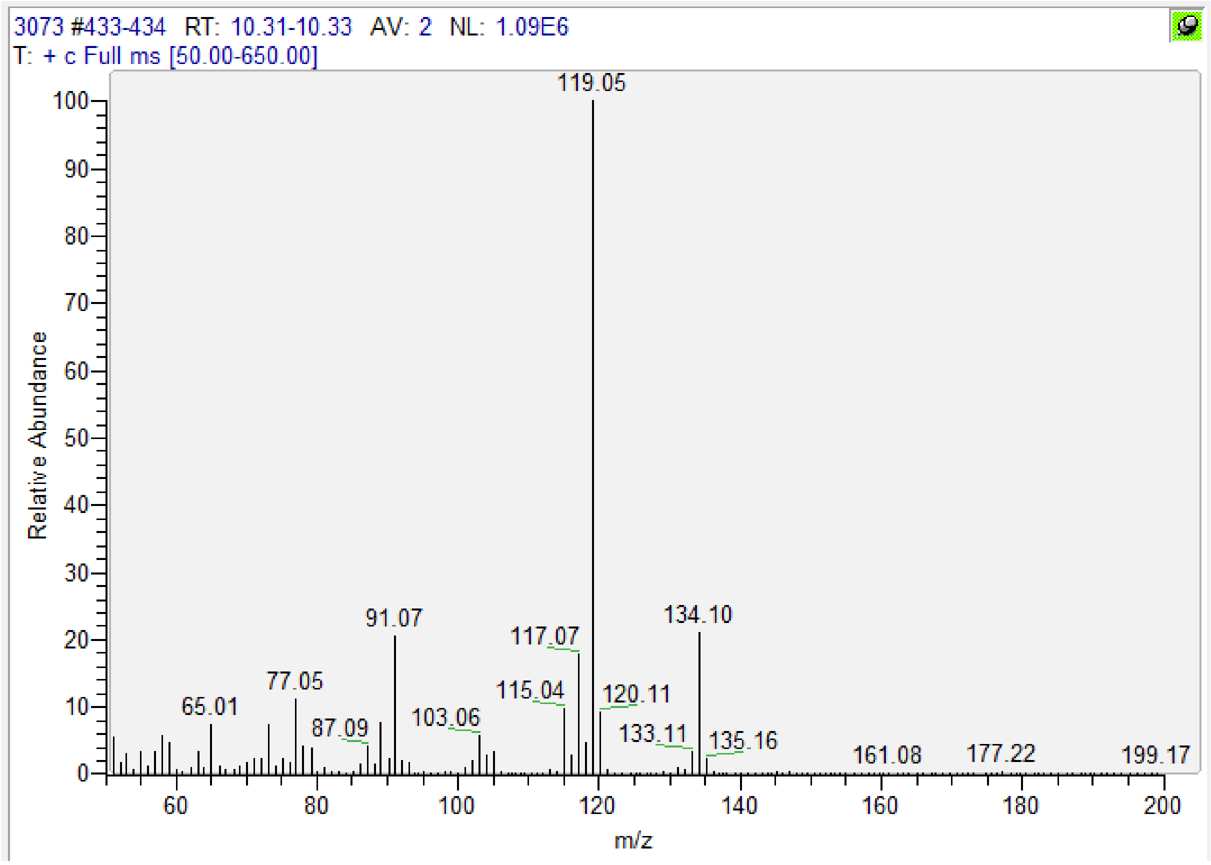
3073 #433-434 RT: 10.31-10.33 AV: 2 NL: 1.09E6
T: + c Full ms [50.00-650.00]
119.05
134.10
91.07
117.07
77.05
115.04
120.11
65.01
103.06
87.09
133.11
135.16
161.08
177.22
199.17
Relative Abundance
m/z

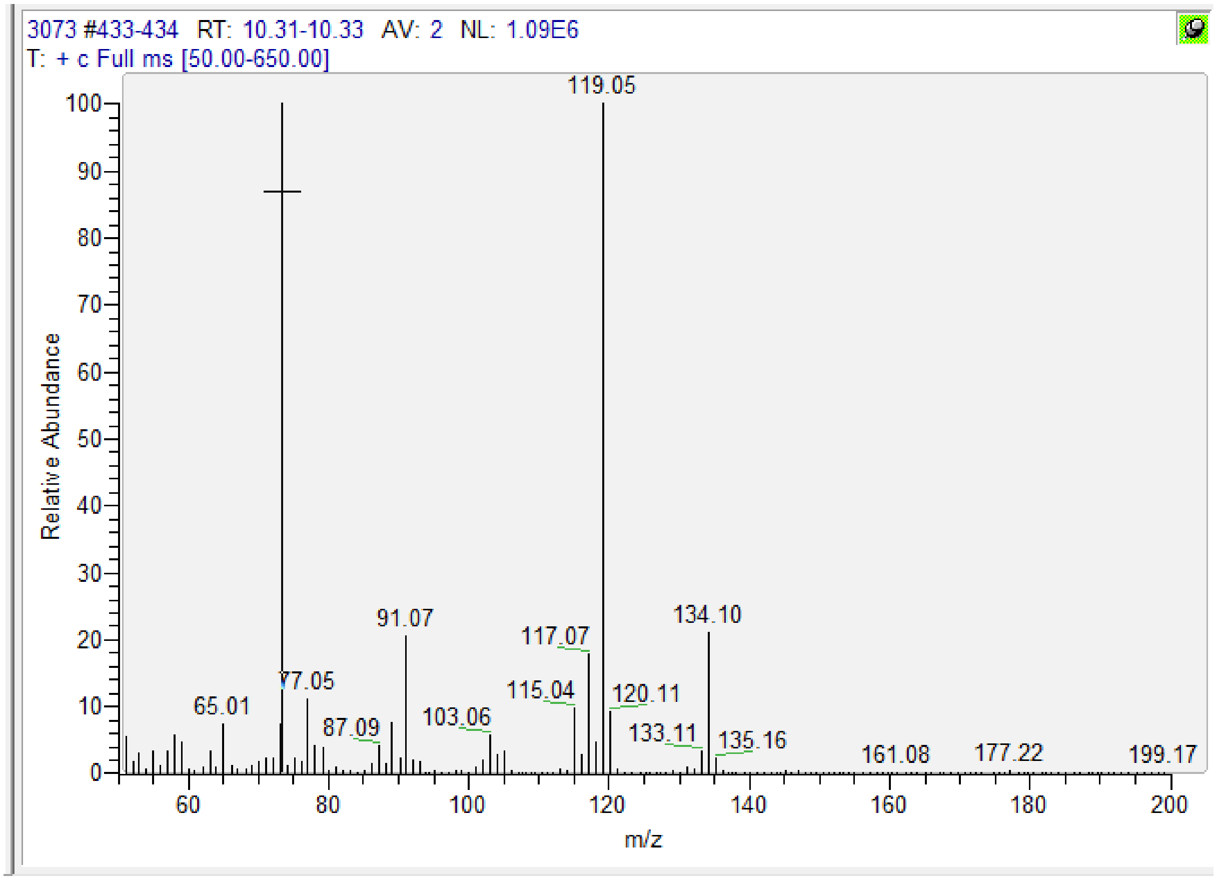

3073 #433-434 RT: 10.31-10.33 AV: 2 NL: 1.09E6
T: + c Full ms [50.00-650.00]
Relative Abundance
m/z
119.05
91.07
117.07
134.10
77.05
65.01
115.04
120.11
87.09
103.06
133.11
135.16
161.08
177.22
199.17

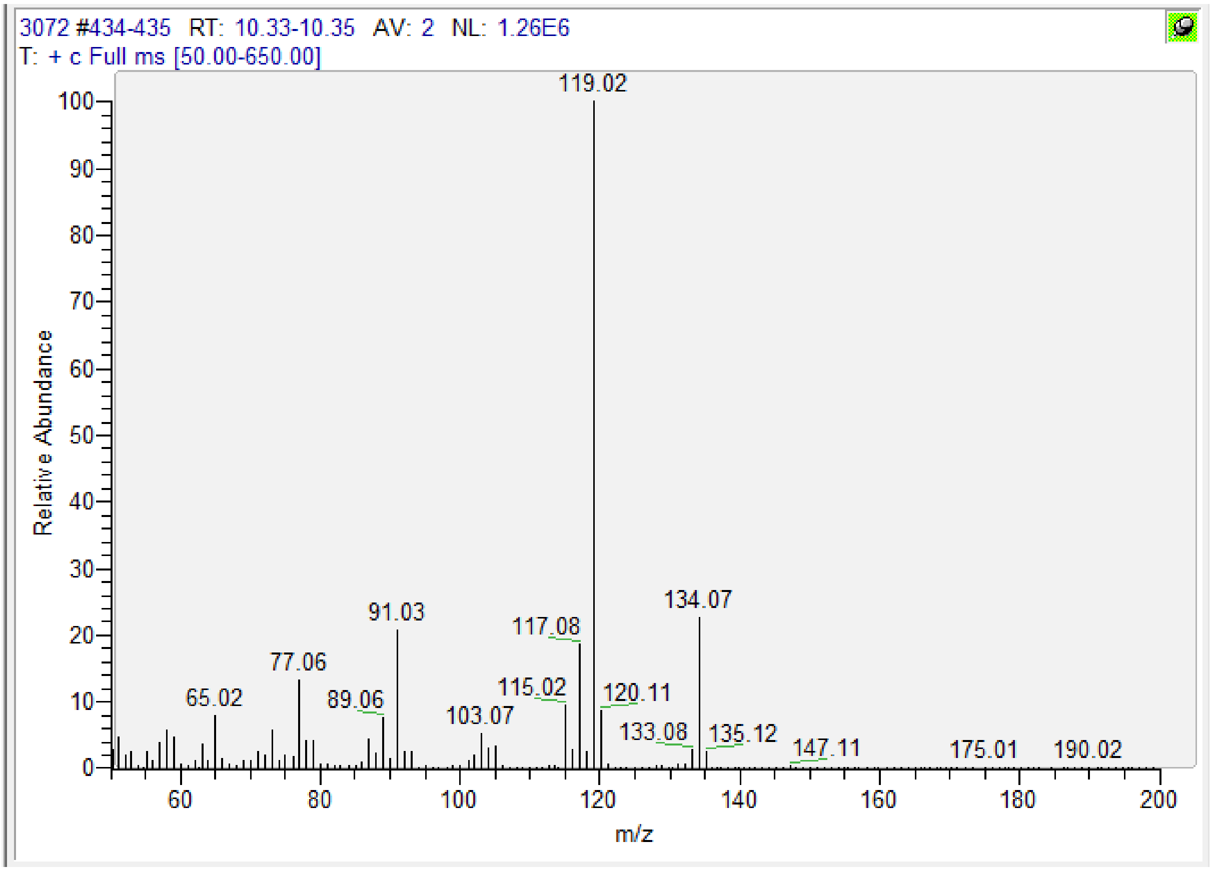
3072 #434-435 RT: 10.33-10.35 AV: 2 NL: 1.26E6
T: + c Full ms [50.00-650.00]
Relative Abundance
m/z
119.02
134.07
91.03
117.08
77.06
65.02
89.06
115.02
120.11
103.07
133.08
135.12
147.11
175.01
190.02

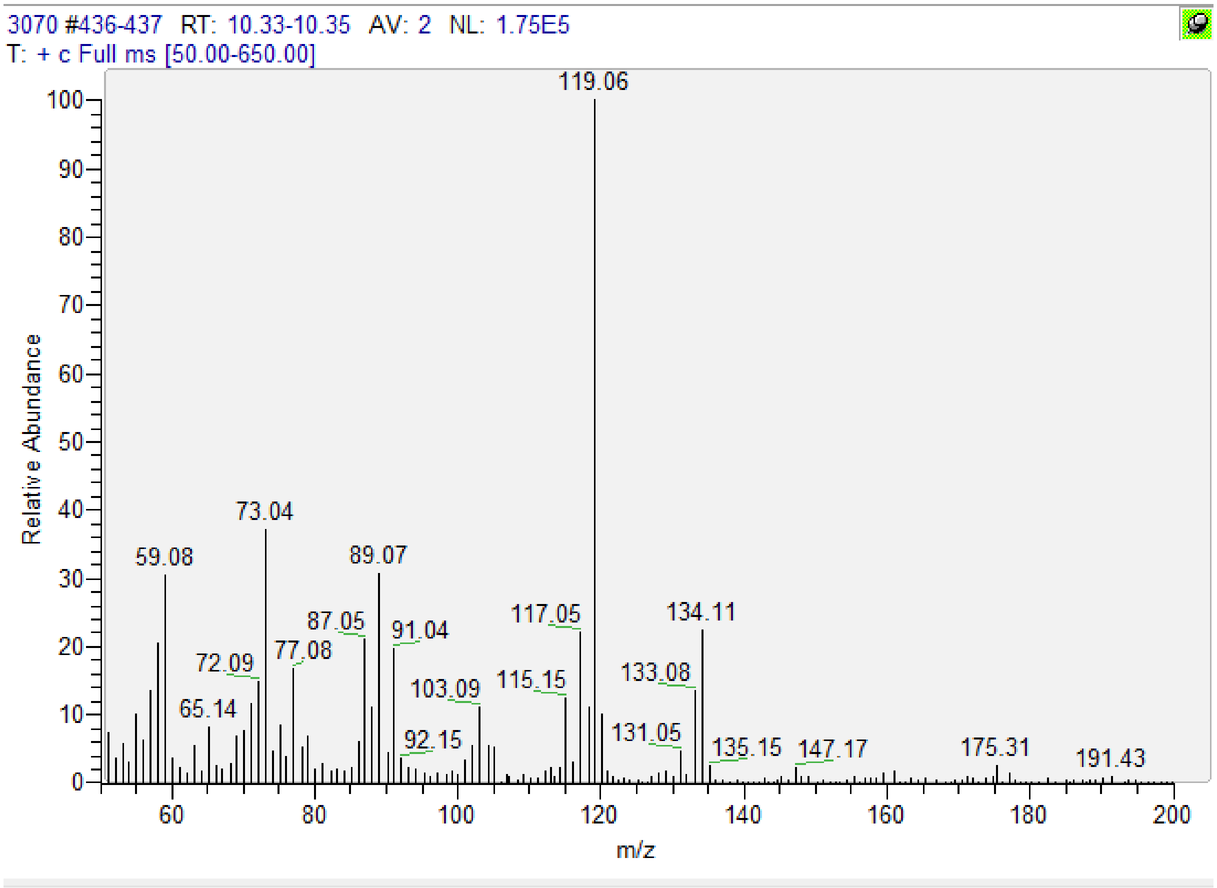

3070 #436-437 RT: 10.33-10.35 AV: 2 NL: 1.75E5
T: + c Full ms [50.00-650.00]
Relative Abundance
m/z
119.06
73.04
59.08
89.07
87.05
91.04
117.05
134.11
72.09
77.08
65.14
103.09
115.15
133.08
92.15
131.05
135.15
147.17
175.31
191.43

Rose oxide

Reference:

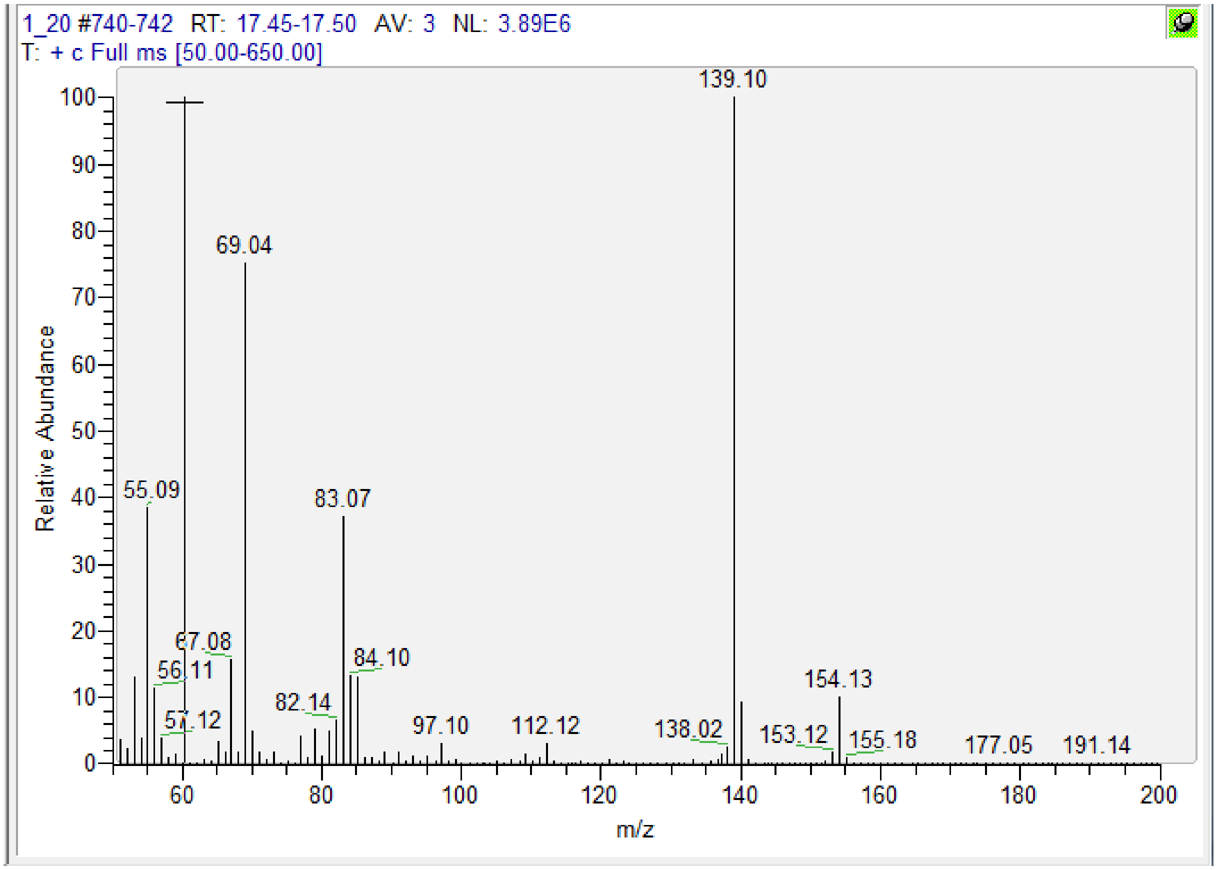

Samples:

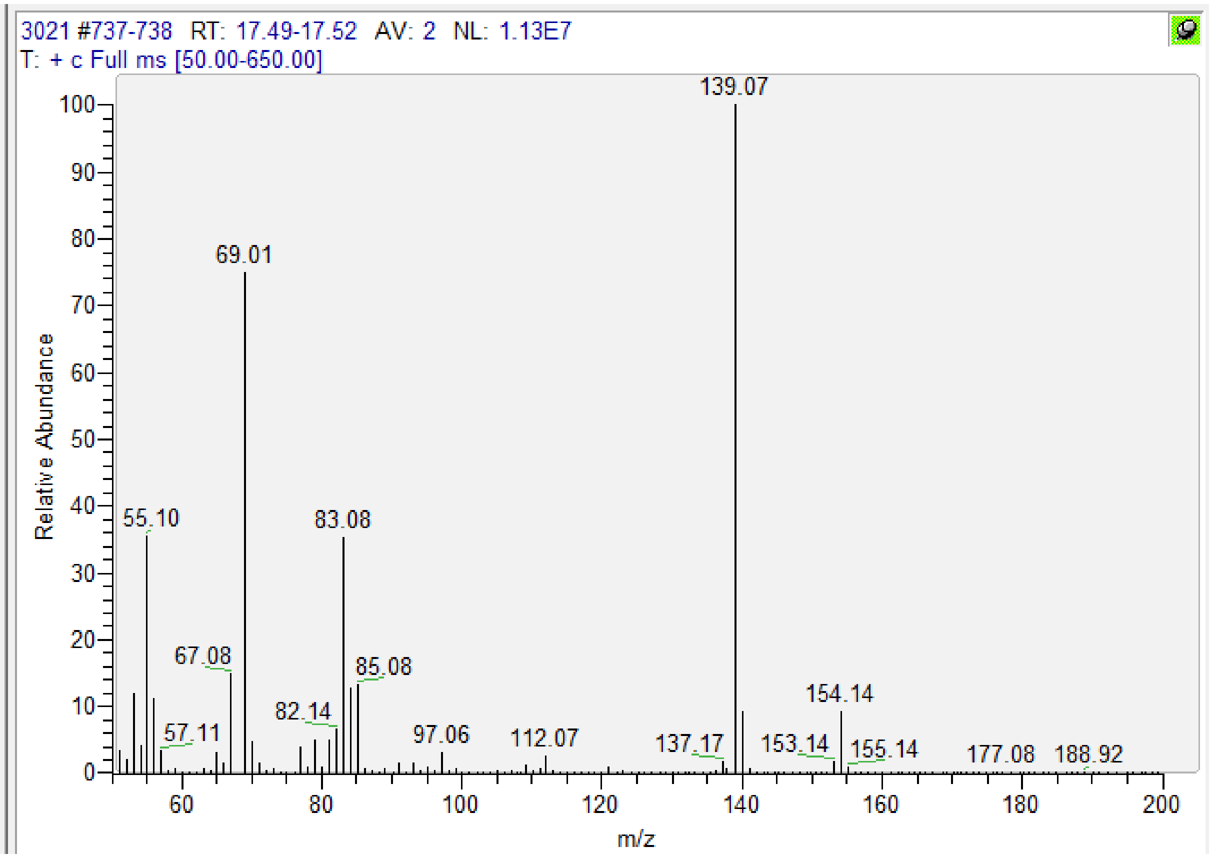

3021 #737-738 RT: 17.49-17.52 AV: 2 NL: 1.13E7
T: + c Full ms [50.00-650.00]
Relative Abundance
m/z
139.07
69.01
55.10
83.08
67.08
85.08
57.11
82.14
97.06
112.07
137.17
153.14
154.14
155.14
177.08
188.92

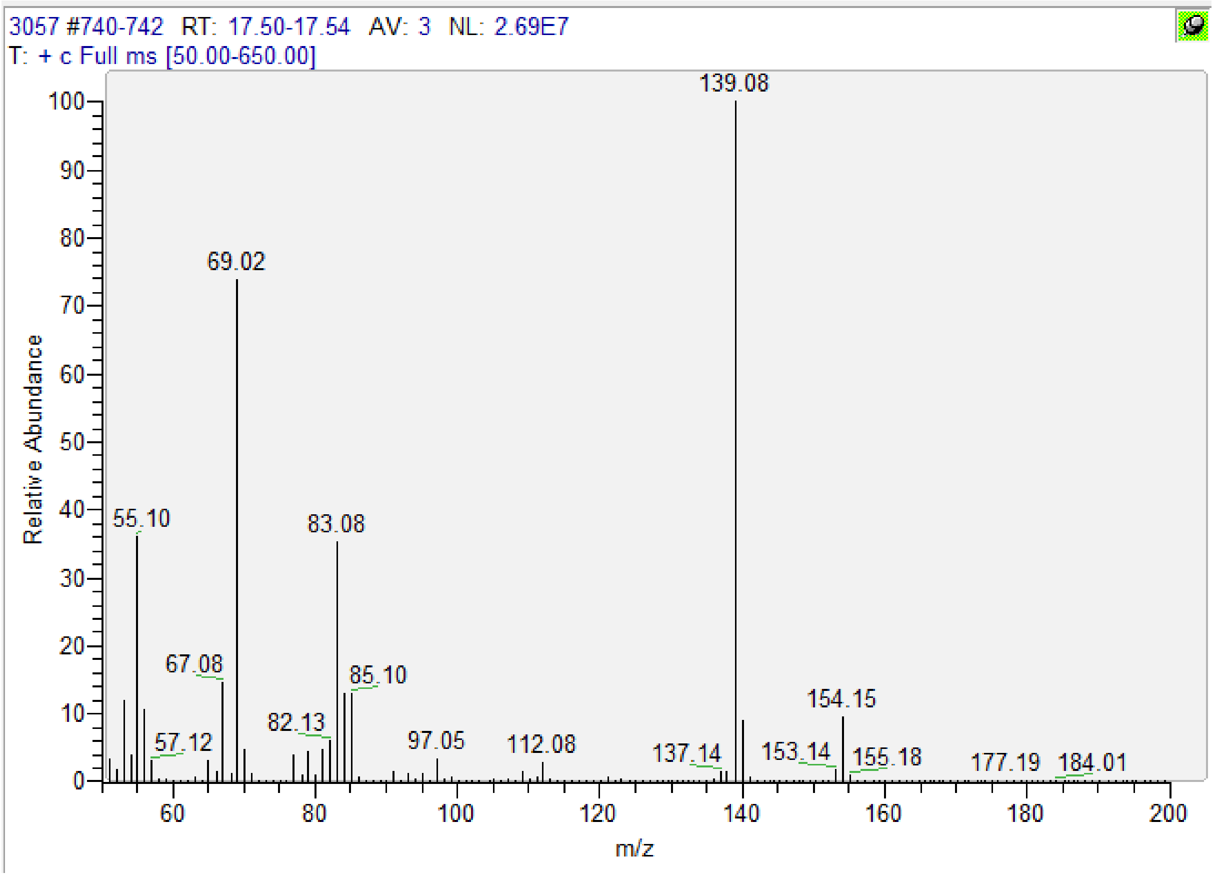

3057 #740-742 RT: 17.50-17.54 AV: 3 NL: 2.69E7
T: + c Full ms [50.00-650.00]
Relative Abundance
m/z
139.08
69.02
55.10
83.08
67.08
85.10
57.12
82.13
97.05
112.08
137.14
153.14
154.15
155.18
177.19
184.01

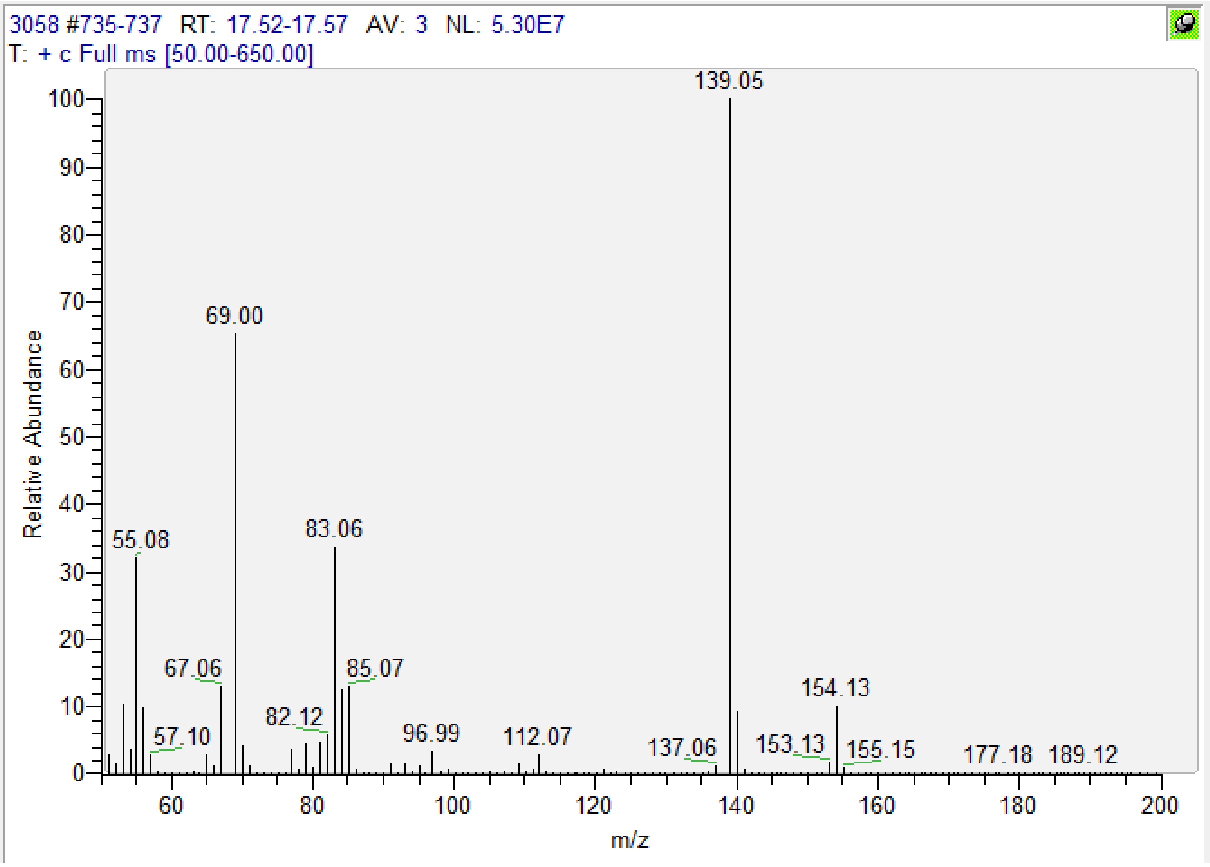

3058 #735-737 RT: 17.52-17.57 AV: 3 NL: 5.30E7
T: + c Full ms [50.00-650.00]
Relative Abundance
m/z
139.05
69.00
83.06
55.08
67.06
85.07
57.10
82.12
96.99
112.07
137.06
153.13
154.13
155.15
177.18
189.12

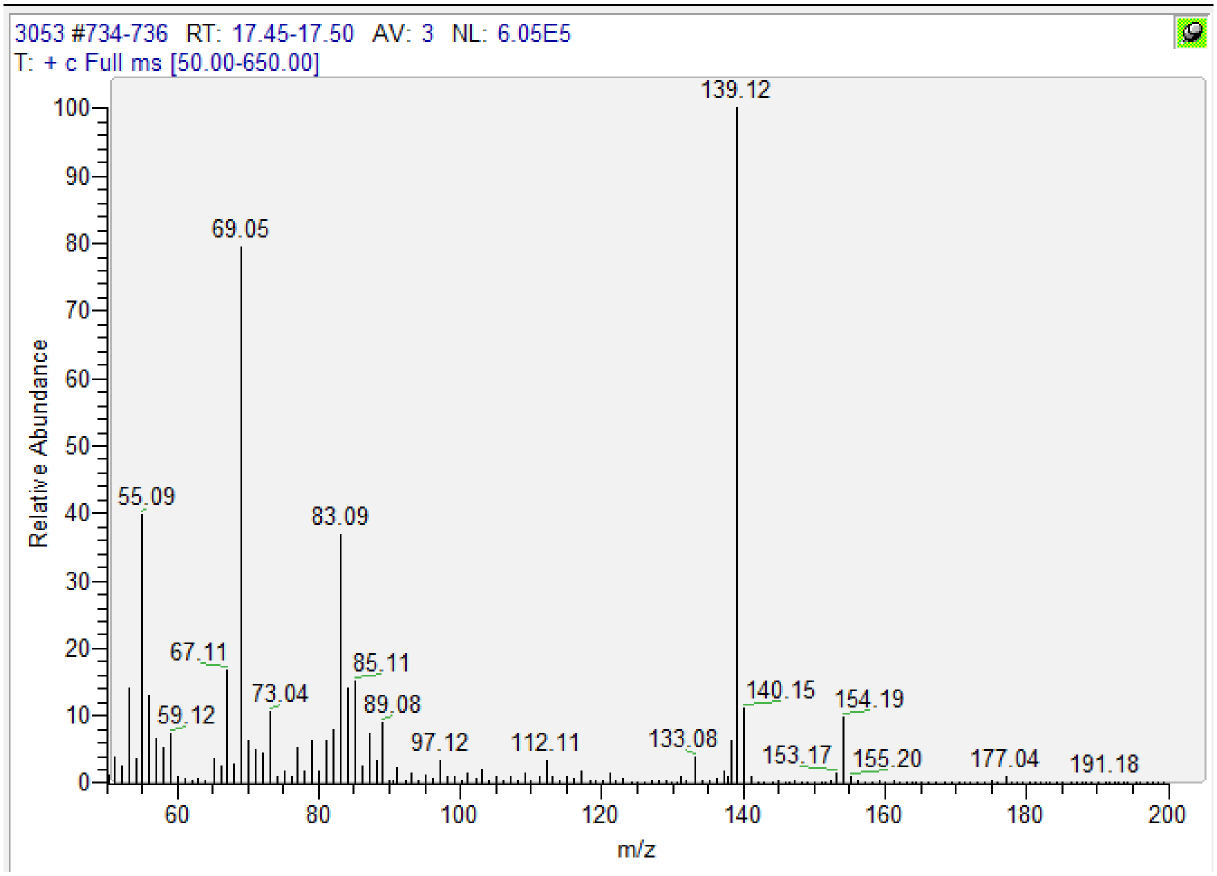
3053 #734-736 RT: 17.45-17.50 AV: 3 NL: 6.05E5
T: + c Full ms [50.00-650.00]
139.12
69.05
55.09
83.09
67.11
85.11
59.12
73.04
89.08
97.12
112.11
133.08
140.15
154.19
153.17
155.20
177.04
191.18
Relative Abundance
m/z

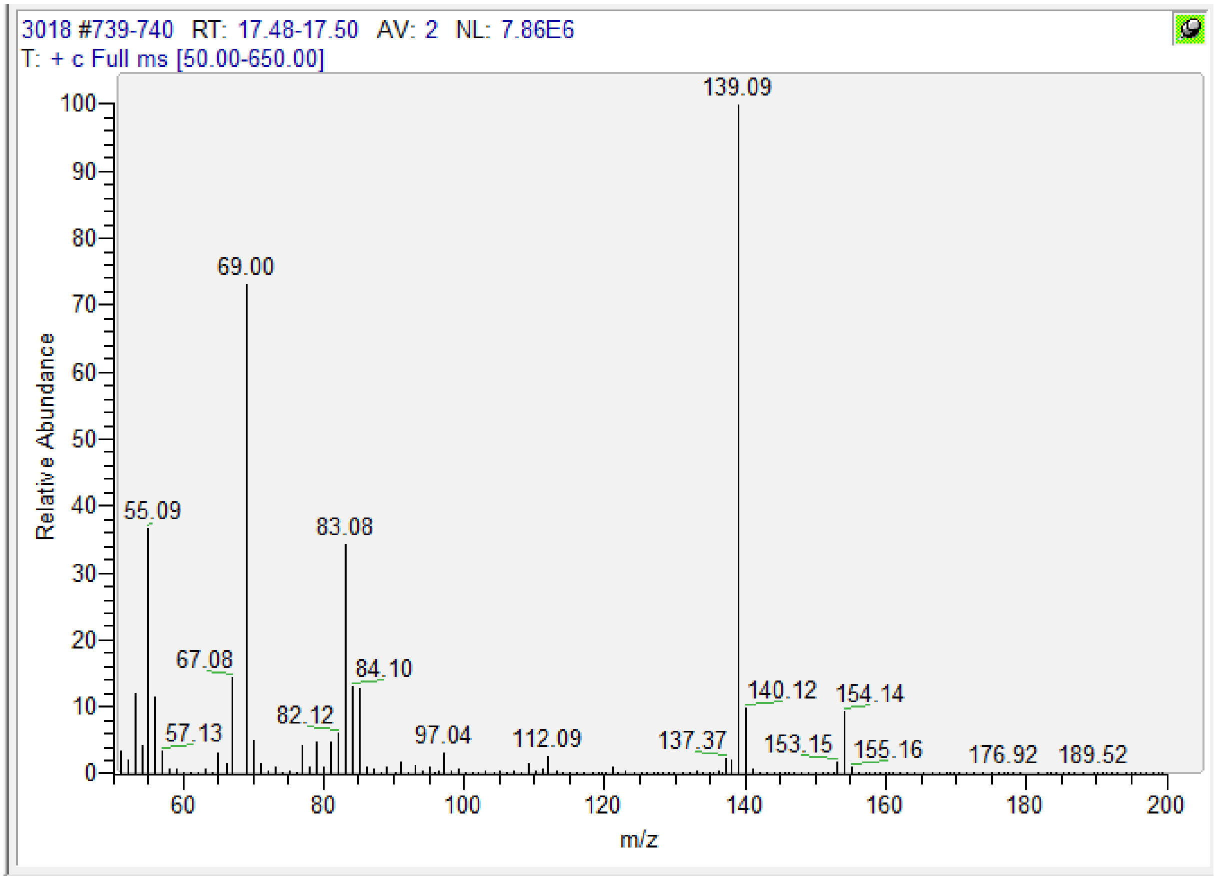
3018 #739-740 RT: 17.48-17.50 AV: 2 NL: 7.86E6
T: + c Full ms [50.00-650.00]
Relative Abundance
m/z
139.09
69.00
55.09
83.08
67.08
84.10
57.13
82.12
97.04
112.09
137.37
140.12
153.15
154.14
155.16
176.92
189.52

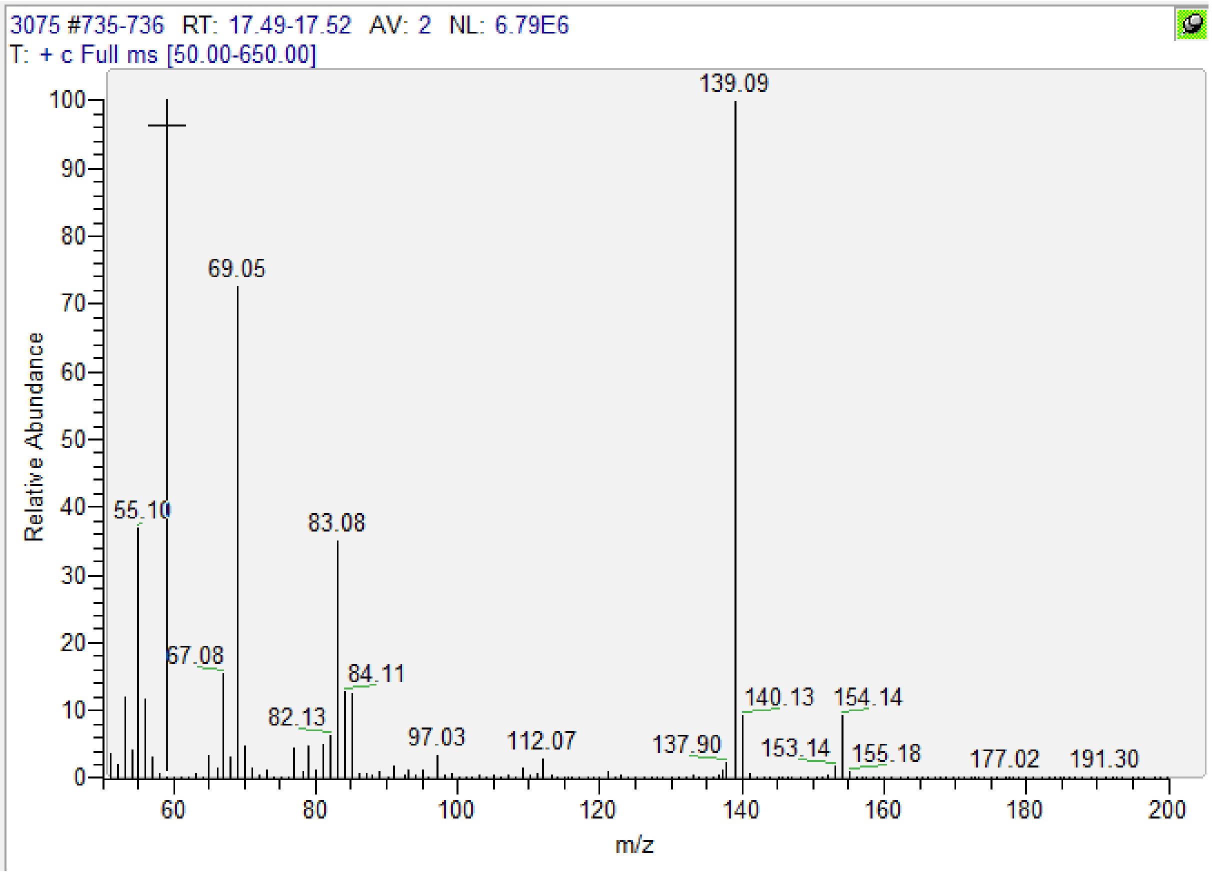
3075 #735-736 RT: 17.49-17.52 AV: 2 NL: 6.79E6
T: + c Full ms [50.00-650.00]
139.09
69.05
55.10
83.08
67.08
84.11
82.13
97.03
112.07
137.90
140.13
154.14
153.14
155.18
177.02
191.30
Relative Abundance
m/z

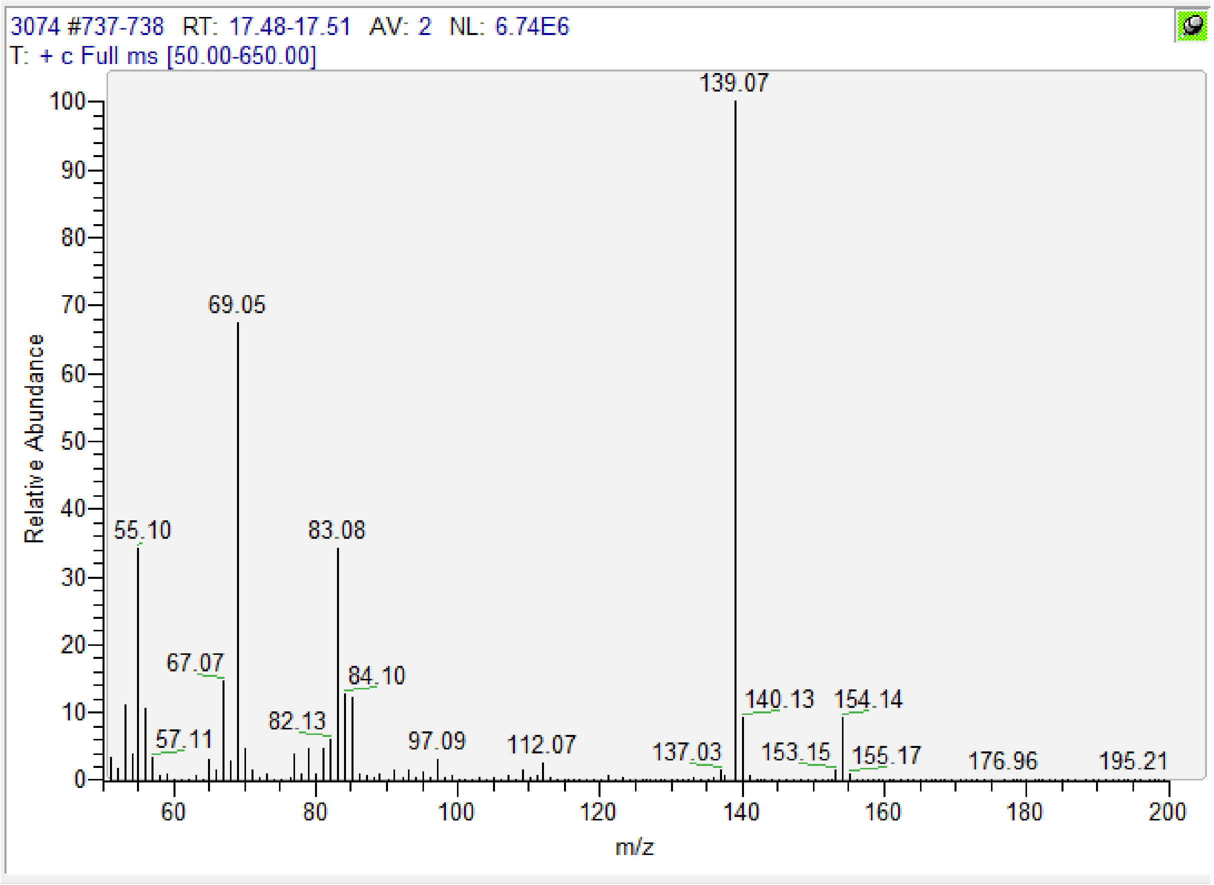
3074 #737-738 RT: 17.48-17.51 AV: 2 NL: 6.74E6
T: + c Full ms [50.00-650.00]
Relative Abundance
m/z
139.07
69.05
55.10
83.08
67.07
84.10
82.13
57.11
97.09
112.07
137.03
140.13
154.14
153.15
155.17
176.96
195.21

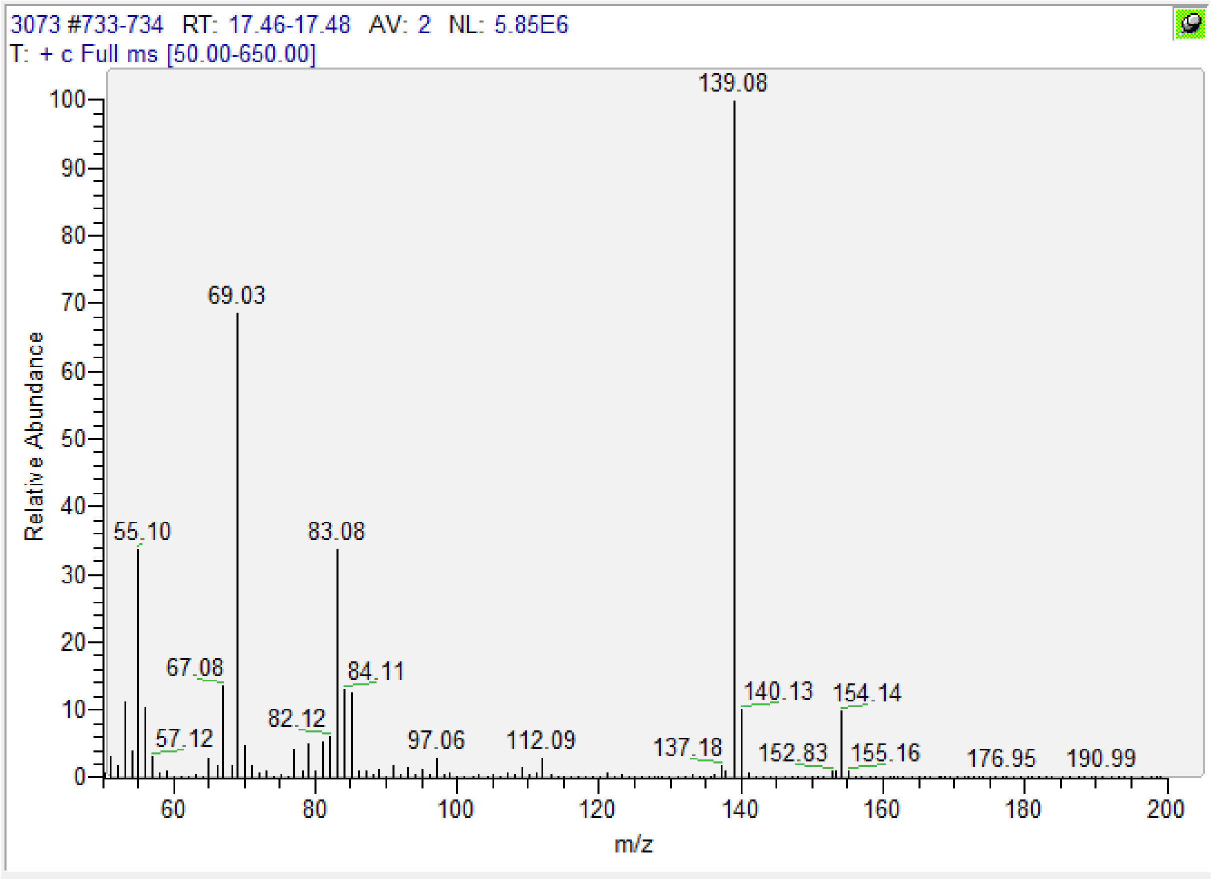

3073 #733-734 RT: 17.46-17.48 AV: 2 NL: 5.85E6
T: + c Full ms [50.00-650.00]
Relative Abundance
m/z
139.08
69.03
55.10
83.08
67.08
84.11
57.12
82.12
97.06
112.09
137.18
140.13
154.14
152.83
155.16
176.95
190.99

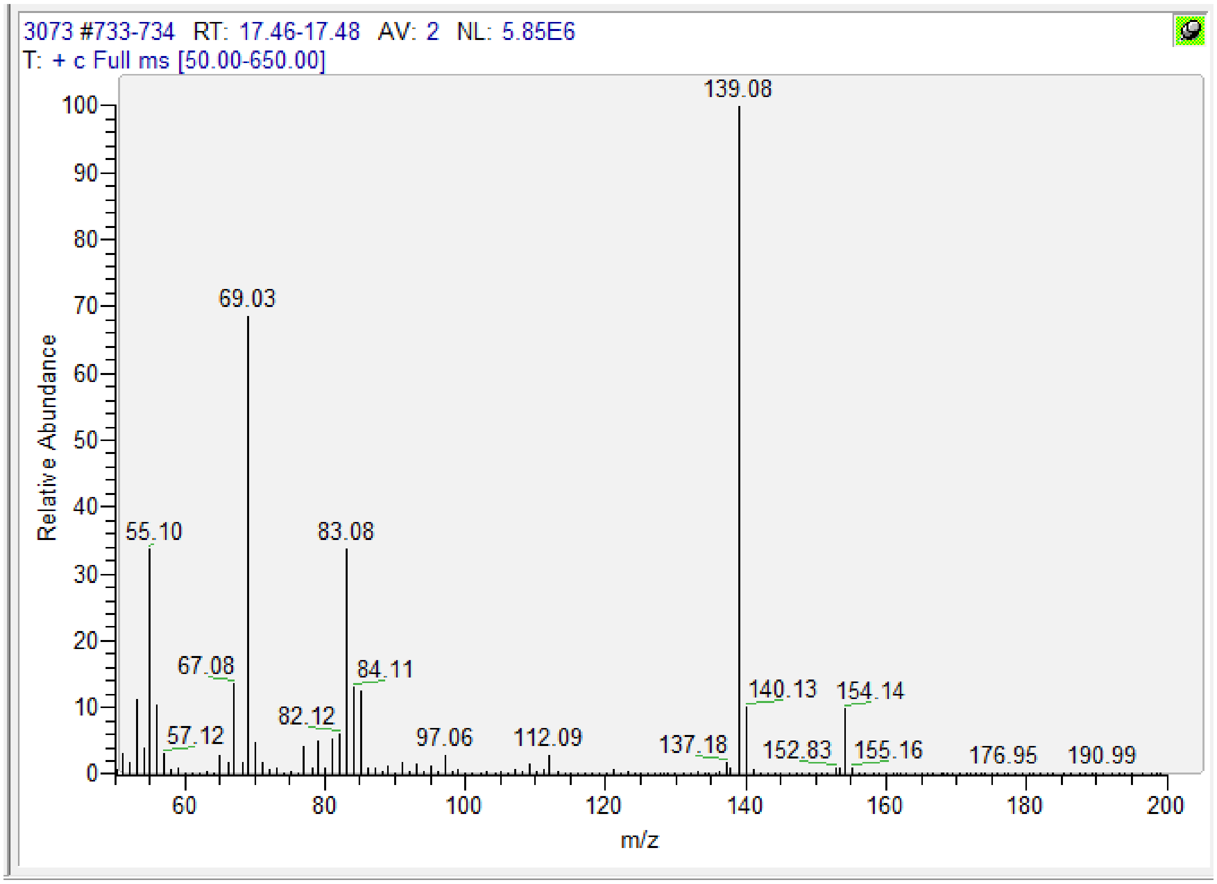

3073 #733-734 RT: 17.46-17.48 AV: 2 NL: 5.85E6
T: + c Full ms [50.00-650.00]
Relative Abundance
m/z
139.08
69.03
55.10
83.08
67.08
84.11
57.12
82.12
97.06
112.09
137.18
140.13
154.14
152.83
155.16
176.95
190.99

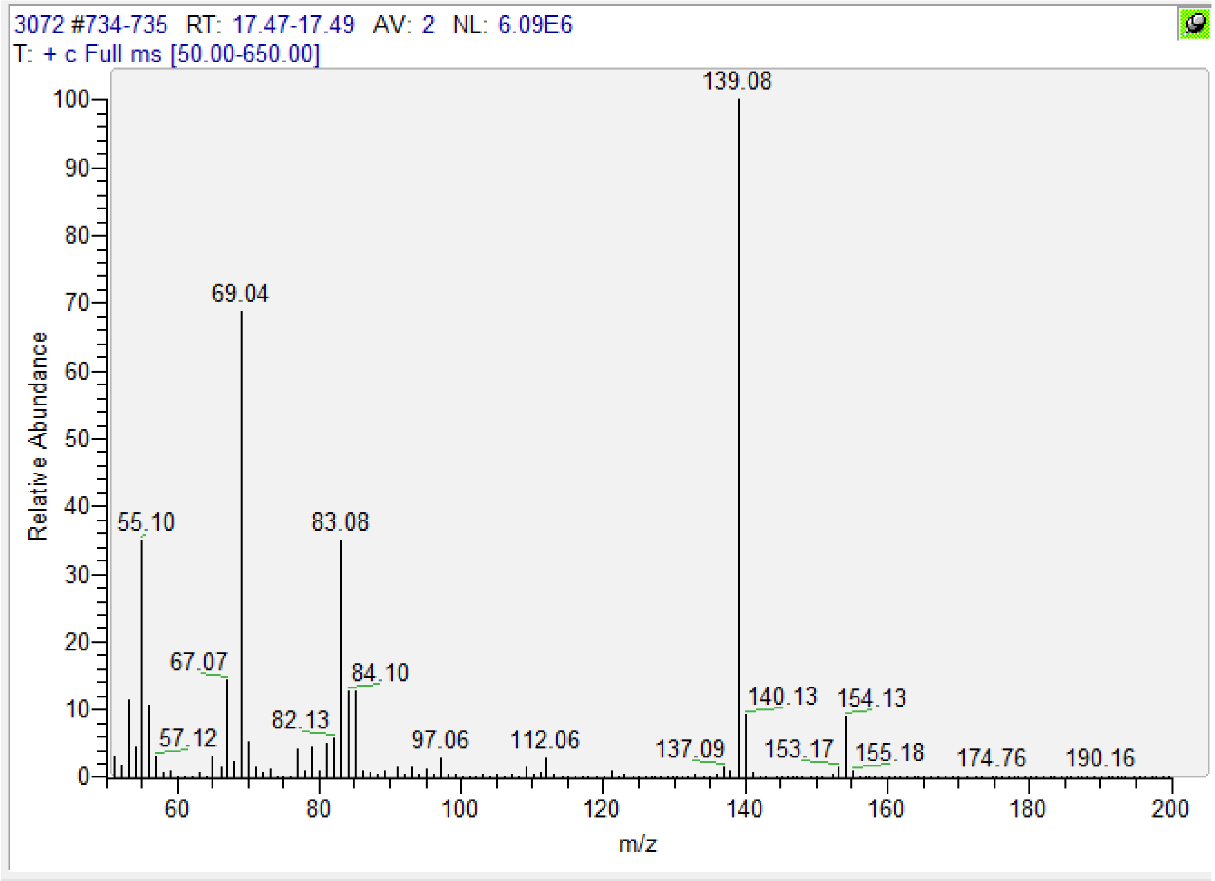

3072 #734-735 RT: 17.47-17.49 AV: 2 NL: 6.09E6
T: + c Full ms [50.00-650.00]
139.08
69.04
55.10
83.08
67.07
84.10
57.12
82.13
97.06
112.06
137.09
140.13
153.17
154.13
155.18
174.76
190.16
Relative Abundance
m/z

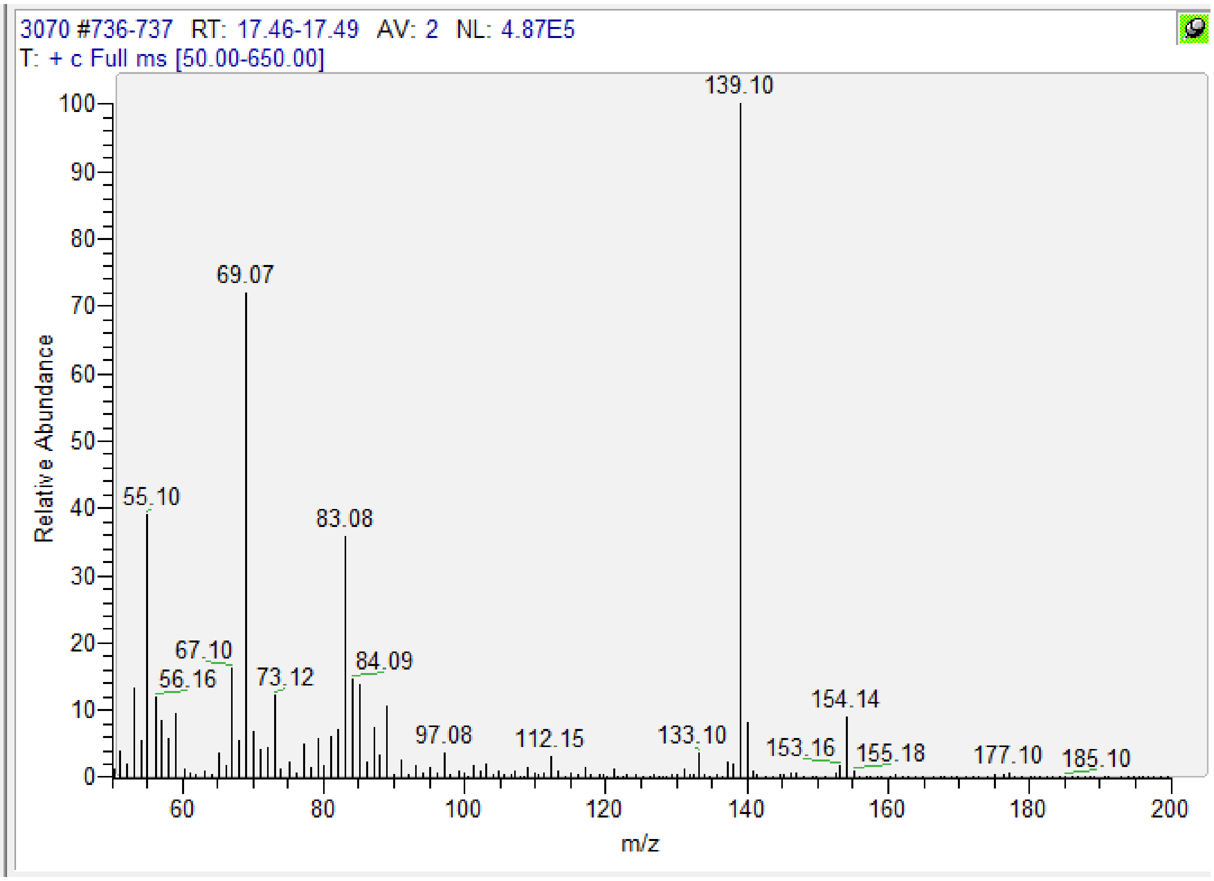

Linalool

Reference

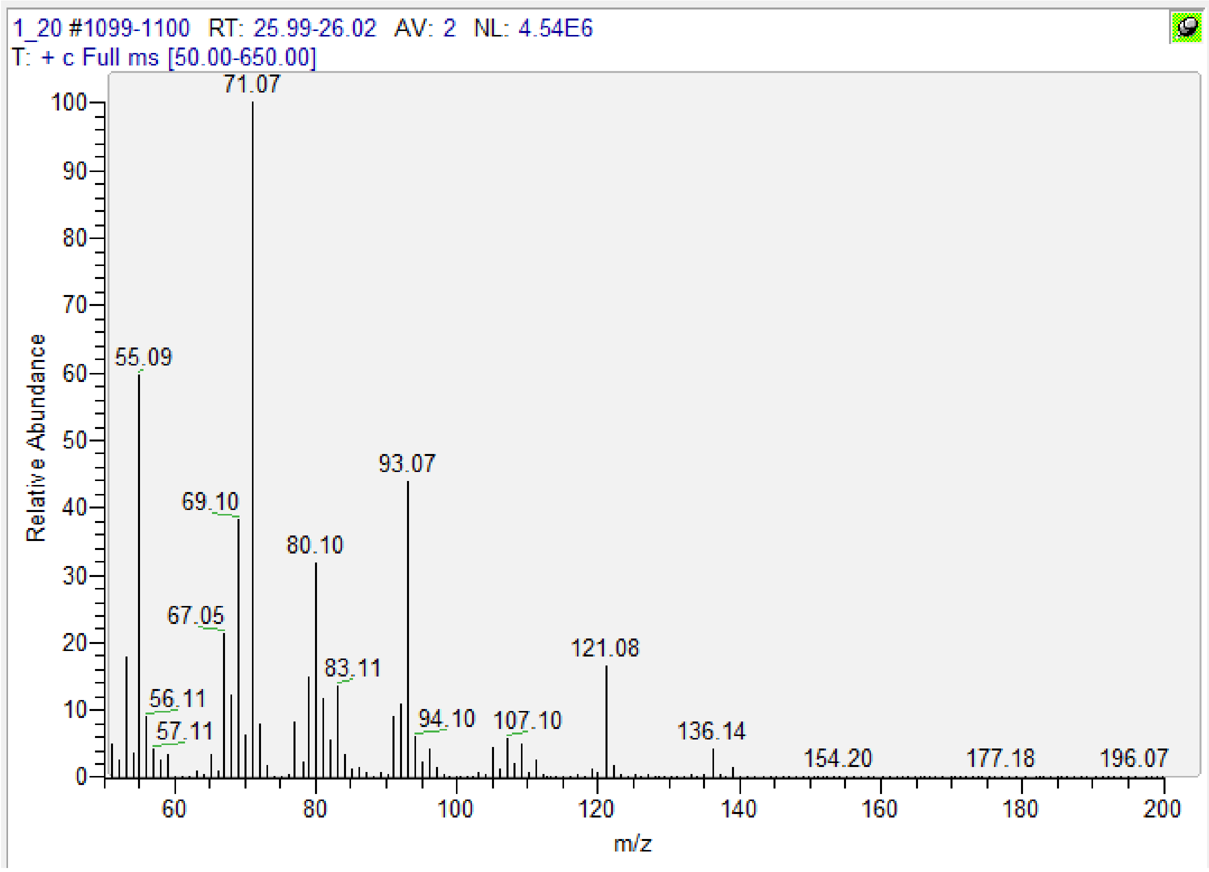

1_20 #1099-1100 RT: 25.99-26.02 AV: 2 NL: 4.54E6
T: + c Full ms [50.00-650.00]
Relative Abundance
m/z
71.07
55.09
93.07
69.10
80.10
67.05
121.08
83.11
56.11
57.11
94.10
107.10
136.14
154.20
177.18
196.07

Samples:

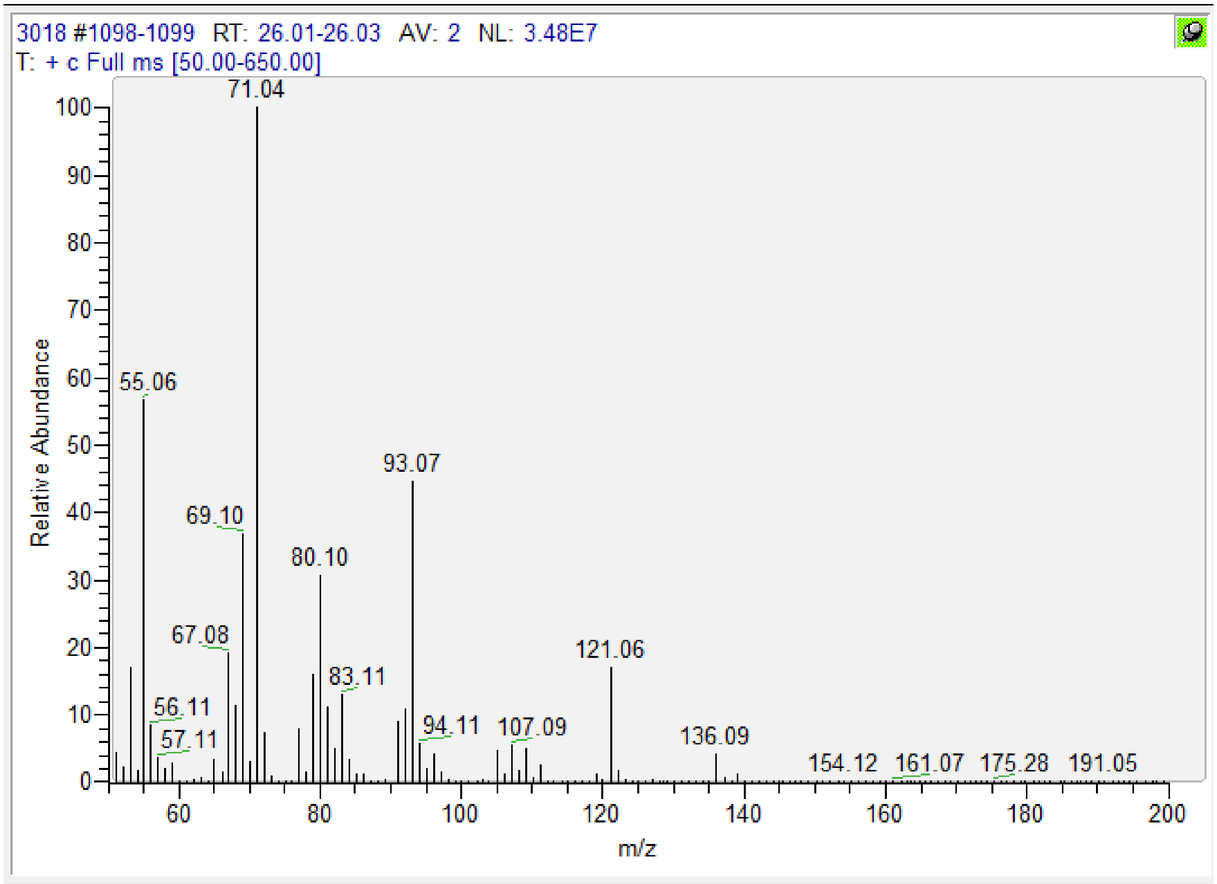

3018 #1098-1099 RT: 26.01-26.03 AV: 2 NL: 3.48E7
T: + c Full ms [50.00-650.00]
Relative Abundance
m/z
71.04
55.06
93.07
69.10
80.10
67.08
121.06
83.11
56.11
57.11
94.11
107.09
136.09
154.12
161.07
175.28
191.05

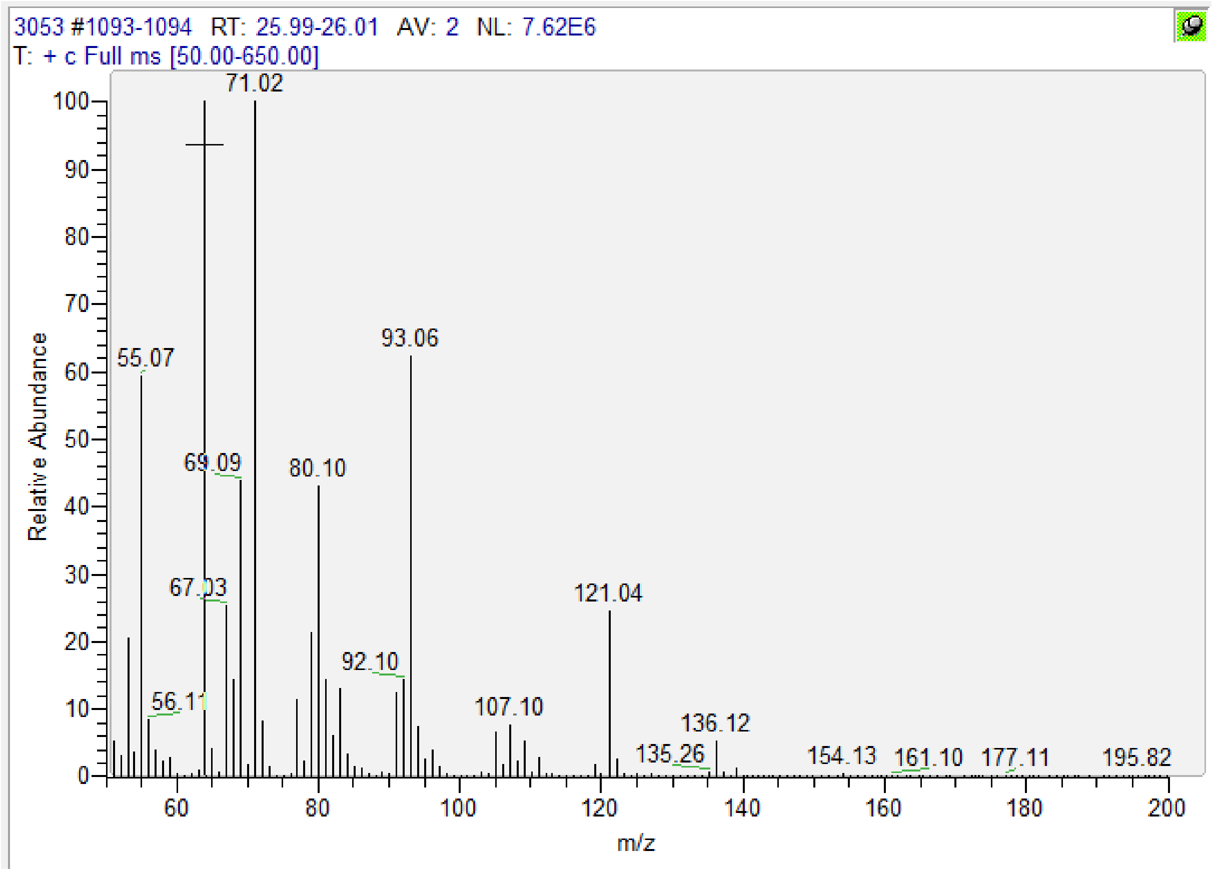

3053 #1093-1094 RT: 25.99-26.01 AV: 2 NL: 7.62E6
T: + c Full ms [50.00-650.00]
Relative Abundance
m/z
71.02
55.07
93.06
69.09
80.10
67.03
121.04
92.10
56.11
107.10
136.12
135.26
154.13
161.10
177.11
195.82

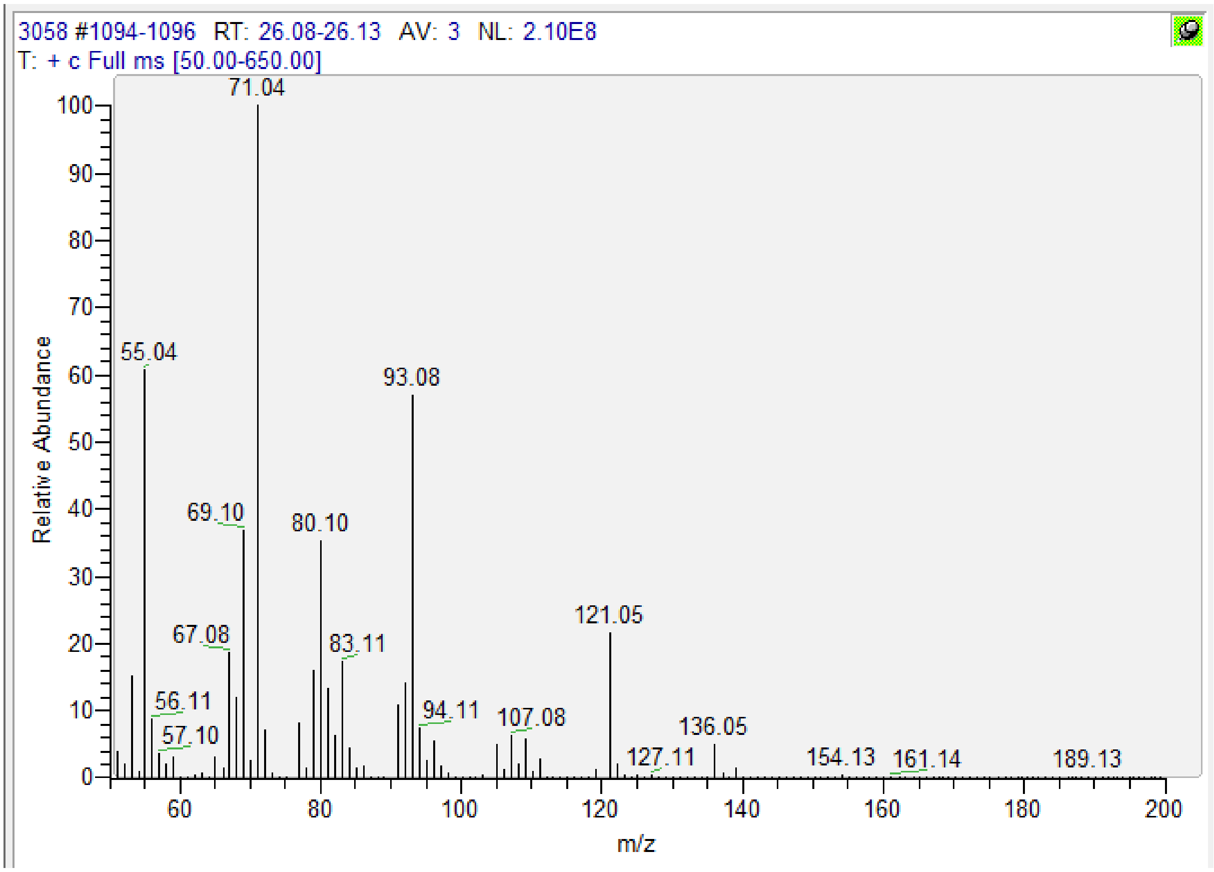
3058 #1094-1096 RT: 26.08-26.13 AV: 3 NL: 2.10E8
T: + c Full ms [50.00-650.00]
71.04
55.04
93.08
69.10
80.10
121.05
67.08
83.11
56.11
94.11
107.08
57.10
136.05
127.11
154.13
161.14
189.13
Relative Abundance
m/z

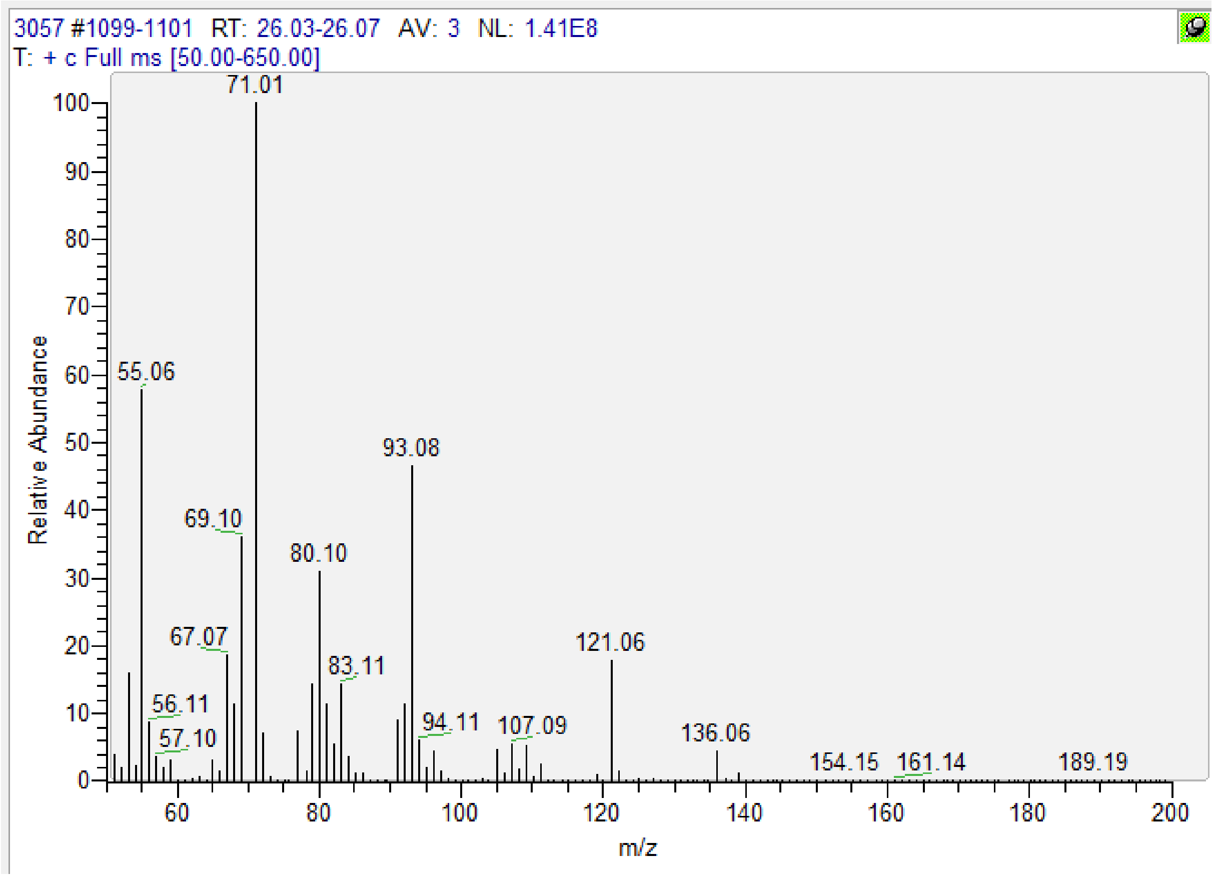
3057 #1099-1101 RT: 26.03-26.07 AV: 3 NL: 1.41E8
T: + c Full ms [50.00-650.00]
71.01
55.06
93.08
69.10
80.10
67.07
83.11
121.06
56.11
94.11
107.09
57.10
136.06
154.15
161.14
189.19
Relative Abundance
m/z

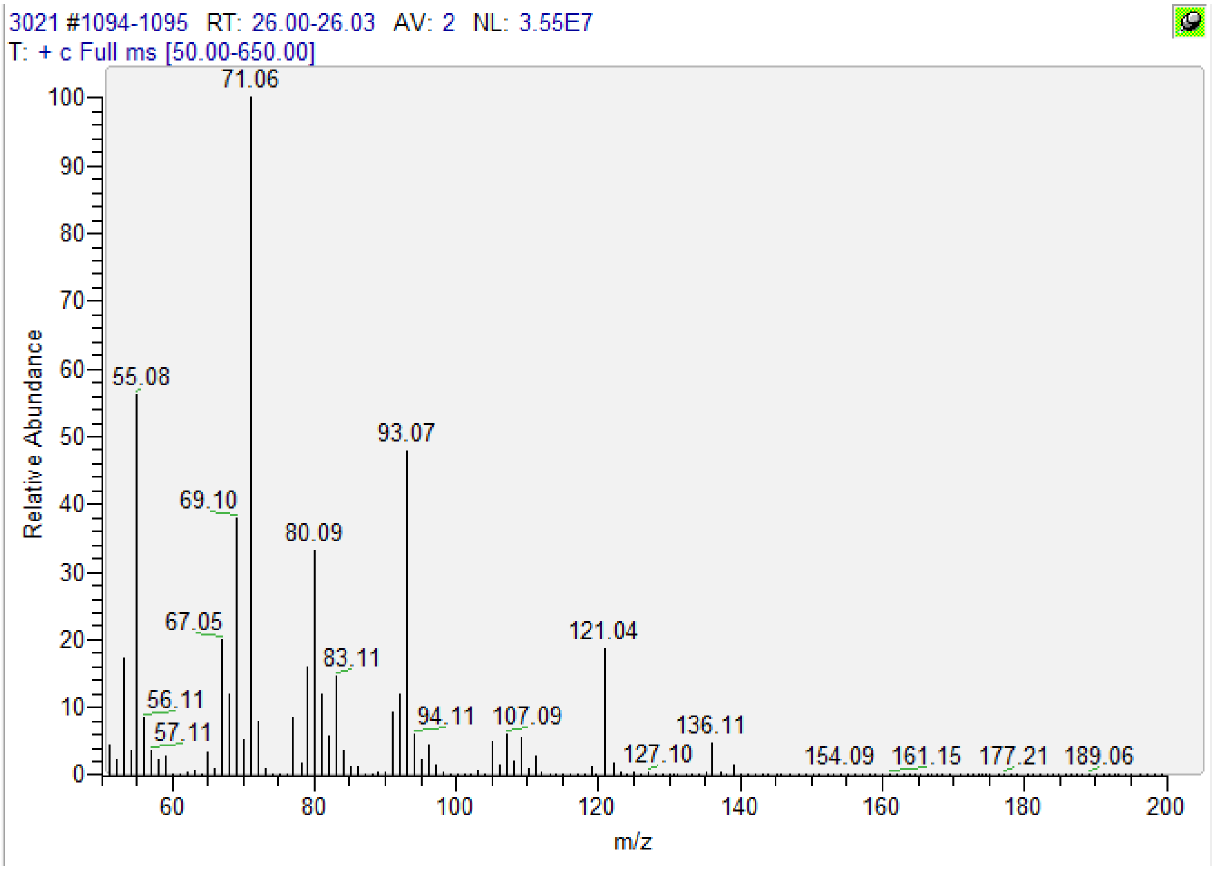

3021 #1094-1095 RT: 26.00-26.03 AV: 2 NL: 3.55E7
T: + c Full ms [50.00-650.00]
Relative Abundance
m/z
71.06
55.08
93.07
69.10
80.09
67.05
121.04
83.11
56.11
57.11
94.11
107.09
136.11
127.10
154.09
161.15
177.21
189.06

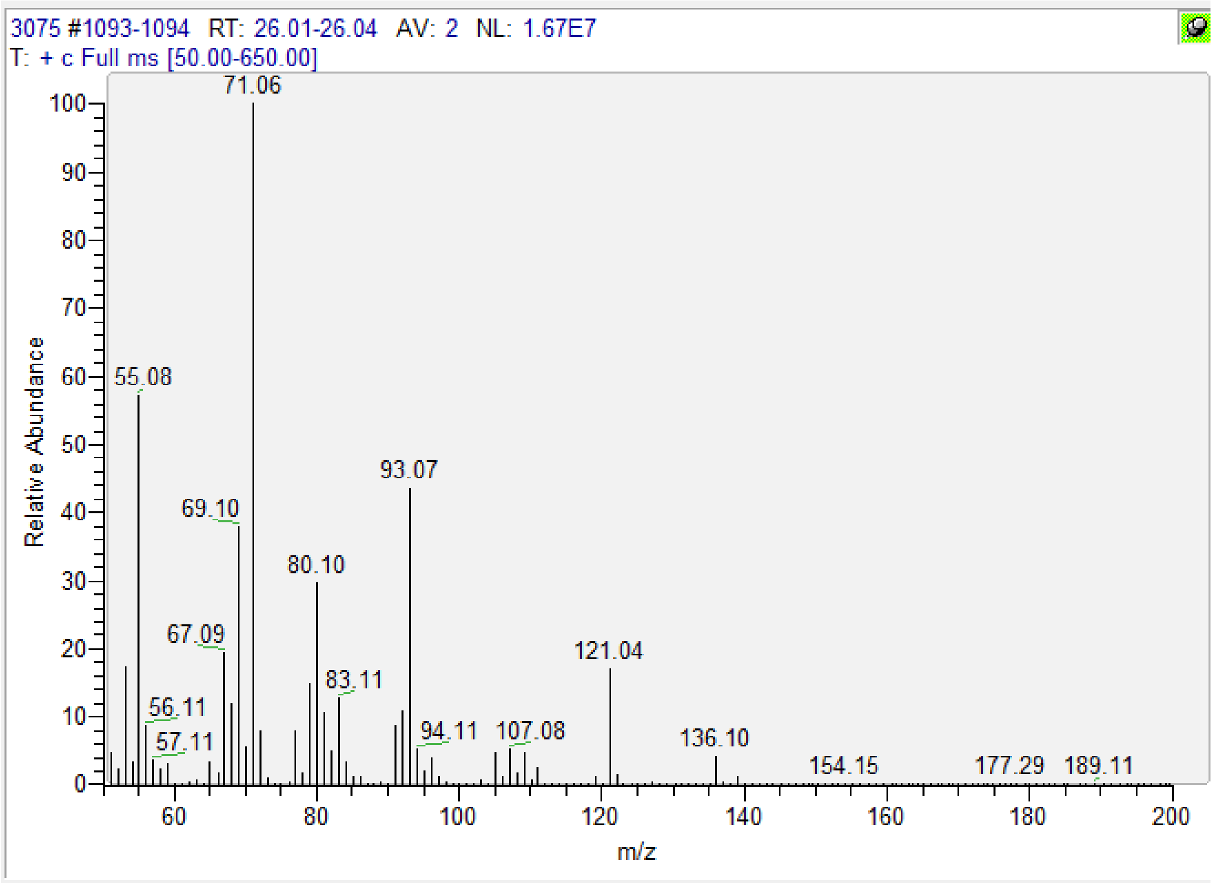

3075 #1093-1094 RT: 26.01-26.04 AV: 2 NL: 1.67E7
T: + c Full ms [50.00-650.00]
Relative Abundance
m/z
71.06
55.08
93.07
69.10
80.10
67.09
121.04
83.11
56.11
57.11
94.11
107.08
136.10
154.15
177.29
189.11

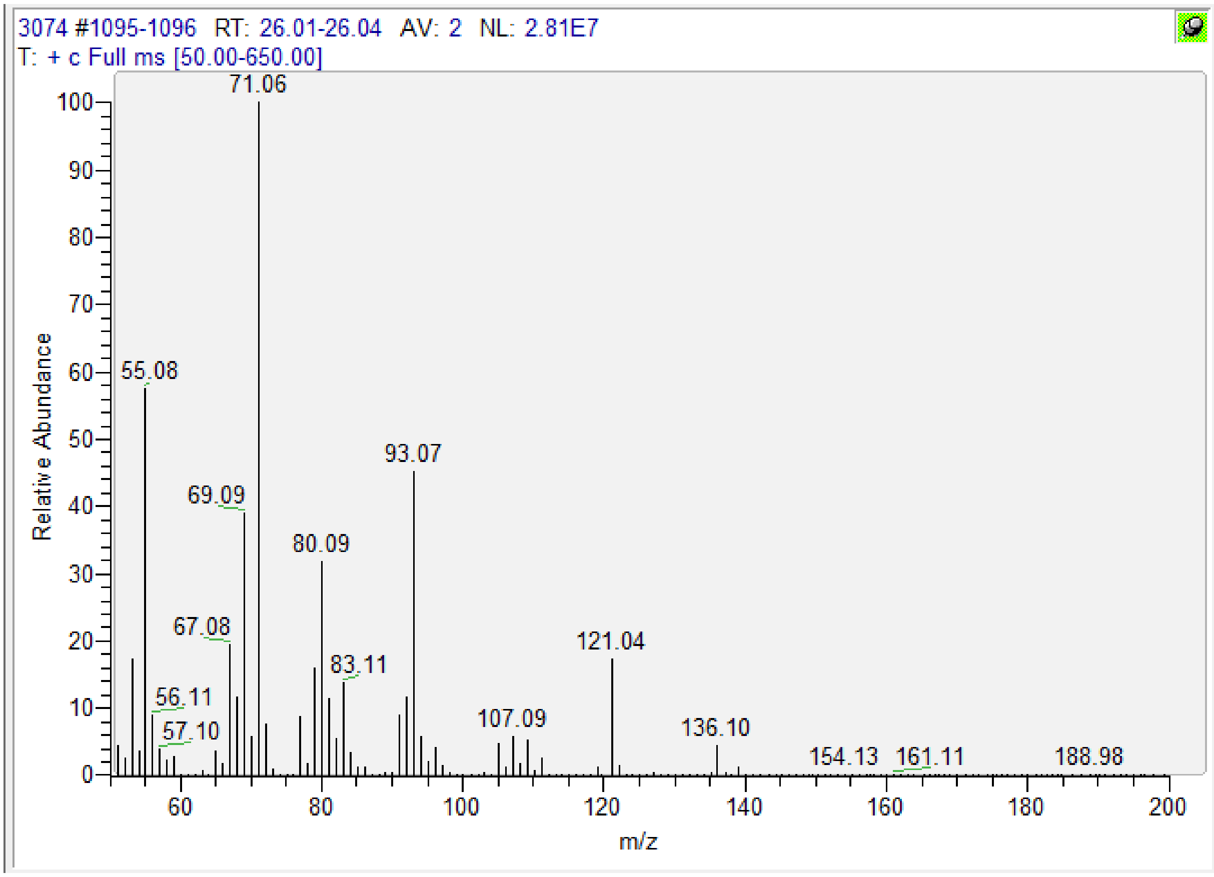

3074 #1095-1096 RT: 26.01-26.04 AV: 2 NL: 2.81E7
T: + c Full ms [50.00-650.00]
Relative Abundance
m/z
71.06
55.08
93.07
69.09
80.09
67.08
121.04
83.11
56.11
57.10
107.09
136.10
154.13
161.11
188.98

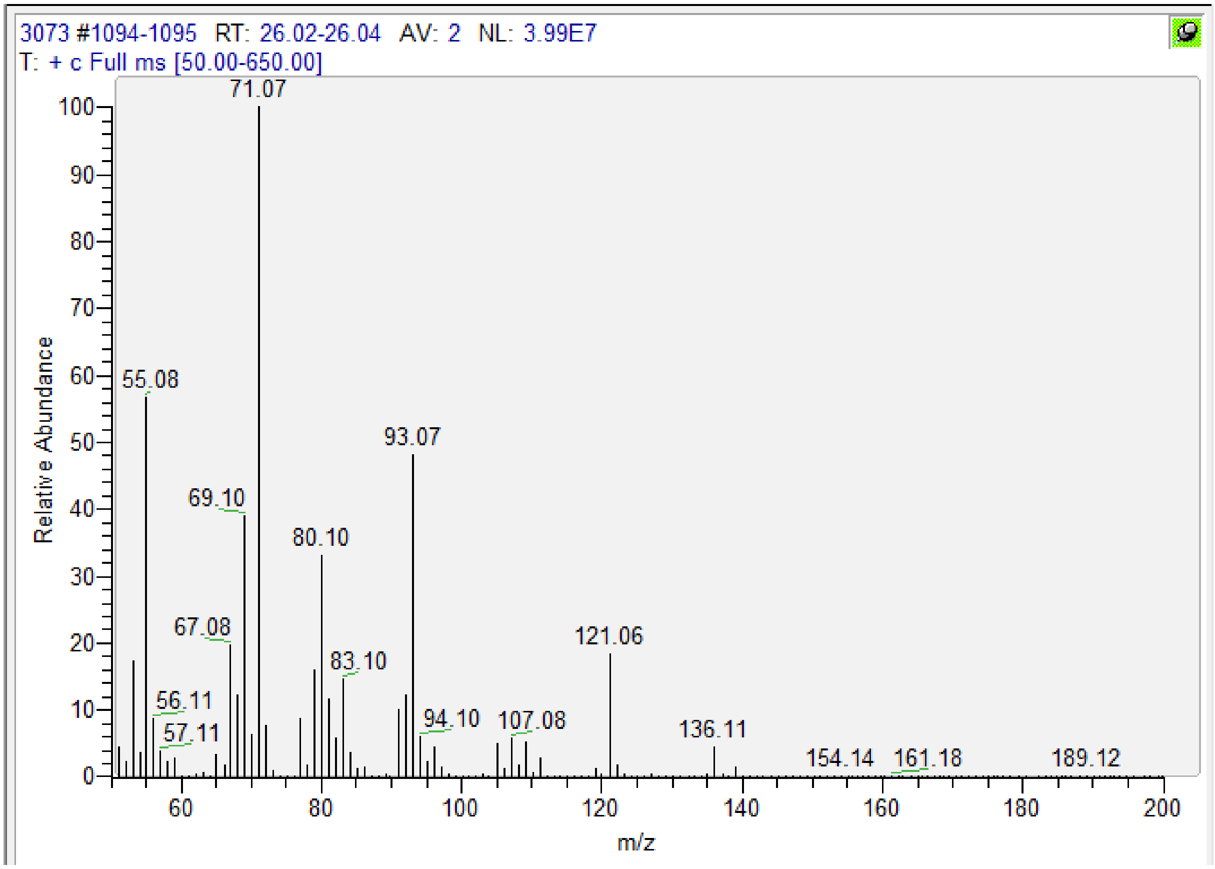

3073 #1094-1095 RT: 26.02-26.04 AV: 2 NL: 3.99E7
T: + c Full ms [50.00-650.00]
Relative Abundance
m/z
71.07
55.08
93.07
69.10
80.10
67.08
83.10
121.06
56.11
57.11
94.10
107.08
136.11
154.14
161.18
189.12

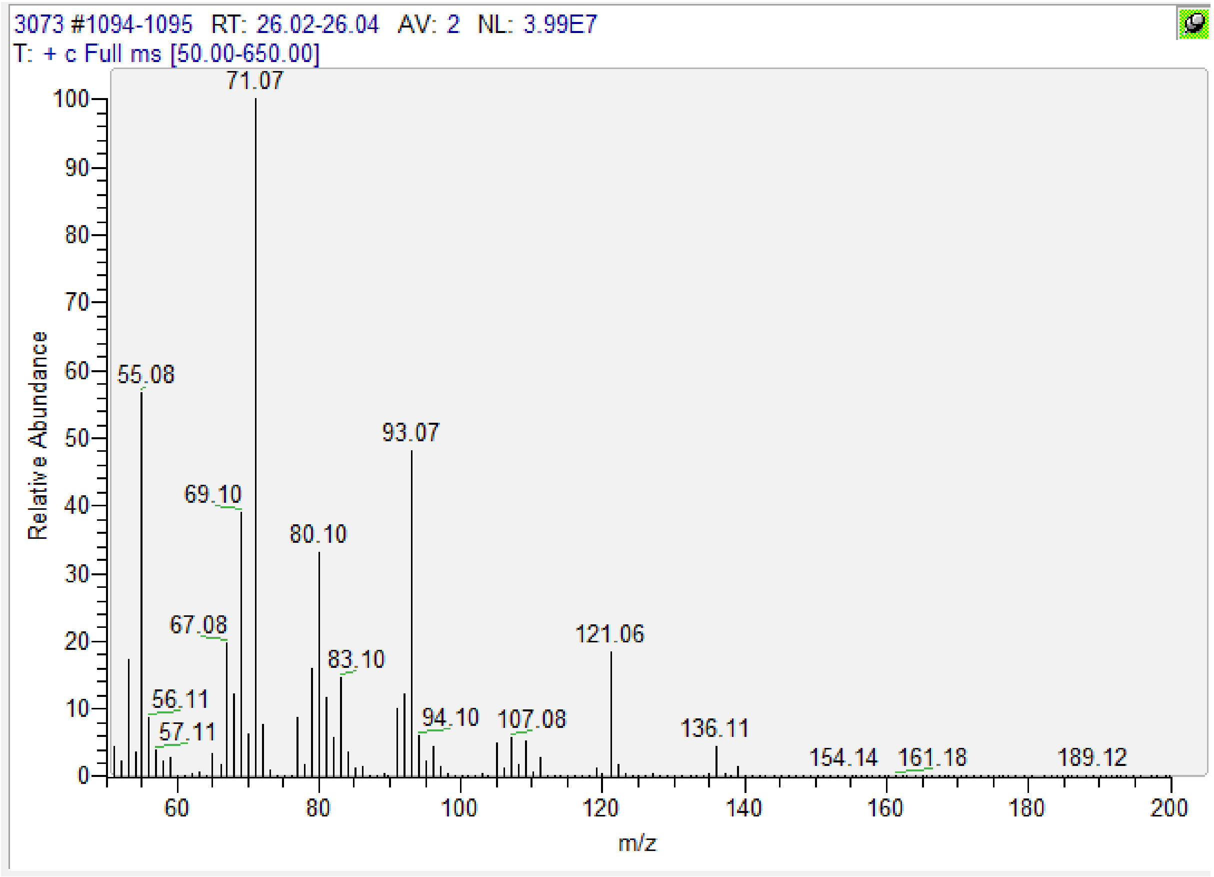
3073 #1094-1095 RT: 26.02-26.04 AV: 2 NL: 3.99E7
T: + c Full ms [50.00-650.00]
Relative Abundance
m/z
71.07
55.08
93.07
69.10
80.10
67.08
121.06
83.10
56.11
57.11
94.10
107.08
136.11
154.14
161.18
189.12

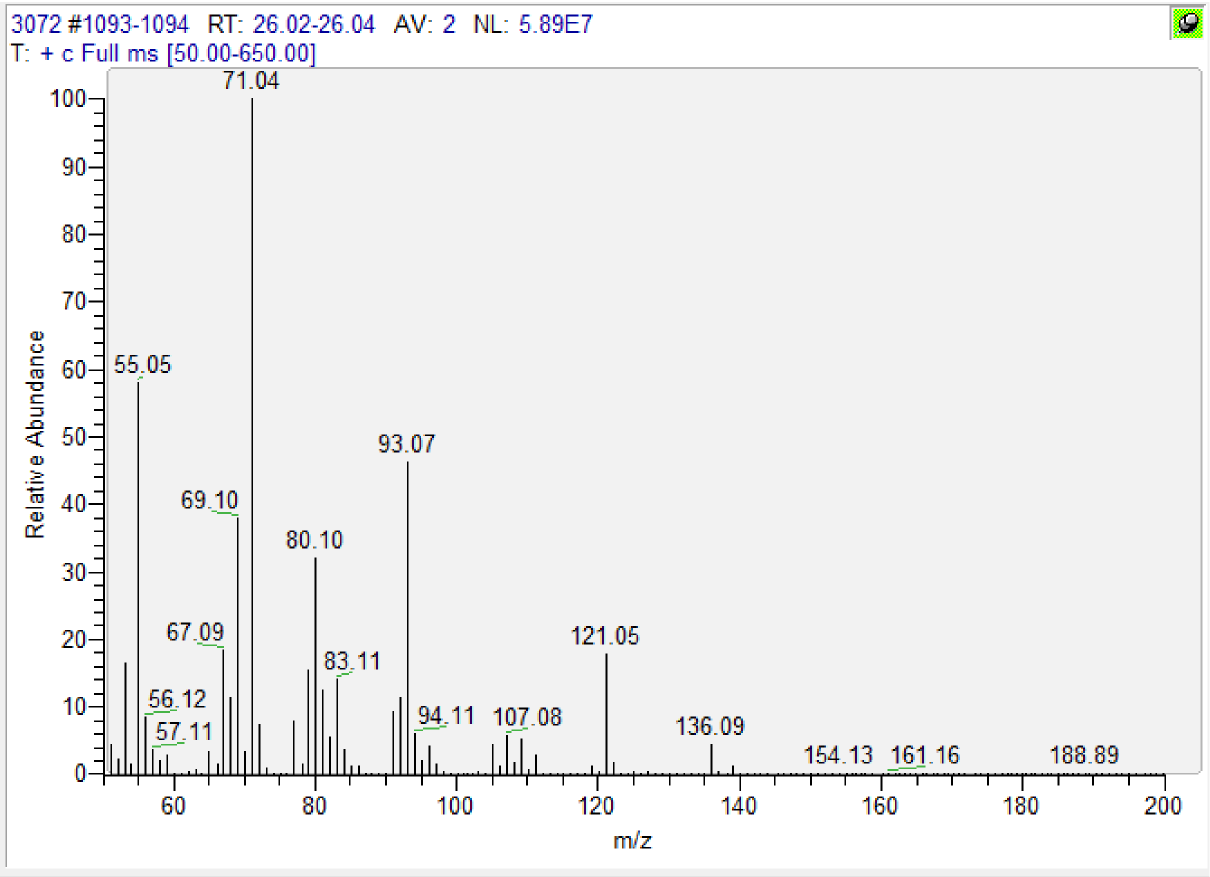
3072 #1093-1094 RT: 26.02-26.04 AV: 2 NL: 5.89E7
T: + c Full ms [50.00-650.00]
Relative Abundance
m/z
71.04
55.05
93.07
69.10
80.10
67.09
121.05
83.11
56.12
57.11
94.11
107.08
136.09
154.13
161.16
188.89

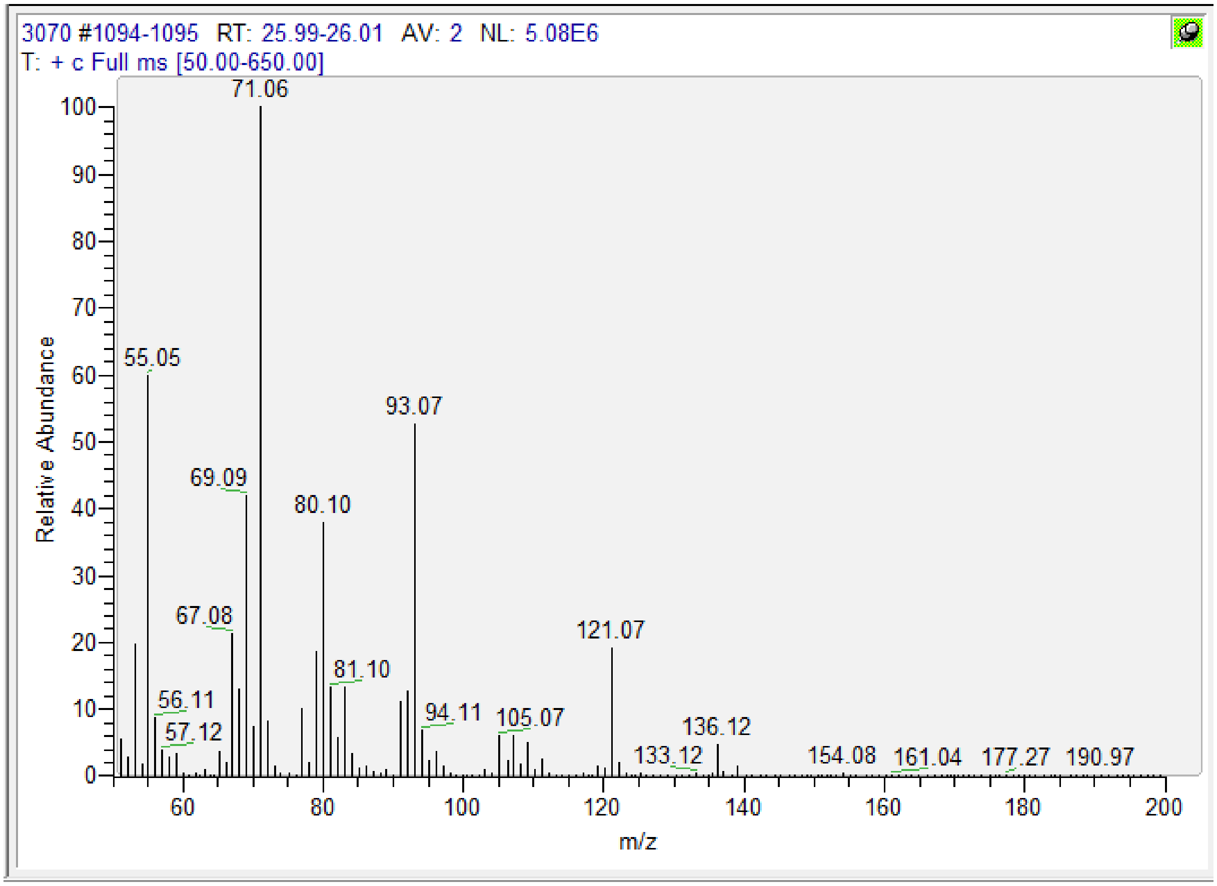
3070 #1094-1095 RT: 25.99-26.01 AV: 2 NL: 5.08E6
T: + c Full ms [50.00-650.00]
Relative Abundance
m/z
71.06
55.05
93.07
69.09
80.10
67.08
121.07
81.10
56.11
57.12
94.11
105.07
136.12
133.12
154.08
161.04
177.27
190.97

Beta Caryophyllene

Reference:

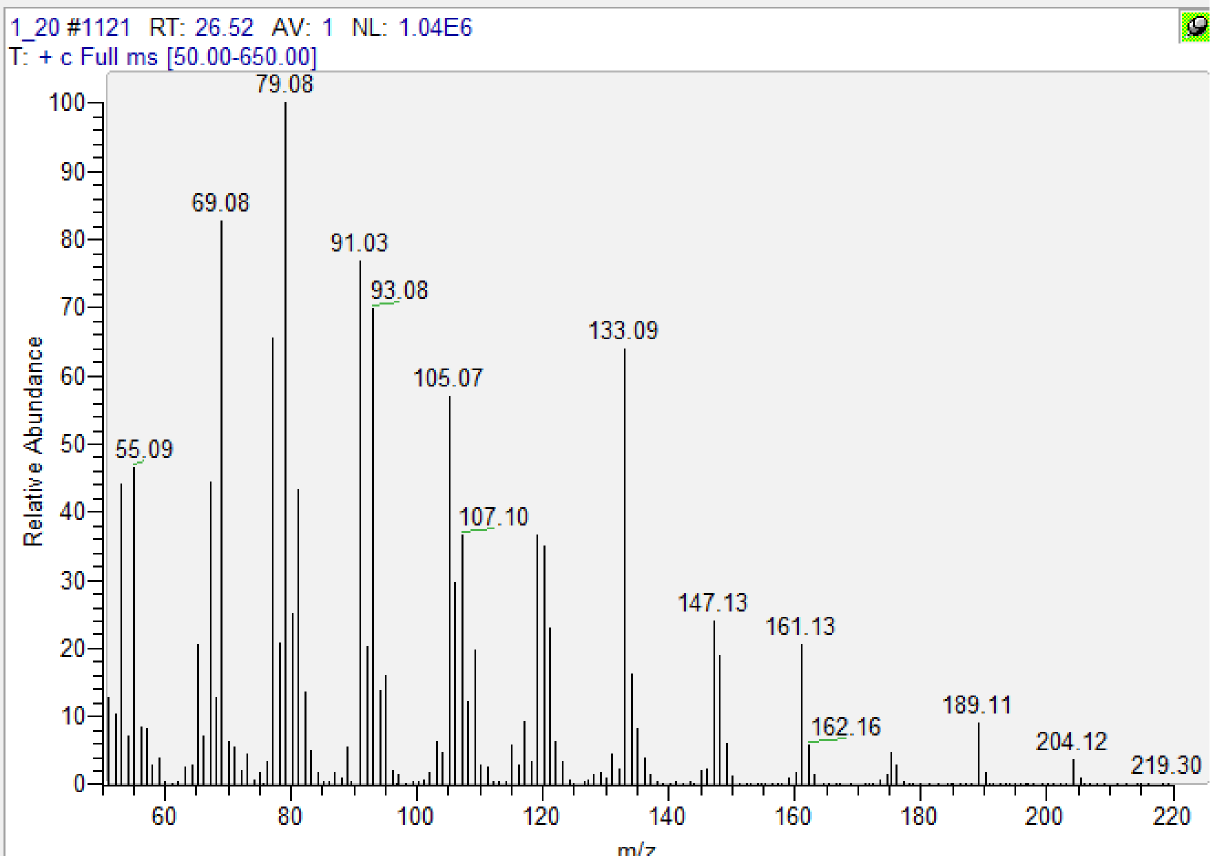

Samples:

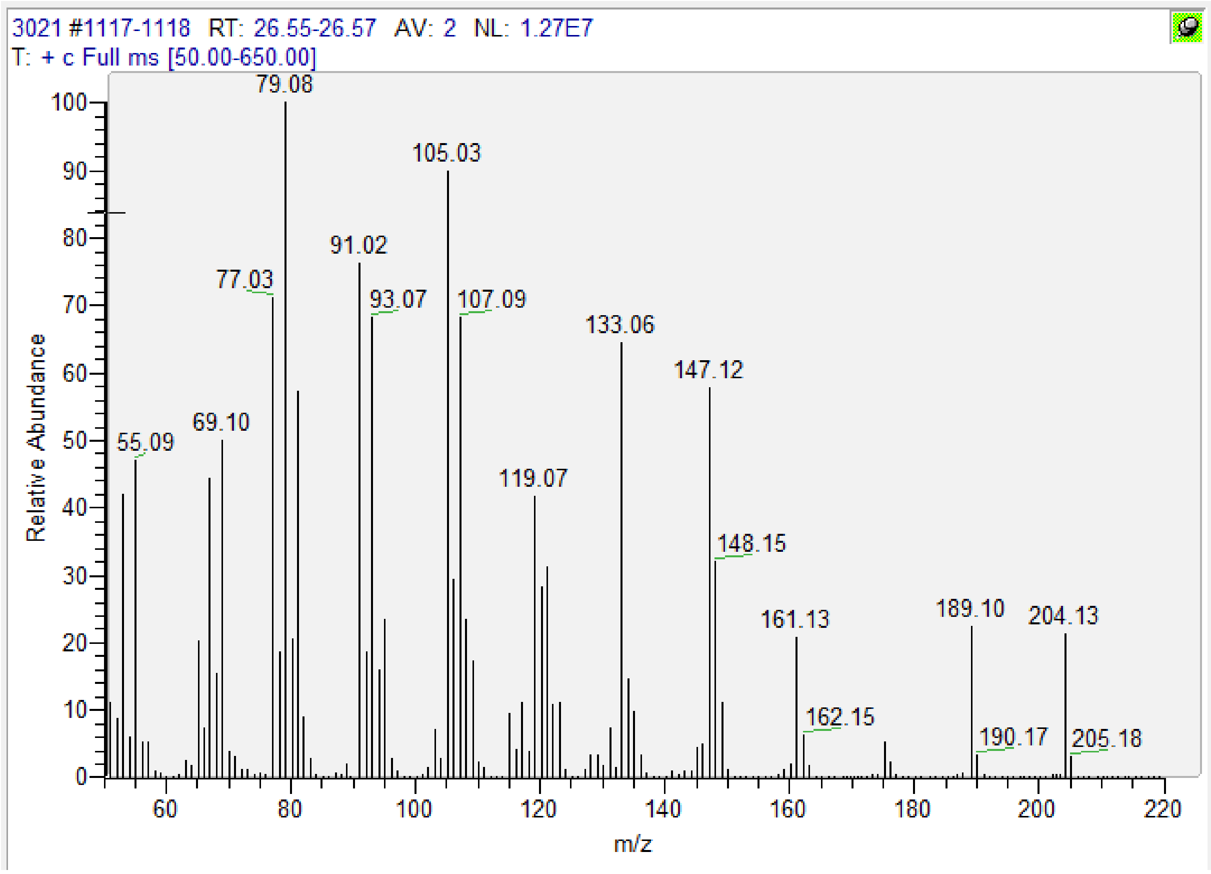

3021 #1117-1118 RT: 26.55-26.57 AV: 2 NL: 1.27E7
T: + c Full ms [50.00-650.00]
Relative Abundance
m/z
79.08
105.03
91.02
77.03
93.07
107.09
133.06
147.12
69.10
55.09
119.07
148.15
161.13
189.10
204.13
162.15
190.17
205.18

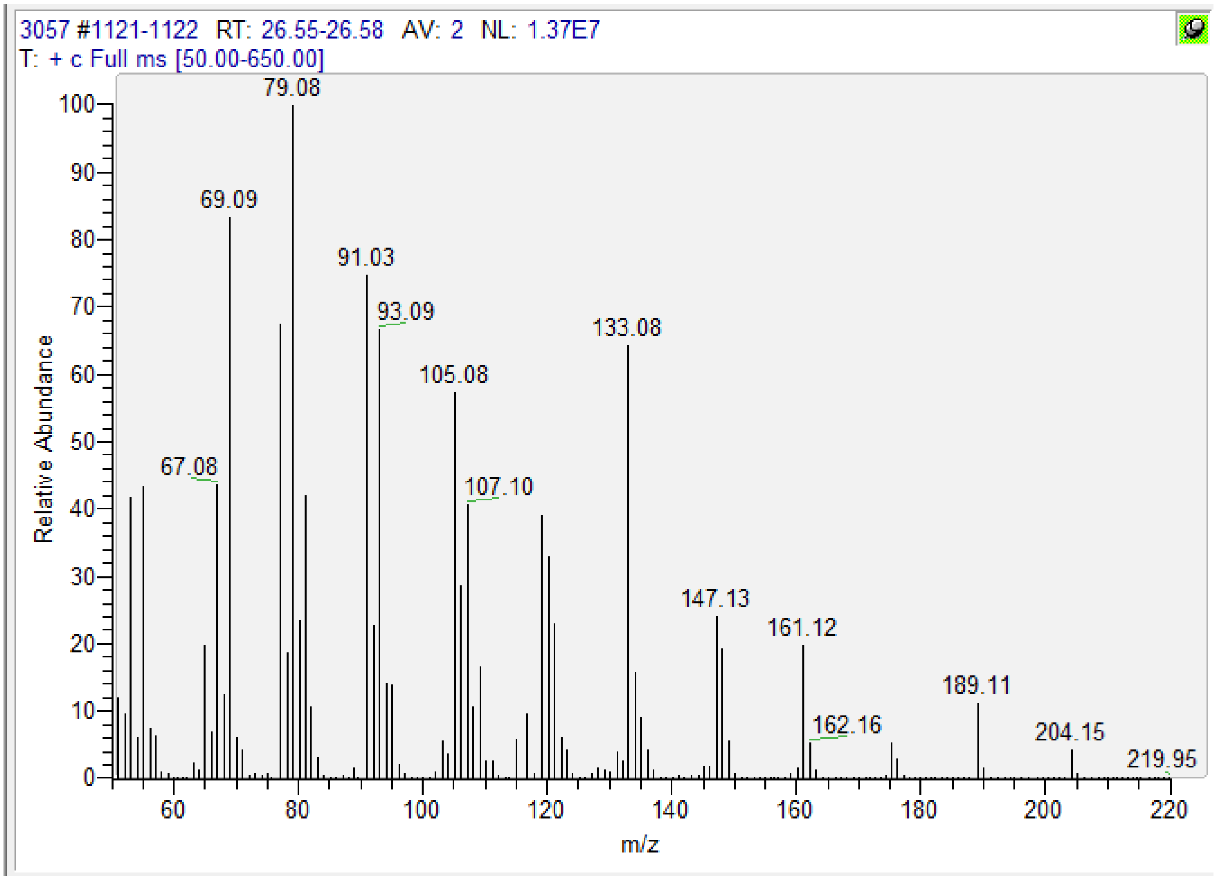

3057 #1121-1122 RT: 26.55-26.58 AV: 2 NL: 1.37E7
T: + c Full ms [50.00-650.00]
Relative Abundance
m/z
79.08
69.09
91.03
93.09
133.08
105.08
67.08
107.10
147.13
161.12
189.11
162.16
204.15
219.95

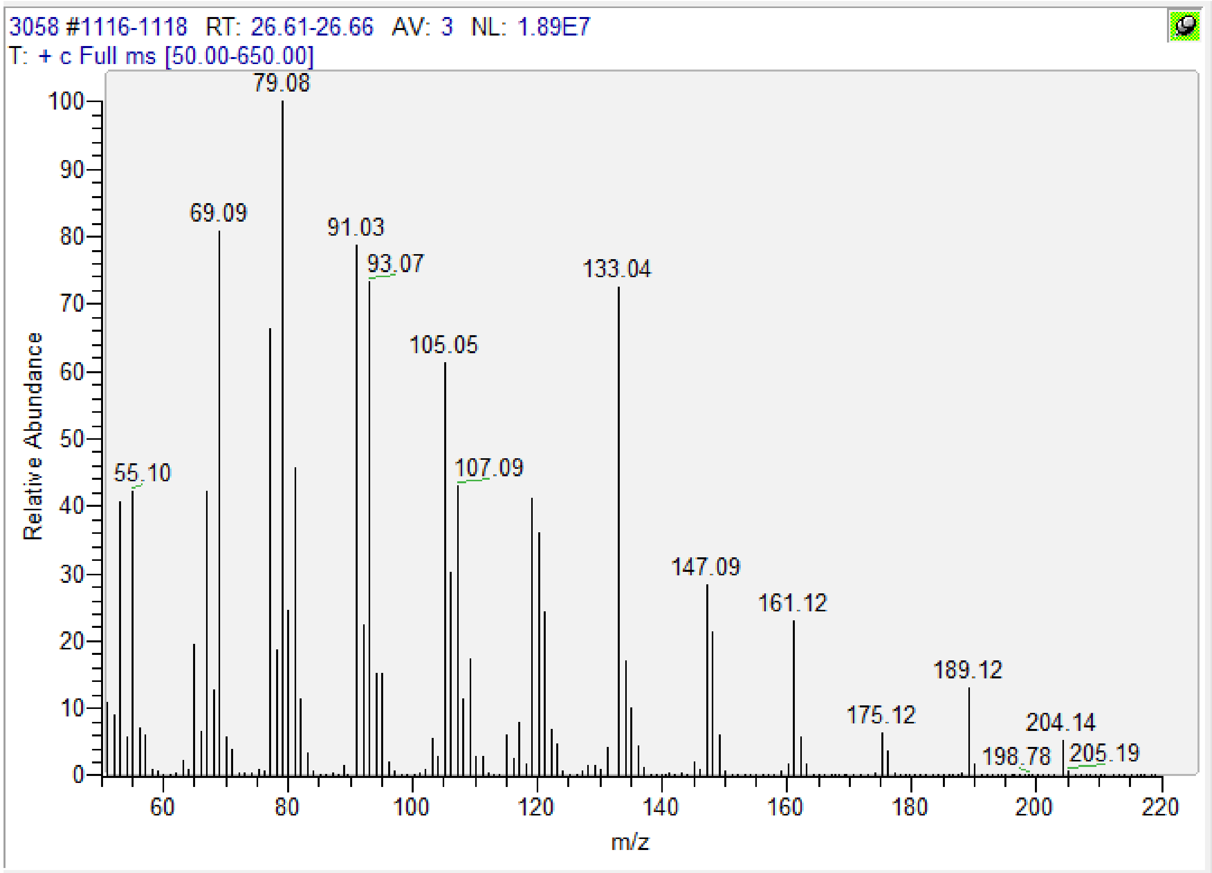

3058 #1116-1118 RT: 26.61-26.66 AV: 3 NL: 1.89E7
T: + c Full ms [50.00-650.00]
Relative Abundance
m/z
79.08
69.09
91.03
93.07
133.04
105.05
55.10
107.09
147.09
161.12
189.12
175.12
204.14
198.78
205.19

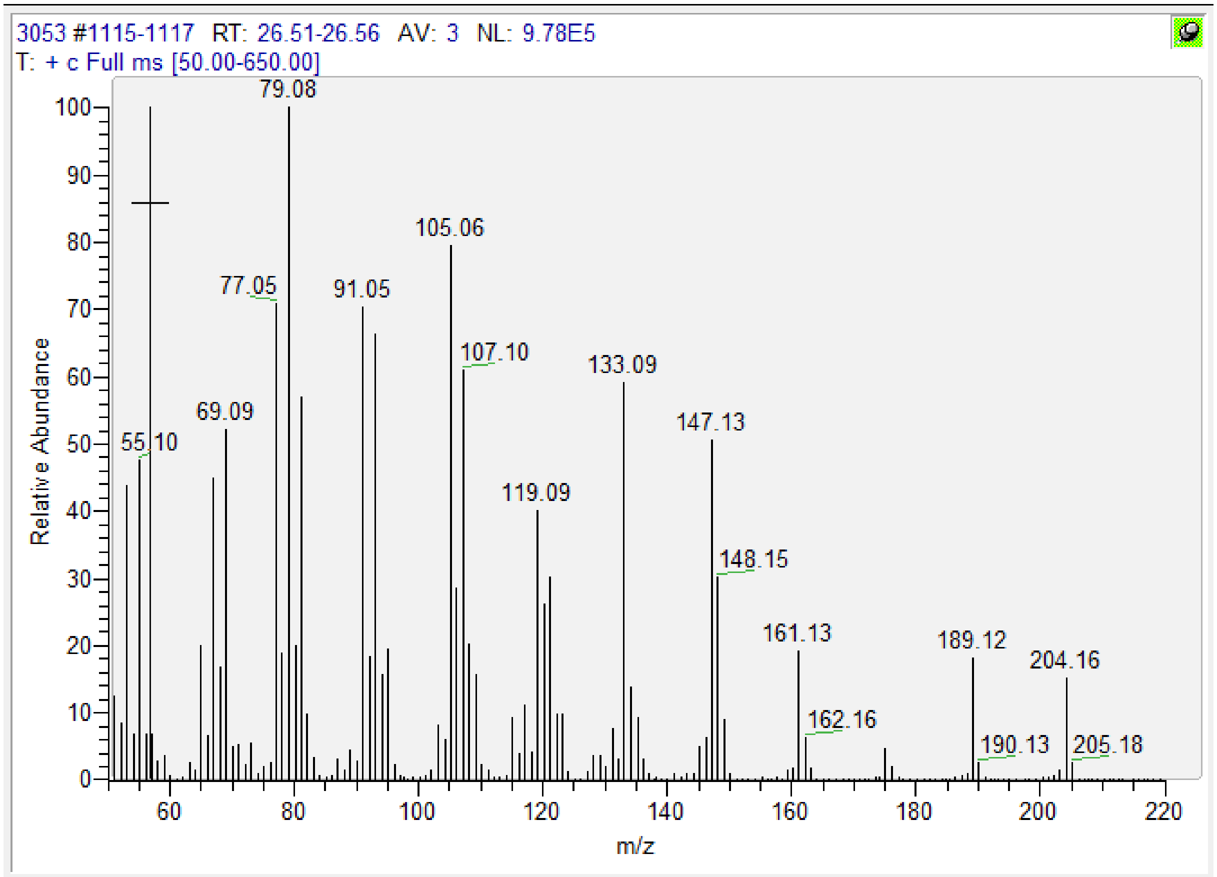
3053 #1115-1117 RT: 26.51-26.56 AV: 3 NL: 9.78E5
T: + c Full ms [50.00-650.00]
Relative Abundance
m/z
79.08
105.06
77.05
91.05
107.10
133.09
69.09
55.10
147.13
119.09
148.15
161.13
189.12
204.16
162.16
190.13
205.18

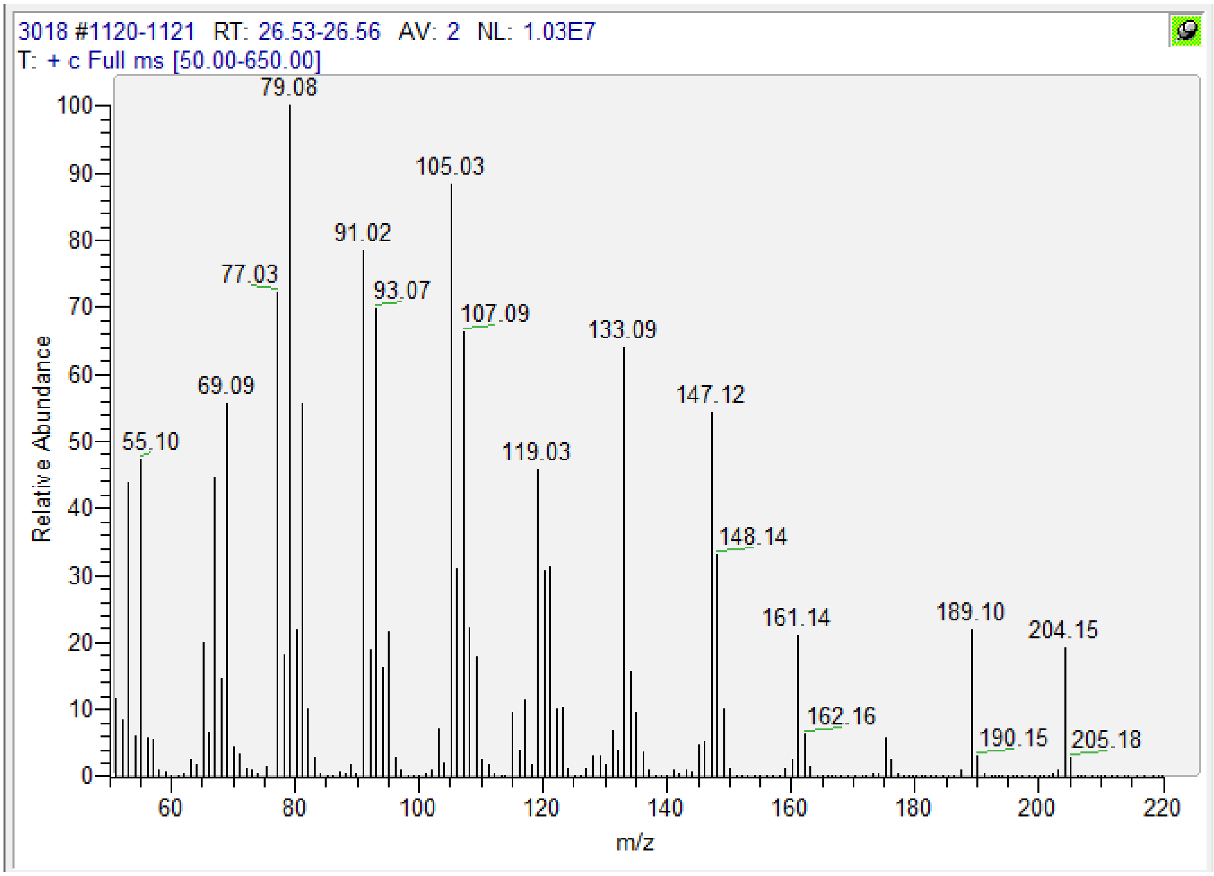
3018 #1120-1121 RT: 26.53-26.56 AV: 2 NL: 1.03E7
T: + c Full ms [50.00-650.00]
Relative Abundance
m/z
79.08
105.03
91.02
77.03
93.07
107.09
133.09
69.09
147.12
55.10
119.03
148.14
161.14
189.10
204.15
162.16
190.15
205.18
0
10
20
30
40
50
60
70
80
90
100
60
80
100
120
140
160
180
200
220

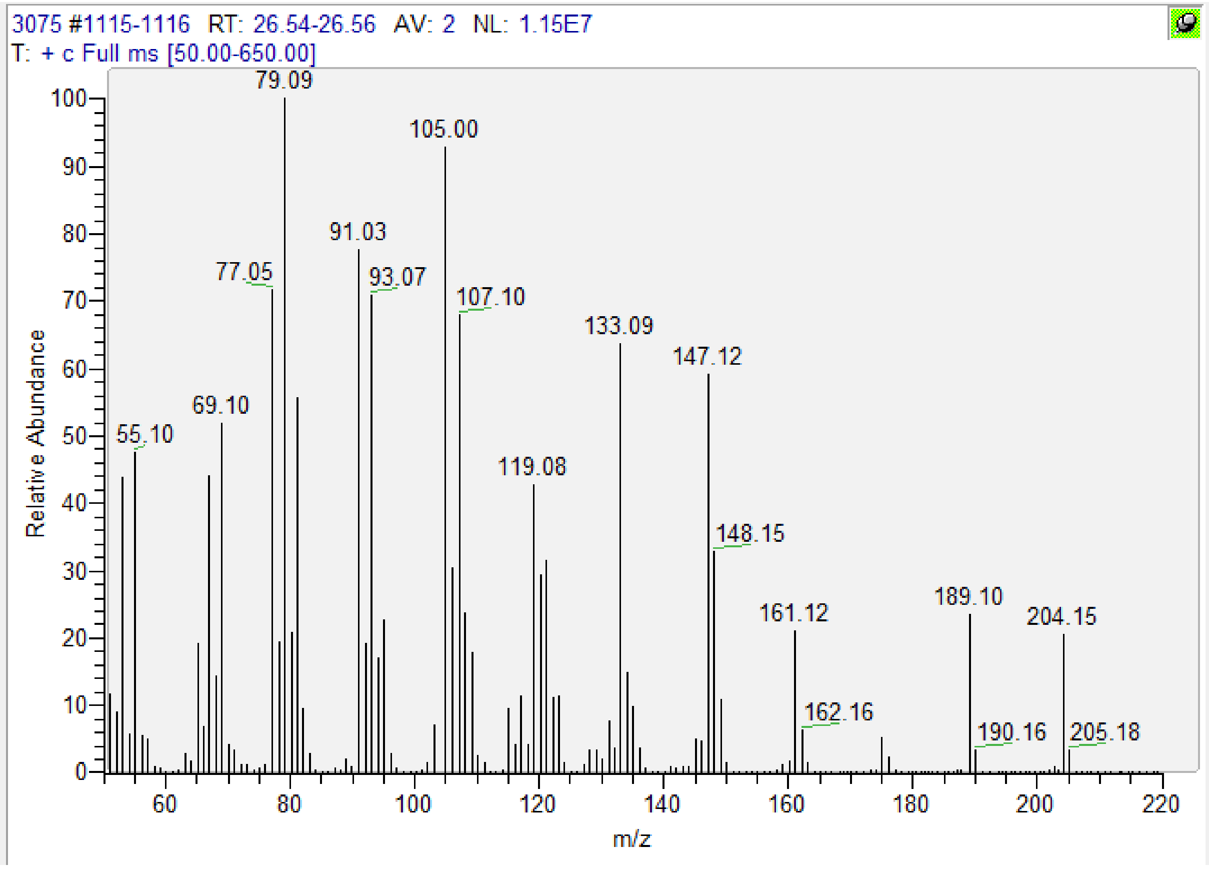

3075 #1115-1116 RT: 26.54-26.56 AV: 2 NL: 1.15E7
T: + c Full ms [50.00-650.00]
Relative Abundance
m/z
79.09
105.00
91.03
77.05
93.07
107.10
133.09
147.12
69.10
55.10
119.08
148.15
161.12
189.10
204.15
162.16
190.16
205.18

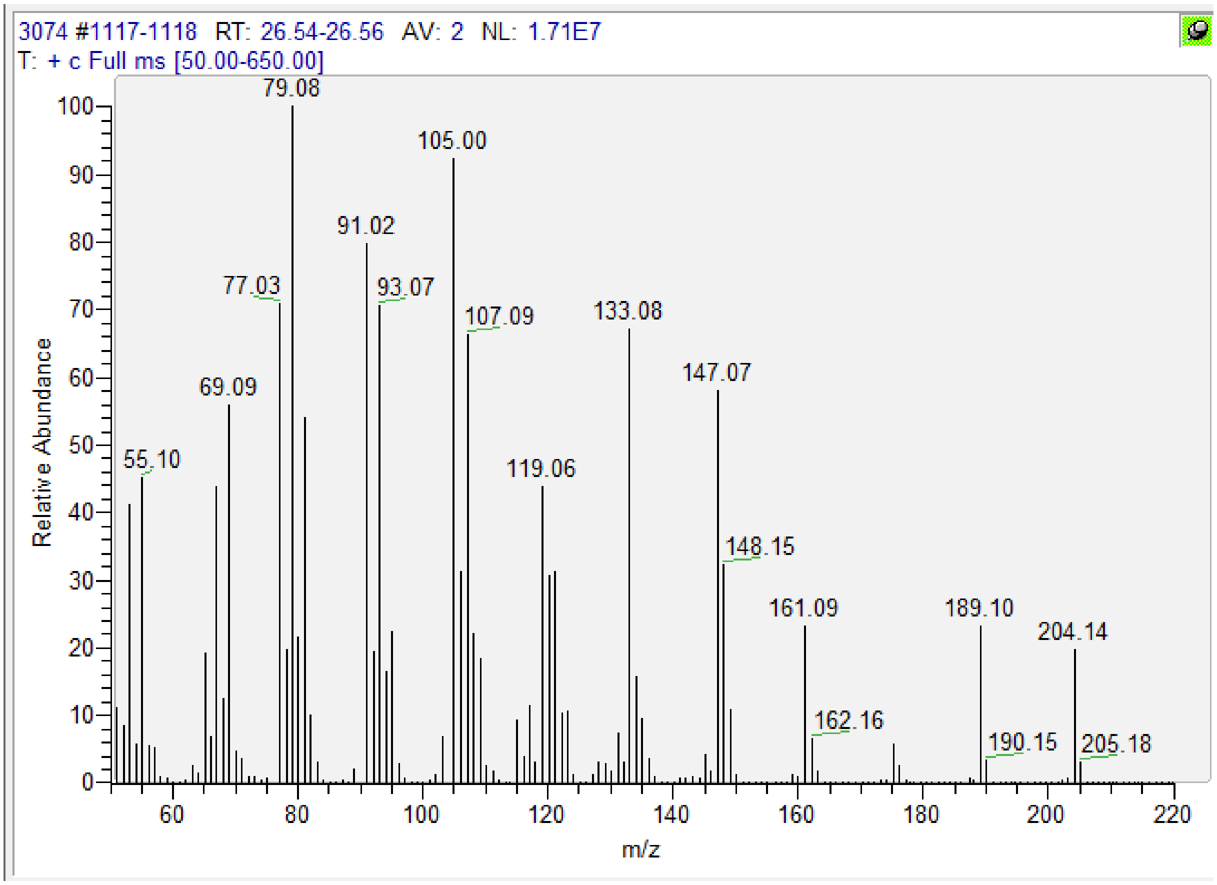

3074 #1117-1118 RT: 26.54-26.56 AV: 2 NL: 1.71E7
T: + c Full ms [50.00-650.00]
Relative Abundance
m/z
79.08
105.00
91.02
77.03
93.07
107.09
133.08
147.07
69.09
55.10
119.06
148.15
161.09
189.10
204.14
162.16
190.15
205.18

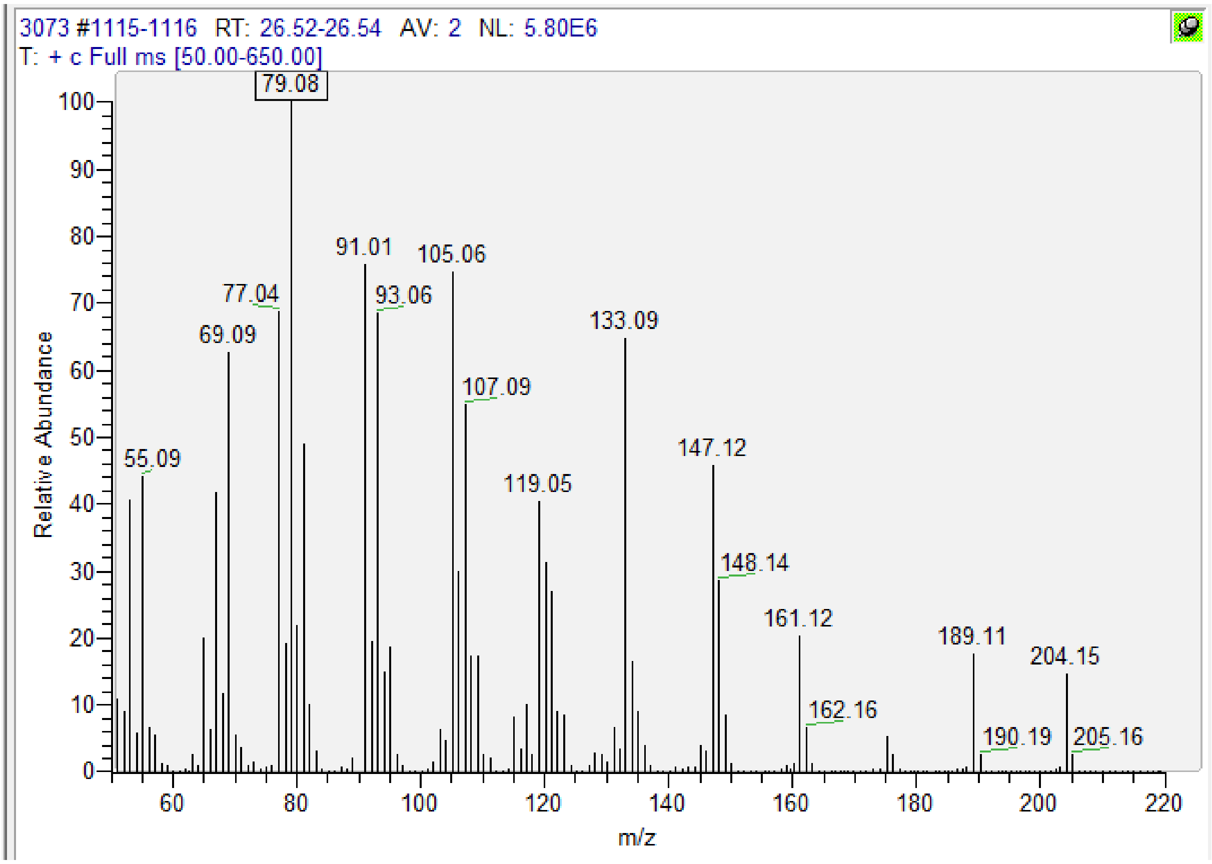
3073 #1115-1116 RT: 26.52-26.54 AV: 2 NL: 5.80E6
T: + c Full ms [50.00-650.00]
79.08
91.01
105.06
77.04
93.06
69.09
133.09
107.09
55.09
147.12
119.05
148.14
161.12
189.11
204.15
162.16
190.19
205.16
Relative Abundance
m/z

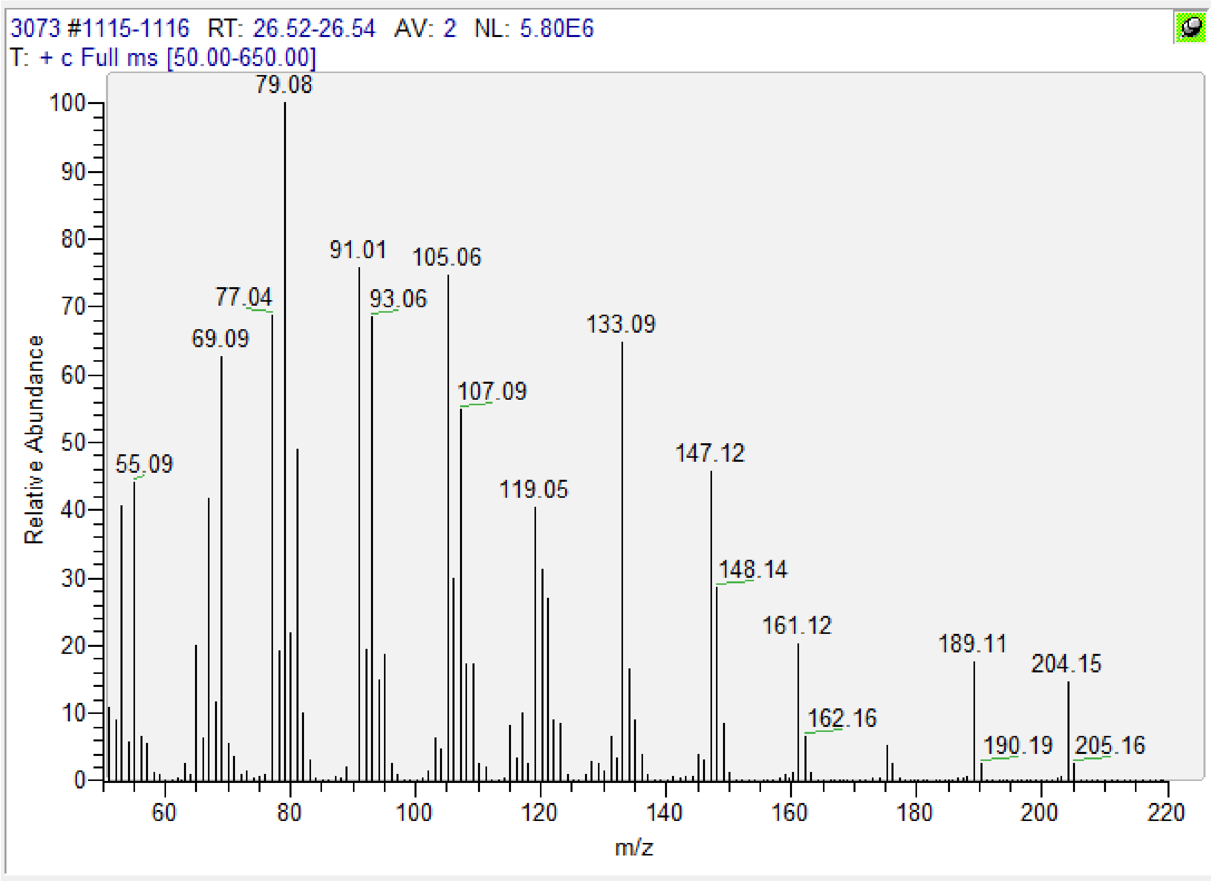
3073 #1115-1116 RT: 26.52-26.54 AV: 2 NL: 5.80E6
T: + c Full ms [50.00-650.00]
79.08
91.01
105.06
77.04
93.06
133.09
69.09
107.09
55.09
147.12
119.05
148.14
161.12
189.11
204.15
162.16
190.19
205.16
Relative Abundance
m/z

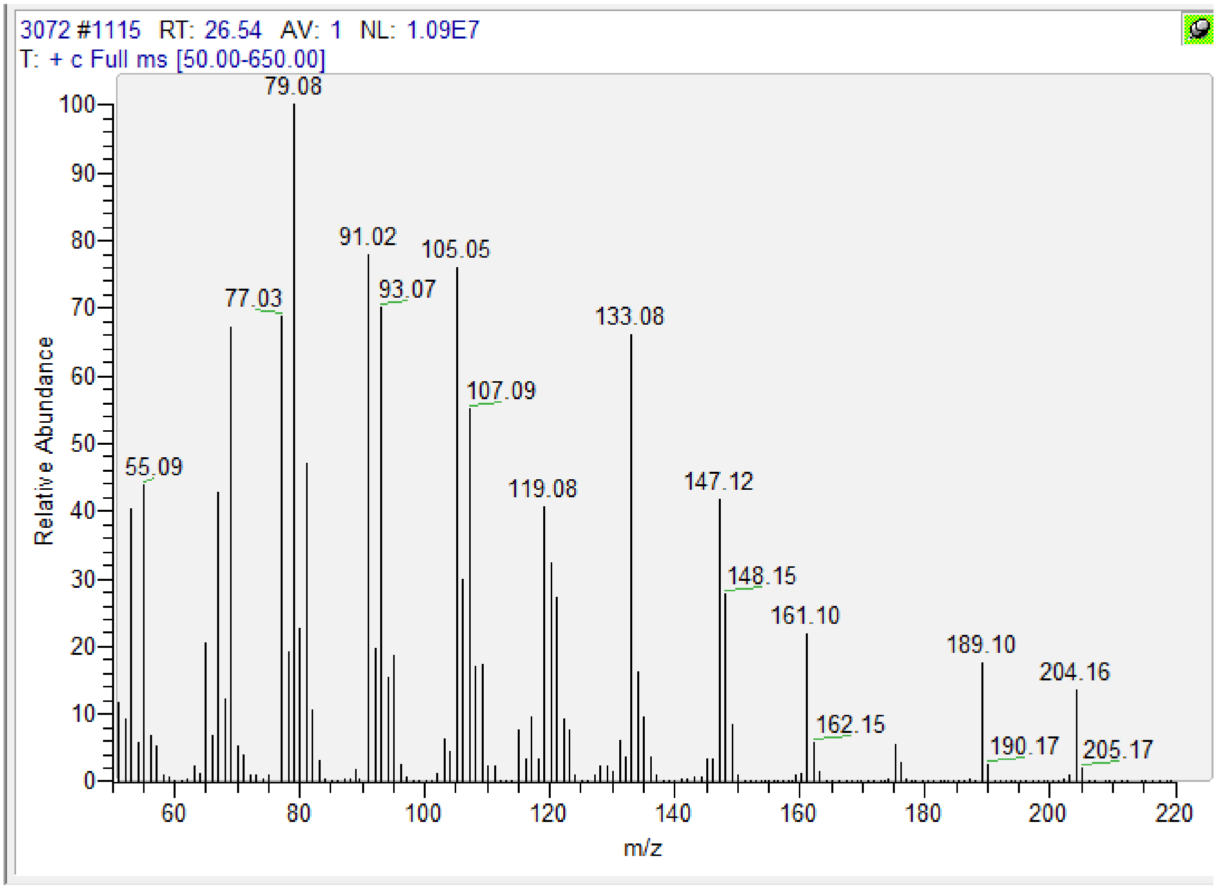
3072 #1115 RT: 26.54 AV: 1 NL: 1.09E7
T: + c Full ms [50.00-650.00]
Relative Abundance
m/z
79.08
91.02
105.05
77.03
93.07
133.08
107.09
55.09
119.08
147.12
148.15
161.10
189.10
204.16
162.15
190.17
205.17

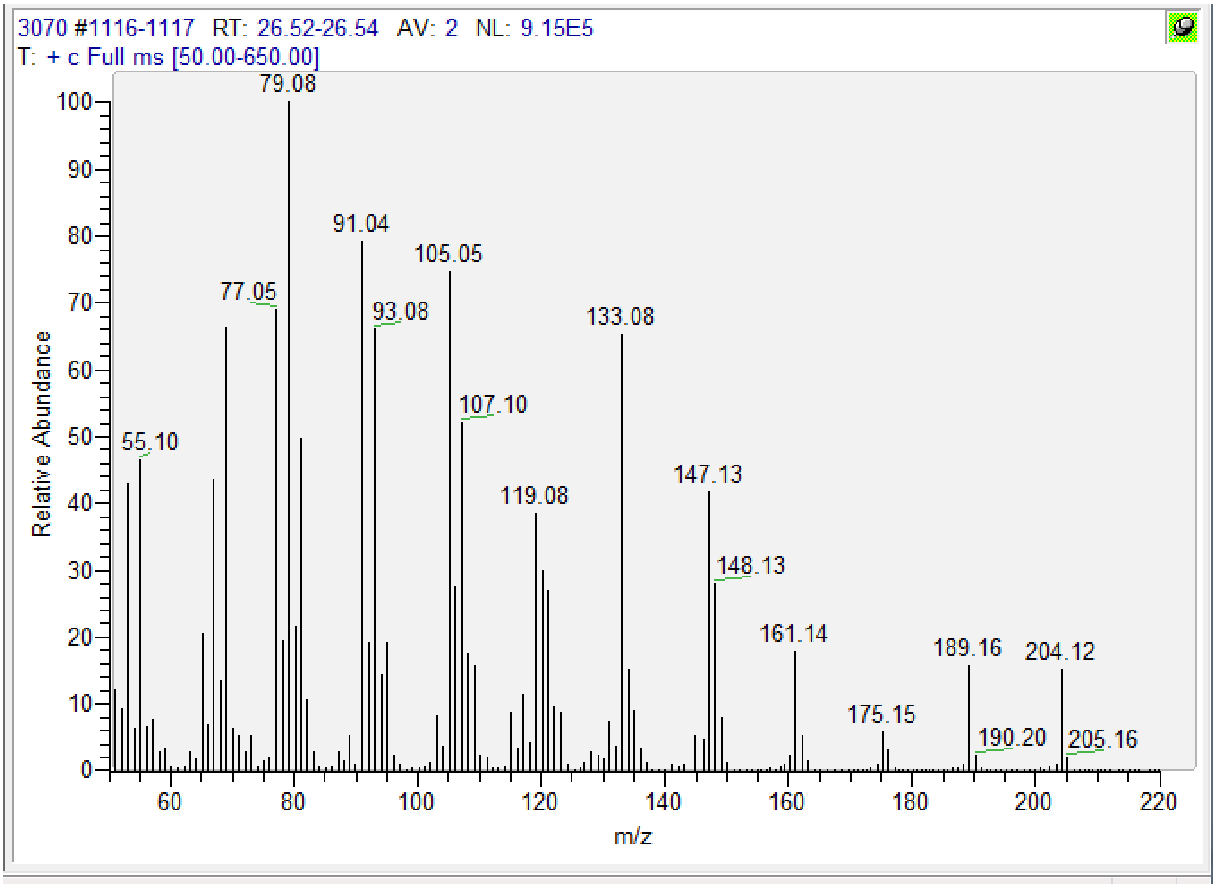
3070 #1116-1117 RT: 26.52-26.54 AV: 2 NL: 9.15E5
T: + c Full ms [50.00-650.00]
79.08
91.04
105.05
77.05
93.08
133.08
107.10
55.10
147.13
119.08
148.13
161.14
189.16
204.12
175.15
190.20
205.16
Relative Abundance
m/z
0
10
20
30
40
50
60
70
80
90
100
60
80
100
120
140
160
180
200
220

Beta damascenone:

Reference:

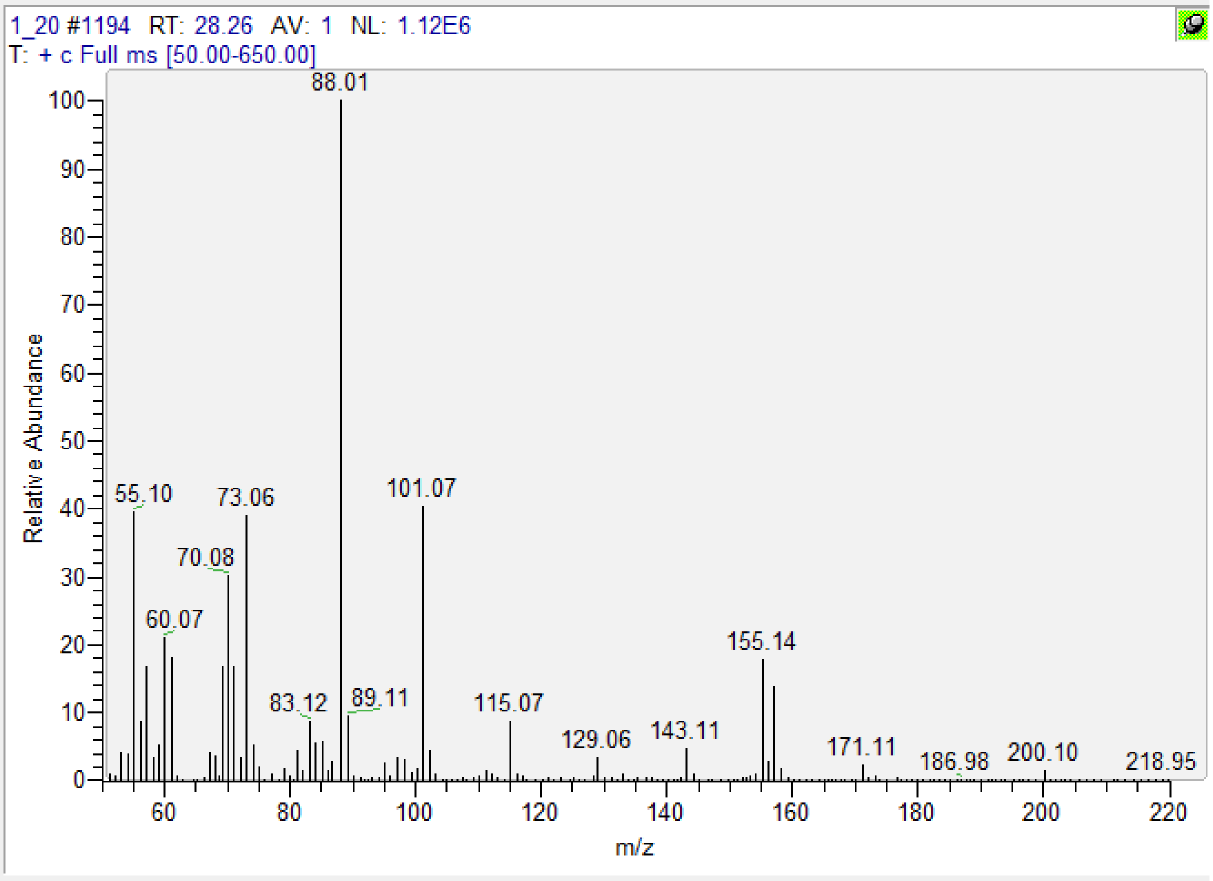

Samples:

Not detected in any samples.

Citronellyl acetate

Reference

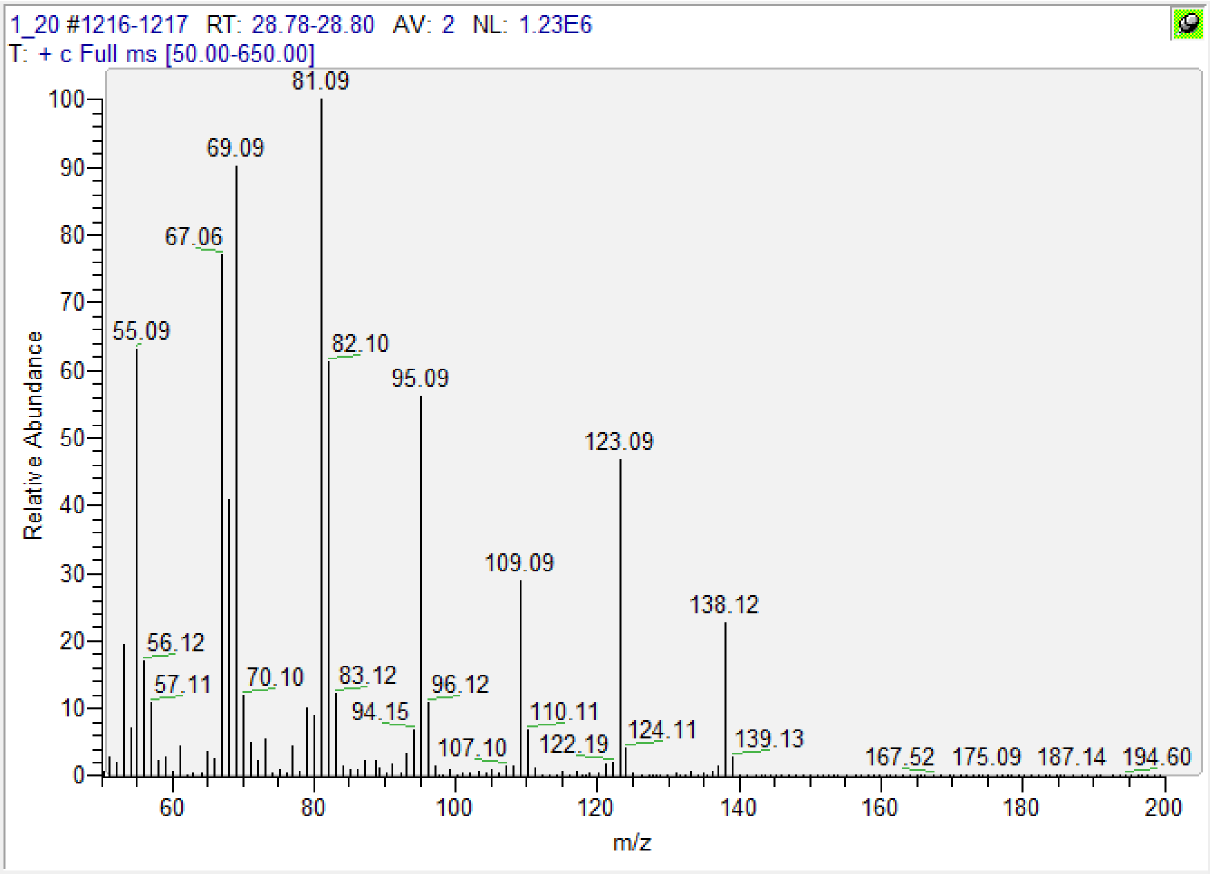

Samples:

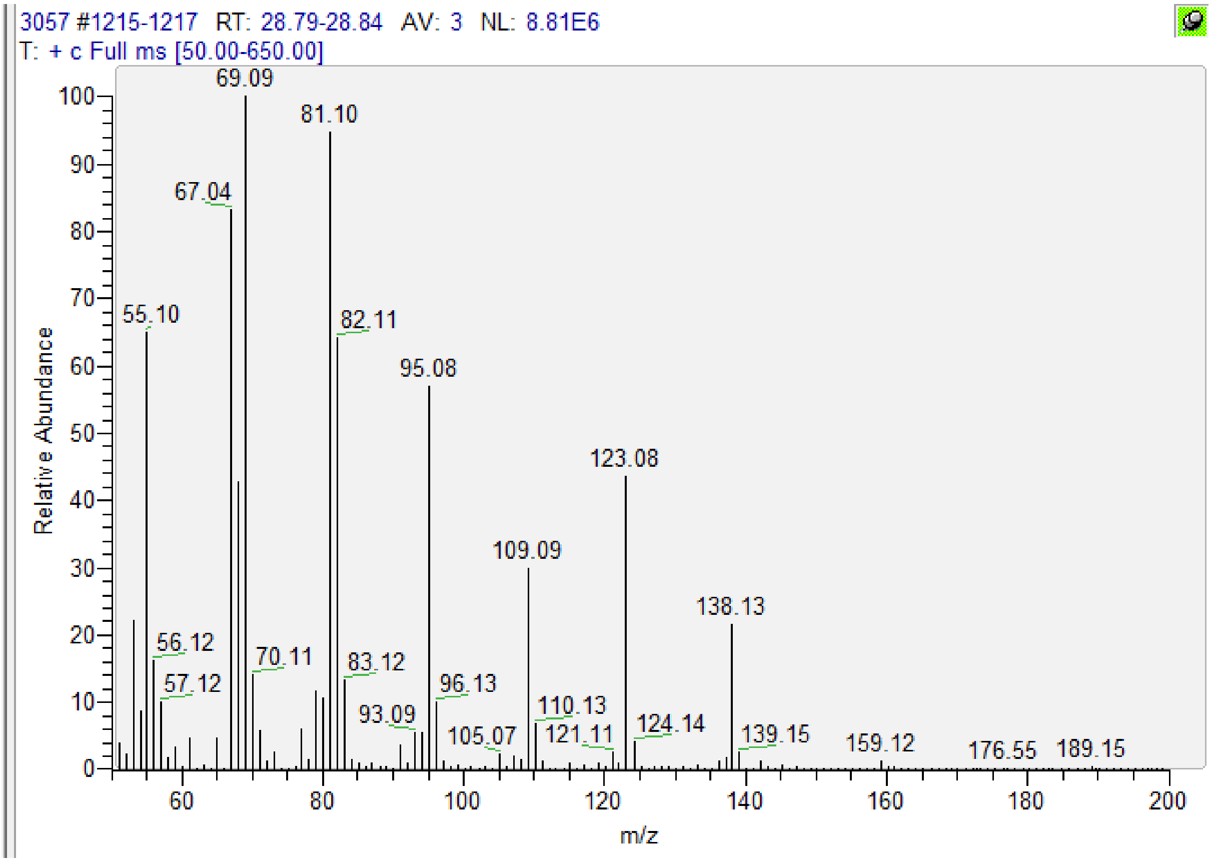
3057 #1215-1217 RT: 28.79-28.84 AV: 3 NL: 8.81E6
T: + c Full ms [50.00-650.00]
Relative Abundance
m/z
69.09
81.10
67.04
55.10
82.11
95.08
123.08
109.09
138.13
56.12
70.11
83.12
57.12
96.13
93.09
110.13
105.07
121.11
124.14
139.15
159.12
176.55
189.15

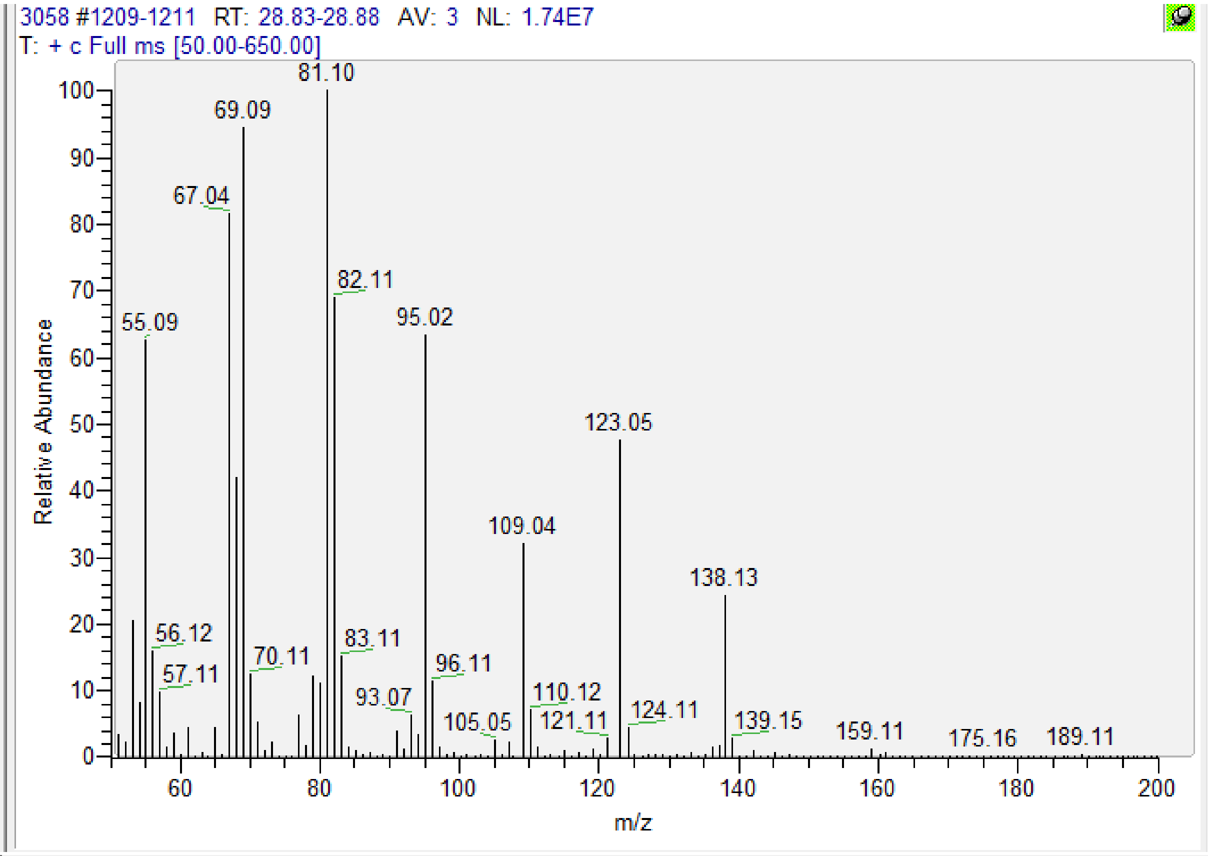
3058 #1209-1211 RT: 28.83-28.88 AV: 3 NL: 1.74E7
T: + c Full ms [50.00-650.00]
Relative Abundance
m/z
81.10
69.09
67.04
82.11
55.09
95.02
123.05
109.04
138.13
56.12
57.11
70.11
83.11
96.11
93.07
110.12
124.11
105.05
121.11
139.15
159.11
175.16
189.11

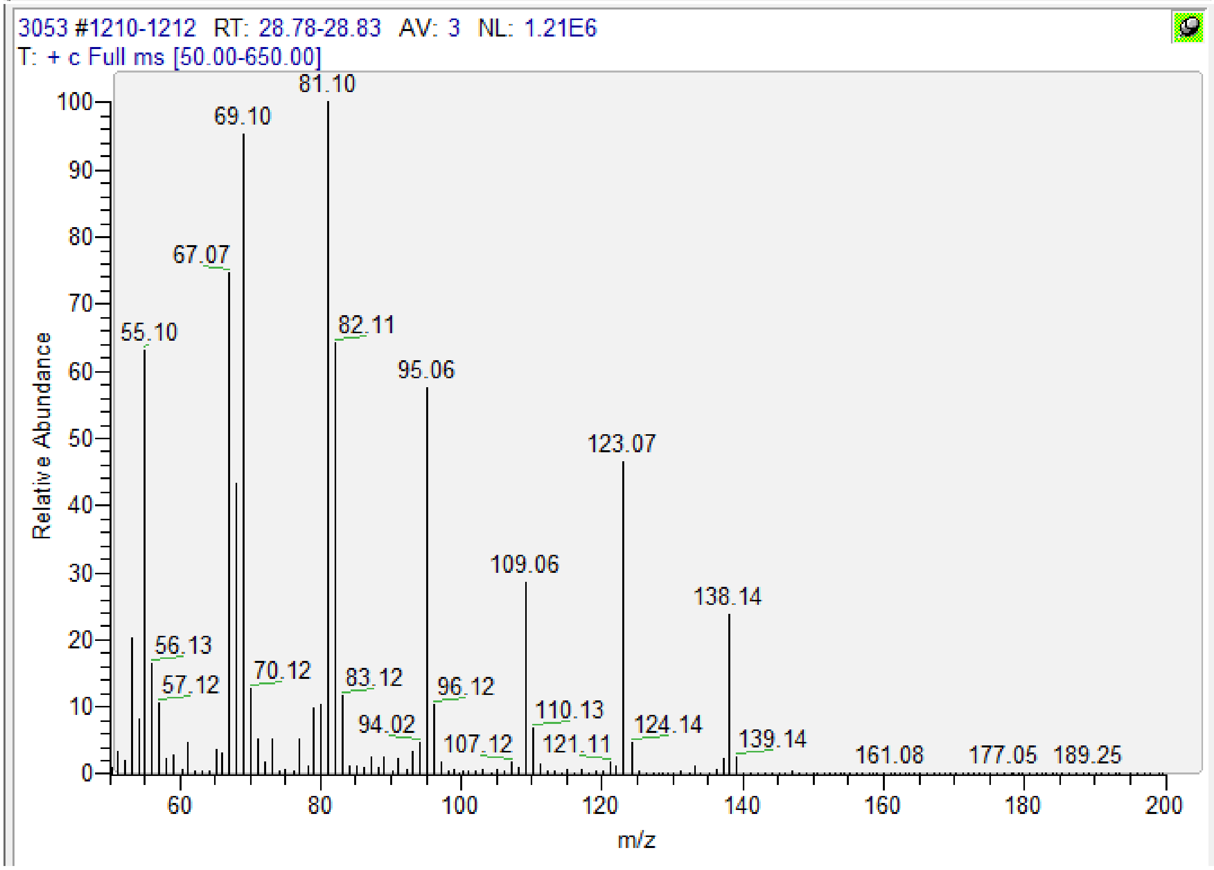
3053 #1210-1212 RT: 28.78-28.83 AV: 3 NL: 1.21E6
T: + c Full ms [50.00-650.00]
Relative Abundance
m/z
81.10
69.10
67.07
55.10
82.11
95.06
123.07
109.06
138.14
56.13
57.12
70.12
83.12
96.12
94.02
110.13
124.14
107.12
121.11
139.14
161.08
177.05
189.25

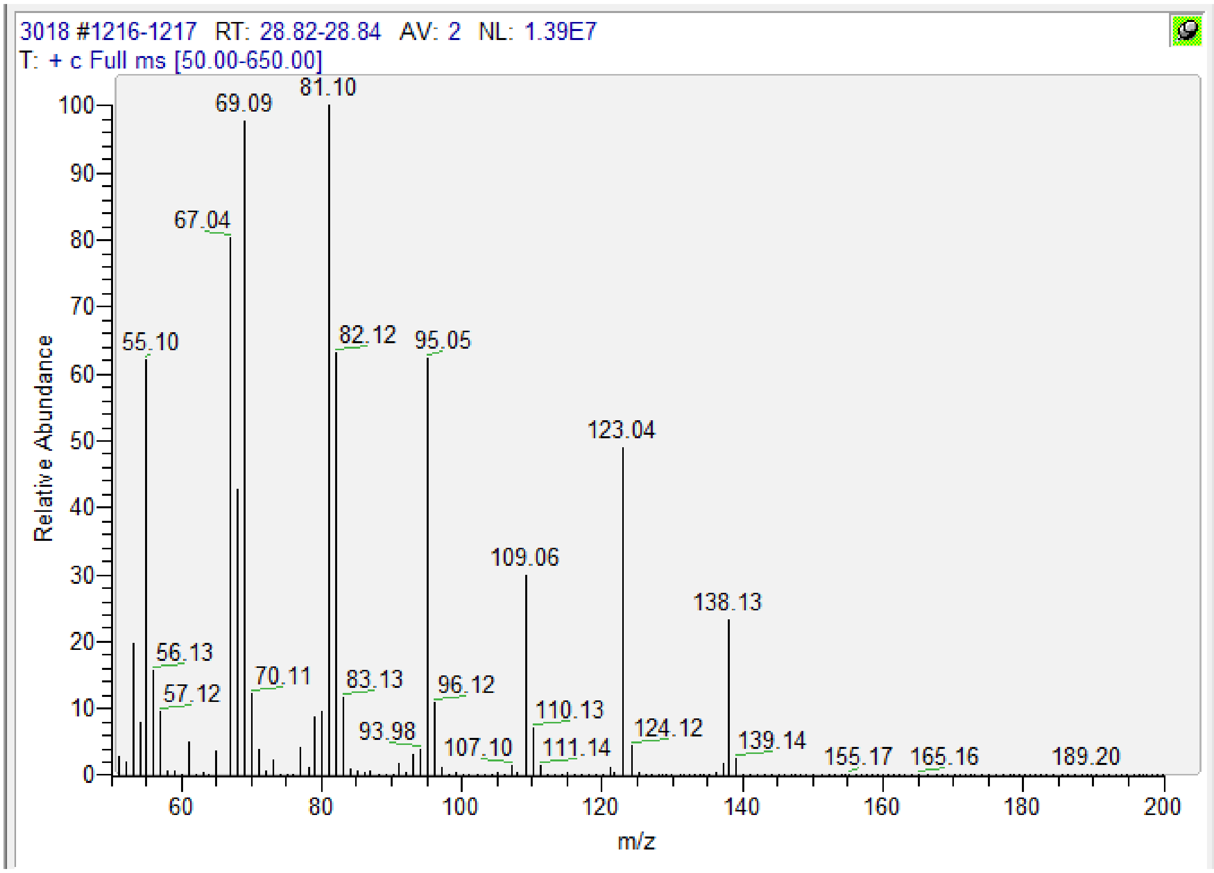
3018 #1216-1217 RT: 28.82-28.84 AV: 2 NL: 1.39E7
T: + c Full ms [50.00-650.00]
Relative Abundance
m/z
55.10
56.13
57.12
67.04
69.09
70.11
81.10
82.12
83.13
93.98
95.05
96.12
107.10
109.06
110.13
111.14
123.04
124.12
138.13
139.14
155.17
165.16
189.20

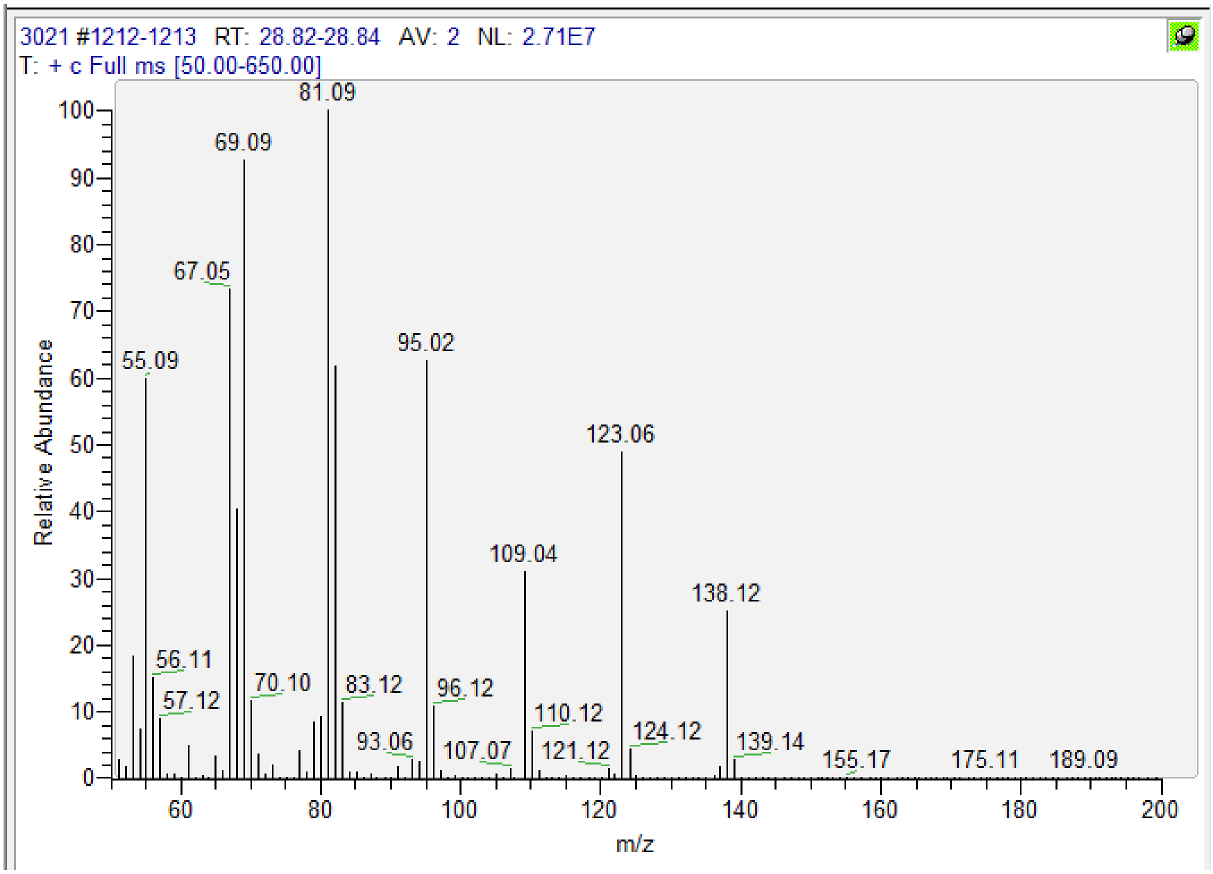
3021 #1212-1213 RT: 28.82-28.84 AV: 2 NL: 2.71E7
T: + c Full ms [50.00-650.00]
Relative Abundance
m/z
81.09
69.09
67.05
55.09
95.02
123.06
109.04
138.12
56.11
57.12
70.10
83.12
96.12
110.12
124.12
139.14
93.06
107.07
121.12
155.17
175.11
189.09

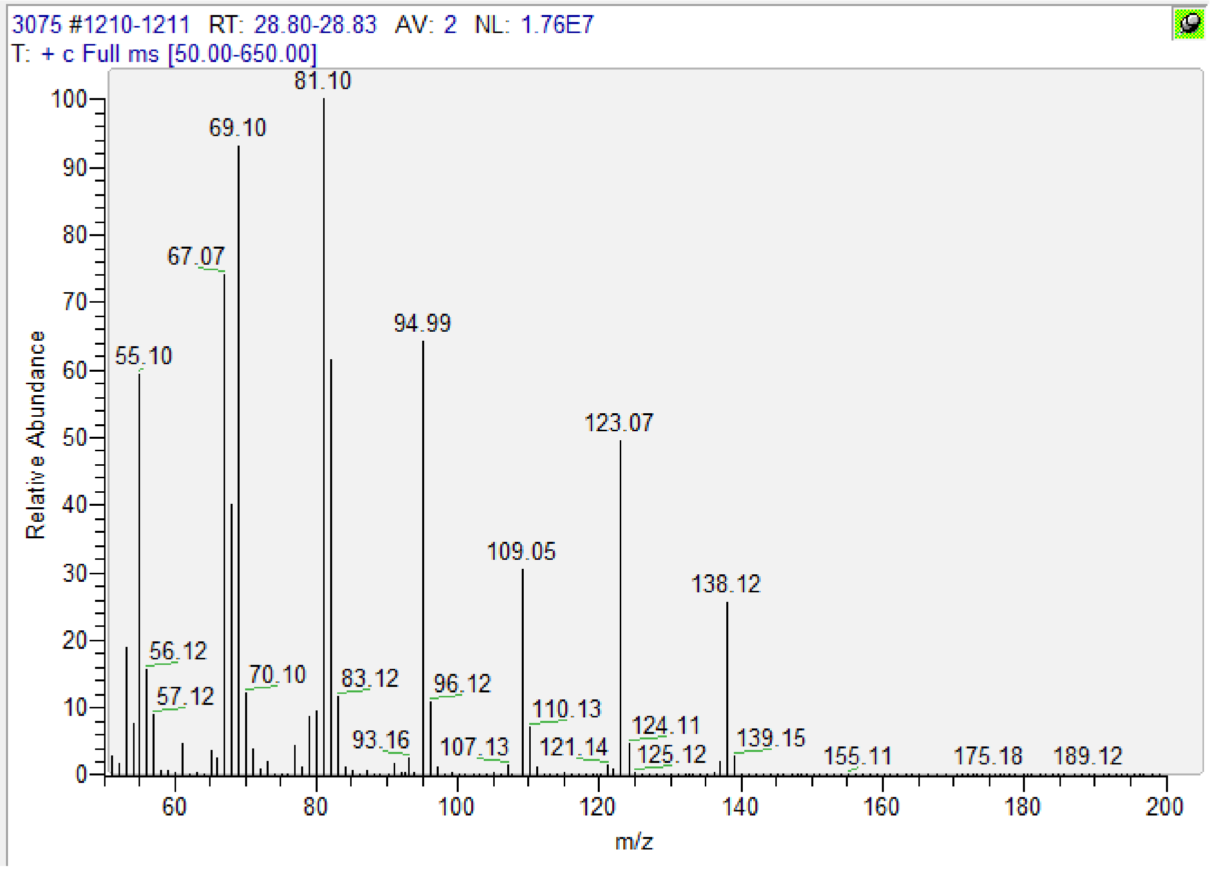

3075 #1210-1211 RT: 28.80-28.83 AV: 2 NL: 1.76E7
T: + c Full ms [50.00-650.00]
Relative Abundance
m/z
81.10
69.10
67.07
55.10
94.99
123.07
109.05
138.12
56.12
57.12
70.10
83.12
96.12
110.13
124.11
93.16
107.13
121.14
125.12
139.15
155.11
175.18
189.12

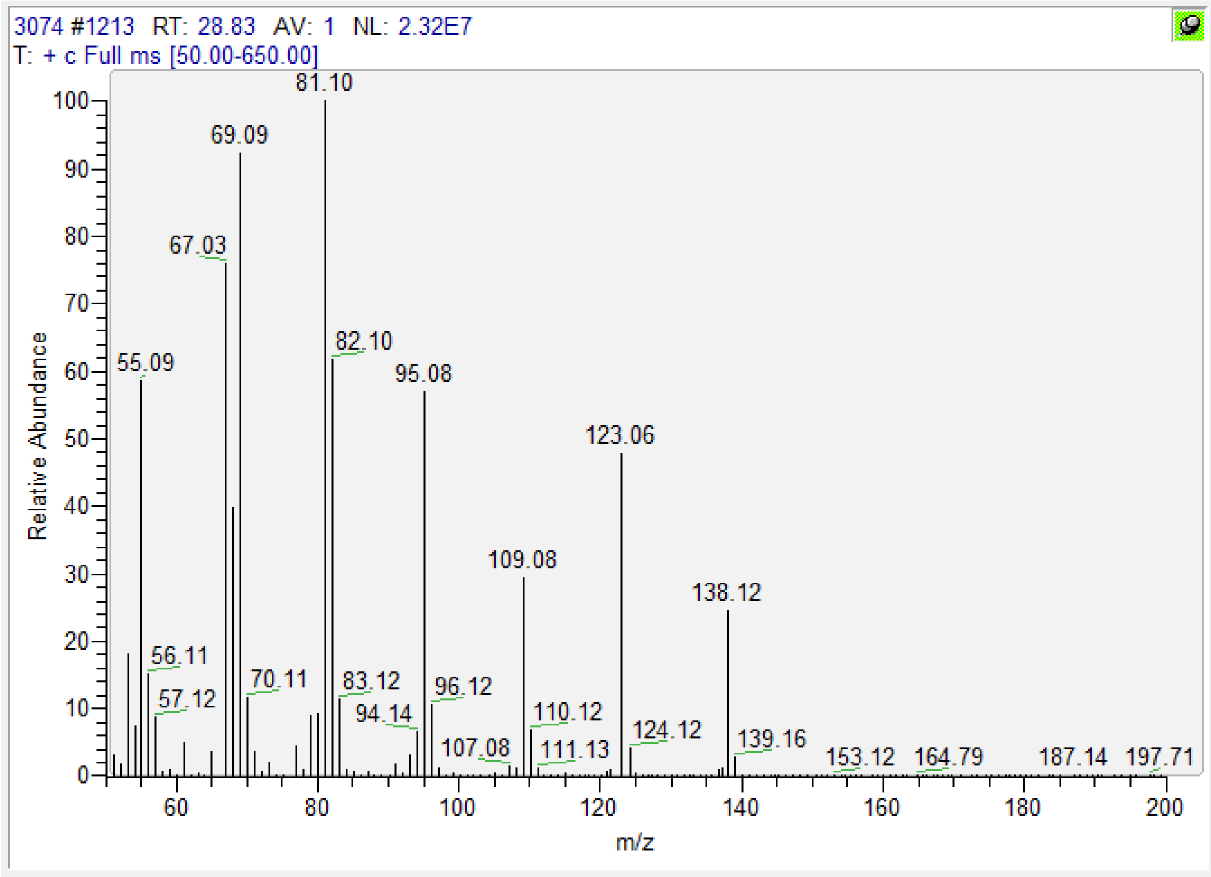

3074 #1213 RT: 28.83 AV: 1 NL: 2.32E7
T: + c Full ms [50.00-650.00]
Relative Abundance
m/z
81.10
69.09
67.03
82.10
55.09
95.08
123.06
109.08
138.12
56.11
57.12
70.11
83.12
94.14
96.12
107.08
110.12
111.13
124.12
139.16
153.12
164.79
187.14
197.71

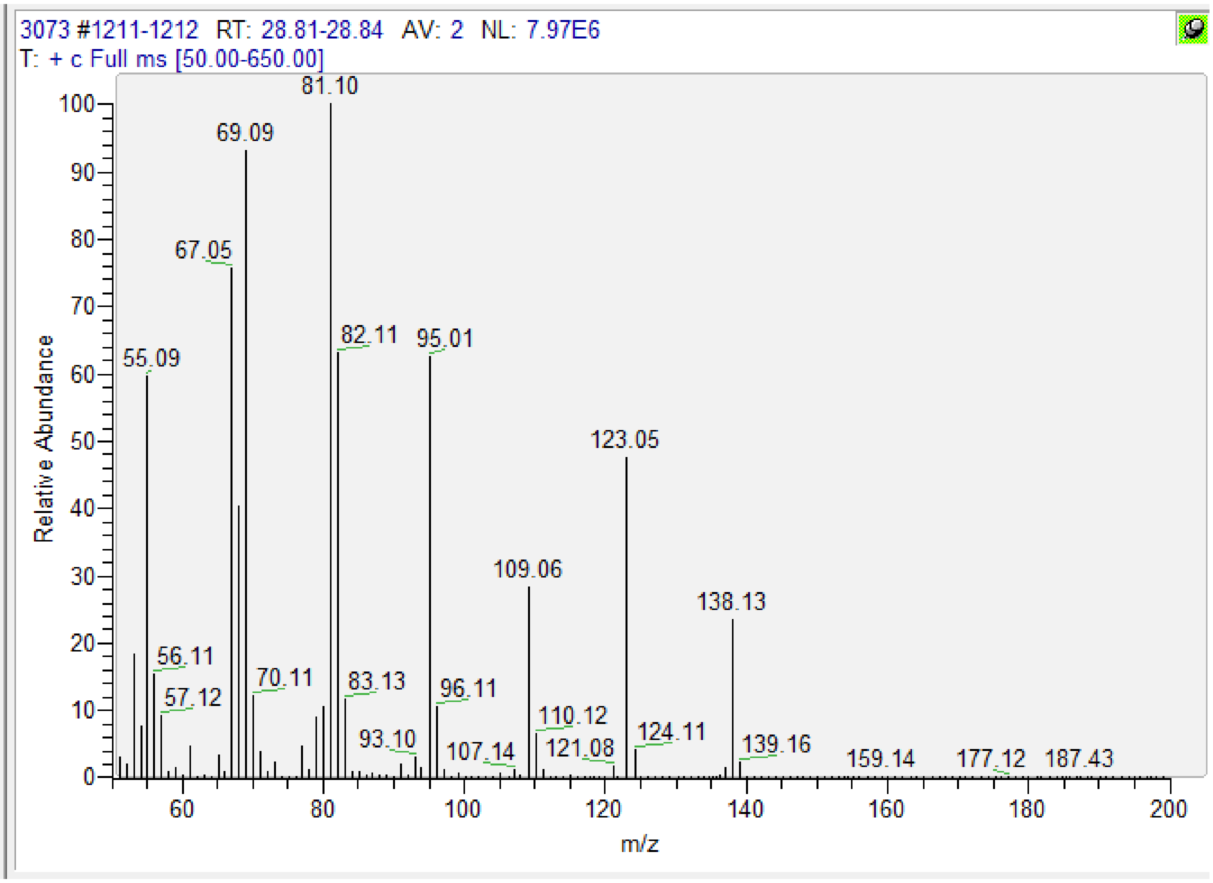
3073 #1211-1212 RT: 28.81-28.84 AV: 2 NL: 7.97E6
T: + c Full ms [50.00-650.00]
Relative Abundance
m/z
81.10
69.09
67.05
55.09
82.11
95.01
123.05
109.06
138.13
56.11
57.12
70.11
83.13
96.11
93.10
110.12
124.11
107.14
121.08
139.16
159.14
177.12
187.43

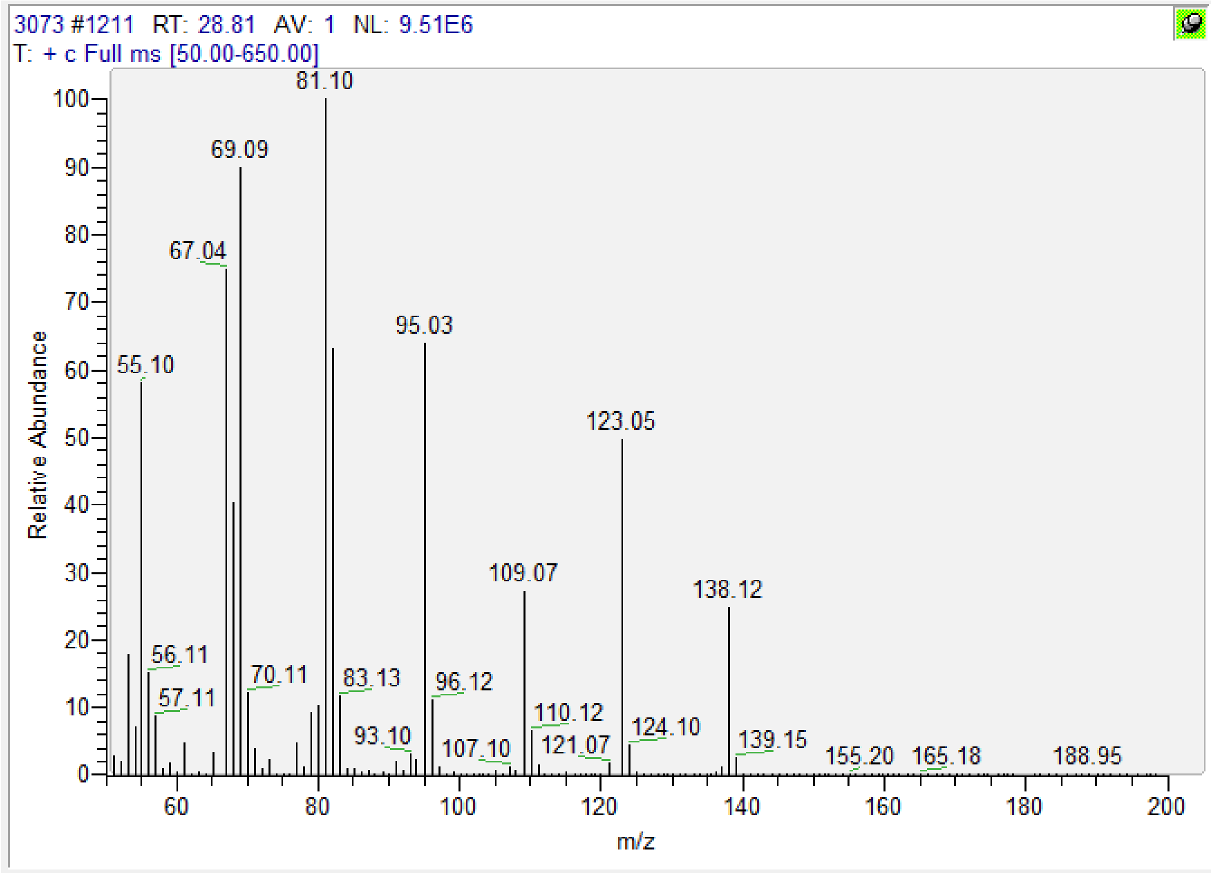

3073 #1211 RT: 28.81 AV: 1 NL: 9.51E6
T: + c Full ms [50.00-650.00]
Relative Abundance
m/z
81.10
69.09
67.04
95.03
55.10
123.05
109.07
138.12
56.11
70.11
83.13
96.12
57.11
110.12
124.10
93.10
121.07
139.15
107.10
155.20
165.18
188.95

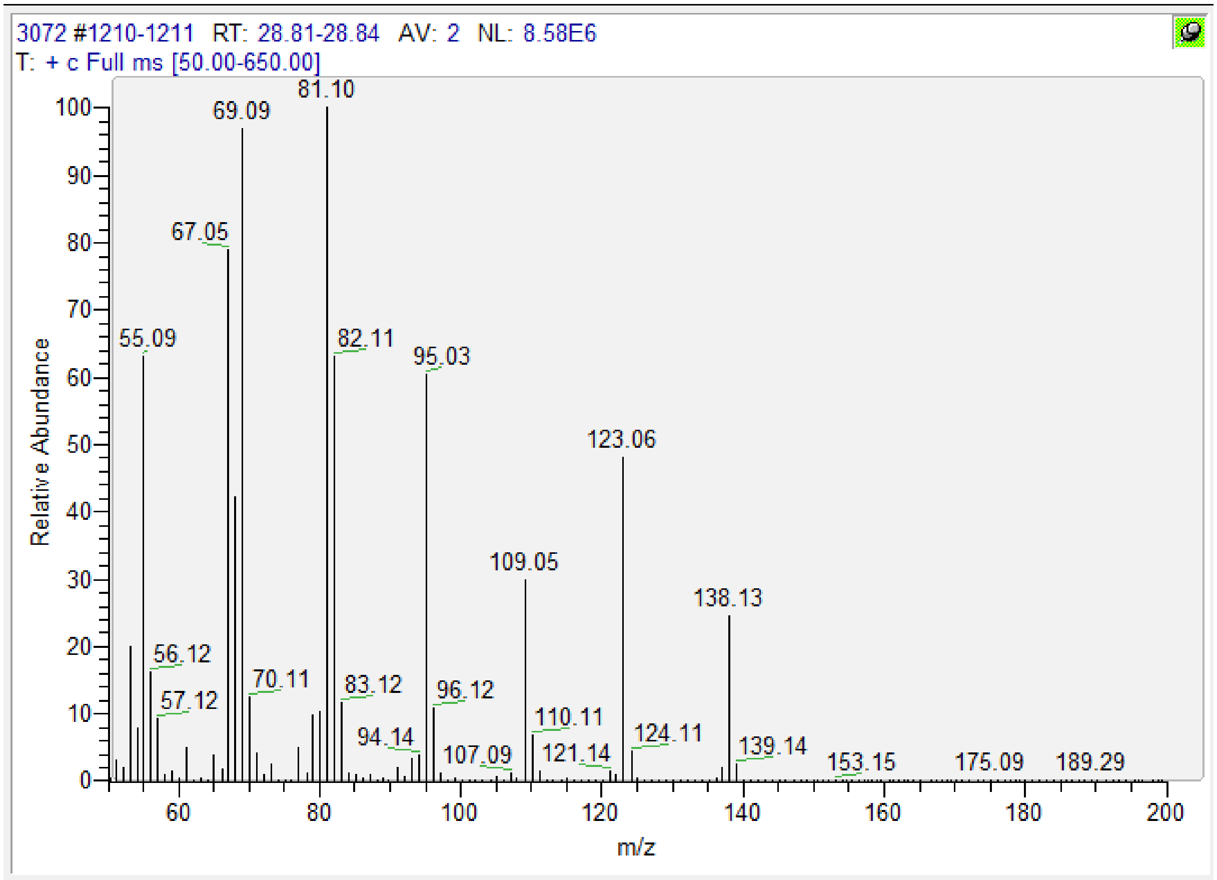
3072 #1210-1211 RT: 28.81-28.84 AV: 2 NL: 8.58E6
T: + c Full ms [50.00-650.00]
81.10
69.09
67.05
55.09
82.11
95.03
123.06
109.05
138.13
56.12
70.11
83.12
96.12
57.12
110.11
94.14
124.11
139.14
107.09
121.14
153.15
175.09
189.29
Relative Abundance
m/z

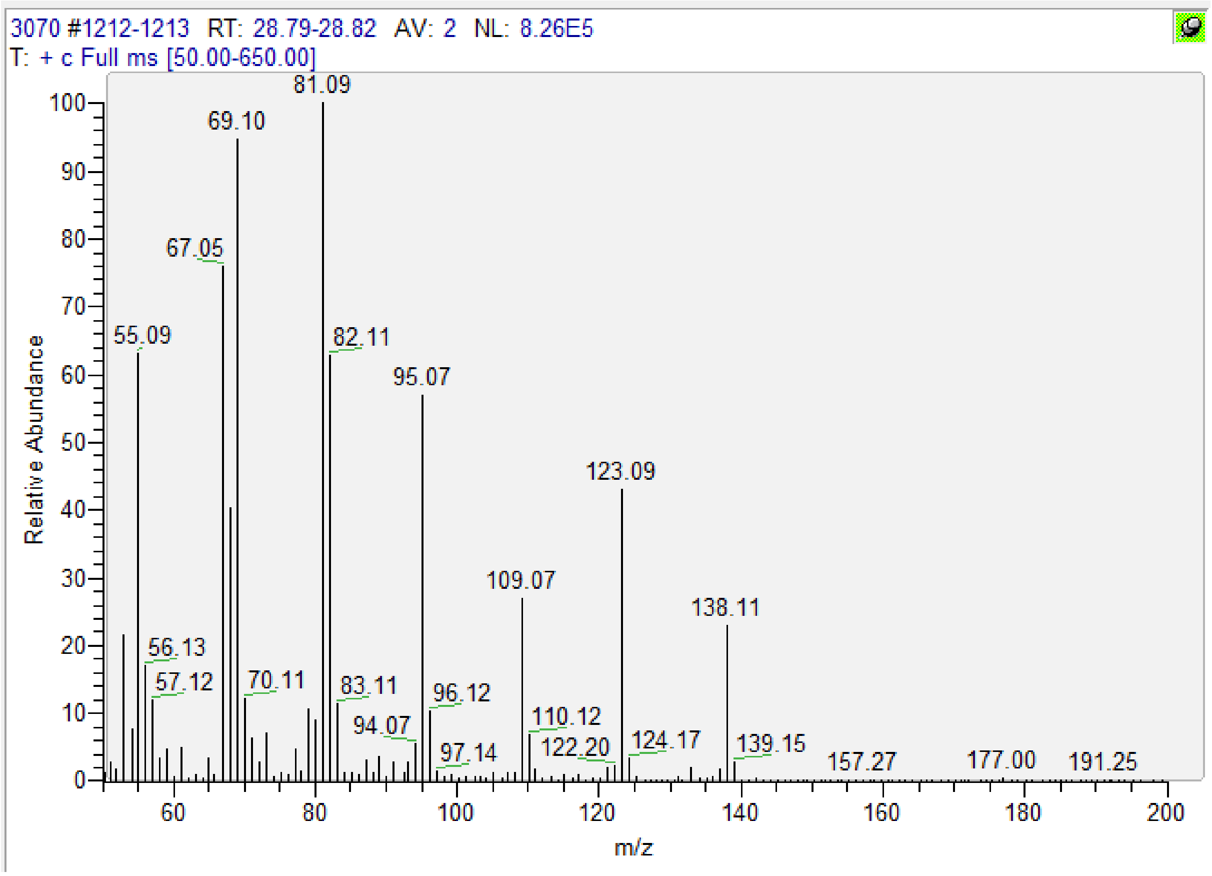

Citral

Reference

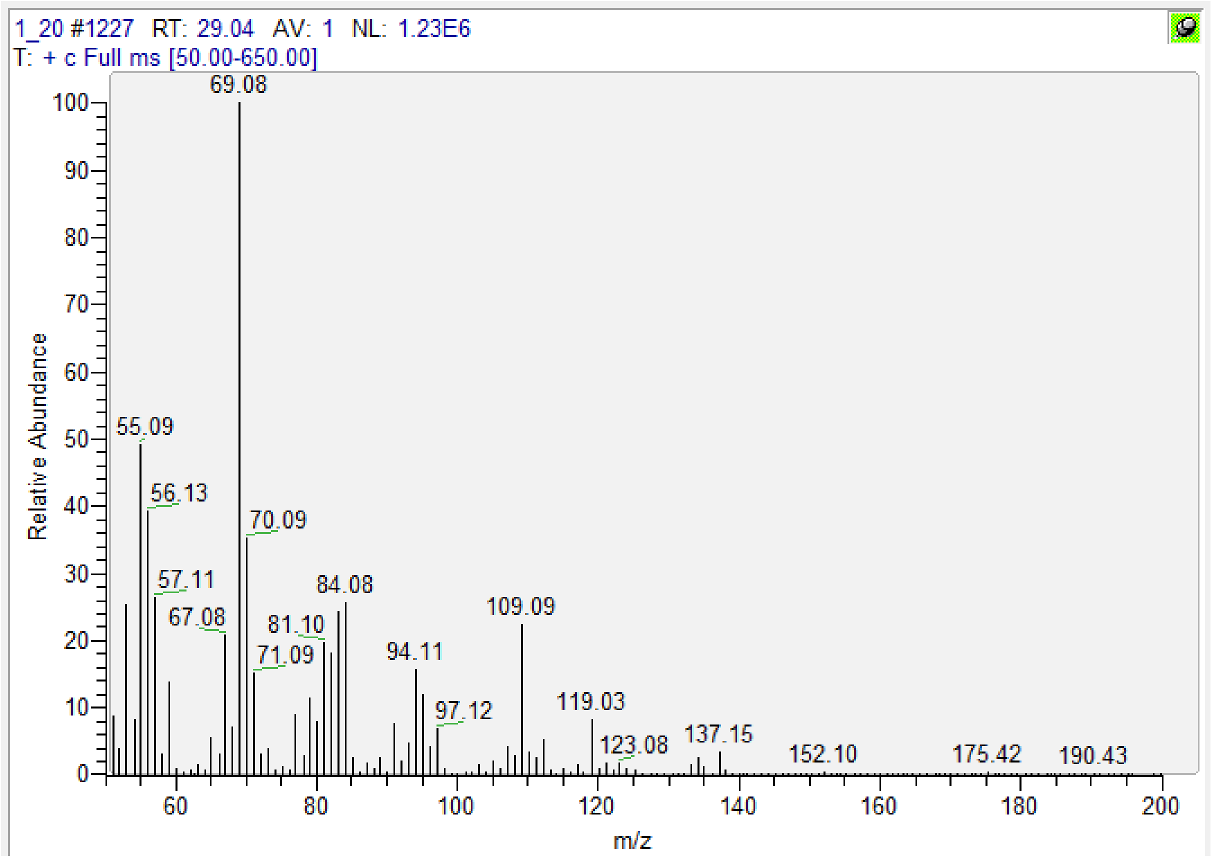

1_20 #1227 RT: 29.04 AV: 1 NL: 1.23E6
T: + c Full ms [50.00-650.00]
Relative Abundance
m/z
69.08
55.09
56.13
70.09
57.11
67.08
84.08
81.10
71.09
109.09
94.11
97.12
119.03
123.08
137.15
152.10
175.42
190.43

Samples

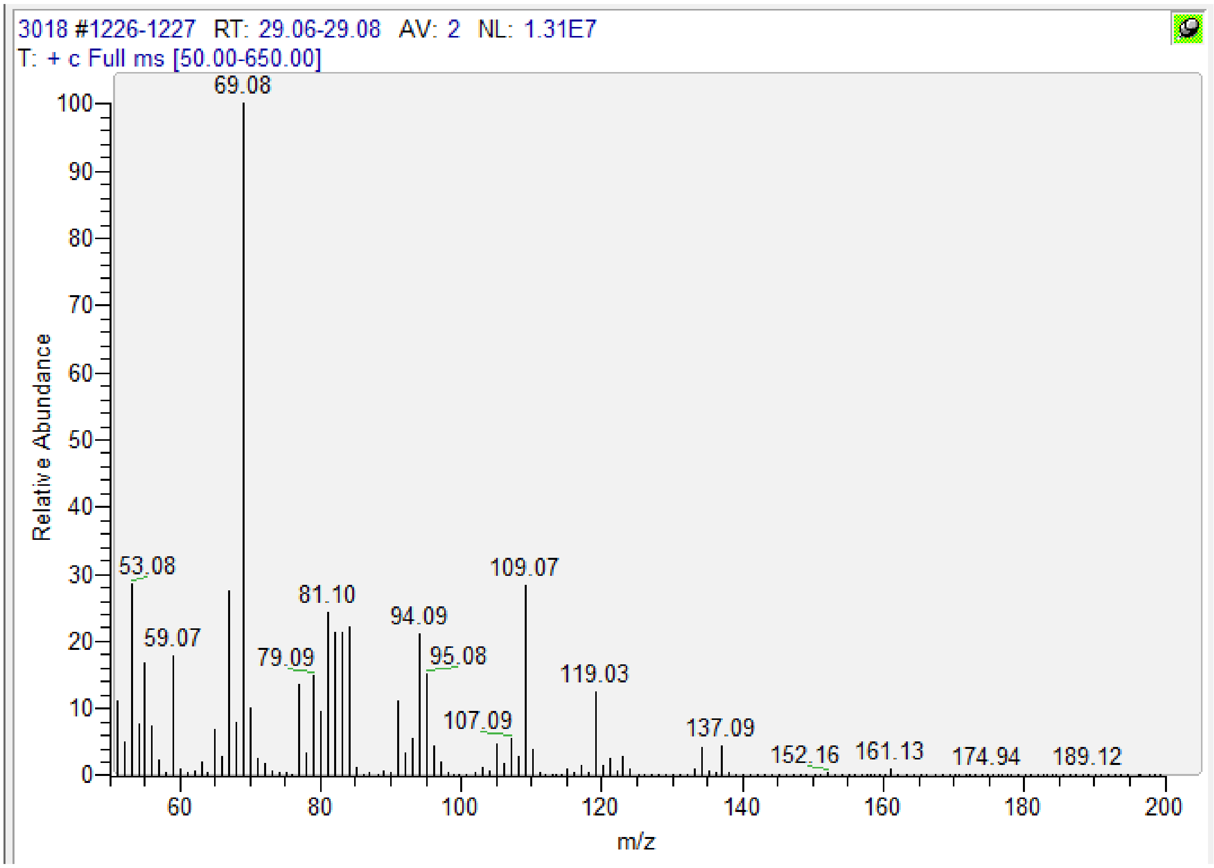

3018 #1226-1227 RT: 29.06-29.08 AV: 2 NL: 1.31E7
T: + c Full ms [50.00-650.00]
Relative Abundance
m/z
69.08
53.08
59.07
79.09
81.10
94.09
95.08
107.09
109.07
119.03
137.09
152.16
161.13
174.94
189.12

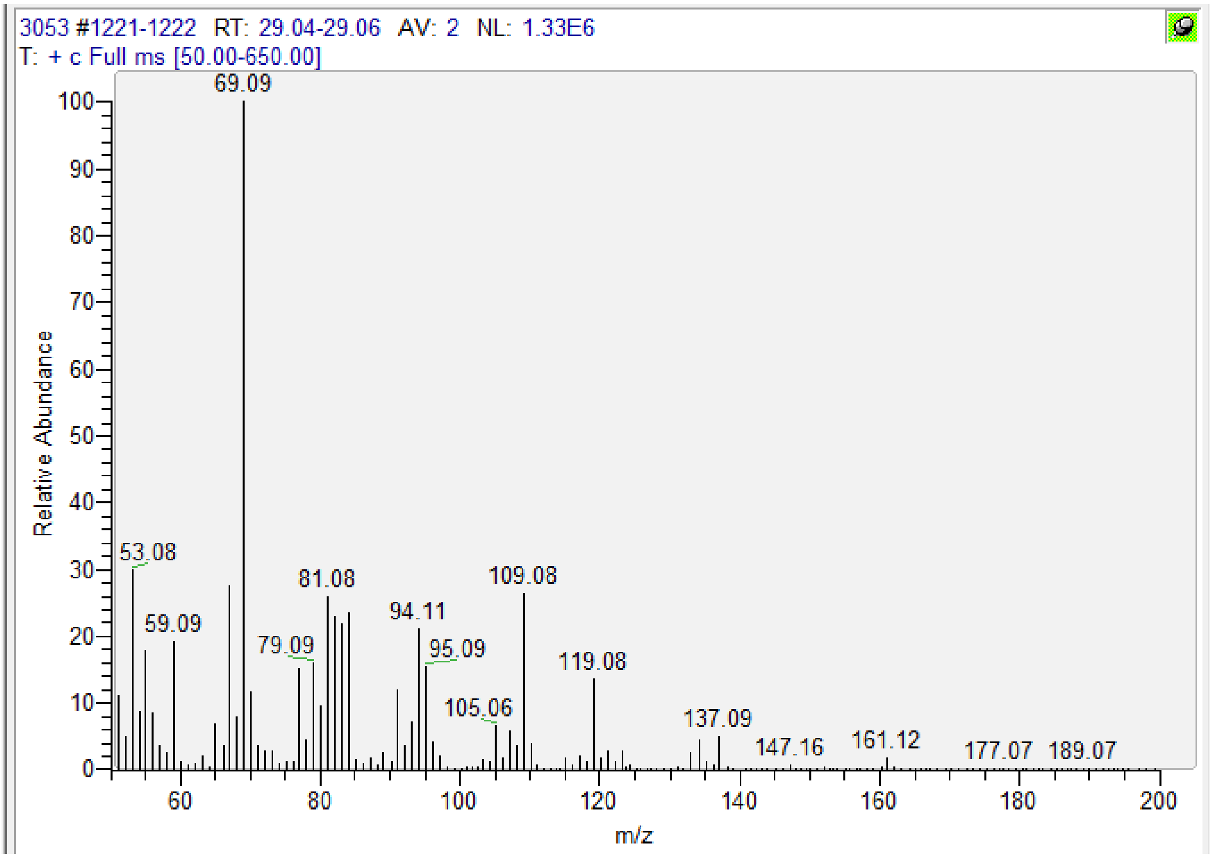

3053 #1221-1222 RT: 29.04-29.06 AV: 2 NL: 1.33E6
T: + c Full ms [50.00-650.00]
69.09
53.08
59.09
79.09
81.08
94.11
95.09
105.06
109.08
119.08
137.09
147.16
161.12
177.07
189.07
Relative Abundance
m/z

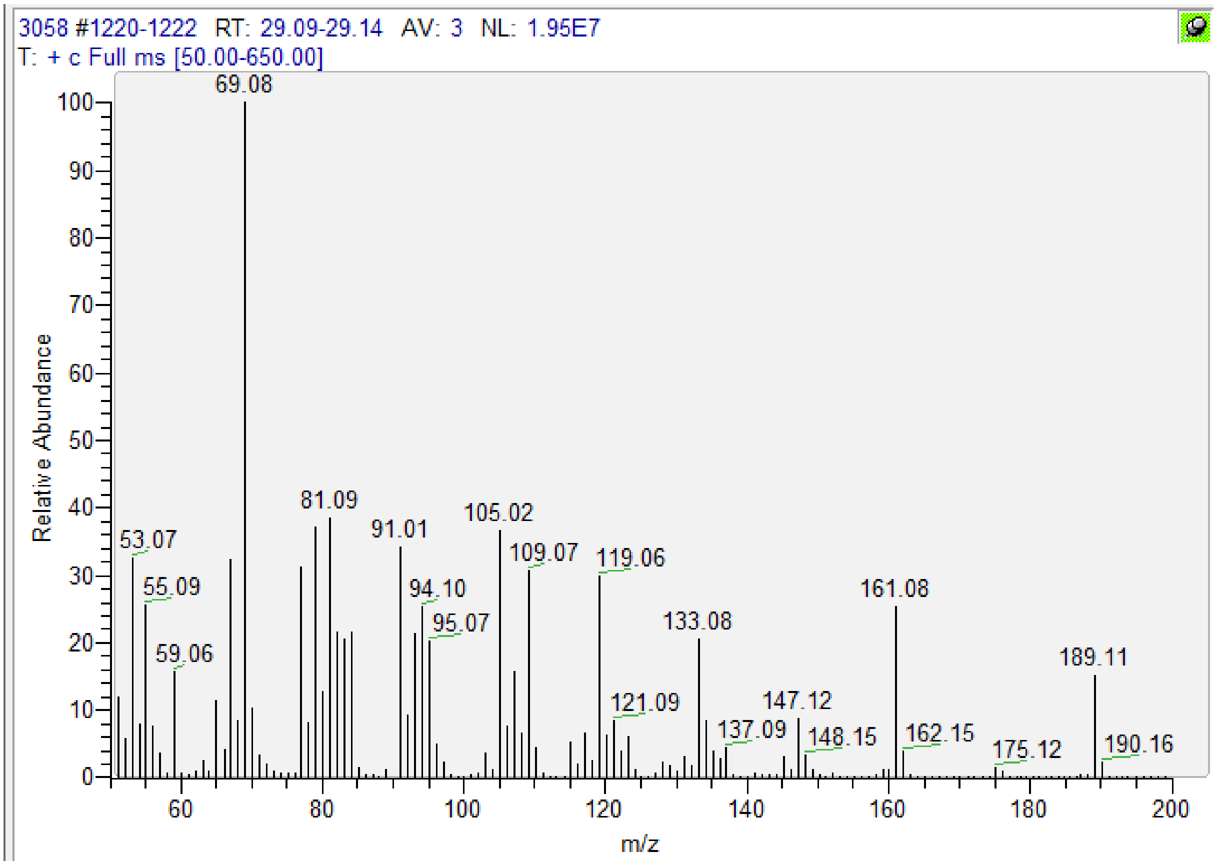
3058 #1220-1222 RT: 29.09-29.14 AV: 3 NL: 1.95E7
T: + c Full ms [50.00-650.00]
Relative Abundance
m/z
69.08
81.09
91.01
105.02
109.07
119.06
53.07
55.09
59.06
94.10
95.07
133.08
161.08
189.11
121.09
137.09
147.12
148.15
162.15
175.12
190.16

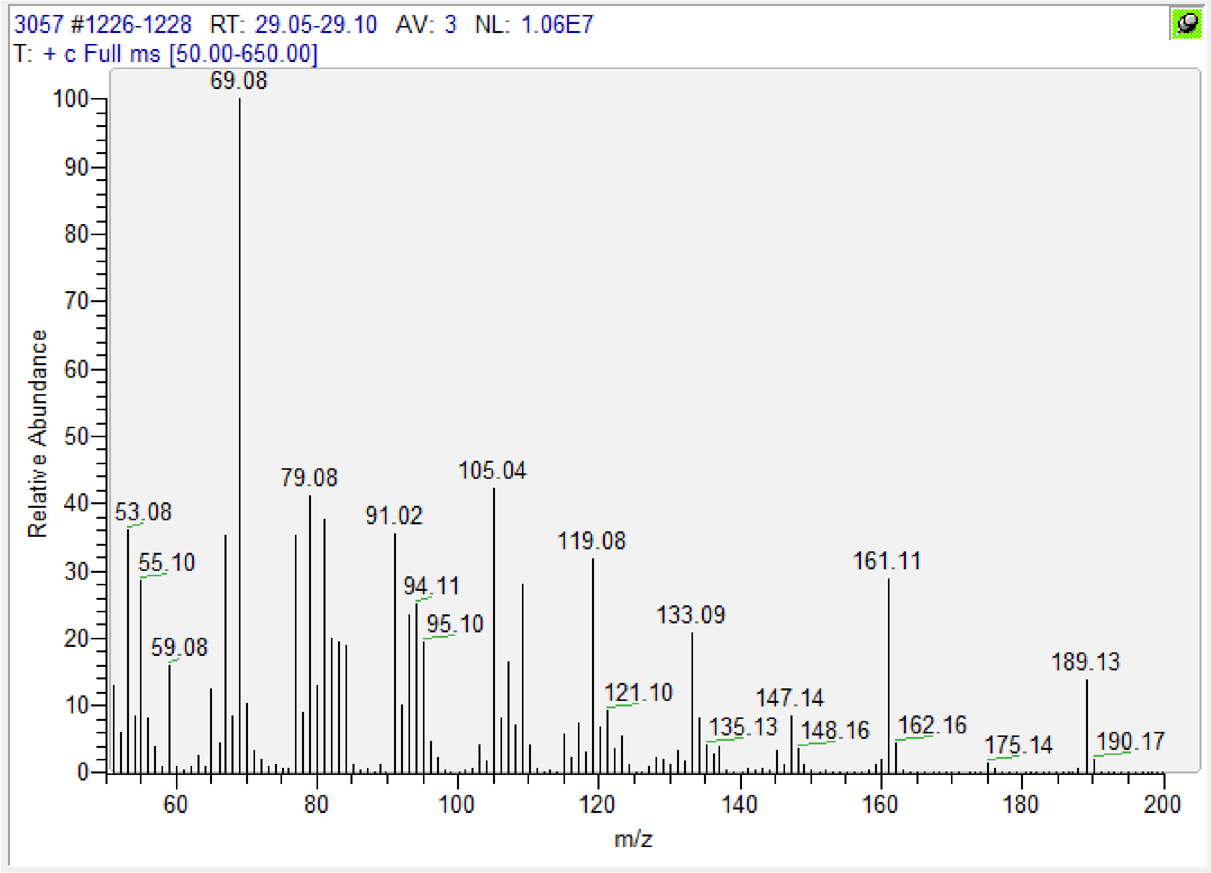
3057 #1226-1228 RT: 29.05-29.10 AV: 3 NL: 1.06E7
T: + c Full ms [50.00-650.00]
69.08
79.08
105.04
53.08
91.02
55.10
119.08
161.11
94.11
133.09
95.10
59.08
189.13
121.10
147.14
135.13
148.16
162.16
175.14
190.17
Relative Abundance
m/z

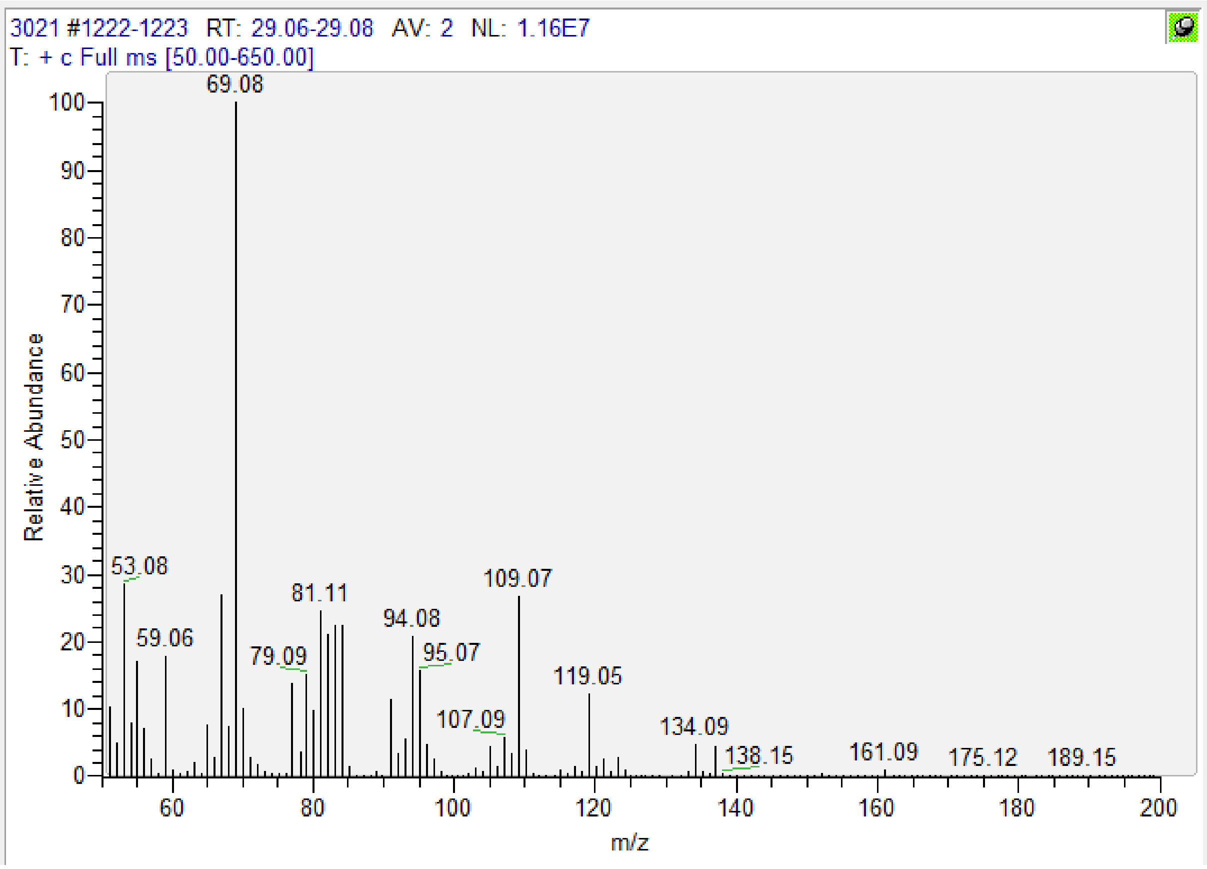

3021 #1222-1223 RT: 29.06-29.08 AV: 2 NL: 1.16E7
T: + c Full ms [50.00-650.00]
Relative Abundance
m/z
69.08
53.08
59.06
79.09
81.11
94.08
95.07
107.09
109.07
119.05
134.09
138.15
161.09
175.12
189.15

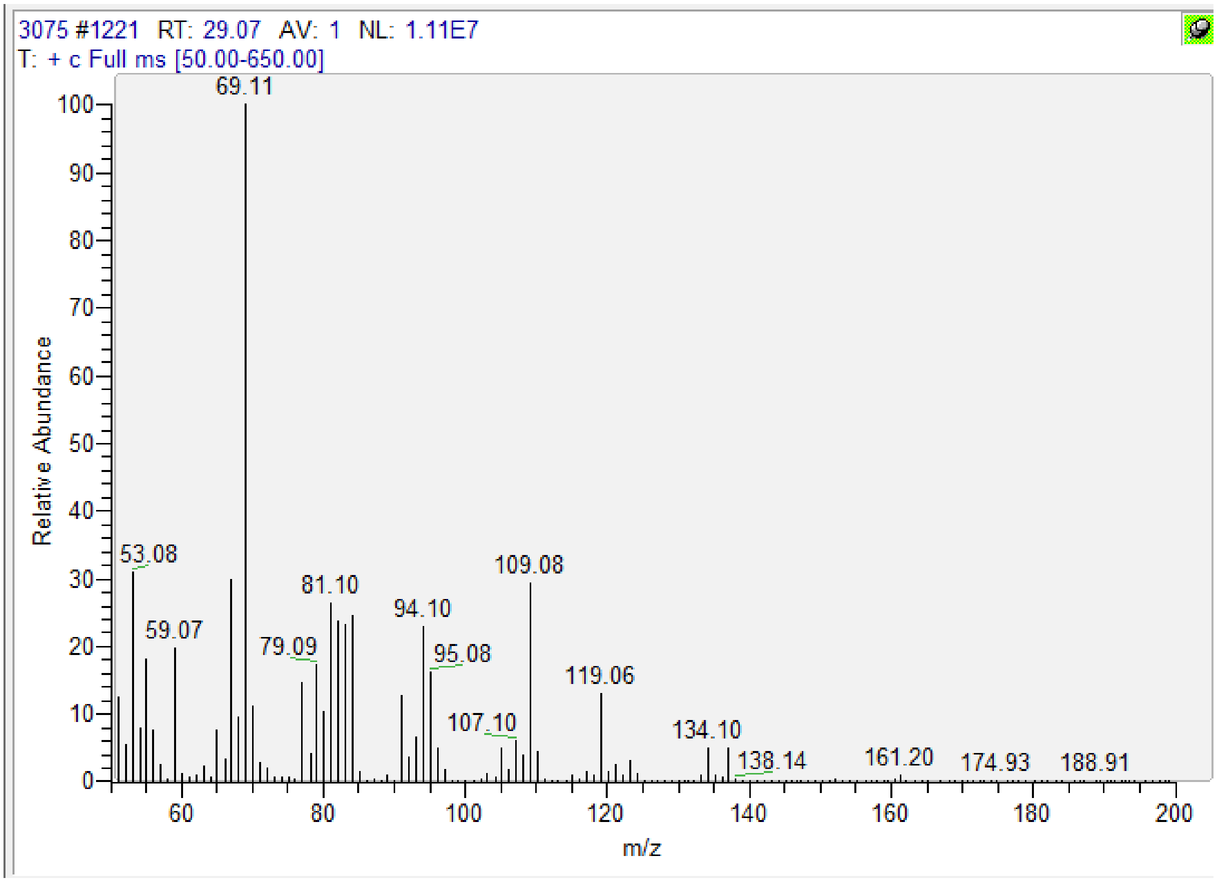
3075 #1221 RT: 29.07 AV: 1 NL: 1.11E7
T: + c Full ms [50.00-650.00]
Relative Abundance
m/z
69.11
53.08
59.07
79.09
81.10
94.10
95.08
107.10
109.08
119.06
134.10
138.14
161.20
174.93
188.91

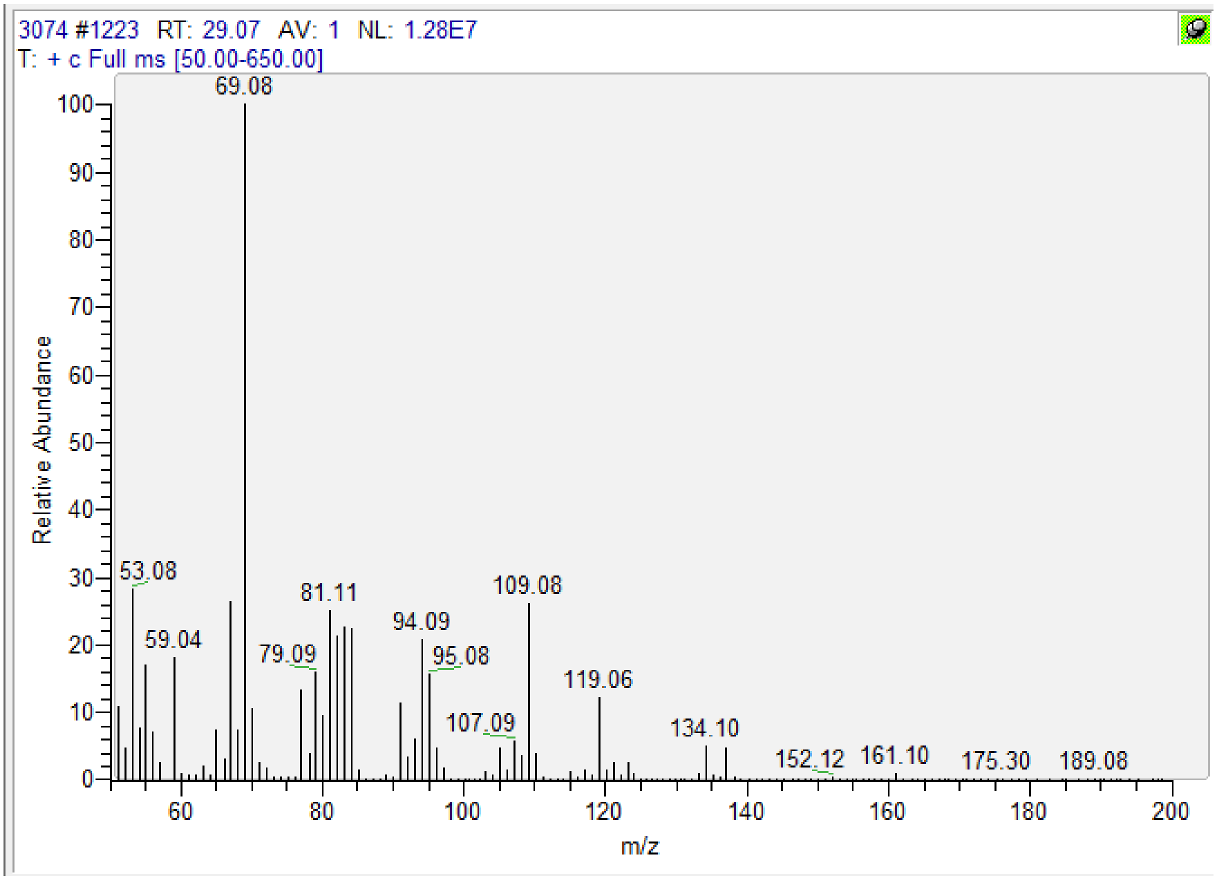
3074 #1223 RT: 29.07 AV: 1 NL: 1.28E7
T: + c Full ms [50.00-650.00]
69.08
53.08
59.04
79.09
81.11
94.09
95.08
107.09
109.08
119.06
134.10
152.12
161.10
175.30
189.08
Relative Abundance
m/z

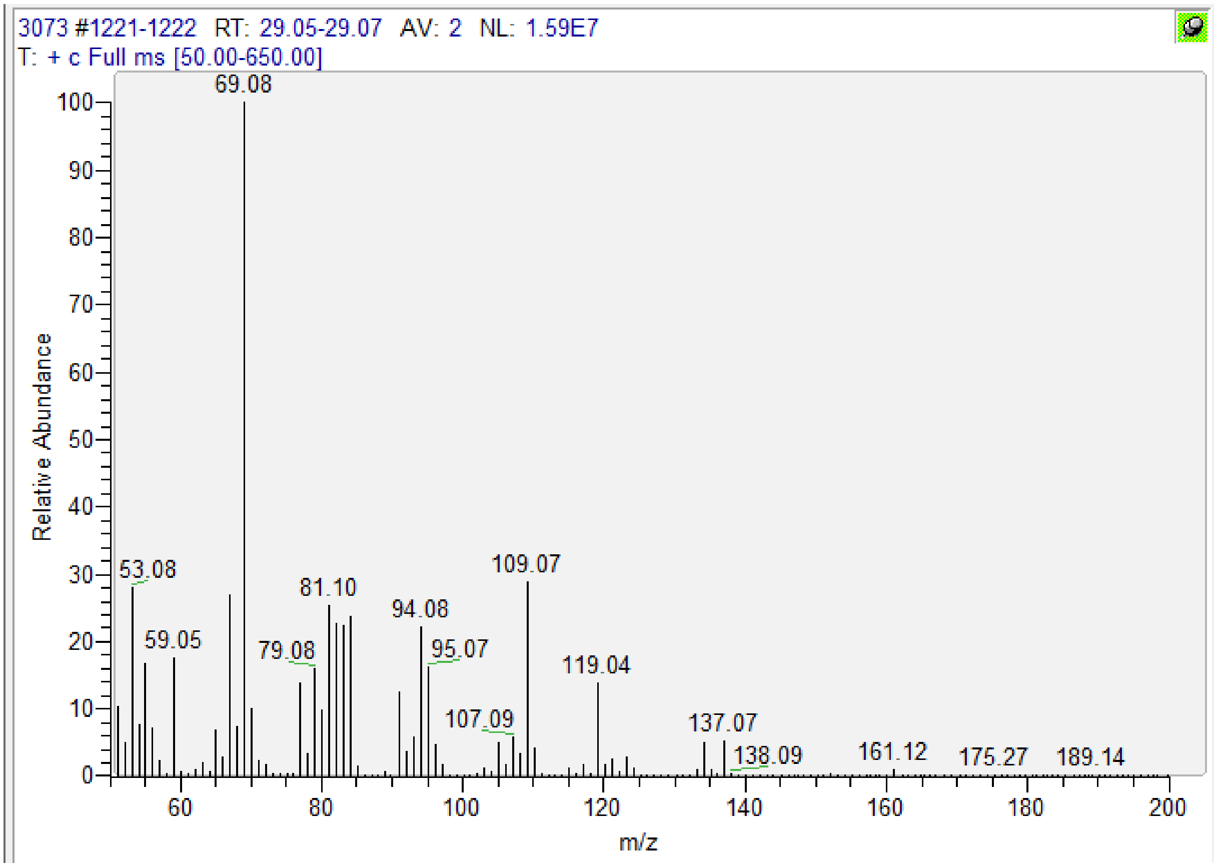
3073 #1221-1222 RT: 29.05-29.07 AV: 2 NL: 1.59E7
T: + c Full ms [50.00-650.00]
Relative Abundance
m/z
69.08
53.08
59.05
79.08
81.10
94.08
95.07
107.09
109.07
119.04
137.07
138.09
161.12
175.27
189.14

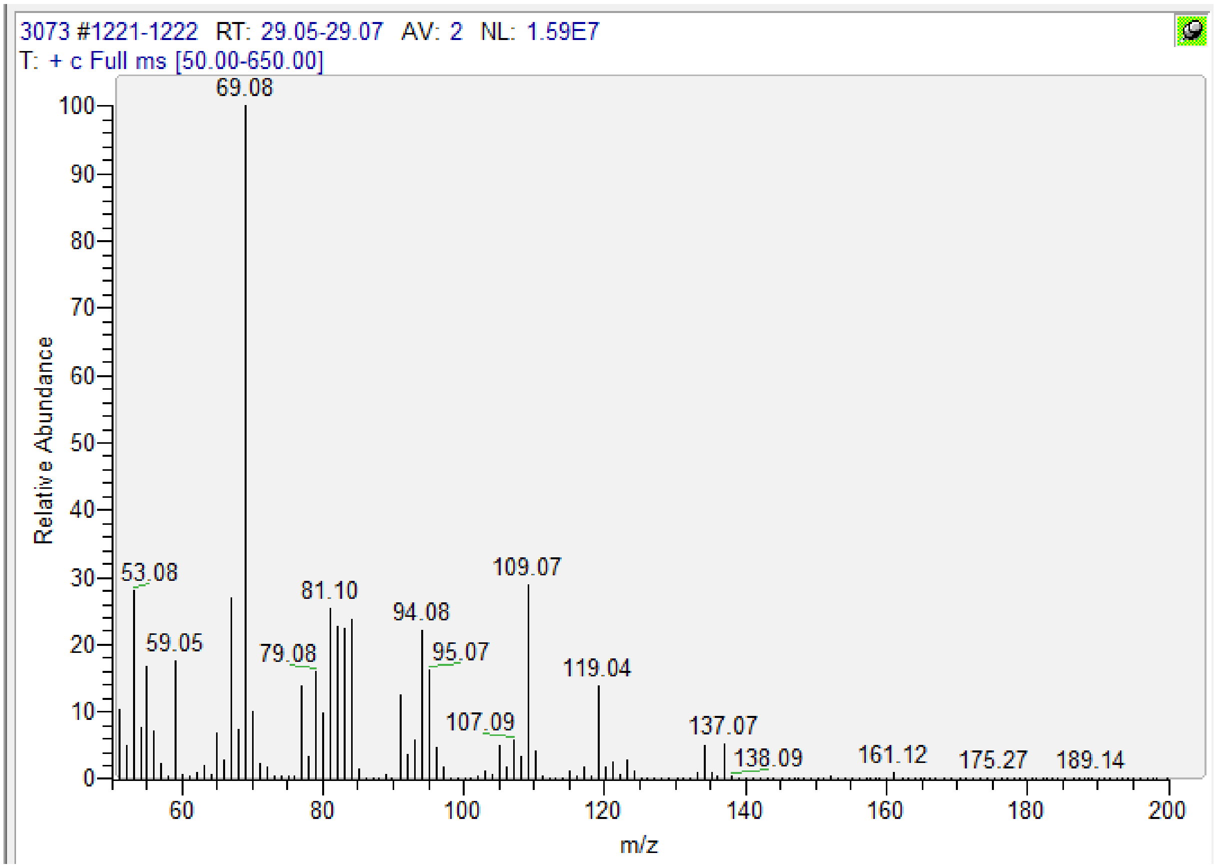
3073 #1221-1222 RT: 29.05-29.07 AV: 2 NL: 1.59E7
T: + c Full ms [50.00-650.00]
Relative Abundance
m/z
69.08
53.08
59.05
79.08
81.10
94.08
95.07
107.09
109.07
119.04
137.07
138.09
161.12
175.27
189.14

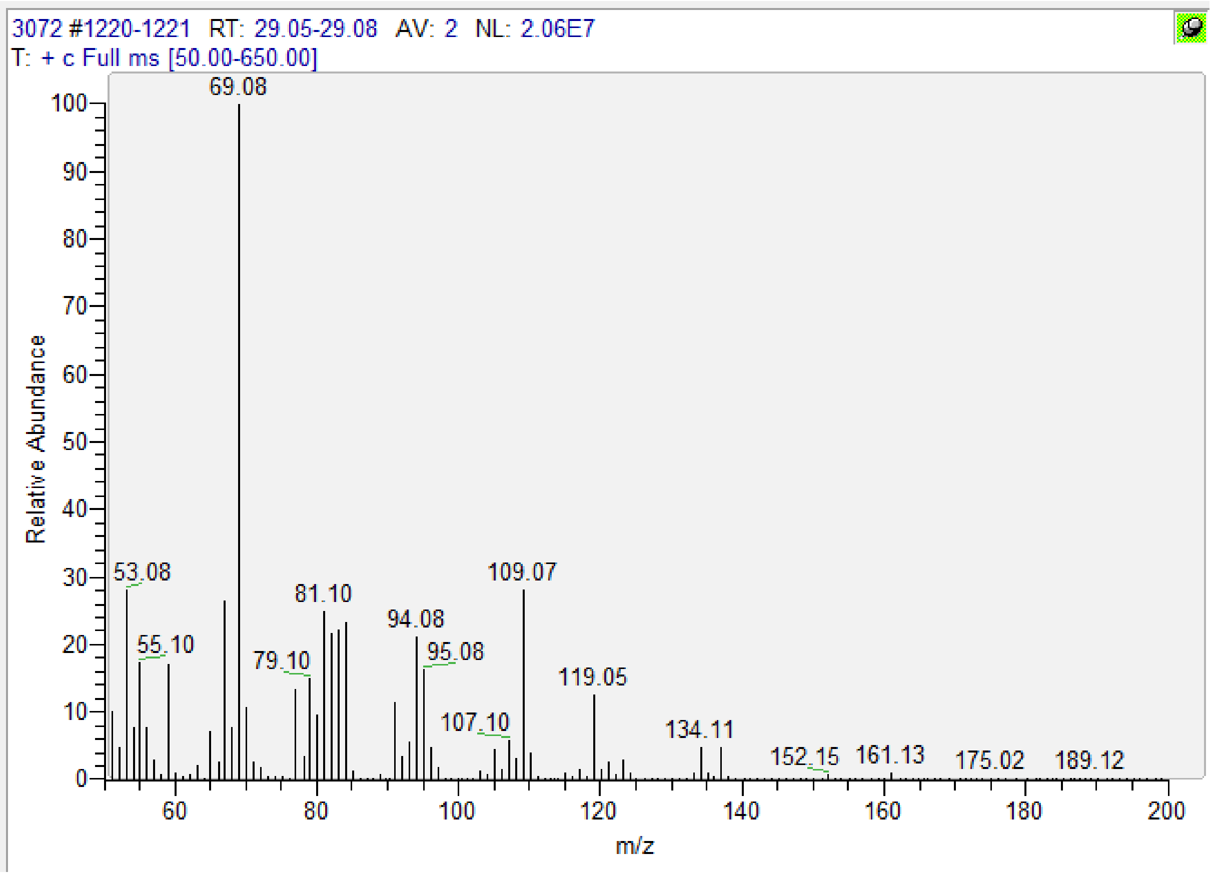
3072 #1220-1221 RT: 29.05-29.08 AV: 2 NL: 2.06E7
T: + c Full ms [50.00-650.00]
Relative Abundance
m/z
69.08
53.08
55.10
79.10
81.10
94.08
95.08
107.10
109.07
119.05
134.11
152.15
161.13
175.02
189.12

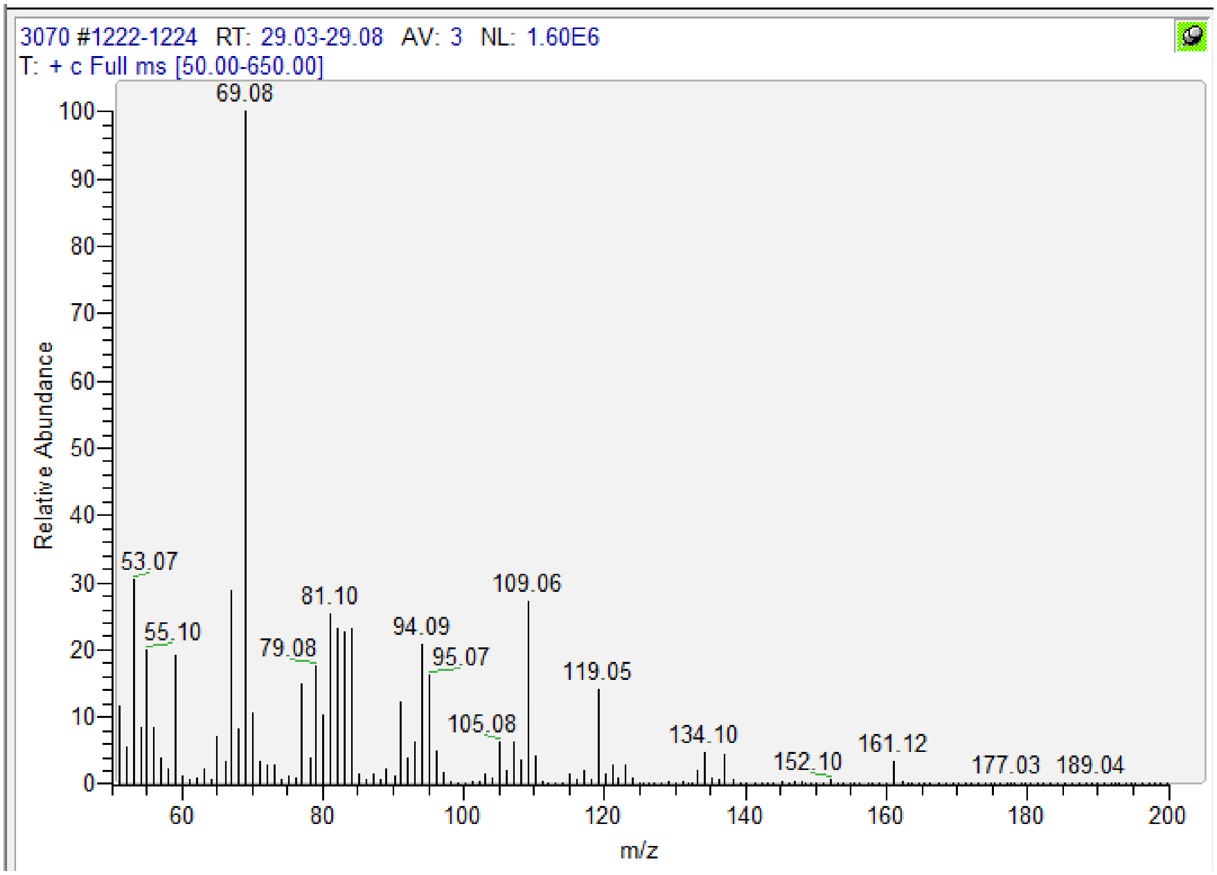

Neryl Acetat

Reference

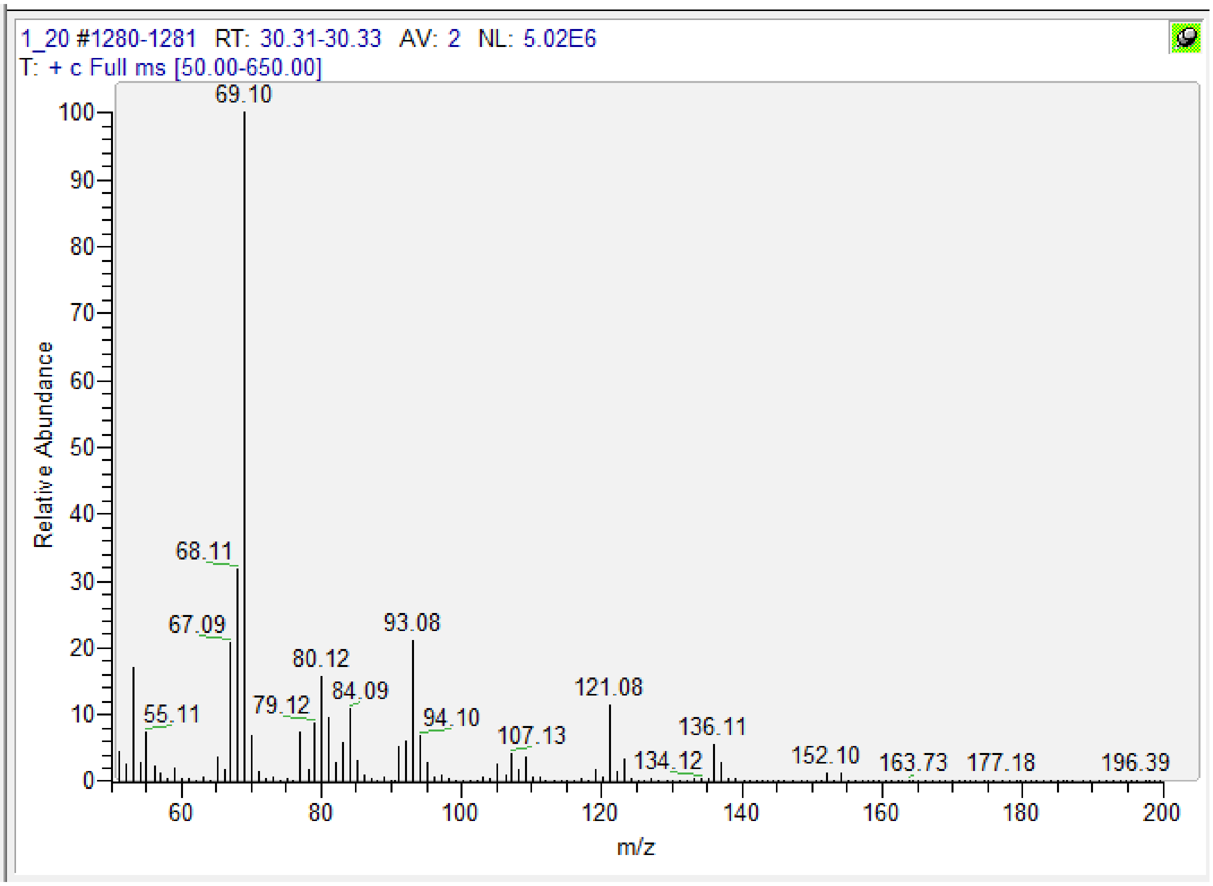
1_20 #1280-1281 RT: 30.31-30.33 AV: 2 NL: 5.02E6
T: + c Full ms [50.00-650.00]
69.10
68.11
67.09
93.08
80.12
84.09
79.12
55.11
94.10
121.08
107.13
136.11
134.12
152.10
163.73
177.18
196.39
Relative Abundance
m/z

Samples

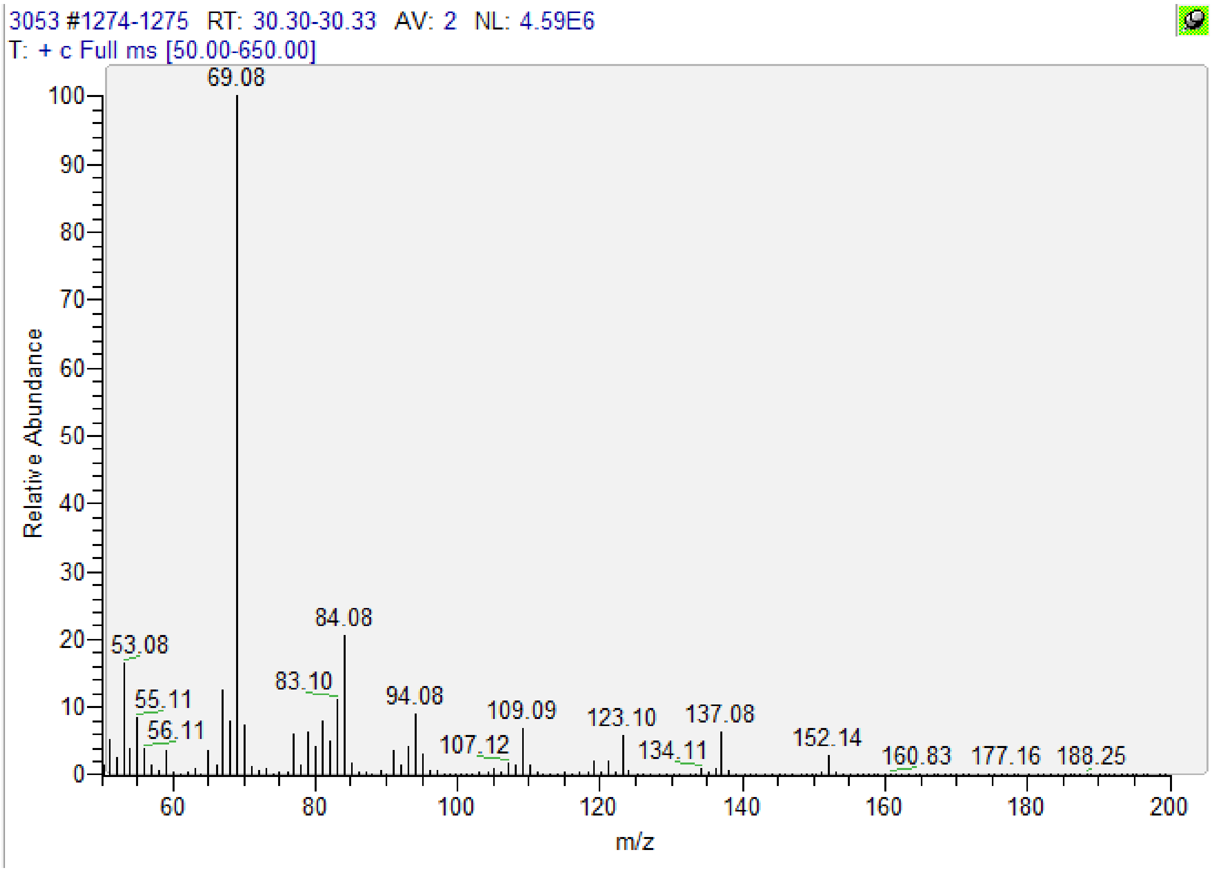
3053 #1274-1275 RT: 30.30-30.33 AV: 2 NL: 4.59E6
T: + c Full ms [50.00-650.00]
Relative Abundance
m/z
69.08
84.08
53.08
83.10
55.11
56.11
94.08
109.09
107.12
123.10
137.08
134.11
152.14
160.83
177.16
188.25

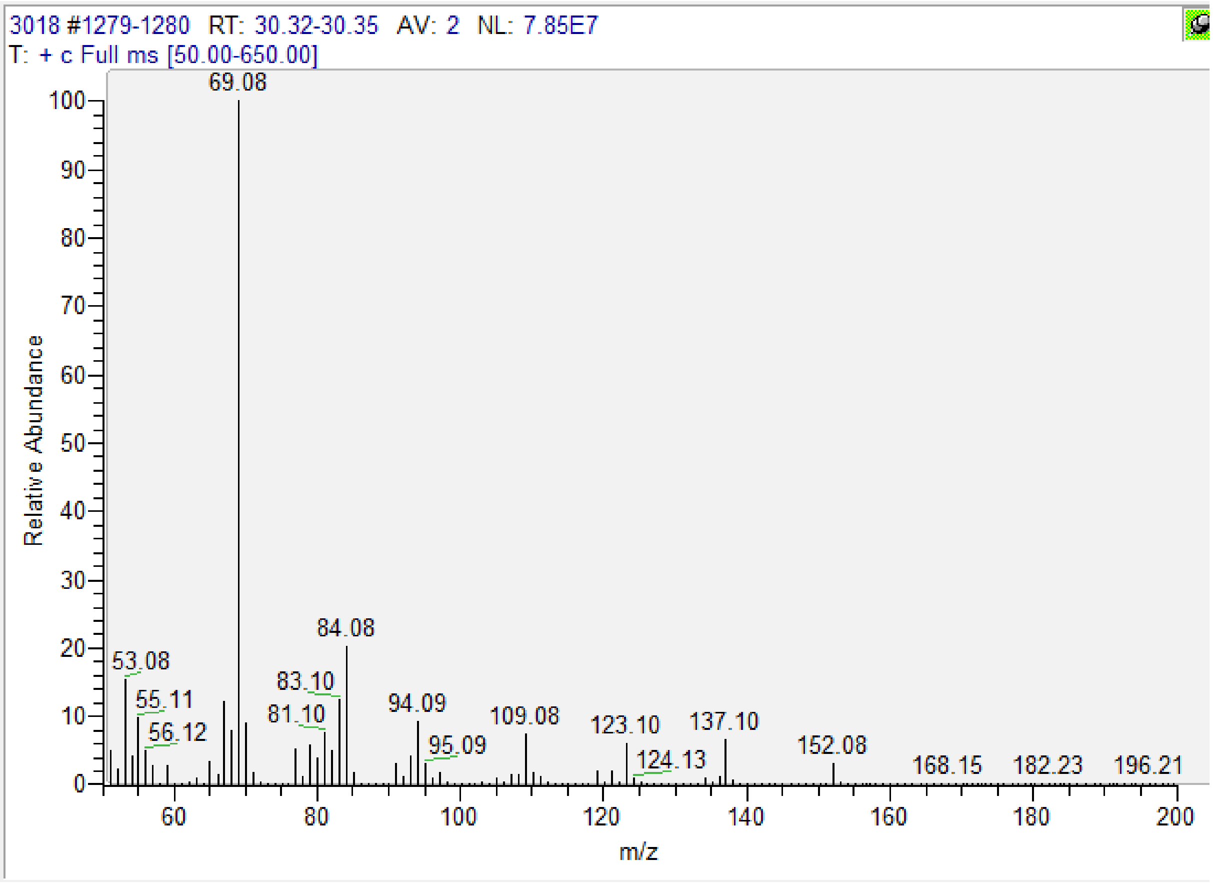
3018 #1279-1280 RT: 30.32-30.35 AV: 2 NL: 7.85E7
T: + c Full ms [50.00-650.00]
Relative Abundance
m/z
69.08
84.08
53.08
55.11
56.12
83.10
81.10
94.09
95.09
109.08
123.10
124.13
137.10
152.08
168.15
182.23
196.21

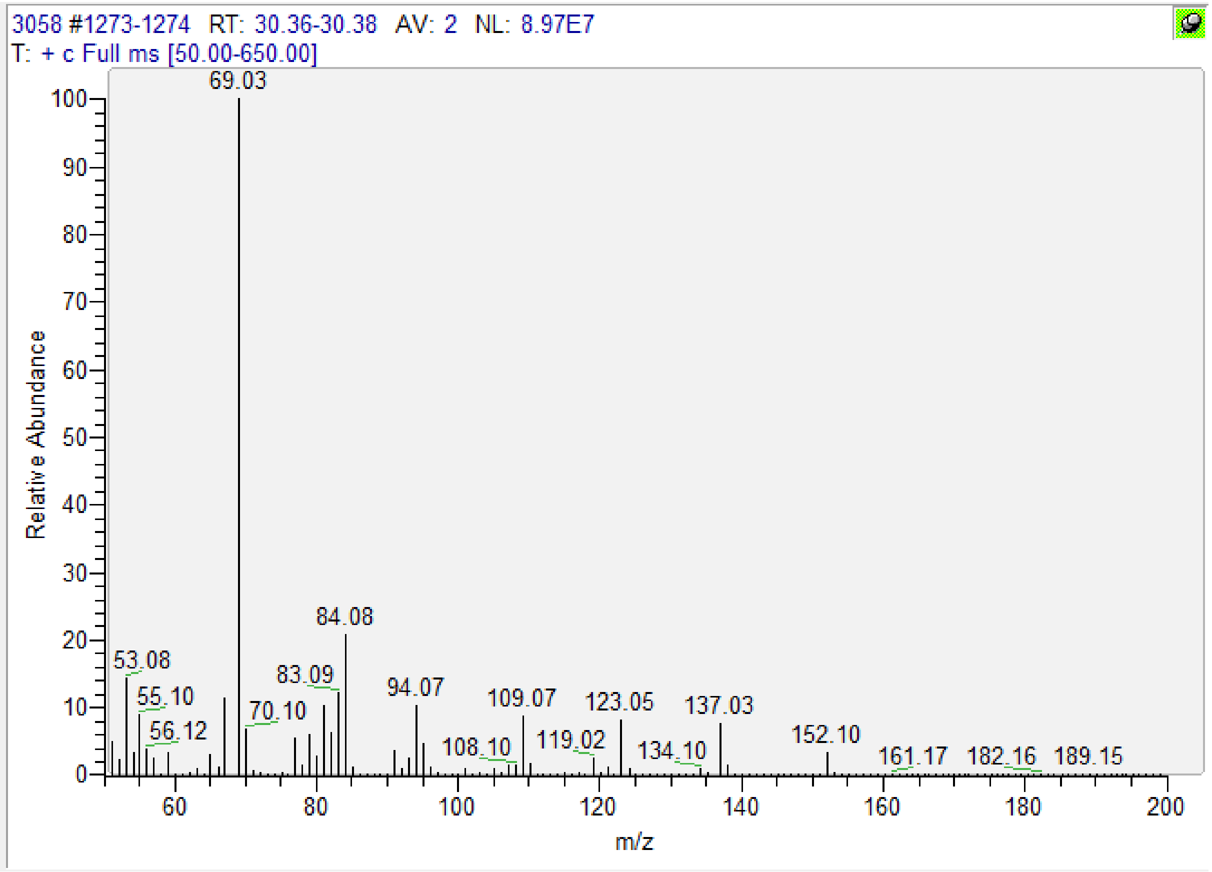

3058 #1273-1274 RT: 30.36-30.38 AV: 2 NL: 8.97E7
T: + c Full ms [50.00-650.00]
69.03
84.08
53.08
55.10
56.12
70.10
83.09
94.07
108.10
109.07
119.02
123.05
134.10
137.03
152.10
161.17
182.16
189.15
Relative Abundance
m/z

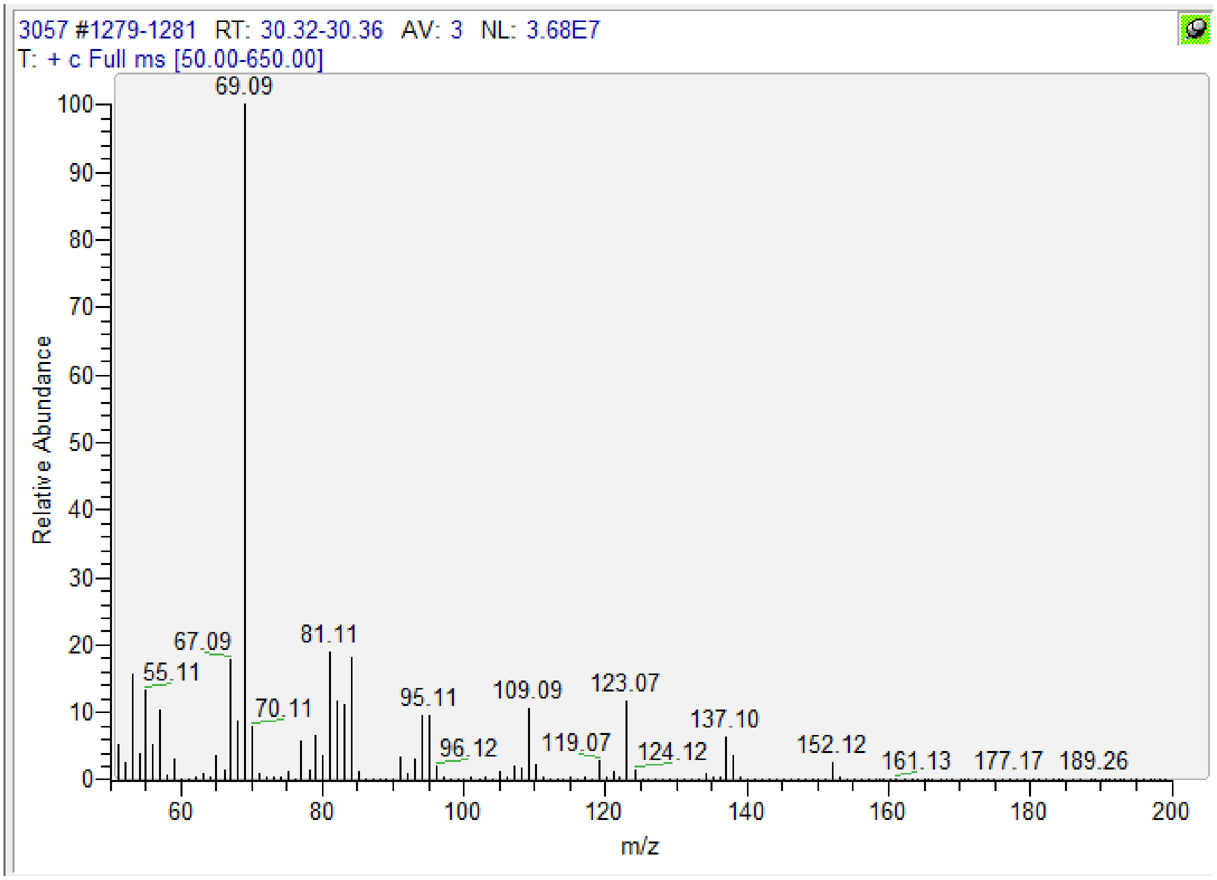

3057 #1279-1281 RT: 30.32-30.36 AV: 3 NL: 3.68E7
T: + c Full ms [50.00-650.00]
69.09
67.09
55.11
70.11
81.11
95.11
96.12
109.09
119.07
123.07
124.12
137.10
152.12
161.13
177.17
189.26
Relative Abundance
m/z

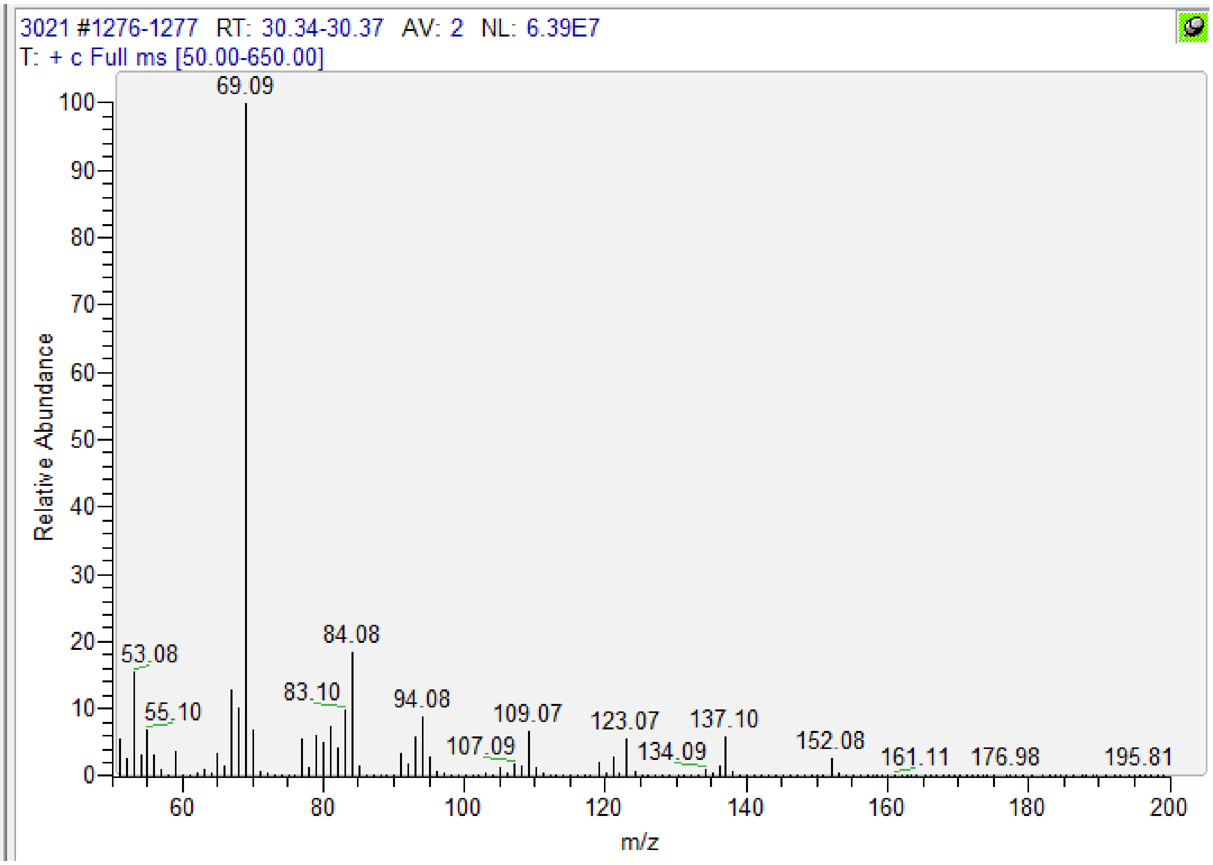
3021 #1276-1277 RT: 30.34-30.37 AV: 2 NL: 6.39E7
T: + c Full ms [50.00-650.00]
Relative Abundance
m/z
69.09
53.08
55.10
84.08
83.10
94.08
107.09
109.07
123.07
134.09
137.10
152.08
161.11
176.98
195.81

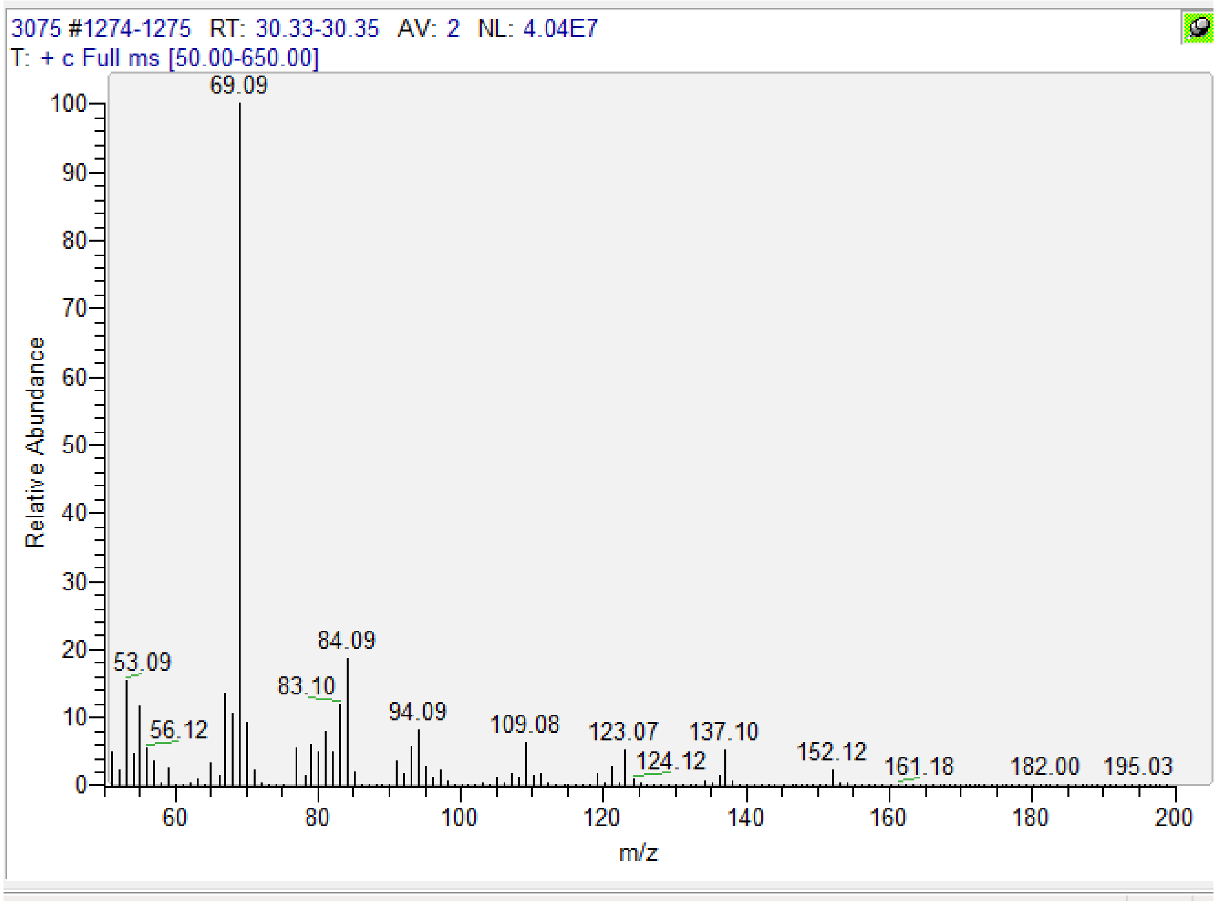
3075 #1274-1275 RT: 30.33-30.35 AV: 2 NL: 4.04E7
T: + c Full ms [50.00-650.00]
Relative Abundance
m/z
69.09
84.09
53.09
83.10
94.09
56.12
109.08
123.07
137.10
124.12
152.12
161.18
182.00
195.03

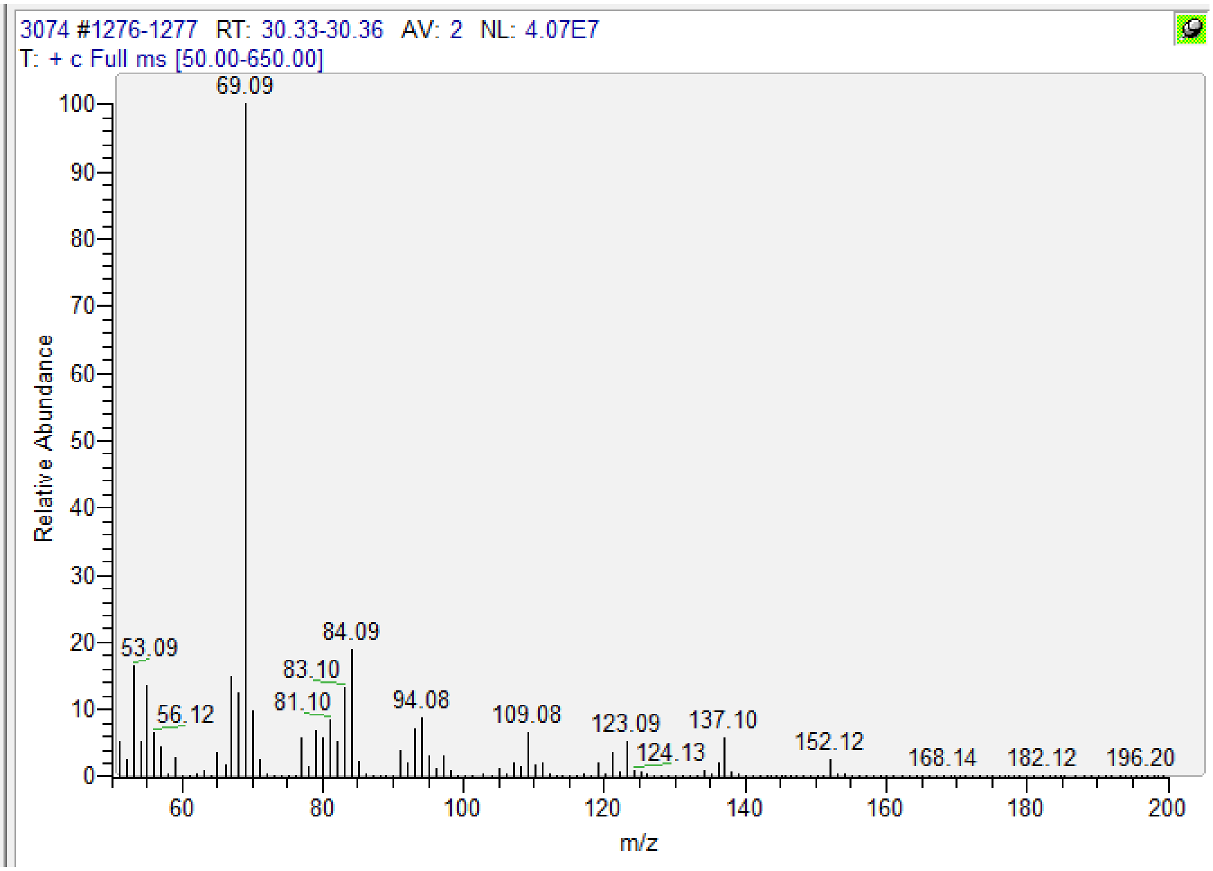

3074 #1276-1277 RT: 30.33-30.36 AV: 2 NL: 4.07E7
T: + c Full ms [50.00-650.00]
Relative Abundance
m/z
69.09
53.09
56.12
84.09
83.10
81.10
94.08
109.08
123.09
124.13
137.10
152.12
168.14
182.12
196.20

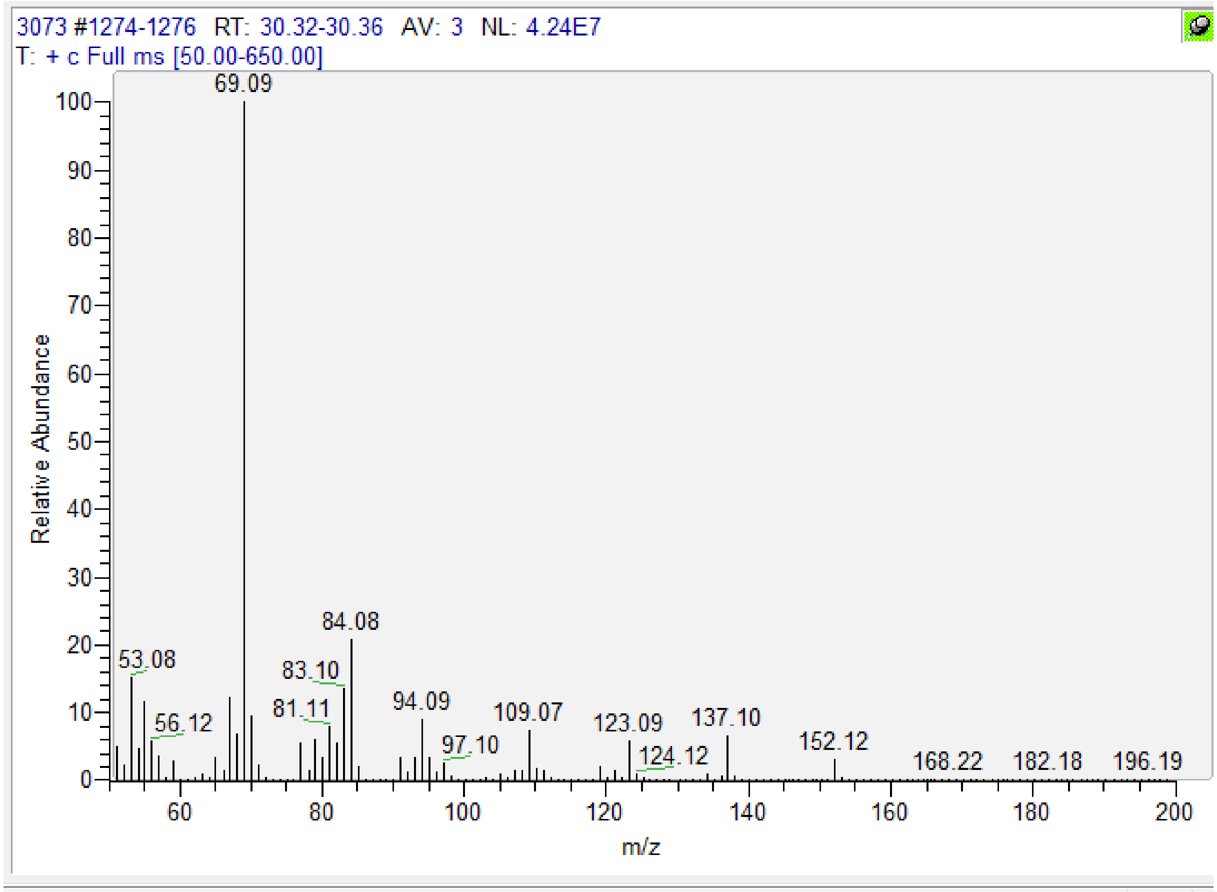
3073 #1274-1276 RT: 30.32-30.36 AV: 3 NL: 4.24E7
T: + c Full ms [50.00-650.00]
Relative Abundance
m/z
69.09
84.08
53.08
83.10
81.11
56.12
94.09
109.07
97.10
123.09
137.10
124.12
152.12
168.22
182.18
196.19

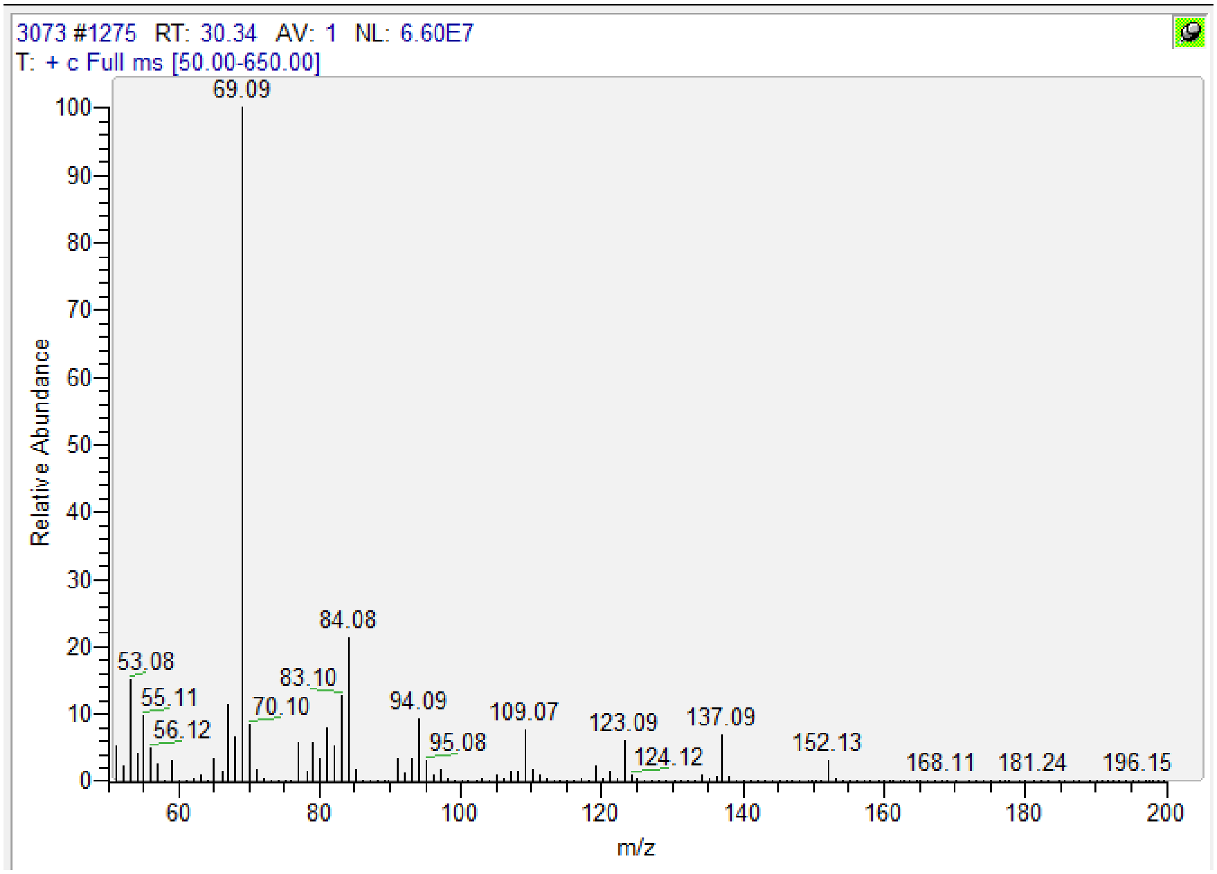
3073 #1275 RT: 30.34 AV: 1 NL: 6.60E7
T: + c Full ms [50.00-650.00]
69.09
84.08
53.08
55.11
56.12
83.10
70.10
94.09
95.08
109.07
123.09
124.12
137.09
152.13
168.11
181.24
196.15
Relative Abundance
m/z

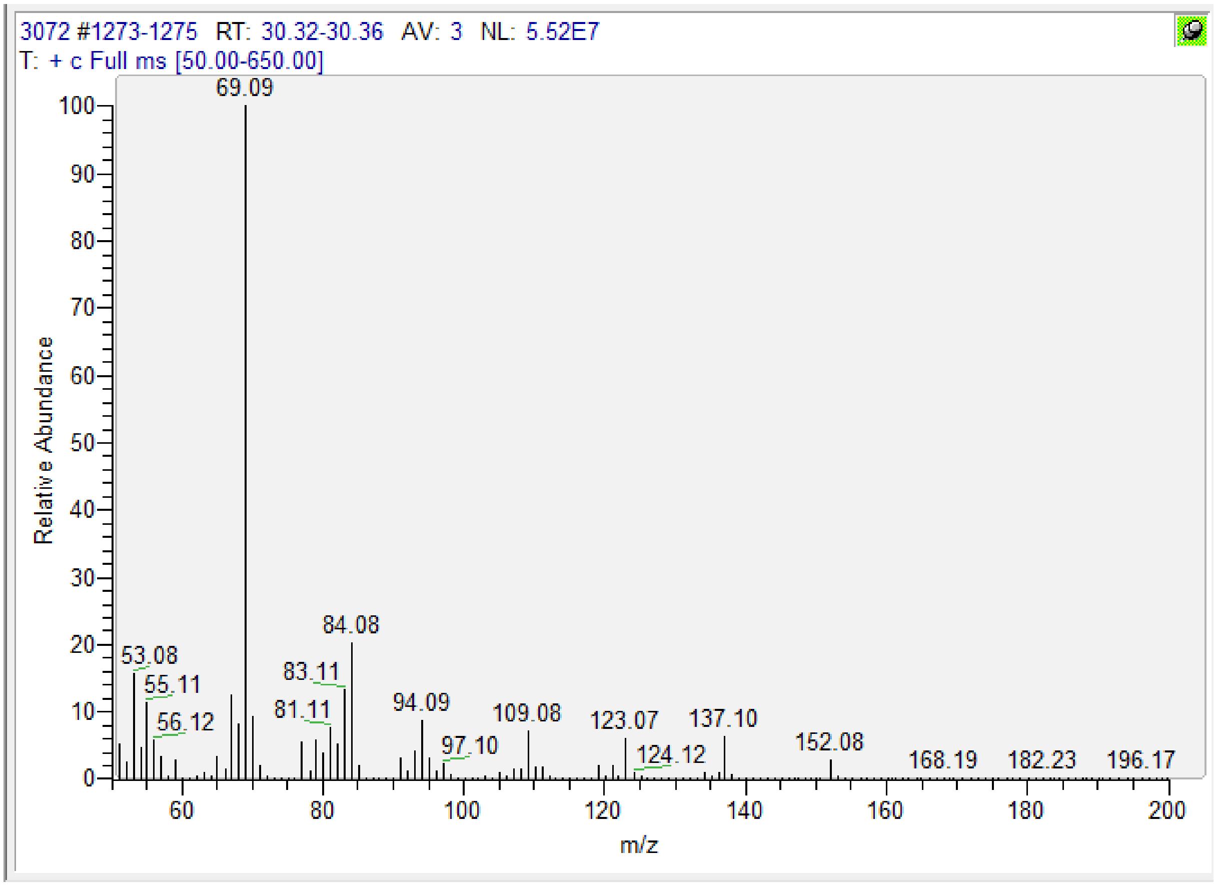
3072 #1273-1275 RT: 30.32-30.36 AV: 3 NL: 5.52E7
T: + c Full ms [50.00-650.00]
Relative Abundance
m/z
69.09
84.08
53.08
55.11
56.12
83.11
81.11
94.09
97.10
109.08
123.07
124.12
137.10
152.08
168.19
182.23
196.17

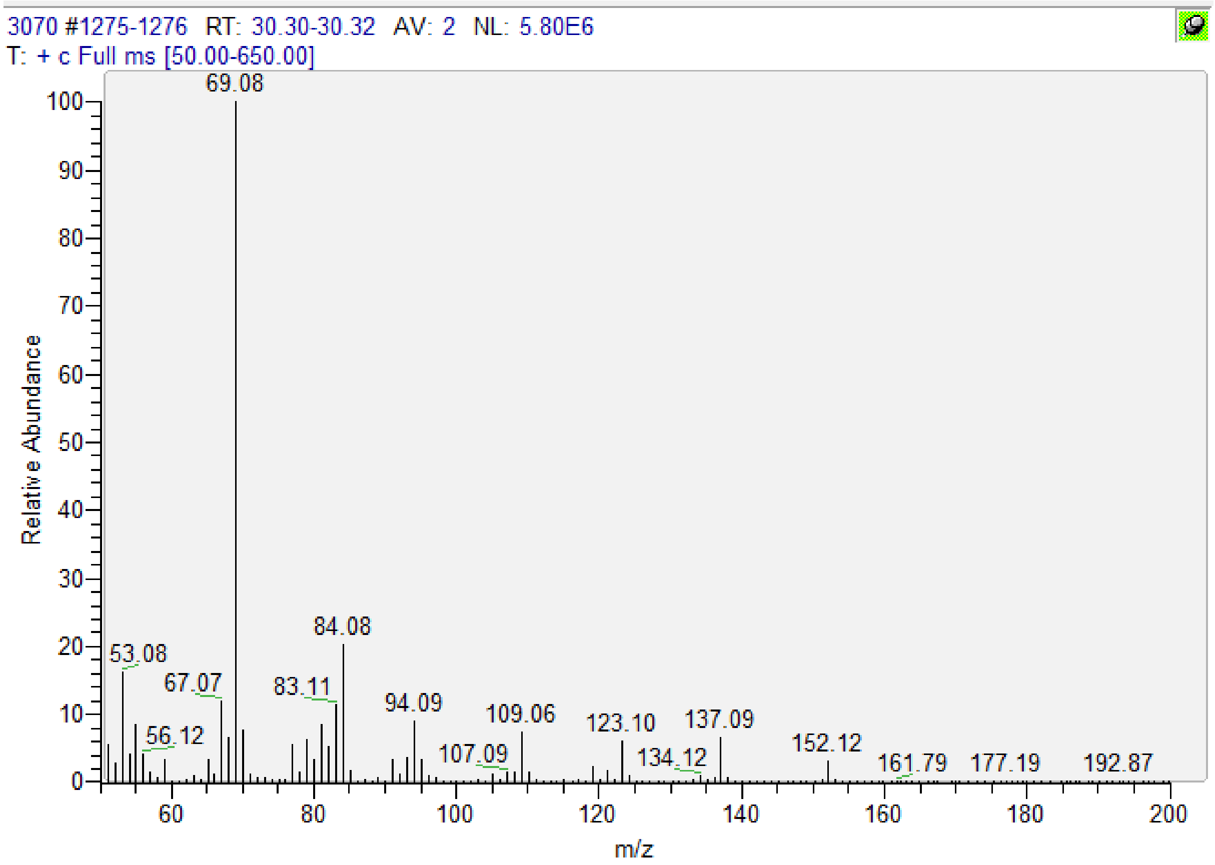
3070 #1275-1276 RT: 30.30-30.32 AV: 2 NL: 5.80E6
T: + c Full ms [50.00-650.00]
69.08
84.08
53.08
67.07
83.11
56.12
94.09
109.06
123.10
137.09
107.09
134.12
152.12
161.79
177.19
192.87
Relative Abundance
m/z
0
10
20
30
40
50
60
70
80
90
100
60
80
100
120
140
160
180
200

Geranyl acetat

Reference

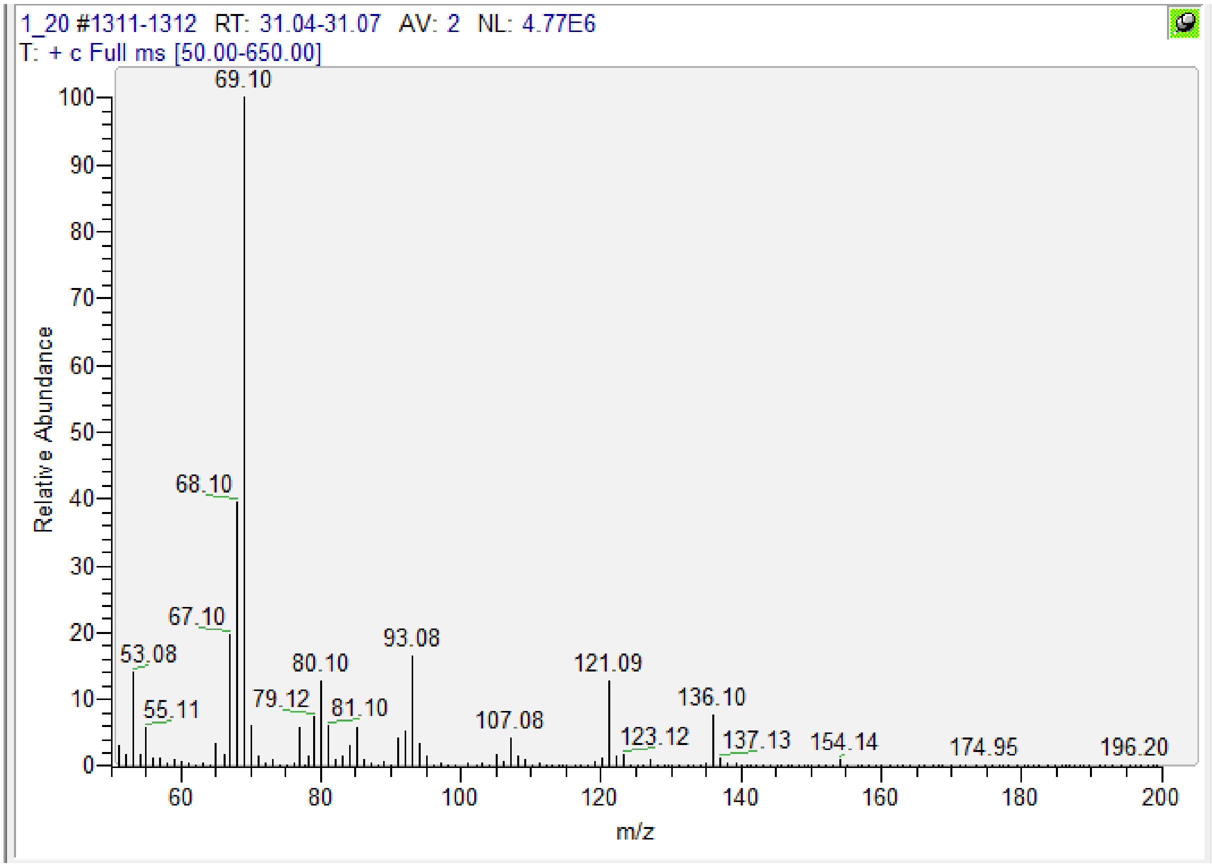

Samples:

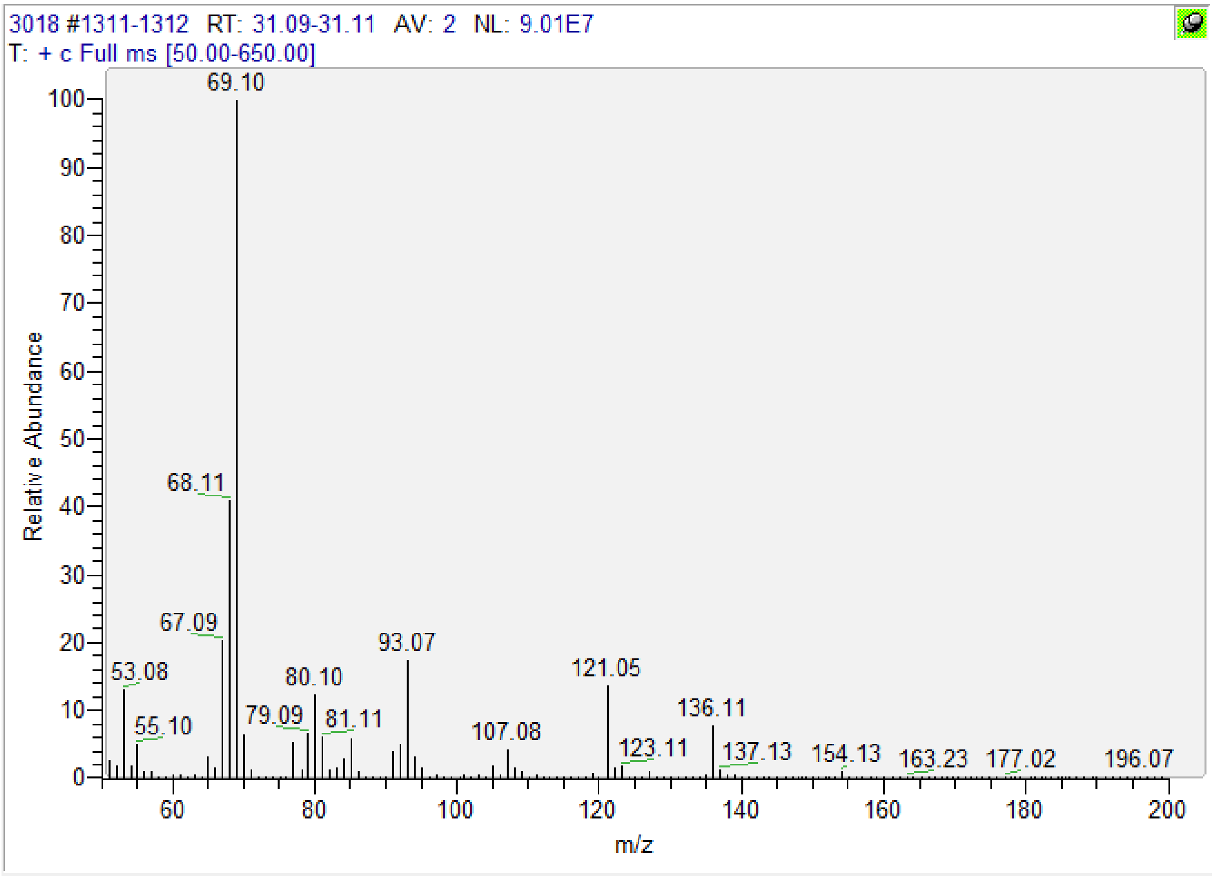

3018 #1311-1312 RT: 31.09-31.11 AV: 2 NL: 9.01E7
T: + c Full ms [50.00-650.00]
69.10
68.11
67.09
53.08
55.10
79.09
80.10
81.11
93.07
107.08
121.05
123.11
136.11
137.13
154.13
163.23
177.02
196.07
Relative Abundance
m/z

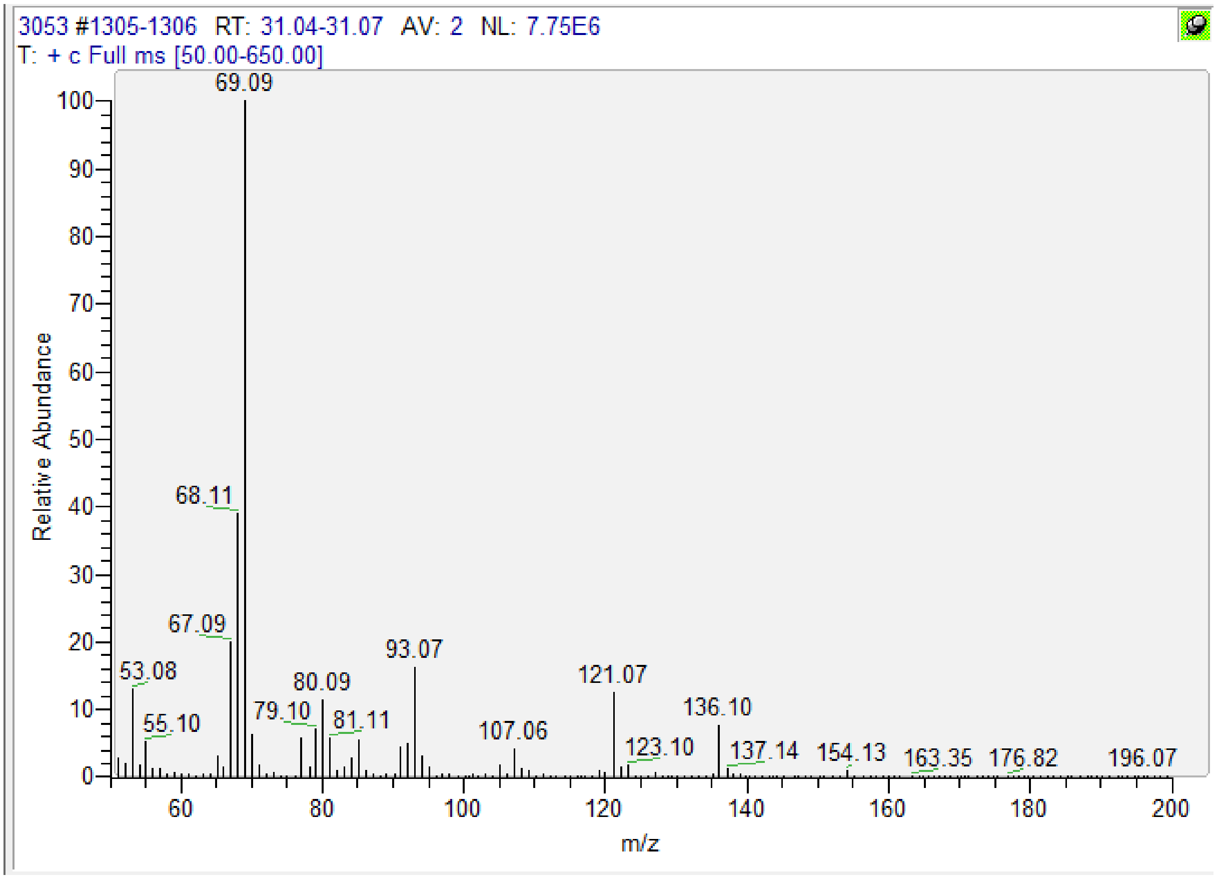
3053 #1305-1306 RT: 31.04-31.07 AV: 2 NL: 7.75E6
T: + c Full ms [50.00-650.00]
69.09
68.11
67.09
53.08
55.10
79.10
80.09
81.11
93.07
107.06
121.07
123.10
136.10
137.14
154.13
163.35
176.82
196.07
Relative Abundance
m/z

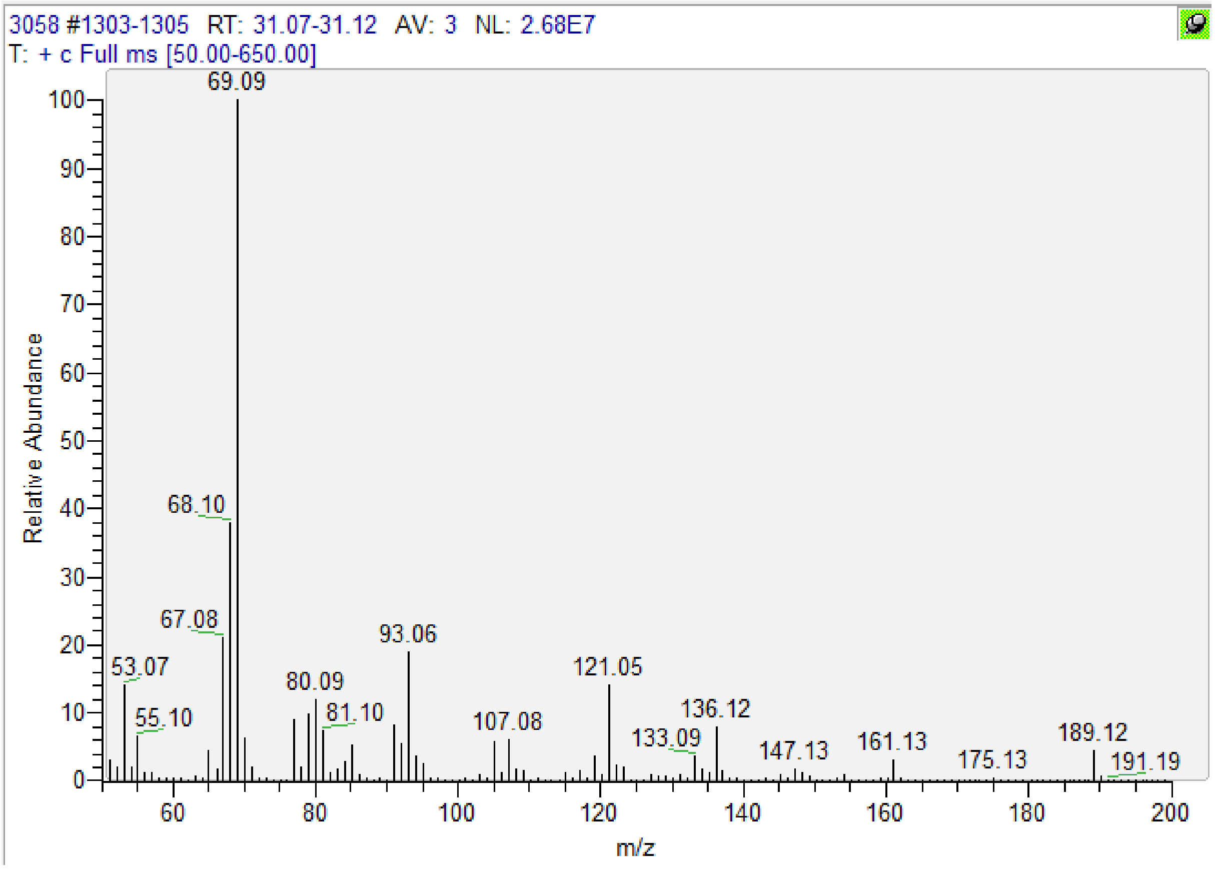
3058 #1303-1305 RT: 31.07-31.12 AV: 3 NL: 2.68E7
T: + c Full ms [50.00-650.00]
Relative Abundance
m/z
69.09
68.10
67.08
53.07
55.10
80.09
81.10
93.06
107.08
121.05
133.09
136.12
147.13
161.13
175.13
189.12
191.19

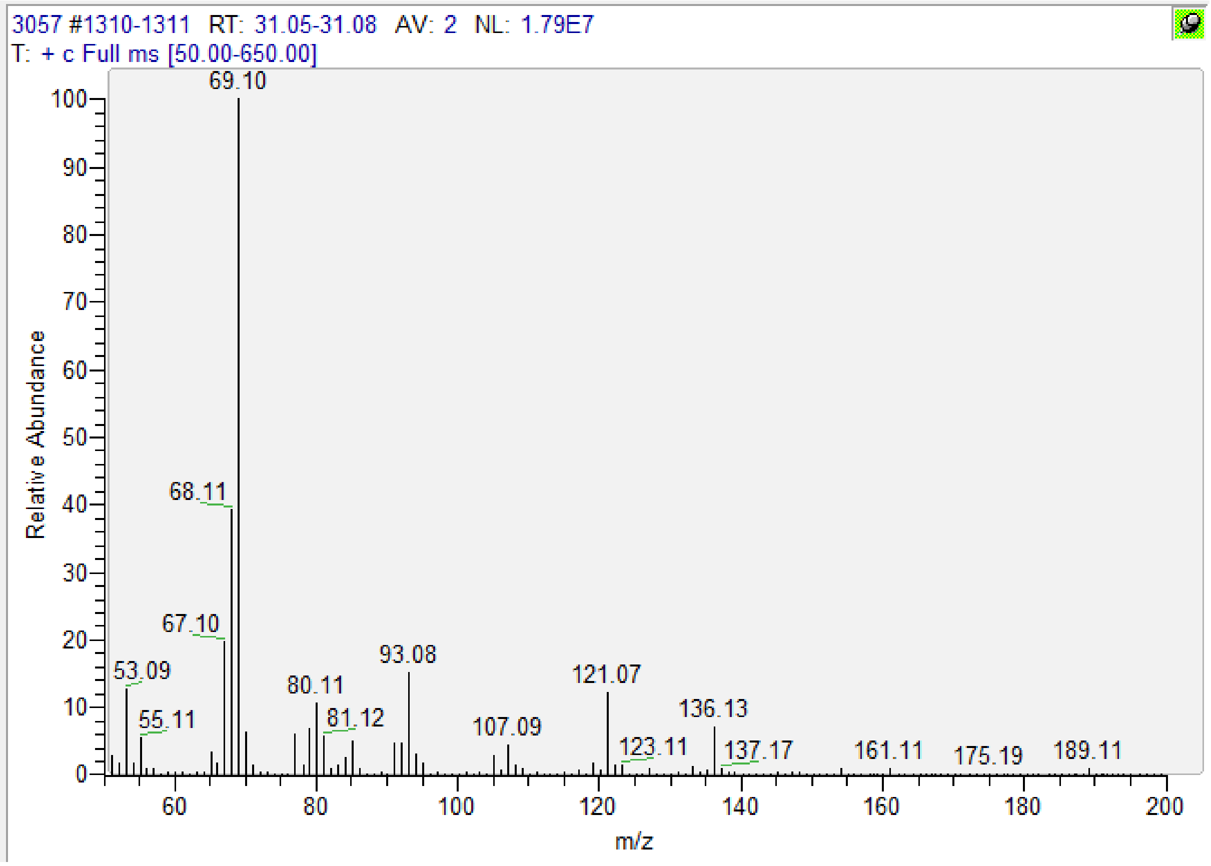
3057 #1310-1311 RT: 31.05-31.08 AV: 2 NL: 1.79E7
T: + c Full ms [50.00-650.00]
69.10
68.11
67.10
53.09
55.11
80.11
81.12
93.08
107.09
121.07
123.11
136.13
137.17
161.11
175.19
189.11
Relative Abundance
m/z

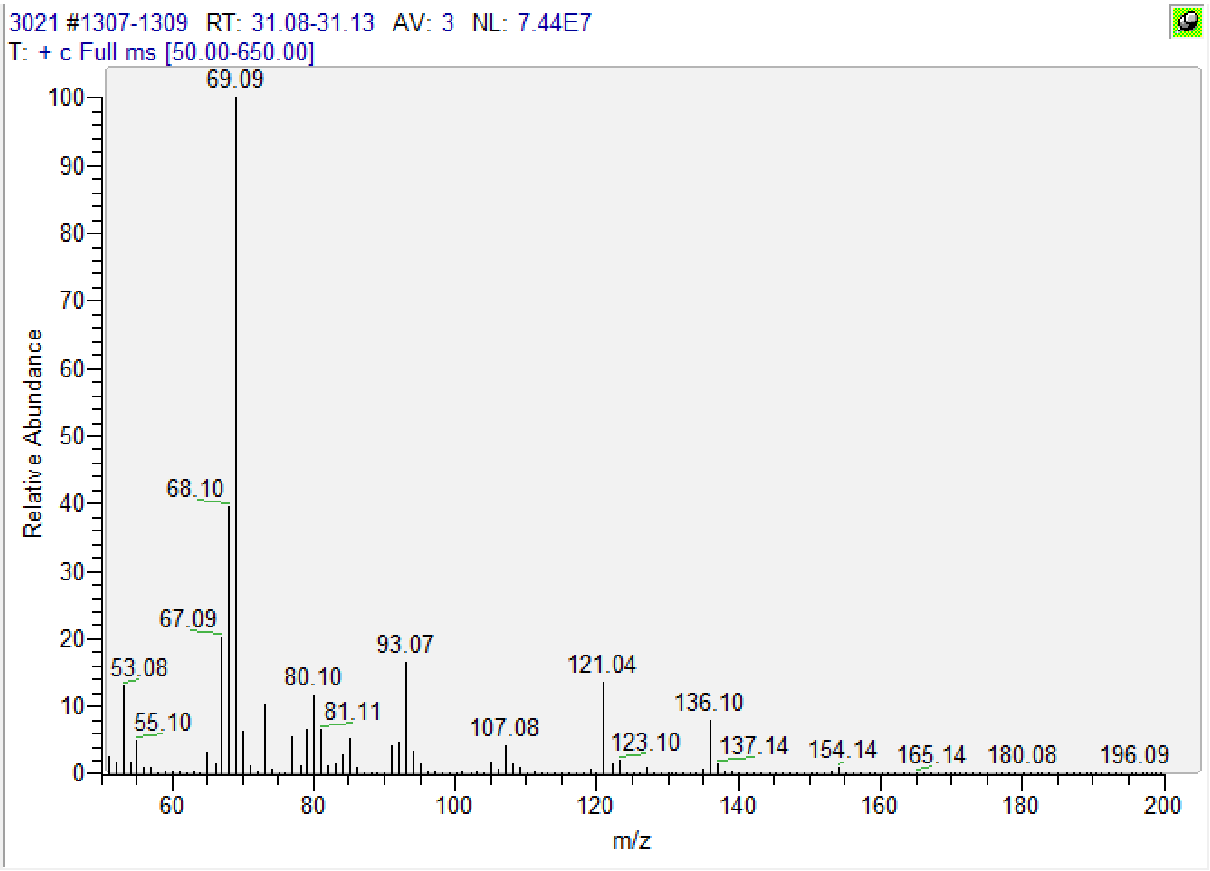
3021 #1307-1309 RT: 31.08-31.13 AV: 3 NL: 7.44E7
T: + c Full ms [50.00-650.00]
Relative Abundance
m/z
69.09
68.10
67.09
53.08
55.10
80.10
81.11
93.07
107.08
121.04
123.10
136.10
137.14
154.14
165.14
180.08
196.09
0
10
20
30
40
50
60
70
80
90
100
60
80
100
120
140
160
180
200

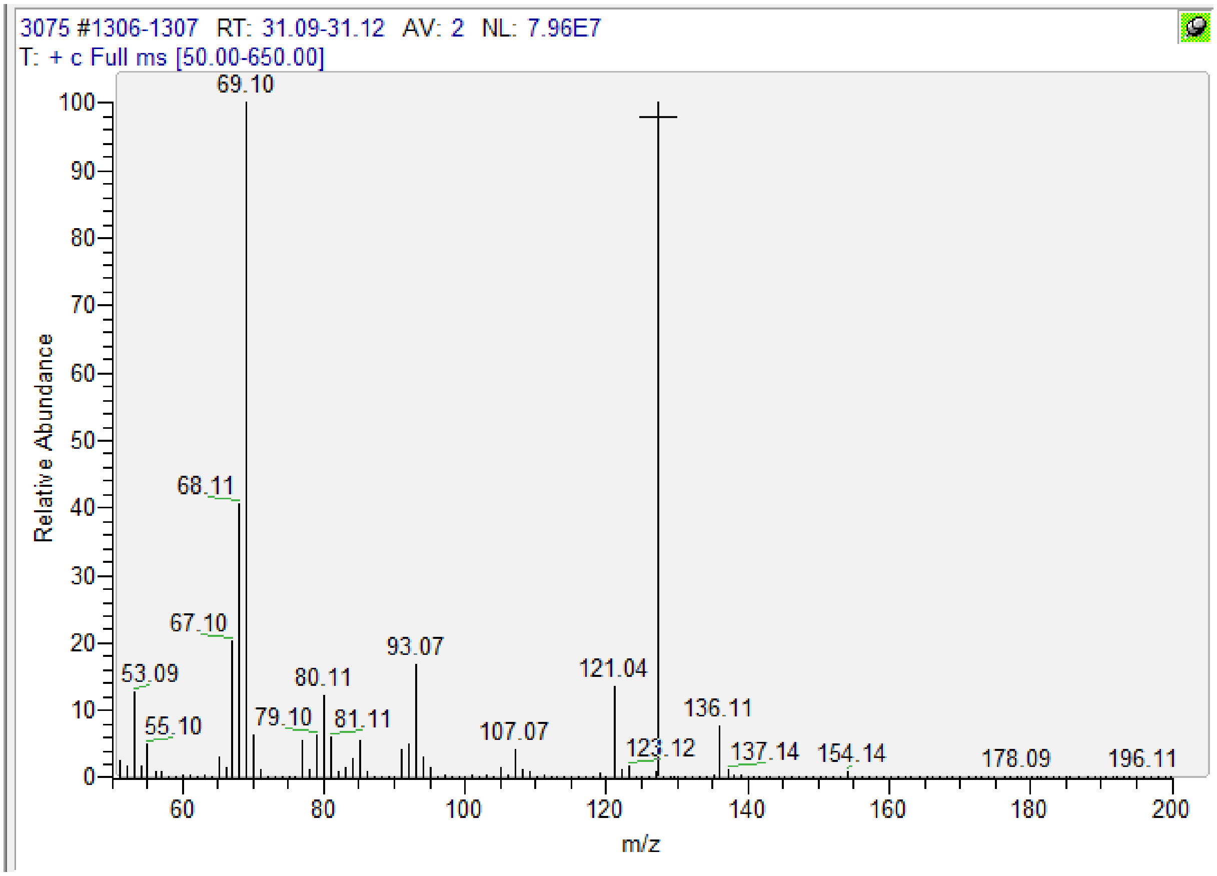
3075 #1306-1307 RT: 31.09-31.12 AV: 2 NL: 7.96E7
T: + c Full ms [50.00-650.00]
Relative Abundance
m/z
69.10
68.11
67.10
53.09
55.10
79.10
80.11
81.11
93.07
107.07
121.04
123.12
136.11
137.14
154.14
178.09
196.11

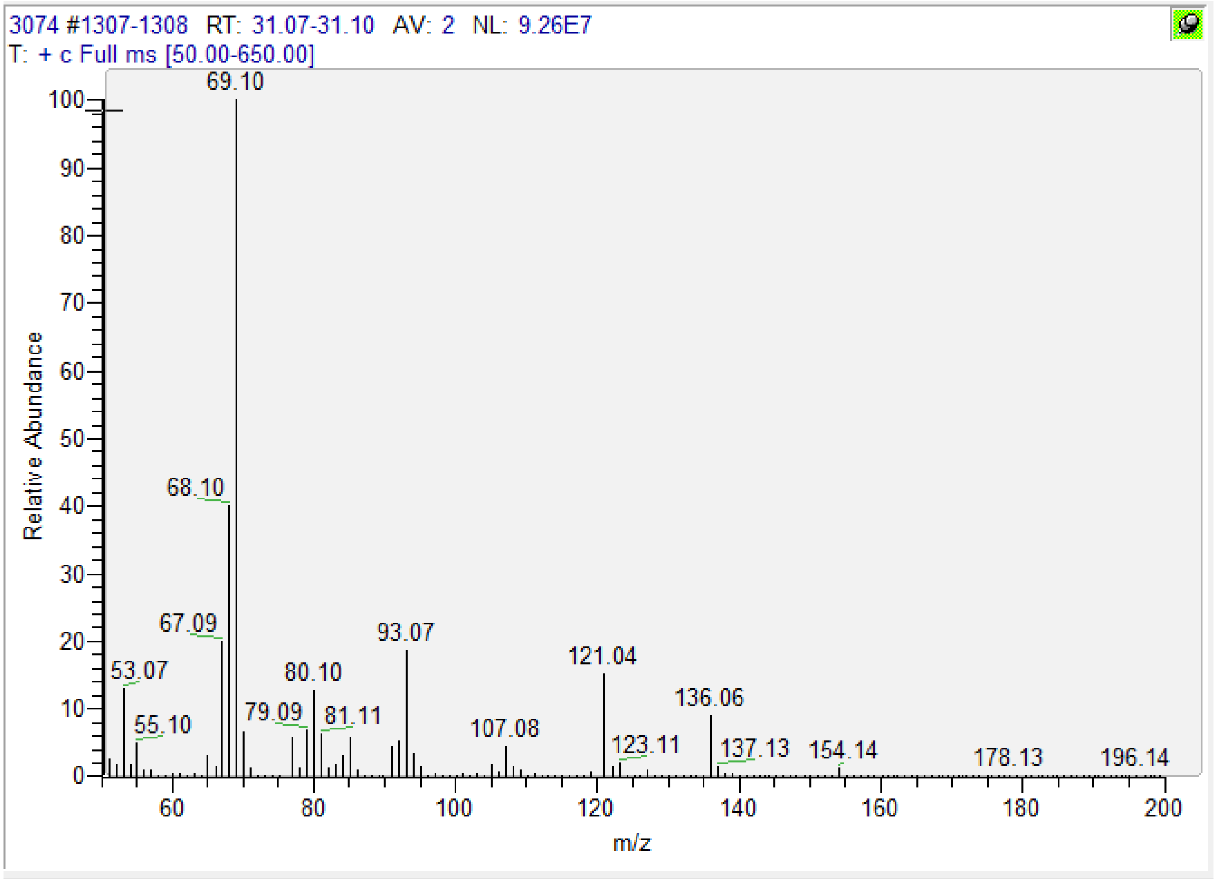
3074 #1307-1308 RT: 31.07-31.10 AV: 2 NL: 9.26E7
T: + c Full ms [50.00-650.00]
Relative Abundance
m/z
69.10
68.10
67.09
53.07
55.10
79.09
80.10
81.11
93.07
107.08
121.04
123.11
136.06
137.13
154.14
178.13
196.14

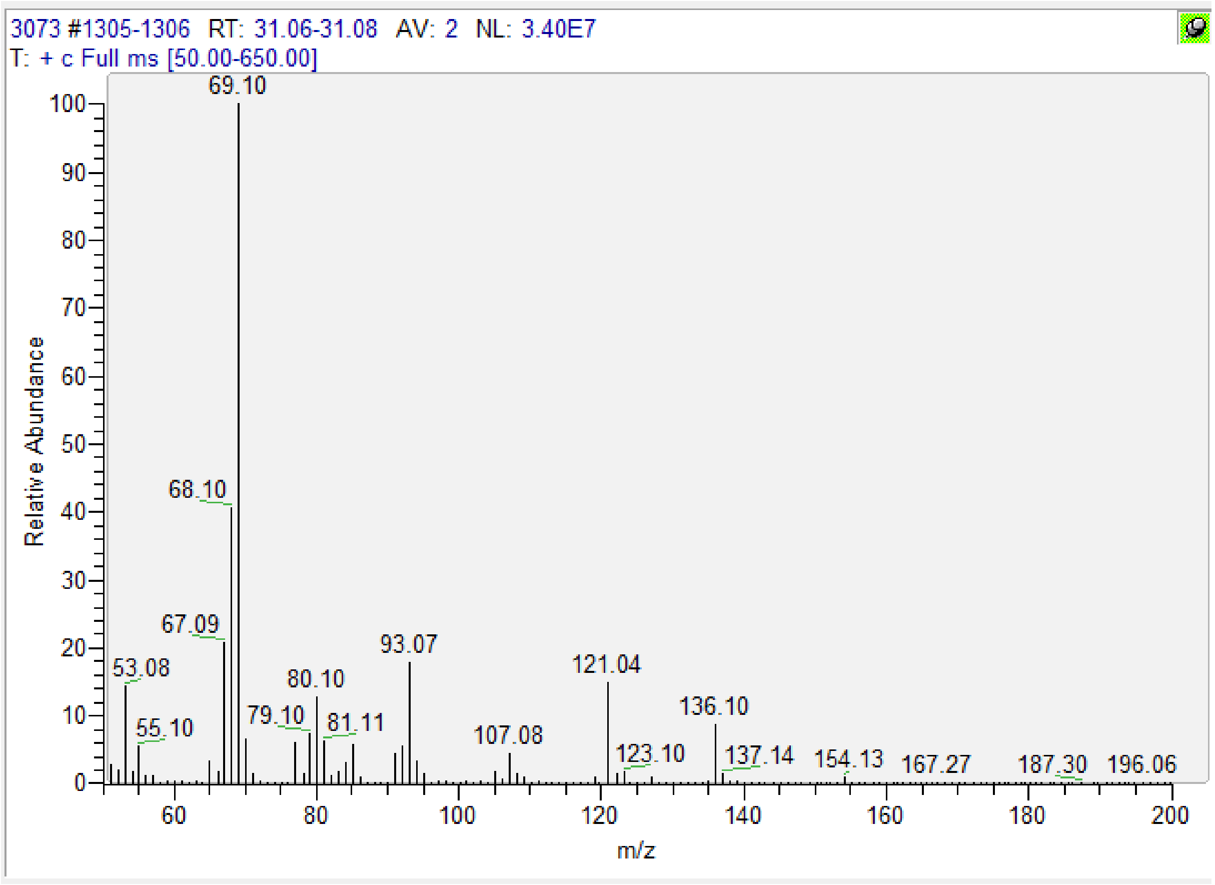

3073 #1305-1306 RT: 31.06-31.08 AV: 2 NL: 3.40E7
T: + c Full ms [50.00-650.00]
Relative Abundance
100
90
80
70
60
50
40
30
20
10
0
69.10
68.10
67.09
53.08
55.10
79.10
80.10
81.11
93.07
107.08
121.04
123.10
136.10
137.14
154.13
167.27
187.30
196.06
60
80
100
120
140
160
180
200
m/z

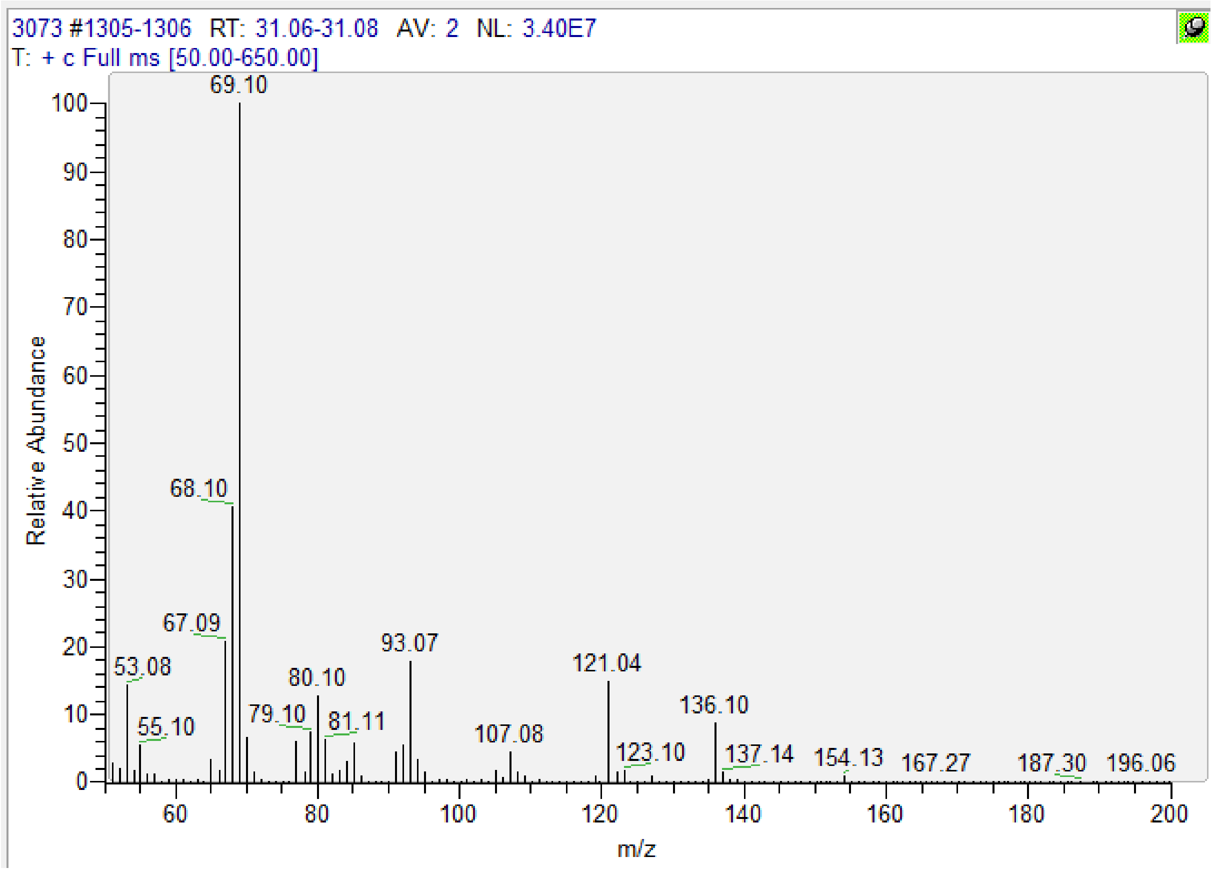
3073 #1305-1306 RT: 31.06-31.08 AV: 2 NL: 3.40E7
T: + c Full ms [50.00-650.00]
Relative Abundance
m/z
69.10
68.10
67.09
53.08
55.10
79.10
80.10
81.11
93.07
107.08
121.04
123.10
136.10
137.14
154.13
167.27
187.30
196.06

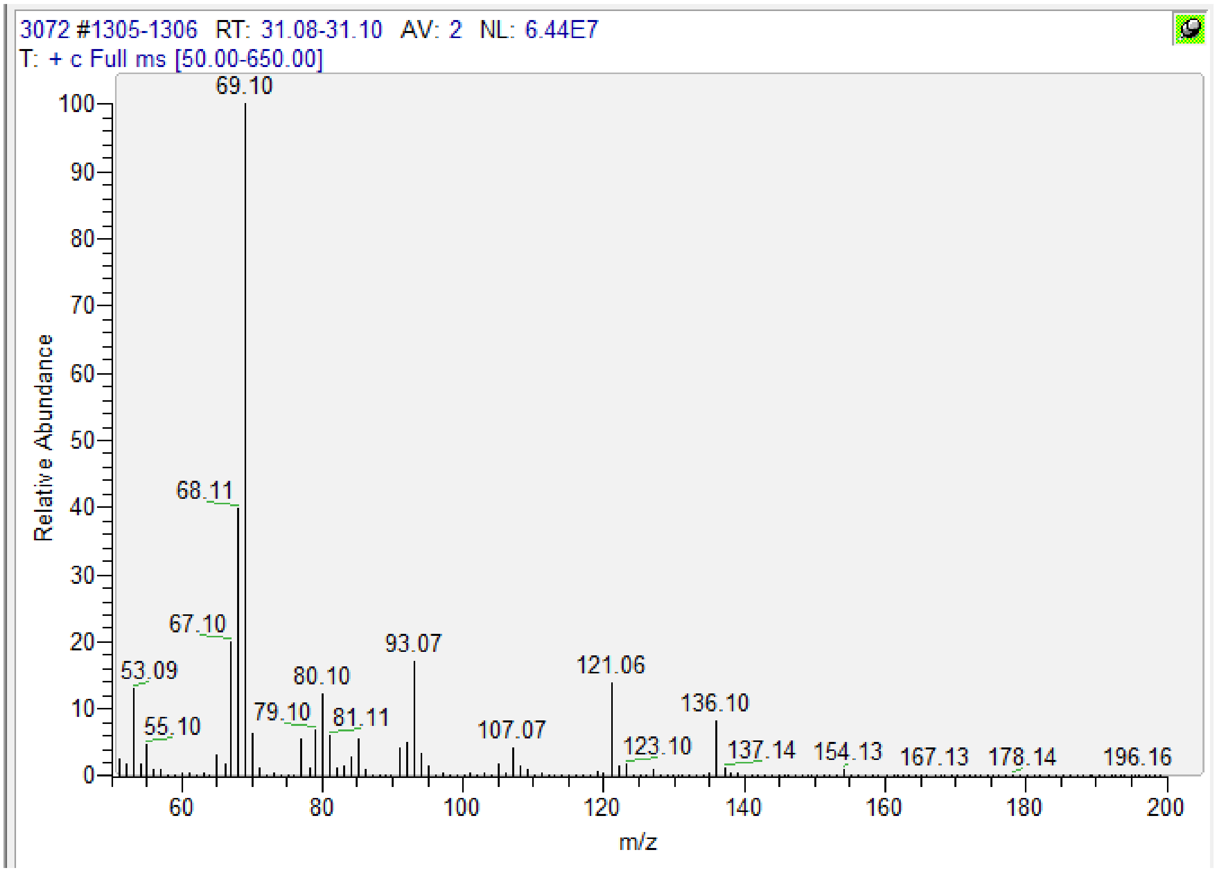
3072 #1305-1306 RT: 31.08-31.10 AV: 2 NL: 6.44E7
T: + c Full ms [50.00-650.00]
Relative Abundance
m/z
69.10
68.11
67.10
53.09
55.10
79.10
80.10
81.11
93.07
107.07
121.06
123.10
136.10
137.14
154.13
167.13
178.14
196.16

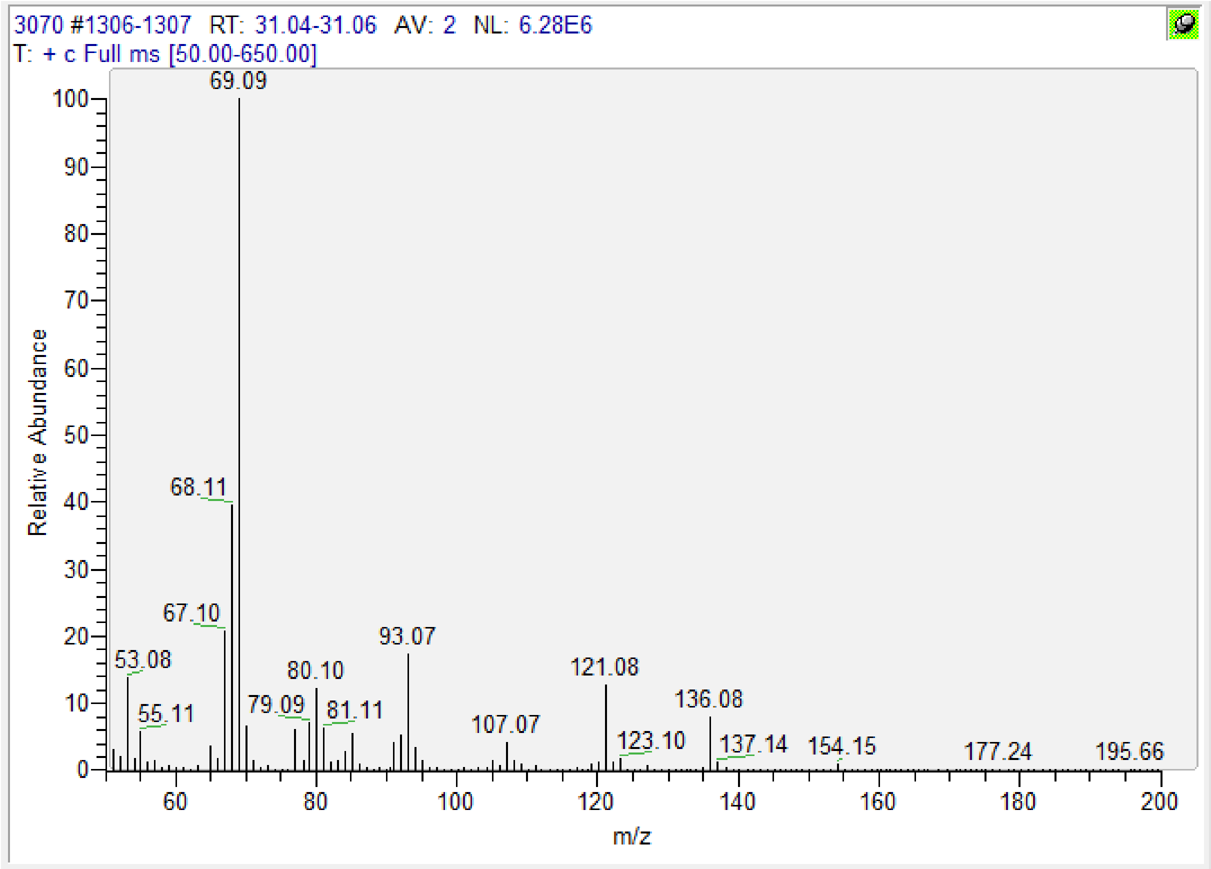
3070 #1306-1307 RT: 31.04-31.06 AV: 2 NL: 6.28E6
T: + c Full ms [50.00-650.00]
Relative Abundance
m/z
69.09
68.11
67.10
53.08
55.11
79.09
80.10
81.11
93.07
107.07
121.08
123.10
136.08
137.14
154.15
177.24
195.66

Citronellol

Reference:

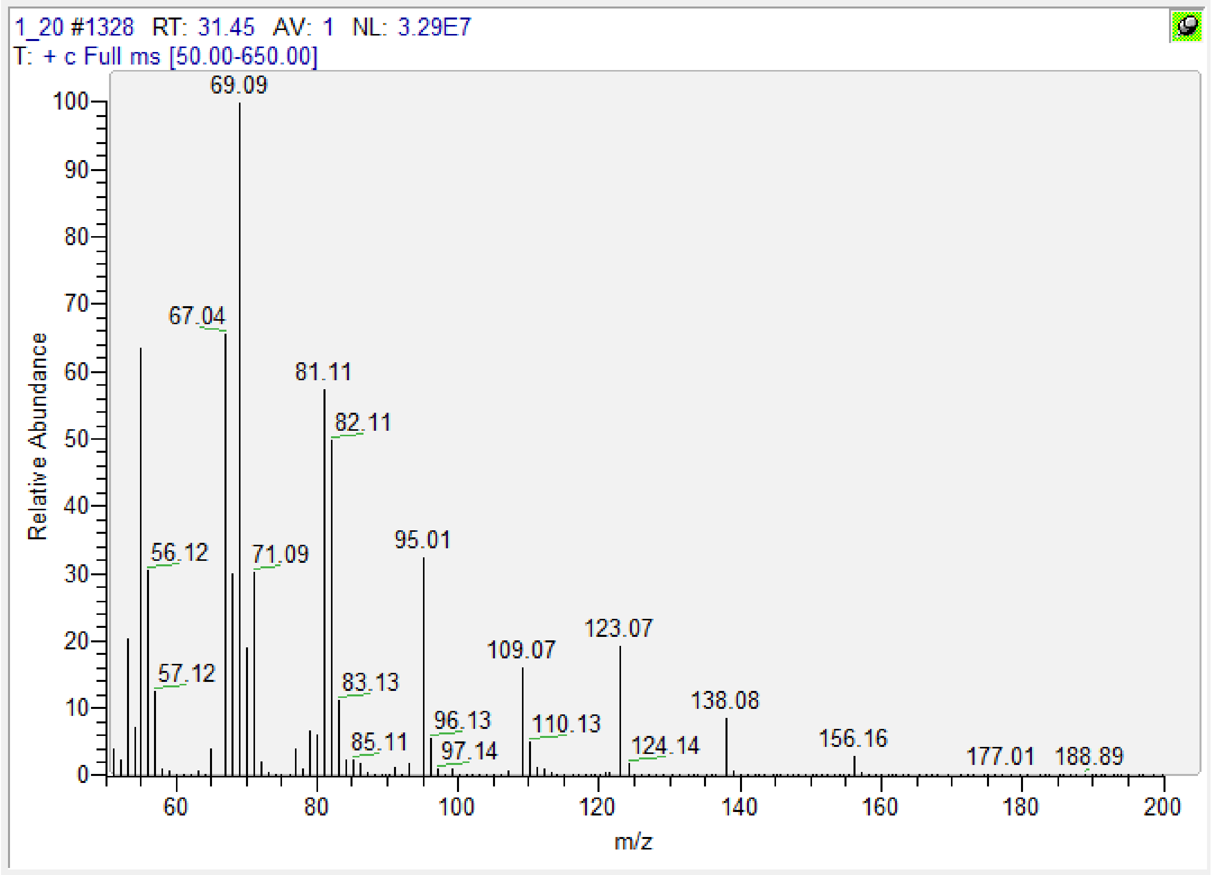

Samples:

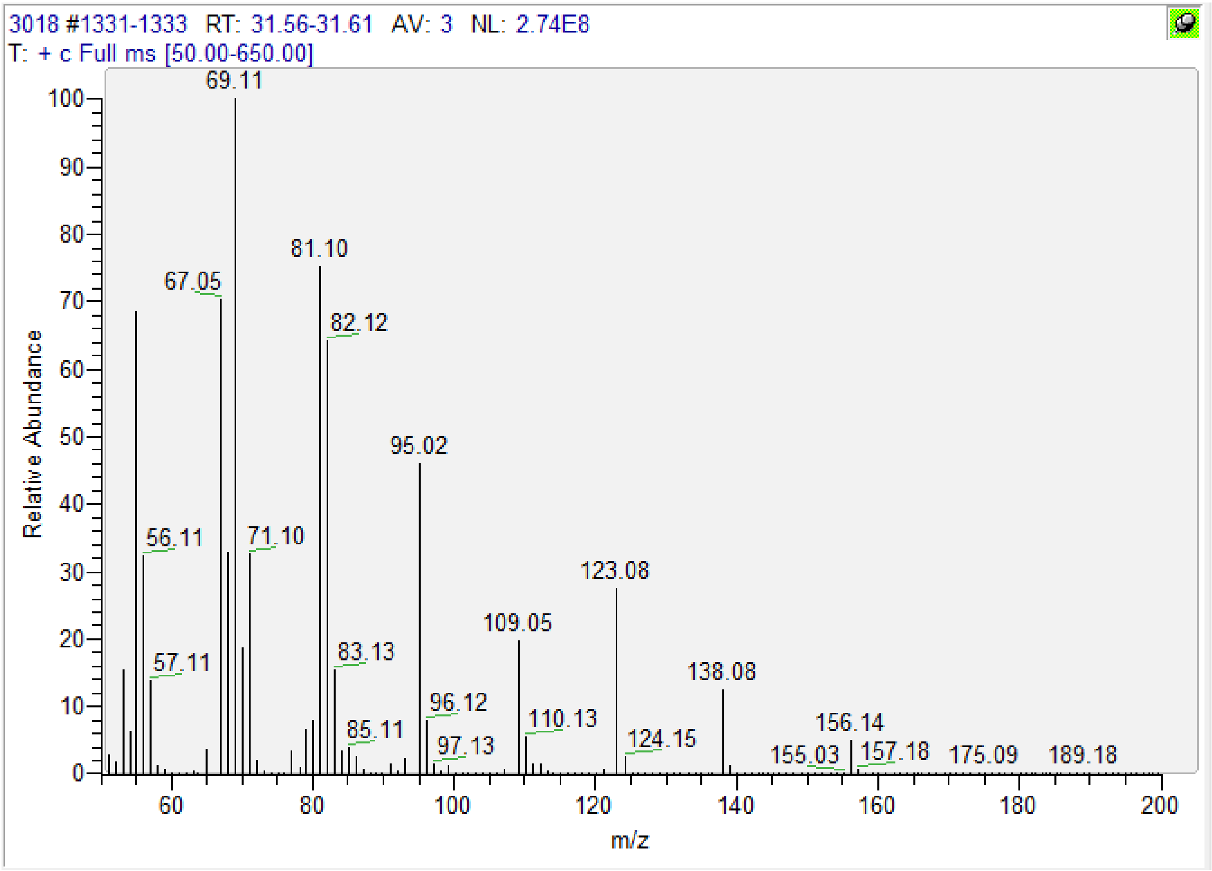

3018 #1331-1333 RT: 31.56-31.61 AV: 3 NL: 2.74E8
T: + c Full ms [50.00-650.00]
Relative Abundance
m/z
69.11
81.10
67.05
82.12
95.02
56.11
71.10
123.08
109.05
138.08
57.11
83.13
96.12
85.11
110.13
97.13
124.15
156.14
155.03
157.18
175.09
189.18

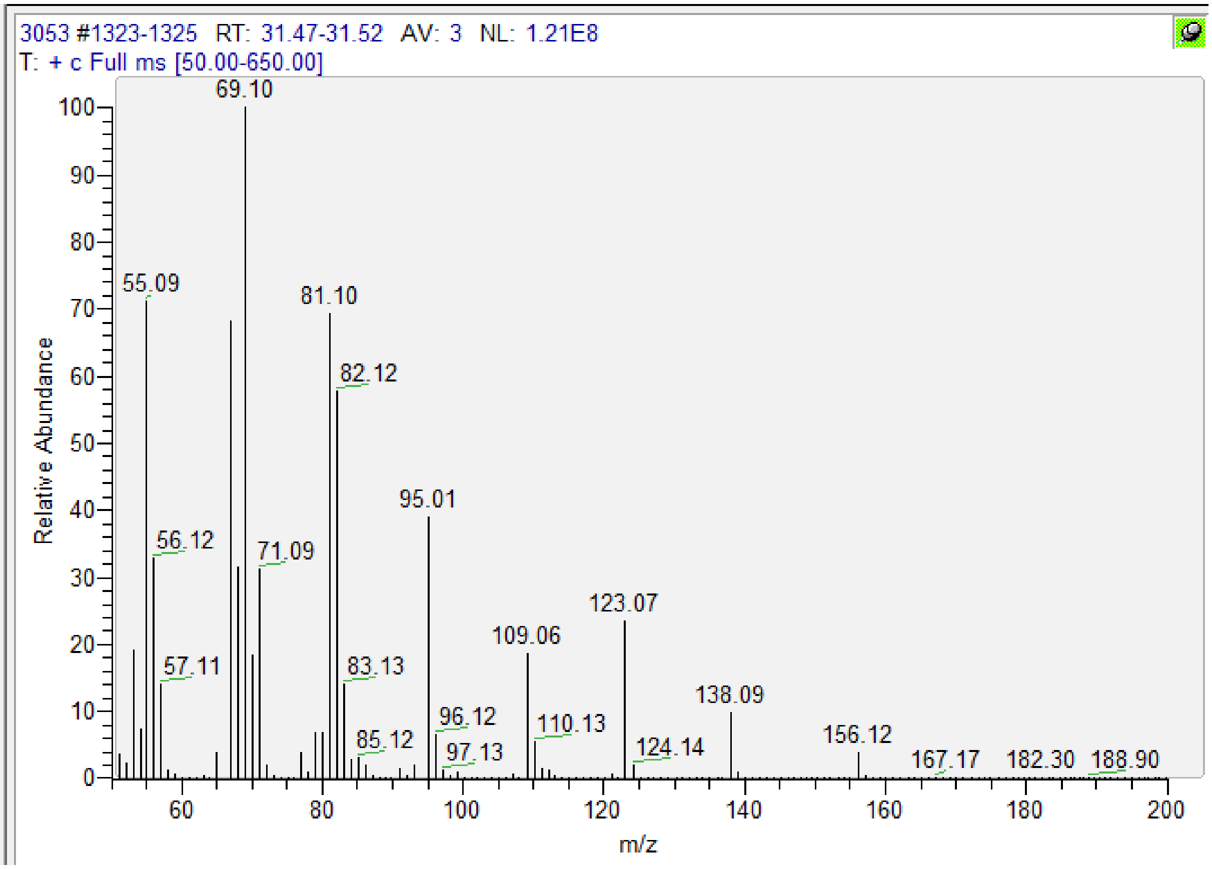
3053 #1323-1325 RT: 31.47-31.52 AV: 3 NL: 1.21E8
T: + c Full ms [50.00-650.00]
Relative Abundance
m/z
69.10
55.09
81.10
82.12
95.01
56.12
71.09
123.07
109.06
57.11
83.13
138.09
96.12
110.13
85.12
124.14
97.13
156.12
167.17
182.30
188.90

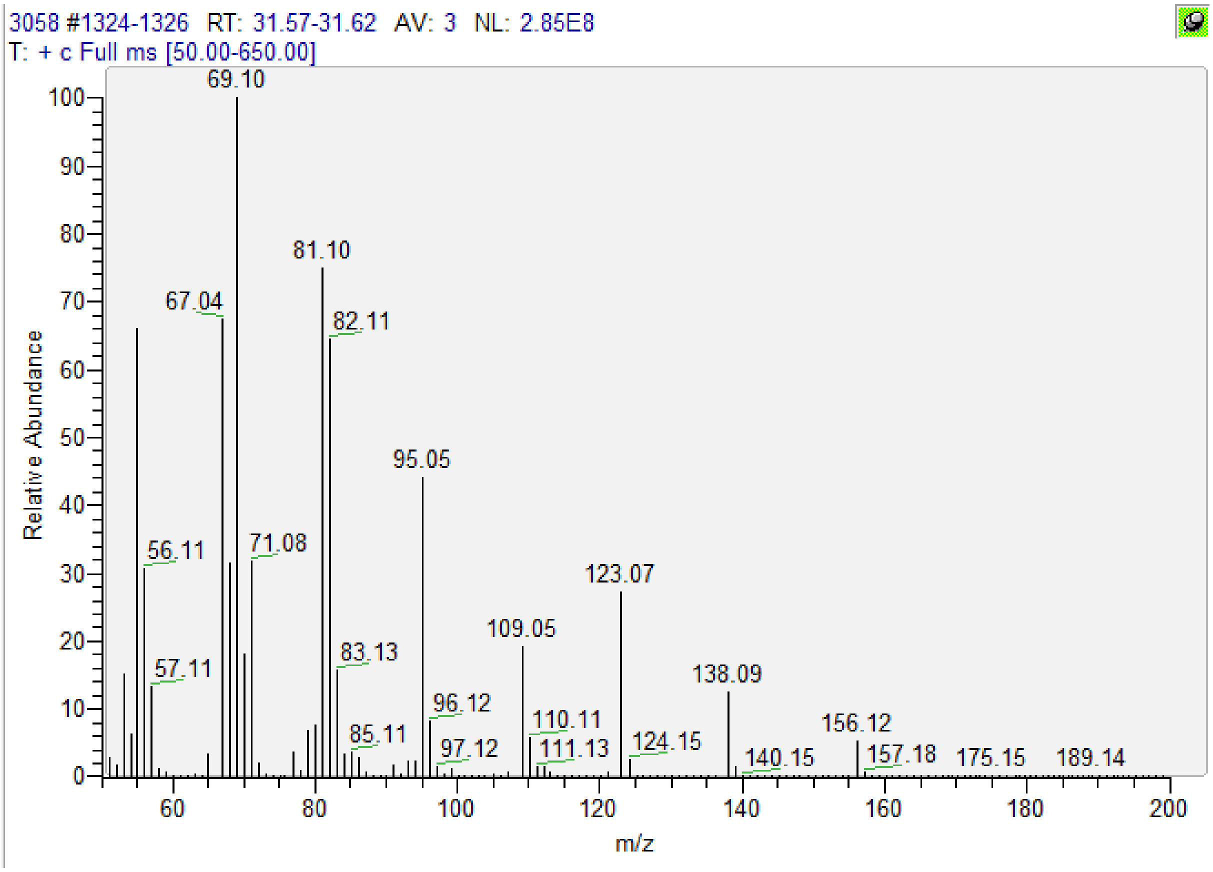
3058 #1324-1326 RT: 31.57-31.62 AV: 3 NL: 2.85E8
T: + c Full ms [50.00-650.00]
Relative Abundance
m/z
69.10
81.10
67.04
82.11
95.05
56.11
71.08
123.07
57.11
83.13
109.05
138.09
96.12
110.11
156.12
85.11
97.12
111.13
124.15
140.15
157.18
175.15
189.14

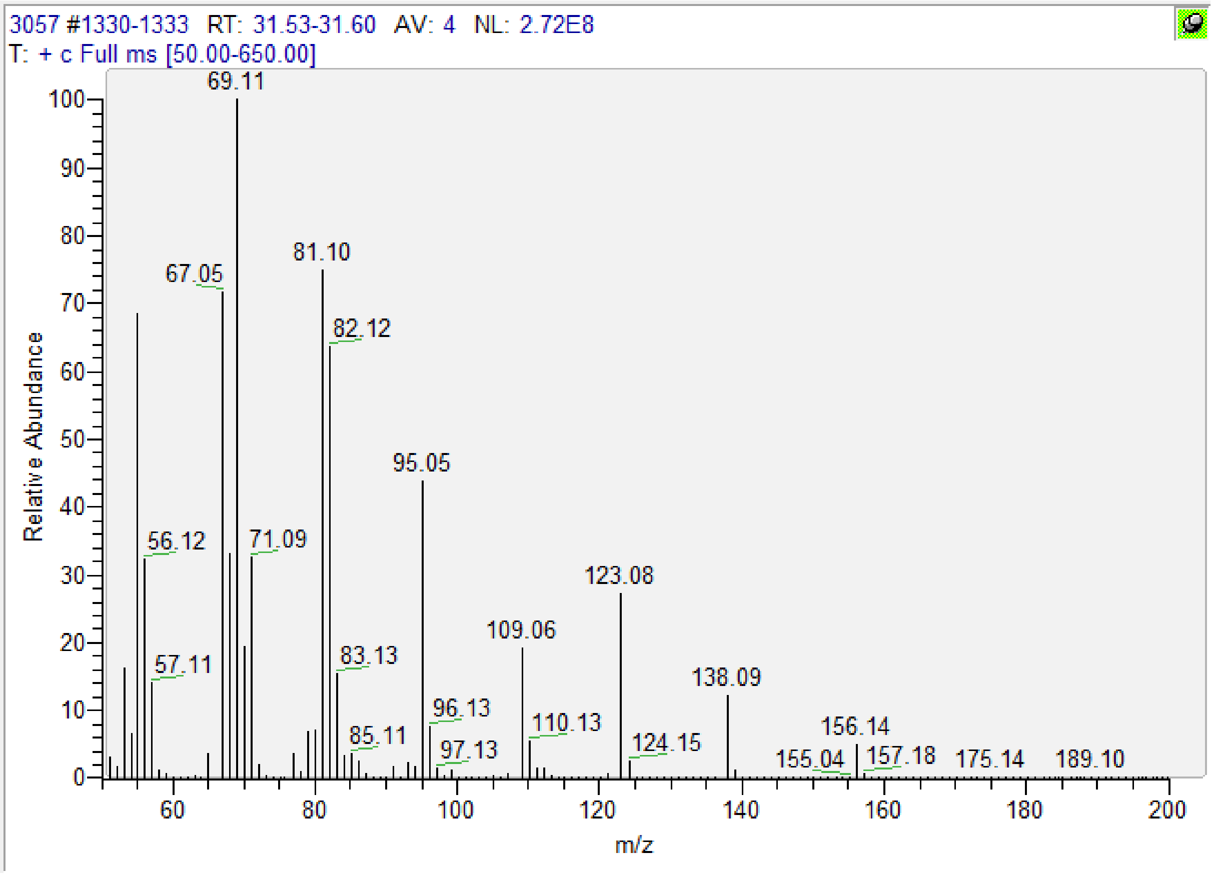
3057 #1330-1333 RT: 31.53-31.60 AV: 4 NL: 2.72E8
T: + c Full ms [50.00-650.00]
Relative Abundance
m/z
69.11
81.10
67.05
82.12
95.05
56.12
71.09
123.08
109.06
83.13
57.11
138.09
96.13
110.13
156.14
85.11
124.15
97.13
157.18
155.04
175.14
189.10

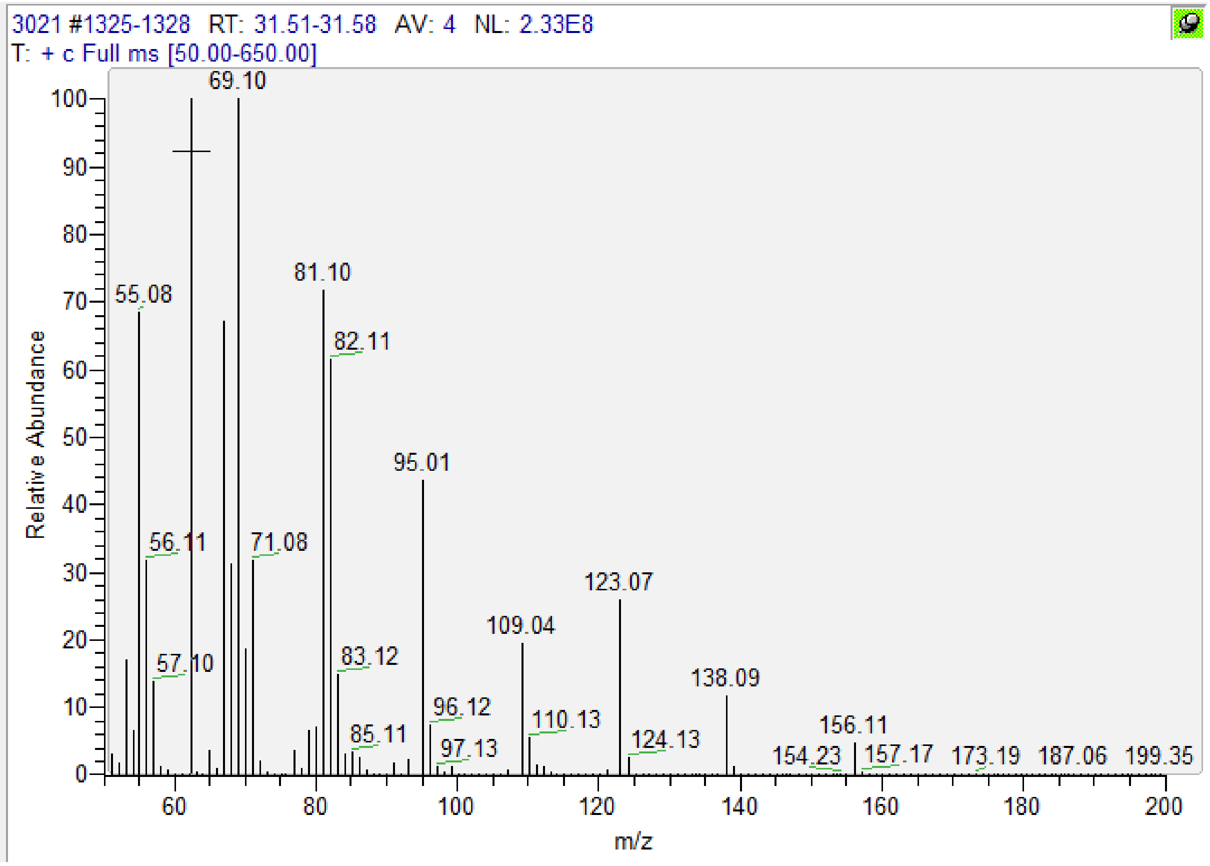

3021 #1325-1328 RT: 31.51-31.58 AV: 4 NL: 2.33E8
T: + c Full ms [50.00-650.00]
Relative Abundance
m/z
69.10
55.08
81.10
82.11
95.01
56.11
71.08
123.07
109.04
57.10
83.12
138.09
96.12
110.13
85.11
156.11
124.13
97.13
154.23
157.17
173.19
187.06
199.35

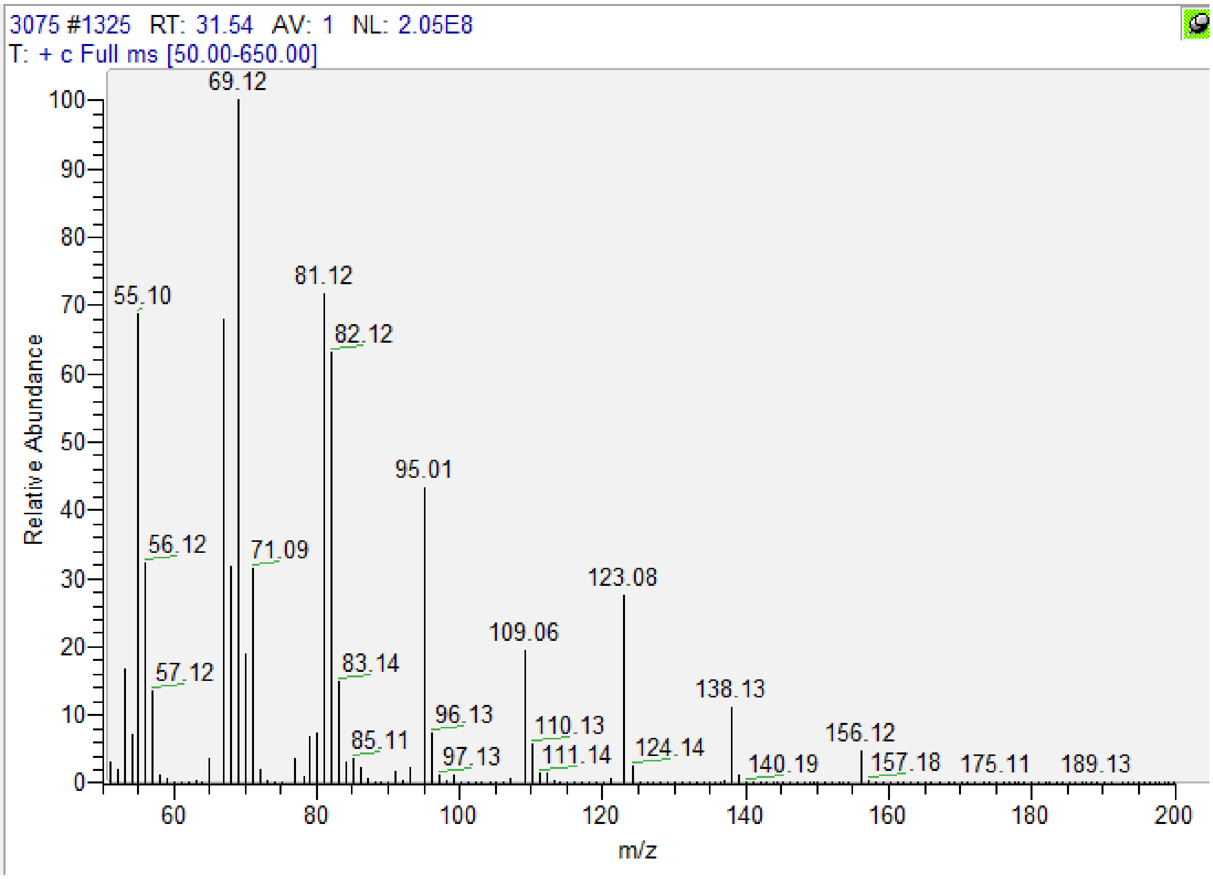

3075 #1325 RT: 31.54 AV: 1 NL: 2.05E8
T: + c Full ms [50.00-650.00]
Relative Abundance
m/z
69.12
55.10
81.12
82.12
95.01
56.12
71.09
123.08
109.06
57.12
83.14
138.13
96.13
110.13
85.11
111.14
124.14
97.13
156.12
140.19
157.18
175.11
189.13

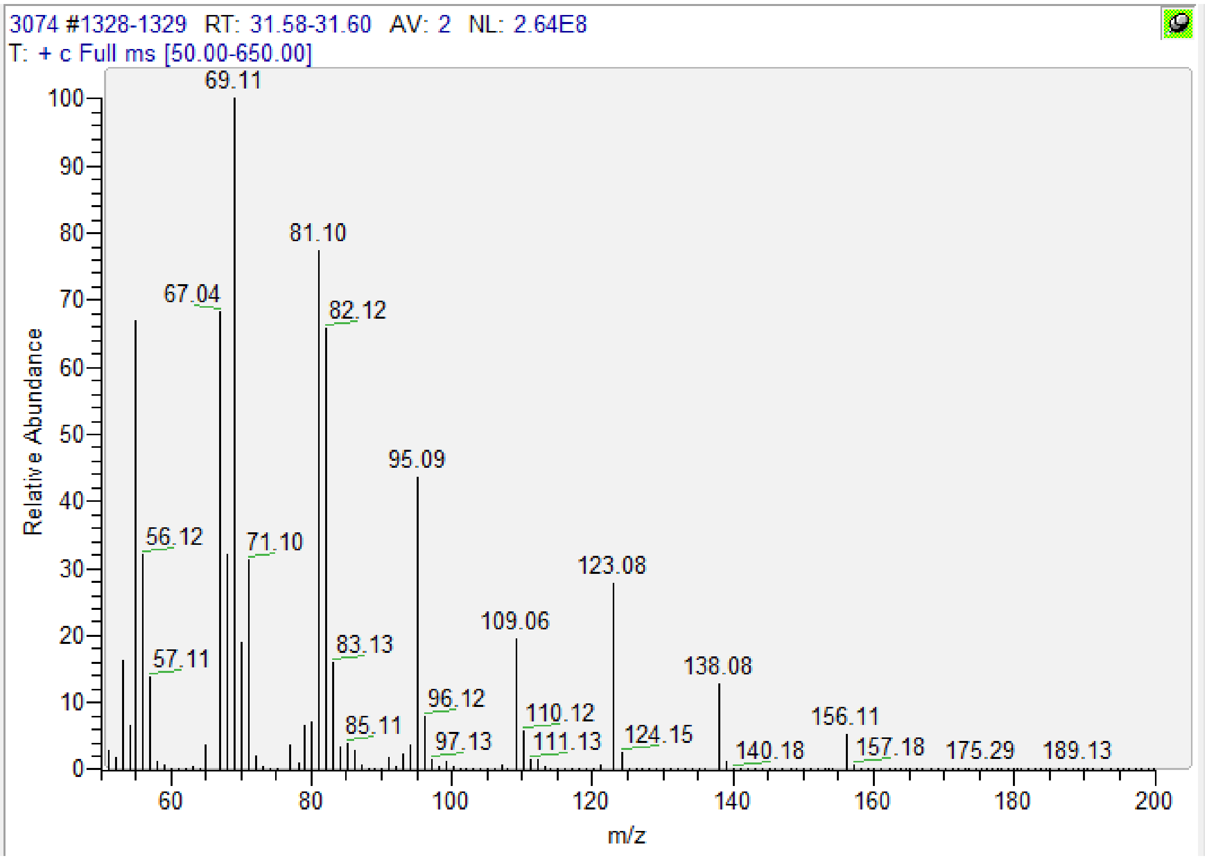

3074 #1328-1329 RT: 31.58-31.60 AV: 2 NL: 2.64E8
T: + c Full ms [50.00-650.00]
Relative Abundance
m/z
69.11
81.10
67.04
82.12
95.09
56.12
71.10
123.08
109.06
83.13
57.11
138.08
96.12
85.11
110.12
156.11
97.13
111.13
124.15
140.18
157.18
175.29
189.13

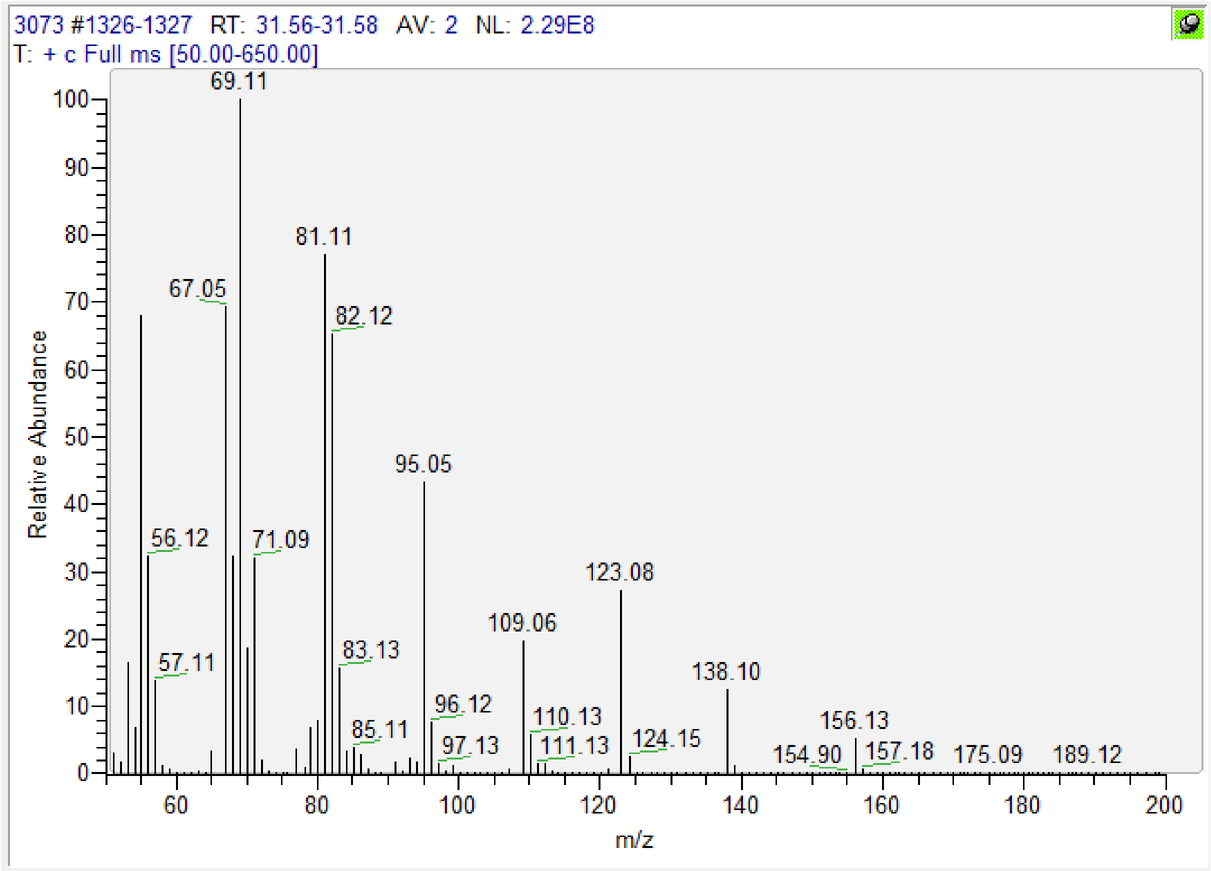

3073 #1326-1327 RT: 31.56-31.58 AV: 2 NL: 2.29E8
T: + c Full ms [50.00-650.00]
Relative Abundance
m/z
69.11
81.11
67.05
82.12
95.05
56.12
71.09
123.08
109.06
83.13
57.11
138.10
96.12
85.11
110.13
156.13
97.13
111.13
124.15
154.90
157.18
175.09
189.12

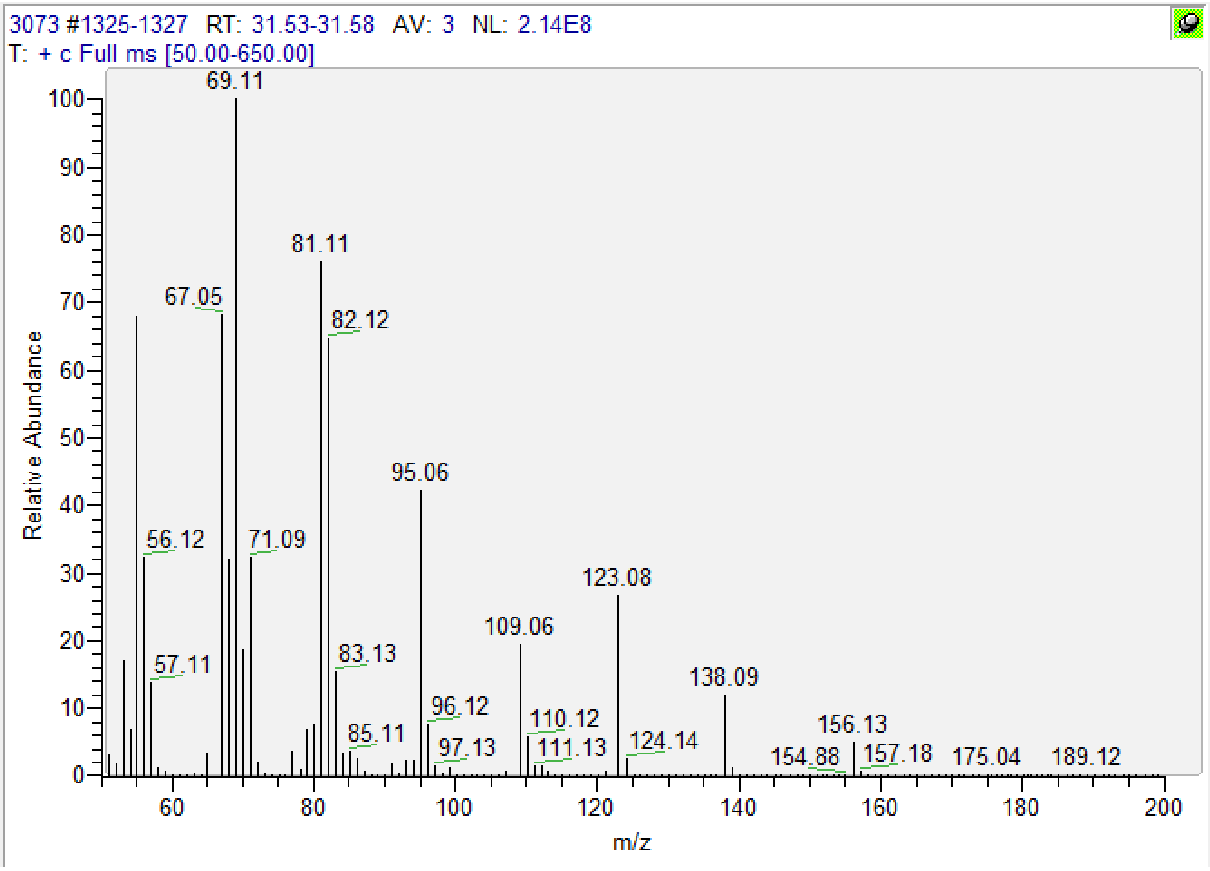
3073 #1325-1327 RT: 31.53-31.58 AV: 3 NL: 2.14E8
T: + c Full ms [50.00-650.00]
Relative Abundance
m/z
69.11
81.11
67.05
82.12
95.06
56.12
71.09
123.08
109.06
83.13
57.11
138.09
96.12
85.11
110.12
156.13
97.13
111.13
124.14
154.88
157.18
175.04
189.12

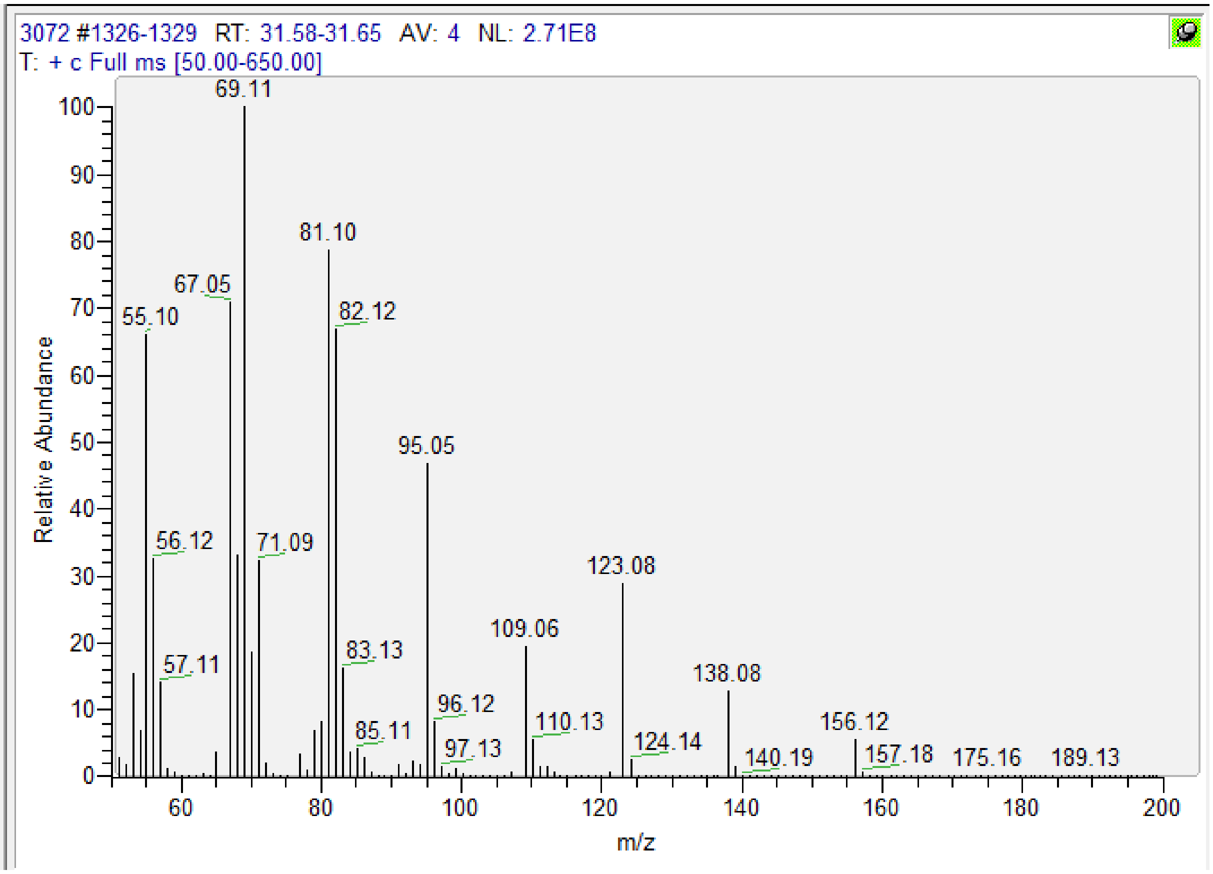
3072 #1326-1329 RT: 31.58-31.65 AV: 4 NL: 2.71E8
T: + c Full ms [50.00-650.00]
Relative Abundance
m/z
69.11
81.10
67.05
55.10
82.12
95.05
56.12
71.09
123.08
109.06
83.13
57.11
138.08
96.12
85.11
110.13
156.12
124.14
97.13
140.19
157.18
175.16
189.13

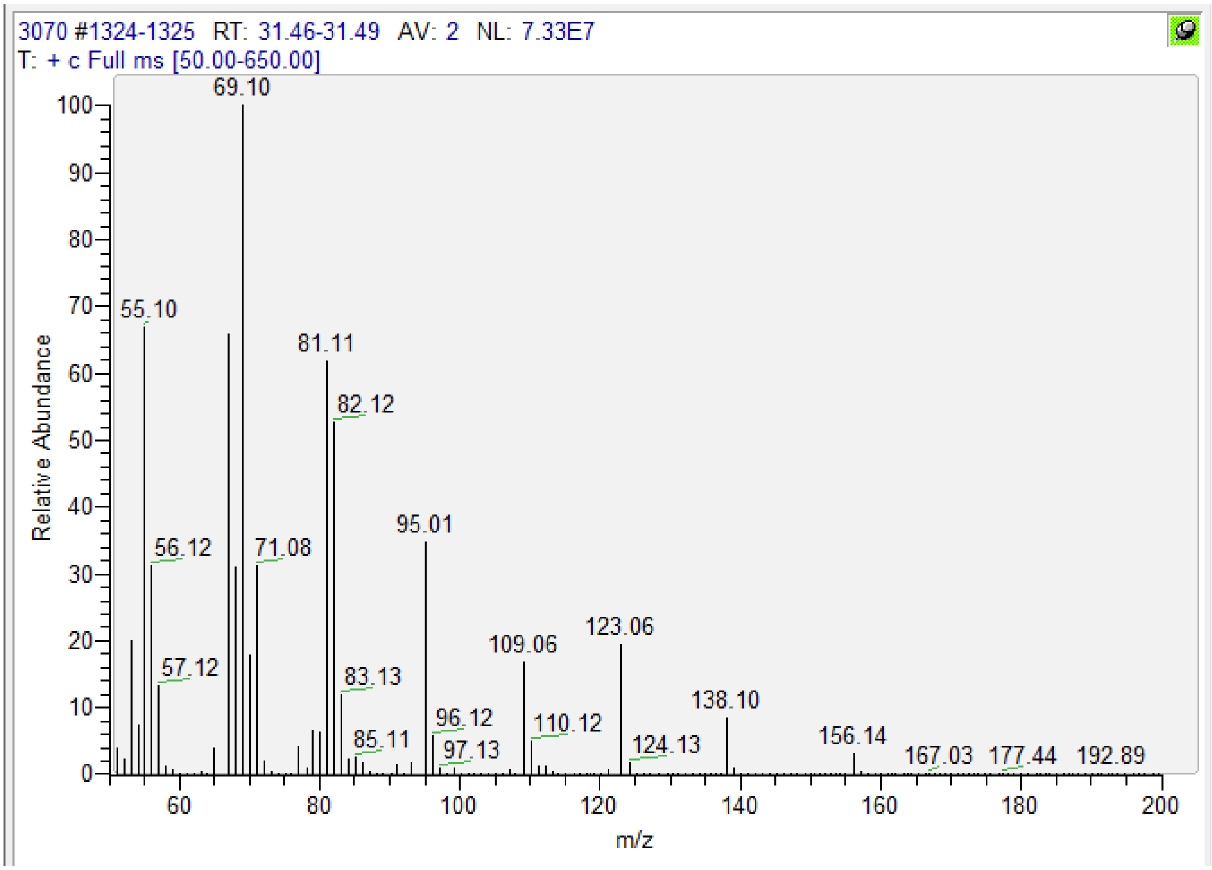
3070 #1324-1325 RT: 31.46-31.49 AV: 2 NL: 7.33E7
T: + c Full ms [50.00-650.00]
Relative Abundance
m/z
69.10
55.10
81.11
82.12
95.01
56.12
71.08
57.12
83.13
109.06
123.06
138.10
96.12
110.12
85.11
97.13
124.13
156.14
167.03
177.44
192.89

Nerol

Reference:

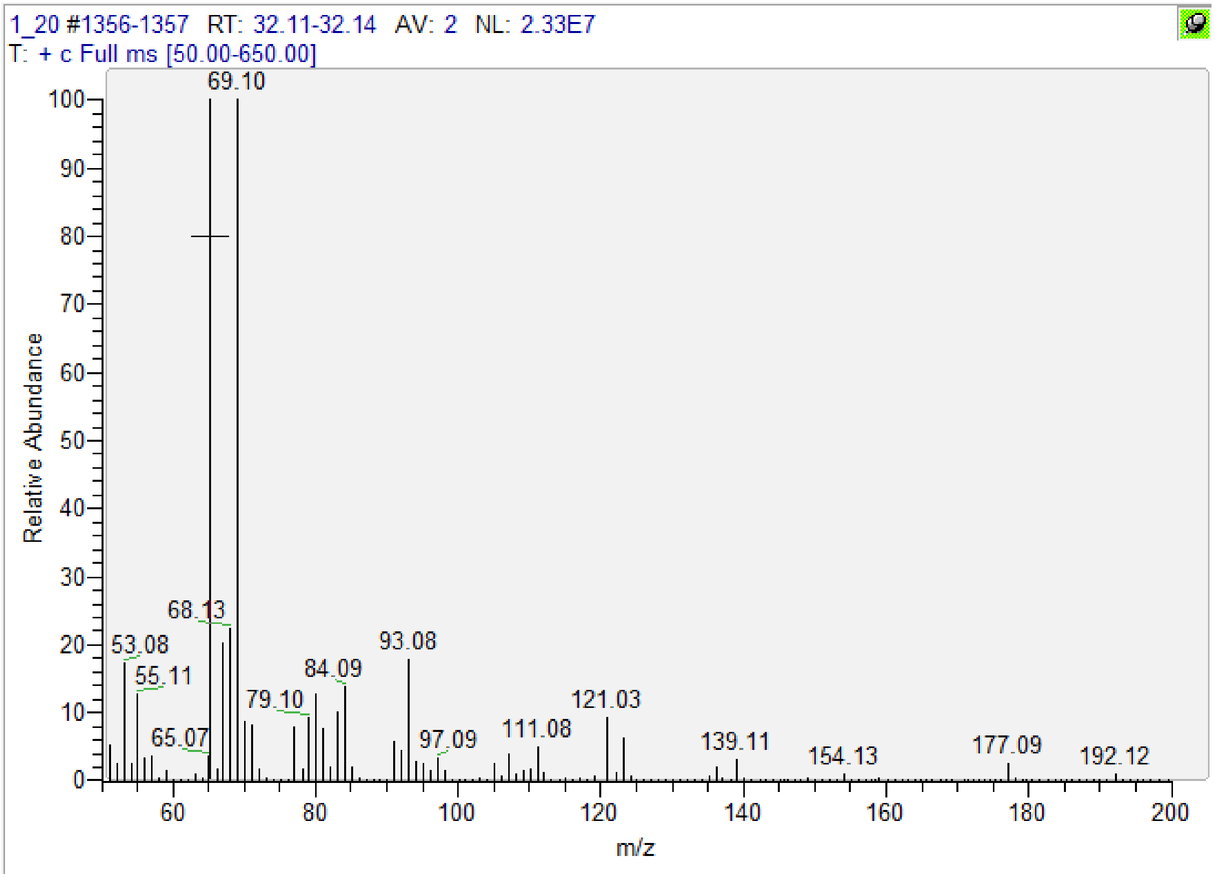

Samples:

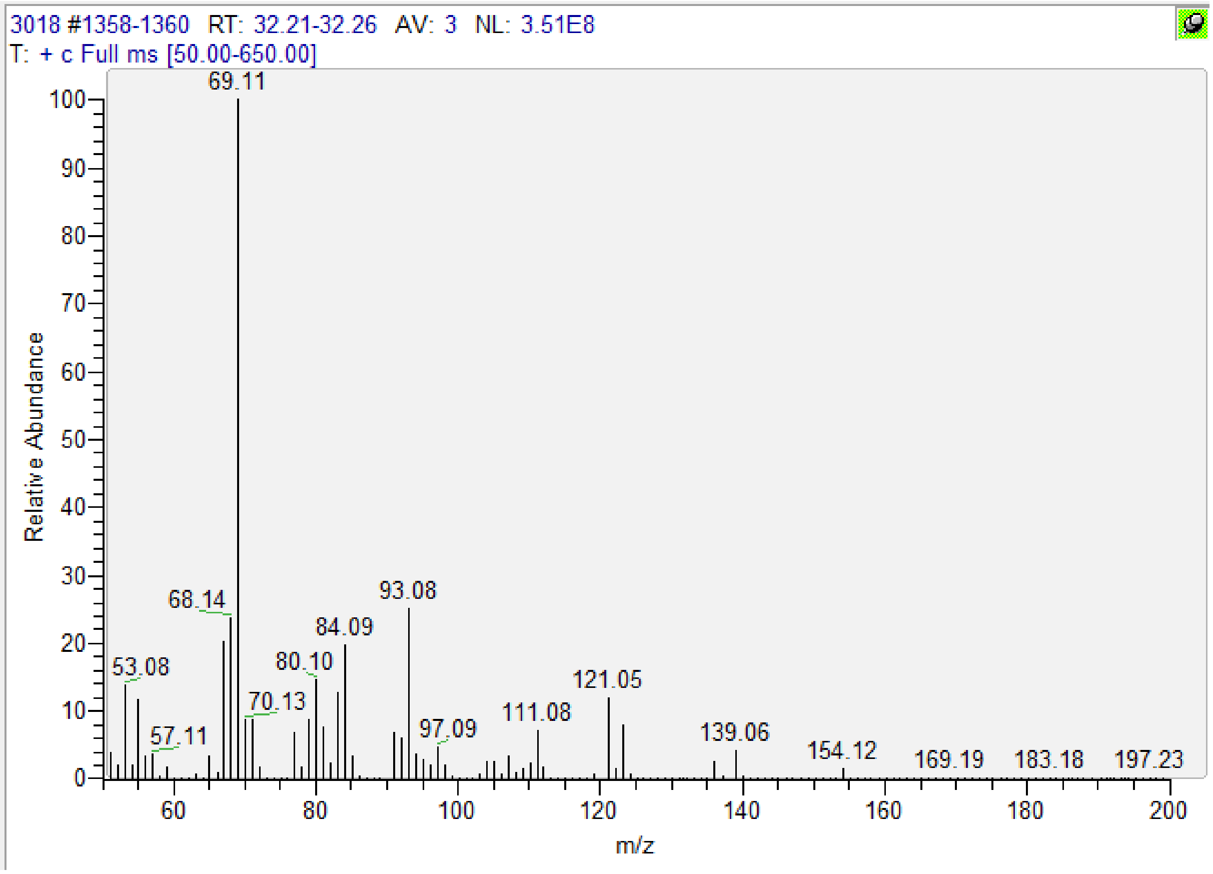

3018 #1358-1360 RT: 32.21-32.26 AV: 3 NL: 3.51E8
T: + c Full ms [50.00-650.00]
Relative Abundance
m/z
69.11
68.14
53.08
57.11
70.13
80.10
84.09
93.08
97.09
111.08
121.05
139.06
154.12
169.19
183.18
197.23

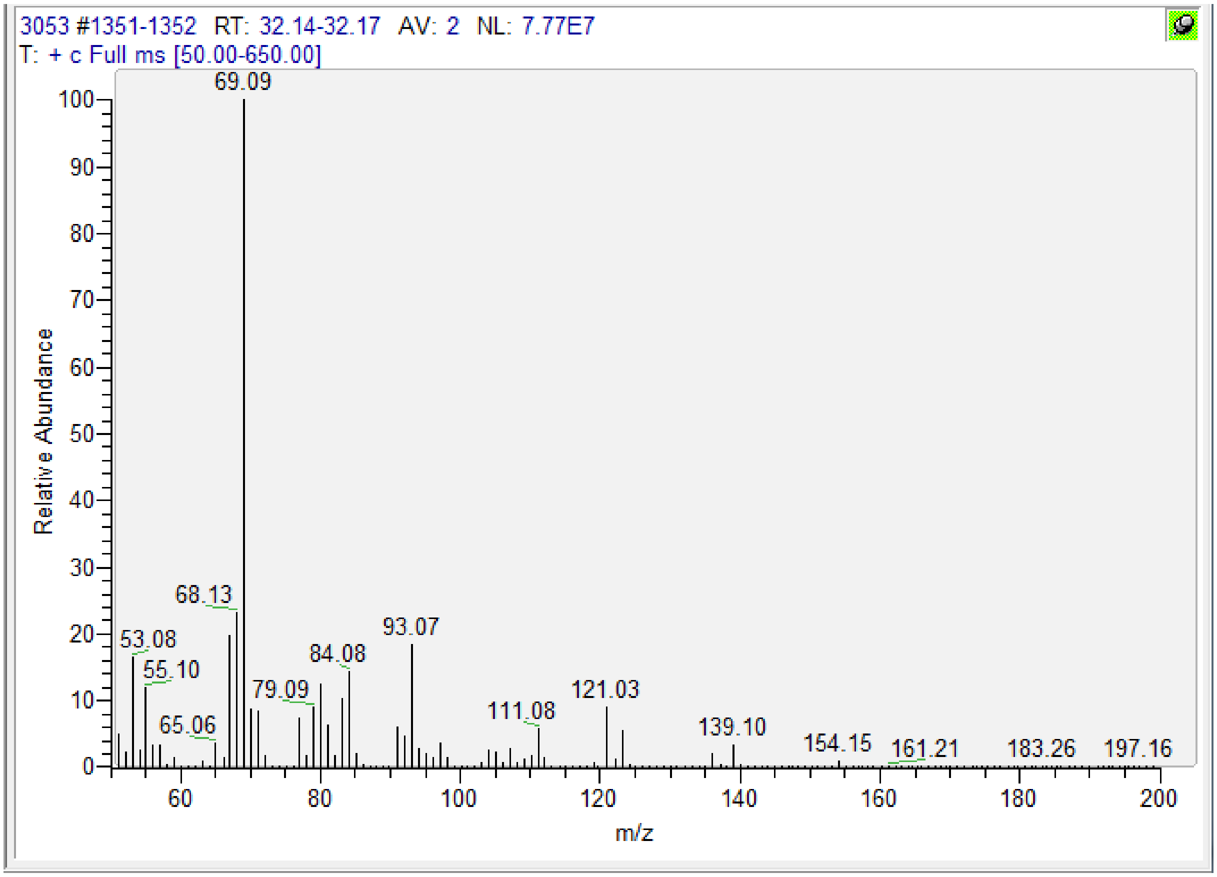
3053 #1351-1352 RT: 32.14-32.17 AV: 2 NL: 7.77E7
T: + c Full ms [50.00-650.00]
Relative Abundance
m/z
69.09
68.13
53.08
55.10
65.06
79.09
84.08
93.07
111.08
121.03
139.10
154.15
161.21
183.26
197.16

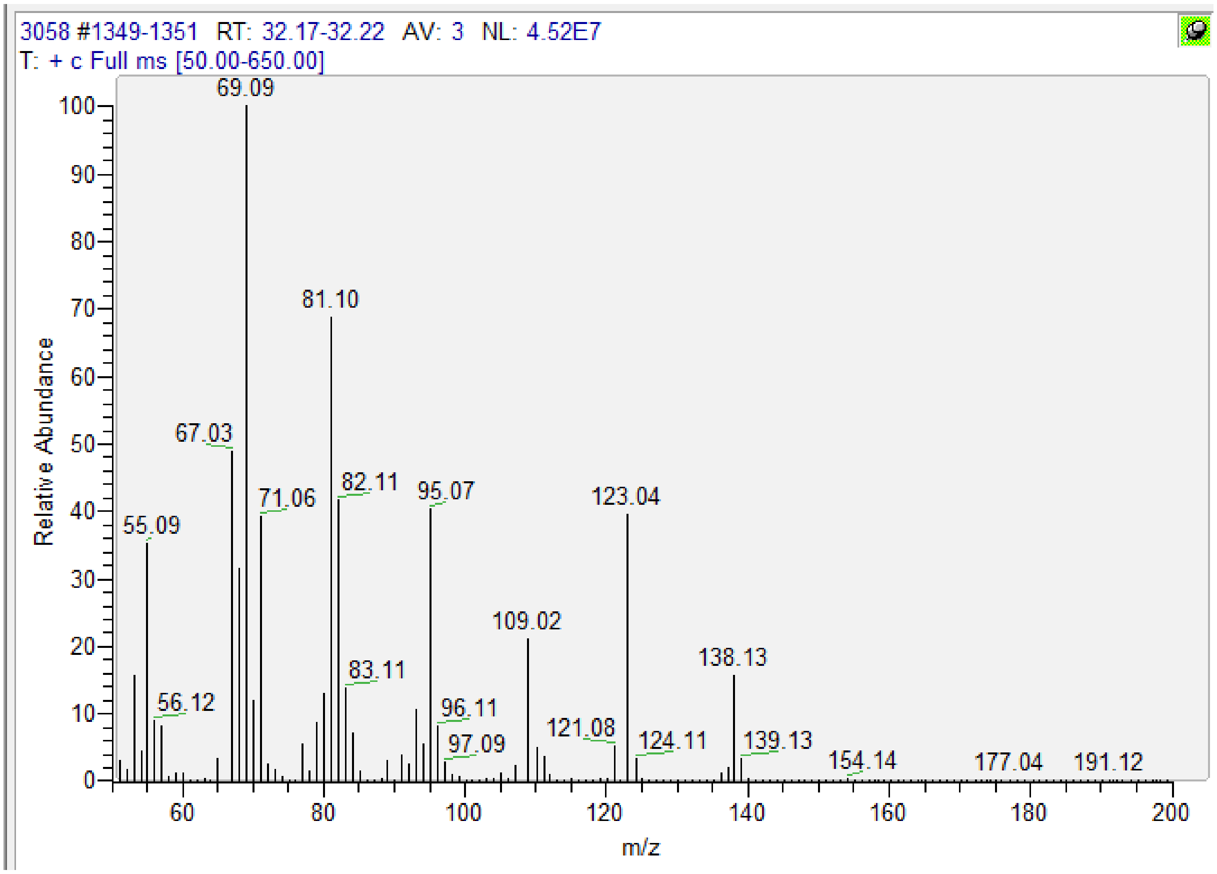
3058 #1349-1351 RT: 32.17-32.22 AV: 3 NL: 4.52E7
T: + c Full ms [50.00-650.00]
Relative Abundance
m/z
69.09
81.10
67.03
82.11
95.07
123.04
71.06
55.09
109.02
138.13
83.11
56.12
96.11
121.08
124.11
139.13
97.09
154.14
177.04
191.12

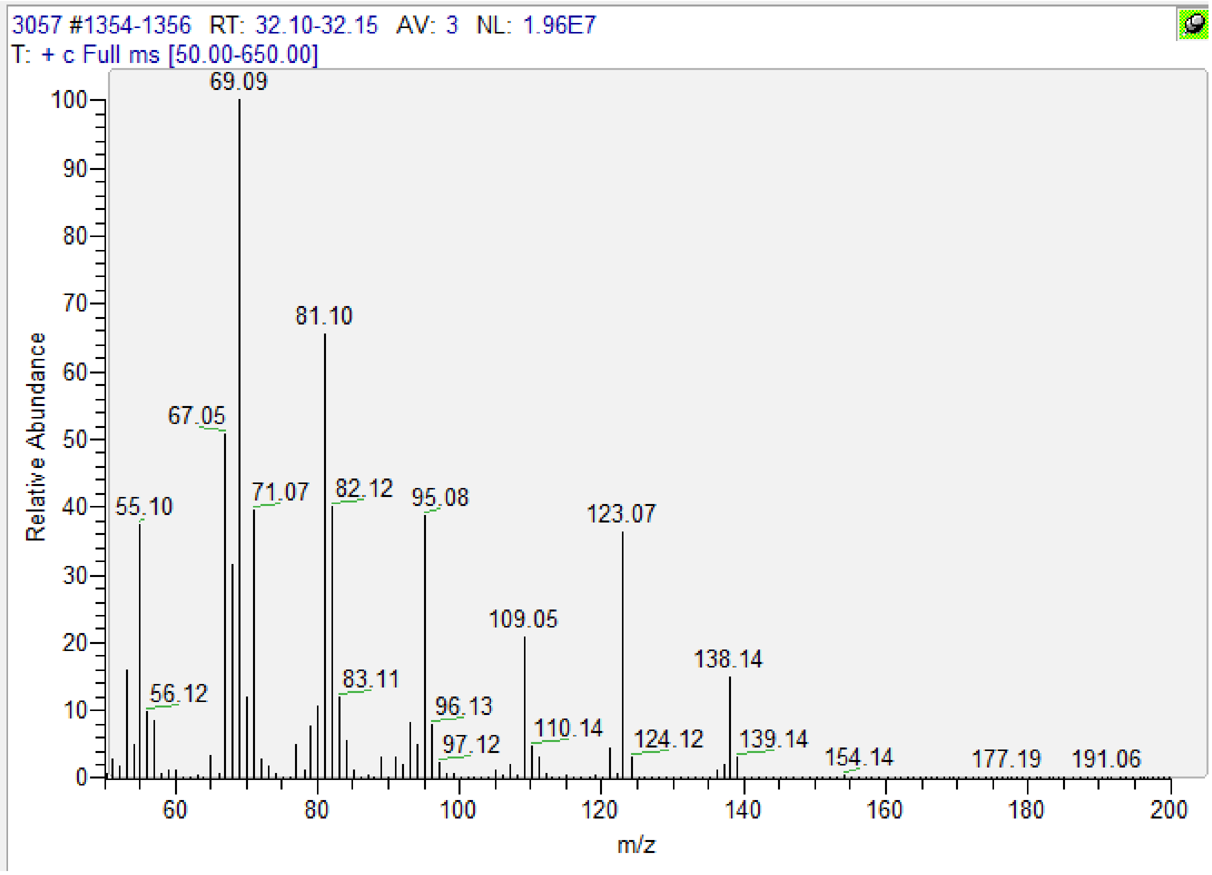

3057 #1354-1356 RT: 32.10-32.15 AV: 3 NL: 1.96E7
T: + c Full ms [50.00-650.00]
Relative Abundance
m/z
69.09
81.10
67.05
55.10
71.07
82.12
95.08
123.07
109.05
138.14
56.12
83.11
96.13
97.12
110.14
124.12
139.14
154.14
177.19
191.06

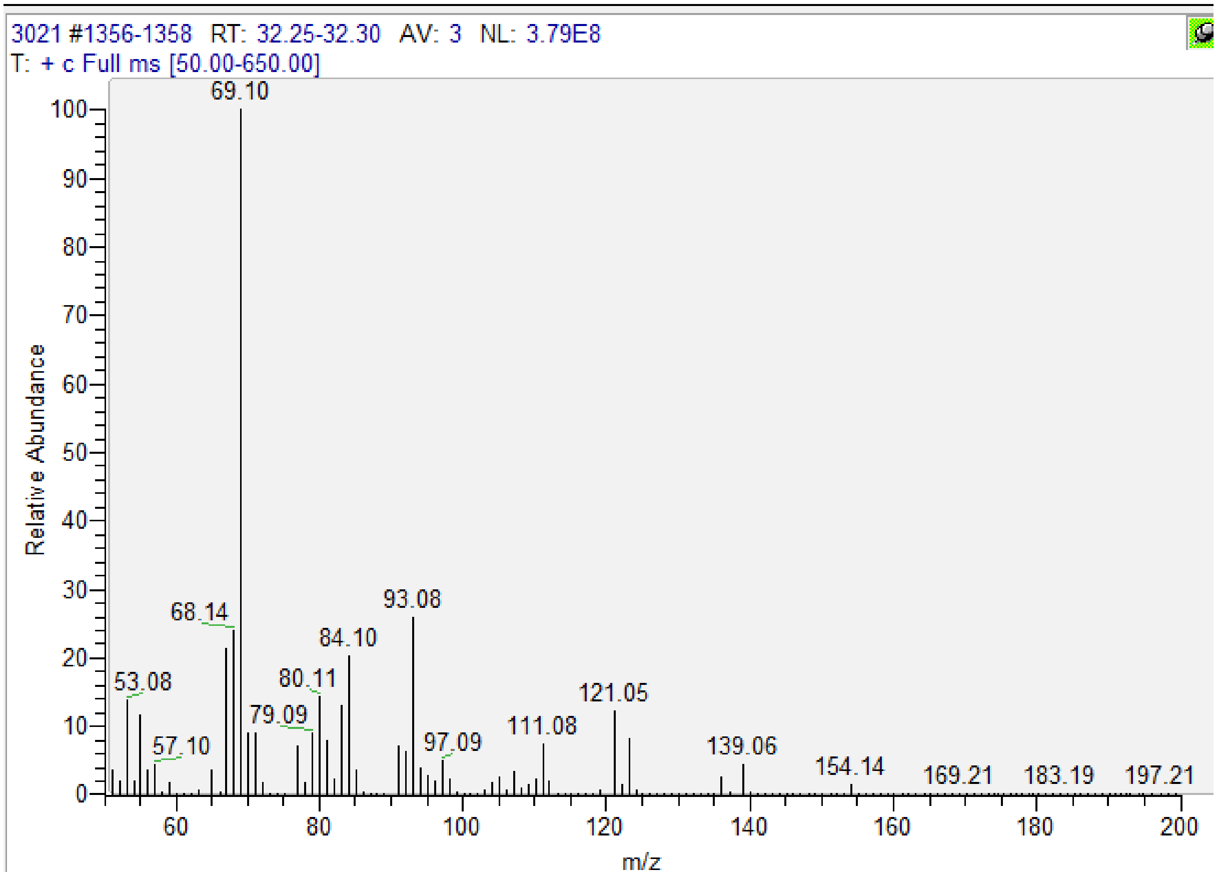
3021 #1356-1358 RT: 32.25-32.30 AV: 3 NL: 3.79E8
T: + c Full ms [50.00-650.00]
Relative Abundance
m/z
69.10
68.14
93.08
84.10
53.08
80.11
79.09
121.05
111.08
57.10
97.09
139.06
154.14
169.21
183.19
197.21
0
10
20
30
40
50
60
70
80
90
100
60
80
100
120
140
160
180
200

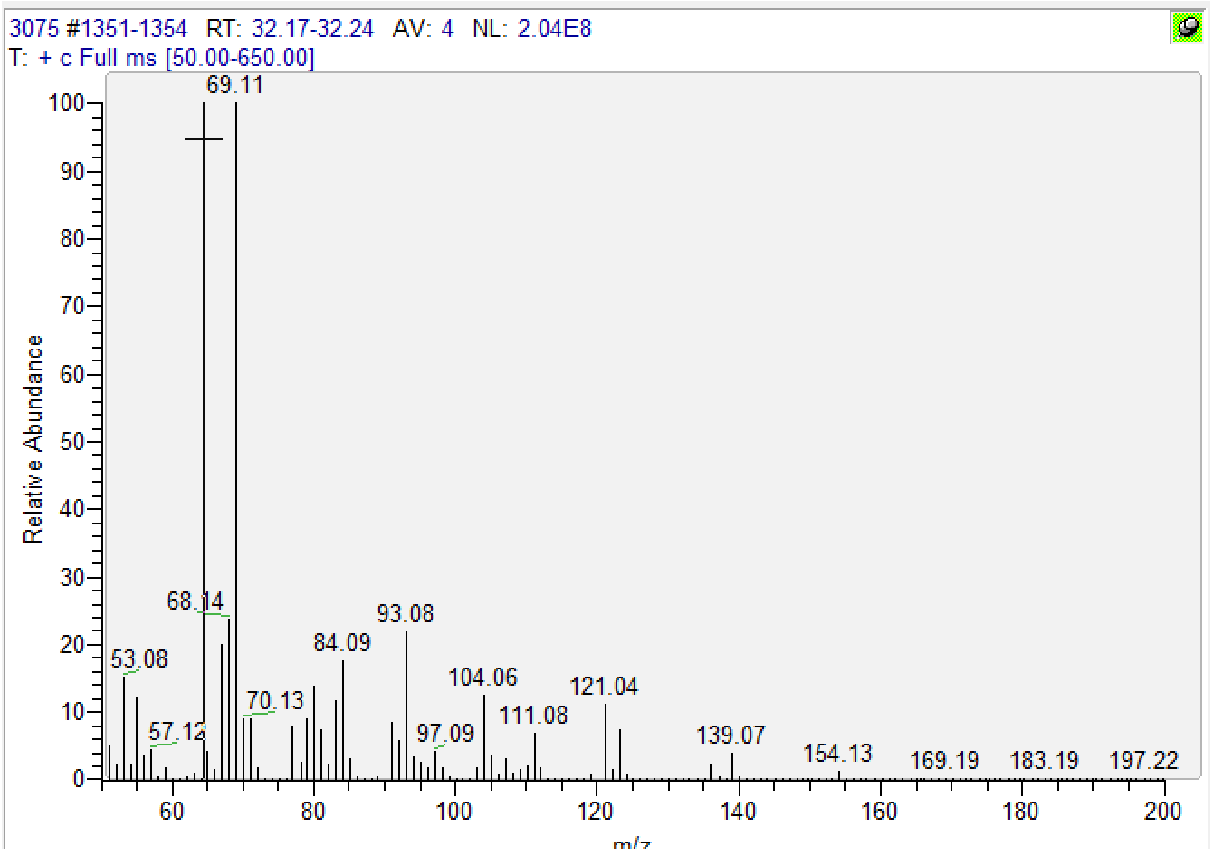
3075 #1351-1354 RT: 32.17-32.24 AV: 4 NL: 2.04E8
T: + c Full ms [50.00-650.00]
Relative Abundance
m/z
69.11
68.14
53.08
57.12
70.13
84.09
93.08
97.09
104.06
111.08
121.04
139.07
154.13
169.19
183.19
197.22

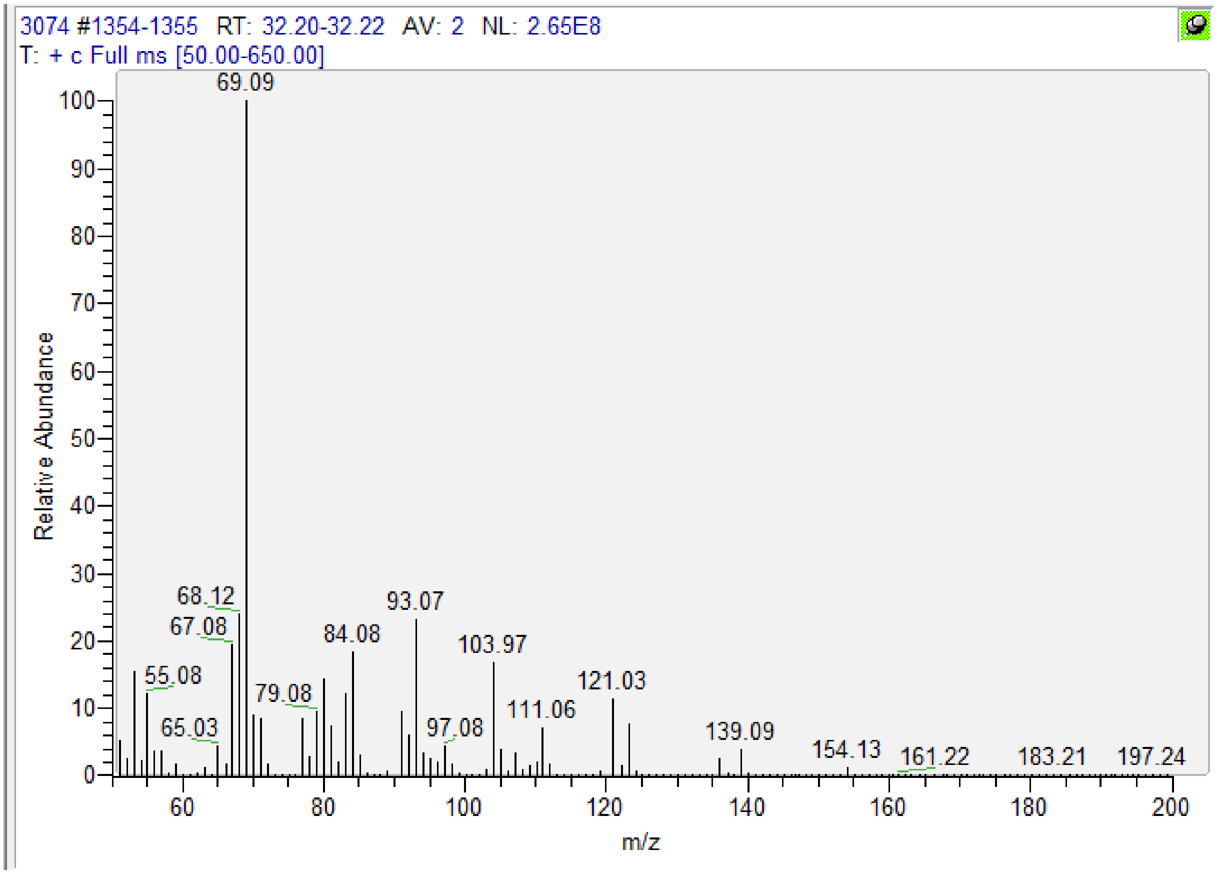

3074 #1354-1355 RT: 32.20-32.22 AV: 2 NL: 2.65E8
T: + c Full ms [50.00-650.00]
Relative Abundance
m/z
69.09
68.12
67.08
55.08
65.03
79.08
84.08
93.07
97.08
103.97
111.06
121.03
139.09
154.13
161.22
183.21
197.24

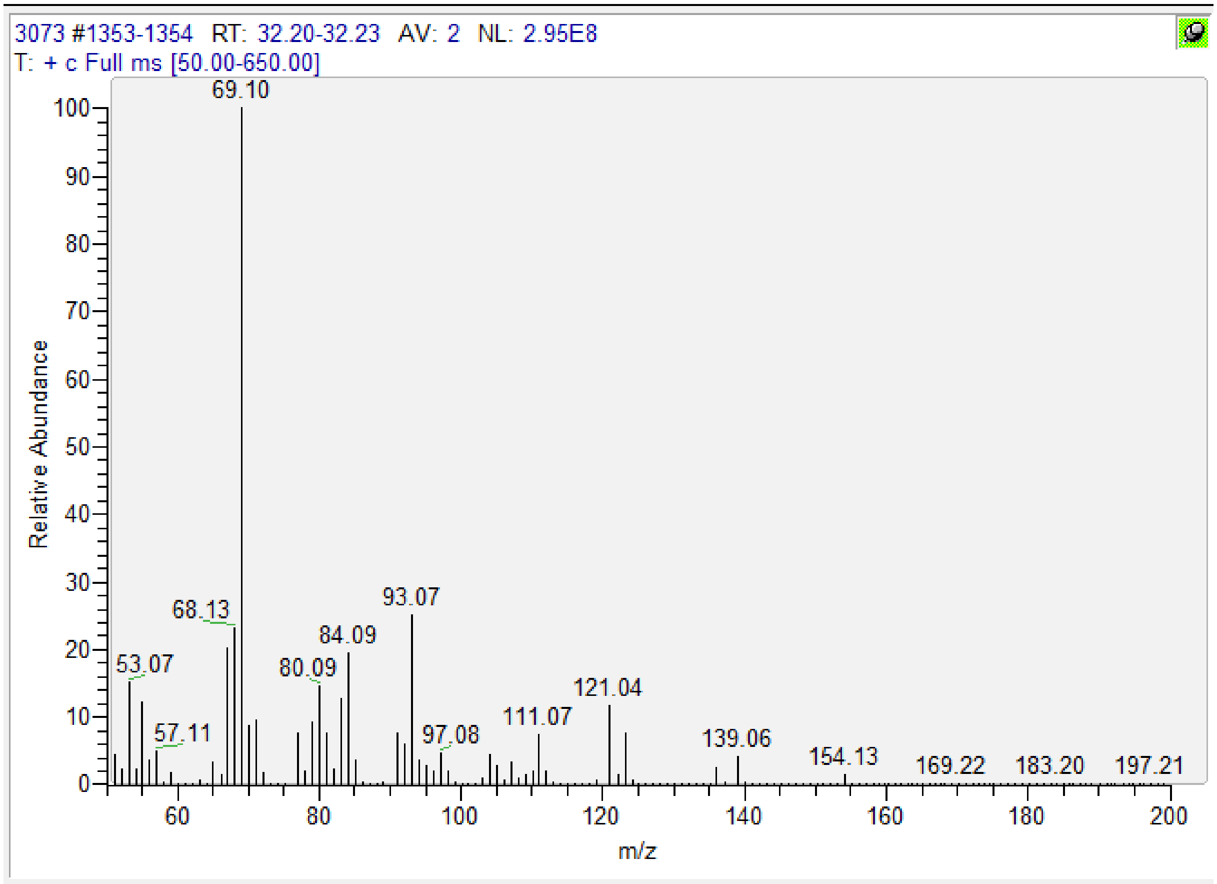

3073 #1353-1354 RT: 32.20-32.23 AV: 2 NL: 2.95E8
T: + c Full ms [50.00-650.00]
Relative Abundance
m/z
69.10
68.13
53.07
57.11
80.09
84.09
93.07
97.08
111.07
121.04
139.06
154.13
169.22
183.20
197.21

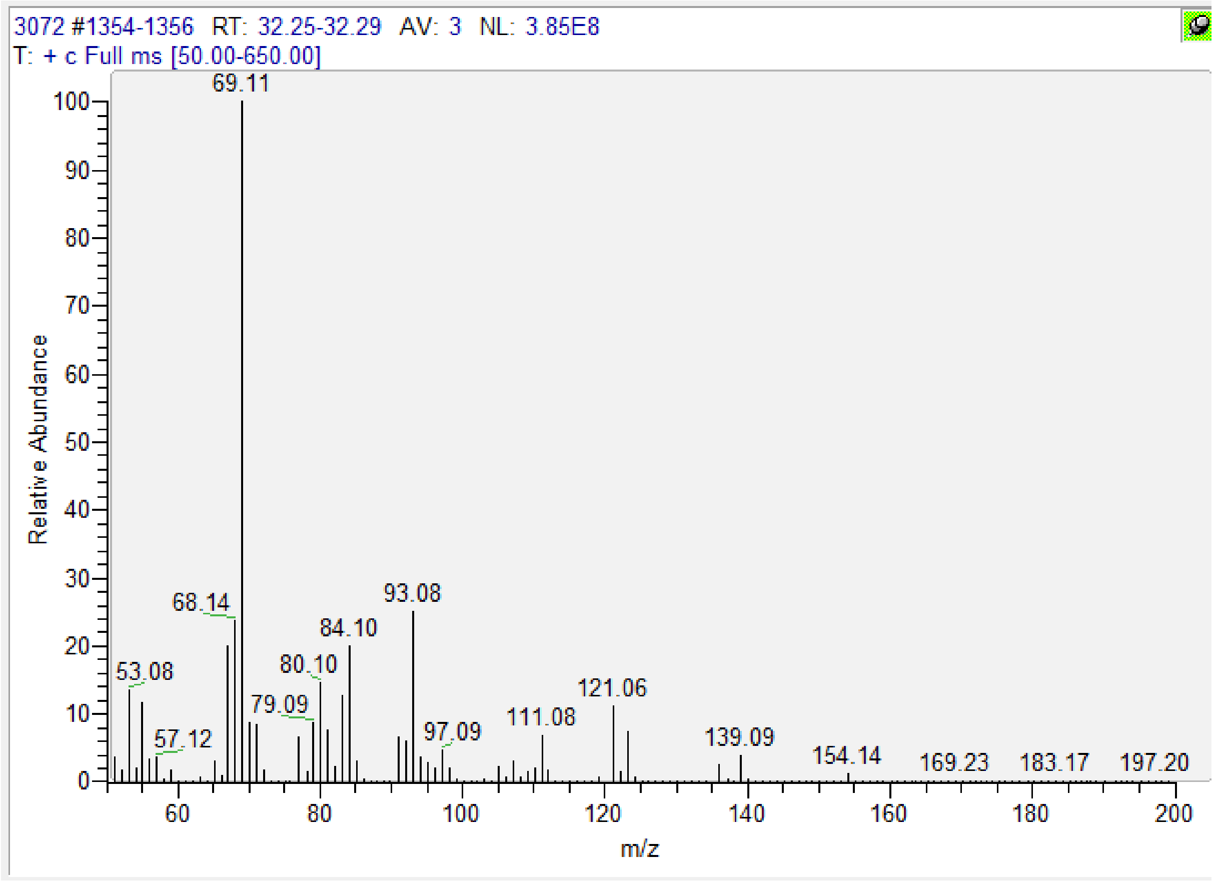

3072 #1354-1356 RT: 32.25-32.29 AV: 3 NL: 3.85E8
T: + c Full ms [50.00-650.00]
Relative Abundance
m/z
69.11
68.14
53.08
57.12
79.09
80.10
84.10
93.08
97.09
111.08
121.06
139.09
154.14
169.23
183.17
197.20

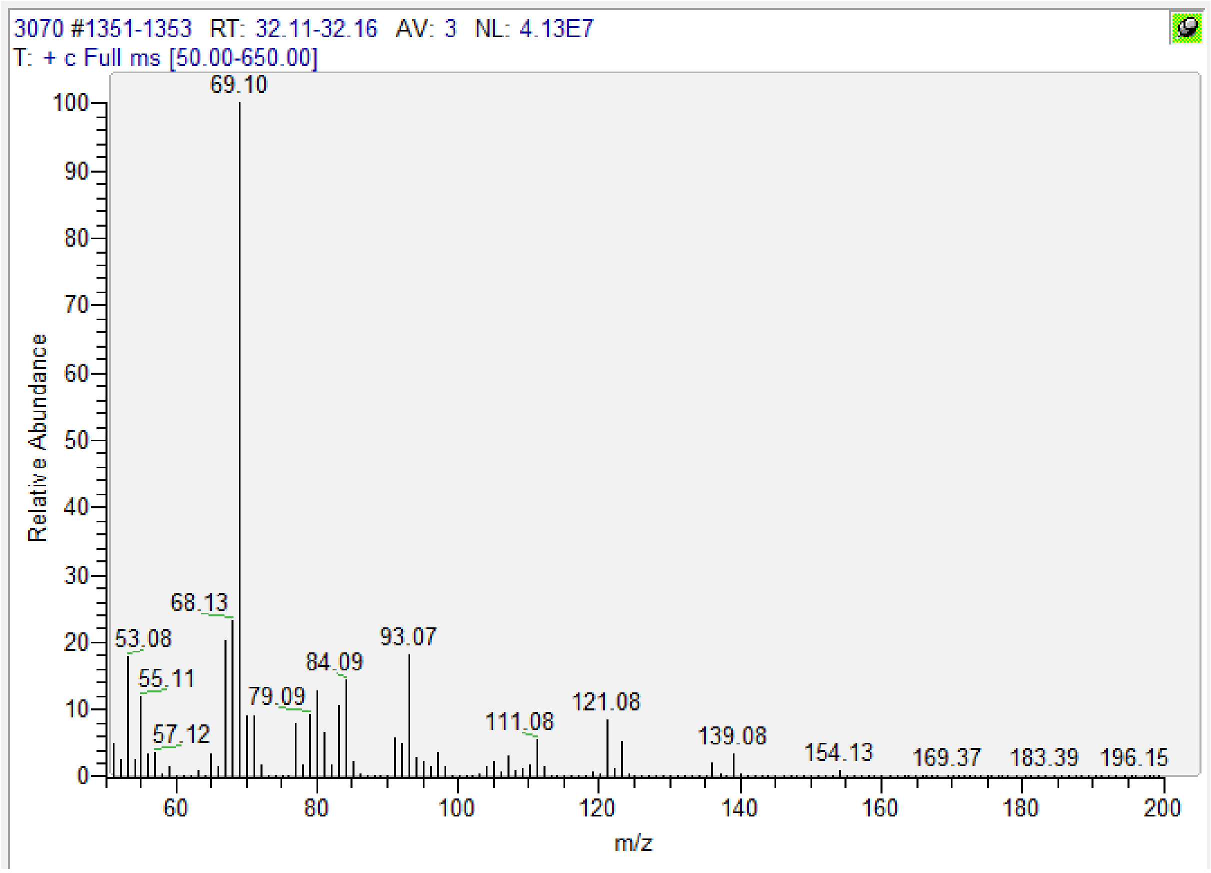
3070 #1351-1353 RT: 32.11-32.16 AV: 3 NL: 4.13E7
T: + c Full ms [50.00-650.00]
69.10
68.13
53.08
55.11
57.12
79.09
84.09
93.07
111.08
121.08
139.08
154.13
169.37
183.39
196.15
Relative Abundance
m/z

Phenylethanol

Reference

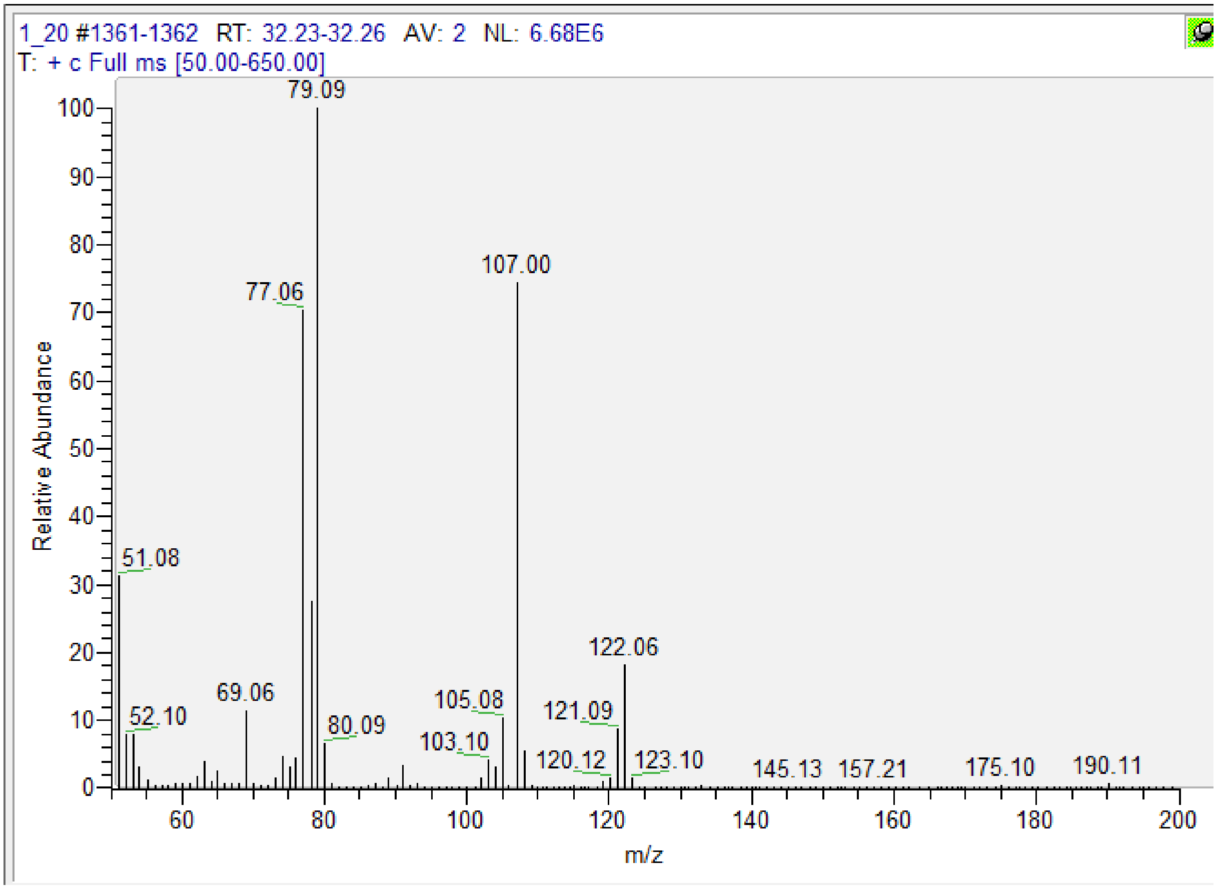

Samples

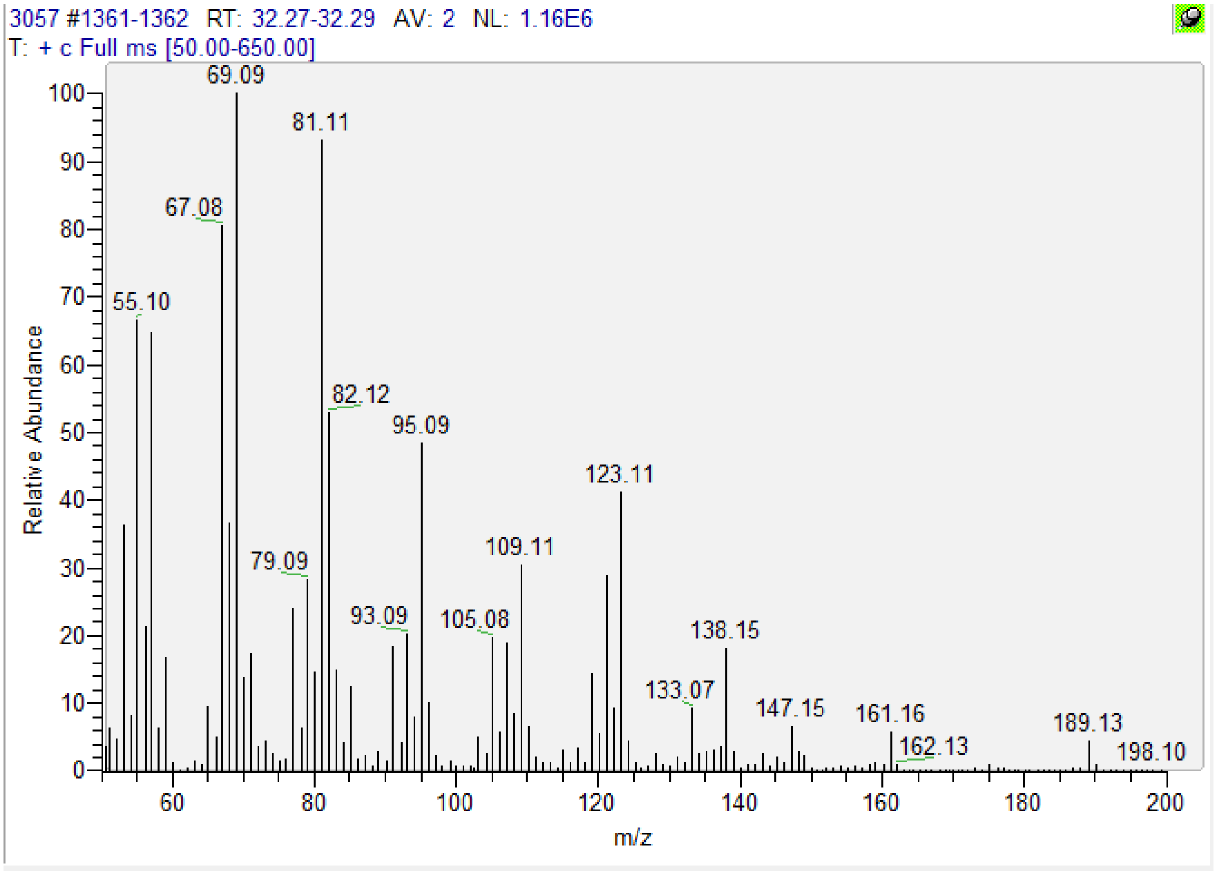

3057 #1361-1362 RT: 32.27-32.29 AV: 2 NL: 1.16E6
T: + c Full ms [50.00-650.00]
69.09
81.11
67.08
55.10
82.12
95.09
123.11
109.11
79.09
93.09
105.08
138.15
133.07
147.15
161.16
162.13
189.13
198.10
Relative Abundance
m/z

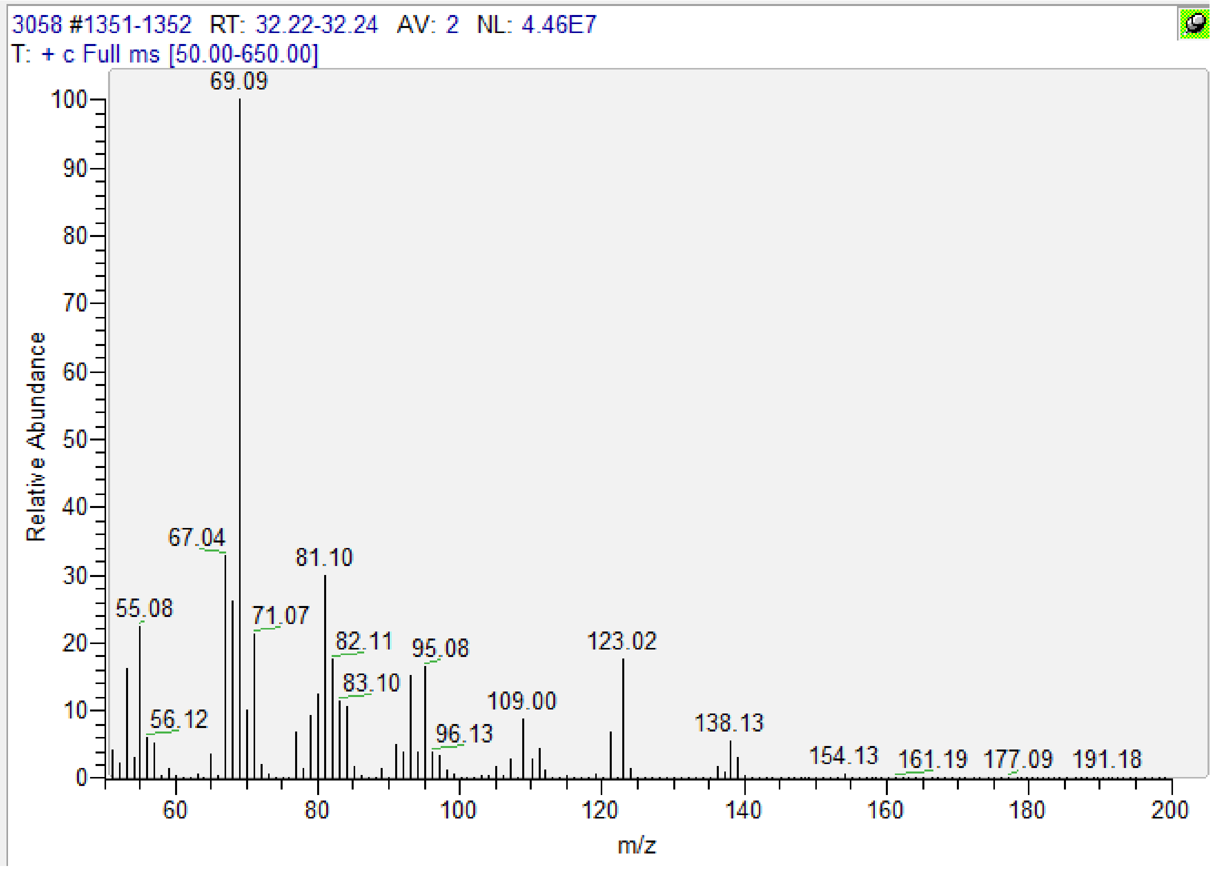

3058 #1351-1352 RT: 32.22-32.24 AV: 2 NL: 4.46E7
T: + c Full ms [50.00-650.00]
Relative Abundance
m/z
69.09
67.04
81.10
55.08
71.07
82.11
95.08
123.02
83.10
109.00
56.12
96.13
138.13
154.13
161.19
177.09
191.18

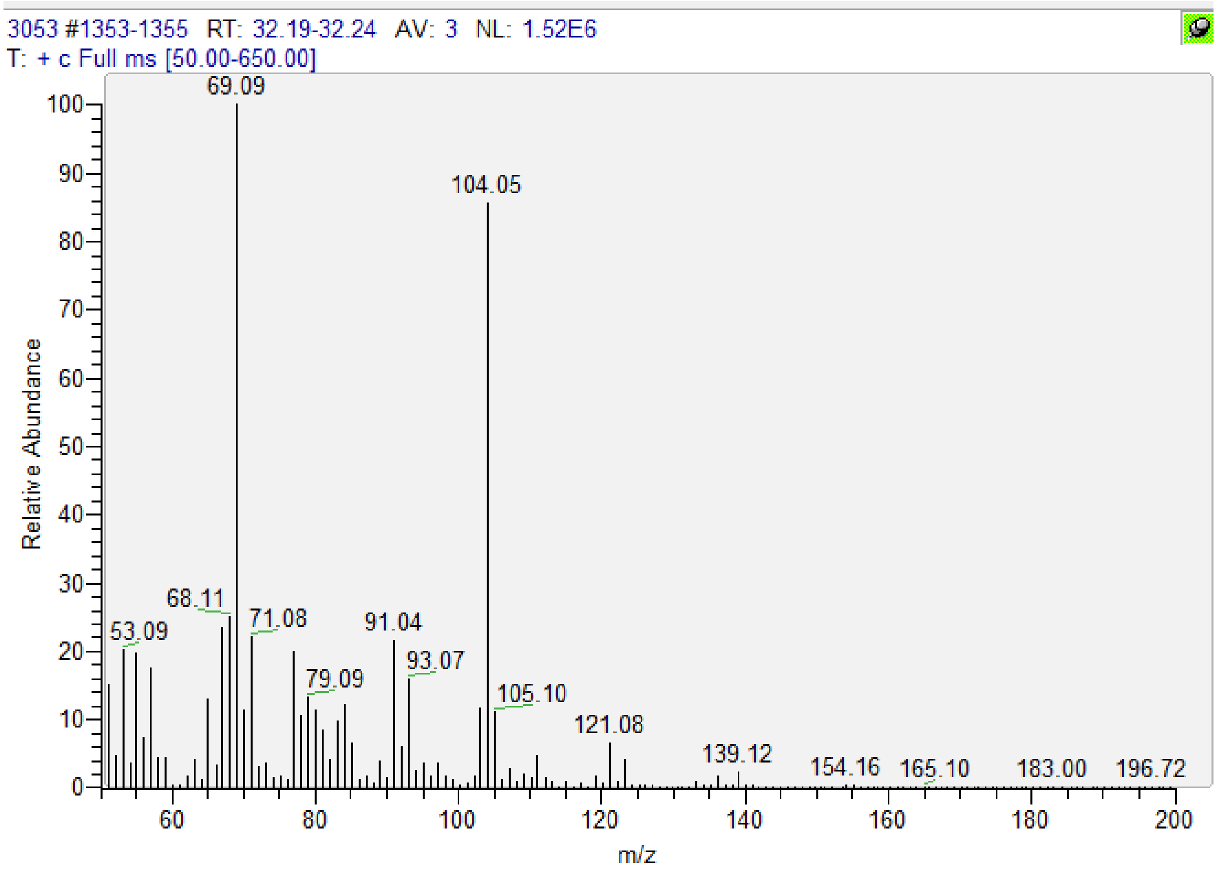

3053 #1353-1355 RT: 32.19-32.24 AV: 3 NL: 1.52E6
T: + c Full ms [50.00-650.00]
Relative Abundance
m/z
69.09
104.05
68.11
71.08
53.09
91.04
93.07
79.09
105.10
121.08
139.12
154.16
165.10
183.00
196.72

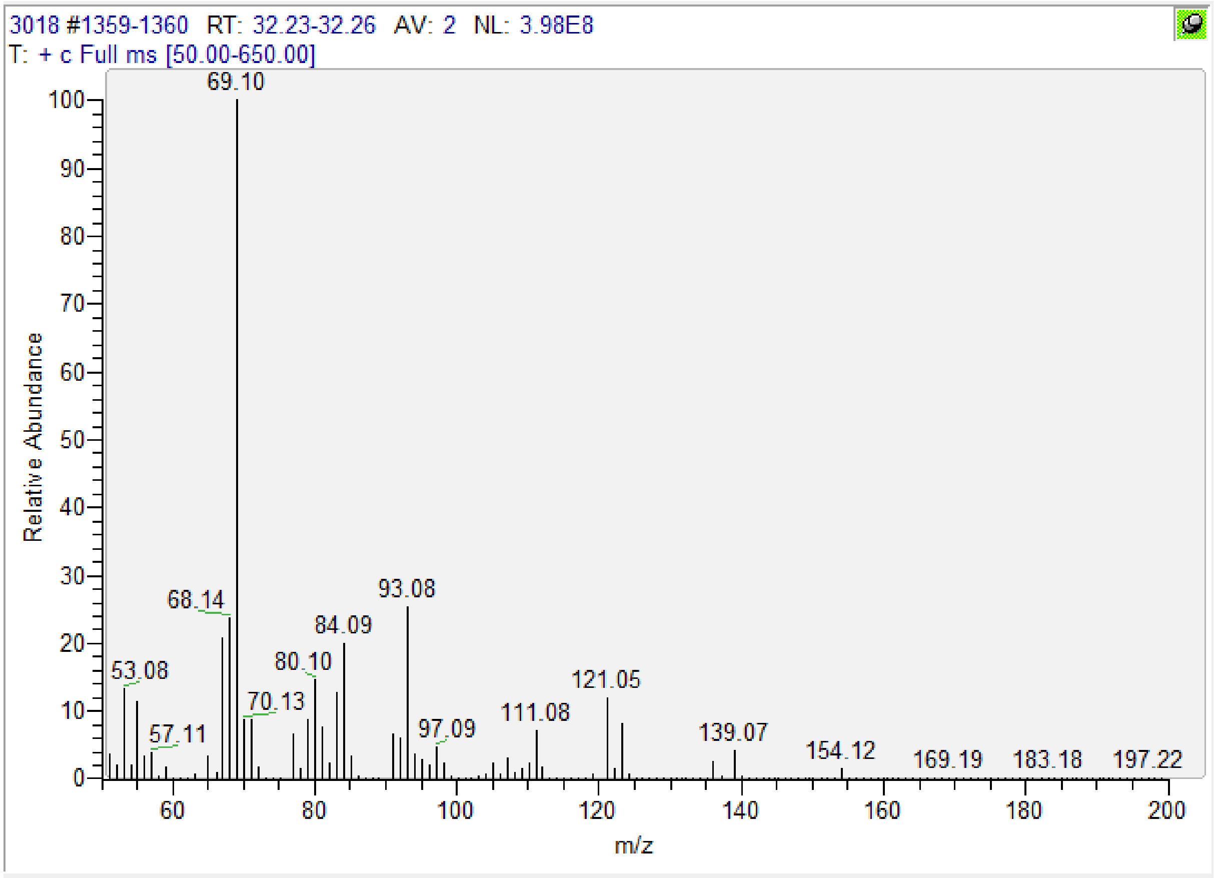
3018 #1359-1360 RT: 32.23-32.26 AV: 2 NL: 3.98E8
T: + c Full ms [50.00-650.00]
Relative Abundance
m/z
69.10
68.14
53.08
57.11
70.13
80.10
84.09
93.08
97.09
111.08
121.05
139.07
154.12
169.19
183.18
197.22

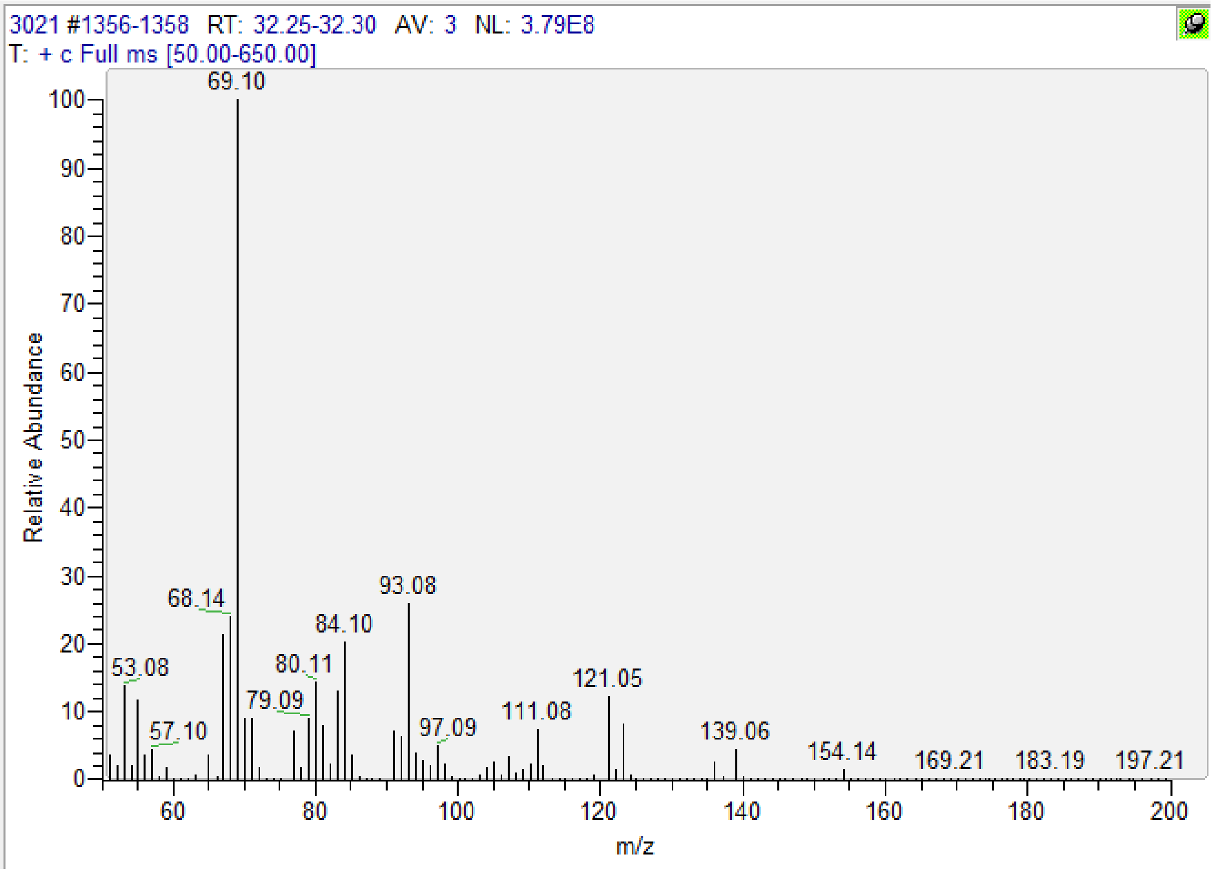
3021 #1356-1358 RT: 32.25-32.30 AV: 3 NL: 3.79E8
T: + c Full ms [50.00-650.00]
Relative Abundance
m/z
69.10
68.14
93.08
84.10
53.08
80.11
79.09
57.10
97.09
111.08
121.05
139.06
154.14
169.21
183.19
197.21

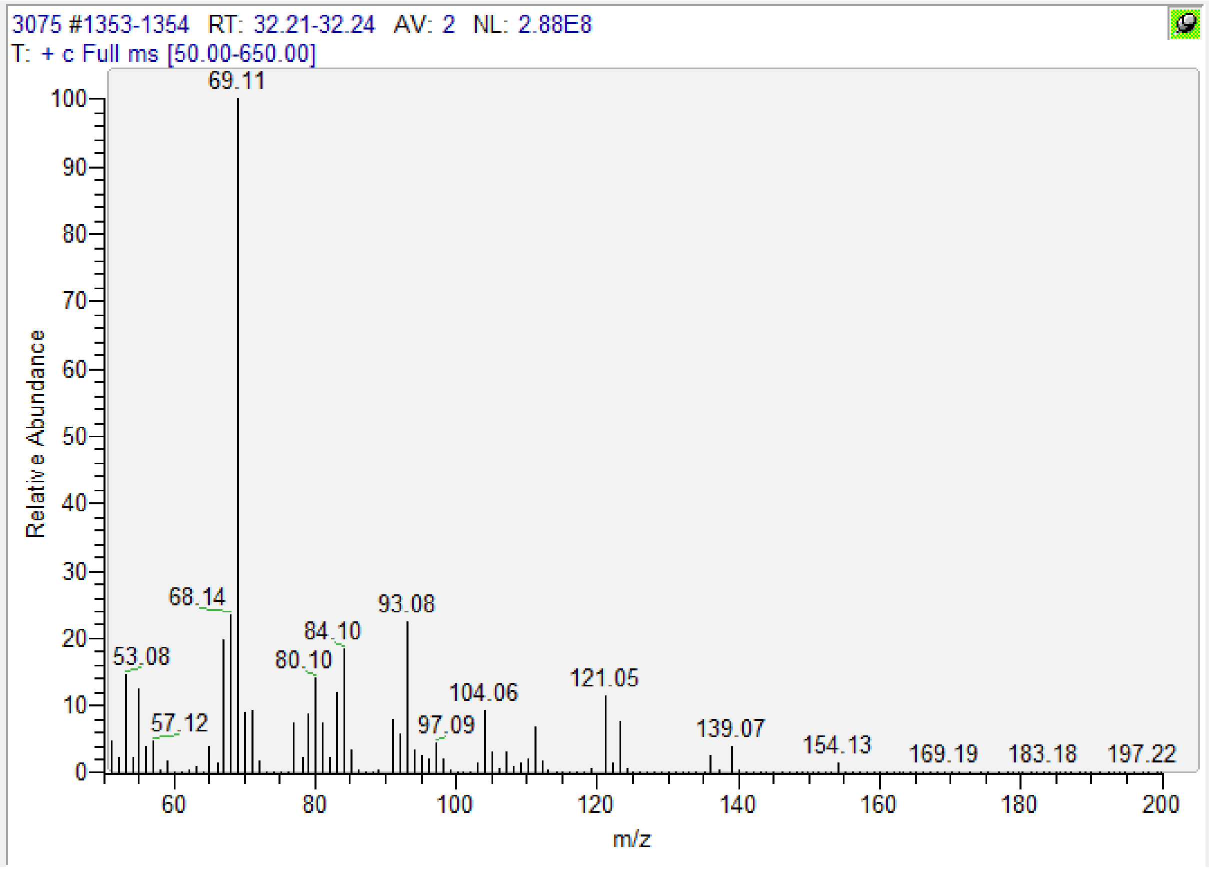
3075 #1353-1354 RT: 32.21-32.24 AV: 2 NL: 2.88E8
T: + c Full ms [50.00-650.00]
Relative Abundance
m/z
69.11
68.14
53.08
57.12
80.10
84.10
93.08
97.09
104.06
121.05
139.07
154.13
169.19
183.18
197.22

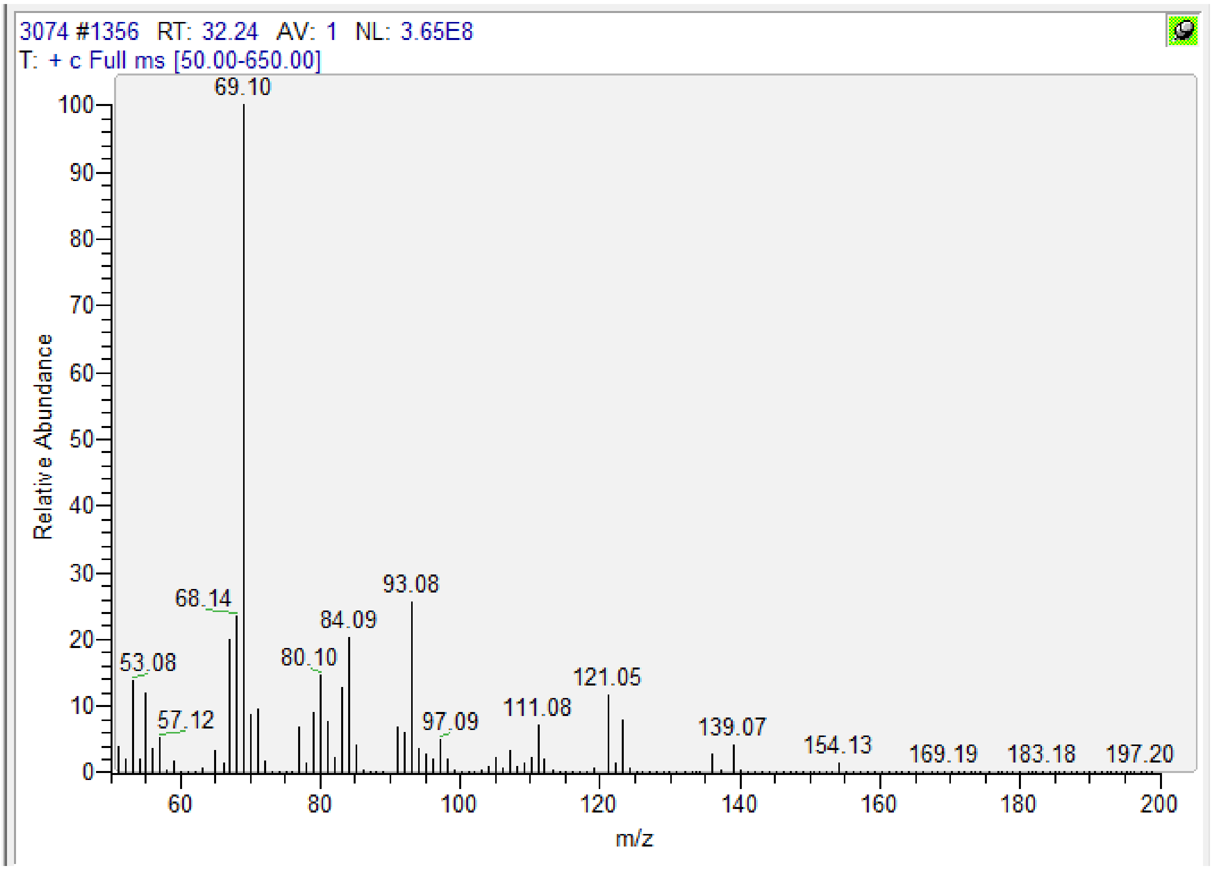

3074 #1356 RT: 32.24 AV: 1 NL: 3.65E8
T: + c Full ms [50.00-650.00]
Relative Abundance
m/z
69.10
68.14
53.08
57.12
80.10
84.09
93.08
97.09
111.08
121.05
139.07
154.13
169.19
183.18
197.20

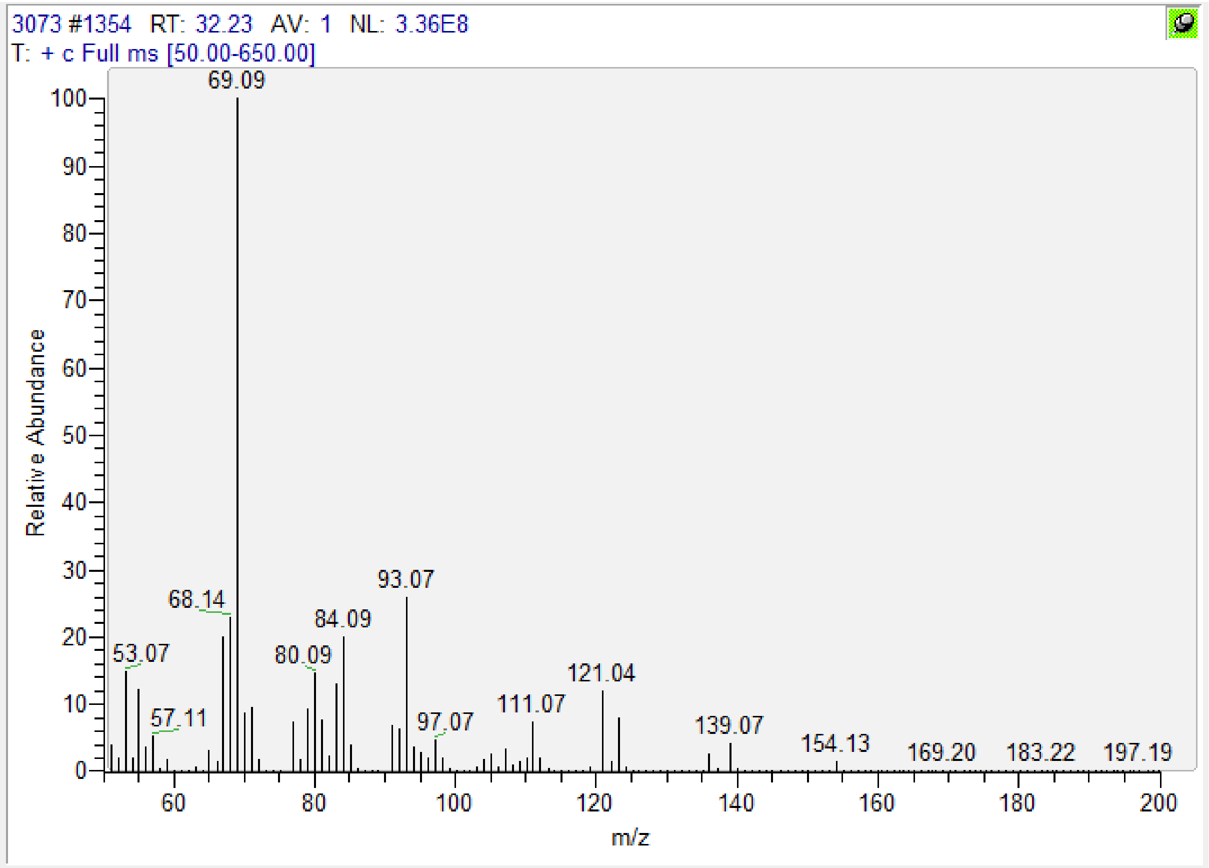

3073 #1354 RT: 32.23 AV: 1 NL: 3.36E8
T: + c Full ms [50.00-650.00]
Relative Abundance
m/z
69.09
68.14
53.07
57.11
80.09
84.09
93.07
97.07
111.07
121.04
139.07
154.13
169.20
183.22
197.19

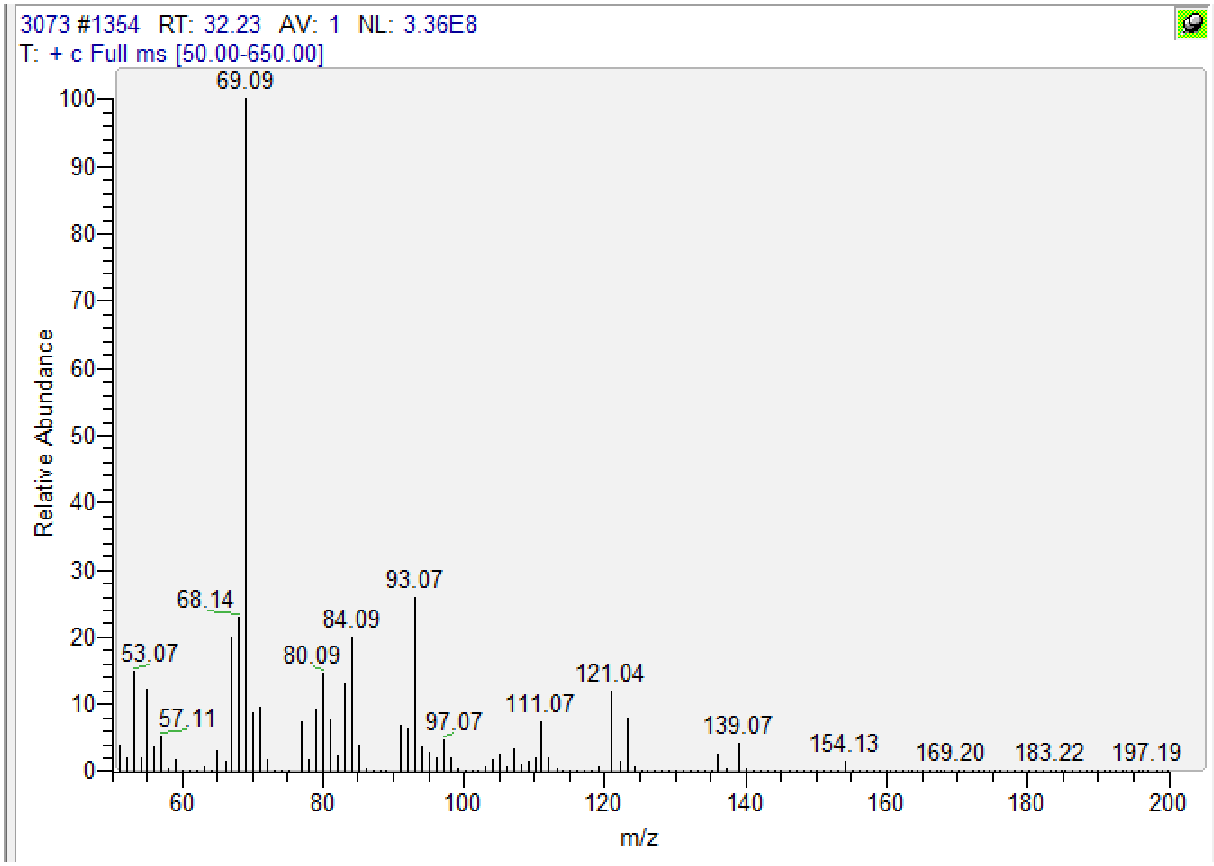
3073 #1354 RT: 32.23 AV: 1 NL: 3.36E8
T: + c Full ms [50.00-650.00]
Relative Abundance
m/z
69.09
68.14
53.07
57.11
80.09
84.09
93.07
97.07
111.07
121.04
139.07
154.13
169.20
183.22
197.19

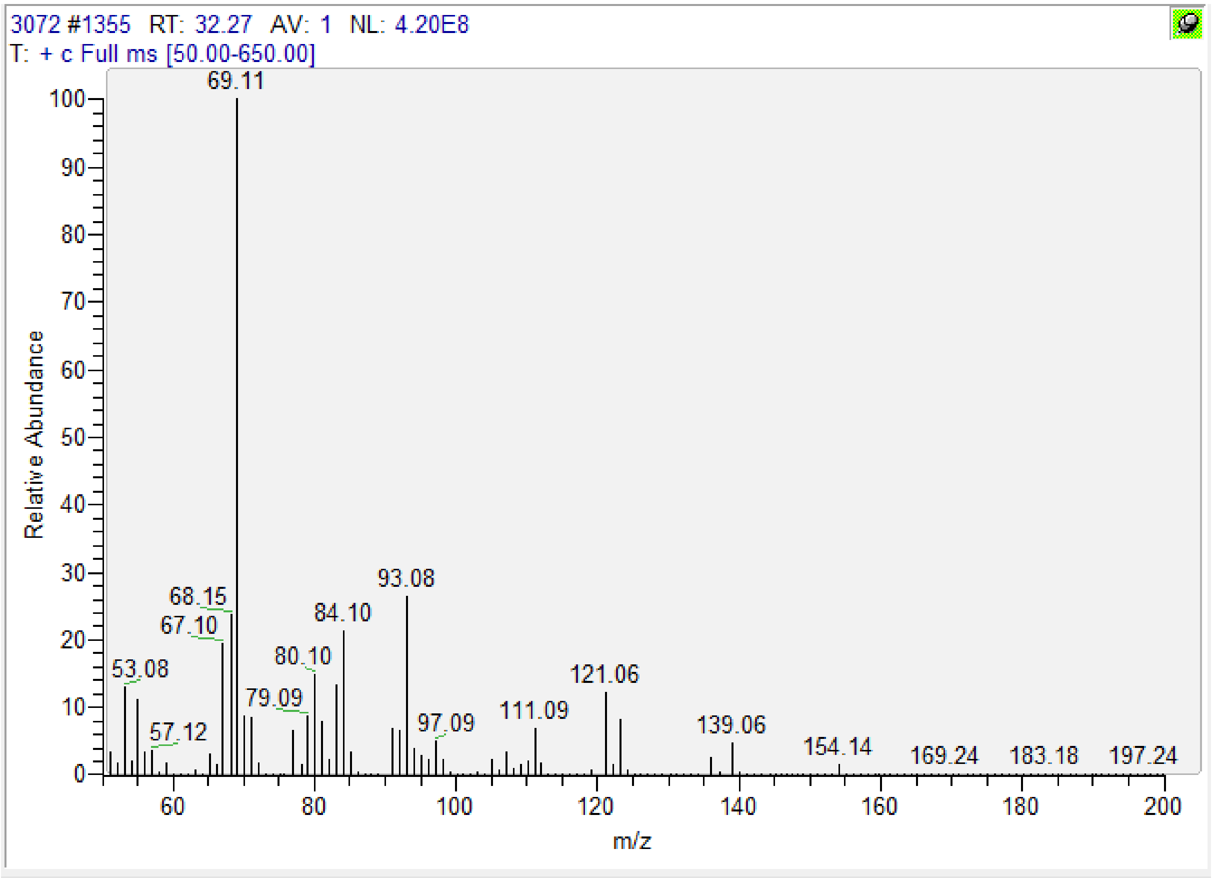
3072 #1355 RT: 32.27 AV: 1 NL: 4.20E8
T: + c Full ms [50.00-650.00]
69.11
68.15
67.10
53.08
57.12
79.09
80.10
84.10
93.08
97.09
111.09
121.06
139.06
154.14
169.24
183.18
197.24
Relative Abundance
m/z

In 3070: ND

Geraniol:

Reference:

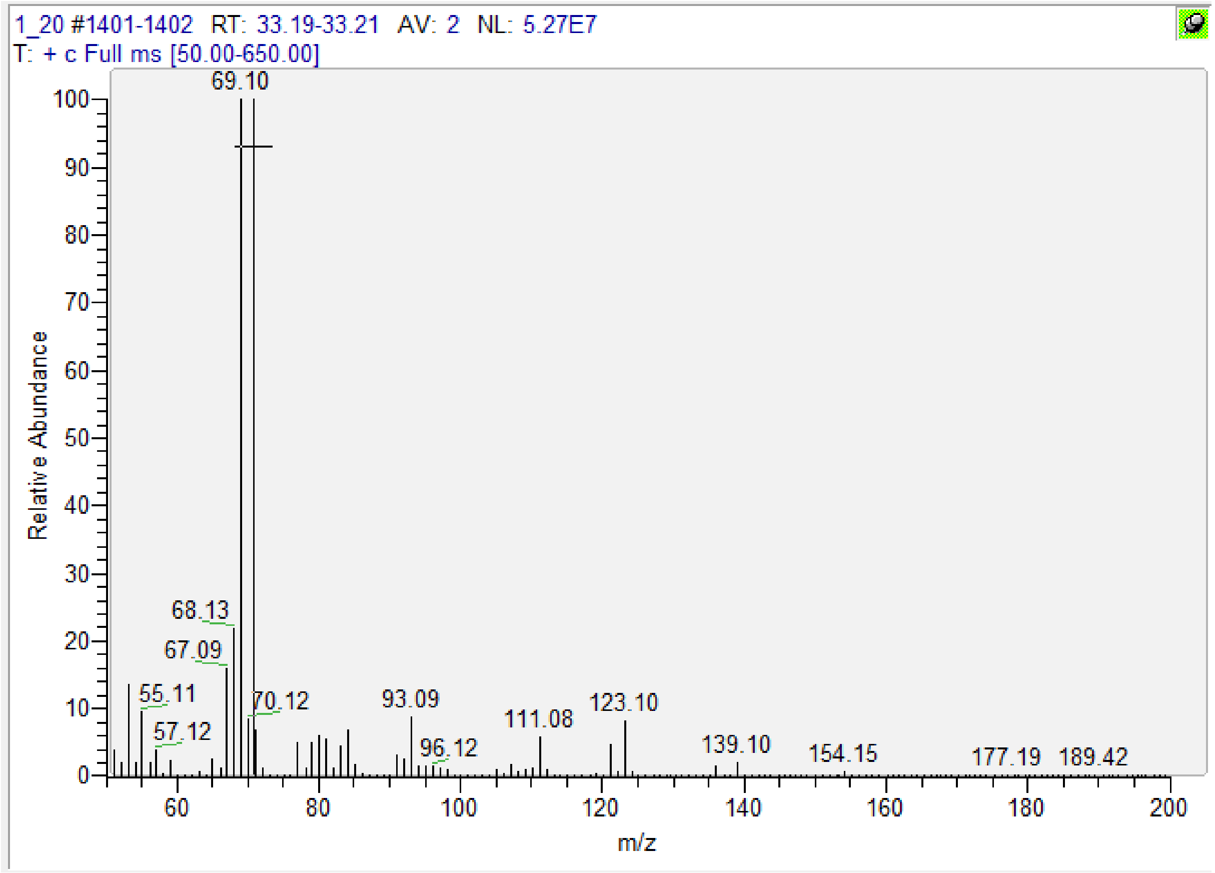

Samples:

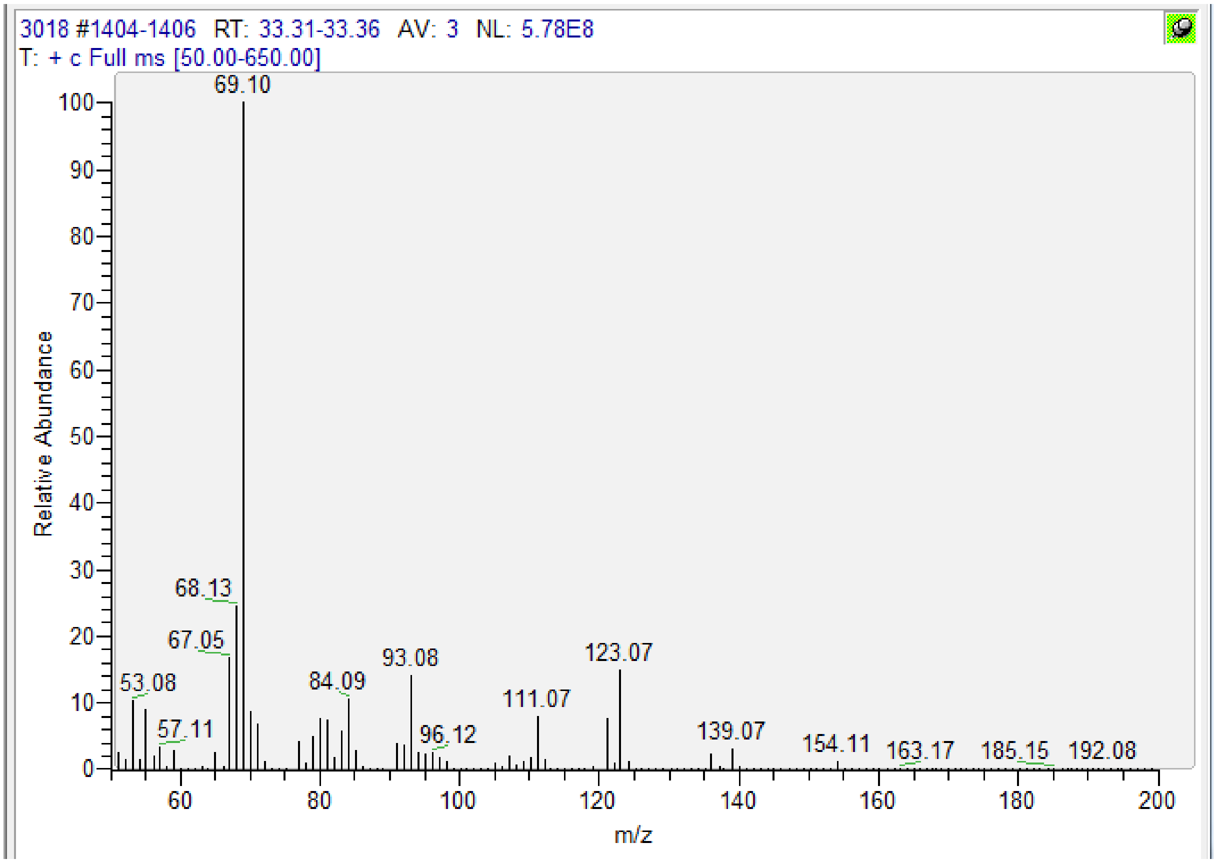

3018 #1404-1406 RT: 33.31-33.36 AV: 3 NL: 5.78E8
T: + c Full ms [50.00-650.00]
Relative Abundance
m/z
69.10
68.13
67.05
53.08
57.11
84.09
93.08
96.12
111.07
123.07
139.07
154.11
163.17
185.15
192.08

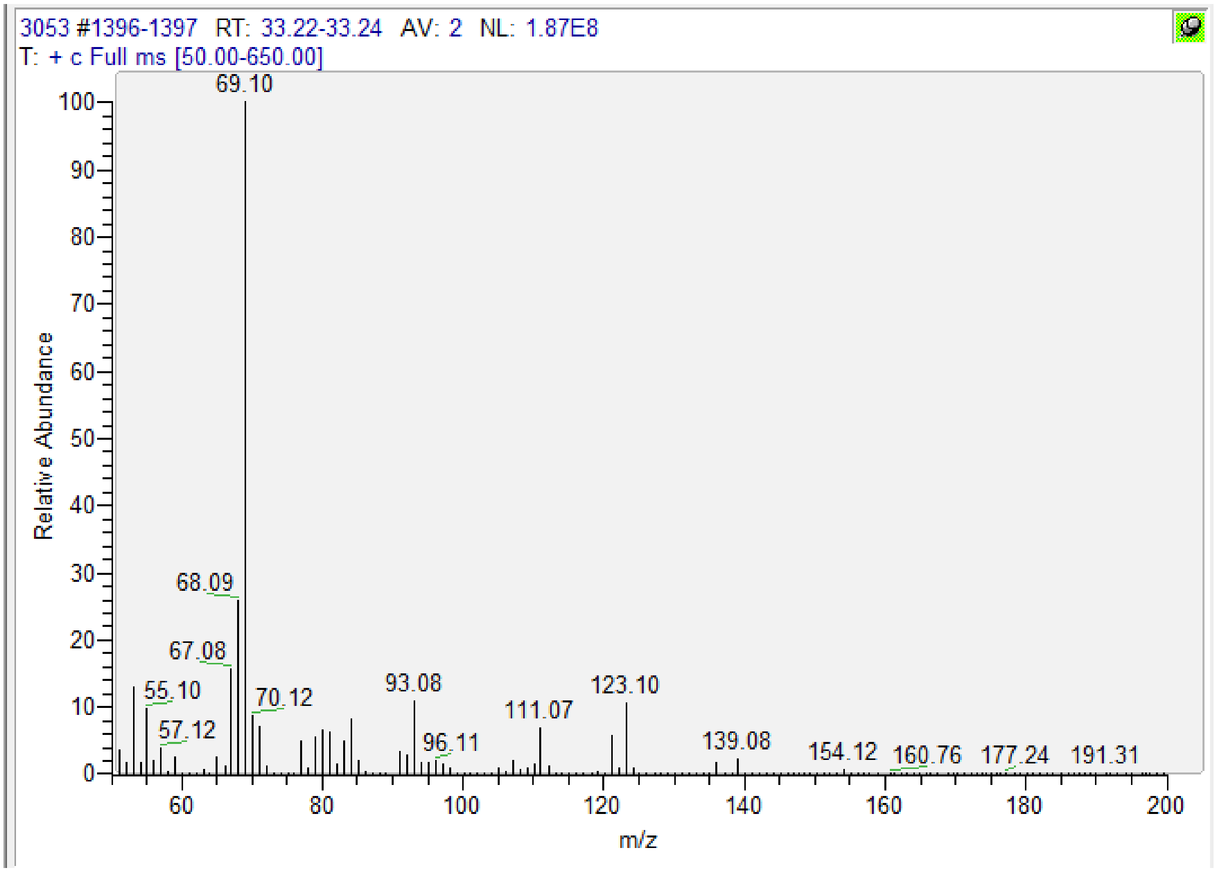
3053 #1396-1397 RT: 33.22-33.24 AV: 2 NL: 1.87E8
T: + c Full ms [50.00-650.00]
69.10
68.09
67.08
55.10
57.12
70.12
93.08
96.11
111.07
123.10
139.08
154.12
160.76
177.24
191.31
Relative Abundance
m/z

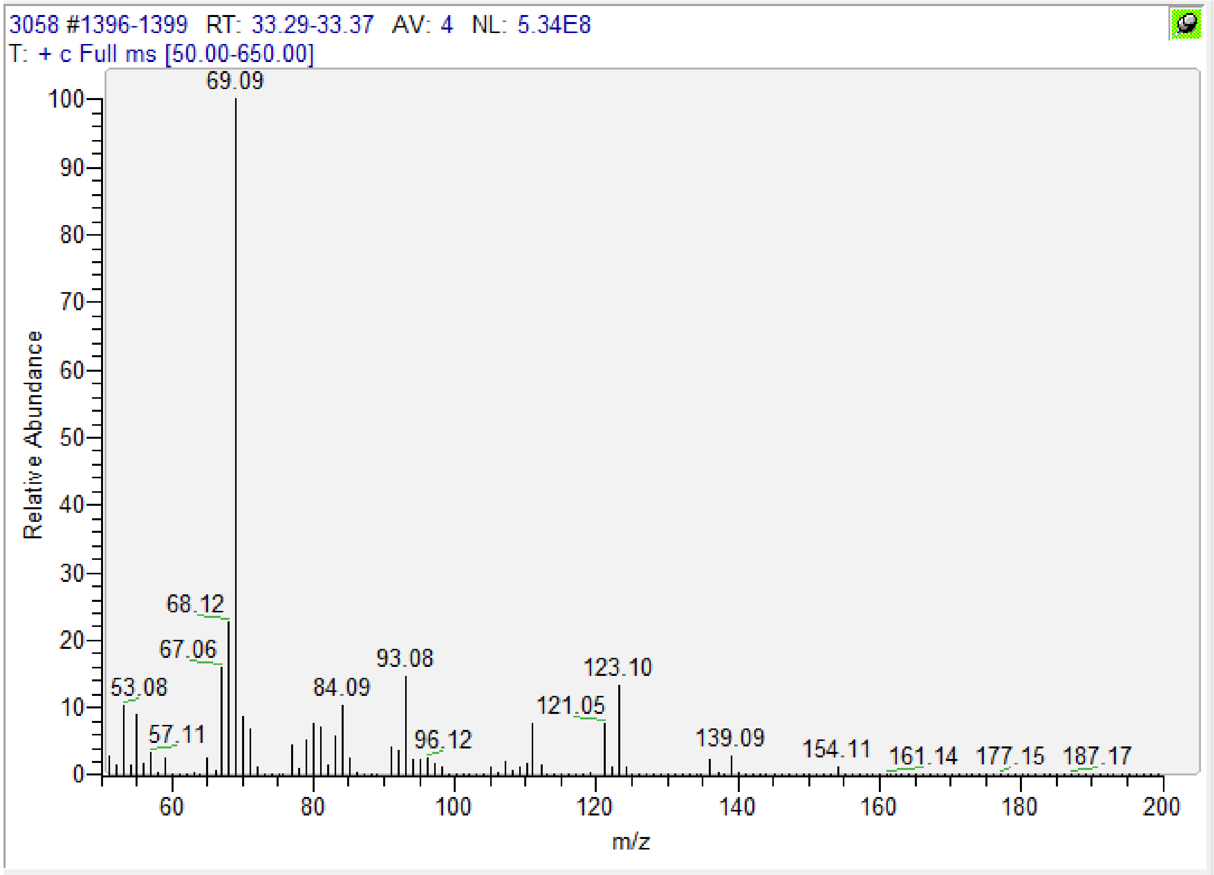

3058 #1396-1399 RT: 33.29-33.37 AV: 4 NL: 5.34E8
T: + c Full ms [50.00-650.00]
Relative Abundance
m/z
69.09
68.12
67.06
53.08
57.11
84.09
93.08
96.12
121.05
123.10
139.09
154.11
161.14
177.15
187.17

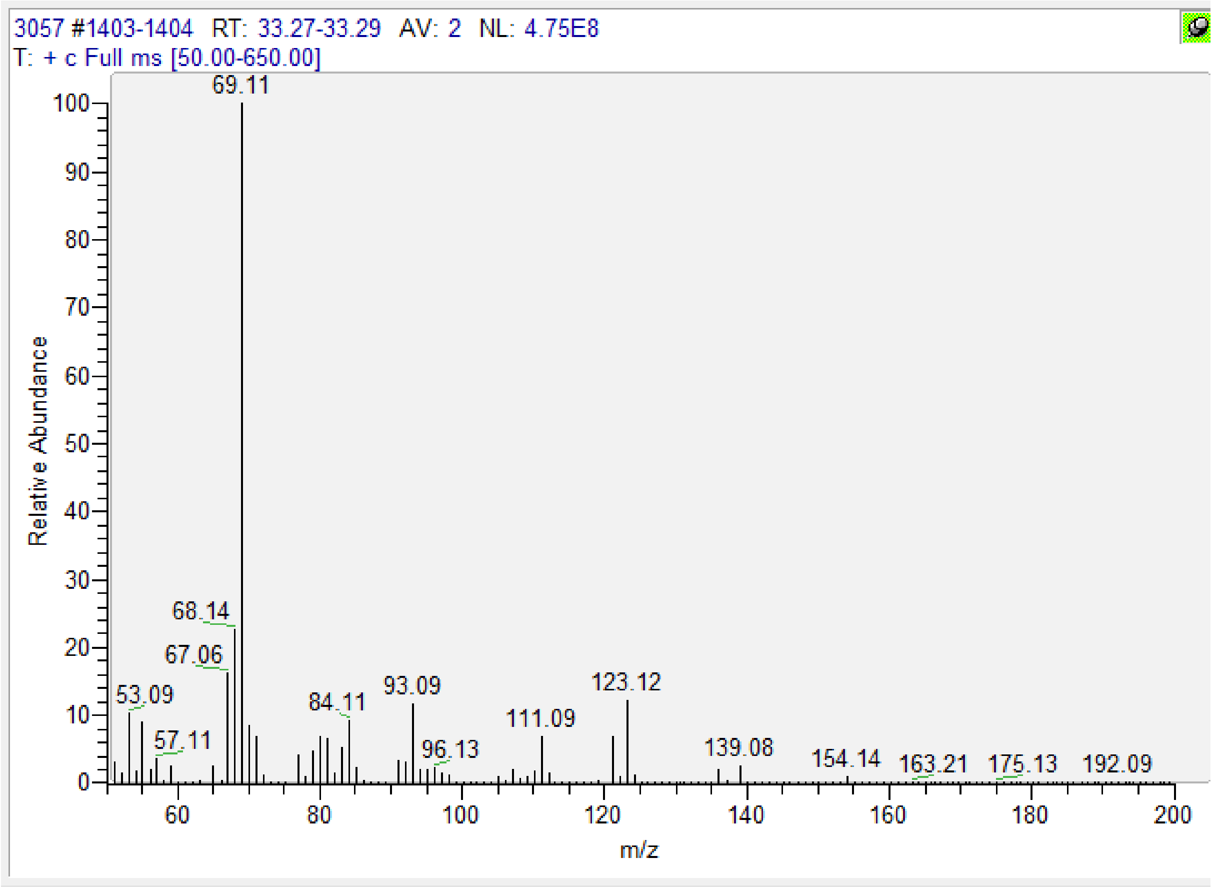

3057 #1403-1404 RT: 33.27-33.29 AV: 2 NL: 4.75E8
T: + c Full ms [50.00-650.00]
Relative Abundance
m/z
69.11
68.14
67.06
53.09
57.11
84.11
93.09
96.13
111.09
123.12
139.08
154.14
163.21
175.13
192.09

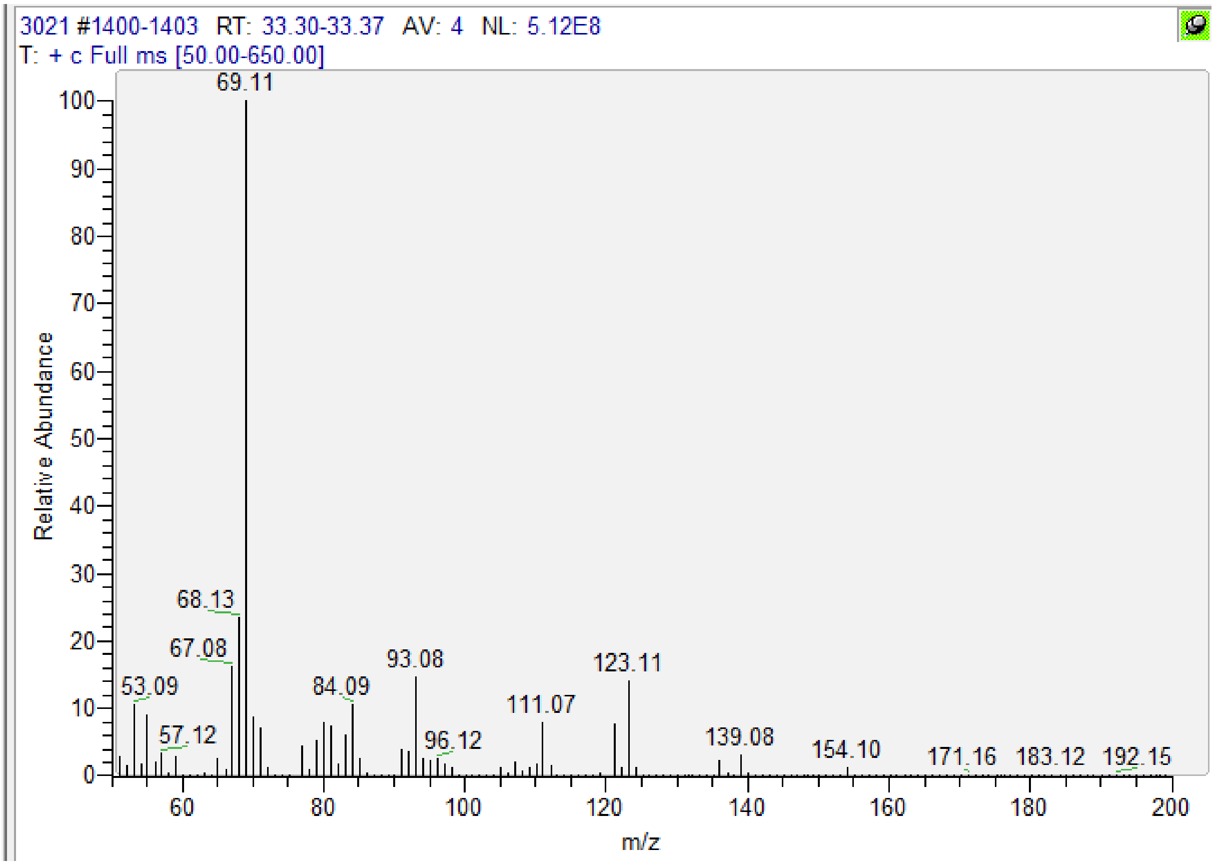
3021 #1400-1403 RT: 33.30-33.37 AV: 4 NL: 5.12E8
T: + c Full ms [50.00-650.00]
Relative Abundance
m/z
69.11
68.13
67.08
53.09
57.12
84.09
93.08
96.12
111.07
123.11
139.08
154.10
171.16
183.12
192.15

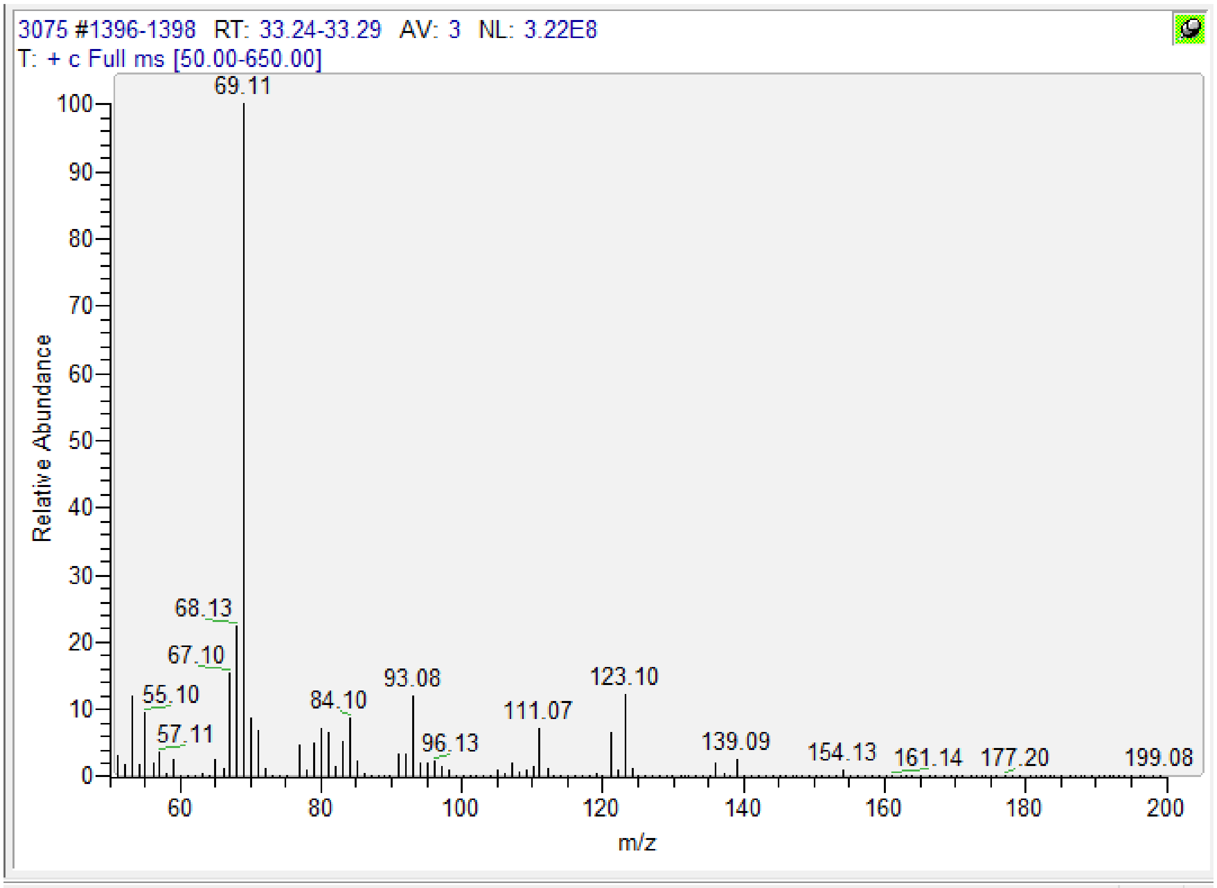

3075 #1396-1398 RT: 33.24-33.29 AV: 3 NL: 3.22E8
T: + c Full ms [50.00-650.00]
Relative Abundance
m/z
69.11
68.13
67.10
55.10
57.11
84.10
93.08
96.13
111.07
123.10
139.09
154.13
161.14
177.20
199.08

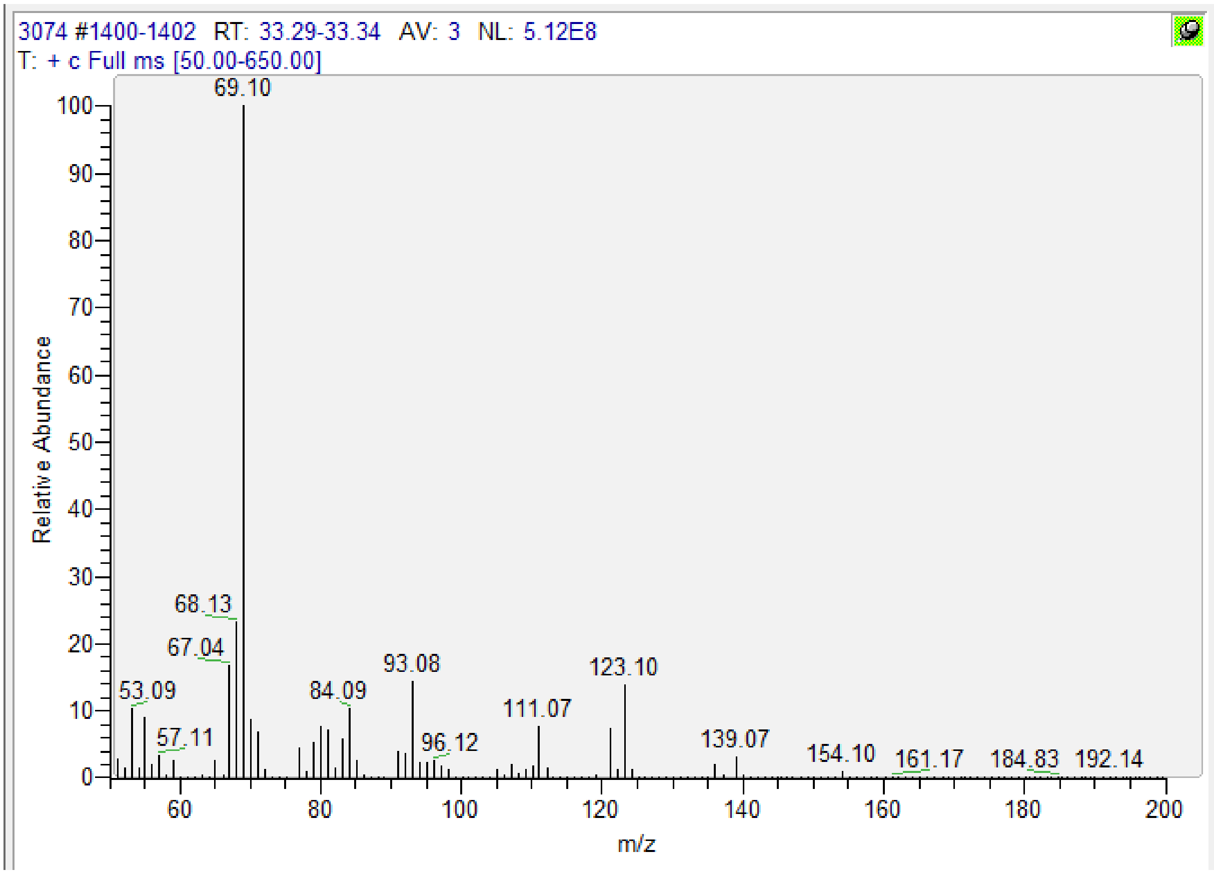

3074 #1400-1402 RT: 33.29-33.34 AV: 3 NL: 5.12E8
T: + c Full ms [50.00-650.00]
Relative Abundance
m/z
69.10
68.13
67.04
53.09
57.11
84.09
93.08
96.12
111.07
123.10
139.07
154.10
161.17
184.83
192.14

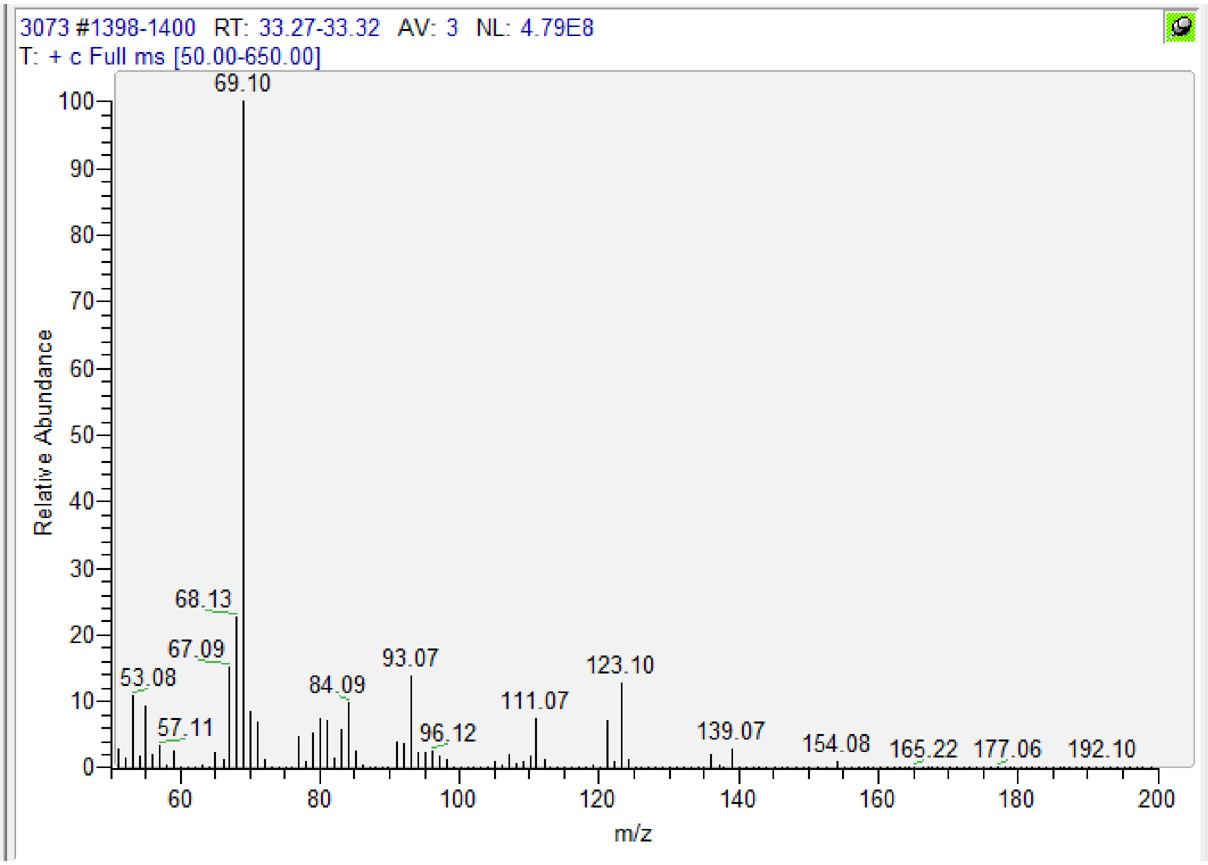
3073 #1398-1400 RT: 33.27-33.32 AV: 3 NL: 4.79E8
T: + c Full ms [50.00-650.00]
Relative Abundance
m/z
69.10
68.13
67.09
53.08
57.11
84.09
93.07
96.12
111.07
123.10
139.07
154.08
165.22
177.06
192.10

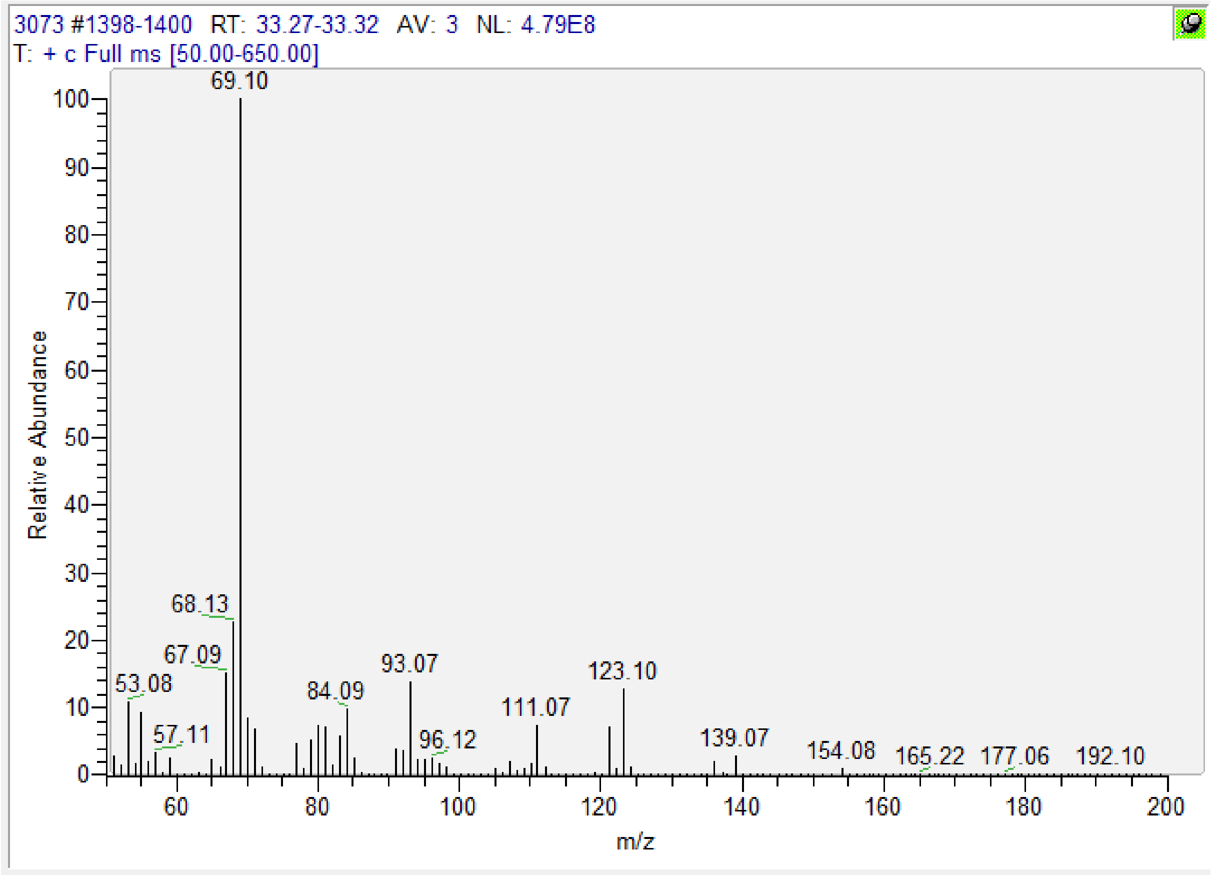

3073 #1398-1400 RT: 33.27-33.32 AV: 3 NL: 4.79E8
T: + c Full ms [50.00-650.00]
Relative Abundance
m/z
69.10
68.13
67.09
53.08
57.11
84.09
93.07
96.12
111.07
123.10
139.07
154.08
165.22
177.06
192.10

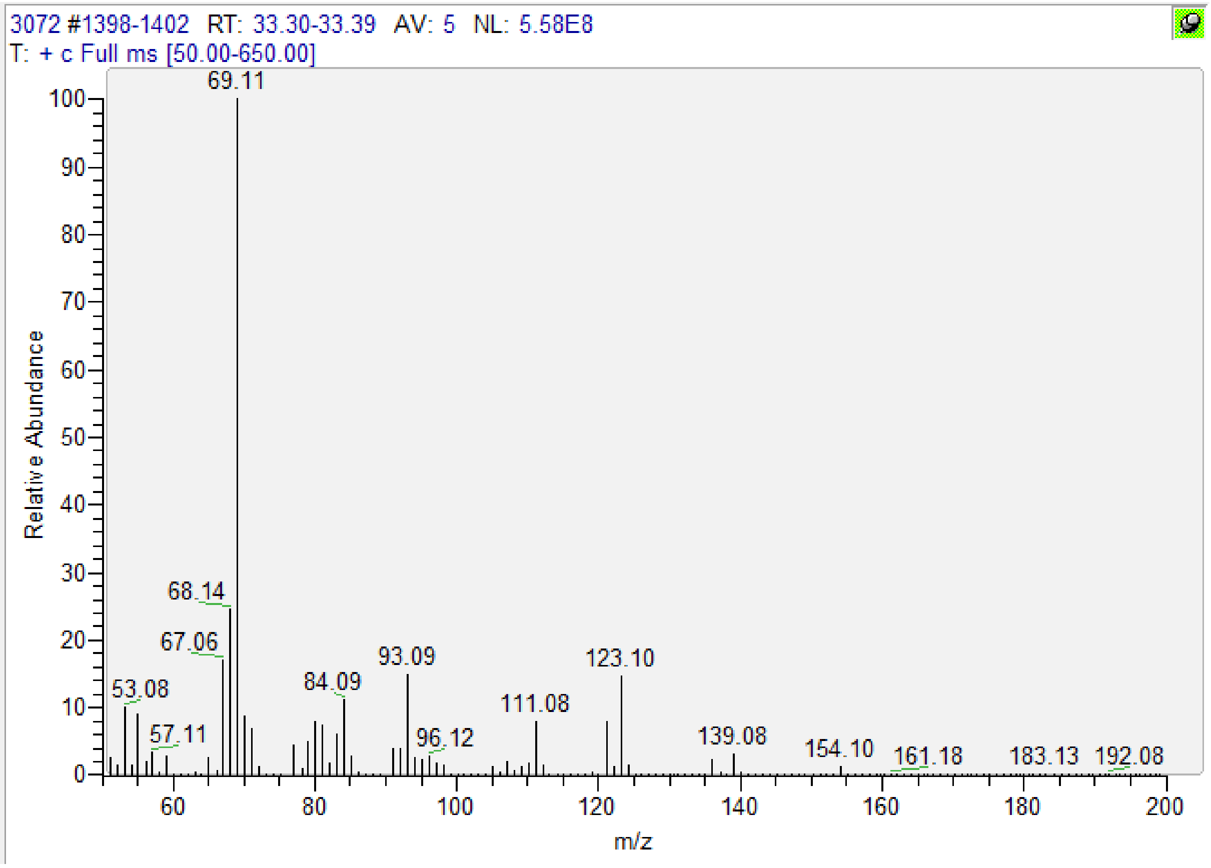
3072 #1398-1402 RT: 33.30-33.39 AV: 5 NL: 5.58E8
T: + c Full ms [50.00-650.00]
Relative Abundance
m/z
69.11
68.14
67.06
53.08
57.11
84.09
93.09
96.12
111.08
123.10
139.08
154.10
161.18
183.13
192.08
0
10
20
30
40
50
60
70
80
90
100
60
80
100
120
140
160
180
200

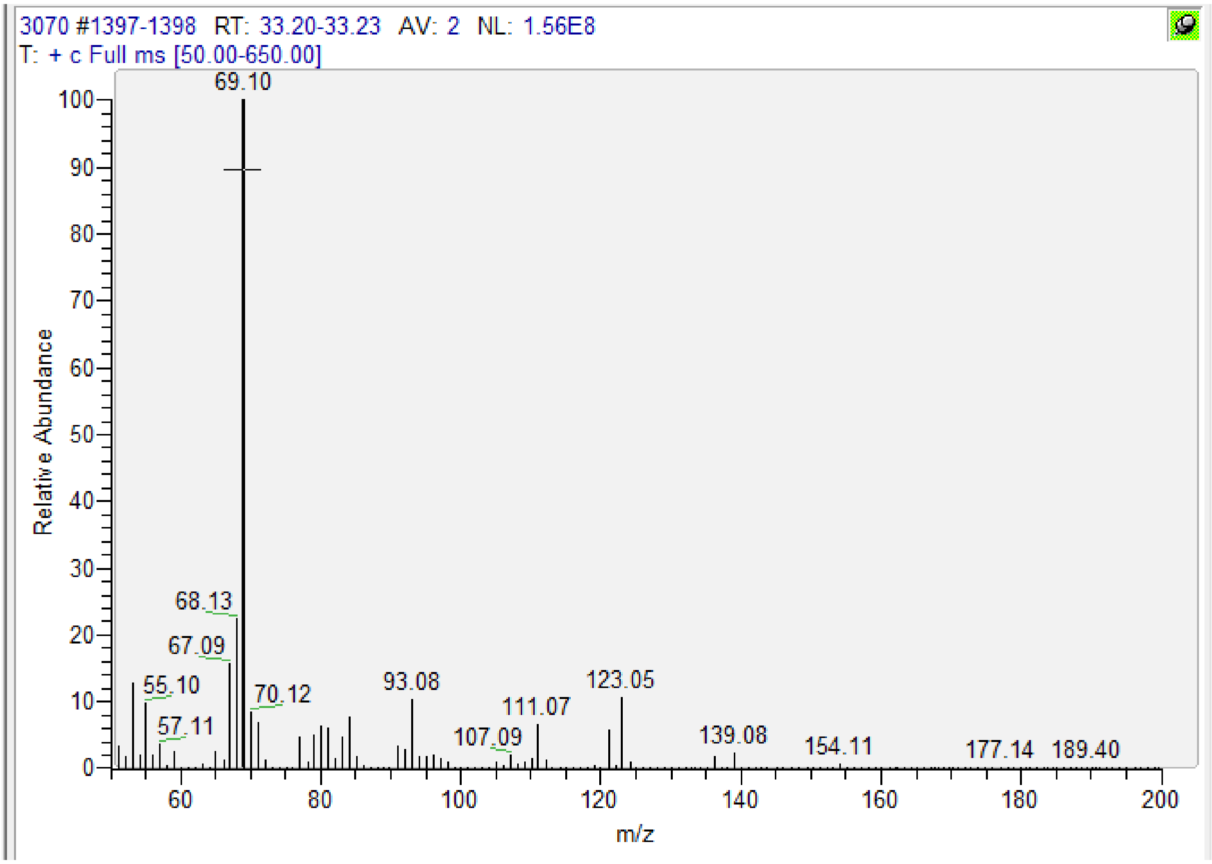
3070 #1397-1398 RT: 33.20-33.23 AV: 2 NL: 1.56E8
T: + c Full ms [50.00-650.00]
69.10
68.13
67.09
55.10
57.11
70.12
93.08
107.09
111.07
123.05
139.08
154.11
177.14
189.40
Relative Abundance
m/z

Methyleugenol

Reference:

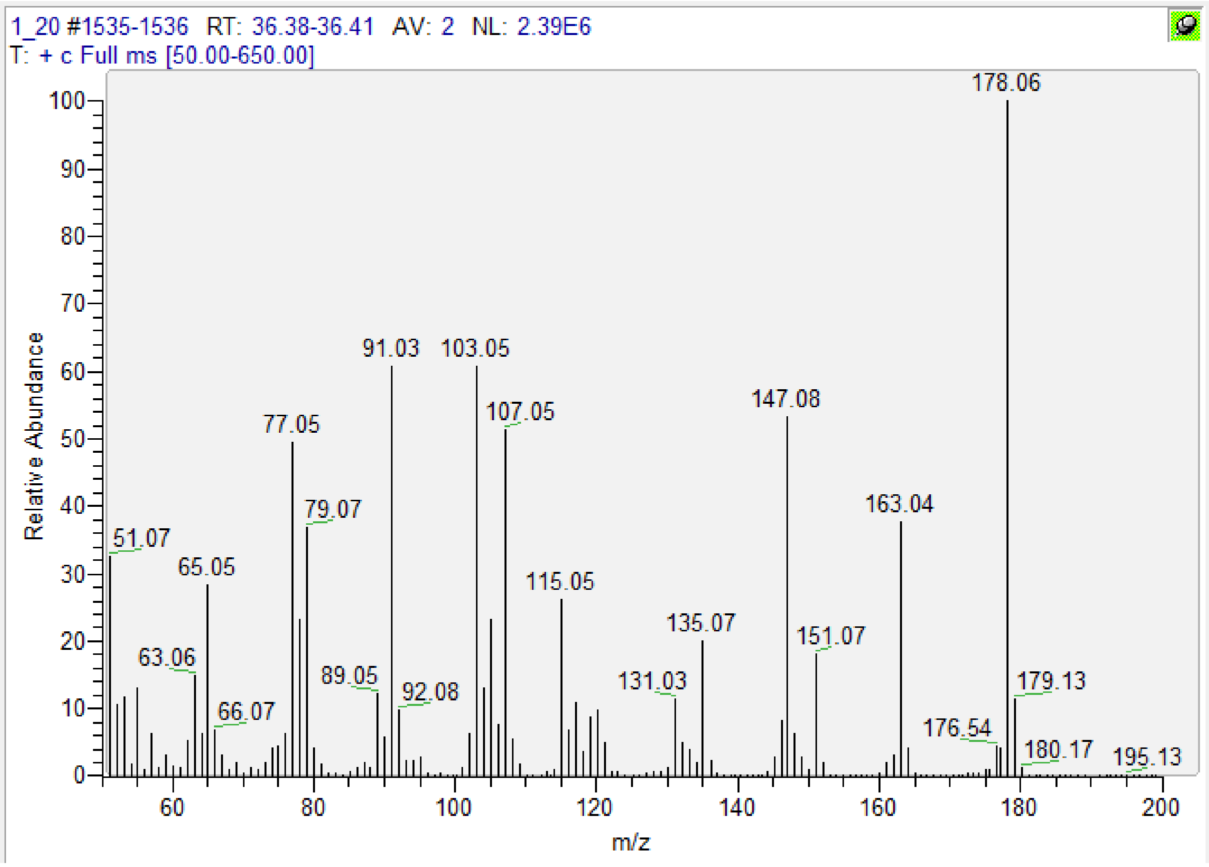

Samples:¨

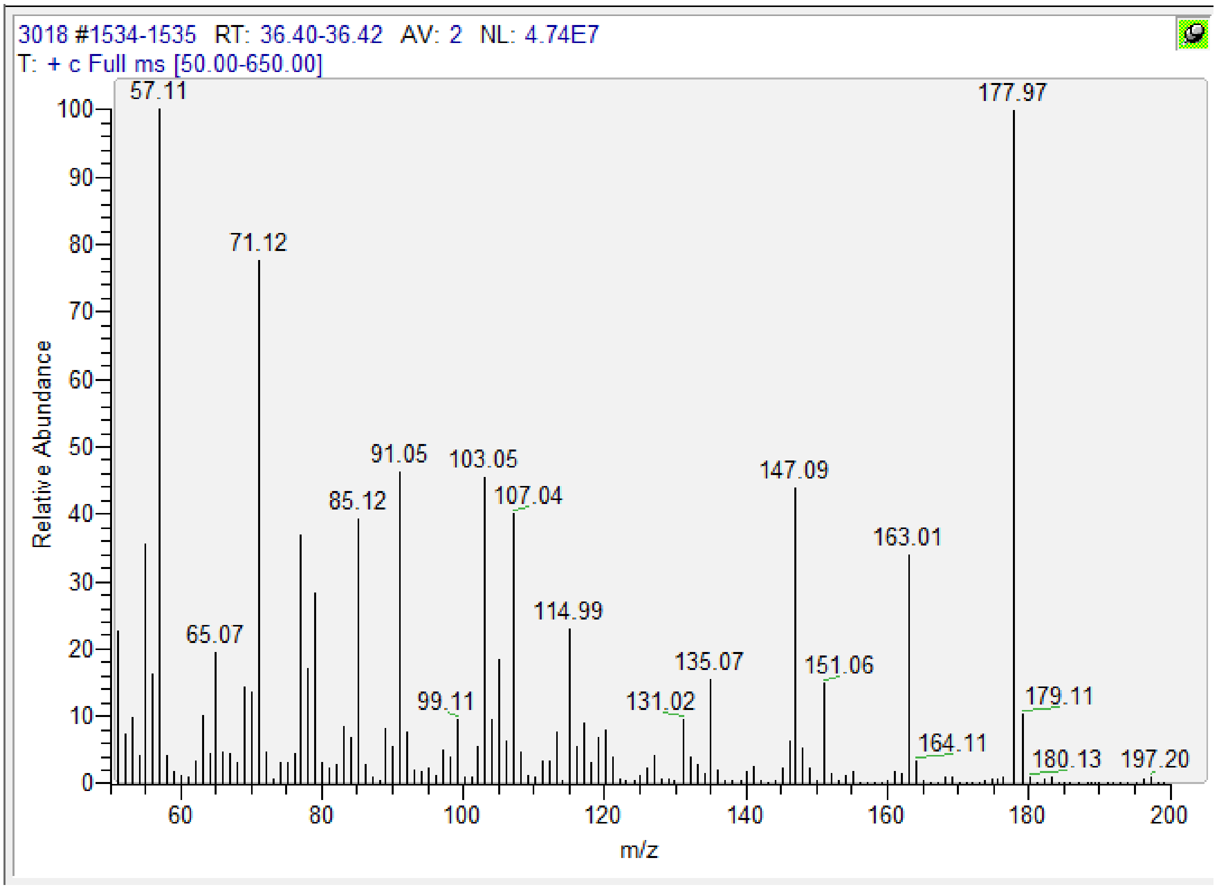

3018 #1534-1535 RT: 36.40-36.42 AV: 2 NL: 4.74E7
T: + c Full ms [50.00-650.00]
Relative Abundance
m/z
57.11
177.97
71.12
91.05
103.05
107.04
85.12
147.09
163.01
114.99
65.07
135.07
151.06
99.11
131.02
179.11
164.11
180.13
197.20

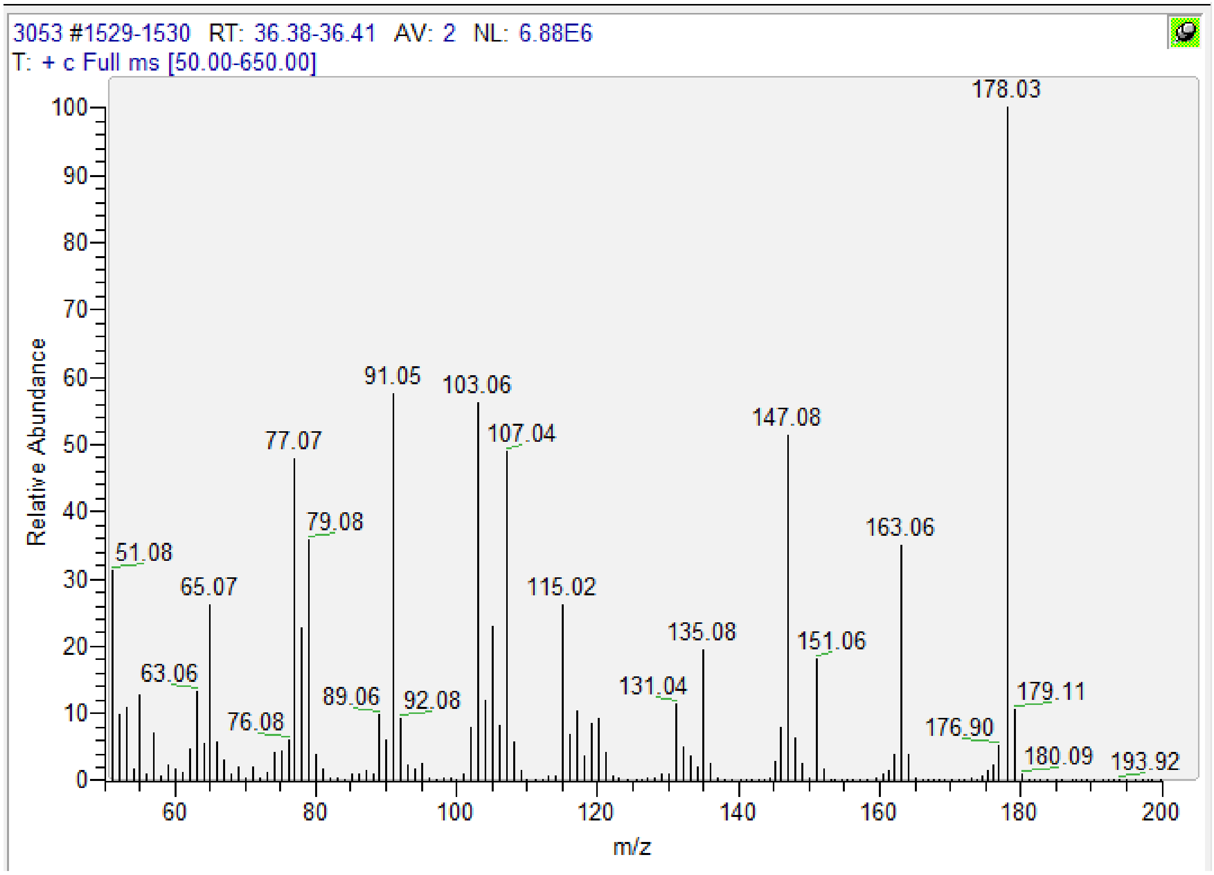
3053 #1529-1530 RT: 36.38-36.41 AV: 2 NL: 6.88E6
T: + c Full ms [50.00-650.00]
Relative Abundance
m/z
178.03
91.05
103.06
107.04
147.08
77.07
79.08
163.06
51.08
65.07
115.02
135.08
151.06
63.06
131.04
179.11
89.06
92.08
76.08
176.90
180.09
193.92

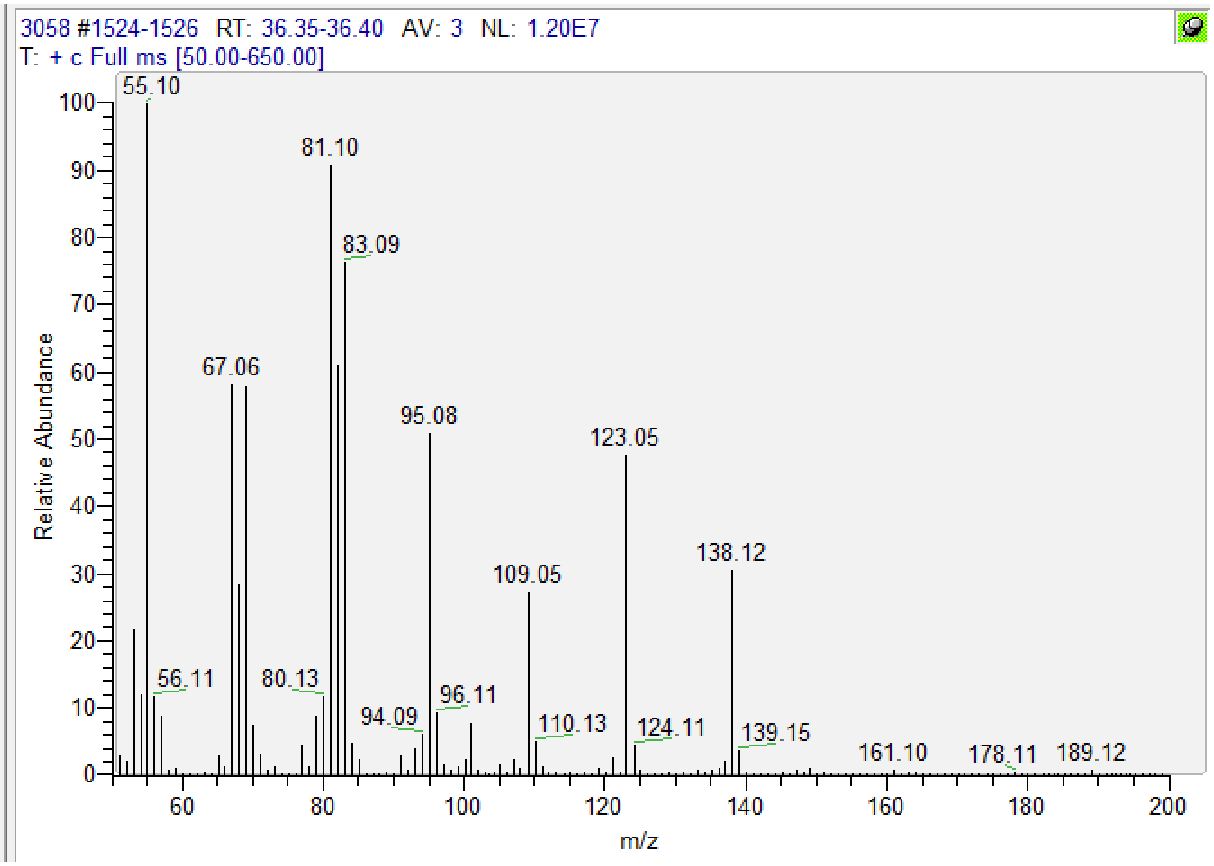

3058 #1524-1526 RT: 36.35-36.40 AV: 3 NL: 1.20E7
T: + c Full ms [50.00-650.00]
Relative Abundance
m/z
55.10
81.10
83.09
67.06
95.08
123.05
138.12
109.05
56.11
80.13
96.11
94.09
110.13
124.11
139.15
161.10
178.11
189.12

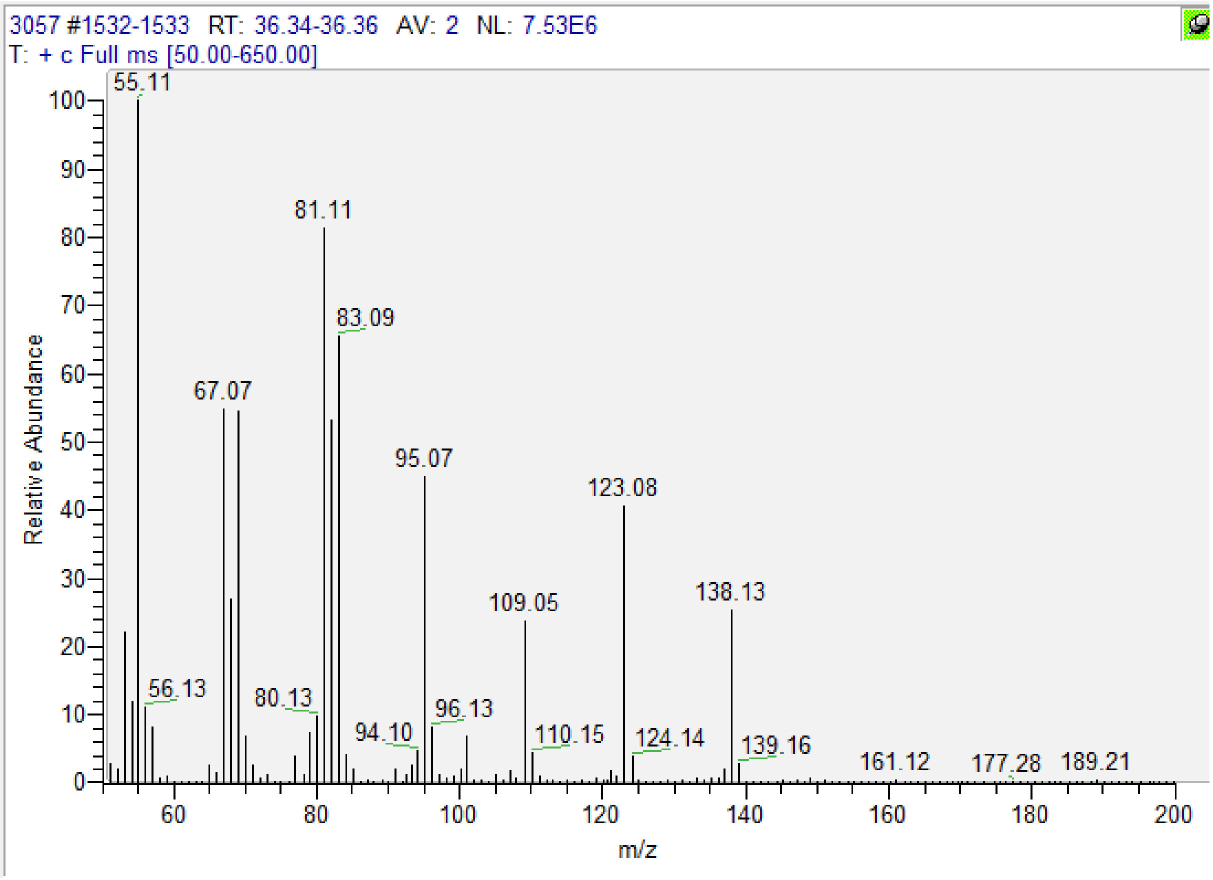

3057 #1532-1533 RT: 36.34-36.36 AV: 2 NL: 7.53E6
T: + c Full ms [50.00-650.00]
Relative Abundance
m/z
55.11
81.11
83.09
67.07
95.07
123.08
109.05
138.13
56.13
80.13
96.13
94.10
110.15
124.14
139.16
161.12
177.28
189.21

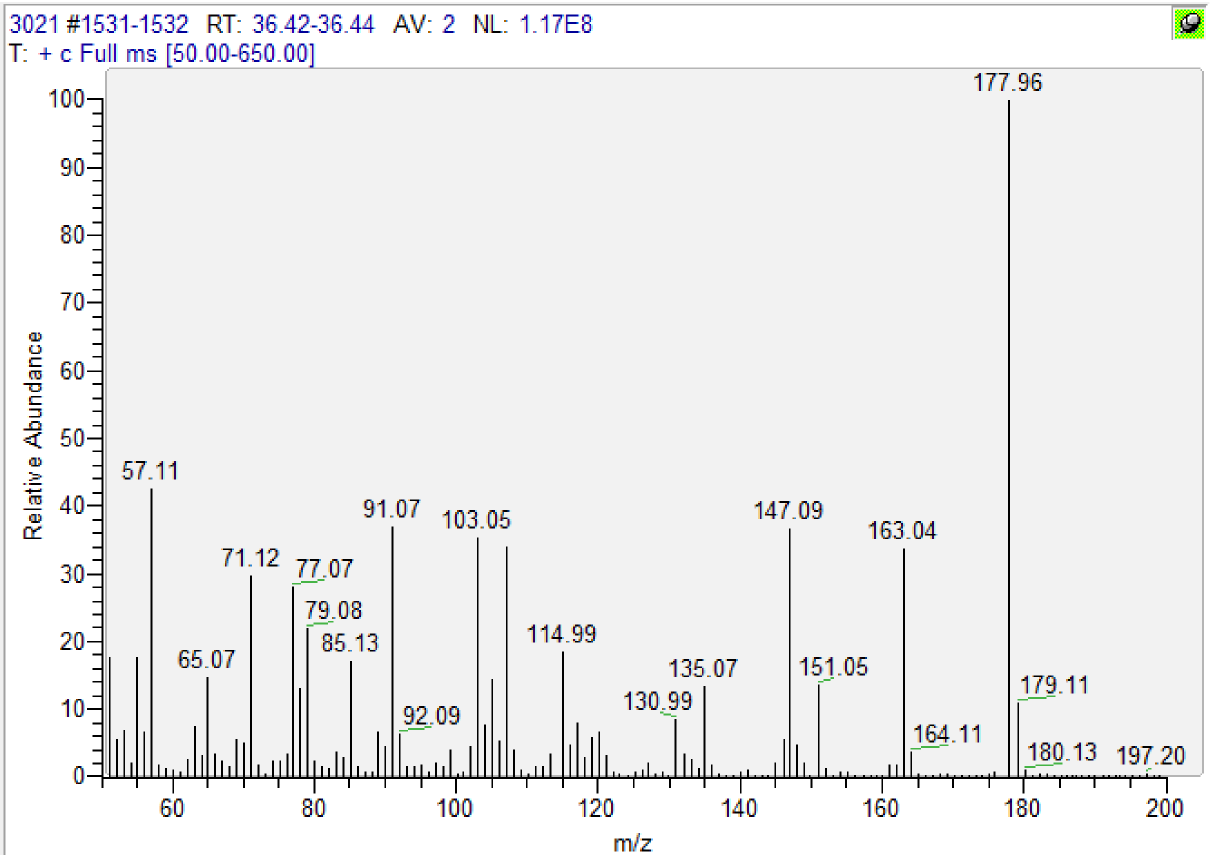
3021 #1531-1532 RT: 36.42-36.44 AV: 2 NL: 1.17E8
T: + c Full ms [50.00-650.00]
Relative Abundance
m/z
177.96
57.11
91.07
103.05
71.12
77.07
79.08
85.13
65.07
114.99
147.09
163.04
135.07
151.05
130.99
92.09
179.11
164.11
180.13
197.20

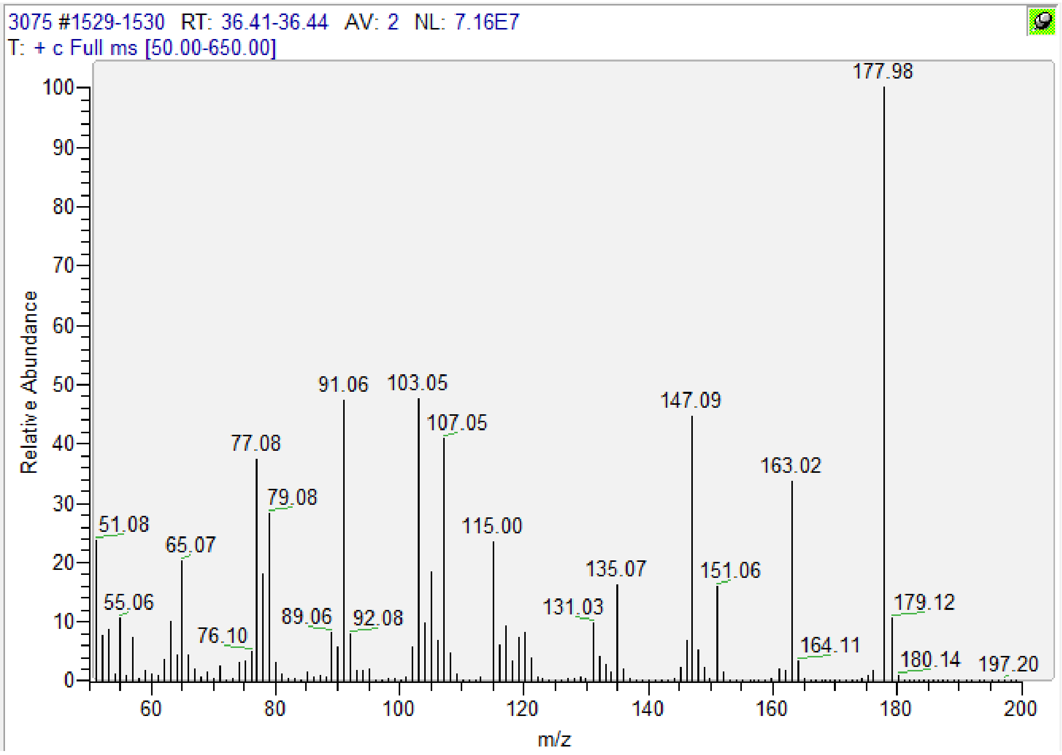
3075 #1529-1530 RT: 36.41-36.44 AV: 2 NL: 7.16E7
T: + c Full ms [50.00-650.00]
Relative Abundance
m/z
177.98
91.06
103.05
107.05
147.09
77.08
163.02
79.08
115.00
51.08
65.07
135.07
151.06
55.06
131.03
89.06
92.08
179.12
76.10
164.11
180.14
197.20

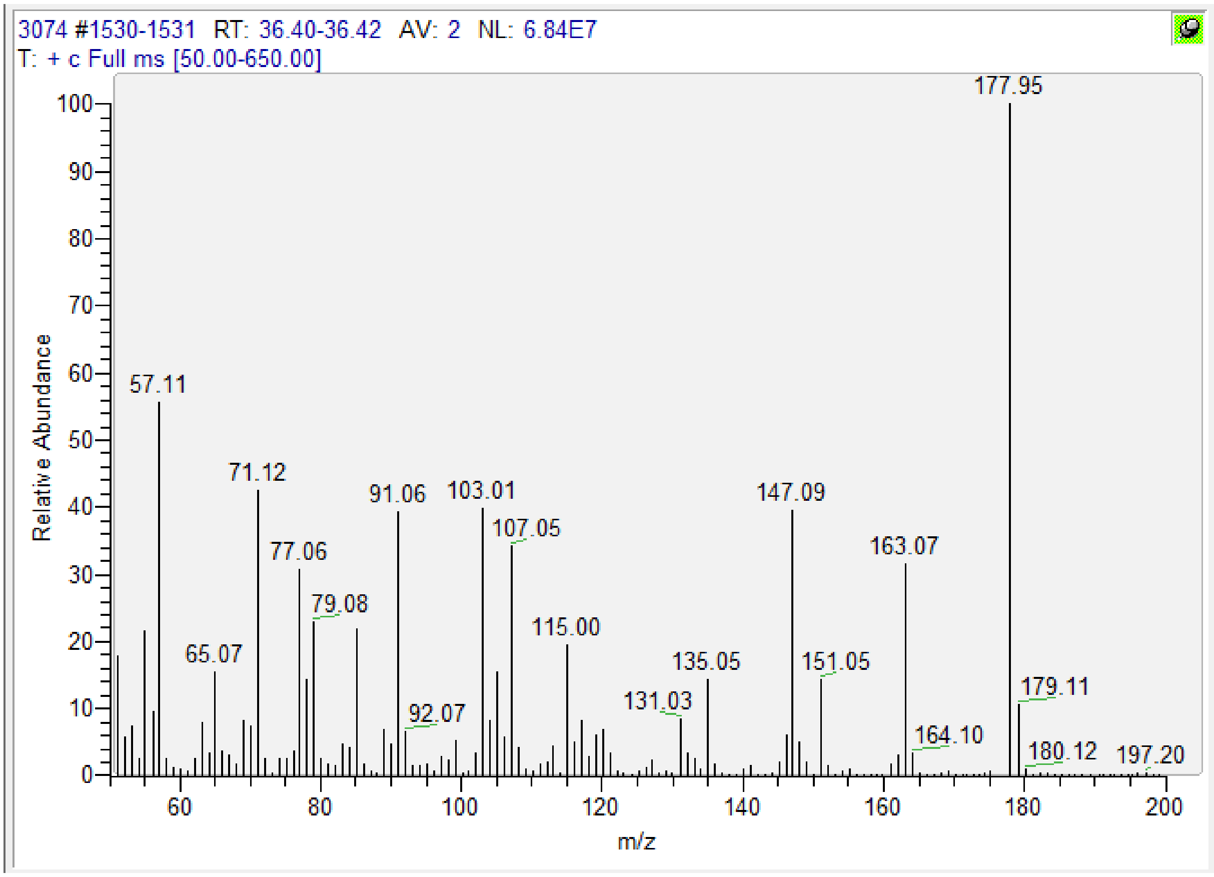
3074 #1530-1531 RT: 36.40-36.42 AV: 2 NL: 6.84E7
T: + c Full ms [50.00-650.00]
Relative Abundance
m/z
177.95
57.11
71.12
77.06
79.08
65.07
91.06
92.07
103.01
107.05
115.00
131.03
135.05
147.09
151.05
163.07
164.10
179.11
180.12
197.20

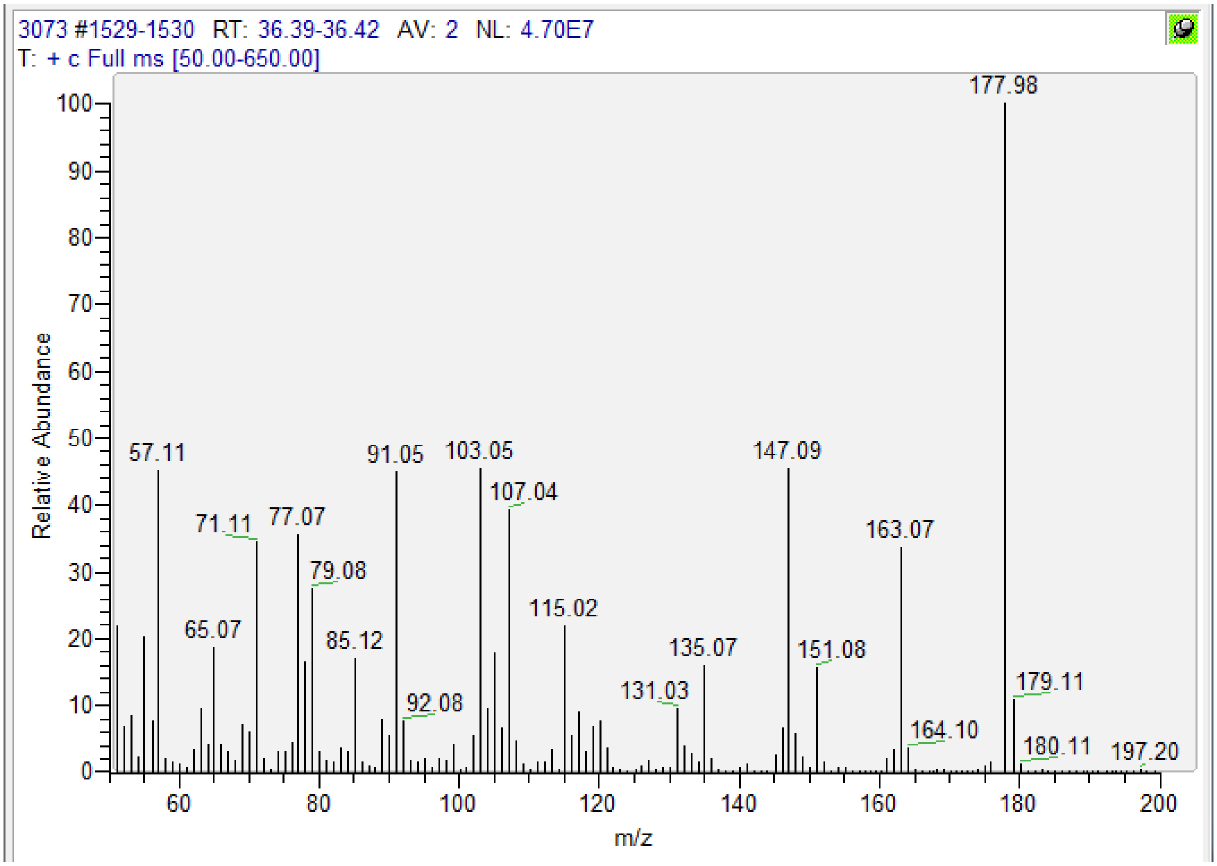
3073 #1529-1530 RT: 36.39-36.42 AV: 2 NL: 4.70E7
T: + c Full ms [50.00-650.00]
Relative Abundance
m/z
177.98
57.11
91.05
103.05
147.09
107.04
71.11
77.07
163.07
79.08
115.02
65.07
85.12
135.07
151.08
131.03
92.08
179.11
164.10
180.11
197.20

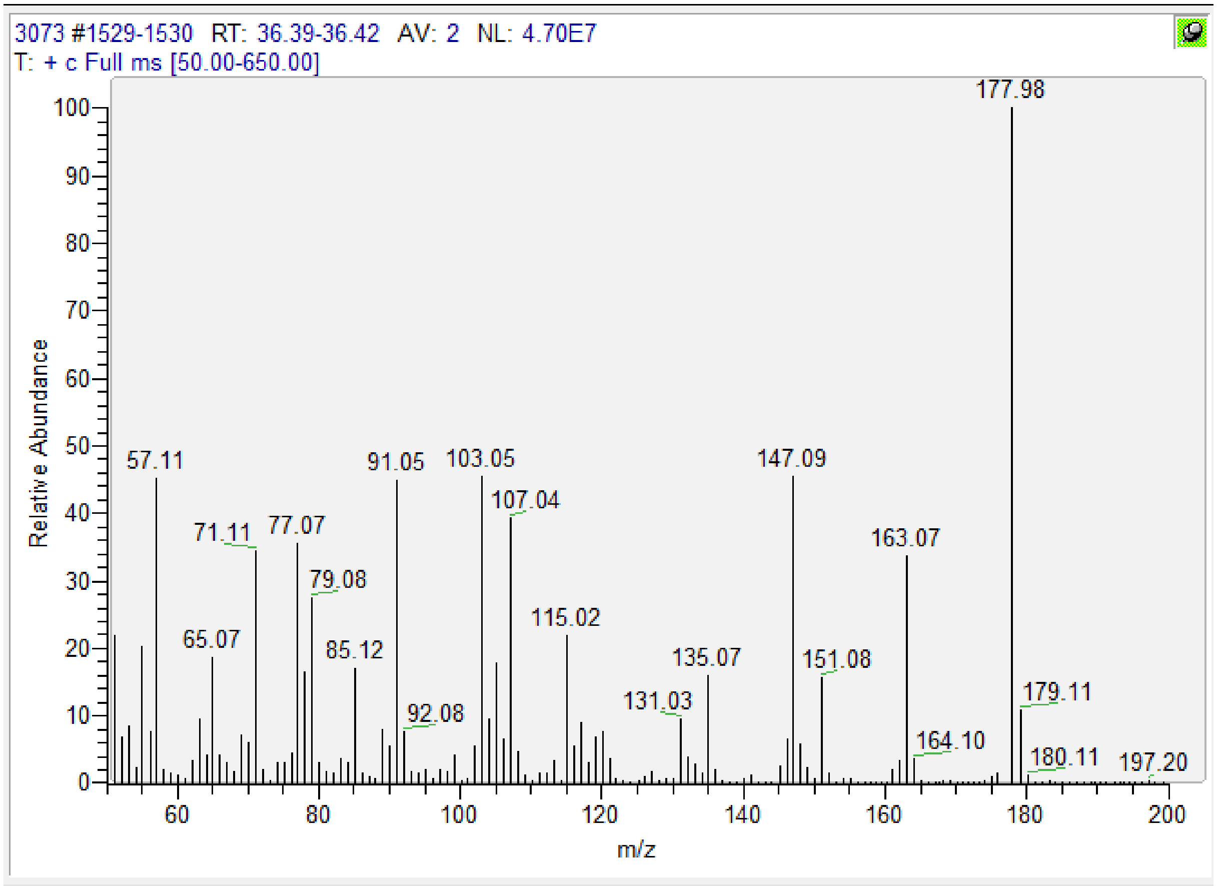
3073 #1529-1530 RT: 36.39-36.42 AV: 2 NL: 4.70E7
T: + c Full ms [50.00-650.00]
Relative Abundance
m/z
177.98
57.11
91.05
103.05
147.09
107.04
71.11
77.07
163.07
79.08
115.02
65.07
85.12
135.07
151.08
131.03
179.11
92.08
164.10
180.11
197.20

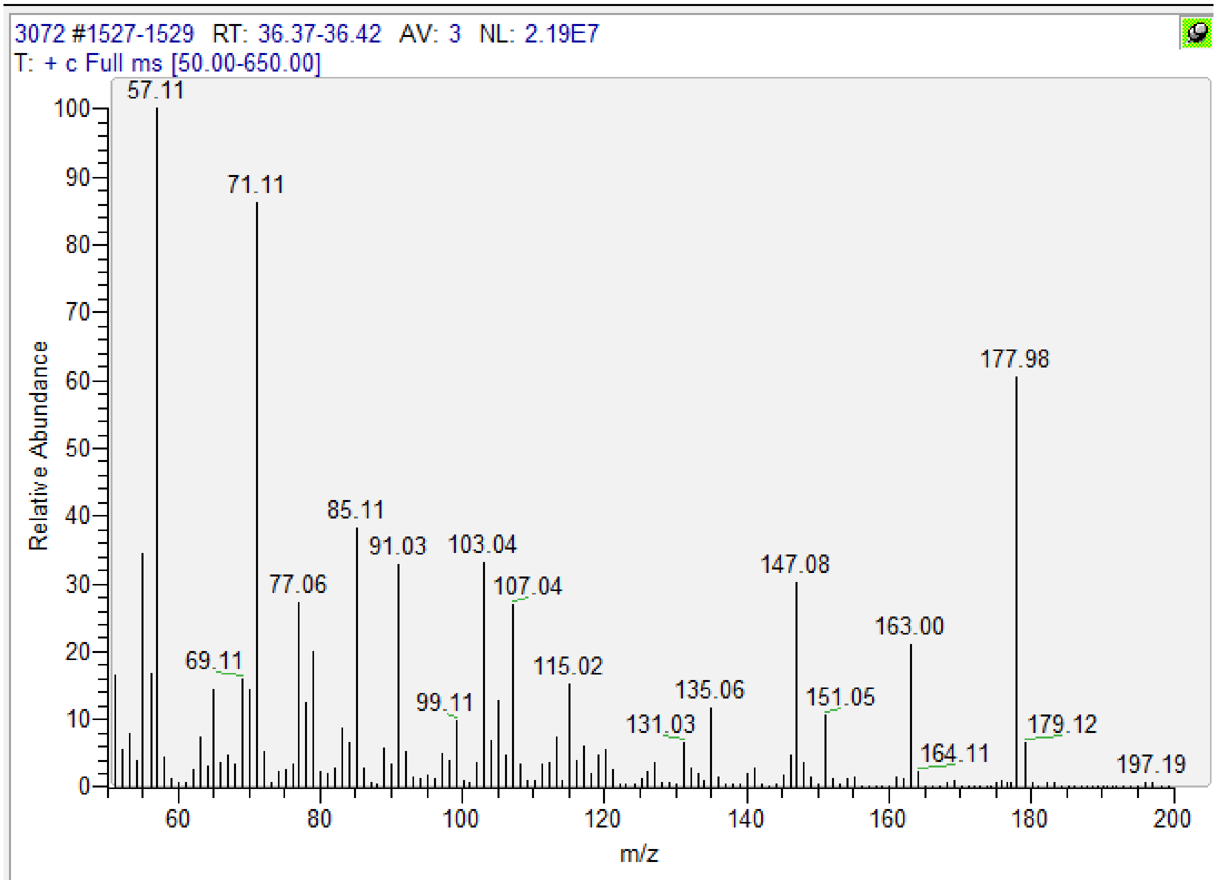
3072 #1527-1529 RT: 36.37-36.42 AV: 3 NL: 2.19E7
T: + c Full ms [50.00-650.00]
57.11
71.11
85.11
91.03
103.04
77.06
107.04
69.11
115.02
99.11
131.03
135.06
147.08
151.05
163.00
164.11
177.98
179.12
197.19
Relative Abundance
m/z

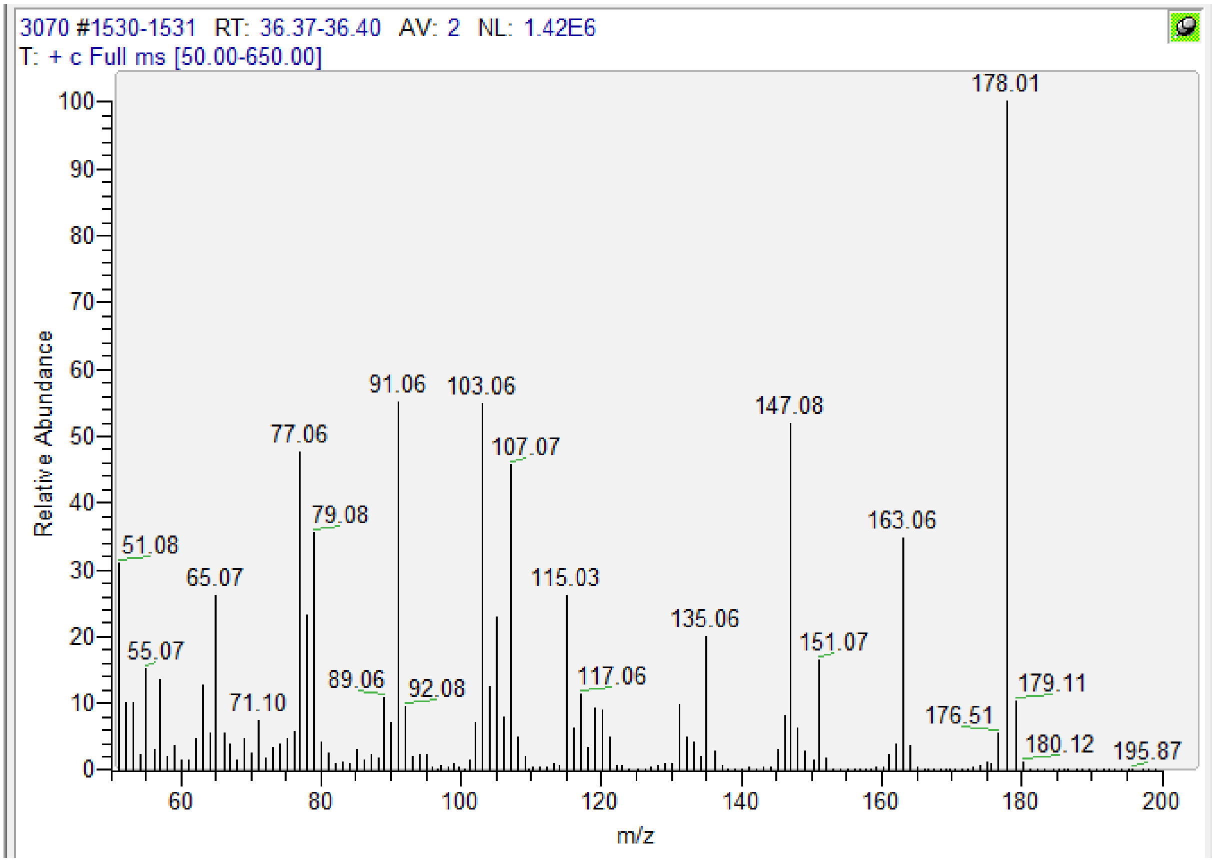
3070 #1530-1531 RT: 36.37-36.40 AV: 2 NL: 1.42E6
T: + c Full ms [50.00-650.00]
Relative Abundance
m/z
178.01
91.06
103.06
147.08
77.06
107.07
79.08
51.08
65.07
115.03
163.06
135.06
55.07
151.07
117.06
89.06
92.08
71.10
179.11
176.51
180.12
195.87

Eugenol

Reference:

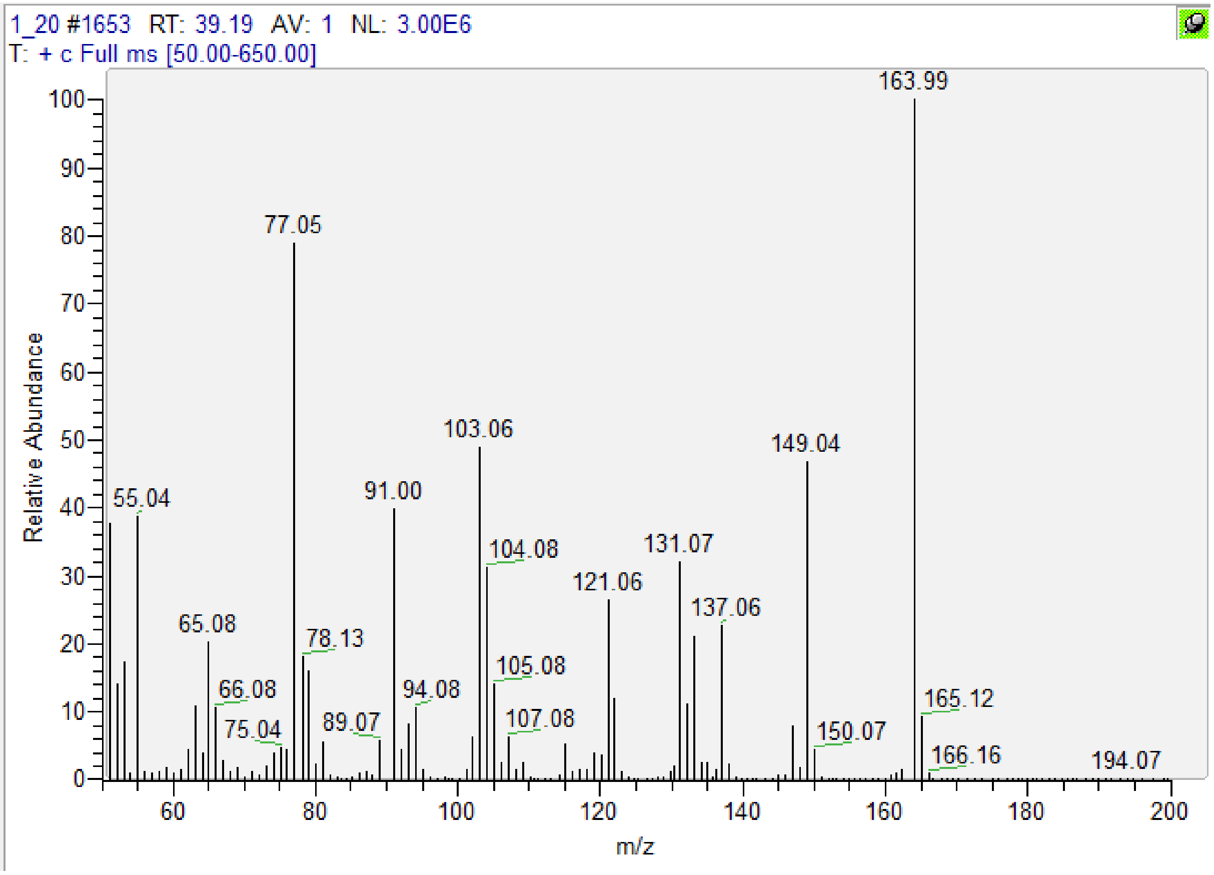

Samples:

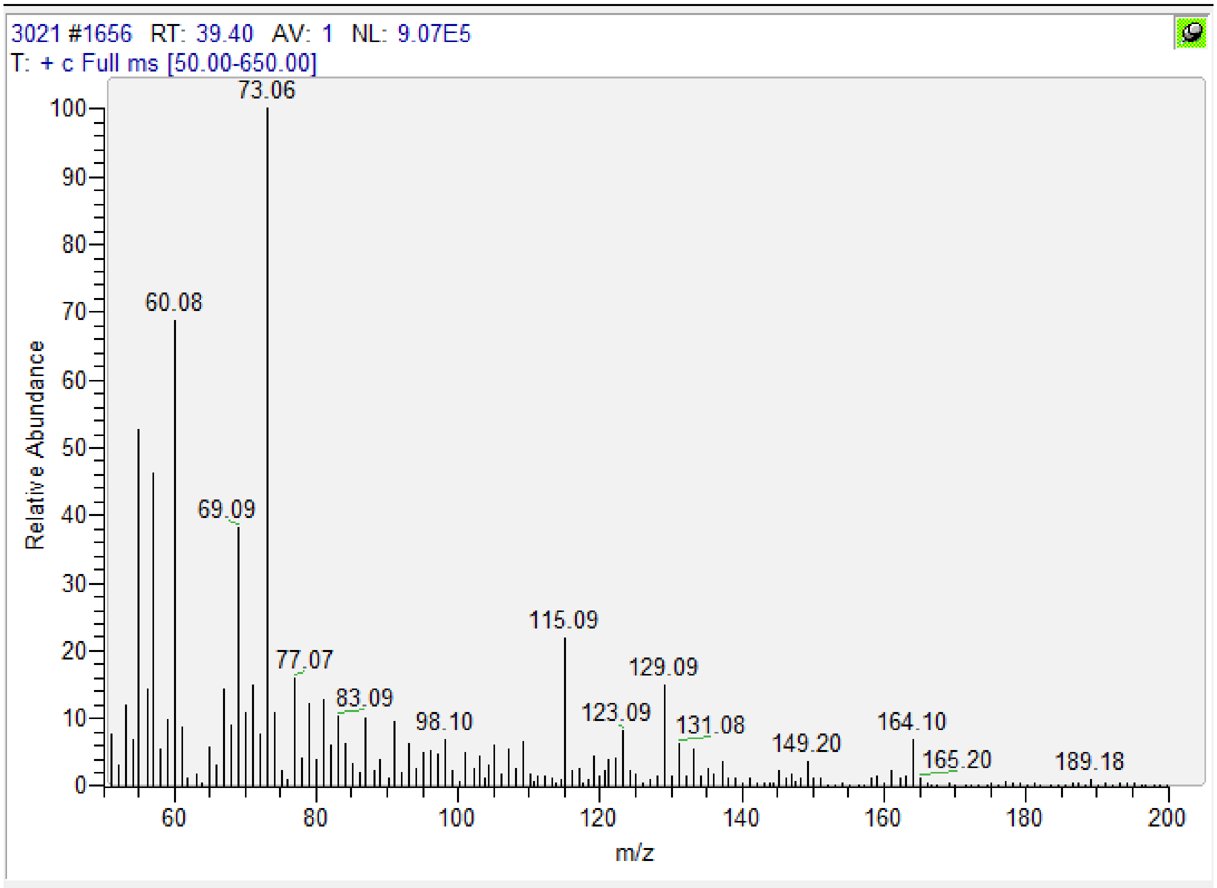

3021 #1656 RT: 39.40 AV: 1 NL: 9.07E5
T: + c Full ms [50.00-650.00]
Relative Abundance
m/z
73.06
60.08
69.09
77.07
83.09
98.10
115.09
123.09
129.09
131.08
149.20
164.10
165.20
189.18

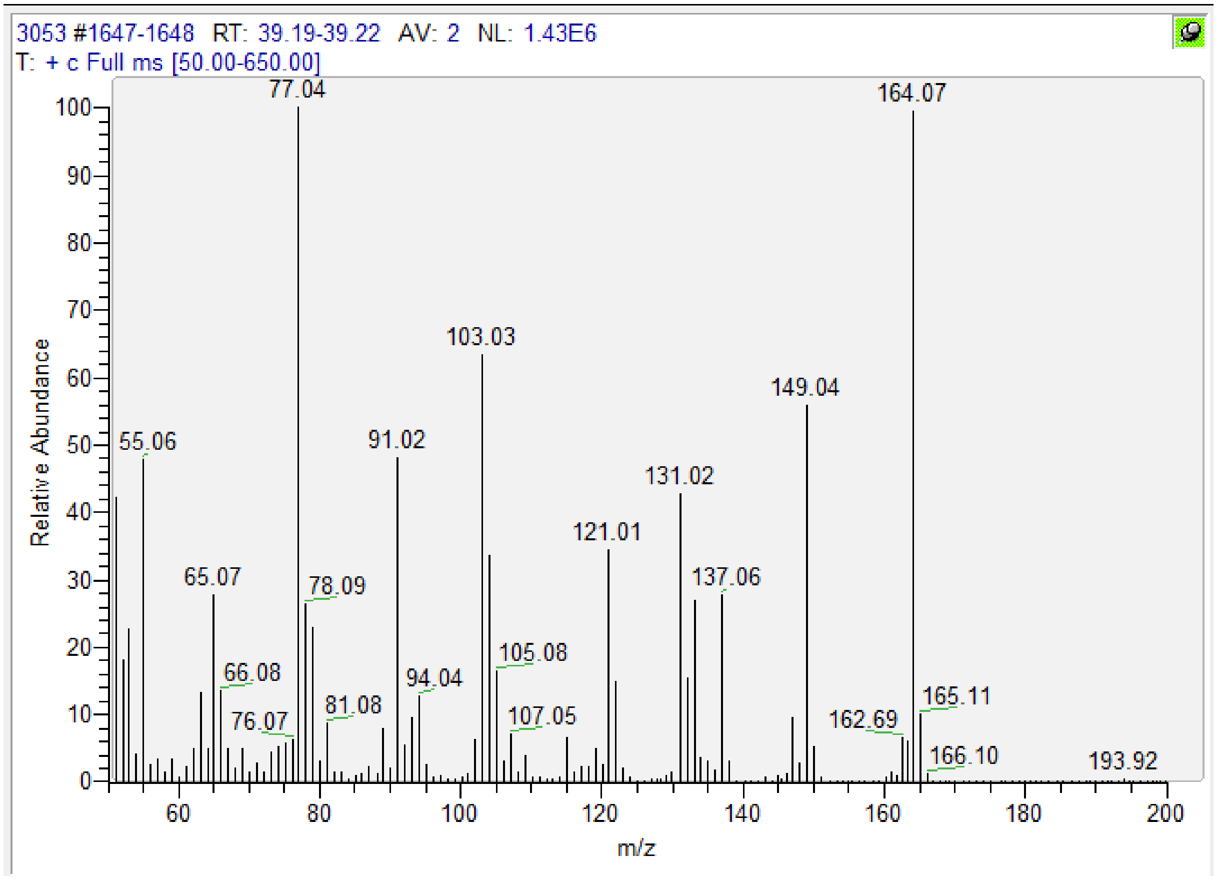
3053 #1647-1648 RT: 39.19-39.22 AV: 2 NL: 1.43E6
T: + c Full ms [50.00-650.00]
Relative Abundance
m/z
77.04
164.07
103.03
149.04
55.06
91.02
131.02
121.01
65.07
78.09
137.06
105.08
66.08
94.04
81.08
165.11
76.07
107.05
162.69
166.10
193.92

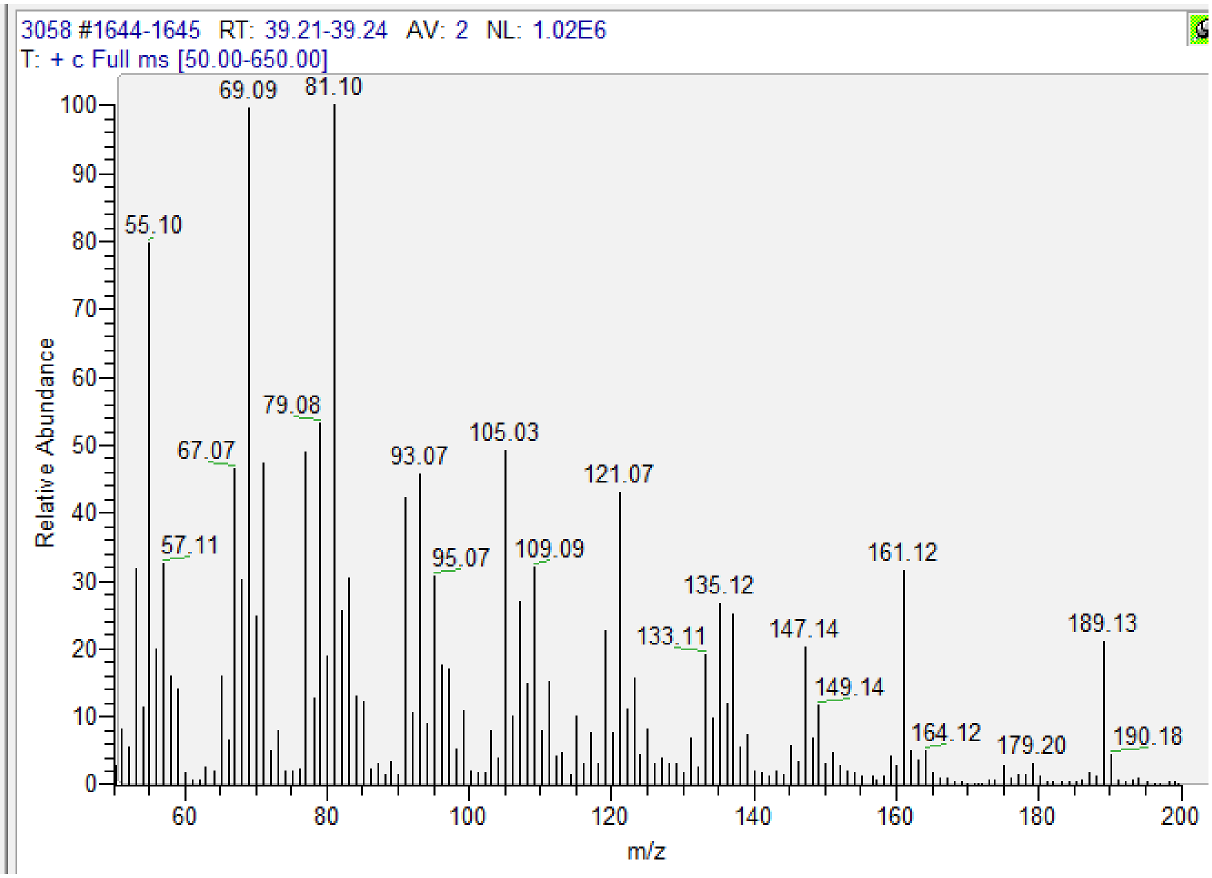

3058 #1644-1645 RT: 39.21-39.24 AV: 2 NL: 1.02E6
T: + c Full ms [50.00-650.00]
Relative Abundance
m/z
55.10
57.11
67.07
69.09
79.08
81.10
93.07
95.07
105.03
109.09
121.07
133.11
135.12
147.14
149.14
161.12
164.12
179.20
189.13
190.18

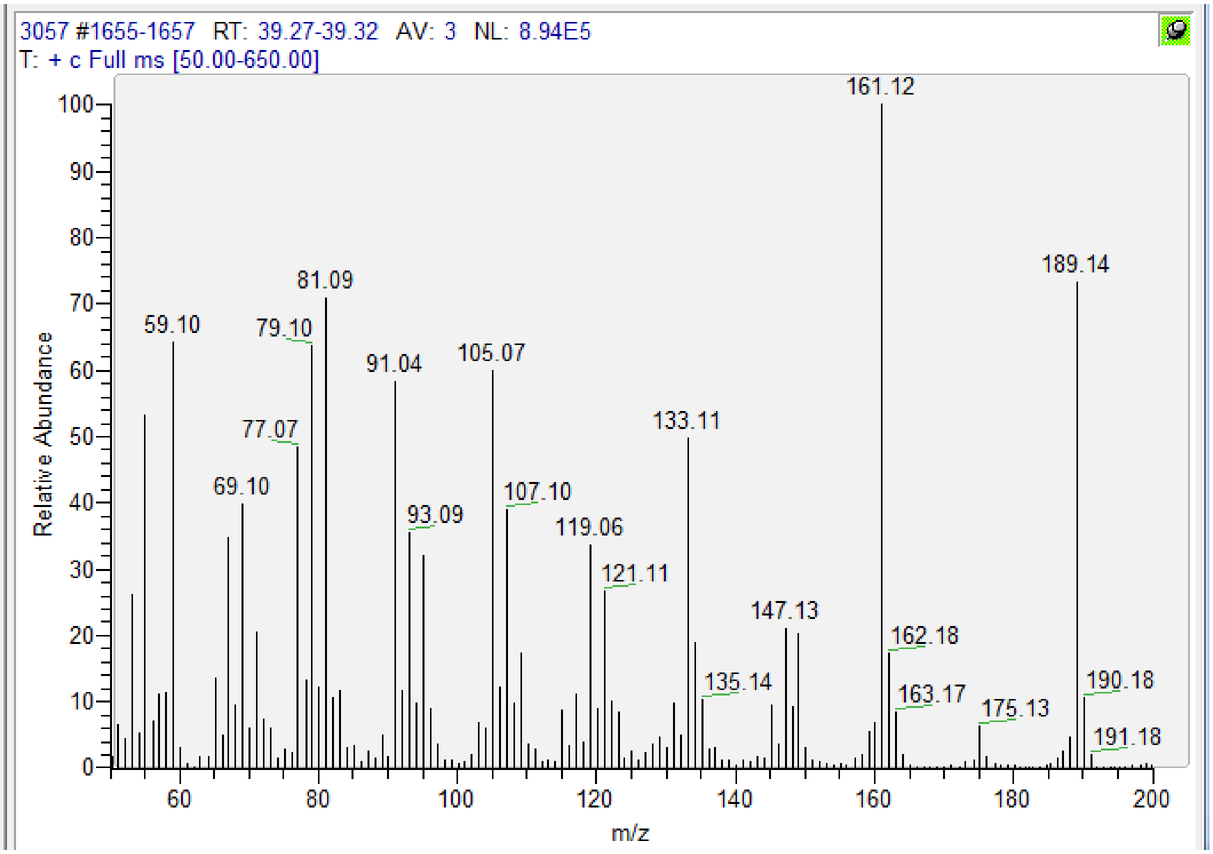
3057 #1655-1657 RT: 39.27-39.32 AV: 3 NL: 8.94E5
T: + c Full ms [50.00-650.00]
Relative Abundance
m/z
161.12
189.14
81.09
59.10
79.10
91.04
105.07
77.07
133.11
69.10
107.10
93.09
119.06
121.11
147.13
162.18
135.14
163.17
175.13
190.18
191.18

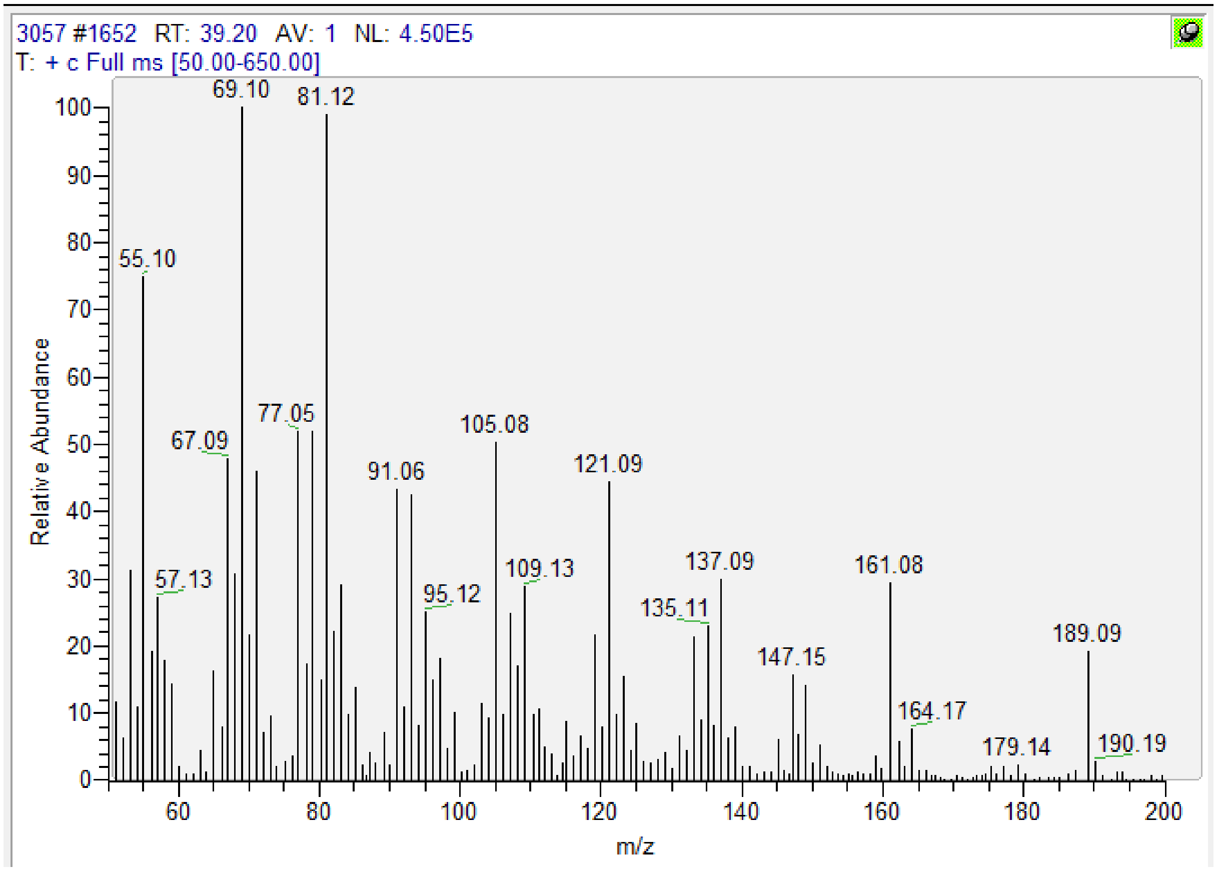

3057 #1652 RT: 39.20 AV: 1 NL: 4.50E5
T: + c Full ms [50.00-650.00]
Relative Abundance
m/z
69.10
81.12
55.10
67.09
77.05
105.08
91.06
121.09
57.13
95.12
109.13
137.09
135.11
161.08
147.15
189.09
164.17
179.14
190.19

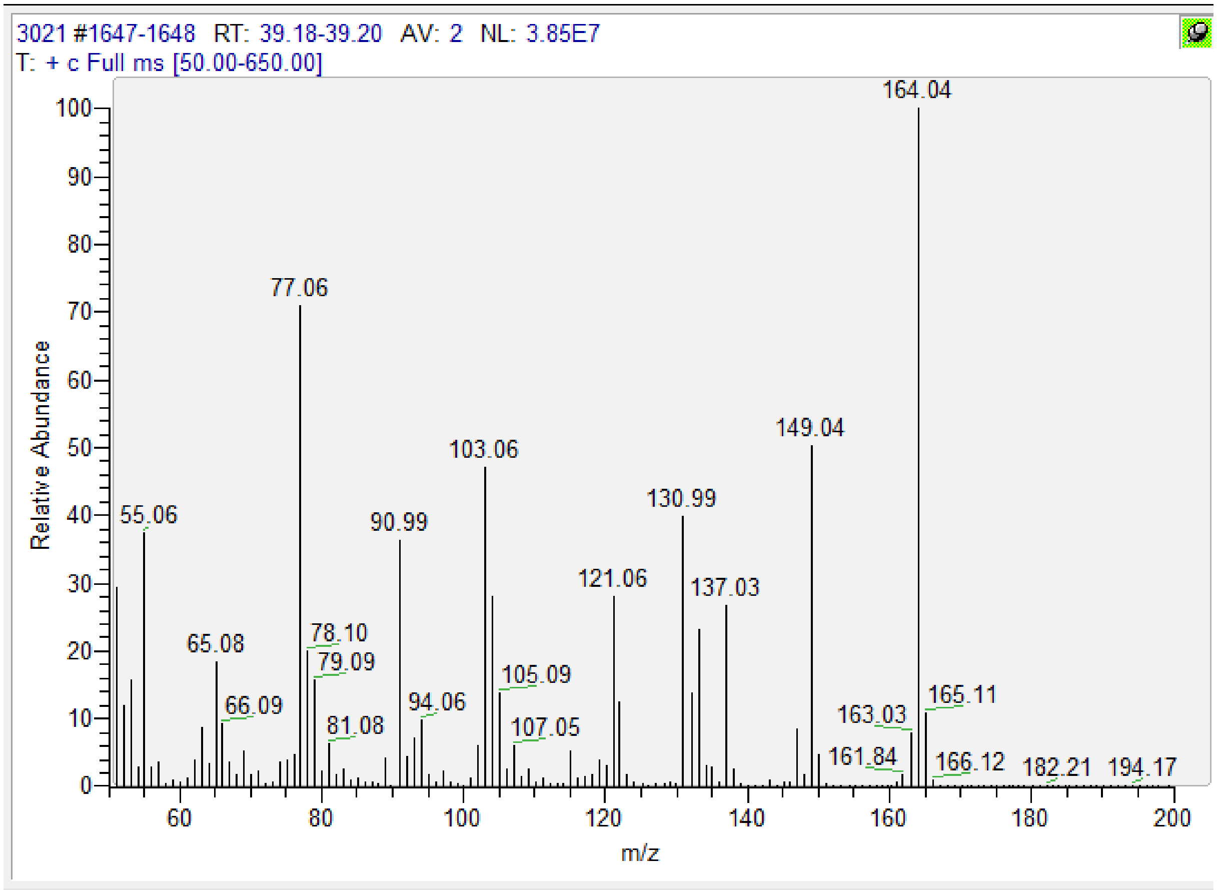

3021 #1647-1648 RT: 39.18-39.20 AV: 2 NL: 3.85E7
T: + c Full ms [50.00-650.00]
Relative Abundance
m/z
164.04
77.06
149.04
103.06
130.99
55.06
90.99
121.06
137.03
65.08
78.10
79.09
105.09
165.11
66.09
94.06
163.03
81.08
107.05
161.84
166.12
182.21
194.17

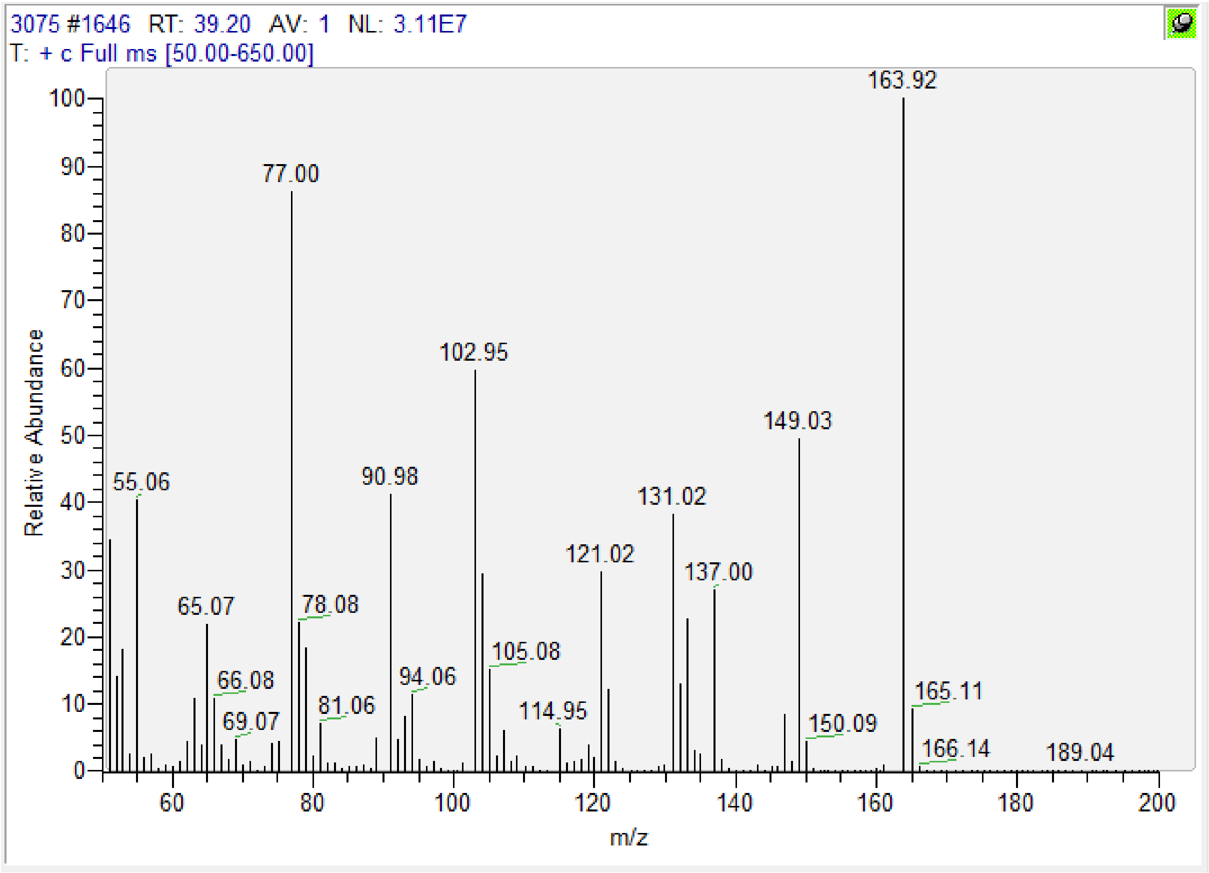
3075 #1646 RT: 39.20 AV: 1 NL: 3.11E7
T: + c Full ms [50.00-650.00]
Relative Abundance
m/z
163.92
77.00
102.95
149.03
55.06
90.98
131.02
121.02
137.00
65.07
78.08
105.08
94.06
66.08
81.06
114.95
69.07
150.09
165.11
166.14
189.04
0
10
20
30
40
50
60
70
80
90
100
60
80
100
120
140
160
180
200

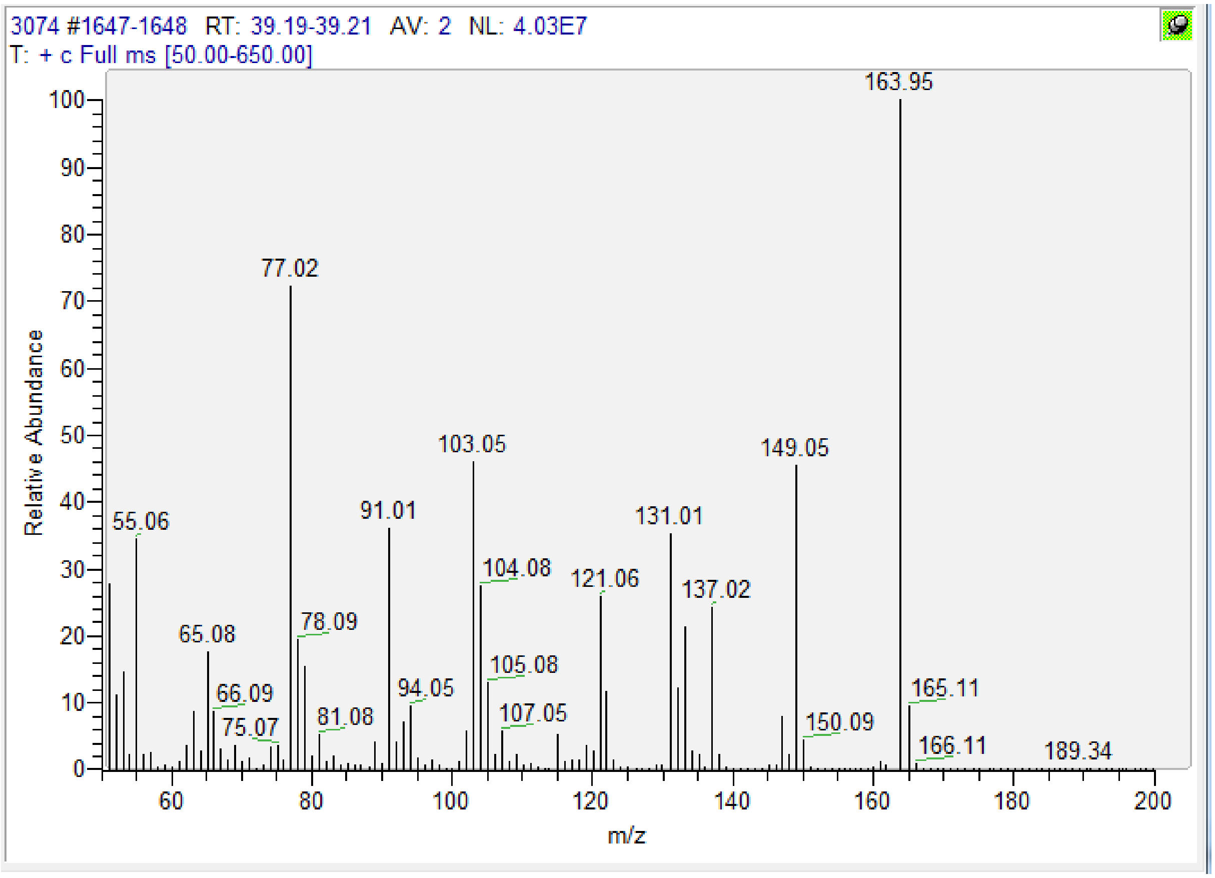

3074 #1647-1648 RT: 39.19-39.21 AV: 2 NL: 4.03E7
T: + c Full ms [50.00-650.00]
Relative Abundance
m/z
163.95
77.02
103.05
149.05
91.01
131.01
55.06
104.08
121.06
137.02
78.09
65.08
105.08
66.09
94.05
165.11
107.05
75.07
81.08
150.09
166.11
189.34

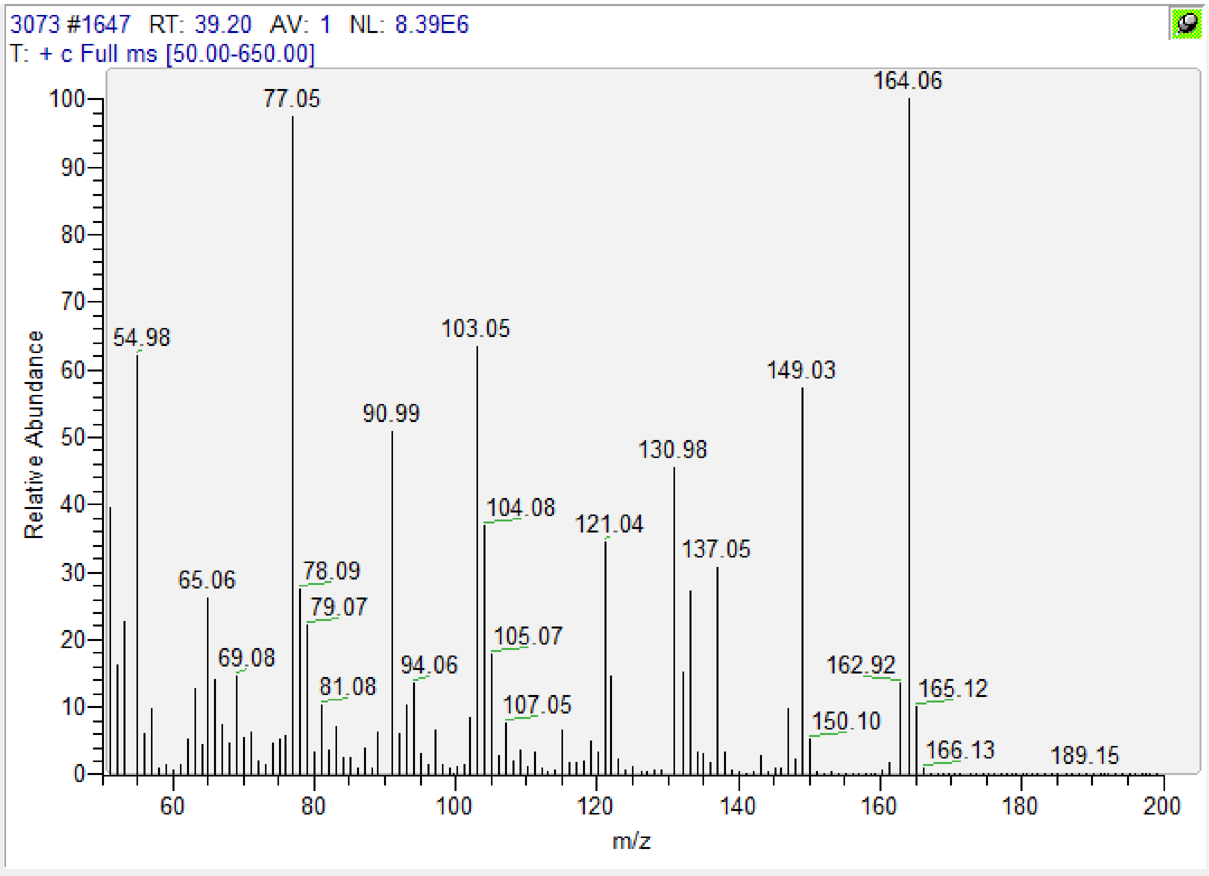

3073 #1647 RT: 39.20 AV: 1 NL: 8.39E6
T: + c Full ms [50.00-650.00]
Relative Abundance
m/z
164.06
77.05
54.98
103.05
149.03
90.99
130.98
104.08
121.04
137.05
65.06
78.09
79.07
105.07
69.08
94.06
162.92
81.08
165.12
107.05
150.10
166.13
189.15

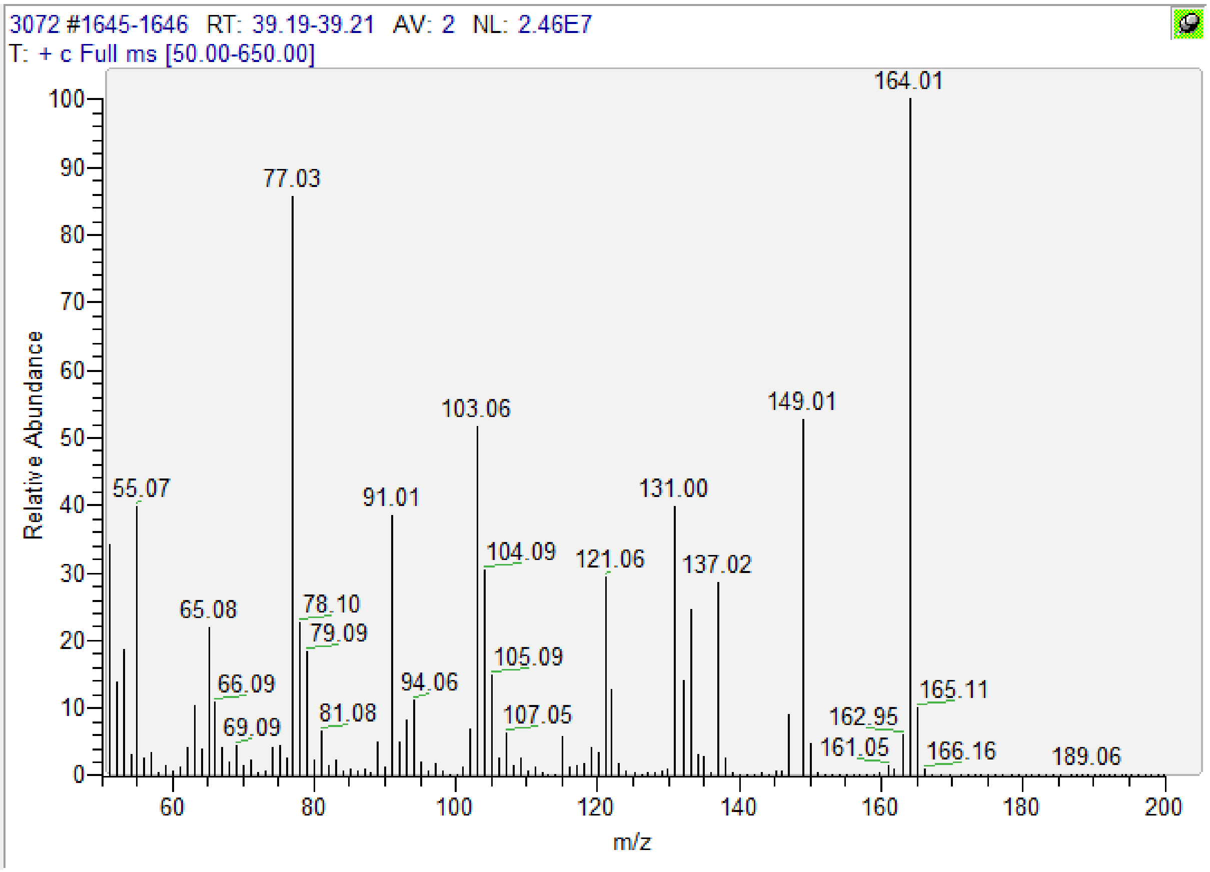
3072 #1645-1646 RT: 39.19-39.21 AV: 2 NL: 2.46E7
T: + c Full ms [50.00-650.00]
Relative Abundance
m/z
164.01
77.03
103.06
149.01
55.07
91.01
131.00
104.09
121.06
137.02
65.08
78.10
79.09
105.09
66.09
94.06
165.11
81.08
107.05
162.95
69.09
161.05
166.16
189.06

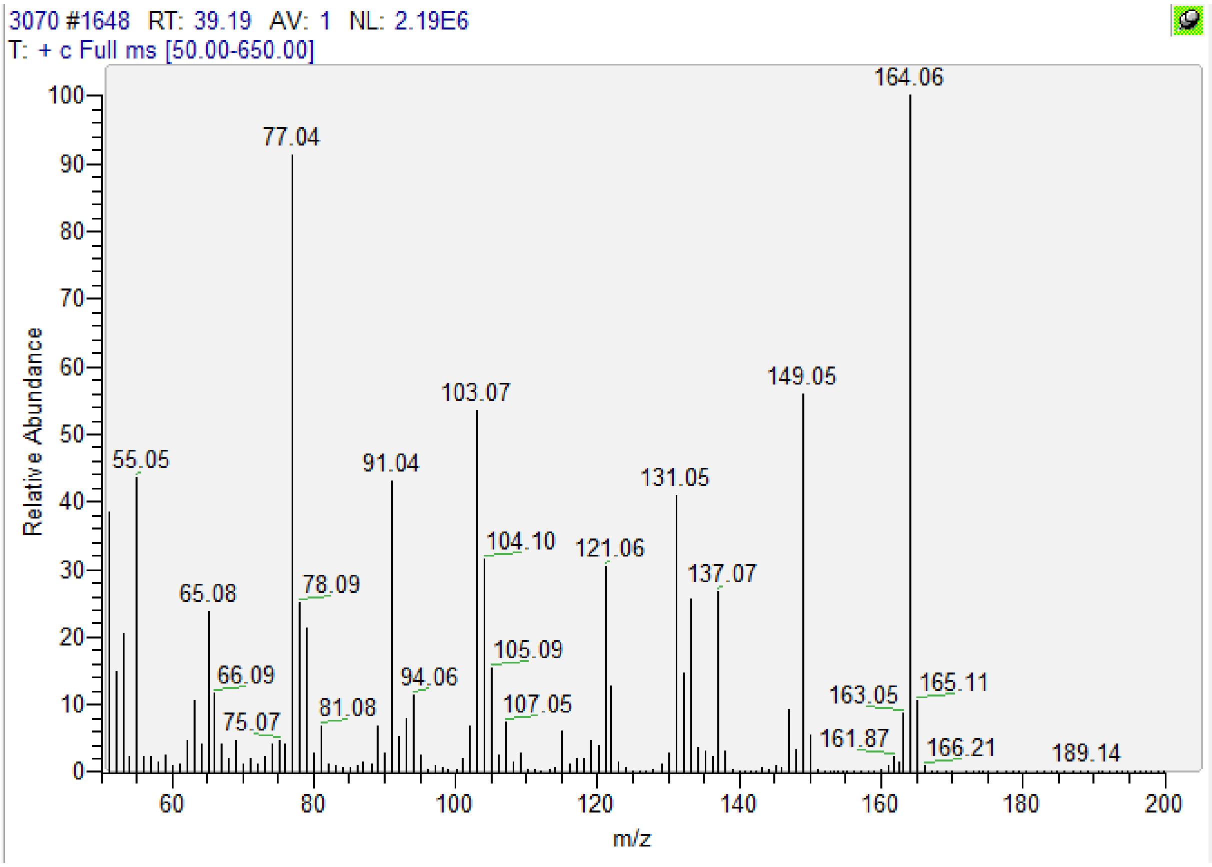
3070 #1648 RT: 39.19 AV: 1 NL: 2.19E6
T: + c Full ms [50.00-650.00]
Relative Abundance
m/z
164.06
77.04
149.05
103.07
55.05
91.04
131.05
104.10
121.06
137.07
78.09
65.08
105.09
94.06
66.09
165.11
163.05
107.05
81.08
75.07
161.87
166.21
189.14

Farnesol

Reference:

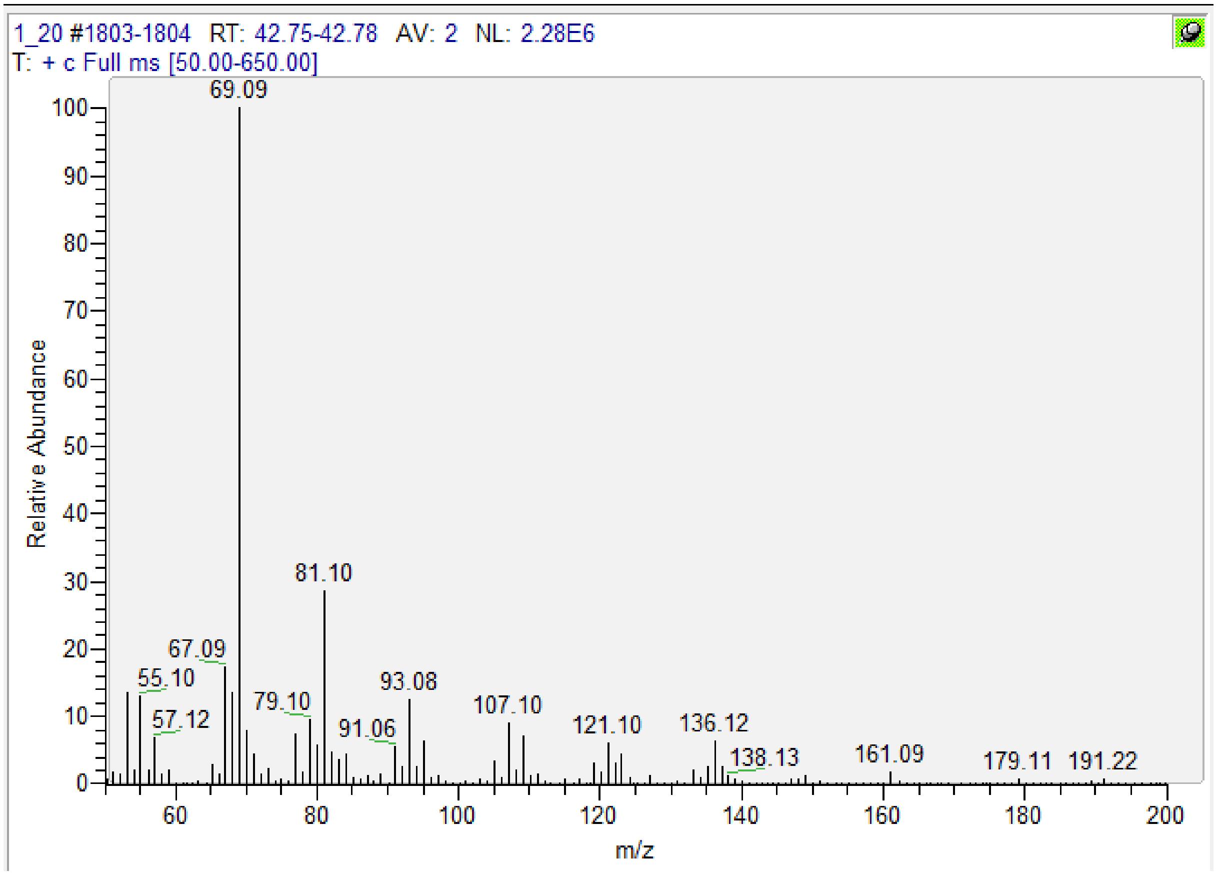

Samples:

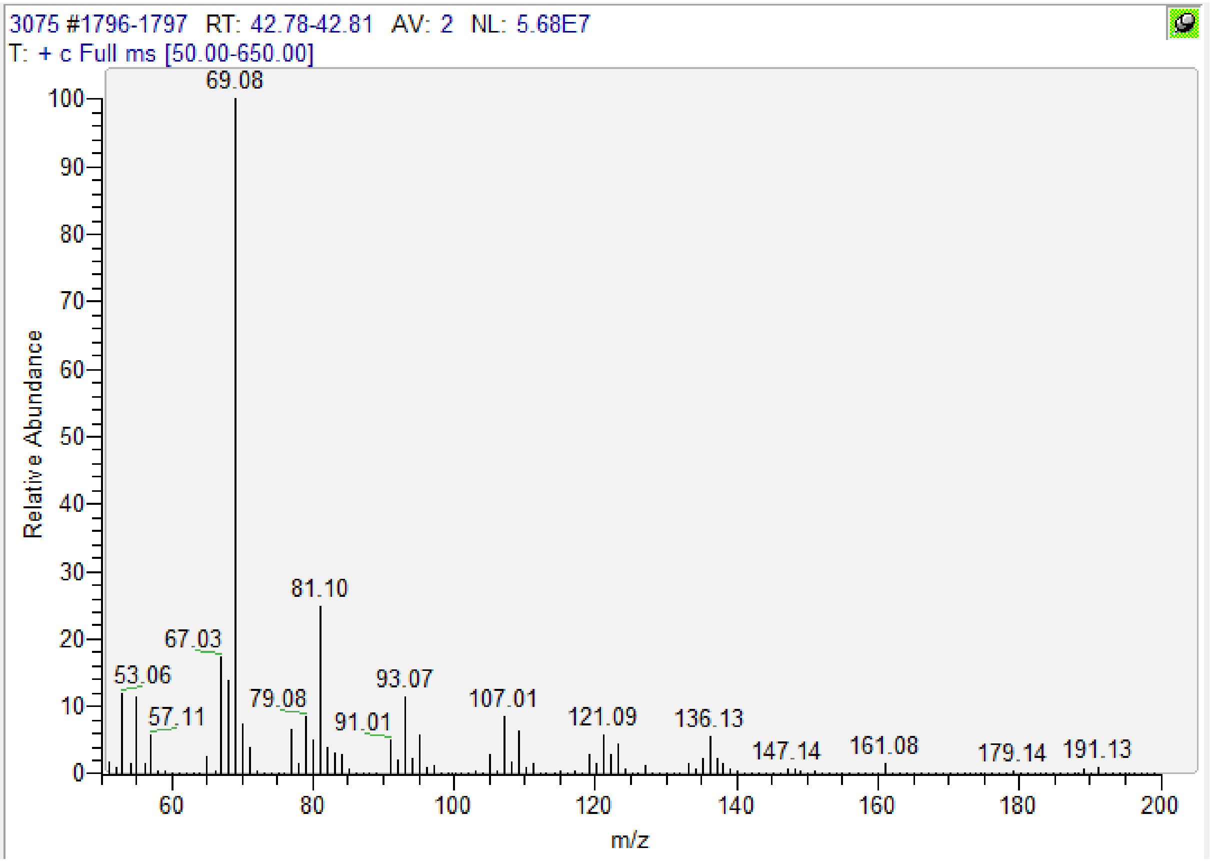

3075 #1796-1797 RT: 42.78-42.81 AV: 2 NL: 5.68E7
T: + c Full ms [50.00-650.00]
Relative Abundance
m/z
69.08
81.10
67.03
53.06
57.11
79.08
91.01
93.07
107.01
121.09
136.13
147.14
161.08
179.14
191.13

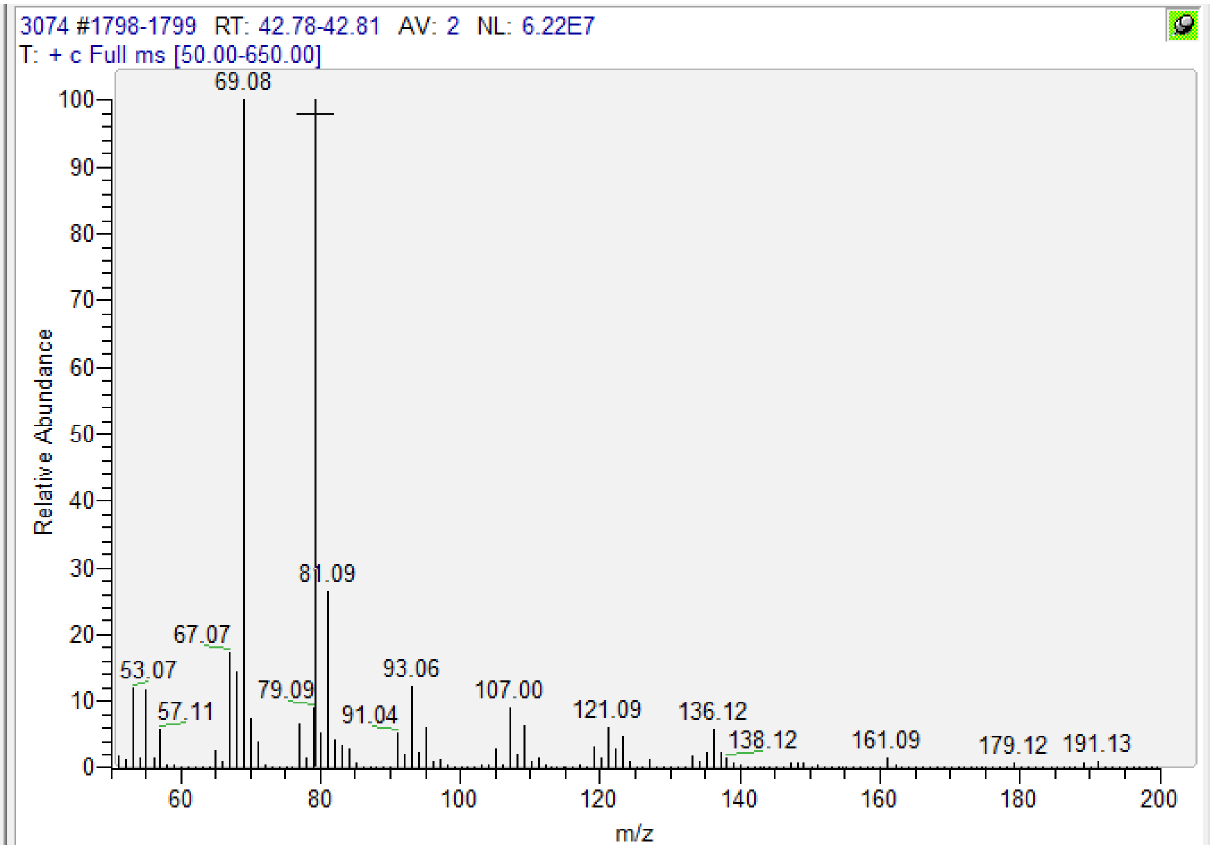

3074 #1798-1799 RT: 42.78-42.81 AV: 2 NL: 6.22E7
T: + c Full ms [50.00-650.00]
Relative Abundance
m/z
69.08
81.09
67.07
53.07
57.11
79.09
91.04
93.06
107.00
121.09
136.12
138.12
161.09
179.12
191.13

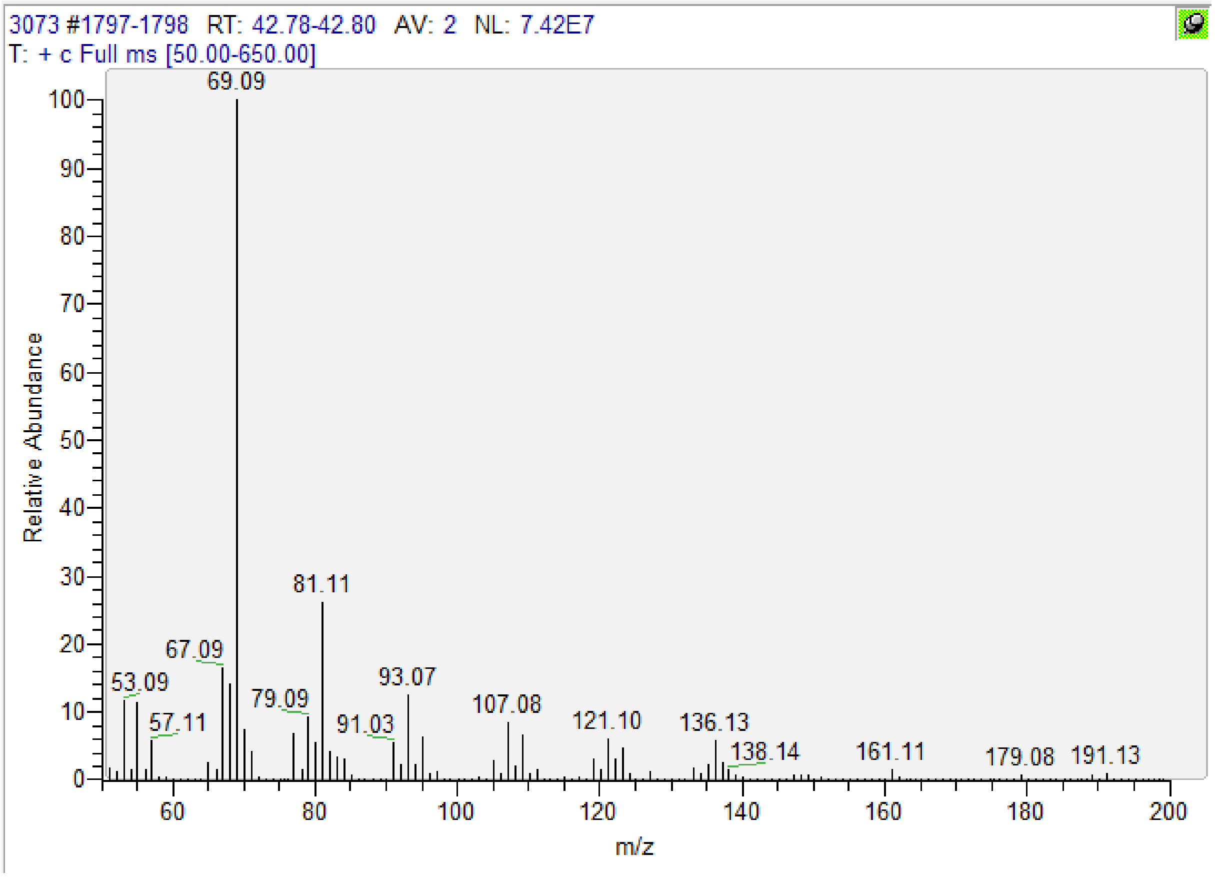
3073 #1797-1798 RT: 42.78-42.80 AV: 2 NL: 7.42E7
T: + c Full ms [50.00-650.00]
Relative Abundance
m/z
69.09
81.11
67.09
53.09
57.11
79.09
91.03
93.07
107.08
121.10
136.13
138.14
161.11
179.08
191.13

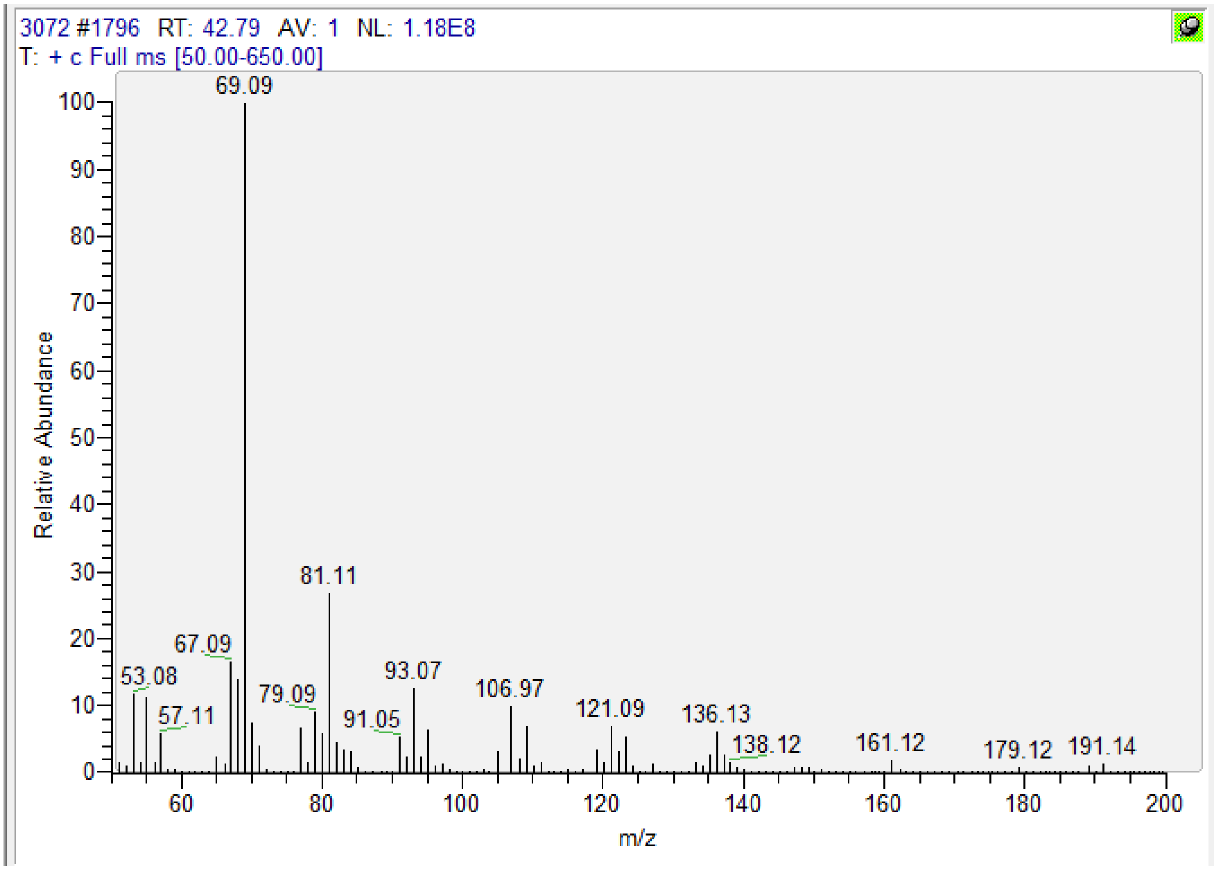

3072 #1796 RT: 42.79 AV: 1 NL: 1.18E8
T: + c Full ms [50.00-650.00]
Relative Abundance
m/z
69.09
81.11
67.09
53.08
57.11
79.09
91.05
93.07
106.97
121.09
136.13
138.12
161.12
179.12
191.14

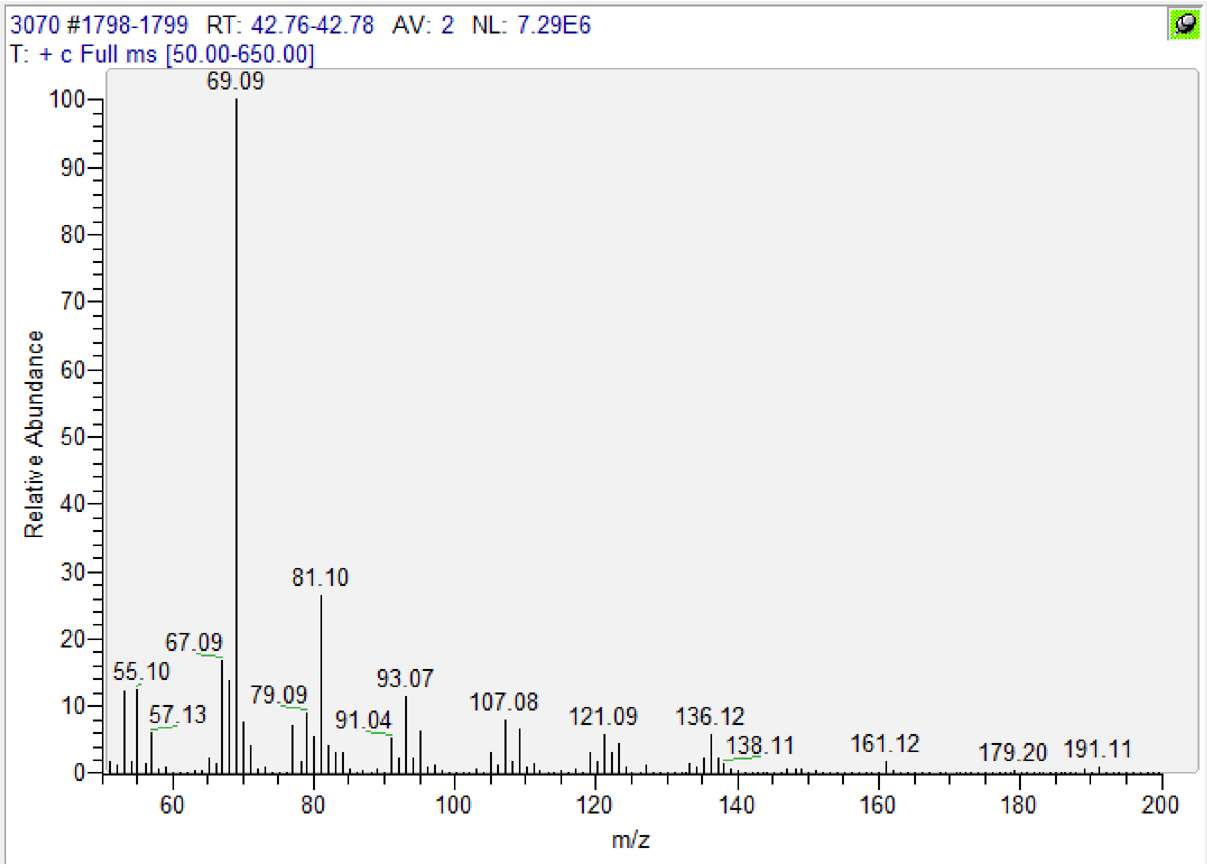
3070 #1798-1799 RT: 42.76-42.78 AV: 2 NL: 7.29E6
T: + c Full ms [50.00-650.00]
Relative Abundance
m/z
69.09
81.10
67.09
55.10
57.13
79.09
91.04
93.07
107.08
121.09
136.12
138.11
161.12
179.20
191.11

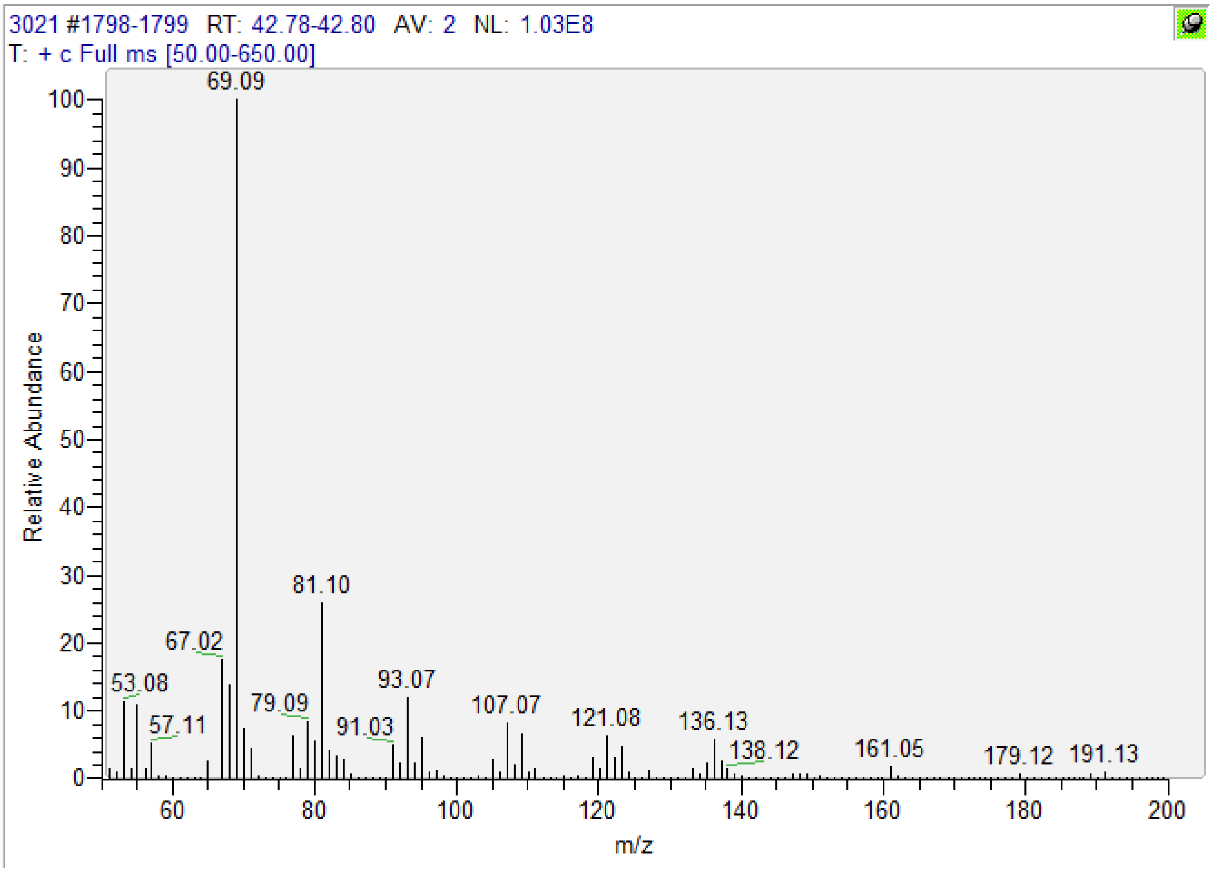
3021 #1798-1799 RT: 42.78-42.80 AV: 2 NL: 1.03E8
T: + c Full ms [50.00-650.00]
69.09
81.10
67.02
53.08
57.11
79.09
91.03
93.07
107.07
121.08
136.13
138.12
161.05
179.12
191.13
Relative Abundance
m/z

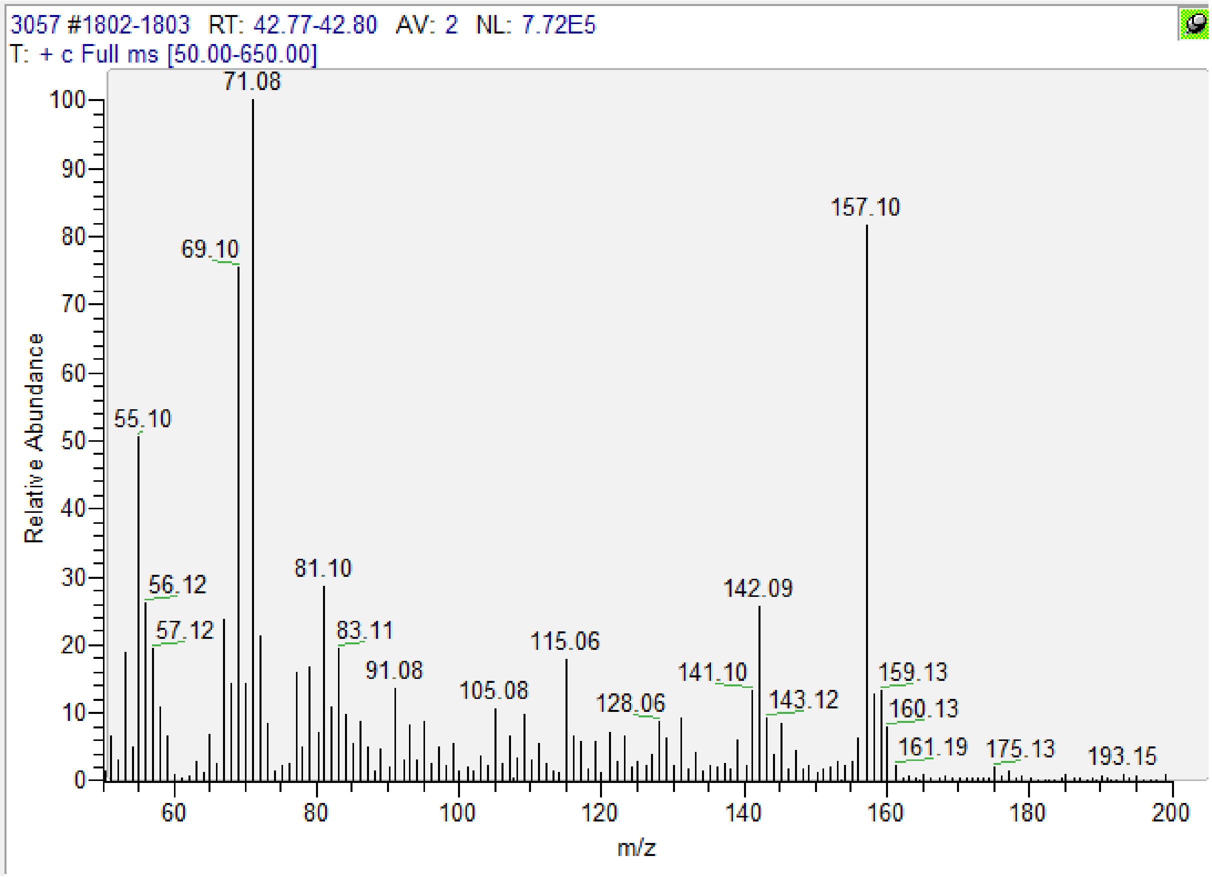
3057 #1802-1803 RT: 42.77-42.80 AV: 2 NL: 7.72E5
T: + c Full ms [50.00-650.00]
Relative Abundance
m/z
71.08
69.10
55.10
56.12
57.12
81.10
83.11
91.08
105.08
115.06
128.06
141.10
142.09
143.12
157.10
159.13
160.13
161.19
175.13
193.15

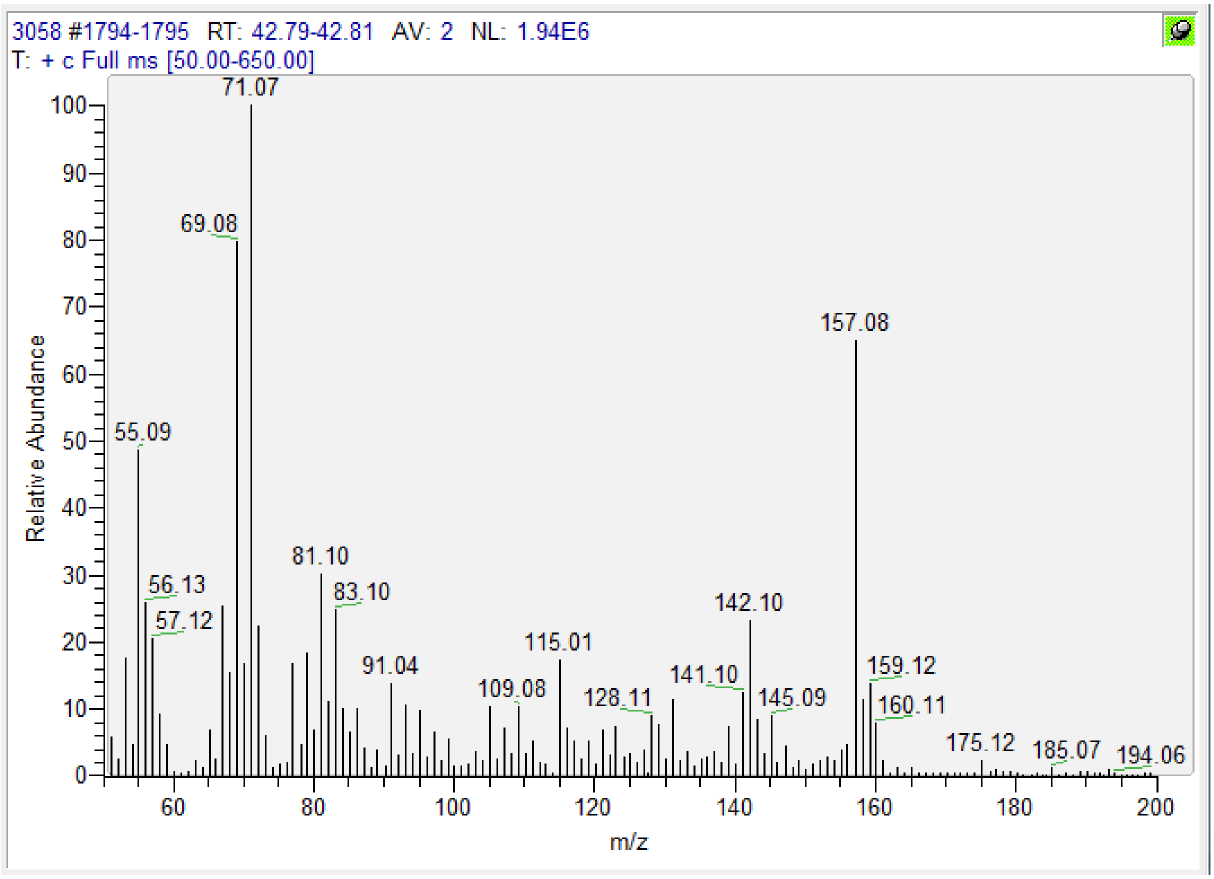

3058 #1794-1795 RT: 42.79-42.81 AV: 2 NL: 1.94E6
T: + c Full ms [50.00-650.00]
Relative Abundance
m/z
71.07
69.08
157.08
55.09
81.10
56.13
83.10
57.12
142.10
115.01
91.04
159.12
141.10
109.08
128.11
145.09
160.11
175.12
185.07
194.06

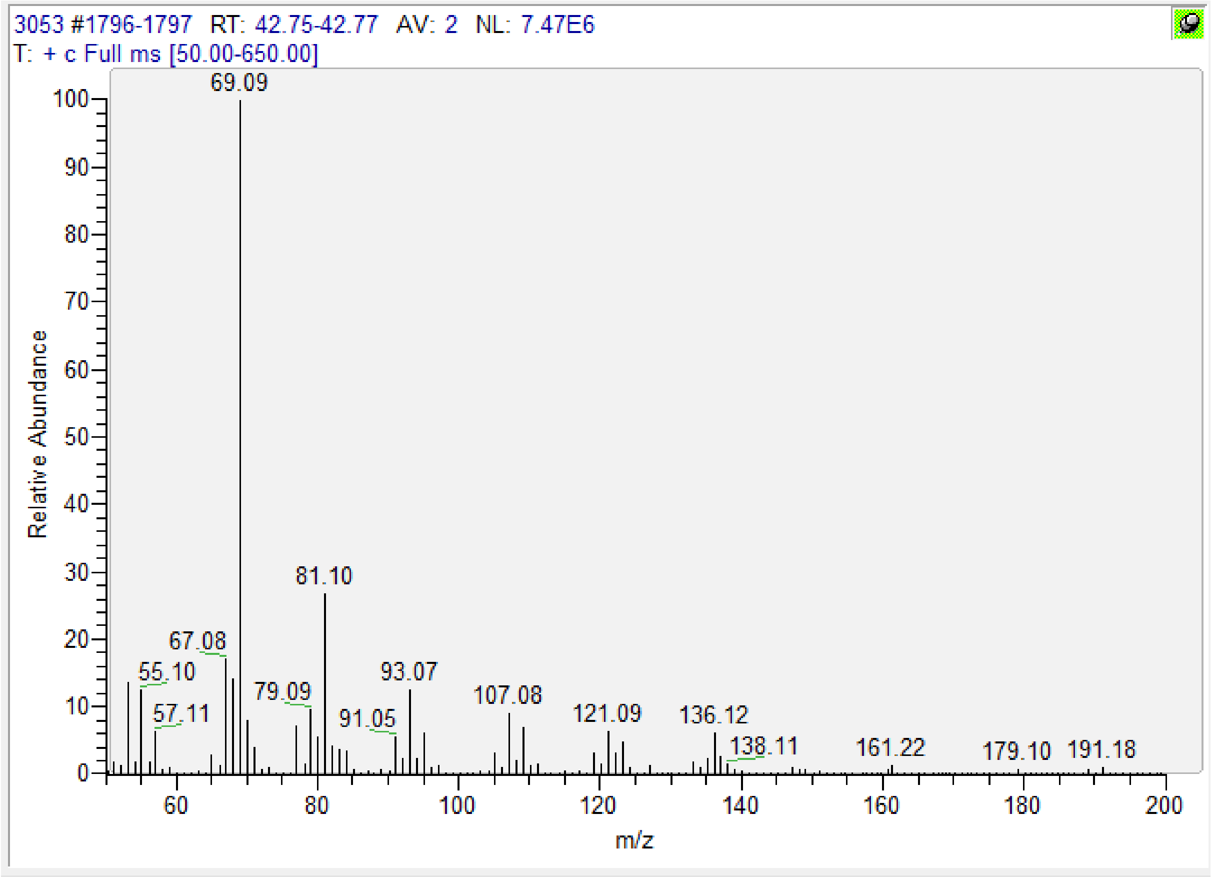

3053 #1796-1797 RT: 42.75-42.77 AV: 2 NL: 7.47E6
T: + c Full ms [50.00-650.00]
69.09
81.10
67.08
55.10
57.11
79.09
91.05
93.07
107.08
121.09
136.12
138.11
161.22
179.10
191.18
Relative Abundance
m/z

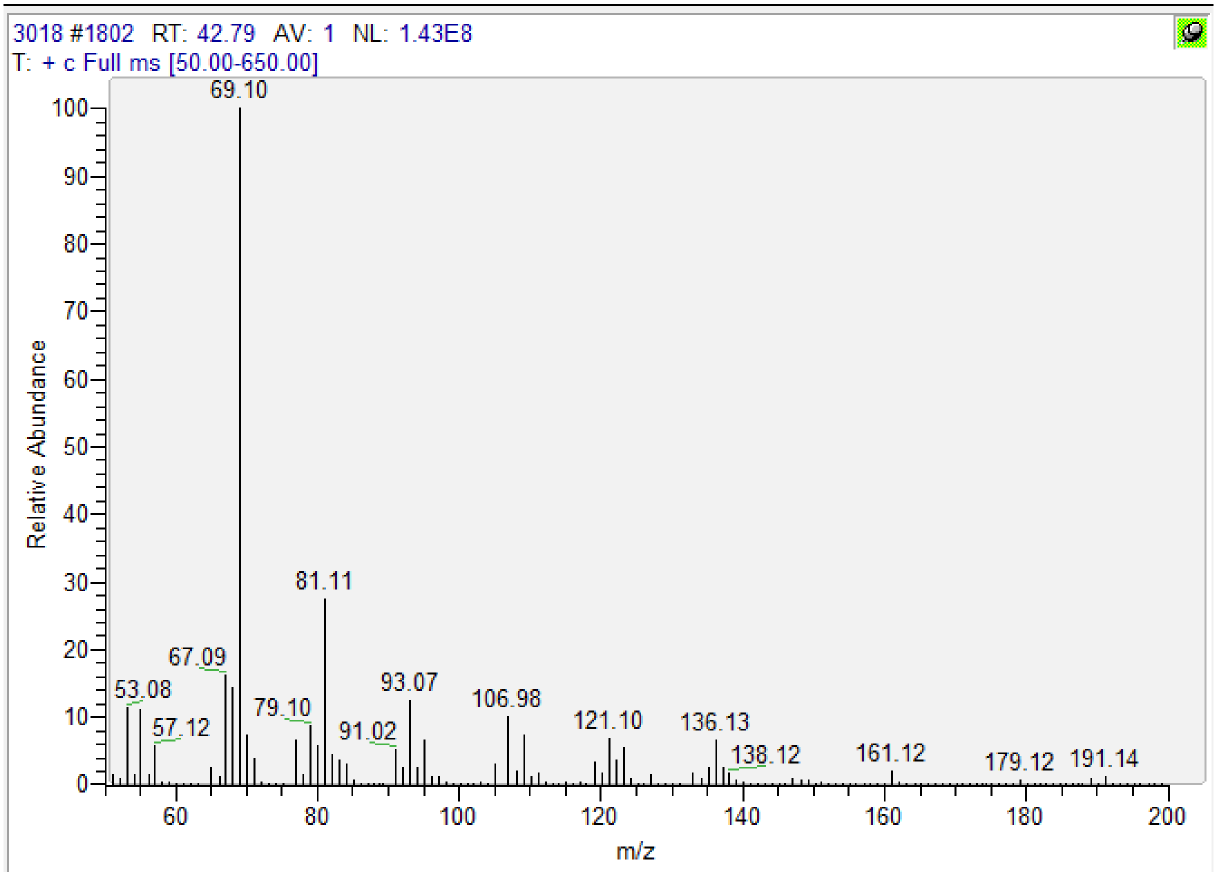

3018 #1802 RT: 42.79 AV: 1 NL: 1.43E8
T: + c Full ms [50.00-650.00]
Relative Abundance
m/z
69.10
81.11
67.09
53.08
57.12
79.10
91.02
93.07
106.98
121.10
136.13
138.12
161.12
179.12
191.14
